# Genome wide *in silico* analysis of *Plasmodium falciparum* phosphatome

**DOI:** 10.1186/1471-2164-15-1024

**Published:** 2014-11-25

**Authors:** Rajan Pandey, Asif Mohmmed, Christine Pierrot, Jamal Khalife, Pawan Malhotra, Dinesh Gupta

**Affiliations:** Structural and Computational Biology group, International Centre for Genetic Engineering and Biotechnology, Aruna Asaf Ali Marg, New Delhi, 110067 India; Malaria Group, International Centre for Genetic Engineering and Biotechnology, Aruna Asaf Ali Marg, New Delhi, 110067 India; Center for Infection and Immunity of Lille, Inserm U1019, CNRS, Institut Pasteur de Lille, Univ Lille Nord de France, 1 rue du Professeur Calmette, Lille cedex, 59019 France

**Keywords:** Posttranslational modifications, PTM, PFAM, CDD, Phosphatome, Phosphatase, Dephosphorylation

## Abstract

**Background:**

Eukaryotic cellular machineries are intricately regulated by several molecular mechanisms involving transcriptional control, post-translational control and post-translational modifications of proteins (PTMs). Reversible protein phosphorylation/dephosphorylation process, which involves kinases as well as phosphatases, represents an important regulatory mechanism for diverse pathways and systems in all organisms including human malaria parasite, *Plasmodium falciparum.* Earlier analysis on *P. falciparum* protein-phosphatome revealed presence of 34 phosphatases in *Plasmodium* genome. Recently, we re-analysed *P. falciparum* phosphatome aimed at identifying parasite specific phosphatases.

**Results:**

*Plasmodium* database (PlasmoDB 9.2) search, combined with PFAM and CDD searches, revealed 67 candidate phosphatases in *P. falciparum*. While this number is far less than the number of phosphatases present in *Homo sapiens*, it is almost the same as in other *Plasmodium* species. These *Plasmodium* phosphatase proteins were classified into 13 super families based on NCBI CDD search. Analysis of proteins expression profiles of the 67 phosphatases revealed that 44 phosphatases are expressed in both schizont as well as gametocytes stages. Fourteen phosphatases are common in schizont, ring and trophozoite stages, four phosphatases are restricted to gametocytes, whereas another three restricted to schizont stage. The phylogenetic trees for each of the known phosphatase super families reveal a considerable phylogenetic closeness amongst apicomplexan organisms and a considerable phylogenetic distance with other eukaryotic model organisms included in the study. The GO assignments and predicted interaction partners of the parasite phosphatases indicate its important role in diverse cellular processes.

**Conclusion:**

In the study presented here, we reviewed the *P. falciparum* phosphatome to show presence of 67 candidate phosphatases in *P. falciparum* genomes/proteomes. Intriguingly, amongst these phosphatases, we could identify six *Plasmodium* specific phosphatases and 33 putative phosphatases that do not have human orthologs, thereby suggesting that these phosphatases have the potential to be explored as novel antimalarial drug targets.

**Electronic supplementary material:**

The online version of this article (doi:10.1186/1471-2164-15-1024) contains supplementary material, which is available to authorized users.

## Background

Malaria is still a serious public health problem in tropical and sub-tropical regions, causing large socio-economic loss in the affected countries. It infects around 219 million people across the world, taking toll of between 6,60,000 to 1.2 million lives each year, vast majority being young children [1]. Malaria has a complex life cycle and requires two hosts to complete it- vertebrate host (Human) where asexual stages of malaria parasite occur and invertebrate host (Anopheles mosquito) where sexual stages of malaria parasite occur. Malaria parasite mainly causes disease while undergoing asexual development and multiplication within human erythrocytes. A number of transcription research studies have emphasized that asexual stage development of *Plasmodium falciparum* is tightly regulated because of gene regulation by transcription factors, acetylation/methylation of genes as well as by protein Post Translational Modifications (PTMs) [2]. Recently, the specific applications of purification methods followed by highly sensitive mass spectrometric approaches and bioinformatics have allowed large-scale analysis of PTMs such as protein phosphorylation, ubiquitination and palmitoylation in *P. falciparum*[3–5].

The reversible phosphorylation of proteins is a major post-translational mechanism of regulation for diverse pathways and processes in the eukaryotic cells. This process is controlled by an intricate balance between the antagonistic activities of two class of proteins- kinases and phosphatases, performing phosphorylation and dephosphorylation respectively. These two enzymes predominantly act on serine, threonine, tyrosine or histidine residues [6]. Recent experiments indicate critical role of phosphorylation in egress, invasion and host cell remodelling by *Plasmodium* parasites [7–14]. For *P. falciparum, ~*8000 phosphopeptides corresponding to ~1,700 *Plasmodium* proteins have been identified [14]. A number of recent studies, using advances in phosphopeptides enrichment, followed by liquid chromatography-tandem mass spectrometry (LC-MS/MS) approaches have revealed the kinome of *P. falciparum* and identified approximately 100 eukaryotic protein kinases (ePKs) profoundly divergent to the mammalian kinases [14–17].

Although a number of *Plasmodium* phosphatases have been characterized in the past [18–21], only a single genome-wide study has illustrated the *Plasmodium* phosphatome [22]. This *in silico* bioinformatics analysis identified 27 protein phosphatase (PPs) subunits encoded in the *Plasmodium* genome [22]. These PPs were classified into four broad families of protein phosphatases; phospho-protein phosphatases (PPP) group, PPM group, protein tyrosine phosphatase (PTP) group and Interacting factor like phosphatase (NIF) group with distinct evolutionary history. The authors also reported presence of rhodanese homology domain (RHOD) and protein tyrosine phosphatase like protein (PTPLA) in *P. falciparum.* While preparing this manuscript, a genome wide functional analysis of *P. berghei* protein phosphatases is published studying its role in parasite development and differentiation [23]. Gene disruption analysis reveals that half of the *P. berghei* protein phosphatases are essential for the parasite growth. Historically, protein kinases have been studied more vigorously for antimalarial and drug discovery as compared to protein phosphatases due to an early view that protein kinases confer fine protein regulation by phosphorylation, whereas phosphatases just remove phosphates without any regulation. However various studies in the last two-decade indicate that phosphatases are also regulated by various mechanisms and play as important role as protein kinases [24–26]. In contrast to a large number of kinase catalytic subunits, phosphatases catalytic subunits are much lower in number. Phosphatases are in general less discriminating than the most of the kinases in substrate selectivity but have very high catalytic efficiency making it highly toxic for the cells in active form [27]. A number of accessory proteins called phosphatase regulatory subunits controls the specificity and regulation of most phosphatases [22], bringing about an element of specificity in its actions. In the present study, we re-examined the *Plasmodium* phosphatome using PlasmoDB database [28] and conserved domain database (CDD) analysis. The results showed identification of sixty seven putative phosphatase sequences in the *Plasmodium* genome, which is significantly higher than previously reported [22]. Amongst the identified putative phosphatase sequences, thirty-three do not have human orthologs and six of them are *Plasmodium* specific (Additional file 1)*.* Profound divergence between the phosphatome of malaria parasites from all major eukaryotic phyla suggests potential of parasite specific phosphatases as new drug targets for antimalarial discovery.

## Results and discussion

**Figure 1:**
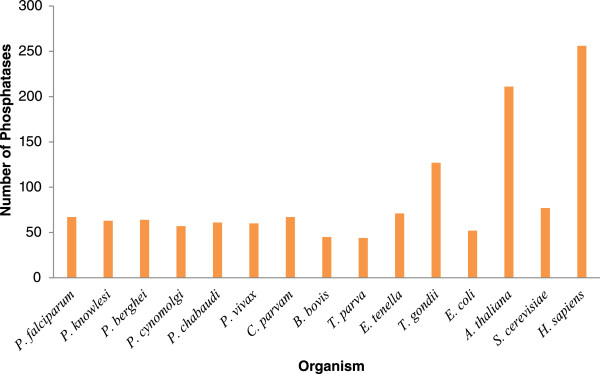
**Summary of protein phosphatomes of model organisms selected for the comparative studies.**

Using the computational methods described in the Methods section, we collected and screened the predicted proteomes of *Plasmodium falciparum, Plasmodium vivax, Plasmodium knowlesi, Plasmodium cynomolgi, Plasmodium berghei, Plasmodium chabaudi, Eimeria tenella, Toxoplasma gondii, Babesia bovis, Theileria parva, Cryptosporidium parvam, Escherichia coli, Arabidopsis thaliana, Saccharomyces cerevisiae* and *Homo sapiens* for phosphatase sequences. We used text search as well as complete proteome of *P. falciparum* and for others organisms only text search was performed to retrieve phosphatase specific sequences as the main focus of our study was to analyze the phosphatases present in human malaria parasite, *P. falciparum.* We selected 1322 phosphatase sequences from these model organism proteomes for further analysis*.* We could identify 67 and 56 phosphatase sequences in *P. falciparum* based on CDD [29] and PFAM [30] classifications, respectively. Using CDD search, almost similar numbers of phosphatase sequences were identified in other *Plasmodium species* too. The apicomplexans included in the study have 44 – 71 phosphatases; with the exception of *T. gondii,* which has 127 candidate phosphatases identified using CDD approach. In comparison to ~250 phosphatases identified in *H. sapiens*, the number of phosphatases is much lower in *Plasmodium species* (Figure 1). Amongst the 67 candidate phosphatases identified in *Plasmodium* genome, only 34 phosphatases have human homologue, whereas six are *Plasmodium* specific*.* Human proteins containing phosphatase domain have been classified into six distinct functional and structural groups: protein tyrosine phosphatases (PTPs, 108 members), metal-dependent protein phosphatases (PPMs, 13 members), phosphoprotein phosphatases (PPPs, 15 members), lipid phosphatases (LPs, 37 members), haloacid dehalogenase (HADs, 21 members) and nucleoside-diphosphate-linked moiety X (NUDT, 5 members) [31]. Number and percentage of phosphatases belonging to each group varies among different organisms (Additional file 2). For example, the maximum number of *H. sapiens* phosphatases belongs to phosphotyrosine phosphatase family, whereas majority of the *A. thaliana* phosphatases belong to PP2Cc and MPP family.

**Figure 2:**
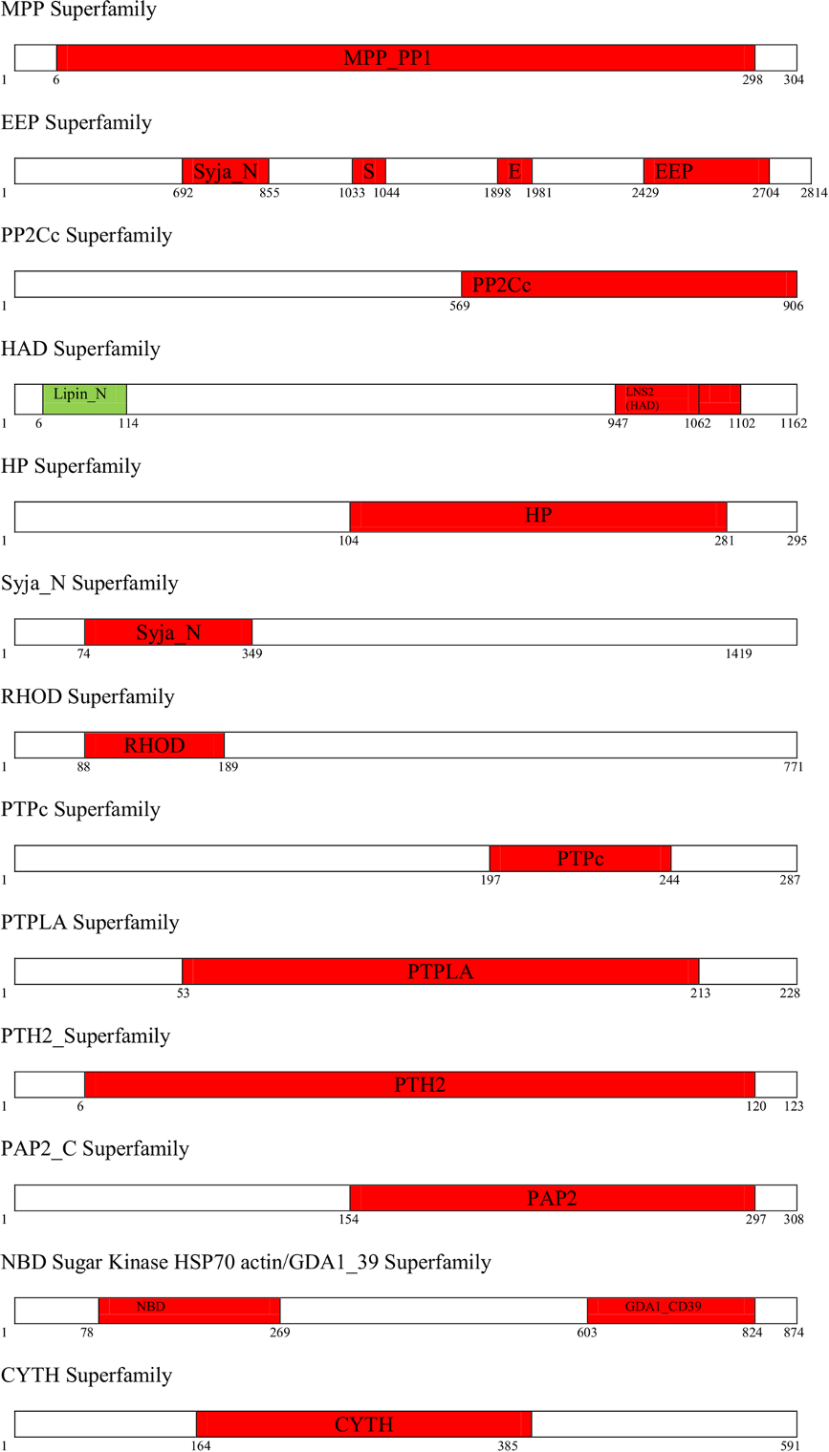
**Schematic diagram of phosphatases belonging to each phosphatase superfamily.**

**Figure 3:**
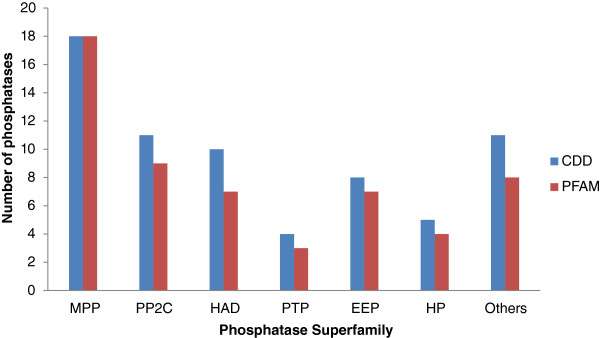
***P. falciparum*** **phosphatase domain classification on the basis of PFAM and CDD.**

Based on the classification adapted for human phosphatases and considering the other functional phosphatase domain, we grouped *Plasmodium* phosphatases into 13 super families using CDD. Schematic diagram of phosphatases in each superfamily is shown in Figure 2 (Additional file 3). Conserved domain alignment of *P. falciparum* phosphatases are shown in Additional files 4 and 5. Table 1 shows the name, accession number and classes of *Plasmodium* phosphatases analysed in the present study. Identity and the number of phosphatases observed in each family remained proportionally similar in the CDD or PFAM domain classifications (Figure 3). The protein family assignment of the phosphatases revealed interesting patterns due to the reason that we used a broader selection criterion as compared to previous studies, enabling identification of complete spectrum of phosphatases. The phosphatase family with highest number of *Plasmodium* phosphatases is the MPP family with 18 phosphatases, whereas few families such as PTPLA (Protein tyrosine phosphatase-like protein) and CYTH-like_Pase (Triphosphate Tunnel Metaloenzyme Phosphatase) have only single member. Several sequences analysed in the study have more than one conserved phosphatase domain, for example PF3D7_1466100 has MPP as well as Kelch like superfamily domain. Kelch family of phosphatases are present in *A. thaliana* and in other plants. Major expansion of protein phosphatase type with MPP and PP2Cc superfamily in *P. falciparum* was similar to that observed in *A. thaliana*. In *H. sapiens,* PTP superfamily is the largest group in contrast to other model organism studied here. These genome specific expansions probably reflect the evolutionary pressure for a flexible and complex intracellular signalling in these multicellular organisms, most likely acquired and developed during the course of evolution.

**Table 1:** ***P. falciparum*** **proteins with conserved phosphatase related superfamily domains (accession numbers as per** http://www.plasmoDB.org **, version 9.2 and superfamily according to NCBI CDD search)**

ID	Description	Length
**MPP ((Metallophosphatase)) superfamily**		
**PF3D7_0314400**	serine/threonine protein phosphatase, putative	308
**PF3D7_0802800**	serine/threonine protein phosphatase, putative	604
**PF3D7_0918000**	glideosome-associated protein 50,secreted acid phosphatase (GAP50)	396
**PF3D7_0925400**	protein phosphatase-beta	466
**PF3D7_0927700**	serine/threonine protein phosphatase, putative	312
**PF3D7_1018200**	serine/threonine protein phosphatase, putative	2166
**PF3D7_1206000**	protein phosphatase, putative	304
**PF3D7_1355500**	serine/threonine protein phosphatase (PP5)	658
**PF3D7_1403900**	phosphatase, putative	298
**PF3D7_1406700**	phosphatase, putative	194
**PF3D7_1414400**	serine/threonine protein phosphatase (PP1)	304
**PF3D7_1423300**	serine/threonine protein phosphatase (PP7)	959
**PF3D7_1464600**	phosphatase, putative	1442
**PF3D7_1466100**	protein serine/threonine phosphatase	889
**PF3D7_1469200**	protein phosphatase, putative	358
**PF3D7_0107800**	DNA repair exonuclease Mre11, putative	1233
**PF3D7_0912400**	conserved Plasmodium protein, unknown function	446
**PF3D7_1340600**	RNA lariat debranching enzyme, putative (DBR1)	575
**HP (Histidine phosphatases) superfamily**		
**PF3D7_1430300**	acid phosphatase, putative	2657
**PF3D7_0208400**	conserved Plasmodium protein, unknown function	2010
**PF3D7_0310300**	phosphoglycerate mutase, putative	1165
**PF3D7_0413500**	phosphoglucomutase-2 (PGM2)	295
**PF3D7_1120100**	phosphoglycerate mutase, putative (PGM1)	250
**HAD_like (Haloacid Dehalogenase) superfamily**		
**PF3D7_0515900**	protein phosphatase, putative	328
**PF3D7_0726900**	protein phosphatase, putative	519
**PF3D7_1012700**	protein phosphatase, putative	1438
**PF3D7_1226100**	hydrolase/phosphatase, putative	316
**PF3D7_1355700**	protein phosphatase, putative	1288
**PF3D7_1363200**	bifunctional polynucleotide phosphatase/kinase (PNKP)	462
**PF3D7_0715000**	4-nitrophenylphosphatase (PNPase)	322
**PF3D7_0817400**	conserved Plasmodium protein, unknown function	739
**PF3D7_1118400**	haloacid dehalogenase-like hydrolase, putative	306
**PF3D7_0303200**	HAD superfamily protein, putative	1162
**PAP2_like (2-phosphatidic acid phosphatases) superfamily**		
**PF3D7_0625000.1**	phosphatidic acid phosphatase (PAP)	439
**PF3D7_0625000.2**	phosphatidic acid phosphatase	461
**PF3D7_0805600**	apicoplast phosphatidic acid phosphatase, putative	308
**PP2Cc (Protein phosphatases 2c domain) superfamily**		
**PF3D7_1138500**	protein phosphatase 2c	924
**PF3D7_0410300**	protein phosphatase, putative	906
**PF3D7_0520100**	protein phosphatase, putative	706
**PF3D7_0810300**	protein phosphatase, putative	550
**PF3D7_0810500**	protein phosphatase, putative	303
**PF3D7_1009600**	protein phosphatase, putative	488
**PF3D7_1135100**	protein phosphatase, putative	689
**PF3D7_1249300**	protein phosphatase, putative	1027
**PF3D7_1309200**	protein phosphatase 2c-like protein, putative	827
**PF3D7_1455000**	protein phosphatase, putative	410
**PF3D7_1208900**	conserved Plasmodium protein, unknown function	1442
**EEP (Exonuclease-endonuclease Phosphatases) superfamily**		
**PF3D7_0319200**	endonuclease/exonuclease/phosphatase family protein, putative	906
**PF3D7_0705500**	inositol-phosphate phosphatase, putative	2814
**PF3D7_1111600**	endonuclease/exonuclease/phosphatase family protein, putative	744
**PF3D7_0107200**	carbon catabolite repressor protein 4, putative	337
**PF3D7_0305600**	AP endonuclease (DNA-[apurinic or apyrimidinic site] lyase), putative	617
**PF3D7_1238600**	sphingomyelin phosphodiesterase, putative	393
**PF3D7_1363500**	DNase I-like protein, putative	836
**PF3D7_1430600**	exodeoxyribonuclease III, putative	876
**Syja_N (SacL homology domain) superfamily**		
**PF3D7_0705500**	inositol-phosphate phosphatase, putative	2814
**PF3D7_0802500**	inositol phosphatase, putative	1419
**PF3D7_1354200**	inositol-polyphosphate 5-phosphatase, putative	803
**PTPLA (Protein tyrosine phosphatase-like protein) superfamily**		
**PF3D7_1331600**	protein tyrosine phosphatase, putative	228
**RHOD (Rhodanese Homology Domain) superfamily**		
**PF3D7_1305500**	protein phosphatase, putative	771
**PF3D7_1206400**	rhodanese like protein, putative	346
**CYTH-like_Pase (Triphosphate Tunnel Metaloenzyme Phosphatases) superfamily**		
**(Apicomplexan)**		
**PF3D7_0322100**	RNA triphosphatase (Prt1)	591
**PTH2_family (Peptidyl-tRNA hydrolase, type 2) superfamily**		
**PF3D7_0610500**	conserved protein, unknown function	123
**PTPc (Protein Tyrosine phosphatase) superfamily**		
**PF3D7_0309000**	dual specificity phosphatase (YVH1)	575
**PF3D7_1113100**	protein tyrosine phosphatase (PRL)	218
**PF3D7_1127000**	protein phosphatase, putative	287
**PF3D7_1455100**	protein phosphatase, putative	171
**Nucleoside Phosphatase superfamily**		
**PF3D7_1431800**	apyrase, putative	874
**PF3D7_1322000**	adenosine-diphosphatase, putative	565

We further analysed various annotated phosphatase family proteins on the basis of conserved phosphatase motifs, gene ontology and its evolutionary characteristics. Different phosphatases interact with various types of proteins and enzymes in various biological processes and metabolic pathways. In order to identify important protein interactions involving the parasite phosphatases, we performed STRING (version 9.05, http://STRING-db.org) [32] and PlasmoMAP analysis (The functional interactome of *Plasmodium*, (http://cbil.upenn.edu/plasmoMAP/downloads/uncompressed/highConfPairs.connections.txt). [33] For the STRING analysis, we used default parameters (Additional file 6). STRING analysis of the 67 phosphatase sequences helped identification of interacting partners for 60 phosphatase sequences (Additional file 7). PlasmoMAP analysis revealed interaction partners for 10 phosphatases overlapped STRING predictions (Additional file 8). Amongst these, the phosphatases corresponding to PF3D7_1414400, PF3D7_1466100 and PF3D7_0927700 have few common predicted interacting partners, increasing the chances of correct predictions by consensus. This interacting partner prediction analysis revealed that majority of the phosphatases are linked directly or indirectly through common targets.

In the following sections, we discuss the *in silico* data analysis of *P. falciparum* phosphatases present in each superfamily as well as their predicted interacting partners.

### MPP group – metallophosphatases

MPP (Metallophosphatases), also known as metallophosphoestrases, phosphodiesterases, binuclear metallophosphoestrases and dimetal-containing phosphoestrases, represent a diverse superfamily of enzymes with a conserved domain containing an active site consisting of two metal ions (usually Mn, Fe, or Zn) coordinated with octahedral geometry by a cage of histidine, asparagines and aspartate residues. This superfamily includes Phospho-protein phosphatases (PPPs), Mre11/SbcD-like exonucleases, Dbr1-like RNA lariat debranching enzymes, YfcE-like phosphodiesterases, purple acid phosphatases (PAPs), YbbF-like UDP-2,3-diacylglucosamine hydrolases, and acid sphingomyelinases (ASMases). Classically enzymes belonging to metallophosphatases have been clustered into 3 major groups: PP1, PP2A and PP2B, on the basis of substrate specificity and inhibitor sensitivity [24]. This classification has been extended with the identification of a range of sequences related to metallophosphatases, but distinct from the 3 classified groups due to early divergence from the other protein phosphatases in the evolutionary history of eukaryotes [27, 21]. Thus PPPs can be clustered into eight distinct subtypes of serine/threonine phosphatases: PP1, PP2A, PP2B, PP4, PP5, PP6, PP7, and plant specific BSU superfamily, characterized by the presence of Kelch motif [34]. PP2, PP4 and PP6 are closely related to each other as compared with other PPPs [35]. Furthermore, a family of bacterial like PPP sequences (Shelphs) is present in *P. falciparum*[36]. Three highly conserved motifs (GDXHG, GDXXDRG, GNH [E/D]) mediating metal ion coordination in the active centre are considered as signature of metallophosphatase family, however sequences without GDXHG or GDXXDRG motif have also been identified in plants, *Plasmodium* and few fungi species [36].

Previously 16 MPP catalytic domains were reported by Wilkes and Doerig [22]. We have included 2 additional sequences (PF3D7_0918000 and PF3D7_0912400) into the MPP superfamily. In this study*,* we identified 18 candidate proteins with catalytic domains conforming to metallophosphatases. Multiple sequence alignment of the 18 sequences along with the sequences of other species cited above was performed and a Neighbour Joining phylogenetic tree was generated. Annotations associated with these sequences indicate that the group mainly contains serine/theronine phosphatase sequences; and also sequences whose substrate is not a phosphoprotein. We further sub-grouped the metallophosphatases on the basis of different metallophosphatase domains and studied them in detail.

PP1: PF3D7_1414400 has been identified as PP1 type phosphatases. Analysis of PP1 inhibition has shown that the PP1 is responsible for a majority of protein phosphatase activity in *P. falciparum*. PlasmoMAP interacting partner prediction analysis shows that this phosphatase may play very important role in parasite growth; being linked with the chromosome condensation protein, minchromosome maintenance (MCM) complex subunit (MCM6), cdc2-like protein kinase, anaphase-promoting complex subunit, replication licensing factor, DNA replication licensing factor mcm5, DNA replication licensing factor mcm7 and others. PP1 is also predicted to interact with UDP-galactose transporter, Chromatin assembly protein (ASF1), and choline kinase. STRING database analysis shows that PP1 interacts with Pfn conserved proteins essential for the invasive blood stages of the parasite. Blisnick et al. have shown that PP1 plays an important role in the release of infectious merozoites [37]. Experimental evidences show that PfLRR1 [38] and PP1 regulators (PfI2, pfI3) inhibit PP1 catalytic activity [39, 40]. PP1 is able to functionally complement a glc7 (PP1) mutation in *S. cerevisiae*[41]. Inhibition activity using PP1 specific inhibitors I-1 and I-2 shows that PP1 is required for the erythrocyte reinvasion but might be dispensable for schizont maturation. PP1 depletion analysis using siRNA, showed inhibition of cell cycle progression in all erythrocytic stages [42]. It is also constitutively expressed [41, 42] as shown by mass spectroscopic studies [43].

PPKL: PF3D7_1466100 has been identified as a PPKL phosphatase and shows higher structural similarity to PP1 as compared to other metallophosphatases. It has 5 short inserts in its catalytic domain and a long unique N terminal domain. The N terminal domain of the protein is composed of tandem Kelch like repeats [34]. Another important feature of PPKL is Pro to Thr substitution in the conserved SAPNY motif and the absence of sequence similarity in the B12-B13 okadaic acid-binding loop. The PPKL gene structure is conserved in homologous sequences from the apicomplexan organisms reported by Wilkes et al. (one sequence per genome), as well as in the *A. thaliana,* with 4 PPKL sequences [22]. Kelch motifs form distinctive ‘propeller like’ tertiary structures proposed to mediate interactions with regulatory subunits [44]. BSU1, an *A. thaliana* PPKL is a nucleus localized protein studied in detail and appears to be involved in regulating signals from the brassinosteroid plant hormones [45]. The limited distribution of PPKLs (these proteins have been found only in plants and apicomplexans) is reminiscent with that of other gene families and is in line with the proposed photosynthetic ancestry of apicomplexa [46, 22]. Recent publications on PPKL have shown that it plays an important role in ookinetes morphology, motility and invasion [18, 47]. PF3D7_1018200 also contain MPP_BSU1 catalytic domain but lacks Kelch like motif in the N-terminal region. STRING database analysis indicates that this phosphatase may interact with the various vacuolar proteins. It is also predicted to interact with cysteine proteinase falcipain, which plays important role in haemoglobin degradation, an essential process for parasite survival.

PP2A, PP4, and PP6: PF3D7_0314400 was assigned to PP2A group of protein phosphatases [22, 48]. PP2A (Protein phosphatase 2A) is a critical regulator of many cellular activities, and comprises about 1% of total parasite cellular proteins. PP2A, together with protein phosphatase 1 (PP1), accounts for more than 90% of all serine/threonine phosphatase activities in most cells and tissues. Interacting partner prediction analysis indicates that it interacts with a wide range of proteins and enzymes like phosphotyrosyl phosphatase activator, Hsp70/Hsp90 organizing protein (HOP), tRNA m5C-methyltransferase, mRNA-decapping enzyme 2 (DCP2), and actin-like protein. Vandomme et al. reported that phosphotyrosyl phosphatase activator binds and activates PfPP2A [49]. STRING database analysis identifies interacting partners similar as of PPKL phosphatase; this similarity might be because of the high similarity of PPKL catalytic subunit with PP1 catalytic domain. The PP2A subunits, in addition to having a catalytic domain homologous to PP1 has a unique C-terminal tail, containing a motif that is conserved in the catalytic subunits of all PP2A-like phosphatases including PP4 and PP6, and plays an important role in PP2A regulation. The PP2A-like family of phosphatases share a similar heterotrimeric architecture, that includes: a 65 kDa scaffolding subunit A (subunit required for the activity of PP2Ac), a 36 kDa catalytic subunit (C), and one of the 18 regulatory subunits (B) [24]. Mass spectroscopic evidences show that PF3D7_0314400 is expressed in asexual stages as well as in gametocytes. PF3D7_0927700 and PF3D7_0925400 (identified as PfPPβ) [50] characterized to this group, show protein expression limited to schizont stage only (Mass spectroscopic evidence, PlasmoDB). PlasmoMAP prediction for PF3D7_0927700 shows that it may play an important role in replication phase of parasite cycle; as it is predicted to interact with almost every enzyme/proteins to which PP1 and PPKL phosphatases interact.

PP2B: PF3D7_0802800 is a PP2B/PP3/Calcineurin, a unique serine/threonine protein phosphatase with regulatory domains comprising of an auto-inhibitory region and sites of interaction with 2 Ca^2+^ binding proteins, calcineurin B and calmodulin, in addition to its catalytic domain [51]. These two confer Ca^2+^ dependent regulation of PP2B phosphatase activity. Cyclosporin A/Cyclophilin acts as inhibitor for PP2B enzymes [48]. Singh et al. showed that treatment of merozoites with calcineurin inhibitors limits the growth *P. falciparum* blood stage parasite [52]. PlasmoMAP interaction partner predictions showed that it interacts with cAMP, cGMP and calmodulin domain kinases as well as Ser/Thr protein kinases. PlasmoMAP also predicts its interaction with the Zinc finger protein, actin and protein phosphatase 2b regulatory subunit, regulating the phosphatase activity. PP2B is highly conserved from yeast to human’s genomes, however it is absent in the plants.

PP5: PF3D7_1355500, a C-terminal metallophosphatase domain identified in Serine/threonine protein phosphatase-5 (PP5) is a member of the PPP gene family of protein phosphatases, highly conserved among eukaryotes and widely expressed in mammalian tissues. PP5 has a C-terminal phosphatase domain and an extended N-terminal TPR (tetratricopeptide repeat) domain [53, 54]. TPR domains typically contain 34 amino acids with a consensus sequence [WLF]-X(2)-[LIM]-[GAS]-X(2)-[YLF]-X(8)-[ASE]-X(3)-[FYL]-X(2)-[ASL]-X(4)-[PKE]. It has an auto-inhibitory effect on the phosphatase activity of PF3D7_1355500, in other system this inhibition is overcome by binding unsaturated fatty acids [54]. Dobson et al. had shown that PP5 interacts with PfHSP90 [53]. PlasmoMAP predicts that PF3D7_1355500 protein interacts with a variety of enzymes and regulatory subunits of proteins ranging from eukaryotic translation initiation factor, lysine-tRNA ligase, erythrocyte membrane-associated antigen, heat shock protein 70, ADP/ATP transporter on adenylate translocase, RNA helicase and several others indicating it may be essential phosphatase required for parasite growth and development.

PP7/PPEF: PF3D7_1423300 protein phosphatase is regulated by Ca^2+^ via the C-terminal EF-hand motifs and a calmodulin binding site in the N-terminal domain [55]. It has been described previously as PfPPJ containing only phosphatase domain [56]. The phosphatase activity is okadaic acid-resistant, and catalysis requires Mn^2+^, and not any other cation like Mg^2+^ or Ca^2+^[22]. Subsequent analysis demonstrated the primary PF3D7_1423300 translation product to be much larger than the PP catalytic domain, with two EF-hand motifs that must be occupied by Ca^2+^ for the enzyme to become fully active [57]. The small size originally predicted for PfPPJ was due to a spurious stop codon in the original cDNA [56], but a fragment corresponding to this size is apparently produced by post-translational processing, detected by Western blotting. Despite the presence of calmodulin binding motif in N-terminal domain, recombinant PfPP7 expressed in *E. coli* is insensitive to calmodulin, but is stimulated by the presence of motifs in C-terminal domain; and its activity is regulated by auto-inhibition by Ca^2+^ binding [57]. STRING database analysis shows its co-expression with cAMP dependent protein kinase, rhoptry protein, merozoites surface protein and others showing it may play a vital role in parasitic growth and its invasion process.

Shelphs: PF3D7_1206000 and PF3D7_1469200 belongs to Shewanella-like phosphatases [36]. The characteristic feature of these phosphatases is the absence of SAPNY motif and the presence of another motif, (I/L/V) D(S/T) G, which may alter their substrate preference from Ser/Thr to broad or even Tyr specific [36]. PF3D7_1469200 is involved in microneme development in *Plasmodium* ookinetes development and parasite transmission [20]. PF3D7_1206000 (SHLP1) and PF3D7_1469200 (SHLP2) are characterized by N-terminal signal sequences. PF3D7_1469200 possess an additional apicoplast targeted motif [58–60]. PF3D7_1206000 has been likely to be involved in invasion of merozoites stage [19, 61, 62]. STRING database analysis reveals that PF3D7_1469200 interacts with myosin d, actin-depolymerising factor, calcium-dependent protein kinase 4 and Erythrocyte membrane protein. Mass spectroscopic evidence shows that the two proteins of this group are expressed in all the *P. falciparum* developmental stages.

ACP5: The *P. falciparum* proteins PF3D7_0918000 and PF3D7_1464600 belong to the MPP_ACP5 family. Acid phosphatase 5 (ACP5) removes the mannose 6-phosphate recognition marker from lysosomal proteins [63]. The exact site of dephosphorylation is not clear; however evidences suggest that dephosphorylation may take place in a pre-lysosomal compartment as well as in lysosome. PF3D7_0918000 is a glideosome associated protein 50, secreted acid phosphatase, with fully active ACP5 catalytic unit and annotated to be involved in protein anchoring [64]. Recent publications using GFP-fused PfGAP50 have confirmed the localization of PfGAP50 in the inner membrane complex of *P. falciparum*[65, 66]*.* Muller et al. had shown that secretion of GAP50 acid phosphatase provides a possible mechanism to acquire host nutrients by *P. falciparum*[65]. PF3D7_1464600 shows elaborate structural similarity to PF3D7_0918000. STRING database analysis shows its interaction with GAP50 acid phosphatase on the basis of binding evidences.

VPS29: VPS29 superfamily protein PF3D7_1406700 is characterized by a vacuolar sorting protein 29 domain, also known as vacuolar membrane protein Pep11, a subunit of the retromer complex which is responsible for the retrieval of mannose-6-phosphate receptors (MPRs) from the endosomes for retrograde transport back to the Golgi [67–69]. VPS29 consist of a phosphoestrase fold which acts as a protein interaction scaffold for retromer complex assembly as well as a phosphatase with specificity for the cytoplasmic tail of the MPR [70]. The retromer includes the following 5 subunits: Vps35, Vps26, Vps29, and a dimer of the sorting nexins Vps5 (Snx1), and Vps17 (Snx2). STRING database analysis shows that it interacts with the other vacuolar proteins like Vps35 and Vps26. The protein is also predicted to interact with vacuolar ATP synthase Subunit F, vacuolar ATP synthase Subunit D - beta 3, and proteasome subunit as well as 20S beta-4 proteasome subunit. It is shown in human that Vps29 binds with Vps35 and Vps26 to perform biological functions by participating in the formation of retromer complexes [71].

MPP_Dbr1: PF3D7_1340600 belongs to MPP_Dbr1 group of metallophosphatases. Dbr1 is an RNA lariat debranching enzyme that hydrolyzes 2′-5′ phosphodiester bonds at the branch points of excised intron lariats [72]. Severe growth defect occurs in a *Schizosaccharomyces pombe* mutant defective in intron lariat degradation [73], which is also true for human RNA lariat debranching enzyme [74]. Structure-function analysis of yeast RNA debranching enzyme (Dbr1) shows that it is a manganese-dependent phosphodiesterases enzyme [75]. To the best of our knowledge, there is no published work related to the *P. falciparum* phosphatase.

MPP_PhoD: PF3D7_0912400 consists of MPP_PhoD domain. PhoD phosphatase also known as alkaline phosphatase D/APaseD in *Bacillus subtilis*, is a secreted phosphodiesterases encoded by PhoD of the Pho regulon in *Bacillus subtilis*[76]. Its homologs are found in prokaryotes, eukaryotes and archaea. It contains a twin arginine (RR) motif and transported by the Tat (Twin-arginine translocation) translocation pathway machinery (TatAyCy) [77]. STRING database analysis shows its interaction with GTP cyclohydrolase I and 6-pyruvoyl tetrahydropterin synthase.

MPP_Mre: Mre11 (also known as SbcD in *E. coli*) is a subunit of the MRX (Mre11, Rad50 and Xrs2/Nbs1) protein complex. This complex includes: Mre11, Rad50, and Xrs2/Nbs1, and plays a vital role in several nuclear processes including DNA double-strand break repair, telomere length maintenance, cell cycle checkpoint control, and meiotic recombination in eukaryotes [78, 79]. During double-strand break repair, the MRX complex is required to hold the two ends of a broken chromosome together. In vitro studies show that Mre11 has 3′-5′ exonuclease activity on dsDNA templates and endonuclease activity on dsDNA and ssDNA templates [80–82]. In addition to the N-terminal phosphatase domain, the eukaryotic MRE11 members of this superfamily have a C-terminal DNA binding domain. MRE11-like proteins are found in prokaryotes, archaea as well as in eukaryotes. PF3D7_0107800 consists of 2 incomplete but catalytically active MPP_Mre11 domains.

MPP_CSTP1: PF3D7_1403900 consists of complete S-transactivated protein 1 (CSTP1) domain. CSTP1 domain is similar to *H. sapiens* protein with a metallophosphatase domain, which is transactivated by the complete S protein of hepatitis B virus. STRING database analysis shows its interaction with other *P. falciparum* phosphatases.

### PP2Cc group

Protein phosphatase 2C is a Mn^2+^ and Mg^2+^ dependent protein serine/theronine phosphatases. The active form of the protein appears as highly diverse monomeric polypeptides, which in many cases possess regulatory domain in C- or N-terminal extension. The protein architecture and deduced catalytic mechanism of PP2Cc group of phosphatases are similar to PP1, PP2A, and PP2B family of Ser/Thr phosphatases, despite low sequence similarity [83]. This group of phosphatases are greatly expanded in plants [84] and are involved in modulating stress responses [84, 85].

Eleven PP2Cc phosphatase sequences identified in *P. falciparum*, together with those from the other organism cited above were subjected to the multiple sequence alignment and Neighbour Joining phylogenetic tree construction. Annotations associated with these sequences indicate that this group mainly contains serine/theronine phosphatase class proteins. Interestingly several phosphatases of this group do not have human ortholog (8/11) (Additional file 1). PF3D7_1208900 is a new inclusion to this group with unknown function.

PF3D7_1138500 is the only PP2Cc characterized in the parasite [86]. It has been implicated in regulation of the translation elongation factor 1B, antagonising its in vitro phosphorylation by mammalian protein kinase C [87]. As compared to other PP2Cc phosphatases, PF3D7_1138500, PF3D7_0810300 and PF3D7_0520100 exhibit 2 distinct PP2Cc domains which may be interacting with each other forming two effective active sites as shown by Mamoun et al. for PF3D7_1138500 [86]. PF3D7_1138500 polypeptide has two distinct PP2Cc type domain, each capable of enzymatic activity [86]. They also concluded that dimerization of PfPP2c (PF3D7_1138500) is required for the optimal enzymatic activity [87].

### PTP group

Protein tyrosine phosphatases (PTP) group can be divided into three main families; tyrosine specific phosphatases, dual specific phosphatases, and low molecular weight phosphatases [88]. The dual specific phosphatases include Cdc25-like, Cdc14 and MAPK phosphatases [89–91]. The tyrosine and dual specific phosphatases are involved in signalling cell growth, differentiation and cell cycle progression control. Cdc25 is a major regulator of cyclin dependent kinases [89] and cdc14 regulates mitosis exit by dephosphorylating CDK targets [91]. The enzyme shares a common catalytic mechanism mediated by cysteine, arginine and aspartic acid residues. The protein family has a distinctive active site signature motif, (I/V) HCXXGXXRS, which harbours the catalytic cysteinyl residue and mutation of cysteine residue, abolishes the catalytic activities of PTPs [92].

The 4 catalytic domains conforming to PTPc were identified in *P. falciparum*, together with those from the other organisms cited above were subjected to the multiple sequence alignment and Neighbour joining Phylogenetic tree construction. Conserved Domain analysis characterizes PF3D7_0309000 (PfYVH1) and PF3D7_1455100 into dual specific phosphatases (DSPs) as reported previously [22, 93]. These DSPs acts on phospho (serine/threonine) as well as on phosphotyrosine; and plays important adaptive role in eukaryotic cells in response to extra or intra cellular stimuli. Two of the *P. falciparum* PTPs (PF3D7_0309000 and PF3D7_1113100) have been biochemically analysed [94, 95]. PF3D7_1113100 belongs to PRL (Protein of Regenerating Liver) group [96] and detected in the extracellular supernatant of infected RBCs [97]. This sequence possesses the CaaX C-terminal motif for farnesylation, a distinguishing feature of this group of phosphatases. It was demonstrated that this motif in PF3D7_1113100 is target of farnesyl transferase activity purified from parasite extracts. The purified protein from parasite extracts and recombinant PF3D7_1113100 displays phosphatase activity [95]. In *P. falciparum* merozoites, it co localizes with AMA-1, a membrane associated protein associated with invasion [95]. PlasmoMAP analysis for interacting partners of PF3D7_0309000 shows that it may be involved in regulation of translation process. It interacts with eukaryotic translation initiation factor-3 subunit 10, 60S Ribosomal protein L36, eukaryotic translation initiation factor eIF2A, Myb2 protein, pre-mRNA-splicing factor ISY1 homolog, proliferation-associated protein 2 g4 and several other proteins (PlasmoMAP). PF3D7_1127000 have incomplete PTP motif and it probably does not contain any catalytic active region for protein phosphatase activity. PF3D7_1455100 contains both active site and active residues to perform phosphatase activity, however no literature is available for this phosphatase.

### HP – histidine phosphatases

The characteristic feature of this group is that the conserved catalytic core of its domain contains a His residue, which is phosphorylated in the reaction along with two key Arg residues. An additional His residue is hydrogen bonded to the phospho group before, during and after transfer [98]. This set of proteins includes cofactor-dependent and cofactor-independent phosphoglycerate mutases (dPGM, and BPGM respectively), fructose-2,6-bisphosphatase (F26BP)ase, Sts-1, SixA, histidine acid phosphatases, and phytases. HP superfamily proteins play functional role in metabolism, signalling and regulation, for example F26BPase affects glycolysis and gluconeogenesis through controlling the concentration of F26BP.

CDD search of *P. falciparum* proteome yielded 5 HP superfamily hits, predicted to be involved in glycolysis pathway. Genetic characterization of PF3D7_0413500 (PfPGM2) homolog in rodent parasite *P. berghei* indicates that PF3D7_0413500 may be essential for blood stage asexual growth and zygote ookinetes development [99]. Their experiments show that PfPGM2 has a prominent phosphatase activity in contrast to weak mutase activity and it acts as an enzyme in phosphate metabolism or regulator of parasite life cycle. STRING database analysis shows the interaction of this phosphatase with the proteins/enzymes involved in glycolysis pathway like enolase, phosphoglycerate kinase, PF3D7_1120100 and others. PF3D7_1430300 consists of an additional domain ATP-grasp_4.

### HAD – haloacid dehalogenase like superfamily

Haloacid dehalogenase-like hydrolases (HAD) includes L-2-haloacid Dehalogenase, epoxide hydrolase, phosphoserine phosphatase, phosphomannomutase, phosphoglycolate phosphatase, and P-type ATPase [100][101]. These proteins catalyze nucleophilic substitution reactions at phosphorus or carbon centers, using a conserved Asp carboxylate in covalent catalysis. All members possess a conserved alpha/beta core domain, and most of the proteins also possess a small cap domain, with varying folds and functions. Members of this superfamily contain DXDX (T/V) motif, hence members of this group belong to the DDDD superfamily of phosphohydrolases [29].

CDD search of *P. falciparum* proteome revealed 10 HAD_like superfamily domain hits. Similar to the *P. falciparum* PP2Cc superfamily proteins, it mainly contains putative phosphatases (7/10), one with conserved *Plasmodium* sequence with unknown function, and 50% identified to this group do not show ortholog in humans (Additional file 1). HAD superfamily consists of proteins belonging to NIF (NLI Interacting Factor-like phosphatase), with active DxDx (T/V motif) and BRCT domain (BRCA-C-terminal domain conserved for phospho-binding [102]) of Fcp1 phosphatases.

PF3D7_0515900, PF3D7_0726900, PF3D7_1012700, and PF3D7_1355700 are previously reported *Plasmodium* proteins of this superfamily [22]. PF3D7_0515900 and PF3D7_1355700 may possess phosphatase activity due to the presence of proper DxDx (T/V) motif. PF3D7_1012700 has DxDx (T/V) motif, but it is not present in catalytic active domain region. It was also reported that PF3D7_1012700 lacks the DxDx (T/V) motif [22] suggesting it may be catalytically inactive. PF3D7_1012700 and PF3D7_1355700 possess the BRCT domain assigned to fcp1 phosphatases. PF3D7_0515900 lacks BRCT domain and hence related to SCP type phosphatases [103, 104]. STRING database analysis predicts that PF3D7_1355700 and PF3D7_1012700 are probably involved in the transcription process as they interact with enzymes or proteins involved in transcription mainly RNA polymerase. PF3D7_0726900 contains several disrupted partial DxDx (T/V) motifs, having only DxDx part of the active motif, suggesting it may be inactive and may not possess phosphatase activity. CDD results also show the absence of catalytically active site in PF3D7_0726900. Gene ontology analysis predicts its involvement in protein binding. Other than the four, all the other members of this superfamily are new inclusions to the HAD_like superfamily.

PF3D7_1363200, a bifunctional polynucleotide phosphatase/kinases (PNKP) enzyme consists of a proximal 5′-kinase module with an essential P-loop motif, GXGK(S/T) and a distal 3′-phosphatase module with an essential acyl-phosphatase motif, DX- DXT. Hence PNKP is able to perform phosphorylation as well as dephosphorylation. Siribal et al. characterized PNKP enzyme and detected phosphatase activity [105]. PF3D7_0817400 also contains two PNKP domains, however only the first containing the active domain region.

PF3D7_0715000 is a 4-nitrophenylphosphatase, which probably interacts with many cellular proteins involving Succinate dehydrogenase, cytidine triphosphate synthetase, asparagine synthetase, GTPases, RNA binding Protein, tRNA synthetase, ribosomal proteins and many more involved in transcription regulation and related cellular functions (STRING database analysis).

### EEP superfamily

Exonuclease/Endonuclease-Phosphatase possess diverse catalytic domain(s) of different proteins including the ExoIII family of apurinic/apyrimidinic (AP) endonucleases, inositol polyphosphate 5-phosphatases (INPP5), neutral sphingomyelinases (nSMases), deadenylases (such as the vertebrate circadian-clock regulated nocturnin), bacterial cytolethal distending toxin B (CdtB) and deoxyribonuclease 1 (DNase1). These diverse enzymes share a common catalytic mechanism of cleaving phosphodiester bonds containing substrates [29].

PlasmoDB and CDD analysis revealed 8 hits in this superfamily, all annotated with putative functions. Out of the eight in EEP superfamily parasite proteins, six do not have any human ortholog. PF3D7_0705500 and PF3D7_1111600 have additional inositol polyphosphate 5-phosphatase (INPP5c) domains. INPP5c phosphatases are Mg^2+^ dependent and hydrolyze the 5-phosphate from the inositol ring of various 5-position phosphorylated phosphoinositides (PIs) as well as inositol phosphatases. PF3D7_0705500, in addition to EEP domain, consists of Syja_N superfamily domain too. PF3D7_0319200 BLAST results showed that sequences similar to it are 2′5 - Phosphodiesterases. PFD7_1238600 is annotated as sphingomyelin phosphodiesterases in PlasmoDB database. STRING database analysis reveals its possible interactions with cAMP-specific 3′,5′-cyclic phosphodiesterases 4D, merozoite surface protein 9, actins (PFL2215w), rhoptry associated protein 2 (RAP2), GAP50 and diacylglycerol kinases based on co-expression profile showing that PF3D7_1238600 may play important role in parasite development and its growth. Other members of this group PF3D7_0305600, PF3D7_0107200, PF3D7_1363500 and PF3D7_1430600 have been assigned to this group on the basis of presence of conserved EEP domain.

### PAP2_like group

PAP2_like proteins are a superfamily of histidine phosphatases and vanadium haloperoxidases. The super-family includes type 2 phosphatidic acid phosphatases, lipid phosphate phosphatases, glucose-6-phosphatases, bacterial acid phosphatases, and phosphatidylglycerophosphatase. Members of this group of proteins harbouring conserved phosphatase motif KXXXXXXRP-(X12-54)-PSGH-(X31-54)-SRXXXXXHXXXD [106], are predicted to be transmembrane proteins.

Two PAP2_like phosphatase sequences (PF3D7_0625000.1/PF3D7_0625000.2 and PF3D7_0805600) are predicted to be phosphatidic acid phosphatase having transmembrane domain. PF3D7_0625000.1 and PF3D7_0625000.2 have similar amino acid sequence in the conserved domain region with 100% (identity!), but PF3D7_0625000.2 consists of an additional 22 amino acid in its non-conserved domain region. It could be due to an incorrect exon/intron prediction or alternate splicing. Active conserved PAP2 phosphatase motif is present only in PF3D7_0805600 while PF3D7_0625000.1/PF3D7_0625000.2 contains incomplete phosphatase motif.

### Nucleoside phosphatase group

This group includes NBD_sugar-kinase_HSP_actin and GDA1_CD39 domain containing phosphatases. NBD_sugar-kinase_HSP_actin group includes actin family, HSP70 family of molecular chaperons and nucleotide exchange factors; hexokinase family and exopolyphosphatase/nucleoside triphosphatase. The nucleotide binding site residues are conserved and the nucleotide sites in a deep cleft are formed between the two lobes of the nucleotide binding domain. Substrate binding to group members is associated with the closure of the catalytic cleft.

CDD classifies two phosphatases PF3D7_1431800 (apyrase) and PF3D7_1322000 (adenosine-diphosphatase) to this superfamily. GDA1_CD39 phosphatase domain is present in both phosphatases, whereas NBD_sugar-kinase_HSP_actin is present only in PF3D7_1431800. STRING results show that apyrase interacts with proteins/enzymes involved in DNA replication, DNA repair as well as in pre-mRNA splicing. Adenosine diphosphatase STRING database analysis shows that it interacts with chromosome condensation protein, peptidase, and cysteine-rich surface protein. Protein expression profile also suggests that it is expressed throughout all the stages of malaria parasite, making it a suitable target for antimalarial drug target.

### RHOD group

Rhodanese homology domain is an alpha beta fold domain found duplicated in the rhodanese protein. The cysteine containing enzymatically active form (CX5R) is also present in Cdc25 class of protein phosphatases. Cdc25 plays an important role in cell cycle [107]. CDD search reported 2 phosphatase sequences; PF3D7_1206400 (two domains) and PF3D7_1305500 (one domain) containing RHOD (an alpha beta fold domain) domain, none of them with CX5R motif. CDD predicts the presence of catalytic active residue in RHOD domain present in both phosphatases. STRING database analysis shows that these phosphatases may be involved in interaction with 6-pyruvoyl tetrahydropterin synthase and NLI interacting factor-like phosphatase. Recently PF3D7_1305500 has been shown as a MAPK phosphatase playing an essential role in the life cycle and development of *P. falciparum*[108, 109].

### PTPLA group

PTPLA group is different from the protein tyrosine phosphatases due to the presence of a proline instead of arginine at the catalytically active site, therefore it is referred as protein tyrosine like protein and the mutation renders its catalytic activity [110]. It is predicted to be involved in the development, differentiation, and maintenance of number of tissue types [111–113]. The presence of PTPLs in distant eukaryotic lineages strongly suggest important role they play in eukaryotes [59]. PTPL represent one of the several examples of phosphatases that have lost their enzymatic activity and are involved in other functions.

PF3D7_1331600 contains a PTPLA domain. Similar to other protozoan PTPLs, PF3D7_1331600 have either ER (Endoplasmic reticulum) retention signal (KKXX) or a similar C-terminal sequence enriched in basic amino acids [59]. It consist of highly positively charged N-terminus; however this predicted structure could not be verified using ESTs and not found conserved in other *Plasmodium* species, suggesting it may be a case of incorrect exon prediction (it has been reported that almost ¼ predicted open reading frame in *P. falciparum* have incorrect exon structure [114, 59]. STRING database analysis suggests that PTPLA may interact with steroid dehydrogenase kik-i, which in turn is involved in many metabolic pathways.

### Syja_N group

This Sacl homology domain is found at the amino terminal of inositol-5-phopshatase synaptojanin. These phosphatases are involved in recycling of synaptic vesicles secreted, nerve impulse transmission and predicted to be involved in the coordination of activities of secretary pathway and actin cytoskeleton. We identified three Syja_N domain containing sequences (PF3D7_0705500, PF3D7_0802500, and PF3D7_1354200) belonging to inositol phosphatases, were identified in *P. falciparum* proteome. This family represents a protein domain, which shows homology to the yeast protein SacI. STRING database analysis shows that PF3D7_1354200 interacts with 2-oxoglutarate/malate translocator protein, P-type ATPase, acyl CoA binding protein and beta-hydroxyacyl-acp dehydratase precursor. It is also predicted to interact with the mitochondrial inner membrane translocase. STRING did not predict any interaction partner for PF3D7_0802500.

### CYTH_like phosphatases group

CYTH group also known as triphosphate tunnel Metaloenzyme hydrolyzes the triphosphate containing substrates and requires metal cation cofactors. PF3D7_0322100, a RNA triphosphatase belongs to CYTH-like_Pase group and is predicted to be involved in polynucleotide 5′-phosphatase activity, protein binding and mRNA capping process [43].

### PTH2 group

Peptidyl-tRNA hydrolase, type 2 causes release of tRNA from the premature translation product peptidyl tRNA. PTH group is present in bacteria and eukaryotes, whereas the same function is performed by PTH2 in archaea and eukaryotes. PTH and PTH2 are structurally different enzymes. CDD search revealed one PTH2_family domain hit (PF3D7_0610500). The protein is predicted to be involved in protein tyrosine phosphatase and protein dephosphorylation activity [43]. STRING predicts it to be involved in the activity of u6 snRNA-associated sm like protein and DNA directed RNA polymerases.

### Additional functional characterizations

Since good quality Protein Data Bank (PDB) experimental structures and homology-based models could be used for *in-silico* screening experiments for finding novel drug leads, we set out to explore available plasmodium phosphatase structures and possibility of generating reliable molecular models of the proteins for which experimental structures are unavailable. A PlasmoDB search identified 12 structures of phosphatases, out of which 3 are experimental structures (PF3D7_0413500, PF3D7_0918000 and PF3D7_1120100). NCBI PDB sequence BLAST helped in identification of 17 phosphatases with more than 40% similarity to suitable templates (threshold value for homology modelling). Homology based modelling could be performed only on 11 malarial phosphatases, with conserved domain region present in the models (Data not shown) while the remaining 7 do not contain conserved domain region in the homology based models, especially in the cases where query sequence similarity with the template is poor.

### Stage specific and sub-cellular distribution of *P. falciparum*Phosphatases

**Figure 4:**
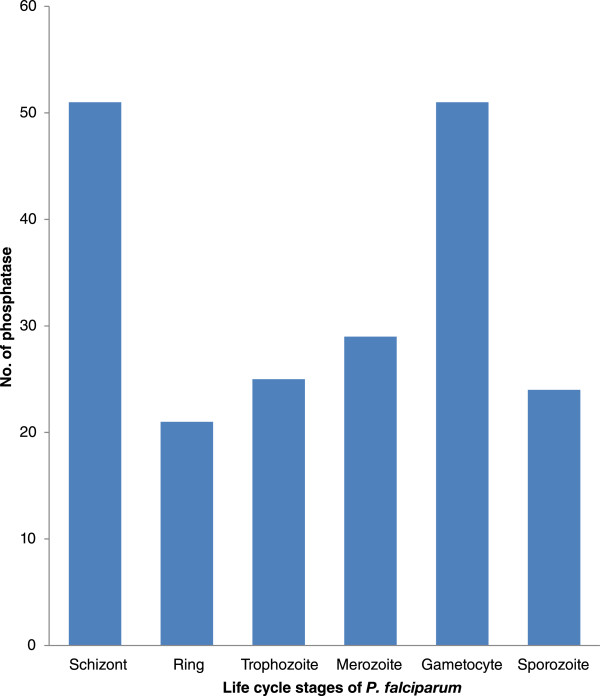
**Phosphatase proteins distribution according to their protein expression (using mass spectroscopic evidences).** One gene could be present in many stages.

We analysed the protein expression profile (Mass spectroscopic evidences, using PlasmoDB version 9.2) of the identified *Plasmodium* phosphatases to understand their distribution in different stages of *P. falciparum* life cycle (Figure 4). Protein expression profile shows that schizont and gametocytes stages share 44 common phosphatases. Fourteen phosphatases are common in schizont, ring and trophozoite stages. Four phosphatases are expressed only in gametocytes, whereas 3 in schizont stage alone.

Sub-cellular localization prediction yielded 6 phosphatase family proteins, namely- PF3D7_0305600, PF3D7_0319200, PF3D7_0918000, PF3D7_1431800, PF3D7_1455100 and PF3D7_1469200, targeted to apicoplast [43, 115]. Mitoprot [116] (http://ihg.gsf.de/ihg/mitoprot.html) and PlasMit algorithm [117] (http://gecco.org.chemie.uni-frankfurt.de/plasmit/) were used to predict mitochondrial targeted genes. Consensus predictions of Mitoprot (>0.60 probability) and PlasMit algorithm (positive score) for mitochondrial-targeted sequences include PF3D7_0413500, PF3D7_0515900, PF3D7_0726900, PF3D7_0810300, PF3D7_0817400, PF3D7_0925400 and PF3D7_1135100.

### Gene ontology and ortholog group analysis

**Figure 5:**
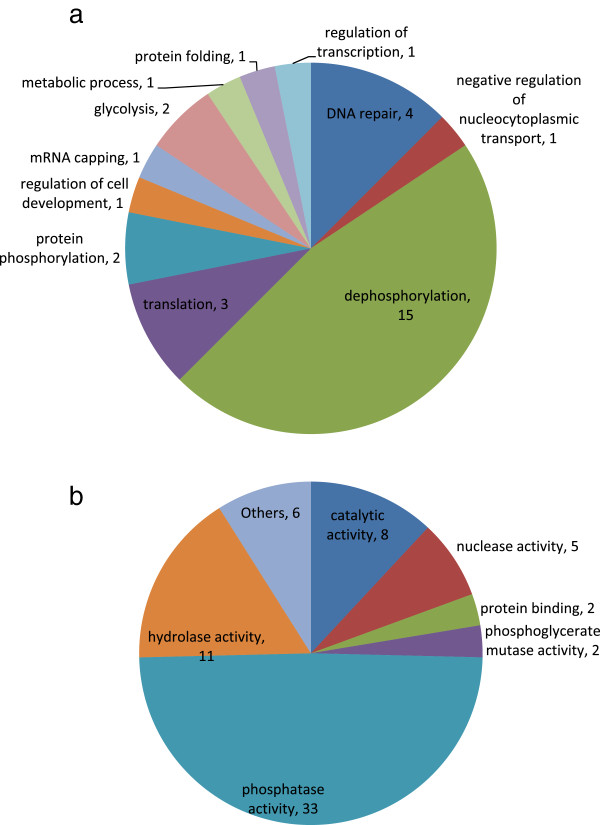
**Gene Ontology annotations for** ***P. falciparum*** **phosphatases– a. Biological processes and b. Molecular functions.**

Gene ontology analysis revealed that 33 of the *P. falciparum* phosphatases are probably involved in phosphatase like activity; out of which, 10 are indicated to have Serine/theronine phosphatase activity. Eleven phosphatases perform hydrolase function. The study further showed that 15 annotated protein phosphatases are directly involved in dephosphorylation process. The two phosphatases; PF3D7_0309000 and PF3D7_1309200, perform both protein phosphorylation as well as dephosphorylation process. The present study thus reveals that *Plasmodium* phosphatases are involved in a wide range of processes and functions. Figure 5a and b show the diagrammatic representation of *P. falciparum* phosphatases involved in various molecular functions and biological processes based on gene ontology function.

OrthoMCL analysis of *P. falciparum* phosphatases shows presence of 33 phosphatase sequences for which no human orthologs are present and out of this, 6 are *Plasmodium* specific. These 33 phosphatases are grouped into the following phosphatase super-families; 8 in PP2Cc, 7 in MPP, 6 in EEP (1 shared with Syja_N superfamily), 5 in HAD, 2 in HP and PAP2, 1 in PTPc, RHOD and CYTH superfamily.

### Phylogenetic analysis

**Figure 6:**
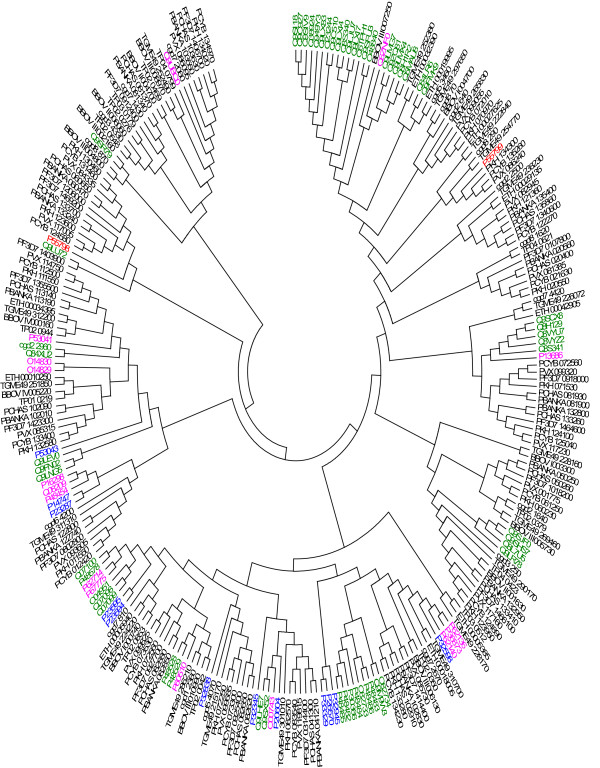
**Phylogenetic analysis for MPP domain superfamily.** *H. sapiens* (pink), *E.coli* (red), *S. cerevisiae* (blue), *A. thaliana* (green), *P. falciparum* (PF3D7), *P. berghei* (PBANKA), *P. vivax* (PVX), *P. chabaudi chabaudi* (PCHAS), *P. cynomolgi* (PCYB), *P. knowlesi* (PKH), *T. gondii* (TGME49), and *E. tenella* (ETH), *B. bovis* (BBOV)*, T. parva* (TP)*, C. parvam* (cgd) is used to perform evolutionary analysis. MEGA software is used to perform Phylogenetic analysis. Sequence alignment is performed using Clustal X and Muscle. NJ method is used to generate the phylogenetic tree.

We performed phylogenetic analysis for the major groups present in *P. falciparum* and the selected model organisms. ClustalX 2 [118] and Muscle [119] were used to perform protein sequence multiple sequence alignments and Mega 5.2 [120] was used to perform phylogenetic analysis. Phylogenetic analysis performed on 13 *P. falciparum* phosphatase superfamily sequences with the model organism cited above validates the OrthoMCL classification. Figure 6 shows phylogenetic tree for MPP domain superfamily proteins studied here. Phylogenetic tree for other super-families are presented in Additional files 9, 10, 11, 12, 13, 14, 15, 16, 17, 18, 19 and 20. Phylogenetic trees in general show clustering of *P. falciparum* phosphatases with the apicomplexans as well as with the non-apicomplexans orthologs, however the phylogenetic trees for each of the known phosphatase super families reveal a considerable phylogenetic closeness amongst apicomplexan organisms and considerable phylogenetic distance with the other eukaryotic model organisms. For few cases our analysis does not show all the orthologs classified by OrthoMCL database due to the fact that we retrieved only the referenced and reviewed non-apicomplexan phosphatase sequences from UNIPROT. Phylogenetic analysis of MPP superfamily shows clustering of phosphatases into distinct groups, according to different metallophosphatase subgroups, namely PP1, PPKL, PP2B, PP5, Shelphs and others. This indicates the conservation of metallophosphatase superfamily during the course of evolution across various lineages. PP2B absent in plants hence, *A. thaliana* sequence is missing in the PP2B phylogenetic tree. PTPc superfamily phylogenetic shows two dual specific phosphatases, PF3D7_0309000 and PF3D7_1455100 clustered together. Interestingly, PF3D7_0309000 and PF3D7_1455100 have human orthologs Q9UNI6 and Q16829, respectively. Similarly, the other two PTPc phosphatases cluster together. Phylogenetic tree of HP superfamily reveals that 2 *P. falciparum* phosphatases have orthologs in all the apicomplexans. Phosphatases belonging to *H. sapiens* and *S. cerevisiae* form separate clusters in HP superfamily. Few of the phosphatases perform different function other than Histidine acid phosphatase or phosphoglycerate mutase and might have lost phosphatase function during course of evolution, however due to sequence similarity and the presence of HP conserved domain, we classified them into this superfamily [121, 122]. Phosphatases belonging to EEP (with few exceptions) domain also get clustered in phylogenetic tree according to the gene ontology function predicted or annotated for them. EEP phosphatases are clustered according to their functional group of inositol phosphatase, CCR4, or nuclease family. In PP2Cc and HAD superfamilies, the phylogenetic analysis shows that in general apicomplexan organism are clustered together on the basis of their orthologies. The *H. sapiens, S. cerevisiae, T. gondii, A. thaliana* and *E. coli* (absent in PP2Cc) phosphatases form separate cluster in both the super-families. *T. gondii* have higher number of phosphatases as compared to other apicomplexans studied here, forms separate cluster in PP2Cc Phylogenetic tree. Phylogenetic tree shows presence of one Syja_N *P. falciparum* phosphatase sequences, PF3D7_0705500, in all the apicomplexans studied here. Phylogenetic tree shows that two human and three *S. cerevisiae* phosphatases may have relatedness with PF3D7_0705500, but there is no OrthoMCL evidence to support this. This group is absent in *E. coli* and although OrthoMCL predicts *A. thaliana* ortholog, we could not get any phosphatase related to *A. thaliana* because strict sequence collection procedure adopted by us. OrthoMCL analysis of PAP2 superfamily phosphatases show absence of human orthologs, phylogenetic tree reveals some similarity with human phosphatases although sequence similarity is low to conclude such relatedness of parasite phosphatase with the host. Phosphatases belonging to RHOD superfamily can be grouped into two clusters; cell cycle control phosphatase Cdc25 and MKP1 (Mitogen Activated Kinase Phosphatase 1) and its ortholog is present in all the model organisms except *E. coli*. For nucleoside phosphatase subfamily, all the *Plasmodium* species contain one phosphatase each except *P. falciparum*, which has two phosphatases belonging to this group. PF3D7_1431800 clusters with *T. gondii* phosphatase (TGME49_307800), which is greatly expanded in *T. gondii* as compared to other apicomplexans studied here. CYTH superfamily phosphatase is present in most of the apicomplexans studied and *S. cerevisiae*. PTPLA phosphatases are present in all model organism studied here, except *E.coli, T. parva* and *S. cerevisiae*. There is one PTPLA phosphatase in all the apicomplexans and *A. thaliana,* however four in *H. sapiens*. Amongst all the model organisms studied here, *P. falciparum*, *P. berghei*, *P. chabaudi*, and *P. Knowlesi* and *H. sapiens* have single PTH2 phosphatase protein, whereas *T. parva* and *C. parvam* have 2 each. The phylogenetic tree analysis also reveals that human phosphatases shows great expansion in PTP group whereas *A. thaliana* shows clustered expansion in MPP and PP2Cc superfamily.

## Conclusion

In this study, we have closely re-examined and classified various *Plasmodium* phosphatase family proteins based on a broader selection criteria such as diverse substrates, apart from conserved phosphatase protein family motifs. Phosphatases can have different substrates- ranging from proteins to non-proteins, for example substrates for the parasite phosphatases: PF3D7_1414400, PF3D7_1340600, PF3D7_0413500 are protein, RNA and carbohydrates, respectively. OrthoMCL analysis showed that 33 *P. falciparum* phosphatase sequences have orthologs in human and 6 phosphatases are restricted to *Plasmodium* species. *P. falciparum* phosphatase proteome and its interacting partner analysis thus provides useful insights about the stage specificity and cellular expression of *Plasmodium* phosphatases. Structures of three phosphatases are known and several others can be approximated, which will enhance our understanding of the molecular functions performed by parasite phosphatases. Identification of six Plasmodium specific phosphatases may be useful in identifying novel drug targets and also help in understanding their role(s) in different parasite pathways.

Although the analysis in this review is based on current annotations of the genomes included in the study, it is likely that most of the annotations will be final but there is still a chance that some annotations may change in future. Prediction of phosphatase function for the candidate *P. falciparum* phosphatases is based on the CDD/PFAM/BLAST/Homolog results, which solely rely on the conserved sequence patterns present throughout the protein families in the evolutionary trees of the species. Interacting partner prediction analysis of candidate phosphatases reveals its involvement in essential biological pathways necessary for the parasite survival. It might be possible that in *P. falciparum,* some of these identified putative proteins are inactive as phosphatases, but are still expressed (Mass spectroscopic evidence) in the parasite and may be involved in different biological processes performing essential functions required for the parasite survival. Although, we took all the precautions for the accuracy and completeness of initial identification, by using multiple strategies, final annotations of molecular functions and pathways involved requires experimental evidences.

## Methods

### Selection of protein phosphatase catalytic domains

We performed query text search with ‘Phosphatase’ in PlasmoDB (http://www.plasmodb.org version 9.2) [43] and EuPathDB (http://eupathdb.org/eupathdb/) [123] to get phosphatase sequences for apicocomplexan model organisms. We retrieved *E. coli, A. thaliana, S. cerevisiae* and *H. sapiens* phosphatase protein sequences from uniprot (http://www.uniprot.org/) dated November, 2012. Only referenced and reviewed sequences were included to perform further analysis. We performed NCBI Conserved domain database (CDD) [29] search for the selected predicted phosphatase sequences. Sequence having phosphatase domain were selected. Text search in PlasmoDB, which resulted in 89 phosphatases after performing CDD analysis and gene ontology analysis for functions, resulted in 67 phosphatases. Further analysis of *P. falciparum* phosphatases resulted in its assignment to 13 phosphatase super-families, on the basis of CDD result. We also used PFAM to confirm predicted phosphatases in amongst selected *P. falciparum* sequences. To cross – check our results, we also repeated the domain search for complete *P. falciparum* proteome (5777 *P. falciparum* proteins) to find phosphatase domain using both CDD and PFAM databases, which finally resulted in same number of predicted phosphatases. Additionally, we performed pBLAST against NCBI non-redundant database, for each predicted phosphatase as query to further validate protein sequence assignment as phosphatases. We used protein expression profile (Mass spectroscopic evidences) of these 67 phosphatase proteins to distribute into different stages of *P. falciparum* life cycle.

### 3D structures

We used PlasmoDB version 9.2 to search the predicted or experimental structures for *P. falciparum* phosphatases. We also performed BLAST search to query parasite protein sequences to a sequence database of PDB structures, in order to identify available parasite PDB structures. This step also helped exploring possible template protein structures for generating phosphatases homology models, experimental structures for which are unavailable.

### Sub-cellular targeted phosphatases

We used Mitoprot and PlasMit algorithm for putative mitochondrial targeting sequence prediction. Mitoprot uses two algorithms to predict mitochondrial targeted sequences (N-terminal protein region that can support a mitochondrial targeting sequence and the cleavage site). PlasMit uses first 20 amino acids to predict mitochondrial targets. PlasmoAP algorithm is used to predict the putative apicoplast targeted sequences [115]. It uses amino acid frequency and distribution to identify putative apicoplast targeted sequences.

### Gene ontology and ortholog group analysis

We used PlasmoDB version 9.2 assigned gene ontologies and orthologs in order to study the molecular and biological function performed by various phosphatase in P. falciparum and orthology with respect to other apicomplexans, host and model organisms. Only annotated gene ontology assigned to protein was selected. PlasmoDB (ver9.2) and OrthoMCL 5 database (http://www.OrthoMCL.org) was used to identify phosphatase orthologs of P. falciparum in the model organisms included in our study.

### Interacting partner analysis

We used STRING 9.05 database and PlasmoMAP (version 1.0) to predict the interacting partners of the phosphatases. STRING uses neighbourhood, gene fusion, co expression, co-occurrence, homology, text mining, database information and experiments to list the interacting partners using scoring method. We preformed sequence search in STRING database, as it does not identify new PlasmoDB IDs. PlasmoMAP network is generated using various computational tools and functional genomic data within a Bayesian framework to predict interacting network.

### Phylogenetic analysis

#### Multiple sequence alignment

*P. falciparum* sequences and the sequences of other apicomplexan model organisms were retrieved from PlasmoDB and EuPathDB databases, respectively. Protein Sequences for *E. coli, A. thaliana, S. cerevisiae* and *H. sapiens* were retrieved from Uniprot database (Dated November, 2012). Pairwise and multiple sequence alignment was performed for different phosphatases group using Clustal *X*2 and then Muscle software to get better alignment. Default parameters (Additional file 6) were used in both the cases to perform multiple sequence alignment.

### Phylogenetic tree generation

MEGA 5.2 software was used to generate phylogenetic tree. We used Neighbour Joining method to generate the phylogenetic tree. Jones Taylor Thornton (JTT) model was used for amino acid substitution. Gaps and missing data’s treatment was partial deletion with 95% site coverage cut-off. 1000 bootstrap replicates were performed to test the phylogeny tree (Other parameters in Additional file 6).

## Electronic supplementary material

Additional file 1:**Summary of** ***P. falciparum*** **phosphatases with their new and previous PlasmoDB ID, Length, maximum PDB similarity (ID and %), stage related protein mass spectroscopic evidences (PlasmoDB version 9.2), mitochondrial targeted (MitoProt) and apicoplast targeted, previously reported (Wilkes et al., BMC Genomics,**[22]**) and presence/absence of human ortholog.**(XLSX 27 KB)

Additional file 2:**A-Percentage of phosphatases distributed in different domain superfamily model organisms, B-Breakup representation of different phosphatase family found in model organism.**(DOCX 16 KB)

Additional file 3:**Schematic of all** ***P. falciparum*** **phosphatase super-families.**(PDF 191 KB)

Additional file 4:**Conserved domain alignment of** ***Plasmodium*** **specific phosphatases.**(PDF 156 KB)

Additional file 5:**Conserved domain alignment of all the** ***P. falciparum*** **phosphatases.**(PDF 544 KB)

Additional file 6:**Parameters used in various software and tools.**(DOCX 15 KB)

Additional file 7:**STRING prediction of interacting partners for** ***P. falciparum*** **phosphatases.**(DOCX 570 KB)

Additional file 8:**PlasmoMAP prediction of protein interacting partners for** ***P. falciparum*** **phosphatases.**(XLSX 14 KB)

Additional file 9:**Phylogenetic analysis for PTPc domain superfamily.** *H. sapiens* (green), *S. cerevisiae* (blue), *A. thaliana* (red) and *P. falciparum* (ID PF3D7), *P. berghei* (PBANKA), *P. vivax* (PVX), *P. chabaudi chabaudi* (PCHAS), *P. cynomolgi* (PCYB), *P. knowlesi* (PKH), *T. gondii* (TGME49), and *E. tenella* (ETH), *B. bovis* (BBOV)*, T. parva* (TP)*, C. parvam* (cgd) is used to perform evolutionary analysis. MEGA software is used to perform Phylogenetic analysis. Sequence alignment is performed using Clustal X and Muscle. NJ method is used to generate the phylogenetic tree. (PDF 98 KB)

Additional file 10:**Phylogenetic analysis for HP domain superfamily.** *H. sapiens* (green), *E.coli* (pink), *S. cerevisiae* (blue), *A. thaliana* (red), *P. falciparum* (PF3D7), *P. berghei* (PBANKA), *P. vivax* (PVX), *P. chabaudi chabaudi* (PCHAS), *P. cynomolgi* (PCYB), *P. knowlesi* (PKH), *T. gondii* (TGME49), and *E. tenella* (ETH), *B. bovis* (BBOV)*, T. parva* (TP)*, C. parvam* (cgd) is used to perform evolutionary analysis. MEGA software is used to perform Phylogenetic analysis. Sequence alignment is performed using Clustal X and Muscle. NJ method is used to generate the phylogenetic tree. (PDF 68 KB)

Additional file 11:**Phylogenetic analysis for HAD domain superfamily.** *H. sapiens* (green), *E.coli* (pink), *S. cerevisiae* (blue), *A. thaliana* (red), *P. falciparum* (PF3D7), *P. berghei* (PBANKA), P. vivax (PVX), *P. chabaudi chabaudi* (PCHAS), *P. cynomolgi* (PCYB), *P. knowlesi* (PKH), *T. gondii* (TGME49), and *E. tenella* (ETH), *B. bovis* (BBOV)*, T. parva* (TP)*, C. parvam* (cgd) is used to perform evolutionary analysis. MEGA software is used to perform Phylogenetic analysis. Sequence alignment is performed using Clustal X and Muscle. NJ method is used to generate the phylogenetic tree. (PDF 86 KB)

Additional file 12:**Phylogenetic analysis for Nucleoside Phosphatase domain superfamily.** *E.coli* (red), *P. falciparum* (PF3D7), *P. berghei* (PBANKA), *P. vivax* (PVX), *P. chabaudi chabaudi* (PCHAS), *P. cynomolgi* (PCYB), *P. knowlesi* (PKH), *T. gondii* (TGME49), and *E. tenella* (ETH), *B. bovis* (BBOV)*, T. parva* (TP)*, C. parvam* (cgd) is used to perform evolutionary analysis. MEGA software is used to perform Phylogenetic analysis. Sequence alignment is performed using Clustal X and Muscle. NJ method is used to generate the phylogenetic tree. (PDF 38 KB)

Additional file 13:**Phylogenetic analysis for EEP domain superfamily.** *H. sapiens* (green), *E.coli* (pink), *S. cerevisiae* (red), *A. thaliana* (blue), *P. falciparum* (PF3D7), *P. berghei* (PBANKA), *P. vivax* (PVX), *P. chabaudi chabaudi* (PCHAS), *P. cynomolgi* (PCYB), *P. knowlesi* (PKH), *T. gondii* (TGME49), and *E. tenella* (ETH), *B. bovis* (BBOV)*, T. parva* (TP)*, C. parvam* (cgd) is used to perform evolutionary analysis. MEGA software is used to perform Phylogenetic analysis. Sequence alignment is performed using Clustal X and Muscle. NJ method is used to generate the phylogenetic tree. (PDF 90 KB)

Additional file 14:**Phylogenetic analysis for CYTH_like_Pases domain superfamily.** *S. cerevisiae* (pink), *P. falciparum* (PF3D7), *P. berghei* (PBANKA), *P. chabaudi chabaudi* (PCHAS), *P. cynomolgi* (PCYB), *P. knowlesi* (PKH) and *T. gondii* (TGME49), *B. bovis* (BBOV)*, T. parva* (TP)*, C. parvam* (cgd) is used to perform evolutionary analysis. MEGA software is used to perform Phylogenetic analysis. Sequence alignment is performed using Clustal X and Muscle. NJ method is used to generate the phylogenetic tree. (PDF 34 KB)

Additional file 15:**Phylogenetic analysis for PP2Cc domain superfamily.** *H. sapiens* (red), *S. cerevisiae* (blue), *A. thaliana* (green), *P. falciparum* (PF3D7), *P. berghei* (PBANKA), *P. vivax* (PVX), *P. chabaudi chabaudi* (PCHAS), *P. cynomolgi* (PCYB), *P. knowlesi* (PKH), *T. gondii* (TGME49), and *E. tenella* (ETH), *B. bovis* (BBOV)*, T. parva* (TP)*, C. parvam* (cgd) is used to perform evolutionary analysis. MEGA software is used to perform Phylogenetic analysis. Sequence alignment is performed using Clustal X and Muscle. NJ method is used to generate the phylogenetic tree. (PDF 104 KB)

Additional file 16:**Phylogenetic analysis for RHOD domain superfamily.** *H. sapiens* (green), *S. cerevisiae* (pink), *A. thaliana* (blue), *P. falciparum* (PF3D7), *P. berghei* (PBANKA), *P. vivax* (PVX), *P. cynomolgi* (PCYB), *P. knowlesi* (PKH), *T. gondii* (TGME49), and *E. tenella* (ETH), *B. bovis* (BBOV)*, T. parva* (TP)*, C. parvam* (cgd) is used to perform evolutionary analysis. MEGA software is used to perform Phylogenetic analysis. Sequence alignment is performed using Clustal X and Muscle. NJ method is used to generate the phylogenetic tree. (PDF 49 KB)

Additional file 17:**Phylogenetic analysis for PTPLA domain superfamily.** *H. sapiens* (green), *A. thaliana* (blue), *P. falciparum* (PF3D7), *P. berghei* (PBANKA), *P. vivax* (PVX), *P. chabaudi chabaudi* (PCHAS), *P. cynomolgi* (PCYB), *P. knowlesi* (PKH), *T. gondii* (TGME49), and *E. tenella* (ETH), *B. bovis* (BBOV)*, C. parvam* (cgd) is used to perform evolutionary analysis. MEGA software is used to perform Phylogenetic analysis. Sequence alignment is performed using Clustal X and Muscle. NJ method is used to generate the phylogenetic tree. (PDF 34 KB)

Additional file 18:**Phylogenetic analysis for PTH2 domain superfamily.** *H. sapiens* (green), *P. falciparum* (ID PF3D7), *P. berghei* (PBANKA), *P. chabaudi chabaudi* (PCHAS) and *P. knowlesi* (PKH), *B. bovis* (BBOV)*, T. parva* (TP)*, C. parvam* (cgd) is used to perform evolutionary analysis. MEGA software is used to perform Phylogenetic analysis. Sequence alignment is performed using Clustal X and Muscle. NJ method is used to generate the phylogenetic tree. (PDF 28 KB)

Additional file 19:**Phylogenetic analysis for PAP2 domain superfamily.** *H. sapiens* (green), *E.coli* (red), *S. cerevisiae* (pink), *A. thaliana* (blue) and *P. falciparum* (PF3D7), *P. berghei* (PBANKA), *P. vivax* (PVX), *P. chabaudi chabaudi* (PCHAS), *P. cynomolgi* (PCYB), *P. knowlesi* (PKH), *T. gondii* (TGME49), and *E. tenella* (ETH), *B. bovis* (BBOV)*, T. parva* (TP)*, C. parvam* (cgd) is used to perform evolutionary analysis. MEGA software is used to perform Phylogenetic analysis. Sequence alignment is performed using Clustal X and Muscle. NJ method is used to generate the phylogenetic tree. (PDF 51 KB)

Additional file 20:**Phylogenetic analysis for Syja_N domain superfamily.** *H. sapiens* (green), *S. cerevisiae* (pink), *P. falciparum* (PF3D7), *P. berghei* (PBANKA), *P. vivax* (PVX), *P. chabaudi chabaudi* (PCHAS), *P. cynomolgi* (PCYB), *P. knowlesi* (PKH), *T. gondii* (TGME49), and *E. tenella* (ETH), *B. bovis* (BBOV)*, T. parva* (TP)*, C. parvam* (cgd) is used to perform evolutionary analysis. MEGA software is used to perform Phylogenetic analysis. Sequence alignment is performed using Clustal X and Muscle. NJ method is used to generate the phylogenetic tree. (PDF 52 KB)
