# Supplementary material for: Genome wide in silico analysis of Plasmodium falciparum phosphatome
Source: BMC Genomics. 2014 Nov 25;15:1024. doi: 10.1186/1471-2164-15-1024 (PMC4256932; doi:10.1186/1471-2164-15-1024)
Supplement: Supplementary file 3 — Additional file 3:Schematic of all P. falciparum phosphatase super-families.(PDF 191 KB) [file 12864_2014_6717_MOESM3_ESM.pdf]

MPP Superfamily

PF3D7_0107800

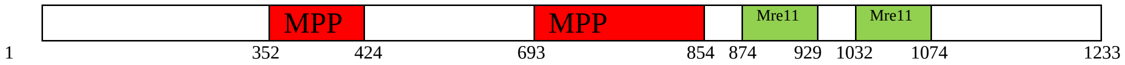

PF3D7_0314400

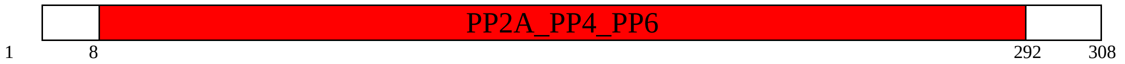

PF3D7_0802800

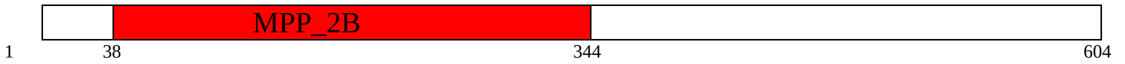

PF3D7_0912400

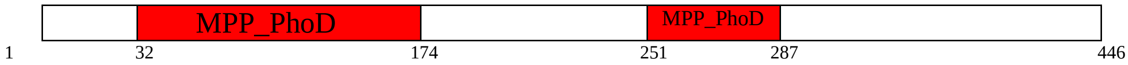

PF3D7_0918000

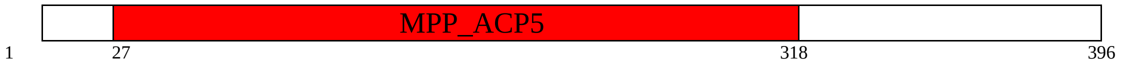

PF3D7_0925400

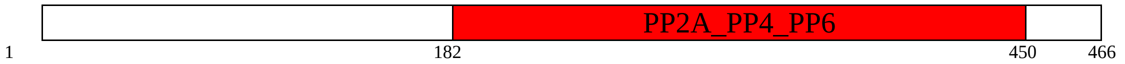

PF3D7_0927700

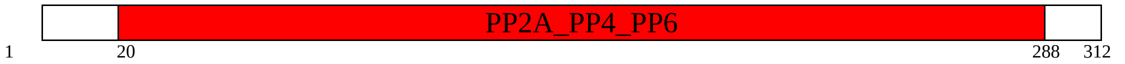

PF3D7_1018200

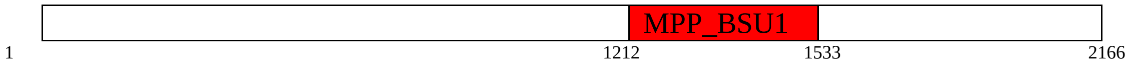

PF3D7_1206000

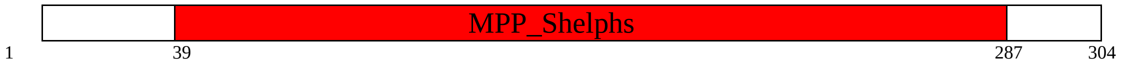

PF3D7_1340600

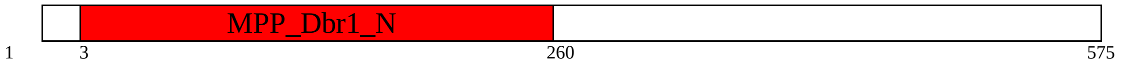

PF3D7_1355500

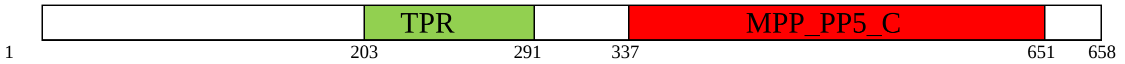

PF3D7_1403900

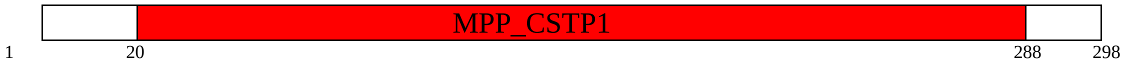

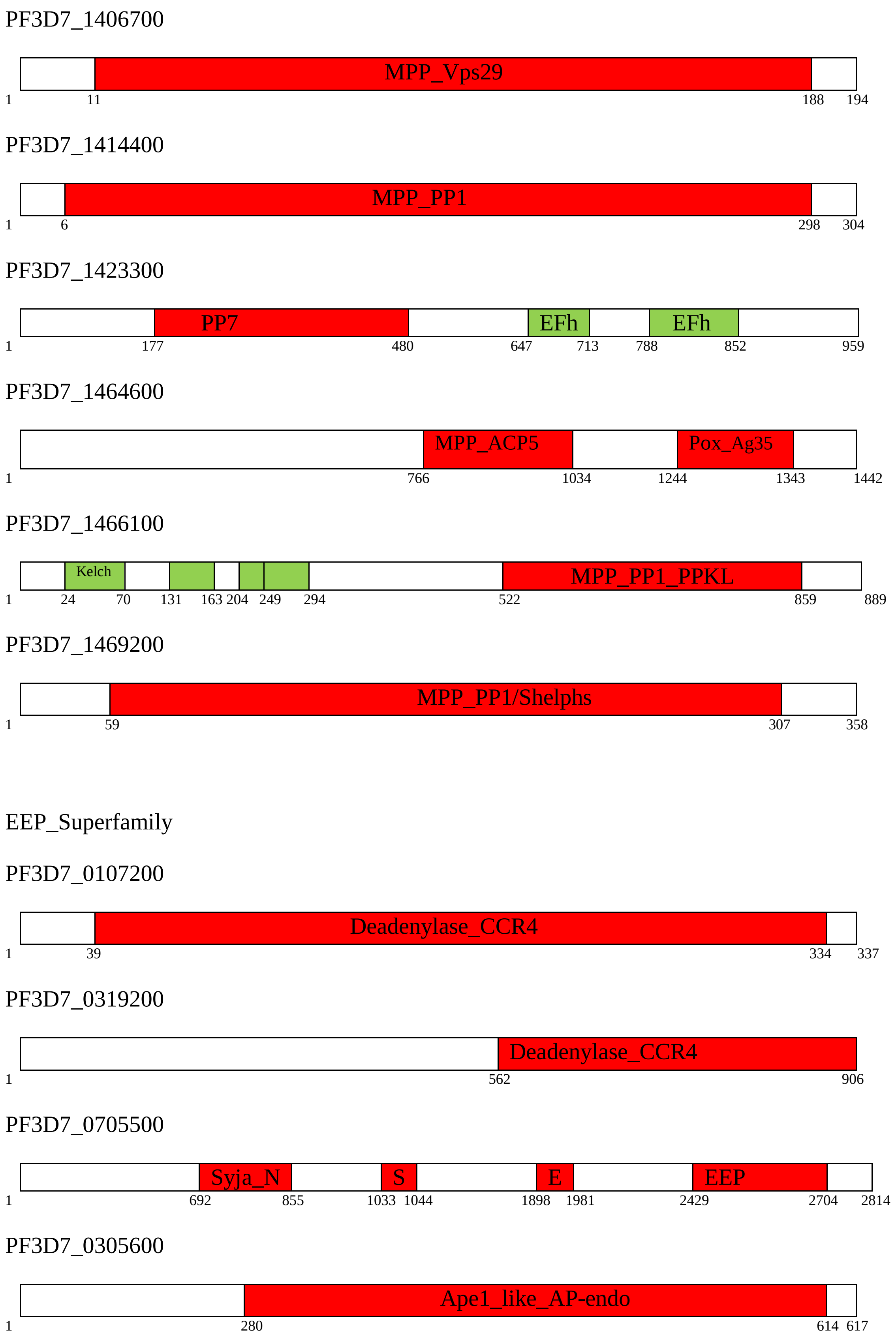
PF3D7_1406700
MPP_Vps29
1 11 188 194
PF3D7_1414400
MPP_PP1
1 6 298 304
PF3D7_1423300
PP7
EFh
EFh
1 177 480 647 713 788 852 959
PF3D7_1464600
MPP_ACP5
Pox_Ag35
1 766 1034 1244 1343 1442
PF3D7_1466100
Kelch
MPP_PP1_PPKL
1 24 70 131 163 204 249 294 522 859 889
PF3D7_1469200
MPP_PP1/Shelphs
1 59 307 358
EEP_Superfamily
PF3D7_0107200
Deadenylase_CCR4
1 39 334 337
PF3D7_0319200
Deadenylase_CCR4
1 562 906
PF3D7_0705500
Syja_N
S
E
EEP
1 692 855 1033 1044 1898 1981 2429 2704 2814
PF3D7_0305600
Ape1_like_AP-endo
1 280 614 617

PF3D7_1111600

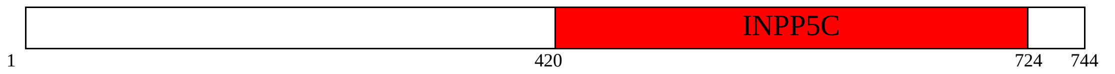

PF3D7_1238600

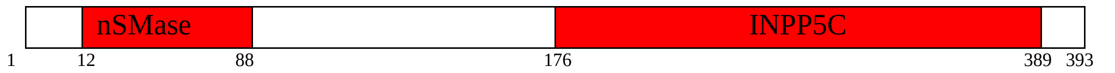

PF3D7_1430600

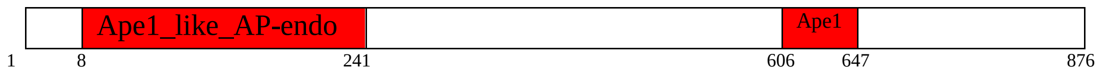

PF3D7_1363500

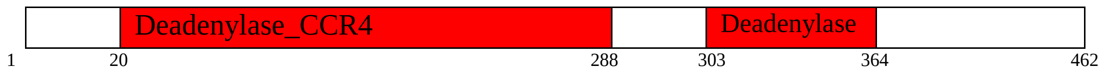

PP2Cc_superfamily

PF3D7_0410300

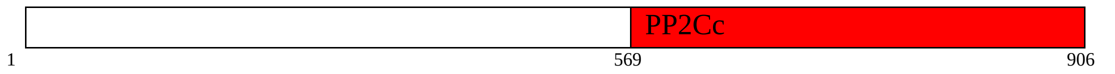

PF3D7_0520100

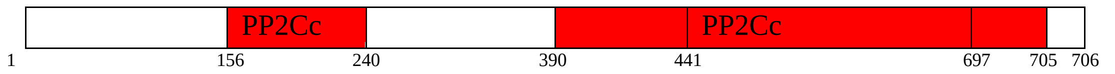

PF3D7_0810300

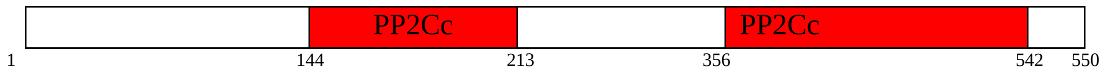

PF3D7_0810500

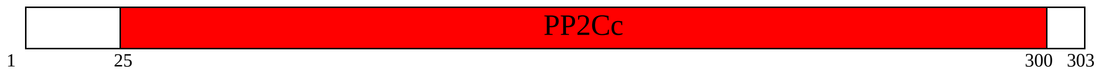

PF3D7_1009600

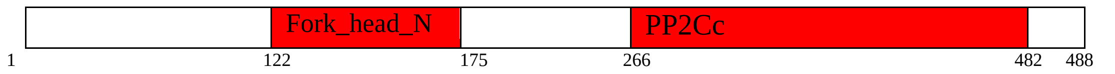

PF3D7_1135100

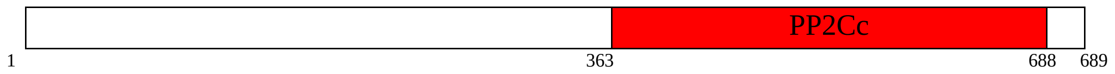

PF3D7_1208900

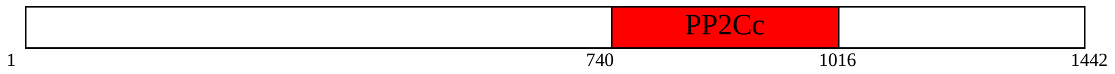

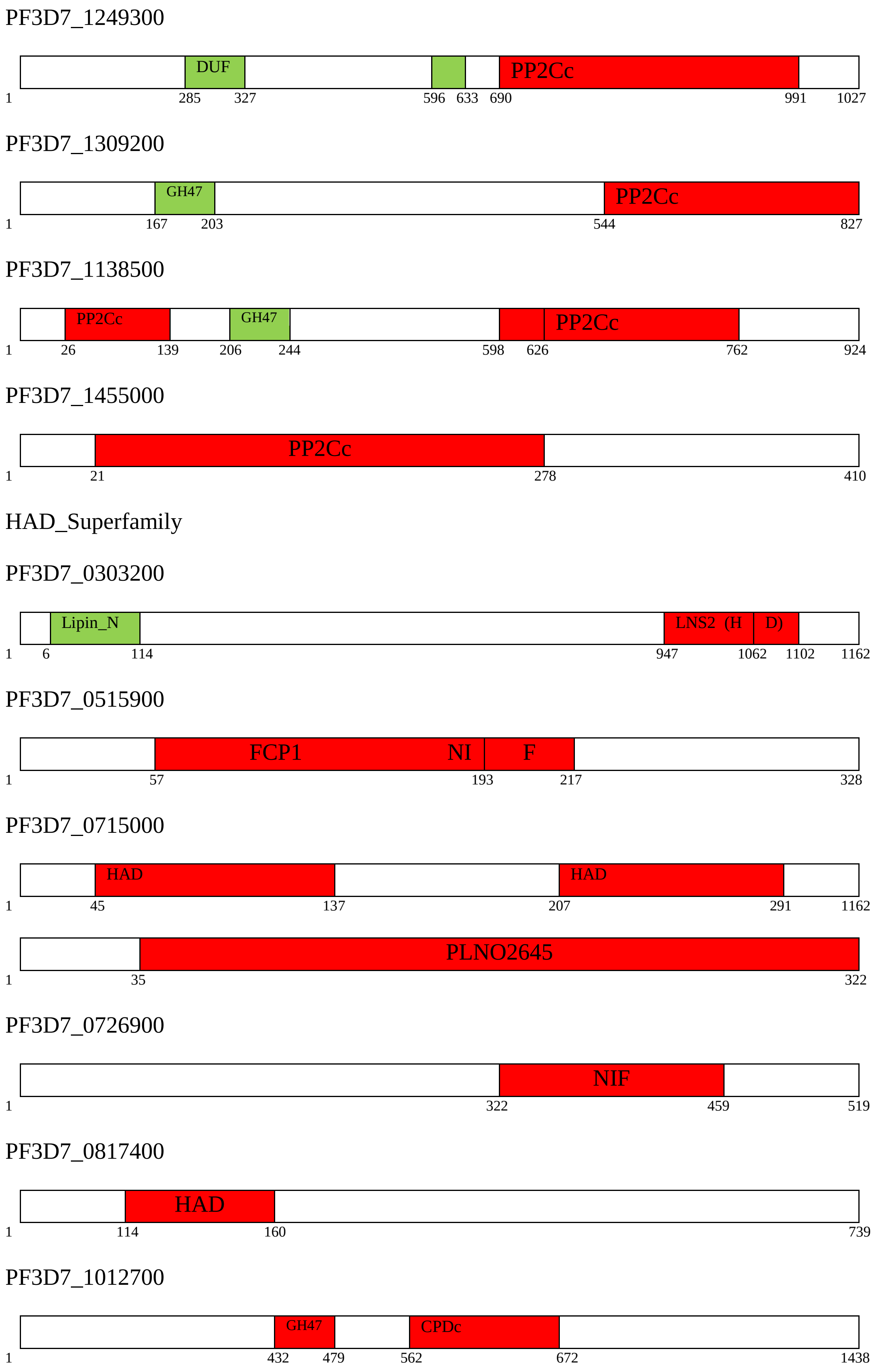
PF3D7_1249300
DUF
PP2Cc
1 285 327 596 633 690 991 1027
PF3D7_1309200
GH47
PP2Cc
1 167 203 544 827
PF3D7_1138500
PP2Cc
GH47
PP2Cc
1 26 139 206 244 598 626 762 924
PF3D7_1455000
PP2Cc
1 21 278 410
HAD_Superfamily
PF3D7_0303200
Lipin_N
LNS2 (H
D)
1 6 114 947 1062 1102 1162
PF3D7_0515900
FCP1
NI
F
1 57 193 217 328
PF3D7_0715000
HAD
HAD
1 45 137 207 291 1162
PLNO2645
1 35 322
PF3D7_0726900
NIF
1 322 459 519
PF3D7_0817400
HAD
1 114 160 739
PF3D7_1012700
GH47
CPDc
1 432 479 562 672 1438

PF3D7_1118400

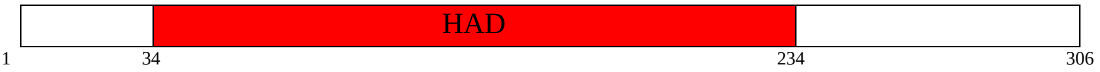

PF3D7_1226100

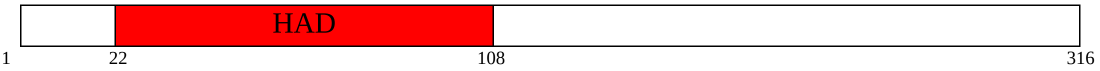

PF3D7_1355700

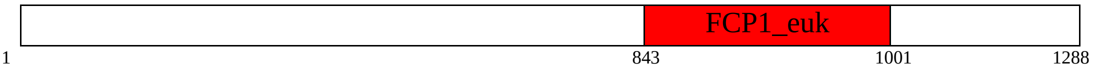

PF3D7_1363200

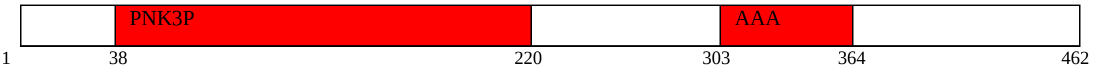

HP_Superfamily

PF3D7_0208400

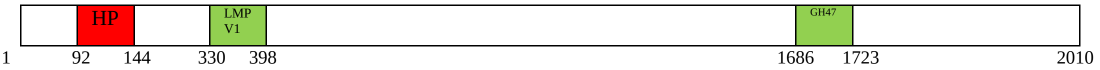

PF3D7_0310300

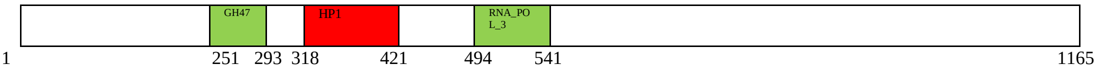

PF3D7_0413500

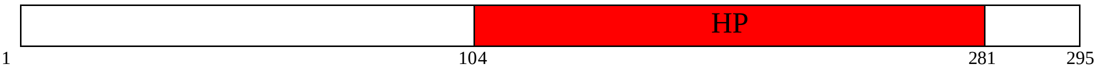

PF3D7_1120100

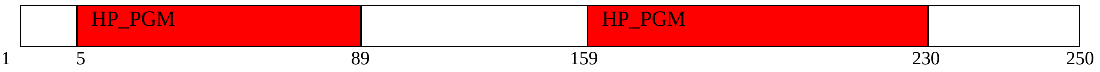

PF3D7_1430300

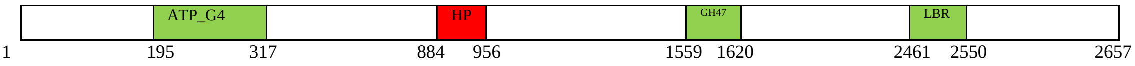

SYJA_N_SUPERFAMILY

PF3D7_0705500

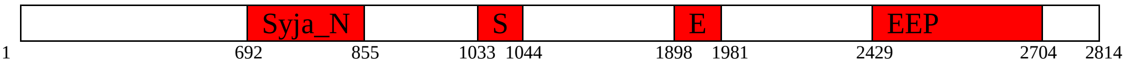

PF3D7_0802500

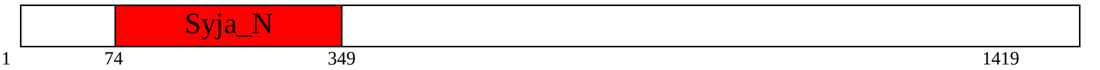

PF3D7_1354200

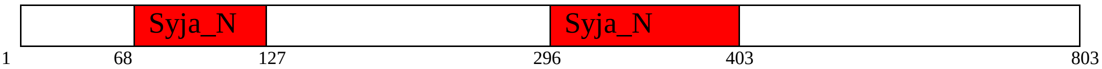

RHOD_SUPERFAMILY

PF3D7_1206400

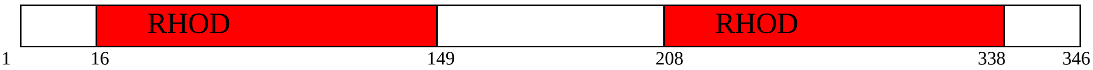

PF3D7_1305500

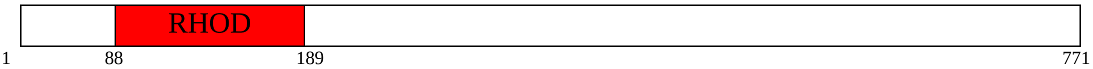

PTPc_Superfamily

PF3D7_0309000

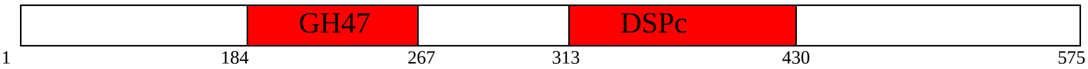

PF3D7_1127000

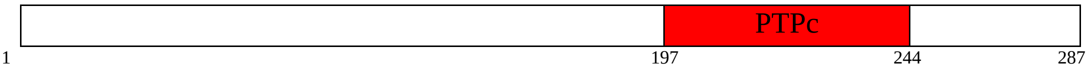

PF3D7_11131000

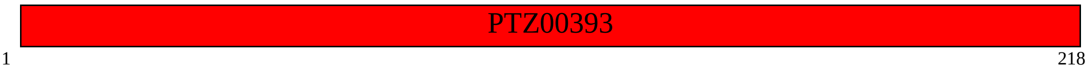

PF3D7_1455100

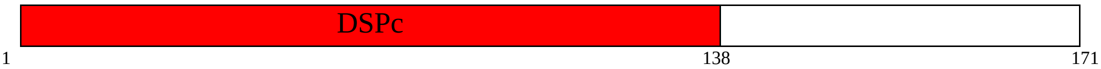

PTPLA_Superfamily

PF3D7_1331000

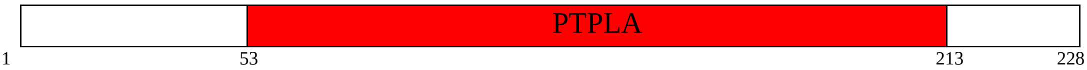

PTH2_Superfamily

PF3D7_0610500

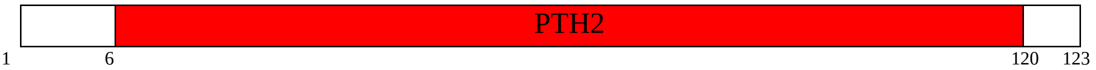

PAP2_C Superfamily

PF3D7_0610500.1

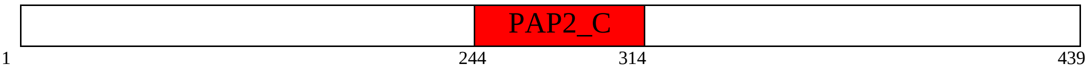

PF3D7_0610500.2

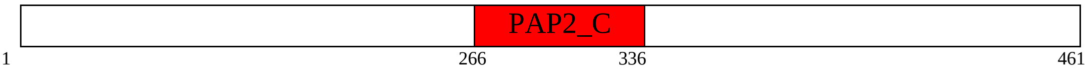

PF3D7_0805600

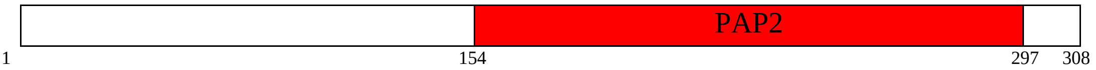

Nucleoside Phosphatase Superfamily

PF3D7_1322000

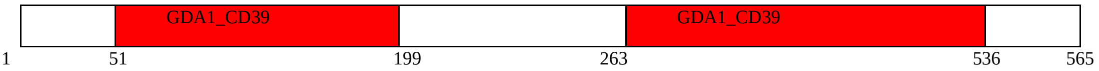

PF3D7_1431800

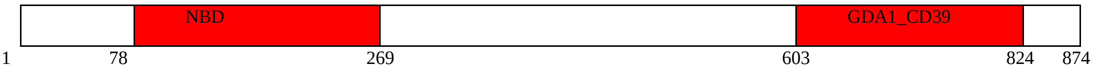

CYTH Superfamily

PF3D7_0322100

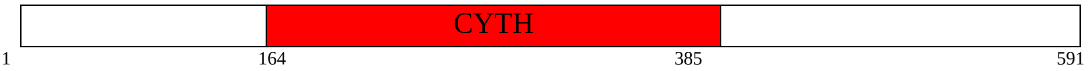

ABBREVIATION

MPP – Metallophosphatase, PTP – Protein Tyrosine Phosphatase, PP2Cc – Protein Phosphatase 2 c, HP – Histidine Phosphatase,

HAD – Haloacid Dehalogenase, PAP – Phosphatidic Acid Phosphatase, EEP – Exonuclease-Endonuclease Phosphatase,

ND – Nucleoside Phosphatase, PTH – Peptidyl-tRNA hydrolase, CYTH-like_pase – Triphosphate Tunnel Metaloenzyme Phosphatase,

RHOD – Rhodanese Homology Domain, PTPLA – Protein Tyrosine phosphatase like protein, Syja_N – SacL Homology Domain,

Mre 11 – Mre 11 nuclease, TPR - Tetratricopeptide Repeat Domain, PP – Protein Phosphatase, EFh – Efh hand calcium binding motif,

Pox-Ag35 – Pox virus Ag35 surface protein, CCR – Carbon Catabolite Repressor protein, INPP5C - Inositol Polyphosphate 5-Phosphatases,

PLNO02645 – Protein Phosphatase 2c, NIF – NLI like Interacting Phosphatase, CPDc – Catalytic domain of ctd like phosphatase,

AAA – ATPase associated cellular activities, PNK3P – Polynucleotide kinase 3 Phosphatase, LMP1 – Latent Membrane Protein 1,

RNA_pol_3 – DNA directed RNA Polymerase III subunit RPC 31, HP_PGM – Histidine Phosphatase Phosphoglycerate Mutase,

LBR – Ice binding protein left handed beta roll, ATP_G4 – ATP Grasp Domain, S – Suya_N , E – EEP, DSPc – Dual specific Phosphatases,

PTZ00393 – Protein Tyrosine Phosphatase, GDA_CD39 – Nucleoside Phosphatase, NBD - NBD Sugar Kinase HSP70 actin,

nSMase – sphingomyelinases, Ape1_like_AP-endo - Apurinic/Apyrimidinic (AP) Endonucleases, GH47 - Glycosyl hydrolase Family 47
