# Supplementary material for: Genome wide in silico analysis of Plasmodium falciparum phosphatome
Source: BMC Genomics. 2014 Nov 25;15:1024. doi: 10.1186/1471-2164-15-1024 (PMC4256932; doi:10.1186/1471-2164-15-1024)
Supplement: Supplementary file 4 — Additional file 4:Conserved domain alignment of Plasmodium specific phosphatases.(PDF 156 KB) [file 12864_2014_6717_MOESM4_ESM.pdf]

PF3D7_0208400 (HP, LMP1, GH47)

Cd Length: 153 **Bit Score:** 45.87 **E-value:** 1.36e-05

```
                                               10        20        30        40        50
                                       ....*....|....*....|....*....|....*....|....*....|....
seqsig_c894f222febf0ec3e38b1c3e2a2e77cc   92 KVIYMRHGARTP-KKKIKNIWpfkeGKGDLTFLGFQQSIKVGEYLRKYYYTFNK 144
Cdd:cd07040                                1 VLYLVRHGEREPnAEGRFTGW----GDGPLTEKGRQQARELGKALRERYIKFDR 50
```

Cd Length: 382 **Bit Score:** 46.17 **E-value:** 6.87e-05

```
                                               10        20        30        40        50        60        70
                                       ....*....|....*....|....*....|....*....|....*....|....*....|....*....|
seqsig_c894f222febf0ec3e38b1c3e2a2e77cc  330 NNKNNDDDNNDDNNNNNNDDNNNNN-NDDNNNDDNNNDNNNNNDDNNNNNNDDDNNyyyyNYNNDETPFN 398
Cdd:pfam05297                            250 DNGPQDPDNTDDNGPQDPDNTDDNGpQDPDNTDDNGPQDPDNTADNGPQDPDNTDD----NGPHDPLPHN 315
```

Cd Length: 522 **Bit Score:** 40.09 **E-value:** 6.42e-03

```
                                               10        20        30
                                       ....*....|....*....|....*....|....*...
seqsig_c894f222febf0ec3e38b1c3e2a2e77cc 1686 DNHDTNNNNNNNNNNNNNSNNNNNNICLKNNKNNIMH 1723
Cdd:PTZ00470                               9 SVHNNADDNYNNNNNNNNQINSNNPNNNGNNQASKLPR 46
```

PF3D7_0520100 (PP2Cc, PP2Cc)

Cd Length: 254 **Bit Score:** 148.24 **E-value:** 2.92e-40

```
                                              10        20        30        40        50        60        70        80
                                      ....*....|....*....|....*....|....*....|....*....|....*....|....*....|....*....|
seqsig_1cebc56cc6f1fc0565117ffc97411f80 441 EKLKFLDELTDEQIMEYIKLAFLKTDEQFLKVSKFPNH----GCTIISLIIFRNKMFVANLGDCRAIgvvnISNTLKTEV 516
Cdd:cd00143                              58 EELEETLTLSEEDIEEALRKAFLRADEEILEEAQDEPDdarsGTTAVVALIRGNKLYVANVGDSRAV----LCRNGEAVQ 133
                                              90       100       110       120       130       140       150       160
                                      ....*....|....*....|....*....|....*....|....*....|....*....|....*....|....*....|
seqsig_1cebc56cc6f1fc0565117ffc97411f80 517 LSNDHKPNDPKEKERIKKMGGDVIclqnVYRVKANarknnkdkpsllerlsmkeevyLAVSRAIGDKDFKFNnvISATPD 596
Cdd:cd00143                             134 LTKDHKPVNEEERERIEKAGGRVS----NGRVPGV----------------------LAVTRALGDFDLKPG--VSAEPD 185
                                             170       180       190       200       210       220       230       240
                                      ....*....|....*....|....*....|....*....|....*....|....*....|....*....|....*....|
seqsig_1cebc56cc6f1fc0565117ffc97411f80 597 VICKEIYSEkcnekkeekeitkrtnvieksdiDEnyfketdnlyysahevnyhYVVMACDGVWDTMSNKDIVKILQTY-- 674
Cdd:cd00143                             186 VTVVKLTED-----------------------DD-------------------FLILASDGLWDVLSNQEAVDIVRSEla 223
                                             250       260       270
                                      ....*....|....*....|....*....|.
seqsig_1cebc56cc6f1fc0565117ffc97411f80 675 NNDPDKACSEIIKTAYAYGSQDNLTAMLLKF 705
Cdd:cd00143                             224 KEDLQEAAQELVDLALRRGSHDNITVVVVRL 254
```

Cd Length: 252 **Bit Score:** 53.53 **E-value:** 4.82e-08

```
                                              10        20        30        40        50        60        70        80
                                      ....*....|....*....|....*....|....*....|....*....|....*....|....*....|....*....|
seqsig_1cebc56cc6f1fc0565117ffc97411f80 156 TMQGRRKKQEDRYLVITDLTkyidsndyktlyfykkNPLYFYSIFDGHRGIKACEYCMSHIIKNIIYYFYNQNMEDDQSS 235
Cdd:smart00332                           15 SMQGVRKPMEDAHVITPDLS----------------DSGGFFGVFDGHGGSEAAKFLSKNLPEILAEELIKEKDELEDVE 78

                                      ....*
seqsig_1cebc56cc6f1fc0565117ffc97411f80 236 TTINK 240
Cdd:smart00332                           79 EALRK 83
```

PF3D7_0817400 (HAD)

```
                                              10        20        30        40
                                      ....*....|....*....|....*....|....*....|....*...
seqsig_1f1c29151dc8a7512f3e3c59917f48e6 114 FDFDGTLIN-KHFSNNHKNNIIFDkERIPILNSLKKKKYEIVVFSNQT 160

Cdd:cd01427                               4 FDLDGTLLDsEPGIAEIEELELYP-GVKEALKELKEKGIKLALATNKS 50
```

PF3D7_1127000 (PTPc)

Cd Length: 105 **Bit Score:** 34.64 **E-value:** 4.63e-03

```
                                              10        20        30        40
                                      ....*....|....*....|....*....|....*....|....*...
seqsig_af78b0293be0b7dcafbf98252903e9f2 197 NLINQIKDLKQKFNTMKNTIffIHCRRGRDRTGEFVSAYKMIEQNKDF 244
Cdd:smart00404                           24 ELLRAVKKNLNQSESSGPVV--VHCSAGVGRTGTFVAIDILLQQLEAE 69
```

PF3D7_1206000 (SHELPHS_MPP)

Cd Length: 208 **Bit Score:** 165.50 **E-value:** 1.40e-49

```
                                              10        20        30        40        50        60        70        80
                                      ....*....|....*....|....*....|....*....|....*....|....*....|....*....|....*....|
seqsig_44cb9be7fec22f1573a259a96417f992  39 FSISDLHSDLDALKKTLLTENIIDEENNAIRENVFVIITGDVLDPAYDDIEILYFIQNYNIKAKPLNSKIQLILGNHEVQ 118
Cdd:cd07425                               1 VAIGDLHGDLDAFREILKGAGVIDSNDHWIGGSTHLVQLGDIFDRGPDVIEILWLLYKLEQEAAKAGGKVHFLLGNHELM 80
                                              90       100       110       120       130       140       150       160
                                      ....*....|....*....|....*....|....*....|....*....|....*....|....*....|....*....|
seqsig_44cb9be7fec22f1573a259a96417f992 119 NICLDF-IGNKKYGEEY----KARNKLFKKGEVLYNYLLDLPFVIKVNDILFSHASIlpyyakrgidyindegrseiknn 193
Cdd:cd07425                              81 NLCGDFrYVHPKYFNEFgglaMRRRELFSPGGELGRWLRSKPVIVKVNDTLFVHGGL----------------------- 137
                                             170       180       190       200       210       220       230       240
                                      ....*....|....*....|....*....|....*....|....*....|....*....|....*....|....*....|
seqsig_44cb9be7fec22f1573a259a96417f992 194 ctllkfkrktgqrfcvccynGPTFNRYFSRaaEMPFRREVCKSLFKTLNKLSAKKLVNGHTIQRNrKVNEYCKGGLIMAD 273
Cdd:cd07425                             138 --------------------GPLWYRGYSK--ETSDKECAAAHLDKVLERLGAKRMVVGHTPQEG-GIVTFCGGKVIRID 194
```

```
                                                   250
                                            ....*....|....
seqsig_44cb9be7fec22f1573a259a96417f992 274 TGISKWKYGVINYV 287
Cdd:cd07425                             195 VGMSKGVYGGPLEV 208
```

PF3D7_1430600 (AP_EEP, AP_EEP)

Cd Length: 309 **Bit Score:** 100.47 **E-value:** 4.61e-23

```
                                                    10        20        30        40        50        60        70        80
                                            ....*....|....*....|....*....|....*....|....*....|....*....|....*....|....*....|
seqsig_54ebe5c5b0ecae34017419b1778c7528   8 SWNVNGWK--KSCEIIKRNDDdLVQFLKKLDIDILCLQETKTNESVIENDCNLLEAdsnmYESYWTCCKKKkgdkthKGY 85
Cdd:cd09088                               4 TWNVNGIRtrLQYQPWNKENS-LKSFLDSLDADIICLQETKLTRDELDEPSAIVEG----YDSFFSFSRGR------KGY 72
                                                    90       100       110       120       130       140       150       160
                                            ....*....|....*....|....*....|....*....|....*....|....*....|....*....|....*....|
seqsig_54ebe5c5b0ecae34017419b1778c7528  86 SGLATYVKnenkiiCSTNNVfddfsfffndyIKKEDLLikkkSEIDKTSISFFLLNDNKKIYNDQNIKCDKNDEhnkkkn 165
Cdd:cd09088                              73 SGVATYCR------DSAATP-----------VAAEEGL----TGVLSSPNQKNELSENDDIGCYGEMLEFTDSK------ 125
                                                   170       180       190       200       210       220       230       240
                                            ....*....|....*....|....*....|....*....|....*....|....*....|....*....|....*....|
seqsig_54ebe5c5b0ecae34017419b1778c7528 166 ktniSVSEFFNEGRILITMHKHFIIVNIYAPY-SGHNYERLYYKVRFFHAVRAKIIQLrIVTGLPIILLGDFNISYRNKD 244
Cdd:cd09088                             126 ----ELLELDSEGRCVLTDHGTFVLINVYCPRaDPEKEERLEFKLDFYRLLEERVEAL-LKAGRRVILVGDVNVSHRPID 200
                                                   250
                                            ....*....|....*...
seqsig_54ebe5c5b0ecae34017419b1778c7528 245 IYYLNNIINLDILLKNIH 262
Cdd:cd09088                             201 HCDPDDSEDFGGESFEDN 218
```

Cd Length: 309 **Bit Score:** 56.56 **E-value:** 1.03e-08

```
                                                    10        20        30        40
                                            ....*....|....*....|....*....|....*....|..
seqsig_54ebe5c5b0ecae34017419b1778c7528 606 DNMIDTFSFFHPNVNGKFTCWDTYRQCRVHNEGSRIDYIFMD 647
Cdd:cd09088                             240 GLLIDSFRYFHPTRKGAYTCWNTLTGARPTNYGTRIDYILAD 281
```
