# Supplementary material for: Genome wide in silico analysis of Plasmodium falciparum phosphatome
Source: BMC Genomics. 2014 Nov 25;15:1024. doi: 10.1186/1471-2164-15-1024 (PMC4256932; doi:10.1186/1471-2164-15-1024)
Supplement: Supplementary file 5 — Additional file 5:Conserved domain alignment of all the P. falciparum phosphatases.(PDF 544 KB) [file 12864_2014_6717_MOESM5_ESM.pdf]

PF3D7_1331000 (PTPLA)

Cd Length: 164 **Bit Score:** 140.09 **E-value:** 2.70e-41

```
                                                    10        20        30        40        50        60        70        80
                                            ....*....|....*....|....*....|....*....|....*....|....*....|....*....|....*....|
seqsig_32eacbc6cf8d2da7e72a0ac2c33bd318  53 QSLAIFEIFFTIIGIINSVVSIVTIQVFSRLFVVYLIFNFLPNT--NKWILSCLIAWAIIDIIRYLFYSLNILNLRFNIL 130
Cdd:pfam04387                             1 QTLAVLEILHAALGLVRSPVLTTFLQVSSRLFVVWGILYSLPEVqsSPAVPSLLIAWSITEIIRYSFYALNLLGFVPYWL 80
                                                    90       100       110       120       130       140       150       160
                                            ....*....|....*....|....*....|....*....|....*....|....*....|....*....|....*....|
seqsig_32eacbc6cf8d2da7e72a0ac2c33bd318 131 ASLRKKLPLILYPIGITSEIVCTLASLNNIYATpflRTYPYSMPNNINFQIDIYYFCIVVLILYIPGSILLYATAVRKSK 210
Cdd:pfam04387                            81 TWLRYTLFIVLYPLGVTSELLLIYQSLPYFEET---KKYSLEMPNALNFSFSYYYFLIFVLLLYIPGFPFLYSHMLKQRR 157

                                            ...
seqsig_32eacbc6cf8d2da7e72a0ac2c33bd318 211 QKI 213
Cdd:pfam04387                           158 KVL 160
```

PF3D7_0615000 (PTH2)

Cd Length: 116 **Bit Score:** 182.60 **E-value:** 5.16e-60

```
                                                    10        20        30        40        50        60        70        80
                                            ....*....|....*....|....*....|....*....|....*....|....*....|....*....|....*....|
seqsig_ba432293d2ec868078c71fc16061002d   6 IIQYILVNKEIIDKK-WPLGSVIAQACHACVAVIAENMDDEIVKDYLSPEHINNMHKVILKIDDTNEIKNLSSILDKESL 84
Cdd:cd02429                               1 LVQYVILRRDLQTKLsWPLGAVIAQACHAAVAVIHLFRSDPDTKKYAYLSNLDNMHKVVLEVPDEAALKNLSSKLTENSI 80
                                                    90       100       110
                                            ....*....|....*....|....*....|....*.
seqsig_ba432293d2ec868078c71fc16061002d  85 KYKIWTEYPENIFTAIALKPYYKNTVRDYFKKYPLL 120
Cdd:cd02429                              81 KHKLWIEQPENIPTCIALKPYPKETVASYLKKLKLL 116
```

CYTH Superfamily

PF3D7_0322100

Cd Length: 243 **Bit Score:** 141.01 **E-value:** 2.62e-38

```
                                                    10        20        30        40        50        60        70        80
                                            ....*....|....*....|....*....|....*....|....*....|....*....|....*....|....*....|
seqsig_c3c8960c90acfa6e185b0d51e029373c 164 ACIYDFLELKTTKSIDKYYVIKNNNSrIRTTTYLNDDNKQETESMMIQSLQKDNLNIWNVYTGNnYDYFDDDEEDDDDDY 243
Cdd:cd07470                              18 SESHLEIEAKLGTIIDKRTGERITLP-VSTDIILADRARTRFESNVTESQHKRINEFLNELVEE-SSKKREKLKYEHSRT 95
                                                    90       100       110       120       130       140       150       160
                                            ....*....|....*....|....*....|....*....|....*....|....*....|....*....|....*....|
seqsig_c3c8960c90acfa6e185b0d51e029373c 244 NNNNNNNNGDTGTKTNIATNNTHGLTTSKSQHIYNNLVDKNDSI-----DYRISINIEYTKPISKLYLSKNTPVHERLKE 318
Cdd:cd07470                              96 RDSFYELPNATGKKTKIRVSYDQKTGRVLACIRKRRLADLDIHSpgspyDIRISINLELPVPGPNSKITGNGPILTRRKD 175
                                                   170       180       190       200       210       220       230
                                            ....*....|....*....|....*....|....*....|....*....|....*....|....*....|..
seqsig_c3c8960c90acfa6e185b0d51e029373c 319 RTTFINTylGLQVDMTKIKTK-----NNELYEVEIEIPSKTIFKAMSNLrnKKDSNYLHFICSNLVNNIRGI 385
Cdd:cd07470                             176 RLSYTHN--AFRIDLTKVTSSepnqhTEESYEVEVELDSKALLDAFDKK--DGDSNLLEELVETLLNNARIL 243
```

RHOD Superfamily

PF3D7_1206400 (RHOD)

Cd Length: 122 **Bit Score:** 63.41 **E-value:** 1.42e-12

```
                                                    10        20        30        40        50        60        70        80
                                            ....*....|....*....|....*....|....*....|....*....|....*....|....*....|....*....|
seqsig_2e7879156c77acd846b5f22f608b9234  16 LIESDKLYDIIKNKEECLLfDTSYYNIHNINNEDifddYDnVESIEGSIPMN--SIISHNenSNFSFFFPTKNEFFIYLK 93
Cdd:cd01448                               1 LVSPDWLAEHLDDPDVRIL-DARWYLPDRDGRKE----YL-EGHIPGAVFFDldEDLDDK--SPGPHMLPSPEEFAELLG 72
                                                    90       100       110       120       130
                                            ....*....|....*....|....*....|....*....|....*....|....*.
seqsig_2e7879156c77acd846b5f22f608b9234  94 NILIKNKRnitmlleniPIILYEKEDIFYSPRIWFIFKMYGFKNVQILNGGLNKWI 149
Cdd:cd01448                              73 SLGISNDD---------TVVVYDDGGGFFAARAWWTLRYFGHENVRVLDGGLQAWK 119
```

Cd Length: 118 **Bit Score:** 52.25 **E-value:** 1.00e-08

```
                                                    10        20        30        40        50        60        70        80
                                            ....*....|....*....|....*....|....*....|....*....|....*....|....*....|....*....|
seqsig_2e7879156c77acd846b5f22f608b9234 208 YYYEDIQNLIKLKeqkqmqNYLLVDTRPNKSFSTLISINENIKVNNFIPFSINIPYNHFLNYHYenykyvTFKNMKDIKI 287
Cdd:cd01449                               1 VTAEEVLANLDSG------DVQLVDARSPERFRGEVPEPRPGLRSGHIPGAVNIPWTSLLDEDG------TFKSPEELRA 68
                                                    90       100       110       120       130
                                            ....*....|....*....|....*....|....*....|....*....|.
seqsig_2e7879156c77acd846b5f22f608b9234 288 LLEKYDLLNDQKIIIStCNKGISACLILFLLNLFDKPfsKLILYPGSLVEY 338
Cdd:cd01449                              69 LFAALGITPDKPVIVY-CGSGVTACVLLLALELLGYK--NVRLYDGSWSEW 116
```

PF3D7_1305500 (RHOD)

Cd Length: 106 **Bit Score:** 57.89 **E-value:** 4.01e-10

```
                                 10        20        30        40        50        60        70        80
                         ....*....|....*....|....*....|....*....|....*....|....*....|....*....|....*....|
lcl|local_MEYKSIDFEE  94 NKSVYILDIRKEDLFNQGHIKSSINIYNKKMMIQINKEMICK------DNLKIIFYDQnNMNNIYDDCINLYNVYFSNIK 167
Cdd:pfam00581         11 DDDVVLIDVRSPEEYAKGHIPGAVNIPLPLPSLDKLESELEElgkkldKDKTIVVYCE-SGNRSGQAAALLLALLLKALG 89
                                 90
                         ....*....|....*..
lcl|local_MEYKSIDFEE 168 VENIYILKGGYEDFERE 184
Cdd:pfam00581         90 YKNVYVLDGGFEAWKAE 106
```

NBD Sugar Kinase HSP30 Superfamily

PF3D7_1322000 (GDA1_CD39)

Cd Length: 424 **Bit Score:** 88.21 **E-value:** 4.84e-19

```
                                                    10        20        30        40        50        60        70        80
                                            ....*....|....*....|....*....|....*....|....*....|....*....|....*....|....*....|
seqsig_6e888ed45e251b3fbd155b6d66d10fad  51 AIVIDAGSTGTRIHIYNYhivDDEKGNIKIYIPSINYR--TTPGLvyilnRYFSGEKEDFHNYFKNIKSFIYDNVEEQKR 128
Cdd:pfam01150                            11 GVVIDAGSSGTRLHVYKW---KDEDLDLLQIVPLIEEFkkLEPGL-----SSFATKPEEAAKYLTPLLEFAEEVIPDSQL 82
                                                    90       100       110       120       130       140       150       160
                                            ....*....|....*....|....*....|....*....|....*....|....*....|....*....|....*....|
seqsig_6e888ed45e251b3fbd155b6d66d10fad 129 FNTIILFRASGGFRLLSINESEKYMNFLKNYfFTHFNEFLLIDDlLVNVLSGKEEAILSFVSIYALLQNFNPSPliftdn 208
Cdd:pfam01150                            83 SETPVFLGATAGMRLLPEDASEKILRALRNG-LKSLSTFPVDDQ-GVRIIDGAEEGLYGWITVNYLLGRFGKDP------ 154
                                                   170       180       190       200       210       220       230       240
                                            ....*....|....*....|....*....|....*....|....*....|....*....|....*....|....*....|
seqsig_6e888ed45e251b3fbd155b6d66d10fad 209 dinegkqndvnndnnndhnndhnndhnnddnnnddnnnndhnndhnnnNNDSDNTIGVLELGGATAQIVIKVplskmndd 288
Cdd:pfam01150                           155 ------------------------------------------------EQCRQSTVGVIDLGGASTQIAFEP-------- 178
                                                   250       260       270       280       290       300       310       320
                                            ....*....|....*....|....*....|....*....|....*....|....*....|....*....|....*....|
seqsig_6e888ed45e251b3fbd155b6d66d10fad 289 ivnlfnyqHKEKKKNSIIEENykNKNIVKINLFNQDIFLYCKSYLVLGRQNAMKTYLHYILHKHKEIDQNNkfiemACFP 368
Cdd:pfam01150                           179 --------QEGFVIASKVEDG--NLYLQQERLYGEKYDVYVHSFLGYGANEALRKYLAKLISNLSNLILSD-----PCLP 243
                                                   330       340       350       360       370       380       390       400
                                            ....*....|....*....|....*....|....*....|....*....|....*....|....*....|....*....|
seqsig_6e888ed45e251b3fbd155b6d66d10fad 369 KNFkfhinnlyKTSIEEDLLEYdgntkindDEYIGVGIGNINMCRQEIQTIL-DYAQIDDLP--F------KIKKFIKLY 439
Cdd:pfam01150                           244 PGF--------NKTVSYSEVEF--------DVFAIRGTGNWEQCSNSIRELLnKNAVCPYEQctFngvhapSIGALQKNI 307
                                                   410       420       430       440       450       460       470       480
                                            ....*....|....*....|....*....|....*....|....*....|....*....|....*....|....*....|
seqsig_6e888ed45e251b3fbd155b6d66d10fad 440 GIENFHHFAVDILNIAESFNPISLNThmYLEKAQEVCPLTIEEI-RKVVRPESNIEKAQTSCFGLIFLYEFMRYILKIDK 518
Cdd:pfam01150                           308 GASSYFYTTGDFFGLVGEYEVASPEK--LTDKAKEACSKNWEDIkSGYPKTLDKNVSEEYACFDLAYILSLLHDGFSLDP 385
                                                   490
                                            ....*....|....*....
seqsig_6e888ed45e251b3fbd155b6d66d10fad 519 SI-LFYSTNYINKTSITWT 536
Cdd:pfam01150                           386 TSeLIQSVKKIAGSEAGWT 404
```

PF3D7_1431800 (NBD, GDA1_CD39)

Cd Length: 185 **Bit Score:** 49.12 **E-value:** 8.08e-07

```
                                                    10        20        30        40        50        60        70        80
                                            ....*....|....*....|....*....|....*....|....*....|....*....|....*....|....*....|
seqsig_935bfd7dc988bfd33b4de35da3edf02e  78 IIIDAGSNGTRIHLFEWKKReyelsnkkeennlIELKEIFNAKVKKSISTISYNEIKDILIYLINKVIDHLIEKKIYVyn 157
Cdd:cd00012                               1 LGIDIGSTSTKAGVADLDGE-------------ILPEEIVPTPVGRPGAVTDLDELEEALRELLKEALRQLKSEIDAV-- 65
                                                    90       100       110       120       130       140       150       160
                                            ....*....|....*....|....*....|....*....|....*....|....*....|....*....|....*....|
seqsig_935bfd7dc988bfd33b4de35da3edf02e 158 kqkwksypfYFQATGGMRNLKQE---DRNLRMKYIKNILSNDNYNPFYFLNeyarilsgeeEGIYGWLAVNnllnsIFSK 234
Cdd:cd00012                              66 ---------GITEPGGVPKENREviiLPNLLLIPLALALEDLGGVPVAVVN----------DAVAAALAEG-----LFGK 121
                                                   170       180       190
                                            ....*....|....*....|....*....|....*
seqsig_935bfd7dc988bfd33b4de35da3edf02e 235 PNNTYGAIDLGGSSTQITFYPMDHNILENYNSILL 269
Cdd:cd00012                             122 EEDTVLVVDLGTGTTGIAIVEDGKGGVGAAGELGI 156
```

Cd Length: 424 **Bit Score:** 53.55 **E-value:** 1.23e-07

```
                                                    10        20        30        40        50        60        70        80
                                            ....*....|....*....|....*....|....*....|....*....|....*....|....*....|....*....|
seqsig_935bfd7dc988bfd33b4de35da3edf02e 603 NKISNTLKNEIIK-----RLFEKIINEKikKLYINITvRIVGSNDFKKCLENTKKLFYEQ-PCFLSSCSFNGIYQPNLEN 676
Cdd:pfam01150                           226 AKLISNLSNLILSdpclpPGFNKTVSYS--EVEFDVF-AIRGTGNWEQCSNSIRELLNKNaVCPYEQCTFNGVHAPSIGA 302
                                                    90       100       110       120       130       140       150       160
                                            ....*....|....*....|....*....|....*....|....*....|....*....|....*....|....*....|
seqsig_935bfd7dc988bfd33b4de35da3edf02e 677 NK---------FVLHGQFKKVITYLGFKkYVDLNQMkiyIQKLCNMNLYE-LTYNMSNKIMHNQIPTFCWKSIWSYSLLF 746
Cdd:pfam01150                           303 LQknigassyfYTTGDFFGLVGEYEVAS-PEKLTDK---AKEACSKNWEDiKSGYPKTLDKNVSEEYACFDLAYILSLLH 378
                                                   170       180       190       200       210       220       230
                                            ....*....|....*....|....*....|....*....|....*....|....*....|....*....|....*...
seqsig_935bfd7dc988bfd33b4de35da3edf02e 747 YGFKFKETTKLLiindntnisydssstsqaskQFYKRVENKEqnnylhdktnynneynlnnkidnISWTHGSMIYQIN 824
Cdd:pfam01150                           379 DGFSLDPTSELI--------------------QSVKKIAGSE-----------------------AGWTLGAMLYLTN 413
```

PAP2 Superfamily

PF3D7_0625000.1

Cd Length: 72 **Bit Score:** 75.29 **E-value:** 6.71e-17

```
                                                    10        20        30        40        50        60        70
                                            ....*....|....*....|....*....|....*....|....*....|....*....|....*....|.
seqsig_53c677908e5e69fc6cdace9faacb727e 244 CTDLVISGHTAFTTLLTFFWFFYERNIYVKTTIFLYSIYIYIIIIISRFHYTVDVLMGYVFGGSVFLFYHY 314
Cdd:pfam14360                             2 CGDLIFSGHTVFTTLAFLFIWEYSPRWFLKVIAWLLSAIGYFLIIASRFHYTVDVLLGYYITTLVFFLYHT 72
```

PF3D7_0625000.2

Cd Length: 72 **Bit Score:** 75.68 **E-value:** 4.88e-17

```
                                                    10        20        30        40        50        60        70
                                            ....*....|....*....|....*....|....*....|....*....|....*....|....*....|.
seqsig_038ecd74a06785798d154f4e21191911 266 CTDLVISGHTAFTTLLTFFWFFYERNIYVKTTIFLYSIYIYIIIIISRFHYTVDVLMGYVFGGSVFLFYHY 336
Cdd:pfam14360                             2 CGDLIFSGHTVFTTLAFLFIWEYSPRWFLKVIAWLLSAIGYFLIIASRFHYTVDVLLGYYITTLVFFLYHT 72
```

PF3D7_0805600

Cd Length: 159 **Bit Score:** 91.57 **E-value:** 1.54e-22

```
                                                10        20        30        40        50        60        70        80
                                        ....*....|....*....|....*....|....*....|....*....|....*....|....*....|....*....|
seqsig_af85922267ec962e96396637d3da7ea8 154 NNFSSVIATVYGYVPFLIMILTVFGFIITFNKNLLYIIFIMPTQTLISdLFLKRIFKKPRPINSALPT---YGMPSSHS- 229
Cdd:cd03382                              11 GDLLSFLLAYLSLLPVAILVGYATLILFRRELEAIYLFIGLLANEALN-YVLKRIIKEPRPCSGAYFVrsgYGMPSSHSq 89
                                                90       100       110       120       130       140       150
                                        ....*....|....*....|....*....|....*....|....*....|....*....|....*....|.
seqsig_af85922267ec962e96396637d3da7ea8 230 ---SFAIALLTFLLLHITEQKKDKWSIITYVIATLTLLPIPWSRVEVEDHTVLQVIVGSLVGIGFGfIFYF 297
Cdd:cd03382                              90 fmgFFAVYLLLFIYLRLGRLNSLVSRFLLSLGLLLLALLVSYSRVYLGYHTVSQVVVGAIVGILLG-ILWF 159
```

Syja_N Superfamily

PF3D7_0705500 (Syja_N, EEP, EEP)

Cd Length: 299 **Bit Score:** 177.80 **E-value:** 2.46e-48

```
                                                  10        20        30        40        50        60        70        80
                                           ....*....|....*....|....*....|....*....|....*....|....*....|....*....|....*....|
seqsig_b411119b5302348402f69874072210e9  692 NEDVRNDEKKNNSKMDIHKYLKTIQKLL-SVHMYYSYDYDLTQCIQKKVKNNIEEveveplvyntrlnnlnnkkkKSFLK 770
Cdd:pfam02383                             45 NSSEYDSESDKKSSKDEEHYLKLLKKLLsSGSFYFSYDYDLTNSLQRRGLRESSS--------------------LSLWK 104
                                                  90       100       110       120       130       140       150       160
                                           ....*....|....*....|....*....|....*....|....*....|....*....|....*....|....*....|
seqsig_b411119b5302348402f69874072210e9  771 MCEKKYVWNYQMIKKTKNKCKIDDNWFCSIIQGYISYTSIEINKKCLELLLISRRSSILGGTRFNKRGINDDGYVANYVE 850
Cdd:pfam02383                            105 RADDRFVWNSYLLKPLIDFRSDLSDWLLPLIQGFVEQRTISVNGKKVTLTLISRRSRKRAGTRYNRRGIDDDGNVANFVE 184
                                                 170       180       190       200       210       220       230       240
                                           ....*....|....*....|....*....|....*....|....*....|....*....|....*....|....*....|
seqsig_b411119b5302348402f69874072210e9  851 SEQIVRINnrninhvdkvdqlnnkdntsmsdinydsyknknftstkgnldknnsnrnntynnsnvsneilaspnlthdqv 930
Cdd:pfam02383                            185 TEQIVSTN------------------------------------------------------------------------ 192
                                                 250       260       270       280       290       300       310       320
                                           ....*....|....*....|....*....|....*....|....*....|....*....|....*....|....*....|
seqsig_b411119b5302348402f69874072210e9  931 nhlkensvrekdknnnsfgssqvvggnistetkeqfvnkdkinqgvfgtykmndsydnnnnnnnnnnntntntnnnnnn 1010
Cdd:pfam02383                                --------------------------------------------------------------------------------
                                                 330       340       350       360       370       380       390       400
                                           ....*....|....*....|....*....|....*....|....*....|....*....|....*....|....*....|
seqsig_b411119b5302348402f69874072210e9 1011 nnnnnnnnnsssstnkyvgrnfENRIISLVQIRGSIPLFWKQ---HSMSSHVNIQRSSLLSIKAFKEHNKKLINSYGnNI 1087
Cdd:pfam02383                            193 ----------------------SGRIFSFVQIRGSVPLFWEQdpnLLYKPKIKITRSSEATQPAFDKHFDDLIKRYG-PI 249
                                                 410       420       430       440       450
                                           ....*....|....*....|....*....|....*....|....*....|....*..
seqsig_b411119b5302348402f69874072210e9 1088 YYINLLSQnKSNEKKLTKKMIEMINFIKKDKHykekdyINYIEYDFHISVKNKSFED 1144
Cdd:pfam02383                            250 YIVNLLDK-KGSEKKLSEAYEEAINYLNENKK------IKYTWFDFHAECKGMKFDN 299
```

Cd Length: 328 **Bit Score:** 170.65 **E-value:** 1.16e-45

```
                                                  10        20        30        40        50        60        70        80
                                           ....*....|....*....|....*....|....*....|....*....|....*....|....*....|....*....|
seqsig_b411119b5302348402f69874072210e9 2429 RKFKYVKLKSVSMIGLFIIIFIDEALVDHIREIEVCKVKVGLKGNTGNKGSVSVKFRLGYNSFCFNNIHLASGQTNIFER 2508
Cdd:cd09089                               90 RDHKYVLLTSEQLVGVCLFVFVRPQHAPFIRDVAVDTVKTGLGGAAGNKGAVAIRFLLHSTSLCFVCSHFAAGQSQVKER 169
                                                  90       100       110       120       130       140       150       160
                                           ....*....|....*....|....*....|....*....|....*....|....*....|....*....|....*....|
seqsig_b411119b5302348402f69874072210e9 2509 NTQMQNILSN-SFQNQQlnNLFNFDYFFACGDFNFRINKNLEEVLKLISSKNIKHLLNYDQFIYNK-MYNIlpFCLFHEH 2586
Cdd:cd09089                              170 NEDFAEIARKlSFPMGR--TLDSHDYVFWCGDFNYRIDLPNDEVKELVRNGDWLKLLEFDQLTKQKaAGNV--FKGFLEG 245
                                                 170       180       190       200       210       220       230       240
                                           ....*....|....*....|....*....|....*....|....*....|....*....|....*....|....*....|
seqsig_b411119b5302348402f69874072210e9 2587 PITFNPTYKYKKHSNMYDIR---RTPAWCDRVLMsgklvhlseiEKKRNEHISREHYRERNISiDQTNKSDMVDYKRNNM 2663
Cdd:cd09089                              246 EINFAPTYKYDLFSDDYDTSekcRTPAWTDRVLW----------RRRKWPSDKTEESLVETND-PTWNPGTLLYYGRAEL 314
                                                 250       260       270       280
                                           ....*....|....*....|....*....|....*....|.
seqsig_b411119b5302348402f69874072210e9 2664 sdkdilndfynndkiyfkyldyKThnnffsSDHKPVSALIE 2704
Cdd:cd09089                              315 ----------------------KT------SDHRPVVAIID 327
```

Cd Length: 291 **Bit Score:** 48.49 **E-value:** 1.31e-05

```
                                                  10        20        30        40        50        60        70        80
                                           ....*....|....*....|....*....|....*....|....*....|....*....|....*....|....*....|
seqsig_b411119b5302348402f69874072210e9 1898 IKLWAGTWNLCGGDLEElhDISSWL--NEVDDYIDMYVFCFQEVVELTGFRILMNmkDKFKEKKIEQMITQTLgevSQRQ 1975
Cdd:cd09090                                1 INIFVGTFNVNGKSYKD--DLSSWLfpEENDELPDIVVIGLQEVVELTAGQILNS--DPSKSSFWEKKIKTTL---NGRG 73

                                           ....*.
seqsig_b411119b5302348402f69874072210e9 1976 KELYLR 1981
Cdd:cd09090                               74 GEKYVL 79
```

PF3D7_0802500

Cd Length: 299 **Bit Score:** 215.16 **E-value:** 8.23e-62

```
                                                 10        20        30        40        50        60        70        80
                                          ....*....|....*....|....*....|....*....|....*....|....*....|....*....|....*....|
seqsig_3973f35e7592a0e87396a5e1176f7b2a  74 CEGIFGCIRFLNYPYLYVLIKKEKVGILFdEHKIYNVKNILLIPFVEDIFD------NYNEENELIDLFYNNTNHKYIYF 147
Cdd:pfam02383                             1 IYGILGLIRLLGGSYLIVITKRSKVGQIR-GHTIYKITSVEFIPLNSSEYDsesdkkSSKDEEHYLKLLKKLLSSGSFYF 79
                                                 90       100       110       120       130       140       150       160
                                          ....*....|....*....|....*....|....*....|....*....|....*....|....*....|....*....|
seqsig_3973f35e7592a0e87396a5e1176f7b2a 148 SYTYNLTYSVQENYFIQKNylKGGNVKYKNNPYMWNSYHSKYFIKQNIFL---CLSIINGYFIQSKFLCSGKIIDISLVG 224
Cdd:pfam02383                            80 SYDYDLTNSLQRRGLRESS--SLSLWKRADDRFVWNSYLLKPLIDFRSDLsdwLLPLIQGFVEQRTISVNGKKVTLTLIS 157
                                                170       180       190       200       210       220       230       240
                                          ....*....|....*....|....*....|....*....|....*....|....*....|....*....|....*....|
seqsig_3973f35e7592a0e87396a5e1176f7b2a 225 RRSNKYAGTRFRKRGLNSYGYSANDVESEIILFekNNSHVILSYTQLRGSVPIFWNQQVNYKILKKPQINfLKTDINYSC 304
Cdd:pfam02383                           158 RRSRKRAGTRYNRRGIDDDGNVANFVETEQIVS--TNSGRIFSFVQIRGSVPLFWEQDPNLLYKPKIKIT-RSSEATQPA 234
                                                250       260       270       280       290
                                          ....*....|....*....|....*....|....*....|....*....|....*.
seqsig_3973f35e7592a0e87396a5e1176f7b2a 305 TQKHFQRLYEKYGyPITVVNLLSKKKYsdEQKLSYHYKESIDRLNKYipKKIHIIY 360
Cdd:pfam02383                           235 FDKHFDDLIKRYG-PIYIVNLLDKKGS--EKKLSEAYEEAINYLNEN--KKIKYTW 285
```

PF3D7_1354200

Cd Length: 299 **Bit Score:** 247.52 **E-value:** 5.24e-75

```
                                                    10        20        30        40        50        60        70        80
                                            ....*....|....*....|....*....|....*....|....*....|....*....|....*....|....*....|
seqsig_4a6afdc7dfd92254328c38dd26d6f8cf  68 FYGCLGIIKAENINFLVIVSEAEVVCNIFNRSIYRIKRISFVQLNcekmnkntnelyeyeicylgSNGNLTSLNNIKKDE 147
Cdd:pfam02383                             1 IYGILGLIRLLGGSYLIVITKRSKVGQIRGHTIYKITSVEFIPLN--------------------SSEYDSESDKKSSKD 60
                                                    90       100       110       120       130       140       150       160
                                            ....*....|....*....|....*....|....*....|....*....|....*....|....*....|....*....|
seqsig_4a6afdc7dfd92254328c38dd26d6f8cf 148 NKKFfcnknkiqtknyfdnlfqstnfllencvykynrfyynetyihkfknctlkedvLKIITYFLhafNKGPFYFSYYYN 227
Cdd:pfam02383                            61 EEHY-----------------------------------------------------LKLLKKLL---SSGSFYFSYDYD 84
                                                   170       180       190       200       210       220       230       240
                                            ....*....|....*....|....*....|....*....|....*....|....*....|....*....|....*....|
seqsig_4a6afdc7dfd92254328c38dd26d6f8cf 228 LTISLQNQYMNEldkkndmnkkkkdylkisehaskklniysnknnnndnnnndndnndndnnnNVNKIQFNEINDEYTWN 307
Cdd:pfam02383                            85 LTNSLQRRGLRE---------------------------------------------------SSSLSLWKRADDRFVWN 113
                                                   250       260       270       280       290       300       310       320
                                            ....*....|....*....|....*....|....*....|....*....|....*....|....*....|....*....|
seqsig_4a6afdc7dfd92254328c38dd26d6f8cf 308 WKILDTFKN--VDAYGFVVFLIHGYINTNIFHVeDNKKISLYLISRKCKNRSGVRFWCRGSNENGDVANFVETEQIVVck 385
Cdd:pfam02383                           114 SYLLKPLIDfrSDLSDWLLPLIQGFVEQRTISV-NGKKVTLTLISRRSRKRAGTRYNRRGIDDDGNVANFVETEQIVS-- 190
                                                   330       340       350       360       370       380       390       400
                                            ....*....|....*....|....*....|....*....|....*....|....*....|....*....|....*....|
seqsig_4a6afdc7dfd92254328c38dd26d6f8cf 386 nKERINIFSYIIVRGSIPVLWKQQPTLSIRPAIHVCPNMSENKRILNLHMKKLQTNYGKISITNLINKKFGEKYLGECFE 465
Cdd:pfam02383                           191 -TNSGRIFSFVQIRGSVPLFWEQDPNLLYKPKIKITRSSEATQPAFDKHFDDLIKRYGPIYIVNLLDKKGSEKKLSEAYE 269
                                                   410       420       430
                                            ....*....|....*....|....*....|
seqsig_4a6afdc7dfd92254328c38dd26d6f8cf 466 NCLSDCN--VEHNFTWFDFHSEFKKLNFEN 493
Cdd:pfam02383                           270 EAINYLNenKKIKYTWFDFHAECKGMKFDN 299
```

PTPc Superfamily

PF3D7_0309000 (DSPc, GH47)

Cd Length: 139 **Bit Score:** 84.58 **E-value:** 3.08e-19

```
                                                    10        20        30        40        50        60        70        80
                                            ....*....|....*....|....*....|....*....|....*....|....*....|....*....|....*....|
seqsig_0f2436048220b55cac4ad807de3f046a 313 RHIYNVC-ELNKCLRENKLIPYnniykmkhLYLNILDTFDENILKHVNKAHLFIDSVIQKKKNILIHCMAGISRCSSIIL 391
Cdd:cd00127                              29 THVLNVAkEVPNENLFLSDFNY--------LYVPILDLPSQDISKYFDEAVDFIDDAREKGGKVLVHCLAGVSRSATLVI 100
                                                    90       100       110
                                            ....*....|....*....|....*....|....*....
seqsig_0f2436048220b55cac4ad807de3f046a 392 SYVSKKNKKGIEYNFNLLKSKYPFAHPNENFYRQLLLYE 430
Cdd:cd00127                             101 AYLMKTLGLSLREAYEFVKSRRPIISPNAGFMRQLKEYE 139
```

Cd Length: 522 **Bit Score:** 45.48 **E-value:** 2.84e-05

```
                                                    10        20        30        40        50        60        70        80
                                            ....*....|....*....|....*....|....*....|....*....|....*....|....*....|....*....|
seqsig_0f2436048220b55cac4ad807de3f046a 184 NKHNNNNNNNNNNNNNNNNNNNNNNNNcctFKNPDISNTSQhhvehiqiHKSNSHSNIPSDN-INFCNKKY----DKNLSRS 258
Cdd:PTZ00470                              5 REHLSVHNNADDNYNNNNNNNNQIN---SNNPNNNGNNQ--------ASKLPRGKKKQENpFNKIDEVYyqneKLNIKRR 73

                                            ....*....
seqsig_0f2436048220b55cac4ad807de3f046a 259 VEISEKDKH 267
Cdd:PTZ00470                             74 ESVREAMKH 82
```

PF3D7_1113100 (PTZ00393)

Cd Length: 241 **Bit Score:** 413.95 **E-value:** 1.17e-147

```
                                                    10        20        30        40        50        60        70        80
                                            ....*....|....*....|....*....|....*....|....*....|....*....|....*....|....*....|
seqsig_b163281da6adfd3ac483de9eeeb1a97d   1 MKSLENNEMHNLCPIHYYFNGRD------------------YNSDTIINNVNIKYFNMDNCLGNANLHMDYLNPVLNHPT 62
Cdd:PTZ00393                              6 DCSTLNCESHPPCNNRISGSSRGrdgvcsrssasipnigrrRNCDQVIHTVYVKYFAMDSYLGNINYNMDYLNPVLNHPT 85
                                                    90       100       110       120       130       140       150       160
                                            ....*....|....*....|....*....|....*....|....*....|....*....|....*....|....*....|
seqsig_b163281da6adfd3ac483de9eeeb1a97d  63 KIEHGKIKILILDAPTNDLLPLYIKEMKNYNVTDLVRTCERTYNDGEIQDAGINVHELIFPDGDAPTEDIVSNWLNIVNN 142
Cdd:PTZ00393                             86 KIEHGKIKILILDAPTNDLLPLYIKEMKNYNVTDLVRTCERTYNDGEITSAGINVHELIFPDGDAPTVDIVSNWLTIVNN 165
                                                   170       180       190       200       210       220       230
                                            ....*....|....*....|....*....|....*....|....*....|....*....|....*....|....*.
seqsig_b163281da6adfd3ac483de9eeeb1a97d 143 VIKNNCAVAVHCVAGLGRAPVLASIVLIEFGMDPIDAIVFIRDRRKGAINKRQLQFLKEYRKKKKKKNCLRKCHFM 218
Cdd:PTZ00393                            166 VIKNNRAVAVHCVAGLGRAPVLASIVLIEFGMDPIDAIVFIRDRRKGAINKRQLQFLKAYKKKKKKKNCLRKCHFM 241
```

PF3D7_1127000 (PTPc)

Cd Length: 105 **Bit Score:** 34.64 **E-value:** 4.63e-03

```
                                                    10        20        30        40
                                            ....*....|....*....|....*....|....*....|....*...
seqsig_af78b0293be0b7dcafbf98252903e9f2 197 NLINQIKDLKQKFNTMKNTIffIHCRRGRDRTGEFVSAYKMIEQNKDF 244
Cdd:smart00404                           24 ELLRAVKKNLNQSESSGPVV--VHCSAGVGRTGTFVAIDILLQQLEAE 69
```

PF3D7_1455100

Cd Length: 139 **Bit Score:** 118.87 **E-value:** 3.59e-34

```
                                                    10        20        30        40        50        60        70        80
                                            ....*....|....*....|....*....|....*....|....*....|....*....|....*....|....*....|
seqsig_41bb8c3adb3efcf2f2d95547876cf902   1 MIQIIPYLYLGKKNDIDNVENLKKNNIKaVVICCTYFEYPEYKIPNGYEILRINLEDIGLENISSYFEESNNFIHSYITK 80
Cdd:cd00127                               2 LSEITPGLYLGSYPAASDKELLKKLGIT-HVLNVAKEVPNENLFLSDFNYLYVPILDLPSQDISKYFDEAVDFIDDAREK 80
                                                    90       100       110       120       130
                                            ....*....|....*....|....*....|....*....|....*....|....*....
seqsig_41bb8c3adb3efcf2f2d95547876cf902  81 EQSVLICCCHGISRSSTISIAYLIGKQNFGLNEAFNFI-MGKKNICPNIGFMEQLCEYE 138
Cdd:cd00127                              81 GGKVLVHCLAGVSRSATLVIAYLMKTLGLSLREAYEFVkSRRPIISPNAGFMRQLKEYE 139
```

HP_Superfamily

PF3D7_0208400 (HP, LMP1, GH47)

Cd Length: 153 **Bit Score:** 45.87 **E-value:** 1.36e-05

```
                                                     10        20        30        40        50
                                             ....*....|....*....|....*....|....*....|....*....|....
seqsig_c894f222febf0ec3e38b1c3e2a2e77cc   92 KVIYMRHGARTP-KKKIKNIWpfkeGKGDLTFLGFQQSIKVGEYLRKYYYTFNK 144
Cdd:cd07040                                1 VLYLVRHGEREPnAEGRFTGW----GDGPLTEKGRQQARELGKALRERYIKFDR 50
```

Cd Length: 382 **Bit Score:** 46.17 **E-value:** 6.87e-05

```
                                                     10        20        30        40        50        60        70
                                             ....*....|....*....|....*....|....*....|....*....|....*....|....*....|
seqsig_c894f222febf0ec3e38b1c3e2a2e77cc  330 NNKNNDDDNNDDNNNNNNDDNNNNN-NDDNNNDDNNNDNNNNNDDNNNNNNDDDNNyyyyNYNNDETPFN 398
Cdd:pfam05297                            250 DNGPQDPDNTDDNGPQDPDNTDDNGpQDPDNTDDNGPQDPDNTADNGPQDPDNTDD----NGPHDPLPHN 315
```

Cd Length: 522 **Bit Score:** 40.09 **E-value:** 6.42e-03

```
                                                     10        20        30
                                             ....*....|....*....|....*....|....*...
seqsig_c894f222febf0ec3e38b1c3e2a2e77cc 1686 DNHDTNNNNNNNNNNNNNNSNNNNNNNICLKNNKNNIMH 1723
Cdd:PTZ00470                               9 SVHNNADDNYNNNNNNNNQINSNNPNNNGNNQASKLPR 46
```

PF3D7_0413500

Cd Length: 153 **Bit Score:** 128.21 **E-value:** 4.16e-36

```
                                                     10        20        30        40        50        60        70        80
                                             ....*....|....*....|....*....|....*....|....*....|....*....|....*....|....*....|
seqsig_5a06addc618532c0481ddac60f885973  104 HIILVRHGQYE----RRYKDDENSKrLTKEGCKQADITGKKLKDIlnNKKVSVIYHSDMIRAKETANIISKYFPDANLIN 179
Cdd:cd07067                                1 RLYLVRHGESEwnaeGRFQGWTDVP-LTEKGREQARALGKRLKEL--GIKFDRIYSSPLKRAIQTAEIILEELPGLPVEV 77
                                                     90       100       110       120       130       140       150       160
                                             ....*....|....*....|....*....|....*....|....*....|....*....|....*....|....*....|
seqsig_5a06addc618532c0481ddac60f885973  180 DPNLNEgtpylpdplprhskfdaqkikednKRINKAYETYFYKPSGdedEYQLVICHGNVIRYFLCRALQIPLFAWLRFS 259
Cdd:cd07067                               78 DPRLRE------------------------ARVLPALEELIAPHDG---KNVLIVSHGGVLRALLAYLLGLSDEDILRLN 130
                                                    170       180
                                             ....*....|....*....|..
seqsig_5a06addc618532c0481ddac60f885973  260 SYNCGITWLVLDDEGSVVLREF 281
Cdd:cd07067                              131 LPNGSISVLELDENGGVLLLR 152
```

PF3D7_0310300 (HP, GH47, RNA_POL3_RPC31)

```
                                                     10        20        30        40        50        60        70        80
                                             ....*....|....*....|....*....|....*....|....*....|....*....|....*....|....*....|
seqsig_e73e603a108e56d6a0feaecfb16de6aa  318 GAFEKNNNF-VIITSPLRRCLETTKYFLN-FKKNIIVYEAVRETagnYYSDQRSKTSDIKKfcDQNFEDYELICFGETDA 395
Cdd:pfam00300                             37 GKRLKGIPFdRIYSSPLLRAIQTAEILAEaLGLPIIVDPRLRER---DFGDWEGLTFDEIK--AEFPEELRAWLEDPADF 111
                                                     90       100
                                             ....*....|....*....|....*.
seqsig_e73e603a108e56d6a0feaecfb16de6aa  396 LSGNkfRETSEQVYCRCLQFLKLIHA 421
Cdd:pfam00300                            112 RPPG--GESLADVYERVEAALEELLA 135
```

Cd Length: 522 **Bit Score:** 47.03 **E-value:** 2.03e-05

```
                                                     10        20        30        40
                                             ....*....|....*....|....*....|....*....|...
seqsig_e73e603a108e56d6a0feaecfb16de6aa  251 KNINNNHNNNNNNNNNNNNHNNNNHNNNNNHNNNNHNNNNHNNNNN 293
Cdd:PTZ00470                               1 NVNLREHLSVHNNADDNYNNNNNNNNQINSNNPNNNGNNQASK 43
```

Cd Length: 221 **Bit Score:** 42.05 **E-value:** 4.09e-04

```
                                                     10        20        30        40        50
                                             ....*....|....*....|....*....|....*....|....*....|..
seqsig_e73e603a108e56d6a0feaecfb16de6aa  494 EKKKKKAEMKDDNEKKEFDENNIKEEEEEEEYDNDDDFTVEN----NKYVDD 541
Cdd:pfam11705                            157 EKKLKELEAEDVDEEDEKDEEEEEEEEEEDEDFDDDDDDDDDdynaENYFDN 208
```

PF3D7_1120100 (HP_PGM, HP_PGM)

Cd Length: 153 **Bit Score:** 128.21 **E-value:** 1.49e-36

```
                                                     10        20        30        40        50        60        70        80
                                             ....*....|....*....|....*....|....*....|....*....|....*....|....*....|....*....|
seqsig_b9437b631427545e290a6399da8d5075    5 TLVLLRHGESTWNKENKFTGWTDVPLSEKGEEEAIAAGKYLKEKNFKFDVVYTSVLKRAICTAWNVLKtaDLLHVPVVKT 84
Cdd:cd07067                                1 RLYLVRHGESEWNAEGRFQGWTDVPLTEKGREQARALGKRLKELGIKFDRIYSSPLKRAIQTAEIILE--ELPGLPVEVD 78

                                             ....*
seqsig_b9437b631427545e290a6399da8d5075   85 WRLNE 89
Cdd:cd07067                               79 PRLRE 83
```

Cd Length: 153 **Bit Score:** 75.82 **E-value:** 2.28e-17

```
                                                     10        20        30        40        50        60        70
                                             ....*....|....*....|....*....|....*....|....*....|....*....|....*....|..
seqsig_b9437b631427545e290a6399da8d5075  159 ERVLPFWFDHIAPdiLANKKVMVAAHGNSLRGLVKHLDNLSEADVLELNIPTGVPLVYELDENLKPIKHYYL 230
Cdd:cd07067                               84 ARVLPALEELIAP--HDGKNVLIVSHGGVLRALLAYLLGLSDEDILRLNLPNGSISVLELDENGGVLLLRL 153
```

PF3D7_1430300 (HP, LBR_ICE, ATP_GRASP_4, GH47)

Cd Length: 242 **Bit Score:** 72.41 **E-value:** 1.05e-13

```
                                                 10        20        30        40        50        60        70
                                             ....*....|....*....|....*....|....*....|....*....|....*....|....*....|...
seqsig_edd89d7bb35e74e793feba71cc143ebe  884 GELTRMGRRQSENLGKRFRAtLYPGdsdgLLRLHSTFRHDFKIFTSDEGRCQITSAAFTKGFLDLDGELTPIL 956
Cdd:cd07061                               17 GELTPFGRQQAFELGRYFRQ-RYGE----LLLLHSYNRSDLYIRSSDSQRTLQSAQAFLAGLFPPDGWQPIAV 84
```

Cd Length: 114 **Bit Score:** 45.85 **E-value:** 1.76e-05

```
                                                 10        20        30        40        50        60        70        80
                                             ....*....|....*....|....*....|....*....|....*....|....*....|....*....|....*....|
seqsig_edd89d7bb35e74e793feba71cc143ebe 2461 NHIDNNDNNNENNNNNNNNENNNNNISNNNNNISNNNN-----NNISNNNNNSSNNNNNCSNNNNSSSNNNNSSSNNKN 2535
Cdd:cd12796                               18 NVIAGGQNLVVSGSNNLVSGGNHNVLSGNNNTVSGNNHvvtgsNNVVTGNGNTVSGSNNVVSGSNNTVSGGNNTVSGSNN 97
                                                 90
                                             ....*....|....*
seqsig_edd89d7bb35e74e793feba71cc143ebe 2536 NNLNSNLNSNNNNNI 2550
Cdd:cd12796                               98 TVSGSNHIVSGNNKV 112
```

Cd Length: 190 **Bit Score:** 47.07 **E-value:** 1.11e-05

```
                                                 10        20        30        40        50        60        70        80
                                             ....*....|....*....|....*....|....*....|....*....|....*....|....*....|....*....|
seqsig_edd89d7bb35e74e793feba71cc143ebe  195 KNTGGGCKKLFRKIKDRSSEYCPEIHKVRTNGTYIYEEFLSTFGT-DIKVYTVGQMFAHAEARKSPALDgkvCRTSDGKE 273
Cdd:pfam08443                             45 KSVFGSQGIGVFLAEDEQSLEQLLEAFKWLKNQILVQEFIAEAGNrDIRCLVVGGEVVGAIHRQSNEGD---FRTNLHRG 121
                                                 90       100       110       120
                                             ....*....|....*....|....*....|....*....|....*
seqsig_edd89d7bb35e74e793feba71cc143ebe  274 VRYAVI-LSEAEKIIAYRIVEAFQQTVCGFDILRTTMGPFVCDVN 317
Cdd:pfam08443                            122 GVAEPYqLSQEEEEIAIKAAQAMGLDVLGVDIFRSKRGLLVCEVN 166
```

Cd Length: 522 **Bit Score:** 41.63 **E-value:** 2.40e-03

```
                                                 10        20        30        40        50        60
                                             ....*....|....*....|....*....|....*....|....*....|....*....|....*....
seqsig_edd89d7bb35e74e793feba71cc143ebe 1559 NINDNNINDNNINDNNINDNNNNNNNNNNNNNYCNNNYNNNSTS-------TFLDTNIGESYNELKNYK 1620
Cdd:PTZ00470                               3 NLREHLSVHNNADDNYNNNNNNNNQINSNNPNNNGNNQASKLPRgkkkqenPFNKIDEVYYQNEKLNIK 71
```

EEP_Superfamily

PF3D7_0107200

Cd Length: 329 **Bit Score:** 134.35 **E-value:** 2.49e-36

```
                                                10        20        30        40        50        60        70        80
                                            ....*....|....*....|....*....|....*....|....*....|....*....|....*....|....*....|
seqsig_90f36da8af3c13ef41a63d61c7f27c37  39 RKNLLYKHFFYNKLDIICLQEVDS-----FMINDLKKKFFDHDFilhtpehvntkSPKSNN------------CCIAYK 100
Cdd:cd09097                              30 RKQNILKEILSYNADILCLQEVETdqyedFFLPELKQHGYDGVF----------KPKSRAktmseaerkhvdgCAIFFK 98
                                                90       100       110       120       130       140       150       160
                                            ....*....|....*....|....*....|....*....|....*....|....*....|....*....|....*....|
seqsig_90f36da8af3c13ef41a63d61c7f27c37 101 KC-FKLIGEKQFDTETSVIKNlikysseSGCEIQDAFLRELSKRKSIANMVILELSKQTYLG-------ICNCHIHWNPL 172
Cdd:cd09097                              99 TSkFKLVEKHLIEFNQLAMAN-------ADAEGSEDMLNRVMTKDNIALIVVLEARETSYEGnkgqlliVANTHIHWDPE 171
                                               170       180       190       200       210       220       230       240
                                            ....*....|....*....|....*....|....*....|....*....|....*....|....*....|....*....|
seqsig_90f36da8af3c13ef41a63d61c7f27c37 173 YPDIKLYHAYLIIRDFY----EFIQNTVQDIPFIPLLLIGDFNSTPhidhkkedeiiqASGVYELITTGRISKNHahhpa 248
Cdd:cd09097                             172 FSDVKLVQTMMLLEELEkiaeKFSRYPYEDSADIPLVVCGDFNSLP-----------DSGVYELLSNGSVSPNH----- 234
                                               250       260       270       280       290       300       310       320
                                            ....*....|....*....|....*....|....*....|....*....|....*....|....*....|....*....|
seqsig_90f36da8af3c13ef41a63d61c7f27c37 249 rlrkseafysyPELKIPPFKSVFKEI-------------NGKEPEFTNKTPSFEGCIDYIFY--KELIPISTETIPRN-- 311
Cdd:cd09097                             235 -----------PDFKEDPYGEYLTASglthsfklksayaNLGELPFTNYTPDFKGVIDYIFYsaDTLSVLGLLGPPDEdw 303
                                               330       340
                                            ....*....|....*....|....
seqsig_90f36da8af3c13ef41a63d61c7f27c37 312 -LKDIKMLPNEHFPSDHIMLTSEF 334
Cdd:cd09097                             304 yLNKVVGLPNPHFPSDHIALLAEF 327
```

PF3D7_0305600

Cd Length: 253 **Bit Score:** 220.12 **E-value:** 9.34e-67

```
                                                10        20        30        40        50        60        70        80
                                            ....*....|....*....|....*....|....*....|....*....|....*....|....*....|....*....|
seqsig_2c333dcfa1387e1ba2220a205645cf66 280 IVTWNMNSITVRYKNkkkwdEFMNFFNNLNADVLCFQEVRLPAmnlsepcdnknknkndgirdrGKIKNsdqkslady 359
Cdd:cd09087                               3 IISWNVNGLRALLKK-----GLLDYVKKEDPDILCLQETKLQE----------------------GDVPK--------- 45
                                                90       100       110       120       130       140       150       160
                                            ....*....|....*....|....*....|....*....|....*....|....*....|....*....|....*....|
seqsig_2c333dcfa1387e1ba2220a205645cf66 360 eimeqILNDDFKDYNAYFSLANIK-YSGQLVLVKKniHIESIRYNLffenNAHIHHDEGRVILVEFSNFFLLSTYTPNNG 438
Cdd:cd09087                              46 -----ELKELLKGYHQYWNAAEKKgYSGTAILSKK--KPLSVTYGI----GIEEHDQEGRVITAEFENFYLVNTYVPNSG 114
                                               170       180       190       200       210       220       230       240
                                            ....*....|....*....|....*....|....*....|....*....|....*....|....*....|....*....|
seqsig_2c333dcfa1387e1ba2220a205645cf66 439 FDHVKFERRRLFDEQLQKFVTILrnEKQKPLVWTGDLNIAPEDIDLSHpaefrrmkkgnvPKEFIGQPGCTDFERKNFQK 518
Cdd:cd09087                             115 RGLERLDRRKEWDVDFRAYLKKL--DSKKPVIWCGDLNVAHEEIDLAN------------PKTNKKSAGFTPEERESFTE 180
                                               250       260       270       280       290       300       310       320
                                            ....*....|....*....|....*....|....*....|....*....|....*....|....*....|....*....|
seqsig_2c333dcfa1387e1ba2220a205645cf66 519 ILTAGnLVDSYRYlqnikLNEDKKnnikhtpnindNIYTWRCPFLLGKSCNKAMRIDHFIVSKEFLNRINKIHIqgfsvf 598
Cdd:cd09087                             181 LLEAG-FVDTFRH-----LHPDKE-----------GAYTFWSYRGNARAKNVGWRLDYFLVSERLKDRVVDSFI------ 237
                                               330
                                            ....*....|....*.
seqsig_2c333dcfa1387e1ba2220a205645cf66 599 HNNFYGSDHCPVILYL 614
Cdd:cd09087                             238 RSDIMGSDHCPIGLEL 253
```

PF3D7_0319200

Cd Length: 329 **Bit Score:** 105.46 **E-value:** 1.44e-24

```
                                                10        20        30        40        50        60        70        80
```

```
                                            ....*....|....*....|....*....|....*....|....*....|....*....|....*....|....*....|
seqsig_bd37e6a51b0924a19194464134b3efbf 562 ILSYNILApiytnTKYALEHMFKNIDPCYLKTNYRSHLLIHDI-NNNYDIISLQEV-SEYLHSHLFCTFLNENYFSNYKP 639
Cdd:cd09097                               1 VMCYNVLC-----DKYATRQQYGYCPSWALNWDYRKQNILKEIlSYNADILCLQEVeTDQYEDFFLPELKQHGYDGVFKP 75
                                                     90       100       110       120       130       140       150       160
                                            ....*....|....*....|....*....|....*....|....*....|....*....|....*....|....*....|
seqsig_bd37e6a51b0924a19194464134b3efbf 640 KNNFGN---------DGCSLFVNKKKFTLIEYKNYEFNQVIKTDEFKSIYETFLNLSKELDDI----IKEIKTVFQIGiy 706
Cdd:cd09097                              76 KSRAKTmseaerkhvDGCAIFFKTSKFKLVEKHLIEFNQLAMANADAEGSEDMLNRVMTKDNIalivVLEARETSYEG-- 153
                                                    170       180       190       200       210       220       230       240
                                            ....*....|....*....|....*....|....*....|....*....|....*....|....*....|....*....|
seqsig_bd37e6a51b0924a19194464134b3efbf 707 mhkNTNSIFLISNTHFYFHSLAQHIRALQSYTILHILQTLKKKYQLTYPE--KEVYIILNGDFNTNFDSEVFHFIQGKDI 784
Cdd:cd09097                             154 ---NKGQLLIVANTHIHWDPEFSDVKLVQTMMLLEELEKIAEKFSRYPYEdsADIPLVVCGDFNSLPDSGVYELLSNGSV 230
                                                    250       260       270       280       290       300       310       320
                                            ....*....|....*....|....*....|....*....|....*....|....*....|....*....|....*....|
seqsig_bd37e6a51b0924a19194464134b3efbf 785 SPtsdiwfNAQLFKKefDDLDKYPnlfeleknknnneqsiigPHLDRKKFMPLYSAYTK-QDIQFTNWNNNFIDVLDYIF 863
Cdd:cd09097                             231 SP------NHPDFKE--DPYGEYL------------------TASGLTHSFKLKSAYANlGELPFTNYTPDFKGVIDYIF 284
                                                    330       340       350       360
                                            ....*....|....*....|....*....|....*....|....*
seqsig_bd37e6a51b0924a19194464134b3efbf 864 LSTN-IKVRKVLKGIDKECFQ-KYKGALSPINPSDHISIAAEVEL 906
Cdd:cd09097                             285 YSADtLSVLGLLGPPDEDWYLnKVVGLPNPHFPSDHIALLAEFRI 329
```

PF3D7_0705500 (Syja_N, EEP, EEP)

Cd Length: 299 **Bit Score:** 177.80 **E-value:** 2.46e-48

```
                                                      10        20        30        40        50        60        70        80
                                              ....*....|....*....|....*....|....*....|....*....|....*....|....*....|....*....|
seqsig_b411119b5302348402f69874072210e9   692 NEDVRNDEKKNNSKMDIHKYLKTIQKLL-SVHMYYSYDYDLTQCIQKKVKNNIEEveveplvyntrlnnlnnkkkKSFLK 770
Cdd:pfam02383                              45 NSSEYDSESDKKSSKDEEHYLKLLKKLLsSGSFYFSYDYDLTNSLQRRGLRESSS-------------------LSLWK 104
                                                      90       100       110       120       130       140       150       160
                                              ....*....|....*....|....*....|....*....|....*....|....*....|....*....|....*....|
seqsig_b411119b5302348402f69874072210e9   771 MCEKKYVWNYQMIKKTKNKCKIDDNWFCSIIQGYISYTSIEINKKCLELLLISRRSSILGGTRFNKRGINDDGYVANYVE 850
Cdd:pfam02383                             105 RADDRFVWNSYLLKPLIDFRSDLSDWLLPLIQGFVEQRTISVNGKKVTLTLISRRSRKRAGTRYNRRGIDDDGNVANFVE 184
                                                     170       180       190       200       210       220       230       240
                                              ....*....|....*....|....*....|....*....|....*....|....*....|....*....|....*....|
seqsig_b411119b5302348402f69874072210e9   851 SEQIVRINnrninhvdkvdqlnnkdntsmsdinydsyknknftstkgnldknnsnrnntynnsnvsneilaspnlthdqv 930
Cdd:pfam02383                             185 TEQIVSTN------------------------------------------------------------------------ 192
                                                     250       260       270       280       290       300       310       320
                                              ....*....|....*....|....*....|....*....|....*....|....*....|....*....|....*....|
seqsig_b411119b5302348402f69874072210e9   931 nhlkensvrekdknnnsfgssqvvggnistetkeqfvnkdkinqgvfgtykmndsydnnnnnnnnnnntntntnnnnnn 1010
Cdd:pfam02383                                 --------------------------------------------------------------------------------
                                                     330       340       350       360       370       380       390       400
                                              ....*....|....*....|....*....|....*....|....*....|....*....|....*....|....*....|
seqsig_b411119b5302348402f69874072210e9  1011 nnnnnnnnnssssstnkyvgrnfENRIISLVQIRGSIPLFWKQ---HSMSSHVNIQRSSLLSIKAFKEHNKKLINSYGnNI 1087
Cdd:pfam02383                             193 ----------------------SGRIFSFVQIRGSVPLFWEQdpnLLYKPKIKITRSSEATQPAFDKHFDDLIKRYG-PI 249
                                                     410       420       430       440       450
                                              ....*....|....*....|....*....|....*....|....*....|....*..
seqsig_b411119b5302348402f69874072210e9  1088 YYINLLSQnKSNEKKLTKKMIEMINFIKKDKHykekdyINYIEYDFHISVKNKSFED 1144
Cdd:pfam02383                             250 YIVNLLDK-KGSEKKLSEAYEEAINYLNENKK------IKYTWFDFHAECKGMKFDN 299
```

Cd Length: 328 **Bit Score:** 170.65 **E-value:** 1.16e-45

```
                                                      10        20        30        40        50        60        70        80
                                              ....*....|....*....|....*....|....*....|....*....|....*....|....*....|....*....|
seqsig_b411119b5302348402f69874072210e9  2429 RKFKYVKLKSVSMIGLFIIIFIDEALVDHIREIEVCKVKVGLKGNTGNKGSVSVKFRLGYNSFCFNNIHLASGQTNIFER 2508
Cdd:cd09089                                90 RDHKYVLLTSEQLVGVCLFVFVRPQHAPFIRDVAVDTVKTGLGGAAGNKGAVAIRFLLHSTSLCFVCSHFAAGQSQVKER 169
                                                      90       100       110       120       130       140       150       160
                                              ....*....|....*....|....*....|....*....|....*....|....*....|....*....|....*....|
seqsig_b411119b5302348402f69874072210e9  2509 NTQMQNILSN-SFQNQQlnNLFNFDYFFACGDFNFRINKNLEEVLKLISSKNIKHLLNYDQFIYNK-MYNIlpFCLFHEH 2586
Cdd:cd09089                               170 NEDFAEIARKlSFPMGR--TLDSHDYVFWCGDFNYRIDLPNDEVKELVRNGDWLKLLEFDQLTKQKaAGNV--FKGFLEG 245
                                                     170       180       190       200       210       220       230       240
                                              ....*....|....*....|....*....|....*....|....*....|....*....|....*....|....*....|
seqsig_b411119b5302348402f69874072210e9  2587 PITFNPTYKYKKHSNMYDIR---RTPAWCDRVLMsgklvhlseiEKKRNEHISREHYRERNISiDQTNKSDMVDYKRNNM 2663
Cdd:cd09089                               246 EINFAPTYKYDLFSDDYDTSekcRTPAWTDRVLW----------RRRKWPSDKTEESLVETND-PTWNPGTLLYYGRAEL 314
                                                     250       260       270       280
                                              ....*....|....*....|....*....|....*....|.
seqsig_b411119b5302348402f69874072210e9  2664 sdkdilndfynndkiyfkyldyKThnnffsSDHKPVSALIE 2704
Cdd:cd09089                               315 ----------------------KT------SDHRPVVAIID 327
```

Cd Length: 291 **Bit Score:** 48.49 **E-value:** 1.31e-05

```
                                                      10        20        30        40        50        60        70        80
                                              ....*....|....*....|....*....|....*....|....*....|....*....|....*....|....*....|
seqsig_b411119b5302348402f69874072210e9  1898 IKLWAGTWNLCGGDLEElhDISSWL--NEVDDYIDMYVFCFQEVVELTGFRILMNmkDKFKEKKIEQMITQTLgevSQRQ 1975
Cdd:cd09090                                 1 INIFVGTFNVNGKSYKD--DLSSWLfpEENDELPDIVVIGLQEVVELTAGQILNS--DPSKSSFWEKKIKTTL---NGRG 73

                                              ....*.
seqsig_b411119b5302348402f69874072210e9  1976 KELYLR 1981
Cdd:cd09090                                74 GEKYVL 79
```

PF3D7_1111600

Cd Length: 299 **Bit Score:** 99.71 **E-value:** 5.50e-23

```
                                                     10        20        30        40        50        60        70        80
                                            ....*....|....*....|....*....|....*....|....*....|....*....|....*....|....*....|
seqsig_e70ab9377079b25c68bf8b910bf3acdf 420 IFSHWIQPYY----DIYVICLQE--------------SISDNIINCLSMYLKEINQetylflpladYKLSGYGdgaflQM 481
Cdd:cd09074                              19 NLENWLSPKGteapDIYAVGVQEvdmsvqgfvgnddsAKAREWVDNIQEALNEKEN----------YVLLGSA-----QL 83
                                                     90       100       110       120       130       140       150       160
                                            ....*....|....*....|....*....|....*....|....*....|....*....|....*....|....*....|
seqsig_e70ab9377079b25c68bf8b910bf3acdf 482 KSTTIAAWVRKSKLhPR---GPVKLCASKSIAFNKLNNsKGCVSILLNIFNQFVLFIGCHMPAKDQEIRQKSREF--ILT 556
Cdd:cd09074                              84 VGIFLFVFVKKEHL-PQikdLEVEGVTVGTGGGGKLGN-KGGVAIRFQINDTSFCFVNSHLAAGQEEVERRNQDYrdILS 161
                                                    170       180       190       200       210       220       230       240
                                            ....*....|....*....|....*....|....*....|....*....|....*....|....*....|....*....|
seqsig_e70ab9377079b25c68bf8b910bf3acdf 557 KLSEYfSNKSTSNFKDVFHHVIWMGDFNFRVHGiHIEKVIHYLKTDNLKELLKHDEGHSAYSYD-LSISFQELPINFLPT 635
Cdd:cd09074                             162 KLKFY-RGDPAIDSIFDHDVVFWFGDLNYRIDS-TDDEVRKLISQGDLDDLLEKDQLKKQKEKGkVFDGFQELPITFPPT 239
                                                    250       260       270       280       290       300       310       320
                                            ....*....|....*....|....*....|....*....|....*....|....*....|....*....|....*....|
seqsig_e70ab9377079b25c68bf8b910bf3acdf 636 YKkngnrpiINRSDTEWVQKEyklihnikwykggkqETRIPSWTDRIFKWSCEKTknclVFVPNSYLSpIPEeksiIMSS 715
Cdd:cd09074                             240 YK-------FDPGTDEYDTSD---------------KKRIPAWCDRILYKSKAGS----EIQPLSYTS-VPL----YKTS 288
```

```
                                             ....*....
seqsig_e70ab9377079b25c68bf8b910bf3acdf 716 DHNPVSCCF 724
Cdd:cd09074                             289 DHKPVRATF 297
```

PF3D7_1238600 (EEP, EEP) SPHINGOMYELIN

Cd Length: 280 **Bit Score:** 101.26 **E-value:** 1.13e-24

```
                                                      10        20        30        40        50        60        70        80
                                                ....*....|....*....|....*....|....*....|....*....|....*....|....*....|....*....|
seqsig_3203ca97a7ab7b6d07d180340dabb34c 176 FHFVNGGVIVLSKHKILHKHALIYSSGKFPDVFCSKGAIYLKCDVMNKKV-NVVATHLQAGD-NKEQQKCRWKQIDELSK 253
Cdd:cd09078                              78 SKLVDGGVVILSRYPIVEKDQYIFPNGCGADCLAAKGVLYAKINKGGTKVyHVFGTHLQASDgSCLDRAVRQKQLDELRA 157
                                                      90       100       110       120       130       140       150       160
                                                ....*....|....*....|....*....|....*....|....*....|....*....|....*....|....*....|
seqsig_3203ca97a7ab7b6d07d180340dabb34c 254 WVYEGIpstfIKKFESLFFVGDFNIRYNADRLFLDKVLsdNYLNSY-----VTKKSLDTTYDSFLNDYCRYIERDyNYKH 328
Cdd:cd09078                             158 FIEEKN----IPDNEPVIIAGDFNVDKRSSRDEYDDML--EQLHDYnapepITAGETPLTWDPGTNLLAKYNYPG-GGGE 230
                                                     170       180       190       200       210       220
                                                ....*....|....*....|....*....|....*....|....*....|....*..
seqsig_3203ca97a7ab7b6d07d180340dabb34c 329 KytLDYILVAN------NSNVEIIVPQTSIQNYYKSLYFIKFflgiipykttyihhpSDHFPIYATF 389
Cdd:cd09078                             231 R--LDYILYSNdhlqpsSWSNEVEVPKSPTWSVTNGYTFADL---------------SDHYPVSATF 280
```

Cd Length: 280 **Bit Score:** 52.34 **E-value:** 4.97e-08

```
                                                      10        20        30        40        50        60        70
                                                ....*....|....*....|....*....|....*....|....*....|....*....|....*....|....*....
seqsig_3203ca97a7ab7b6d07d180340dabb34c  12 LTIMSYNIQMLSIPFSVHLNSW--TRRNAIIDYicslddIYDIDILVLNEVFTKKCYKLLTSGkIKEKFPYHTNVIGRN 88
Cdd:cd09078                               1 LKVLTYNVFLLPPLLYNNGDDGqdERLDLIPKA------LLQYDVVVLQEVFDARARKRLLNG-LKKEYPYQTDVVGRS 72
```

PF3D7_1430600 (AP, AP)

Cd Length: 309 **Bit Score:** 100.47 **E-value:** 4.61e-23

```
                                                      10        20        30        40        50        60        70        80
                                                ....*....|....*....|....*....|....*....|....*....|....*....|....*....|....*....|
seqsig_54ebe5c5b0ecae34017419b1778c7528   8 SWNVNGWK--KSCEIIKRNDDdLVQFLKKLDIDILCLQETKTNESVIENDCNLLEAdsnmYESYWTCCKKKkgdkthKGY 85
Cdd:cd09088                               4 TWNVNGIRtrLQYQPWNKENS-LKSFLDSLDADIICLQETKLTRDELDEPSAIVEG----YDSFFSFSRGR------KGY 72
                                                      90       100       110       120       130       140       150       160
                                                ....*....|....*....|....*....|....*....|....*....|....*....|....*....|....*....|
seqsig_54ebe5c5b0ecae34017419b1778c7528  86 SGLATYVKnenkiiCSTNNVfddfsfffndyIKKEDLLikkkSEIDKTSISFFLLNDNKKIYNDQNIKCDKNDEhnkkkn 165
Cdd:cd09088                              73 SGVATYCR------DSAATP-----------VAAEEGL----TGVLSSPNQKNELSENDDIGCYGEMLEFTDSK------ 125
                                                     170       180       190       200       210       220       230       240
                                                ....*....|....*....|....*....|....*....|....*....|....*....|....*....|....*....|
seqsig_54ebe5c5b0ecae34017419b1778c7528 166 ktniSVSEFFNEGRILITMHKHFIIVNIYAPY-SGHNYERLYYKVRFFHAVRAKIIQLrIVTGLPIILLGDFNISYRNKD 244
Cdd:cd09088                             126 ----ELLELDSEGRCVLTDHGTFVLINVYCPRaDPEKEERLEFKLDFYRLLEERVEAL-LKAGRRVILVGDVNVSHRPID 200
                                                     250
                                                ....*....|....*...
seqsig_54ebe5c5b0ecae34017419b1778c7528 245 IYYLNNIINLDILLKNIH 262
Cdd:cd09088                             201 HCDPDDSEDFGGESFEDN 218
```

Cd Length: 309 **Bit Score:** 56.56 **E-value:** 1.03e-08

```
                                                      10        20        30        40
                                                ....*....|....*....|....*....|....*....|..
seqsig_54ebe5c5b0ecae34017419b1778c7528 606 DNMIDTFSFFHPNVNGKFTCWDTYRQCRVHNEGSRIDYIFMD 647
Cdd:cd09088                             240 GLLIDSFRYFHPTRKGAYTCWNTLTGARPTNYGTRIDYILAD 281
```

PF3D7_1363500 (DEADENYLASE)

Cd Length: 329 **Bit Score:** 65.40 **E-value:** 1.34e-11

```
                                                      10        20        30        40        50        60        70        80
                                                ....*....|....*....|....*....|....*....|....*....|....*....|....*....|....*....|
seqsig_83f7859c0d0069614112e9852c948039 244 VFSFNILANslvdyKY---DNNGYN---IMQWMNRKKWIHQNIMNKLSDIICLQEIE-ESYFIELKNELEKLHFKGLFLK 316
Cdd:cd09097                               1 VMCYNVLCD-----KYatrQQYGYCpswALNWDYRKQNILKEILSYNADILCLQEVEtDQYEDFFLPELKQHGYDGVFKP 75
                                                      90       100       110       120       130       140       150       160
                                                ....*....|....*....|....*....|....*....|....*....|....*....|....*....|....*....|
seqsig_83f7859c0d0069614112e9852c948039 317 K--------KKDTCKDGICIFYNTKVFELLFFDEVIYDKSCLlkkwhvgliiaLRNIISKRIDHFEEFhennMNKKNYKN 388
Cdd:cd09097                              76 KsraktmseAERKHVDGCAIFFKTSKFKLVEKHLIEFNQLAM-----------ANADAEGSEDMLNRV----MTKDNIAL 140
                                                     170       180       190       200       210       220       230       240
                                                ....*....|....*....|....*....|....*....|....*....|....*....|....*....|....*....|
seqsig_83f7859c0d0069614112e9852c948039 389 ECdrILQNSNTHMNNNKNNnnnnnknncnifnmcddIVIVSNTHLIFDSYKGDVKLYQIcymtyrlILMMKKCINYIKKR 468
Cdd:cd09097                             141 IV--VLEARETSYEGNKGQ-----------------LLIVANTHIHWDPEFSDVKLVQT-------MMLLEELEKIAEKF 194
                                                     250       260       270       280       290       300
                                                ....*....|....*....|....*....|....*....|....*....|....*...
seqsig_83f7859c0d0069614112e9852c948039 469 KKGNDNTNSDTSssscynykndlslcpkddtflkpcIIVCGDFNITPNSLLYYFIVNRFINLKHINLK 536
Cdd:cd09097                             195 SRYPYEDSADIP------------------------LVVCGDFNSLPDSGVYELLSNGSVSPNHPDFK 238
```

Cd Length: 329 **Bit Score:** 39.59 **E-value:** 2.45e-03

```
                                                      10        20        30        40        50        60
                                                ....*....|....*....|....*....|....*....|....*....|....*....|.
seqsig_83f7859c0d0069614112e9852c948039 769 QIPFTVFHGKQKGCVDYIFYSYKNLKQVSYTNLPTFDK-LSKYGCLPNQKYaSSDHLYLHA 828
Cdd:cd09097                             266 ELPFTNYTPDFKGVIDYIFYSADTLSVLGLLGPPDEDWyLNKVVGLPNPHF-PSDHIALLA 325
```

HAD Superfamily

PF3D7_0303200 (HAD, LIPIN_N, LNS2)

Cd Length: 139 **Bit Score:** 40.43 **E-value:** 4.75e-04

```
                                                       10        20        30        40        50        60        70        80
                                                 ....*....|....*....|....*....|....*....|....*....|....*....|....*....|....*....|
seqsig_303ed259024b135e13c073d1d2a60b61  947 IVISDVDGTITRStvlGHIMPIVGKDWSHVGVSQLFNKINKNGYHILYLTARaigqadSTREYLFRLKKNDNNKLPD--- 1023
Cdd:cd01427                                1 AVLFDLDGTLLDS---EPGIAEIEELELYPGVKEALKELKEKGIKLALATNK------SRREVLELLEELGLDDYFDpvi 71
                                                       90       100       110       120
```

```
                                                ....*....|....*....|....*....|....*....|..
seqsig_303ed259024b135e13c073d1d2a60b61 1024 ---GPLILSPDRLFPSFKREVIDKKPYIFKIAALRDIRNLFP 1062
Cdd:cd01427                               72 tsnGAAIYYPKEGLFLGGGPFDIGKPNPDKLLAALKLLGVDP 113
```

Cd Length: 111 **Bit Score:** 111.30 **E-value:** 3.89e-28

```
                                                        10        20        30        40        50        60        70        80
                                                ....*....|....*....|....*....|....*....|....*....|....*....|....*....|....*....|
seqsig_303ed259024b135e13c073d1d2a60b61    6 VSSVSNalDFNQATLSGCIDIICIESEIENklkndkieviYKSTPFHVRFGKTKLLRSKEKIVSILVNGKSTNLHMKLGS 85
Cdd:pfam04571                             12 VKELYN--PINPATLSGAIDVIVVEQPDGS----------LRCSPFHVRFGKFGVLKSSEKVVDIEVNGVEVDFHMKLGD 79
                                                        90       100
                                                ....*....|....*....|....*....
seqsig_303ed259024b135e13c073d1d2a60b61   86 AGEAYFVEKTYEDVEE---DLETSPLSSP 111
Cdd:pfam04571                             80 SGEAFFVEETEDDVEDvpdYLATSPIPNG 108
```

Cd Length: 156 **Bit Score:** 257.68 **E-value:** 1.51e-79

```
                                                        10        20        30        40        50        60        70        80
                                                ....*....|....*....|....*....|....*....|....*....|....*....|....*....|....*....|
seqsig_303ed259024b135e13c073d1d2a60b61  947 IVISDVDGTITRSTVLGHIMPIVGKDWSHVGVSQLFNKINKNGYHILYLTARAIGQADSTREYLFRLKKNDNNkLPDGPL 1026
Cdd:pfam08235                              1 VVISDIDGTITASDALGHILSIMGKDWVHAGVVDLVRKIQENGYLIIYLTARPDGQADRTRAYLSQHKQDGYN-LPHGPV 79
                                                        90       100       110       120       130       140       150
                                                ....*....|....*....|....*....|....*....|....*....|....*....|....*....|....*...
seqsig_303ed259024b135e13c073d1d2a60b61 1027 ILSPDRLFPSFKREVIDKKPYIFKIAALRDIRNLFPlNHNPFYAAFGNTESDHRAYISVGVPEAKVFIIDNRGIVHHV 1104
Cdd:pfam08235                             80 ILSPDRLFAALHREVILKKPLVFKIACLRDIRQLFP-NRNPFYAGFGNRIKDVIVYRSVGVPPSRIFTVNPKGEVHQE 156
```

PF3D7_0515900 (HIF_SF, FCP1)

Cd Length: 162 **Bit Score:** 222.55 **E-value:** 6.45e-72

```
                                                      10        20        30        40        50        60        70        80
                                              ....*....|....*....|....*....|....*....|....*....|....*....|....*....|....*....|
seqsig_fc2183a48fdf37324927f2f6b65e5691  57 TMTLVLDLDETLIYCTKKRKY-HYQKEVDVLINGKYLPLYVCKRPYIDLFFSSLYPFYEIIIFTTAIKSYADTVLNIID- 134
Cdd:TIGR02251                             1 KKTLVLDLDETLVHSTFKMPKvDADFKVPVLIDGKIIPVYVFKRPHVDEFLERVSKWYELVIFTASLEEYADPVLDILDr 80
                                                      90       100       110       120       130       140       150       160
                                              ....*....|....*....|....*....|....*....|....*....|....*....|....*....|....*....|
seqsig_fc2183a48fdf37324927f2f6b65e5691 135 VDHYIDKKFYREDCYEMNEKlYIKNLTNIKKELSKIILIDDSNISGFQYPDNFFPIKKWQGDLNDNELLHLIPFFLNLRK 214
Cdd:TIGR02251                            81 GGKVISRRLYRESCVFTNGK-YVKDLSLVGKDLSKVIIIDNSPYSYSLQPDNAIPIKSWFGDPNDTELLNLIPFLEGLRF 159

                                              ...
seqsig_fc2183a48fdf37324927f2f6b65e5691 215 LKD 217
Cdd:TIGR02251                           160 EDD 162
```

Cd Length: 156 **Bit Score:** 46.12 **E-value:** 1.38e-06

```
                                                      10        20        30        40        50        60        70        80
                                              ....*....|....*....|....*....|....*....|....*....|....*....|....*....|....*....|
seqsig_fc2183a48fdf37324927f2f6b65e5691  58 MTLVLDLDETLIYCTKKRKYHYQKEVD------------VLINGKYLPLYVCKRPYIDLFFSSLYPFYEIIIFTTAIKSY 125
Cdd:TIGR02250                             7 LHLVLDLDQTLIHTTKDPTLSEWEKYDieepnsetrrdlRKFNLGTMWYLTKLRPFLHEFLKEASKLYEMHVYTMGTRAY 86
                                                      90       100       110       120       130       140       150
                                              ....*....|....*....|....*....|....*....|....*....|....*....|....*....|.
seqsig_fc2183a48fdf37324927f2f6b65e5691 126 ADTVLNIIDVDHYI--DKKFYREDCYEmnekLYIKNLTNI-KKELSKIILIDDSNISGFQYPDNFFPIKKW 193
Cdd:TIGR02250                            87 AQAIAKLIDPDGKYfgDRIISRDESGS----PHTKSLLRLfPADESMVVIIDDREDVWPWHKRNLIQIEPY 153
```

PF3D7_0715000 (HAD, HAD, PLN02645)

Cd Length: 139 **Bit Score:** 57.77 **E-value:** 1.11e-10

```
                                                      10        20        30        40        50        60        70        80
                                              ....*....|....*....|....*....|....*....|....*....|....*....|....*....|....*....|
seqsig_c3f569364d79abb839725ed0058b46c2  45 VFFFDCDGVLWHGN---------ELIEGSIEVINYLLREGKKVYFITNnstKSRASFLEKFHKLGFtNVKREHIICTAYA 115
Cdd:cd01427                               1 AVLFDLDGTLLDSEpgiaeieelELYPGVKEALKELKEKGIKLALATN---KSRREVLELLEELGL-DDYFDPVITSNGA 76
                                                      90       100
                                              ....*....|....*....|..
seqsig_c3f569364d79abb839725ed0058b46c2 116 VTkylYDKEEYRLRKKKIYVIG 137
Cdd:cd01427                              77 AI---YYPKEGLFLGGGPFDIG 95
```

Cd Length: 139 **Bit Score:** 54.68 **E-value:** 1.64e-09

```
                                                      10        20        30        40        50        60        70        80
                                              ....*....|....*....|....*....|....*....|....*....|....*....|....*....|....*....|
seqsig_c3f569364d79abb839725ed0058b46c2 207 FIATNKDATG---NFTSKQKWAGTGAIVSSIEA---------VSLKKPIVVGKPNVYMIENVLKDLNIHHSKVVMIGDRL 274
Cdd:cd01427                              44 ALATNKSRREvleLLEELGLDDYFDPVITSNGAaiyypkeglFLGGGPFDIGKPNPDKLLAALKLLGVDPEEVLMVGDSL 123
                                                      90
                                              ....*....|....*..
seqsig_c3f569364d79abb839725ed0058b46c2 275 eTDIHFAKNCNIKSILV 291
Cdd:cd01427                             124 -NDIEMAKAAGGLGVAV 139
```

Cd Length: 311 **Bit Score:** 302.02 **E-value:** 7.05e-101

```
                                                      10        20        30        40        50        60        70        80
                                              ....*....|....*....|....*....|....*....|....*....|....*....|....*....|....*....|
seqsig_c3f569364d79abb839725ed0058b46c2  35 NSKDLCLEFDVFFFDCDGVLWHGNELIEGSIEVINYLLREGKKVYFITNNSTKSRASFLEKFHKLGFtNVKREHIICTAY 114
Cdd:PLN02645                             20 NADELIDSVETFIFDCDGVIWKGDKLIEGVPETLDMLRSMGKKLVFVTNNSTKSRAQYGKKFESLGL-NVTEEEIFSSSF 98
                                                      90       100       110       120       130       140       150       160
                                              ....*....|....*....|....*....|....*....|....*....|....*....|....*....|....*....|
seqsig_c3f569364d79abb839725ed0058b46c2 115 AVTKYLydKEEYRLRKKKIYVIGEKGICDELDASNLDWLGGSNDNDKKIILKDDLEIIVDKNIGAVVVGIDFNINYYKIQ 194
Cdd:PLN02645                             99 AAAAYL--KSINFPKDKKVYVIGEEGILEELELAGFQYLGGPEDGDKKIELKPGFLMEHDKDVGAVVVGFDRYINYYKIQ 176
                                                     170       180       190       200       210       220       230       240
                                              ....*....|....*....|....*....|....*....|....*....|....*....|....*....|....*....|
seqsig_c3f569364d79abb839725ed0058b46c2 195 YAQLCINELN-AEFIATNKDATGNFTSKQKWAGTGAIVSSIEAVSLKKPIVVGKPNVYMIENVLKDLNIHHSKVVMIGDR 273
Cdd:PLN02645                            177 YATLCIRENPgCLFIATNRDAVTHLTDAQEWAGAGSMVGAIKGSTEREPLVVGKPSTFMMDYLANKFGIEKSQICMVGDR 256
                                                     250       260       270       280
                                              ....*....|....*....|....*....|....*....|....*....
seqsig_c3f569364d79abb839725ed0058b46c2 274 LETDIHFAKNCNIKSILVSTGVTNANIyLNHNSLNIHPDYFMKSISELL 322
Cdd:PLN02645                            257 LDTDILFGQNGGCKTLLVLSGVTSESM-LLSPENKIQPDFYTSKISDFL 304
```

PF3D7_0726900 (NIF)

Cd Length: 153 **Bit Score:** 131.58 **E-value:** 3.31e-36

```
                                                  10        20        30        40        50        60        70        80
                                              ....*....|....*....|....*....|....*....|....*....|....*....|....*....|....*....|
seqsig_7b298445317b9151927ce631704eb6a7 322 PTLVIDLNYVI--AKLEYD--------RKTGWRVLKRPYADRFFKELSSFYEIVIWSDDNFPVAQEVISKW---GIPAIG 388
Cdd:pfam03031                             1 KTLVLDLDETLvhSSFEPDlpfdfvlnFNHGVYVKKRPGLDEFLQELSKYYEIVIFTASSKEYADPVLDKLdpkKKYFKH 80
                                                  90       100       110       120       130       140       150
                                              ....*....|....*....|....*....|....*....|....*....|....*....|....*....|.
seqsig_7b298445317b9151927ce631704eb6a7 389 CLHRDQCskkkKSYVKDLKRLGRNLDRVVIIDHDAKAFMLQPENGILIKEFHGDLNDKEMLCLIDLLKSFA 459
Cdd:pfam03031                            81 RLYRESC----TFYVKDLSLLGRDLSRVVIVDNSPRSFLLQPDNGIPIPPFYGDPDDTELLKLLPFLKELA 147
```

PF3D7_0817400 (HAD)

```
                                                  10        20        30        40
                                              ....*....|....*....|....*....|....*....|....*...
seqsig_1f1c29151dc8a7512f3e3c59917f48e6 114 FDFDGTLIN-KHFSNNHKNNIIFDkERIPILNSLKKKKYEIVVFSNQT 160

Cdd:cd01427                               4 FDLDGTLLDsEPGIAEIEELELYP-GVKEALKELKEKGIKLALATNKS 50
```

PF3D7_1012700 (CPDc, GH47)

Cd Length: 148 **Bit Score:** 78.42 **E-value:** 2.29e-16

```
                                                   10        20        30        40        50        60        70        80
                                               ....*....|....*....|....*....|....*....|....*....|....*....|....*....|....*....|
seqsig_9154e3039ce65ddeb4a4a22036346cbc  562 NVKYQKGAYIIYYKLRPGVIEFLRTMSEKYEIYLYTMGTLEHAKSCLFLLDPlRKFFGNRVFSRKDCL----NSLKHLNK 637
Cdd:smart00577                            32 PVLIDGHPHGVYVKKRPGVDEFLKRASELFELVVFTAGLRMYADPVLDLLDP-KKYFGYRRLFRDECVfvkgKYVKDLSL 110
                                                   90       100       110       120
                                               ....*....|....*....|....*....|....*....|.
seqsig_9154e3039ce65ddeb4a4a22036346cbc  638 ILPTYRSVSIcIDDSDYIW---KENSscIKVHGYNYFPDIN 675
Cdd:smart00577                           111 LNRDLSKVII-IDDSPDSWpfhPENL--IPIKPWFGDPDDT 148
```

Cd Length: 522 **Bit Score:** 44.71 **E-value:** 1.41e-04

```
                                                   10        20        30        40
                                               ....*....|....*....|....*....|....*....|....*...
seqsig_9154e3039ce65ddeb4a4a22036346cbc  432 NININVNNNNNNNNNNYNNNNNYNNNNNYNNNNNYNNNNNYNNNSNK 479
Cdd:PTZ00470                               1 NVNLREHLSVHNNADDNYNNNNNNNNQINSNNPNNNGNNQASKLPRGK 48
```

PF3D7_1118400 (HAD, HAD_2)

Cd Length: 139 **Bit Score:** 47.36 **E-value:** 4.02e-07

```
                                                  10        20        30        40        50        60        70        80
                                              ....*....|....*....|....*....|....*....|....*....|....*....|....*....|....*....|
seqsig_d6fc9c1850fa52c1d7d49d98027dca74 134 ISPGTLEYLRELKNRGYILGAITNGDSD--------VNEIKFLNEIFS------------FVVRSMDYNFAKPNVEIFNI 193
Cdd:cd01427                              25 LYPGVKEALKELKEKGIKLALATNKSRRevlelleeLGLDDYFDPVITsngaaiyypkegLFLGGGPFDIGKPNPDKLLA 104
                                                  90       100       110       120
                                              ....*....|....*....|....*....|....*....|.
seqsig_d6fc9c1850fa52c1d7d49d98027dca74 194 AENLLKEKninfhVDEWLHVGDDvYTDIMGAKNKNINCAWI 234
Cdd:cd01427                             105 ALKLLGVD-----PEEVLMVGDS-LNDIEMAKAAGGLGVAV 139
```

Cd Length: 176 **Bit Score:** 68.13 **E-value:** 4.05e-14

```
                                                  10        20        30        40        50        60        70        80
                                              ....*....|....*....|....*....|....*....|....*....|....*....|....*....|....*....|
seqsig_d6fc9c1850fa52c1d7d49d98027dca74  26 ITFDLDHTIWNIDALLnyadnecykymeqnYKRLYDYLSKEYALSMTNlvkELLERNIDMNKDgvqiltrirtDALKYLA 105
Cdd:pfam13419                             1 IIFDLDGTLIDFDPVI--------------FEALRDLAAERLGLDISA---EELREAGGLPFD----------EALADLL 53
                                                  90       100       110       120       130       140       150       160
                                              ....*....|....*....|....*....|....*....|....*....|....*....|....*....|....*....|
seqsig_d6fc9c1850fa52c1d7d49d98027dca74 106 KQTNYDeikfASEIQKLWKE--KKKDVHLFisPGTLEYLRELKNRGYILGAITNGDSDVNEIKF----LNEIFSFVVRSM 179
Cdd:pfam13419                            54 REHPID----PDEILEALLEynLESRLEPF--PDVVELLRRLKAKGVKLVILSNGSREAVERLLeklgLLDLFDAVFTSD 127
                                                 170       180       190       200       210
                                              ....*....|....*....|....*....|....*....|....*....|....*
seqsig_d6fc9c1850fa52c1d7d49d98027dca74 180 DYNFAKPNVEIFNIAENLLkekniNFHVDEWLHVGDDVYtDIMGAKNKNINCAWI 234
Cdd:pfam13419                           128 DVGARKPDPEAYERVLERL-----GLPPEEILFIDDSPE-DLEAARAAGIKTVHV 176
```

PF3D7_1226100 (HAD, Yrb1, Hydrolase)

Cd Length: 139 **Bit Score:** 40.82 **E-value:** 6.95e-05

```
                                                  10        20        30        40        50        60        70        80
                                              ....*....|....*....|....*....|....*....|....*....|....*....|....*....|....*....|
seqsig_02abc89cb7f300b4793a2519d6dcc2a7  22 LIAIDIDGTLADDTG--------KISDENLKAIEVCKKGGIEIILASGRLHSYAMKMFTNEQIEKYKiekldGVYS--HG 91
Cdd:cd01427                               1 AVLFDLDGTLLDSEPgiaeieelELYPGVKEALKELKEKGIKLALATNKSRREVLELLEELGLDDYF----DPVITsnGA 76
                                                  90
                                              ....*....|....*..
seqsig_02abc89cb7f300b4793a2519d6dcc2a7  92 AYIHMKGYDYVYRKFSY 108
Cdd:cd01427                              77 AIYYPKEGLFLGGGPFD 93
```

Cd Length: 154 **Bit Score:** 41.74 **E-value:** 4.36e-05

```
                                                  10        20        30        40        50        60
                                              ....*....|....*....|....*....|....*....|....*....|....*....|
seqsig_02abc89cb7f300b4793a2519d6dcc2a7 248 QFYRINLNNILSIGNDDNDIELLSSTGFSVAVKNSTPRALQVARCVSTKTNNENAVANII 307
Cdd:TIGR01670                            86 EKLALAPENVAYIGDDLIDWPVMEKVGLSVAVADAHPLLIPRADYVTRIAGGRGAVREVC 145
```

Cd Length: 254 **Bit Score:** 152.39 **E-value:** 7.54e-44

```
                                                  10        20        30        40        50        60        70        80
                                              ....*....|....*....|....*....|....*....|....*....|....*....|....*....|....*....|
seqsig_02abc89cb7f300b4793a2519d6dcc2a7  23 IAIDIDGTLADDTGKISDENLKAIEVCKKGGIEIILASGRLHSYAMKMFTNEQIEKYKIekldgvYSHGAYIHM-KGYDY 101
```

```
Cdd:pfam08282                             1 IASDLDGTLLNSDKKISERTKEAIKKLQEKGIKVVIATGRPYRGALPVLEELGLDLPVI------CYNGAYIYDeNGKIL 74
                                                    90       100       110       120       130       140       150       160
                                            ....*....|....*....|....*....|....*....|....*....|....*....|....*....|....*....|
seqsig_02abc89cb7f300b4793a2519d6dcc2a7 102 VYRKFSYKDLELILFSLGSYNIlrNAVFLTVDSAYVINDDiklieeyiytpeSEGIISDIEYVKIIDTNYKPILINKIKD 181
Cdd:pfam08282                            75 YKNPISKEDVKEIIEYLKENNL--SILLYTDDGAYILNDV------------SEKIVREERYVKSFVLVIDDFELLEDED 140
                                                   170       180       190       200       210       220       230       240
                                            ....*....|....*....|....*....|....*....|....*....|....*....|....*....|....*....|
seqsig_02abc89cb7f300b4793a2519d6dcc2a7 182 IFNIGDIVSIEIYDKLYpnQDIYSDLFKVLfyelqphykIYIPSSNNKIVLSPINTAKVHTTQLYAQFYRINLNNILSIG 261
Cdd:pfam08282                           141 INKILIVTDPEDLDELE--KELKELFGSLI---------TITSSGPGYLEIMPKGVSKGTALKALAKHLGIDLEEVIAFG 209
                                                   250       260       270       280
                                            ....*....|....*....|....*....|....*....|....*.
seqsig_02abc89cb7f300b4793a2519d6dcc2a7 262 NDDNDIELLSSTGFSVAVKNSTPRALQVARCVsTKTNNENAVANII 307
Cdd:pfam08282                           210 DGENDIEMLELAGLGVAMGNASPEVKAAADYV-TGSNNEDGVAKAL 254
```

PF3D7_1355700 (NIF, FCP1)

Cd Length: 153 **Bit Score:** 92.29 **E-value:** 4.71e-21

```
                                                    10        20        30        40        50        60        70        80
                                            ....*....|....*....|....*....|....*....|....*....|....*....|....*....|....*....|
seqsig_6ee9921ccf62c31246641095efc256d2 848 LILLLDLDNTLLQATSFAKFNMElplenfvddngeaelykfYLPQYNFFYYLKFRPYVRQFLQILSLYYELAIYTNATRE 927
Cdd:pfam03031                             1 KTLVLDLDETLVHSSFEPDLPFD------------------FVLNFNHGVYVKKRPGLDEFLQELSKYYEIVIFTASSKE 62
                                                    90       100       110       120       130       140
                                            ....*....|....*....|....*....|....*....|....*....|....*....|....*.
seqsig_6ee9921ccf62c31246641095efc256d2 928 YADVVIAILDPDRTIFSDRIvARCSSTDRDENkyFSRIypNVDPKYVIAFDDRKDVWIDIPQSHIL 993
Cdd:pfam03031                            63 YADPVLDKLDPKKKYFKHRL-YRESCTFYVKD--LSLL--GRDLSRVVIVDNSPRSFLLQPDNGIP 123
```

Cd Length: 156 **Bit Score:** 101.97 **E-value:** 2.05e-24

```
                                                    10        20        30        40        50        60        70        80
                                            ....*....|....*....|....*....|....*....|....*....|....*....|....*....|....*....|
seqsig_6ee9921ccf62c31246641095efc256d2 843 LKDGKLILLLDLDNTLLQATSFAKFNM-ELPLENFVDDNGEAELYKFYLpqYNFFYYLKFRPYVRQFLQILSLYYELAIY 921
Cdd:TIGR02250                             2 LREKKLHLVLDLDQTLIHTTKDPTLSEwEKYDIEEPNSETRRDLRKFNL--GTMWYLTKLRPFLHEFLKEASKLYEMHVY 79
                                                    90       100       110       120       130       140       150       160
                                            ....*....|....*....|....*....|....*....|....*....|....*....|....*....|....*....|
seqsig_6ee9921ccf62c31246641095efc256d2 922 TNATREYADVVIAILDPDRTIFSDRIVARCSSTDRDEnKYFSRIYPNvDPKYVIAFDDRKDVWIDIPqSHILKAEHYNFF 1001
Cdd:TIGR02250                            80 TMGTRAYAQAIAKLIDPDGKYFGDRIISRDESGSPHT-KSLLRLFPA-DESMVVIIDDREDVWPWHK-RNLIQIEPYNYF 156
```

PF3D7_1363200 (HAD, AAA, PNK3P, AAA_33)

Cd Length: 139 **Bit Score:** 52.37 **E-value:** 1.51e-08

```
                                                    10        20        30        40        50        60        70        80
                                            ....*....|....*....|....*....|....*....|....*....|....*....|....*....|....*....|
seqsig_5f7efda8ba445ecf11a060a4ac68f9a8  42 FDLDNTLILSRGFFKPAQNEKdyiFYSDIIEFLKKKHEENYKIMIFSNQ--KGVSSgKLSLSNLINRVDDIIEKIGLP-- 117
Cdd:cd01427                               4 FDLDGTLLDSEPGIAEIEELE---LYPGVKEALKELKEKGIKLALATNKsrREVLE-LLEELGLDDYFDPVITSNGAAiy 79
                                                    90       100       110       120
                                            ....*....|....*....|....*....|....*....|....*
seqsig_5f7efda8ba445ecf11a060a4ac68f9a8 118 -LECYLALKNDKY--RKPRIGMYLFATQNYKQNLDEVIYVGDNAN 159
Cdd:cd01427                              80 yPKEGLFLGGGPFdiGKPNPDKLLAALKLLGVDPEEVLMVGDSLN 124
```

Cd Length: 131 **Bit Score:** 37.96 **E-value:** 9.68e-04

```
                                                    10        20        30        40        50        60        70
                                            ....*....|....*....|....*....|....*....|....*....|....*....|....*....|...
seqsig_5f7efda8ba445ecf11a060a4ac68f9a8 303 IILIGPPGCGKTFL----CTNYFSTYIRIN----MEEY--KTISKCIQVLKEKLLQRKNVI-IDNVNSLIKNR 364
Cdd:pfam00004                             1 LLLYGPPGTGKTTLakavAKELGAPFIEISgselVSKYvgESEKRLRELFEAAKKLAPCVIfIDEIDALAGSR 73
```

Cd Length: 158 **Bit Score:** 182.44 **E-value:** 4.80e-55

```
                                                    10        20        30        40        50        60        70        80
                                            ....*....|....*....|....*....|....*....|....*....|....*....|....*....|....*....|
seqsig_5f7efda8ba445ecf11a060a4ac68f9a8  38 KIFSFDLDNTLILSRGFFKPAQNEKDYIFYSD-IIEFLKKKHEENYKIMIFSNQKGVSS-GKLSLSNLINRVDDIIEKIG 115
Cdd:pfam08645                             1 KIAAFDLDGTLIKTKSGKVFPKDADDWKWLYPsVPEKLKKLHEDGYKIVIFTNQGGISTkGKKSAESFKNKIESILKKLD 80
                                                    90       100       110       120       130       140       150       160
                                            ....*....|....*....|....*....|....*....|....*....|....*....|....*....|....*....|
seqsig_5f7efda8ba445ecf11a060a4ac68f9a8 116 LPLECYLALKNDKYRKPRIGMYLFATQNYKQ----NLDEVIYVGDNANRIYdekfkqefinhlkyiysknnvninindiy 191
Cdd:pfam08645                            81 VPLQVYAATKKDKYRKPNTGMWDELLEDYNDgitiDLEKSFYVGDAAGRPA----------------------------- 131
                                                   170       180
                                            ....*....|....*....|....*....
seqsig_5f7efda8ba445ecf11a060a4ac68f9a8 192 krLKKDYTDTDLKFALNINAKFYTPEDLF 220
Cdd:pfam08645                           132 --RKKDFSDSDRKFALNVGIKFKTPEEFF 158
```

Cd Length: 143 **Bit Score:** 66.57 **E-value:** 3.36e-13

```
                                                    10        20        30        40        50        60        70        80
                                            ....*....|....*....|....*....|....*....|....*....|....*....|....*....|....*....|
seqsig_5f7efda8ba445ecf11a060a4ac68f9a8 302 LIILIGPPGCGKTFLCTNYFST--YIRIN--------------MEEYKTISK-----CIQVLKEKLLQRKNVIIDNVNS 359
Cdd:pfam13671                             1 LILMVGLPGSGKSTFARRLLRElgAVVLSsdtlrkrlrgdgppdISYYARASGrvyqrLLELAREALRAGRPVIVDATNL 80
                                                    90       100       110       120       130       140
                                            ....*....|....*....|....*....|....*....|....*....|....*....|....*..
seqsig_5f7efda8ba445ecf11a060a4ac68f9a8 360 LIKNRLLYINEAKkiNQNIEIRLIYFNYSKELIIHLNTFKliTDKTNKFNDVPTIAIHSFYKYVQEP 426
Cdd:pfam13671                            81 SREERARLIDLAR--RYGAPVRIVYLEAPEEVLRERNAQR--ERSGGDPSDVPEAVLDRMLARFEPP 143
```

PP2Cc Superfamily

PF3D7_0413500

Cd Length: 254 **Bit Score:** 224.90 **E-value:** 5.17e-67

```
                                                    10        20        30        40        50        60        70        80
                                            ....*....|....*....|....*....|....*....|....*....|....*....|....*....|....*....|
seqsig_c586bbade8990603eb7f47f84cafe49a 569 FKCGFYSFKGNRTYNEDRVIIIEDMNNflkeeydiltkkekkeyelmdeeylniinnikdmetPSYIYCAIYDGHNGDNA 648
Cdd:cd00143                               1 FSAGVSDKGGDRKTNEDAVVIKPNLNN-----------------------------------EDGGLFGVFDGHGGHAA 44
                                                    90       100       110       120       130       140       150       160
                                            ....*....|....*....|....*....|....*....|....*....|....*....|....*....|....*....|
```

```
seqsig_c586bbade8990603eb7f47f84cafe49a 649 VNIVQKLLHIHMYYYFINGNGL-----ENSLKYSFQEIDNYLCKNIINIKEEnhsnYSSGTTACVSVIFKNMLYVANIGD 723
Cdd:cd00143                              45 GEFASKLLVEELLEELEETLTLseediEEALRKAFLRADEEILEEAQDEPDD----ARSGTTAVVALIRGNKLYVANVGD 120
                                                   170       180       190       200       210       220       230       240
                                            ....*....|....*....|....*....|....*....|....*....|....*....|....*....|....*....|
seqsig_c586bbade8990603eb7f47f84cafe49a 724 SRCIISKNGRAIVLTVDHRASiNKKEQDRILKSGGILDDEGYLGGcLGVCRGFGSFHKKtkeklKGLICEPDLFHIKLTD 803
Cdd:cd00143                             121 SRAVLCRNGEAVQLTKDHKPV-NEEERERIEKAGGRVSNGRVPGV-LAVTRALGDFDLK-----PGVSAEPDVTVVKLTE 193
                                                   250       260       270       280       290       300
                                            ....*....|....*....|....*....|....*....|....*....|....*....|..
seqsig_c586bbade8990603eb7f47f84cafe49a 804 DDEFLIICCDGIFDVITSQEAVNTVKNSLiQSRDAKTAAEALCQLAYKKKSLDNLSVLVVIF 865
Cdd:cd00143                             194 DDDFLILASDGLWDVLSNQEAVDIVRSEL-AKEDLQEAAQELVDLALRRGSHDNITVVVVRL 254
```

PF3D7_0520100

Cd Length: 254 **Bit Score:** 148.24 **E-value:** 2.92e-40

```
                                                    10        20        30        40        50        60        70        80
                                            ....*....|....*....|....*....|....*....|....*....|....*....|....*....|....*....|
seqsig_1cebc56cc6f1fc0565117ffc97411f80 441 EKLKFLDELTDEQIMEYIKLAFLKTDEQFLKVSKFPNH----GCTIISLIIFRNKMFVANLGDCRAIgvvnISNTLKTEV 516
Cdd:cd00143                              58 EELEETLTLSEEDIEEALRKAFLRADEEILEEAQDEPDdarsGTTAVVALIRGNKLYVANVGDSRAV----LCRNGEAVQ 133
                                                    90       100       110       120       130       140       150       160
                                            ....*....|....*....|....*....|....*....|....*....|....*....|....*....|....*....|
seqsig_1cebc56cc6f1fc0565117ffc97411f80 517 LSNDHKPNDPKEKERIKKMGGDVIclqnVYRVKANarknnkdkpsllerlsmkeevyLAVSRAIGDKDFKFNnvISATPD 596
Cdd:cd00143                             134 LTKDHKPVNEEERERIEKAGGRVS----NGRVPGV---------------------LAVTRALGDFDLKPG--VSAEPD 185
                                                   170       180       190       200       210       220       230       240
                                            ....*....|....*....|....*....|....*....|....*....|....*....|....*....|....*....|
seqsig_1cebc56cc6f1fc0565117ffc97411f80 597 VICKEIYSEkcnekkeekeitkrtnvieksdiDEnyfketdnlyysahevnyhYVVMACDGVWDTMSNKDIVKILQTY-- 674
Cdd:cd00143                             186 VTVVKLTED-----------------------DD-------------------FLILASDGLWDVLSNQEAVDIVRSEla 223
                                                   250       260       270
                                            ....*....|....*....|....*....|.
seqsig_1cebc56cc6f1fc0565117ffc97411f80 675 NNDPDKACSEIIKTAYAYGSQDNLTAMLLKF 705
Cdd:cd00143                             224 KEDLQEAAQELVDLALRRGSHDNITVVVVRL 254
```

Cd Length: 252 **Bit Score:** 53.53 **E-value:** 4.82e-08

```
                                                    10        20        30        40        50        60        70        80
                                            ....*....|....*....|....*....|....*....|....*....|....*....|....*....|....*....|
seqsig_1cebc56cc6f1fc0565117ffc97411f80 156 TMQGRRKKQEDRYLVITDLTkyidsndyktlyfykkNPLYFYSIFDGHRGIKACEYCMSHIIKNIIYYFYNQNMEDDQSS 235
Cdd:smart00332                           15 SMQGVRKPMEDAHVITPDLS----------------DSGGFFGVFDGHGGSEAAKFLSKNLPEILAEELIKEKDELEDVE 78

                                            ....*
seqsig_1cebc56cc6f1fc0565117ffc97411f80 236 TTINK 240
Cdd:smart00332                           79 EALRK 83
```

PF3D7_0810300

Cd Length: 254 **Bit Score:** 142.08 **E-value:** 1.33e-38

```
                                                    10        20        30        40        50        60        70        80
                                            ....*....|....*....|....*....|....*....|....*....|....*....|....*....|....*....|
seqsig_9d655d09a0ab744fbbb896214ed95aa6 308 DEFNKENKKTKKENDPDVKE--EKSLttrkkkqkkKKMKKRKKRKNKIFFDSTMSGTTATIIvhLFKEKKLYVAYVGDSR 385
Cdd:cd00143                              54 EELLEELEETLTLSEEDIEEalRKAF---------LRADEEILEEAQDEPDDARSGTTAVVA--LIRGNKLYVANVGDSR 122
                                                    90       100       110       120       130       140       150       160
                                            ....*....|....*....|....*....|....*....|....*....|....*....|....*....|....*....|
seqsig_9d655d09a0ab744fbbb896214ed95aa6 386 AVLGKRKNgskqlsAVELTKDHKPNCAAEKKRILSSGGQVMklegdiPYRVFiknkfyPGLAMSRAIGDTIGHQiGIIAE 465
Cdd:cd00143                             123 AVLCRNGE------AVQLTKDHKPVNEEERERIEKAGGRVS------NGRVP------GVLAVTRALGDFDLKP-GVSAE 183
                                                   170       180       190       200       210       220       230
                                            ....*....|....*....|....*....|....*....|....*....|....*....|....*....|....*...
seqsig_9d655d09a0ab744fbbb896214ed95aa6 466 PDFIEVNINEDEDILVLiCSDGVWEFISSEEAVNLI-YEFGYNNVQDAVENLARESWDRWLNeeenivDDITIQAIYL 542
Cdd:cd00143                             184 PDVTVVKLTEDDDFLIL-ASDGLWDVLSNQEAVDIVrSELAKEDLQEAAQELVDLALRRGSH------DNITVVVVRL 254
```

Cd Length: 254 **Bit Score:** 60.80 **E-value:** 1.21e-10

```
                                                    10        20        30        40        50        60        70        80
                                            ....*....|....*....|....*....|....*....|....*....|....*....|....*....|....*....|
seqsig_9d655d09a0ab744fbbb896214ed95aa6 130 NGIGYVCRKGLKPesPNQDDFIIITM---ENLALYAIFDGHGpyGHDVSNYVQKELPYMIIKNENFL-----KNPKEVFT 201
Cdd:cd00143                               1 FSAGVSDKGGDRK--TNEDAVVIKPNlnnEDGGLFGVFDGHG--GHAAGEFASKLLVEELLEELEETltlseEDIEEALR 76
                                                    90
                                            ....*....|
seqsig_9d655d09a0ab744fbbb896214ed95aa6 202 KAFLNIHENI 211
Cdd:cd00143                              77 KAFLRADEEI 86
```

PF3D7_0810500

Cd Length: 254 **Bit Score:** 161.73 **E-value:** 1.16e-47

```
                                                    10        20        30        40        50        60        70        80
                                            ....*....|....*....|....*....|....*....|....*....|....*....|....*....|....*....|
seqsig_eafc4d443b683d18295bc8bbafe9e369  25 KGVKKVYEDEYLICENLKSFNKNLhpnfnfacFCLFDGHNGKNTAMFLKRNLAQELSNSFLEMQNTYDSSlpipdhfIKI 104
Cdd:cd00143                               9 GGDRKTNEDAVVIKPNLNNEDGGL--------FGVFDGHGGHAAGEFASKLLVEELLEELEETLTLSEED-------IEE 73
                                                    90       100       110       120       130       140       150       160
                                            ....*....|....*....|....*....|....*....|....*....|....*....|....*....|....*....|
seqsig_eafc4d443b683d18295bc8bbafe9e369 105 SVNNTCKRIDERIAQEYPNSRD----GATCVIVLIKDEYAYIINIGDSCAYLCRylnnSNQAIELVDIHKPWVITEKERI 180
Cdd:cd00143                              74 ALRKAFLRADEEILEEAQDEPDdarsGTTAVVALIRGNKLYVANVGDSRAVLCR----NGEAVQLTKDHKPVNEEERERI 149
                                                   170       180       190       200       210       220       230       240
                                            ....*....|....*....|....*....|....*....|....*....|....*....|....*....|....*....|
seqsig_eafc4d443b683d18295bc8bbafe9e369 181 IKHGGTIENGRVNDIIDVTRSFGDLSLkKYGLLCTGTFKKFKINSDDNFIILGTDGFFGSVDINYVINEITNLSKKEerl 260
Cdd:cd00143                             150 EKAGGRVSNGRVPGVLAVTRALGDFDL-KPGVSAEPDVTVVKLTEDDDFLILASDGLWDVLSNQEAVDIVRSELAKE--- 225
                                                   250       260       270       280
                                            ....*....|....*....|....*....|....*....|
seqsig_eafc4d443b683d18295bc8bbafe9e369 261 vnvekkktvfDAKSICNIMVEHAIvDKKSQDNVTVVLIKF 300
Cdd:cd00143                             226 ----------DLQEAAQELVDLAL-RRGSHDNITVVVVRL 254
```

PF3D7_1009600 (PP2Cc, FORK_HEAD_N)

Cd Length: 254 **Bit Score:** 83.92 **E-value:** 2.00e-18

```
                                                    10        20        30        40        50        60        70        80
                                            ....*....|....*....|....*....|....*....|....*....|....*....|....*....|....*....|
seqsig_32381123a43aa632633ef9260d301e41 266 PDKLESEDCCL-------NDQGFIAVADGVGSWIKYGINPRKYPEKFLQLLQKKINENENiQIEELLNYAYINNDKE--- 335
Cdd:cd00143                              10 GDRKTNEDAVVikpnlnnEDGGLFGVFDGHGGHAAGEFASKLLVEELLEELEETLTLSEE-DIEEALRKAFLRADEEile 88
                                                    90       100       110       120       130       140       150       160
                                            ....*....|....*....|....*....|....*....|....*....|....*....|....*....|....*....|
seqsig_32381123a43aa632633ef9260d301e41 336 ----------GSTTICLIIFNknDNTVSTANIGDSQFLIIRNNQIIYRSKP----QQYEFN----------FPYQLGSNA 391
Cdd:cd00143                              89 eaqdepddarSGTTAVVALIR--GNKLYVANVGDSRAVLCRNGEAVQLTKDhkpvNEEEREriekaggrvsNGRVPGVLA 166
                                                   170       180       190       200       210       220       230       240
                                            ....*....|....*....|....*....|....*....|....*....|....*....|....*....|....*....|
seqsig_32381123a43aa632633ef9260d301e41 392 VS--------KPN---DADIAHIEV-KKNDIIVVGTDGLWDNLYDSQILTIVKENNFATLSEKIANEAFSYSKMKrwmsp 459
Cdd:cd00143                             167 VTralgdfdlKPGvsaEPDVTVVKLtEDDDFLILASDGLWDVLSNQEAVDIVRSELAKEDLQEAAQELVDLALRR----- 241
                                                   250       260
                                            ....*....|....*....|...
seqsig_32381123a43aa632633ef9260d301e41 460 fiksynkefkchktgGKMDDITV 482
Cdd:cd00143                             242 ---------------GSHDNITV 249
```

Cd Length: 137 **Bit Score:** 43.04 **E-value:** 2.09e-05

```
                                                    10        20        30        40        50        60
                                            ....*....|....*....|....*....|....*....|....*....|....*....|
seqsig_32381123a43aa632633ef9260d301e41 122 NMNNMNNMNNVNNVNNMNNMNNMNNMNN----MNNMNNMNNMNNMNNMNN--MNNMNNHY 175
Cdd:pfam08430                             8 ESYSSVSGGMVYSMNSMNTYGPMNTSQGsansSMNMSGYAGPGAMNGMSSssMNGMSPGY 67
```

PF3D7_1135100

Cd Length: 254 **Bit Score:** 134.38 **E-value:** 2.49e-35

```
                                                    10        20        30        40        50        60        70        80
                                            ....*....|....*....|....*....|....*....|....*....|....*....|....*....|....*....|
seqsig_a38277b5127090cc13e0eaf86fa5d757 363 NSNE---YKNFQTSNPNFLFAAVIDGHAGGTIADVARKSLGYYLKKELIEIGVNSkrggcrERAIVSALKKAHLNFDNDL 439
Cdd:cd00143                              13 KTNEdavVIKPNLNNEDGGLFGVFDGHGGHAAGEFASKLLVEELLEELEETLTLS------EEDIEEALRKAFLRADEEI 86
                                                    90       100       110       120       130       140       150       160
                                            ....*....|....*....|....*....|....*....|....*....|....*....|....*....|....*....|
seqsig_a38277b5127090cc13e0eaf86fa5d757 440 LNQSKDYFLNGTSkyartGACSLSVLIDERNYYISNIGDSVGLLIKKHFYLPLNRIHNASEFNEKRKLLEEHPneedilv 519
Cdd:cd00143                              87 LEEAQDEPDDARS-----GTTAVVALIRGNKLYVANVGDSRAVLCRNGEAVQLTKDHKPVNEEERERIEKAGG------- 154
                                                   170       180       190       200       210       220       230       240
                                            ....*....|....*....|....*....|....*....|....*....|....*....|....*....|....*....|
seqsig_a38277b5127090cc13e0eaf86fa5d757 520 ckictrdyktvnnnntyelcktpfhllshHYDNCYVKGRLQPTRSFGDFHLKkkmfaysvdgtrlfvpephsfPYISAEP 599
Cdd:cd00143                             155 -----------------------------RVSNGRVPGVLAVTRALGDFDLK---------------------PGVSAEP 184
                                                   250       260       270       280       290       300       310       320
                                            ....*....|....*....|....*....|....*....|....*....|....*....|....*....|....*....|
seqsig_a38277b5127090cc13e0eaf86fa5d757 600 ELRVMKKHPDDQFIVLMSDGVYEFLNHAQVINVIKTY--GASPERAAKELINRVleaaayssgmtmkqllnldpsIRRNF 677
Cdd:cd00143                             185 DVTVVKLTEDDDFLILASDGLWDVLSNQEAVDIVRSElaKEDLQEAAQELVDLA--------------------LRRGS 243
                                                   330
                                            ....*....|.
seqsig_a38277b5127090cc13e0eaf86fa5d757 678 YDDVSVVVIKL 688
Cdd:cd00143                             244 HDNITVVVVRL 254
```

PF3D7_1208900

Cd Length: 252 **Bit Score:** 44.29 **E-value:** 1.18e-04

```
                                                     10        20        30        40        50        60        70        80
                                             ....*....|....*....|....*....|....*....|....*....|....*....|....*....|....*....|
seqsig_dfac32e1e5ee7da84045e1b6a3efc8f5  749 YGGQCRIGkVKGRCEDATFQT-DVPPA---FGIFDGVGswsleGIDASKFSiglslacqreAEKLSKNLNGYAKVSYNTI 824
Cdd:smart00332                            11 YGLSSMQG-VRKPMEDAHVITpDLSDSggfFGVFDGHG-----GSEAAKFL----------SKNLPEILAEELIKEKDEL 74
                                                     90       100       110       120       130       140       150       160
                                             ....*....|....*....|....*....|....*....|....*....|....*....|....*....|....*....|
seqsig_dfac32e1e5ee7da84045e1b6a3efc8f5  825 TRSKLLLKNSLESVKKEY---ADAYGSSTAIVGILDEytGKCGISSLGDSVCMILRREflpgdiNFERESYPKFAAESFL 901
Cdd:smart00332                            75 EDVEEALRKAFLSTDEEIleeLEALSGSTAVVALISG--NKLYVANVGDSRAVLCRNG------KAVQLTEDHKPSNEDE 146
                                                    170       180       190       200       210       220       230       240
                                             ....*....|....*....|....*....|....*....|....*....|....*....|....*....|....*....|
seqsig_dfac32e1e5ee7da84045e1b6a3efc8f5  902 YYNVKGRNPSIIRKIIwkttdqkweNGaPYQLSnlpdRSqwkdLENRGLHSFVkILEKvdidgDSPDMALTPPSEILcmp 981
Cdd:smart00332                           147 RARIEAAGGFVINGRV---------NG-VLALS----RA----IGDFFLKPYV-SAEP-----DVTVVELTEKDDFL--- 199
                                                    250       260       270
                                             ....*....|....*....|....*....|....*..
seqsig_dfac32e1e5ee7da84045e1b6a3efc8f5  982 gdliLLMSDGVSDNLFDEEIEAYCTFAIS--PEEACE 1016
Cdd:smart00332                           200 ----ILASDGLWDVLSNQEVVDIVRKHLSkdPKEAAK 232
```

PF3D7_1249300 (PP2C, DUF20, DUF20, GH47, PP2C)

Cd Length: 254 **Bit Score:** 167.50 **E-value:** 2.99e-46

```
                                                     10        20        30        40        50        60        70        80
                                             ....*....|....*....|....*....|....*....|....*....|....*....|....*....|....*....|
seqsig_8a6e1114bbb7d4e73c2c4b8f0f00d0e4  690 NKRENEDFYITKDildlNNVSESQGLCfysGIFDGHGGSNCARYVMNHLKTNViakfrqsflitckKKFKEKGSKLNELS 769
Cdd:cd00143                               11 DRKTNEDAVVIKP----NLNNEDGGLF---GVFDGHGGHAAGEFASKLLVEEL-------------LEELEETLTLSEED 70
                                                     90       100       110       120       130       140       150       160
                                             ....*....|....*....|....*....|....*....|....*....|....*....|....*....|....*....|
seqsig_8a6e1114bbb7d4e73c2c4b8f0f00d0e4  770 VElralyDSCIKGFDMTDKNYIELSKKYNYKD--GSTACIVLIYGpdddgsLKVLCANCGDSGAFICHNKKPIKLSLRHK 847
Cdd:cd00143                               71 IE-----EALRKAFLRADEEILEEAQDEPDDArsGTTAVVALIRG------NKLYVANVGDSRAVLCRNGEAVQLTKDHK 139
                                                    170       180       190       200       210       220       230       240
                                             ....*....|....*....|....*....|....*....|....*....|....*....|....*....|....*....|
seqsig_8a6e1114bbb7d4e73c2c4b8f0f00d0e4  848 PDLQEERIRILKCGGIIANINginriitkHKDRNNLnennnnkskektflalstSRSFGDISYKiprKIVQCKPFISVYT 927
Cdd:cd00143                              140 PVNEEERERIEKAGGRVSNGR--------VPGVLAV------------------TRALGDFDLK---PGVSAEPDVTVVK 190
                                                    250       260       270       280       290       300
                                             ....*....|....*....|....*....|....*....|....*....|....*....|....*
seqsig_8a6e1114bbb7d4e73c2c4b8f0f00d0e4  928 IDfDLDSFLVLATDGILNVLSDEEIIDIIWKNIHRK-PEQAAEEVVNEATRRGSTDDKTCTVIFF 991
Cdd:cd00143                              191 LT-EDDDFLILASDGLWDVLSNQEAVDIVRSELAKEdLQEAAQELVDLALRRGSHDNITVVVVRL 254
```

Cd Length: 234 **Bit Score:** 48.51 **E-value:** 2.54e-06

```
                                                  10        20        30        40
                                          ....*....|....*....|....*....|....*....|...
seqsig_8a6e1114bbb7d4e73c2c4b8f0f00d0e4  285 NKNEYNNDDDDNNNDDDDNNNDDDNNNNDDNNNDDNNNDDDNN 327
Cdd:pfam09849                            191 INNYGDDDSDAAGGDQGSNGDDDDGGFADSGYDDDDMDDDDDD 233
```

Cd Length: 234 **Bit Score:** 43.89 **E-value:** 9.26e-05

```
                                                  10        20        30
                                          ....*....|....*....|....*....|....*...
seqsig_8a6e1114bbb7d4e73c2c4b8f0f00d0e4  596 NNDDDDVDNDDVDDVDNDDDVDNDDDVDDDDIDDDDDN 633
Cdd:pfam09849                            196 DDDSDAAGGDQGSNGDDDDGGFADSGYDDDDMDDDDDD 233
```

Cd Length: 522 **Bit Score:** 39.32 **E-value:** 4.42e-03

```
                                                  10        20        30        40        50        60        70        80
                                          ....*....|....*....|....*....|....*....|....*....|....*....|....*....|....*....|
seqsig_8a6e1114bbb7d4e73c2c4b8f0f00d0e4  304 NNDDDNNNNDDNNNDDNNNDDDNNNNNNNNNNNSFSNNSFYNNPFYNNDDQCENETKC-KSYTHSTNINIRNTSNVSEVV 382
Cdd:PTZ00470                               1 NVNLREHLSVHNNADDNYNNNNNNNNQINSNNPNNNGNNQASKLPRGKKKQENPFNKIdEVYYQNEKLNIKRRESVREAM 80

                                          ....*.
seqsig_8a6e1114bbb7d4e73c2c4b8f0f00d0e4  383 KESEEK 388
Cdd:PTZ00470                              81 KHAWEG 86
```

Cd Length: 252 **Bit Score:** 124.79 **E-value:** 2.05e-31

```
                                                  10        20        30        40        50        60        70        80
                                          ....*....|....*....|....*....|....*....|....*....|....*....|....*....|....*....|
seqsig_8a6e1114bbb7d4e73c2c4b8f0f00d0e4  690 NKRENEDFYITKDILDLNNVSESQGlcfYSGIFDGHGGSNCARYVMNHLKTnvIAKFRQSFLitckkkfkekgsklneLS 769
Cdd:pfam00481                             11 FRKFMEDAHIAGKNLNASSGKDSFG---FFAVFDGHGGSQAAKYAGKHLET--ILALRRSFL----------------TL 69
                                                  90       100       110       120       130       140       150       160
                                          ....*....|....*....|....*....|....*....|....*....|....*....|....*....|....*....|
seqsig_8a6e1114bbb7d4e73c2c4b8f0f00d0e4  770 VELRALYDSCIKGFDMTDknyielSKKYNYKD---GSTACIVLIYGpdddgsLKVLCANCGDSGAFICHNKKPIK-LSLR 845
Cdd:pfam00481                             70 DKLDALRKSFLEADEELR------SDAANHEDlssGSTAVVALIRG------QKLYVANVGDSRAVLCRNGNAIKqLTED 137
                                                 170       180       190       200       210       220       230       240
                                          ....*....|....*....|....*....|....*....|....*....|....*....|....*....|....*....|
seqsig_8a6e1114bbb7d4e73c2c4b8f0f00d0e4  846 HKPDLQEERIRILKCGGiiaNINGINRIitkhkdrnnlnenNNNkskektflaLSTSRSFGDISYKIPR-KIVQCKPFIS 924
Cdd:pfam00481                            138 HKPSNEDERRRIRGAGG---FVSRNGRV-------------NGV---------LAVSRAFGDFELKKGKpQPVSAEPDVT 192
                                                 250       260       270       280       290       300
                                          ....*....|....*....|....*....|....*....|....*....|....*....|
seqsig_8a6e1114bbb7d4e73c2c4b8f0f00d0e4  925 VYTIDfDLDSFLVLATDGILNVLSDEEIIDIIWKNIH-RKPEQAAEEVVNEATRRGSTDD 983
Cdd:pfam00481                            193 SHKIT-ESDEFLILASDGLWDVLSDQEVVDIVRSELSdGSPMEAAEKLVDEAIAYGSEDN 251
```

PF3D7_1309200 (PP2C, GH47)

Cd Length: 254 **Bit Score:** 160.95 **E-value:** 2.04e-44

```
                                                 10        20        30        40        50        60        70        80
                                         ....*....|....*....|....*....|....*....|....*....|....*....|....*....|....*....|
seqsig_e0961706ec69eade5d623b82e5680c1f 544 ISVACKKGKKVdfPNQDDFTII---QTNDWILIMVFDGHGpsGHDISNFVHVVLPLLFSYNIEKIYENPVRTMKTlfYMI 620
Cdd:cd00143                               3 AGVSDKGGDRK--TNEDAVVIKpnlNNEDGGLFGVFDGHG--GHAAGEFASKLLVEELLEELEETLTLSEEDIEE--ALR 76
                                                 90       100       110       120       130       140       150       160
                                         ....*....|....*....|....*....|....*....|....*....|....*....|....*....|....*....|
seqsig_e0961706ec69eade5d623b82e5680c1f 621 NCYLVnysycINNNINPININFIDYNLSGTTCTIILynFITKKIYSAHTGDSRAVMGKQNpqtnkfSAYNITEDHKPSLK 700
Cdd:cd00143                              77 KAFLR-----ADEEILEEAQDEPDDARSGTTAVVAL--IRGNKLYVANVGDSRAVLCRNG------EAVQLTKDHKPVNE 143
                                                170       180       190       200       210       220       230       240
                                         ....*....|....*....|....*....|....*....|....*....|....*....|....*....|....*....|
seqsig_e0961706ec69eade5d623b82e5680c1f 701 LEKDRILAFGGEVKKLHgdvayrvfvkdeMYPGLAMSRAIGDiTSSFIGVTCEPTIKILDKLEEDKFIIVATDGIWEFIS 780
Cdd:cd00143                             144 EERERIEKAGGRVSNGR------------VPGVLAVTRALGD-FDLKPGVSAEPDVTVVKLTEDDDFLILASDGLWDVLS 210
                                                250       260       270       280
                                         ....*....|....*....|....*....|....*....|....*..
seqsig_e0961706ec69eade5d623b82e5680c1f 781 SEECVQMVSKKKKKKvhiAMEEIIKESWRRWARIDTVDDMTLVILYF 827
Cdd:cd00143                             211 NQEAVDIVRSELAKE---DLQEAAQELVDLALRRGSHDNITVVVVRL 254
```

Cd Length: 522 **Bit Score:** 39.32 **E-value:** 3.89e-03

```
                                                 10        20        30
                                         ....*....|....*....|....*....|....*..
seqsig_e0961706ec69eade5d623b82e5680c1f 167 HVNYKNEKREYDNNNNNNNNNNNNNNIFSNNNCNNS 203
Cdd:PTZ00470                              1 NVNLREHLSVHNNADDNYNNNNNNNNQINSNNPNNNG 37
```

PF3D7_1138500 (PP2C, PP2C, GH47, PP2C)

Cd Length: 254 **Bit Score:** 166.73 **E-value:** 3.53e-46

```
                                                 10        20        30        40        50        60        70        80
                                         ....*....|....*....|....*....|....*....|....*....|....*....|....*....|....*....|
seqsig_490942f1fa1b126783a87d8ec888dd58 626 YSCGSTALVAVILKGYLIVANAGDSRAIVCFNGNSLGMSTDHKPHLQTEEARIKKAGGYIANGRVDGNLNLTRAIGDLHY 705
Cdd:cd00143                              97 ARSGTTAVVALIRGNKLYVANVGDSRAVLCRNGEAVQLTKDHKPVNEEERERIEKAGGRVSNGRVPGVLAVTRALGDFDL 176
                                                 90       100       110       120       130
                                         ....*....|....*....|....*....|....*....|....*....|....*..
seqsig_490942f1fa1b126783a87d8ec888dd58 706 KrdpflpqkdQKISAFPEITCVTLTPEDEFLFLACDGIWDCKDGQDVVGFVKTRLEK 762
Cdd:cd00143                             177 K---------PGVSAEPDVTVVKLTEDDDFLILASDGLWDVLSNQEAVDIVRSELAK 224
```

Cd Length: 254 **Bit Score:** 80.06 **E-value:** 1.30e-16

```
                                                 10        20        30        40        50        60        70        80
                                         ....*....|....*....|....*....|....*....|....*....|....*....|....*....|....*....|
seqsig_490942f1fa1b126783a87d8ec888dd58  26 RYGLSCMQGWRKNMEDAHICYnnlkFNEIEEDVSIYGVFDGHGGPNVSKWISYNFRrifirclkeaneEMIKKNMKRSEN 105
Cdd:cd00143                               2 SAGVSDKGGDRKTNEDAVVIK----PNLNNEDGGLFGVFDGHGGHAAGEFASKLLV------------EELLEELEETLT 65
                                                 90       100       110
                                         ....*....|....*....|....*....|....
seqsig_490942f1fa1b126783a87d8ec888dd58 106 YKLKLIKLTLEKTFLKLDEEMLLSENQEKLKKYS 139
Cdd:cd00143                              66 LSEEDIEEALRKAFLRADEEILEEAQDEPDDARS 99
```

Cd Length: 522 **Bit Score:** 39.71 **E-value:** 3.24e-03

```
                                                10        20        30
                                          ....*....|....*....|....*....|....*....
seqsig_490942f1fa1b126783a87d8ec888dd58 206 NQDQPEHNQLYEDGNDNNNSNNNNNNNNNNISPNLYGTN 244
Cdd:PTZ00470                              1 NVNLREHLSVHNNADDNYNNNNNNNNQINSNNPNNNGNN 39
```

Cd Length: 252 **Bit Score:** 156.76 **E-value:** 1.21e-42

```
                                                10        20        30        40        50        60        70        80
                                          ....*....|....*....|....*....|....*....|....*....|....*....|....*....|....*....|
seqsig_490942f1fa1b126783a87d8ec888dd58 598 DDCNGVYSSEELRLFENYYSNDYEDNIaySCGSTALVAVILKGYLIVANAGDSRAIVCFNGNSLG-MSTDHKPHLQTEEA 676
Cdd:pfam00481                            70 DKLDALRKSFLEADEELRSDAANHEDL--SSGSTAVVALIRGQKLYVANVGDSRAVLCRNGNAIKqLTEDHKPSNEDERR 147
                                                90       100       110       120       130       140       150       160
                                          ....*....|....*....|....*....|....*....|....*....|....*....|....*....|....*....|
seqsig_490942f1fa1b126783a87d8ec888dd58 677 RIKKAGGYI-ANGRVDGNLNLTRAIGDLHYKrdpflPQKDQKISAFPEITCVTLTPEDEFLFLACDGIWDCKDGQDVVGF 755
Cdd:pfam00481                           148 RIRGAGGFVsRNGRVNGVLAVSRAFGDFELK-----KGKPQPVSAEPDVTSHKITESDEFLILASDGLWDVLSDQEVVDI 222

                                          ....*..
seqsig_490942f1fa1b126783a87d8ec888dd58 756 VKTRLEK 762
Cdd:pfam00481                           223 VRSELSD 229
```

PF3D7_1455000

Cd Length: 254 **Bit Score:** 184.84 **E-value:** 3.69e-55

```
                                                10        20        30        40        50        60        70        80
                                          ....*....|....*....|....*....|....*....|....*....|....*....|....*....|....*....|
seqsig_a5b97053d8b55da50f7c5abd9409fa51  21 HVCAGTMQGYRATEEDATVILASLKNfPSCRMCTIFDGHIGKETALYCA----RNIADFIGNCTTLDVNNITNAC----I 92
Cdd:cd00143                               2 SAGVSDKGGDRKTNEDAVVIKPNLNN-EDGGLFGVFDGHGGHAAGEFASkllvEELLEELEETLTLSEEDIEEALrkafL 80
                                                90       100       110       120       130       140       150       160
                                          ....*....|....*....|....*....|....*....|....*....|....*....|....*....|....*....|
seqsig_a5b97053d8b55da50f7c5abd9409fa51  93 QMDNEILNSNFAHN-----GSTAIIAIIEKiinkdfFKLYICNLGDSRAMLIKKdGSFISLSEDHKPYNKKEKERIYKIG 167
Cdd:cd00143                              81 RADEEILEEAQDEPddarsGTTAVVALIRG------NKLYVANVGDSRAVLCRN-GEAVQLTKDHKPVNEEERERIEKAG 153
                                               170       180       190       200       210       220       230       240
                                          ....*....|....*....|....*....|....*....|....*....|....*....|....*....|....*....|
seqsig_a5b97053d8b55da50f7c5abd9409fa51 168 GFVENGRILGYIGVSRSFGDKNYKIksdcpynphetMISCIPDIKIFY-ANCDDILFLGCDGLFEMLSWNDVAKFTYDCM 246
Cdd:cd00143                             154 GRVSNGRVPGVLAVTRALGDFDLKP-----------GVSAEPDVTVVKlTEDDDFLILASDGLWDVLSNQEAVDIVRSEL 222
                                               250       260       270
                                          ....*....|....*....|....*....|..
seqsig_a5b97053d8b55da50f7c5abd9409fa51 247 NRHTLSDAVINILDYALLSGSKDNITIQIIKF 278
Cdd:cd00143                             223 AKEDLQEAAQELVDLALRRGSHDNITVVVVRL 254
```

MPP Superfamily

PF3D7_0107800 (MPP, MPP, Mre11, Mre11)

Cd Length: 223 **Bit Score:** 94.74 **E-value:** 2.58e-21

```
                                                10        20        30        40        50        60        70        80
                                          ....*....|....*....|....*....|....*....|....*....|....*....|....*....|....*....|
seqsig_04f45037ed66a56e4dcd4c97497968d2  693 FKKL----IPFYTIHGNHDYPYSYEYISPLDILNisnlinYIGKNNLNNIVVKPILLNKYKSKISIYAVGWMKDERLYRS 768
Cdd:cd00840                                69 LRRLkeagIPVFIIAGNHDSPSRLGALSPLLALS------GLHLVGVEEDVLTPLLLPKGGTGVAIYGLPYLRRSRLRDL 142
                                                90       100       110       120       130       140       150       160
                                          ....*....|....*....|....*....|....*....|....*....|....*....|....*....|....*....|
seqsig_04f45037ed66a56e4dcd4c97497968d2  769 FENNEvkFILPSDYKNRINILVLHQNRYIRNAYGNNTKNFIKESFIPKFIDLVIWGHEHfsKPYLEESilNSFYNIQLGS 848
Cdd:cd00840                               143 LADAE--LRPRPLDPDDFNILLLHGGVAGAGPSDSERAPFVPEALLPAGFDYVALGHIH--RPQIILG--GGPPIVYPGS 216

                                          ....*.
seqsig_04f45037ed66a56e4dcd4c97497968d2  849 SVRTSI 854
Cdd:cd00840                               217 PEGLSF 222
```

Cd Length: 223 **Bit Score:** 73.17 **E-value:** 2.53e-14

```
                                                10        20        30        40        50        60        70
                                          ....*....|....*....|....*....|....*....|....*....|....*....|....*....|....*.
seqsig_04f45037ed66a56e4dcd4c97497968d2  352 KILLCTDNHLGYKENNSIQ---KKDSFNSFEEILFIAKKLNVDMILNSGDLFHKNKVSEYTLFKSMYIIRKYCHIN 424
Cdd:cd00840                                 1 RFLHTADWHLGKPLKGLSRdrrREDQFEAFEEIVELAIEEKVDFVLIAGDLFDSNNPSPEALELLIEALRRLKEAG 76
```

Cd Length: 166 **Bit Score:** 48.34 **E-value:** 1.61e-06

```
                                                10        20        30        40        50        60
                                          ....*....|....*....|....*....|....*....|....*....|....*....|...
seqsig_04f45037ed66a56e4dcd4c97497968d2 1032 EKPLLKIKVEY-DDVNIINTQLFGSLFINSIANPSEFLSFYRKKIRQRDIYNNNDNNNDNIND 1093
Cdd:pfam04152                             65 PLPLIRLRVDYsGGFEVENPQRFGQRFVGKVANPNDILQFYKKKKRKTKTKKTEEDDPDEEEL 127
```

Cd Length: 166 **Bit Score:** 41.79 **E-value:** 2.59e-04

```
                                                10        20        30        40        50
                                          ....*....|....*....|....*....|....*....|....*....|....*....
seqsig_04f45037ed66a56e4dcd4c97497968d2  874 FRFLKINLETVRPFEMKDIKLADYELNFKS---ESVLKeFLHEQTHAILEKIKNNFSHE 929
Cdd:pfam04152                              1 FRLTPIPLKTVRPFVMKEVVLSEEPLALDPndkDEVTK-FLIEKVEEMIEEAKEEWLEL 58
```

PF3D7_0314400 (PP2A_PP4_PP6)

Cd Length: 285 **Bit Score:** 508.30 **E-value:** 0e+00

```
                                                10        20        30        40        50        60        70        80
                                          ....*....|....*....|....*....|....*....|....*....|....*....|....*....|....*....|
seqsig_0de7cb891a67efe427d37d54736c57d5   8 KWIEQLRMNppKLLDESDLRLVCQRVKEILVEENNVQSIKPPVIICGDIHGQFFDLLELFDVGGDIMNNDYIFLGDYVDR 87
Cdd:cd07415                               4 KWIEQLKKC--ELLPESEVKSLCEKAKEILVKESNVQRVRSPVTVCGDIHGQFYDLLELFRVGGDPPDTNYLFLGDYVDR 81
                                                90       100       110       120       130       140       150       160
                                          ....*....|....*....|....*....|....*....|....*....|....*....|....*....|....*....|
seqsig_0de7cb891a67efe427d37d54736c57d5  88 GYNSVETFEYLLLLKLLFPKNITLLRGNHESRQITTVYGFYDECFKKYGNANAWKYCTDIFDYLTLAALVDNQIFCVHGG 167
Cdd:cd07415                              82 GYYSVETFLLLLALKVRYPDRITLLRGNHESRQITQVYGFYDECLRKYGNANVWKYCTDLFDYLPLAALIDNQIFCVHGG 161
                                               170       180       190       200       210       220       230       240
                                          ....*....|....*....|....*....|....*....|....*....|....*....|....*....|....*....|
```

```
seqsig_0de7cb891a67efe427d37d54736c57d5 168 LSPEIKLIDQLRLINRVQEIPHEGAFGDIMWSDPDEVDDWVANPRGAGWLFGPNVTKKFNHINNLELIARAHQLAMEGYR 247
Cdd:cd07415                             162 LSPSIDTLDQIRAIDRFQEVPHEGPMCDLLWSDPDDIEGWGISPRGAGYLFGQDVVEEFNHNNGLTLICRAHQLVMEGYQ 241
                                                   250       260       270       280
                                            ....*....|....*....|....*....|....*....|....*
seqsig_0de7cb891a67efe427d37d54736c57d5 248 YMFEDsTIITVWSAPNYCYRCGNVAAIMRIDEYMNRQMLIFKDTP 292
Cdd:cd07415                             242 WMFDD-KLVTVWSAPNYCYRCGNVASIMELDEHLKRSFKVFEAAP 285
```

PF3D7_0802800 (PP2B, CEMA1)

Cd Length: 305 **Bit Score:** 522.25 **E-value:** 0e+00

```
                                                    10        20        30        40        50        60        70        80
                                            ....*....|....*....|....*....|....*....|....*....|....*....|....*....|....*....|
seqsig_ca72928db61020290345a6ade86a7e87  38 PDYKALRDHLKKEGRIRKEDCLDIIKKVIDIVSNEPNLLRLKDPITIVGDIHGQYYDLLKLLEVGGNPDHTQFLFLGDYV 117
Cdd:cd07416                               1 PRIDVLKAHFMREGRLSEEDALRIITEGAEILRQEPNLLRIEAPVTVCGDIHGQFYDLLKLFEVGGSPANTRYLFLGDYV 80
                                                    90       100       110       120       130       140       150       160
                                            ....*....|....*....|....*....|....*....|....*....|....*....|....*....|....*....|
seqsig_ca72928db61020290345a6ade86a7e87 118 DRGSFSIEVLLLLYALKINFPDRIWLIRGNHECRQMTTFFNFRDECEYKYDIVVYYAFMESFDTIPLSAVINGKFLGVHG 197
Cdd:cd07416                              81 DRGYFSIECVLYLWALKILYPKTLFLLRGNHECRHLTEYFTFKQECKIKYSERVYDACMEAFDCLPLAALMNQQFLCVHG 160
                                                   170       180       190       200       210       220       230       240
                                            ....*....|....*....|....*....|....*....|....*....|....*....|....*....|....*....|
seqsig_ca72928db61020290345a6ade86a7e87 198 GLSPDLILLNQICSFTRFQEPPRSGIFCDILWSDPIDEDKEEHTiqTESYFPNDIRGCSYFFGYNAATTFLEKNGLLSII 277
Cdd:cd07416                             161 GLSPELKTLDDIRKLDRFREPPAFGPMCDLLWSDPLEDFGNEKT--QEHFVHNTVRGCSYFYSYRAVCEFLQKNNLLSII 238
                                                   250       260       270       280       290       300
                                            ....*....|....*....|....*....|....*....|....*....|....*....|....*..
seqsig_ca72928db61020290345a6ade86a7e87 278 RAHEAQLEGYKMHQTNLKTGFPIVITIFSAPNYCDVYNNKGAVLKFDSNTLNIQQFSFSPHPYHLPN 344
Cdd:cd07416                             239 RAHEAQDAGYRMYRKSQTTGFPSLITIFSAPNYLDVYNNKAAVLKYENNVMNIRQFNCSPHPYWLPN 305
```

Cd Length: 286 **Bit Score:** 38.35 **E-value:** 3.57e-03

```
                                                    10        20        30        40        50        60        70        80
                                            ....*....|....*....|....*....|....*....|....*....|....*....|....*....|....*....|
seqsig_ca72928db61020290345a6ade86a7e87 395 IEENNKRINEmnfnnNDDNVQYEDNGPyinqsnnnnnnnNKDNKFDDITYDDHKKEKDKRNKISSNGNMQDNNQLYDHSE 474
Cdd:pfam07418                           170 VEETLSTIHE-----DDASTLHEDDEL------------DEEVTSYLNELDDEVTSYFNDGENEENDDELEAEVISYLKD 232
                                                    90       100       110       120       130
                                            ....*....|....*....|....*....|....*....|....*....|
seqsig_ca72928db61020290345a6ade86a7e87 475 GHNNYNDEDEFFKNVK--KTDTNNNNNNEEEDEEEDEEEEEDGRKTKDVG 522
Cdd:pfam07418                           233 GENDNEVKEKIRREYRewKGDKANTNETEIEDESEDEYEEEAGEEQENED 282
```

PF3D7_0912400 (PhoD, PhoD)

Cd Length: 228 **Bit Score:** 92.49 **E-value:** 1.18e-21

```
                                                    10        20        30        40        50        60        70        80
                                            ....*....|....*....|....*....|....*....|....*....|....*....|....*....|....*....|
seqsig_e5aa1cc5d5fed3e76e3793edbd42b71d  32 NFVFLSCNYQKGKVNN---NLLNSIEKRKPQLMLWIGDYFY-------------------TECSEIKCLDDAYTYIKKD 88
Cdd:cd07389                               1 RFAFGSCNKYESGYFNayrALAYDHSEEDPDLFLHLGDQIYaddvgglmpaliegrplepaHEALTLEEYRERYRQYRSD 80
                                                    90       100       110       120       130       140       150       160
                                            ....*....|....*....|....*....|....*....|....*....|....*....|....*....|....*....|
seqsig_e5aa1cc5d5fed3e76e3793edbd42b71d  89 PFYMKLKKKFKIDGIYDDHDYNKNNGD---------RLYKYKKESKKKYLDYLNVDKND-VRYKRNGAYISKLYIDPdne 158
Cdd:cd07389                              81 PDLQRLLAQVPTIGIWDDHDIGDNWGGdgawvqdspVFYARKAAARQAYLEFQPVRNPSpRRGGRGGIYRSFRFGDL--- 157
                                                   170
                                            ....*....|....*.
seqsig_e5aa1cc5d5fed3e76e3793edbd42b71d 159 knqVKIIMLDTRYNKD 174
Cdd:cd07389                             158 ---VDLILLDTRTYRD 170
```

Cd Length: 228 **Bit Score:** 57.05 **E-value:** 9.92e-10

```
                                                    10        20        30        40        50
                                            ....*....|....*....|....*....|....*....|....*....|....*....
seqsig_e5aa1cc5d5fed3e76e3793edbd42b71d 251 ENWGLMPYSLRRLRELIKKTKPKGLLFLSGDVHFGSI-------IGKEESVIEVTSSSV 302
Cdd:cd07389                             170 DSWDGYPAERERLLDLLAKRKIKNVVFLSGDVHLAEAsdlpldaPGDGYVLVEFTSSGL 228
```

PF3D7_0918000 (ACP5)

Cd Length: 277 **Bit Score:** 264.93 **E-value:** 7.16e-86

```
                                                    10        20        30        40        50        60        70        80
                                            ....*....|....*....|....*....|....*....|....*....|....*....|....*....|....*....|
seqsig_fc33fe615df1a2010d32d29cedce6d2d  27 LRFASLGDWG-KDTKGQILNAKYFKQFIKNERVTFIVSPGSNFID-GVKGLNDPAWKNLYEDVYSEEKgdMYMPFFTVLG 104
Cdd:cd07378                               1 LRFLALGDWGgGGTAGQKAVAKAMAKVAAELGPDFILSLGDNFYDdGVGSVDDPRFETTFEDVYSAPS--LQVPWYLVLG 78
                                                    90       100       110       120       130       140       150       160
                                            ....*....|....*....|....*....|....*....|....*....|....*....|....*....|....*....|
seqsig_fc33fe615df1a2010d32d29cedce6d2d 105 TRDWTGNYNAQLLKGQgiyiekngetsiekdadATNYPKWIMPNYWYHYFTHFTVSsgpsivktghkDLAAAFIFIDTWV 184
Cdd:cd07378                              79 NHDYSGNVSAQIDYTK-----------------RPNSPRWTMPAYYYRVSFPFPSS-----------DTTVEFIMIDTVP 130
                                                   170       180       190       200       210       220       230       240
                                            ....*....|....*....|....*....|....*....|....*....|....*....|....*....|....*....|
seqsig_fc33fe615df1a2010d32d29cedce6d2d 185 LSSNF---------PYKKIHEKAWNDLKSQLSVAKkiADFIIVVGDQPIYSSGYSRGSSYLAYYLLPLLKDAEVDLYISG 255
Cdd:cd07378                             131 LCGNSddiaspygpPNGKLAEEQLAWLEKTLAAST--ADWKIVVGHHPIYSSGEHGPTSCLVDRLLPLLKKYKVDAYLSG 208
                                                   250       260       270       280       290       300
                                            ....*....|....*....|....*....|....*....|....*....|....*....|....*....
seqsig_fc33fe615df1a2010d32d29cedce6d2d 256 HDNNMEVIEDND--MAHITCGSGSMSQGKSGMKNSK----SLFFSSDIGFCVHELSNNGIVTKFVSSKK 318
Cdd:cd07378                             209 HDHNLQHIKDDGsgTSFVVSGAGSKARPSVKHIDKVpqffSGFTSSGGGFAYLELTKEELTVRFYDADG 277
```

PF3D7_0925400 (PP2A_PP4_PP6)

Cd Length: 285 **Bit Score:** 546.05 **E-value:** 0e+00

```
                                                    10        20        30        40        50        60        70        80
                                            ....*....|....*....|....*....|....*....|....*....|....*....|....*....|....*....|
seqsig_20ffc2a594aee58830a2d2c43e515092 160 NVDEWICKLLKCELLKIEEVKLMCDLLIDILKNEENCVRINVPVTVAGDIHGQFFDLLELFHIGGLPPDVNYLFLGDYVD 239
Cdd:cd07415                               1 DLDKWIEQLKKCELLPESEVKSLCEKAKEILVKESNVQRVRSPVTVCGDIHGQFYDLLELFRVGGDPPDTNYLFLGDYVD 80
                                                    90       100       110       120       130       140       150       160
                                            ....*....|....*....|....*....|....*....|....*....|....*....|....*....|....*....|
seqsig_20ffc2a594aee58830a2d2c43e515092 240 RGYYSCECFCLVACFKIKYPSRVTLLRGNHESRQITKVYGFYDECIRKYNNNNiVWKYLTDVFDYLPLTAIINDEIFCDH 319
Cdd:cd07415                              81 RGYYSVETFLLLLALKVRYPDRITLLRGNHESRQITQVYGFYDECLRKYGNAN-VWKYCTDLFDYLPLAALIDNQIFCVH 159
                                                   170       180       190       200       210       220       230       240
```

```
                                             ....*....|....*....|....*....|....*....|....*....|....*....|....*....|....*....|
seqsig_20ffc2a594aee58830a2d2c43e515092  320 GGISPCLQTIDEINKLDRFKEIPQDGAICDLLWSDPagpeDEItEGWKPSPRGAGVLFSEEKTESFLRMNKLSCICRAHQ 399
Cdd:cd07415                              160 GGLSPSIDTLDQIRAIDRFQEVPHEGPMCDLLWSDP----DDI-EGWGISPRGAGYLFGQDVVEEFNHNNGLTLICRAHQ 234
                                                    250       260       270       280       290
                                             ....*....|....*....|....*....|....*....|....*....|.
seqsig_20ffc2a594aee58830a2d2c43e515092  400 LVQDGFQWMHNDKVVTIFSAPNYCYRCGNCASLMLVDEFMEKDFITFNTAP 450
Cdd:cd07415                              235 LVMEGYQWMFDDKLVTVWSAPNYCYRCGNVASIMELDEHLKRSFKVFEAAP 285
```

PF3D7_0927700 (PP2A_PP4_PP6)

Cd Length: 285 **Bit Score:** 502.53 **E-value:** 3.06e-180

```
                                                     10        20        30        40        50        60        70        80
                                             ....*....|....*....|....*....|....*....|....*....|....*....|....*....|....*....|
seqsig_f7b5e0e4c4612c0aa83fd3398b4edcd8    5 DLDKVIDILKKCKLIEEREVRNLCSEAKLLLSKEDNVRNVDIPVIICGDIHGQFHDLKELFNIGNELPHVNYIFLGDYVD 84
Cdd:cd07415                                1 DLDKWIEQLKKCELLPESEVKSLCEKAKEILVKESNVQRVRSPVTVCGDIHGQFYDLLELFRVGGDPPDTNYLFLGDYVD 80
                                                     90       100       110       120       130       140       150       160
                                             ....*....|....*....|....*....|....*....|....*....|....*....|....*....|....*....|
seqsig_f7b5e0e4c4612c0aa83fd3398b4edcd8   85 RGKYSIETFLLLLALKIKYPLHLTLIRGNHESRQISEIYGFYDECLKKYGSINVWKYCTDVFDYLCIGAIIEGKYFCIHG 164
Cdd:cd07415                               81 RGYYSVETFLLLLALKVRYPDRITLLRGNHESRQITQVYGFYDECLRKYGNANVWKYCTDLFDYLPLAALIDNQIFCVHG 160
                                                    170       180       190       200       210       220       230       240
                                             ....*....|....*....|....*....|....*....|....*....|....*....|....*....|....*....|
seqsig_f7b5e0e4c4612c0aa83fd3398b4edcd8  165 GLSPSIEKIDELKKIYRFQEIPKNGPLCDIMWSDPNDQNGWTKSPRGAGHLYGQDIVEKFCYINNIHIIARAHQLVMEGY 244
Cdd:cd07415                              161 GLSPSIDTLDQIRAIDRFQEVPHEGPMCDLLWSDPDDIEGWGISPRGAGYLFGQDVVEEFNHNNGLTLICRAHQLVMEGY 240
                                                    250       260       270       280
                                             ....*....|....*....|....*....|....*....|....
seqsig_f7b5e0e4c4612c0aa83fd3398b4edcd8  245 KWSFNKKLVTIWSAPNYCYRCGNIASIMEIDENLFFNFKRFGPS 288
Cdd:cd07415                              241 QWMFDDKLVTVWSAPNYCYRCGNVASIMELDEHLKRSFKVFEAA 284
```

PF3D7_1018200 (Bsu1)

Cd Length: 311 **Bit Score:** 230.37 **E-value:** 1.86e-66

```
                                                     10        20        30        40        50        60        70        80
                                             ....*....|....*....|....*....|....*....|....*....|....*....|....*....|....*....|
seqsig_d2fc240643b7616d38b45462f76bd1f6 1212 TYNDILLLCDEVAKLFKMENSLENVTLPCKVFGDVHGNLFDLLDFFNLYNWPMHNNKNllltesdlrkfeyihndkdihi 1291
Cdd:cd07419                               22 NWNEILELCDAAEDIFKQEPMVLRLRAPIKIFGDIHGQFGDLMRLFDEYGSPVTEAAG---------------------- 79
                                                     90       100       110       120       130       140       150       160
                                             ....*....|....*....|....*....|....*....|....*....|....*....|....*....|....*....|
seqsig_d2fc240643b7616d38b45462f76bd1f6 1292 dknkDMQYNDndikYVFLGNLINRGNYSLEVICLLFSLKILFPKHIYLLRGNHEDRLFNYIYGFYKDIEIKMKTNmeymg 1371
Cdd:cd07419                               80 ----DIEYID----YLFLGDYVDRGSNSLETICLLLALKVKYPNQIHLIRGNHEDRDINALFGFREECKERLGED----- 146
                                                    170       180       190       200       210       220       230       240
                                             ....*....|....*....|....*....|....*....|....*....|....*....|....*....|....*....|
seqsig_d2fc240643b7616d38b45462f76bd1f6 1372 iINYQEQVisahsyelFNRINDVFEFFPLCVLLDKNILCIHGGIGDSVMTISDYQHIRKPilIPQNVDrysnnksehvKK 1451
Cdd:cd07419                              147 -PNDGDSV--------WRRINRLFEWLPLAAIIEDKILCMHGGIGRSINHVSEIEDLKRP--LTMEFG----------EQ 205
                                                    250       260       270       280       290       300       310       320
                                             ....*....|....*....|....*....|....*....|....*....|....*....|....*....|....*....|
seqsig_d2fc240643b7616d38b45462f76bd1f6 1452 IIIDTLWSDPINyNDELdLQLLKNsstyDIIPSRRGNItFKFGKHRLNDFLKNNQLKLIIRGHECVQEGYKYSYKRKLLT 1531
Cdd:cd07419                              206 VVMDLLWSDPTE-NDSV-LGLRPN----AIDPRGPGLI-VKFGPDRVHRFLEENDLQMIIRAHECVMDGFERFAQGKLIT 278
                                                    330       340
                                             ....*....|....*....|....*...
seqsig_d2fc240643b7616d38b45462f76bd1f6 1532 LFSAKNYCNKYnNNASNAFIVRKNKNII 1559
Cdd:cd07419                              279 LFSATNYCGTA-GNAGAILVLGRDLTII 305
```

PF3D7_1206000 (SHELPHS)

Cd Length: 208 **Bit Score:** 165.50 **E-value:** 1.40e-49

```
                                                     10        20        30        40        50        60        70        80
                                             ....*....|....*....|....*....|....*....|....*....|....*....|....*....|....*....|
seqsig_44cb9be7fec22f1573a259a96417f992   39 FSISDLHSDLDALKKTLLTENIIDEENNAIRENVFVIITGDVLDPAYDDIEILYFIQNYNIKAKPLNSKIQLILGNHEVQ 118
Cdd:cd07425                                1 VAIGDLHGDLDAFREILKGAGVIDSNDHWIGGSTHLVQLGDIFDRGPDVIEILWLLYKLEQEAAKAGGKVHFLLGNHELM 80
                                                     90       100       110       120       130       140       150       160
                                             ....*....|....*....|....*....|....*....|....*....|....*....|....*....|....*....|
seqsig_44cb9be7fec22f1573a259a96417f992  119 NICLDF-IGNKKYGEEY----KARNKLFKKGEVLYNYLLDLPFVIKVNDILFSHASIlpyyakrgidyindegrseiknn 193
Cdd:cd07425                               81 NLCGDFrYVHPKYFNEFgglaMRRRELFSPGGELGRWLRSKPVIVKVNDTLFVHGGL----------------------- 137
                                                    170       180       190       200       210       220       230       240
                                             ....*....|....*....|....*....|....*....|....*....|....*....|....*....|....*....|
seqsig_44cb9be7fec22f1573a259a96417f992  194 ctllkfkrktgqrfcvccynGPTFNRYFSRaaEMPFRREVCKSLFKTLNKLSAKKLVNGHTIQRNrKVNEYCKGGLIMAD 273
Cdd:cd07425                              138 --------------------GPLWYRGYSK--ETSDKECAAAHLDKVLERLGAKRMVVGHTPQEG-GIVTFCGGKVIRID 194
                                                    250
                                             ....*....|....
seqsig_44cb9be7fec22f1573a259a96417f992  274 TGISKWKYGVINYV 287
Cdd:cd07425                              195 VGMSKGVYGGPLEV 208
```

PF3D7_1469200 (SHELPHS)

Cd Length: 208 **Bit Score:** 244.08 **E-value:** 2.61e-79

```
                                                     10        20        30        40        50        60        70        80
                                             ....*....|....*....|....*....|....*....|....*....|....*....|....*....|....*....|
seqsig_590ff0ac21e2d4dc67c52d5efa98faad   59 IAIGDIHGDVESLKLILRHSNLIDENDEWIAEDVMLVQVGDILDRGVFGTYIYDYLLKLQKDAIKKNSKVILIMGNHEQL 138
Cdd:cd07425                                1 VAIGDLHGDLDAFREILKGAGVIDSNDHWIGGSTHLVQLGDIFDRGPDVIEILWLLYKLEQEAAKAGGKVHFLLGNHELM 80
                                                     90       100       110       120       130       140       150       160
                                             ....*....|....*....|....*....|....*....|....*....|....*....|....*....|....*....|
seqsig_590ff0ac21e2d4dc67c52d5efa98faad  139 NLCGAFHYVNESEVMLFFQNNRnnRLFSFTNKNGYYFKKLIRLPVIVKINNIIFTHGGIskhmseydintinlktrlqie 218
Cdd:cd07425                               81 NLCGDFRYVHPKYFNEFGGLAM--RRRELFSPGGELGRWLRSKPVIVKVNDTLFVHGGL--------------------- 137
                                                    170       180       190       200       210       220       230       240
                                             ....*....|....*....|....*....|....*....|....*....|....*....|....*....|....*....|
seqsig_590ff0ac21e2d4dc67c52d5efa98faad  219 nkckmfqfekynylskeGVLWNNEISHQVKLQPKKTcKHLRNILKKYNAKGLVVGHTRQKSHEIqTYCNNSFFLIDTGMS 298
Cdd:cd07425                              138 -----------------GPLWYRGYSKETSDKECAA-AHLDKVLERLGAKRMVVGHTPQEGGIV-TFCGGKVIRIDVGMS 198

                                             ....*....
seqsig_590ff0ac21e2d4dc67c52d5efa98faad  299 LFMNNGQPY 307
Cdd:cd07425                              199 KGVYGGPLE 207
```

PF3D7_1340600 (Dbr1_N)

Cd Length: 262 **Bit Score:** 444.35 **E-value:** 1.27e-153

```
                                                   10        20        30        40        50        60        70        80
                                            ....*....|....*....|....*....|....*....|....*....|....*....|....*....|....*....|
seqsig_9acb7d07ed17bc4826c0beccb414b2c0   3 IAVVGCTHGELDLIYSTLEKIEEENKIKVDLLICCGDFQSVRYNVDNECLNVPAKYKKEQnDFVDYFTGKKKAKILTIFV 82
Cdd:cd00844                               1 IAVEGCCHGELDKIYETLEKIEKKEGTKVDLLICCGDFQAVRNEADLKCMAVPPKYRKMG-DFYKYYSGEKKAPILTIFI 79
                                                   90       100       110       120       130       140       150       160
                                            ....*....|....*....|....*....|....*....|....*....|....*....|....*....|....*....|
seqsig_9acb7d07ed17bc4826c0beccb414b2c0  83 GGNHEAMNVLKQLYYGGWVAPNIYYLGYSSVHNINNFRICSLSGIYKKYSFFKKYYESYPYTDITKVSAYHIRKYEIEKL 162
Cdd:cd00844                              80 GGNHEASNYLWELPYGGWVAPNIYYLGYAGVVNFGGLRIAGLSGIYKSHDYRKGHFERPPYSEDTKRSAYHVRNIEVFKL 159
                                                  170       180       190       200       210       220       230       240
                                            ....*....|....*....|....*....|....*....|....*....|....*....|....*....|....*....|
seqsig_9acb7d07ed17bc4826c0beccb414b2c0 163 KLLKNNVDIVVTHDWPNNIEKHGDVHDLLRRKYHFQSDVYNNTLGNPHTEILLNKLKPYFWFASHLHVKYSALYIHN--- 239
Cdd:cd00844                             160 KQLKQPIDIFLSHDWPRGIYKHGDKKQLLRKKPFFRQDIESGTLGSPAAEELLKHLKPRYWFSAHLHVKFAALVPHEnks 239
                                                  250       260
                                            ....*....|....*....|...
seqsig_9acb7d07ed17bc4826c0beccb414b2c0 240 --DQKQYTRFLSLDKAQEYKHFI 260
Cdd:cd00844                             240 pgNTNKETKFLALDKCLPGRDFL 262
```

PF3D7_1355500 (TPR, PP5, TPR, TPR)

Cd Length: 100 **Bit Score:** 63.17 **E-value:** 4.15e-12

```
                                                   10        20        30        40        50        60        70        80
                                            ....*....|....*....|....*....|....*....|....*....|....*....|....*....|....*....|
seqsig_12e07dcb7afd2dae1b1ee42aca0f00e0 203 EYYNKsAISKKSDFISiketdlhiYYTNRSFCHIKLENYGTAIEDIDEAIKINPYYAKAYYRKGCSYLLLSDLKRASECF 282
Cdd:cd00189                              21 EYYEK-ALELDPDNAD--------AYYNLAAAYYKLGKYEEALEDYEKALELDPDNAKAYYNLGLAYYKLGKYEEALEAY 91

                                            ....*....
seqsig_12e07dcb7afd2dae1b1ee42aca0f00e0 283 QKVLKLTKD 291
Cdd:cd00189                              92 EKALELDPN 100
```

Cd Length: 316 **Bit Score:** 476.36 **E-value:** 3.58e-164

```
                                                   10        20        30        40        50        60        70        80
                                            ....*....|....*....|....*....|....*....|....*....|....*....|....*....|....*....|
seqsig_12e07dcb7afd2dae1b1ee42aca0f00e0 337 EAPIYDRNNLNLDFLKKVADYISIPNnKLNKKCVCAIVLDVIKLLKELPTLVYLNLEEDETLTICGDVHGQYYDLLNIMK 416
Cdd:cd07417                               2 DGPRLEDEKVTLEFVKEMIEWFKDQK-KLHKKYAYQILLQVKELLKKLPSLVEITIPEGEKITVCGDTHGQFYDLLNIFE 80
                                                   90       100       110       120       130       140       150       160
                                            ....*....|....*....|....*....|....*....|....*....|....*....|....*....|....*....|
seqsig_12e07dcb7afd2dae1b1ee42aca0f00e0 417 INGYPSEKNSYLFNGDFVDRGSFSVEVIIFLYLAKLTFPNNVYLTRGNHETDNMNKIYGFLGELQEKYDEKMHVLFSDSF 496
Cdd:cd07417                              81 LNGLPSETNPYLFNGDFVDRGSFSVEVILTLFAFKLLYPNHFHLNRGNHETDNMNKMYGFEGEVKAKYNEQMFDLFSEVF 160
                                                  170       180       190       200       210       220       230       240
                                            ....*....|....*....|....*....|....*....|....*....|....*....|....*....|....*....|
seqsig_12e07dcb7afd2dae1b1ee42aca0f00e0 497 KFLPLAYVLNKNIFICHGGIPSKTDTTLEDIEKIDRNKEPLDEGVMTDLLWSDPNEEKGFKPSKRGIGFSFGTDITENFL 576
Cdd:cd07417                             161 NWLPLAHLINGKVLVVHGGLFSDDGVTLDDIRKIDRFRQPPDSGLMCELLWSDPQPQPGRSPSKRGVGCQFGPDVTKRFL 240
                                                  250       260       270       280       290       300       310
                                            ....*....|....*....|....*....|....*....|....*....|....*....|....*....|....*
seqsig_12e07dcb7afd2dae1b1ee42aca0f00e0 577 KINNLSLIIRSHEVRDEGYSLEQNGQLYTVFSAPNYCDIMKNKGAFLKFKGNSIKPECVTFTEVEHPNVPSLKYA 651
Cdd:cd07417                             241 EENNLEYIIRSHEVKDEGYEVEHDGKCITVFSAPNYCDQMGNKGAFIRITGSDLKPKFTQFEAVPHPNVKPMAYA 315
```

Cd Length: 78 **Bit Score:** 41.59 **E-value:** 5.55e-05

```
                                                   10        20        30        40        50        60        70
                                            ....*....|....*....|....*....|....*....|....*....|....*....|....*....|....*..
seqsig_12e07dcb7afd2dae1b1ee42aca0f00e0 222 TDLHIYYTNRSFCHIKLENYGTAIEDIDEAIKI-------NPYYAKAYYRKGCSYLLLSDLKRASECFQKVLKLTKD 291
Cdd:pfam13424                             2 PDLAAALNNLALVLRRLGDYDEALELLEKALELarelgedHPETARALNNLARLYLALGDYDEALEYLEKALALREA 78
```

Cd Length: 69 **Bit Score:** 35.37 **E-value:** 7.20e-03

```
                                                   10        20        30        40        50        60        70        80
                                            ....*....|....*....|....*....|....*....|....*....|....*....|....*....|....*....|
seqsig_12e07dcb7afd2dae1b1ee42aca0f00e0 135 DALKNIGNKYFKENNYIISLRYYTEAIDLikksfeqCPNNvtdidnenntnnlhddvevddedkelfkeyynksaiskks 214
Cdd:pfam13414                             4 EALKNLGNALFKLGDYDEAIEAYEKALEL-------DPDN---------------------------------------- 36
                                                   90       100       110       120
                                            ....*....|....*....|....*....|....*....|...
seqsig_12e07dcb7afd2dae1b1ee42aca0f00e0 215 dfisiketdlHIYYTNRSFCHIKL-ENYGTAIEDIDEAIKINP 256
Cdd:pfam13414                            37 ----------AEAYYNLALAYLKLgKDYEEALEDLEKALELDP 69
```

PF3D7_1403900 (CSTP1)

Cd Length: 262 **Bit Score:** 232.18 **E-value:** 1.01e-74

```
                                                   10        20        30        40        50        60        70        80
                                            ....*....|....*....|....*....|....*....|....*....|....*....|....*....|....*....|
seqsig_991433d80e292142dbde3430d127693a  20 EPFFFVLFGDIQYGMIRGN-----HGWYEERELLKSAIEKTNKL--KPPFVVALGDLTNKFPLDPIQTNQITDLKNDFKL 92
Cdd:cd07395                               3 GPFYFIQGADPQLGLIKKNlegggDEWDEEIKLTEQAVQAINKLnpKPKFVVVCGDLVNAMPGDELRERQVSDLKDVLSL 82
                                                   90       100       110       120       130       140       150       160
                                            ....*....|....*....|....*....|....*....|....*....|....*....|....*....|....*....|
seqsig_991433d80e292142dbde3430d127693a  93 LDKDIDLYVFCGNHDVGNKPSMEGMEYFEEQWGDSYYSFVYNNCAFIVLNSPILYDETHVKEMKEEQLKWLEKTLEKLHS 172
Cdd:cd07395                              83 LDPDIPLVCVCGNHDVGNTPTEESIKDYRDVFGDDYFSFWVGGVFFIVLNSQLFFDPSEVPELAQAQDVWLEEQLEIAKE 162
                                                  170       180       190       200       210       220       230       240
                                            ....*....|....*....|....*....|....*....|....*....|....*....|....*....|....*....|
seqsig_991433d80e292142dbde3430d127693a 173 LNVKHKFLLLHHALMYDDIYEgenigliygdkfhnysEKNEFHLKKEPRLFIYELMKKYKVTHVFCAHLHANrENDIDHN 252
Cdd:cd07395                             163 SDCKHVIVFQHIPWFLEDPDE----------------EDSYFNIPKSVRKPLLDKFKKAGVKAVFSGHYHRN-AGGRYGG 225
                                                  250       260       270
                                            ....*....|....*....|....*....|....*.
seqsig_991433d80e292142dbde3430d127693a 253 IKQITISAVGMQAKDDKSGIFIVQVTEDKVDYKYYP 288
Cdd:cd07395                             226 LEMVVTSAIGAQLGNDKSGLRIVKVTEDKIVHEYYS 261
```

PF3D7_1406700 (VPS29)

Cd Length: 178 **Bit Score:** 287.18 **E-value:** 3.89e-99

```
                                                   10        20        30        40        50        60        70        80
                                            ....*....|....*....|....*....|....*....|....*....|....*....|....*....|....*....|
```

```
seqsig_37dcd7f6709a3b27b863bfdaf12200a6  11 LVLLIGDFHSPIRNLGLPDCFKELLKTDKIKHVLCTGNVGCNENLELLKNIADSVHITKGDMDDNFDFPEDITLCIGDFK 90
Cdd:cd07394                               1 LVLVIGDLHIPHRASDLPAKFKKLLVPGKIQHVLCTGNLCSKETYDYLKTIAPDVHIVRGDFDENLNYPETKVITVGQFK 80
                                                    90       100       110       120       130       140       150       160
                                            ....*....|....*....|....*....|....*....|....*....|....*....|....*....|....*....|
seqsig_37dcd7f6709a3b27b863bfdaf12200a6  91 ISLIHGHQIIPWGDMNALLQWQKKYDSDIIISGHTHKNSIVQYEGKYFINPGSVTGAFQPWLSEPTPTFILMAVAKSNIV 170
Cdd:cd07394                              81 IGLIHGHQVVPWGDPDSLAALQRQLDVDILISGHTHKFEAFEHEGKFFINPGSATGAFSPLDPNVIPSFVLMDIQGSKVV 160
                                                   170
                                            ....*....|....*...
seqsig_37dcd7f6709a3b27b863bfdaf12200a6 171 LYVYEEKNGKTNVEMSEL 188
Cdd:cd07394                             161 TYVYQLIDGEVKVEKIEY 178
```

PF3D7_1414400 (PP1)

Cd Length: 293 **Bit Score:** 663.68 **E-value:** 0e+00

```
                                                    10        20        30        40        50        60        70        80
                                            ....*....|....*....|....*....|....*....|....*....|....*....|....*....|....*....|
seqsig_50f2729d4070f091f263cae24f333e3b   6 DIDNVISKLIEVRGTRPGKNVNLTENEIKILCLSSREIFLNQPILLELEAPIKICGDIHGQFYDLLRLFEYGGFPPDANY 85
Cdd:cd07414                               1 DIDSIIERLLEVRGSRPGKNVQLTEAEIRGLCLKSREIFLSQPILLELEAPLKICGDIHGQYYDLLRLFEYGGFPPESNY 80
                                                    90       100       110       120       130       140       150       160
                                            ....*....|....*....|....*....|....*....|....*....|....*....|....*....|....*....|
seqsig_50f2729d4070f091f263cae24f333e3b  86 LFLGDYVDRGKQSLETICLLLAYKIKYPENFFLLRGNHECASINRIYGFYDECKRRYSVKLWKTFIDCFNCLPVAAIIDE 165
Cdd:cd07414                              81 LFLGDYVDRGKQSLETICLLLAYKIKYPENFFLLRGNHECASINRIYGFYDECKRRYNIKLWKTFTDCFNCLPVAAIIDE 160
                                                   170       180       190       200       210       220       230       240
                                            ....*....|....*....|....*....|....*....|....*....|....*....|....*....|....*....|
seqsig_50f2729d4070f091f263cae24f333e3b 166 KIFCMHGGLSPELNNMEQIRKITRPTDVPDNGLLCDLLWSDPEKEINGWGENDRGVSFTFGQDVVHNFLRKHELDLICRA 245
Cdd:cd07414                             161 KIFCMHGGLSPDLQSMEQIRRIMRPTDVPDQGLLCDLLWSDPDKDVQGWGENDRGVSFTFGKDVVAKFLNKHDLDLICRA 240
                                                   250       260       270       280       290
                                            ....*....|....*....|....*....|....*....|....*....|...
seqsig_50f2729d4070f091f263cae24f333e3b 246 HQVVEDGYEFFAKRQLVTLFSAPNYCGEFDNAGAMMSVDETLMCSFQILKPVE 298
Cdd:cd07414                             241 HQVVEDGYEFFAKRQLVTLFSAPNYCGEFDNAGAMMSVDETLMCSFQILKPAE 293
```

PF3D7_1423300 (PP5, PP7, EFh, EFh)

Cd Length: 316 **Bit Score:** 266.04 **E-value:** 8.41e-81

```
                                                    10        20        30        40        50        60        70        80
                                            ....*....|....*....|....*....|....*....|....*....|....*....|....*....|....*....|
seqsig_10bcd44708039df9e56ab0942d5be2de 177 PRLKD-KIDRTFATEMFHFFLTSKEIILPYnlVHKVLIKTKKMLEEniKSSVINLDMSKkskDTKLIILGDVHGQLHDVL 255
Cdd:cd07417                               4 PRLEDeKVTLEFVKEMIEWFKDQKKLHKKY--AYQILLQVKELLKK--LPSLVEITIPE---GEKITVCGDTHGQFYDLL 76
                                                    90       100       110       120       130       140       150       160
                                            ....*....|....*....|....*....|....*....|....*....|....*....|....*....|....*....|
seqsig_10bcd44708039df9e56ab0942d5be2de 256 WLFNRFGIPSSTNIYIFNGDIADRGENATEIFILLFIFKLSCNDSVIINRGNHECSYMNEVYGFYNEVLSKYDNTIFDLF 335
Cdd:cd07417                              77 NIFELNGLPSETNPYLFNGDFVDRGSFSVEVILTLFAFKLLYPNHFHLNRGNHETDNMNKMYGFEGEVKAKYNEQMFDLF 156
                                                   170       180       190       200       210       220       230       240
                                            ....*....|....*....|....*....|....*....|....*....|....*....|....*....|....*....|
seqsig_10bcd44708039df9e56ab0942d5be2de 336 QNIFELLGLAVNVQNQIFVVHGGLSRYQDITLKEIDELDRKKQeilhPEqyEDIVIFDLLWSDPQKKEGIGGNARGNNCi 415
Cdd:cd07417                             157 SEVFNWLPLAHLINGKVLVVHGGLFSDDGVTLDDIRKIDRFRQ----PP--DSGLMCELLWSDPQPQPGRSPSKRGVGC- 229
                                                   250       260       270       280       290       300
                                            ....*....|....*....|....*....|....*....|....*....|....*....|....*
seqsig_10bcd44708039df9e56ab0942d5be2de 416 TFGPDVTEMFLKNNNLDILIRSHQVPKtlKGIESHHEGKCITLFSASNYCNKIKNLGAAIIFNQD 480
Cdd:cd07417                             230 QFGPDVTKRFLEENNLEYIIRSHEVKD--EGYEVEHDGKCITVFSAPNYCDQMGNKGAFIRITGS 292
```

Cd Length: 377 **Bit Score:** 183.85 **E-value:** 8.10e-51

```
                                                    10        20        30        40        50        60        70        80
                                            ....*....|....*....|....*....|....*....|....*....|....*....|....*....|....*....|
seqsig_10bcd44708039df9e56ab0942d5be2de 202 ILPYNLVHKVLIKTKKMLEEniKSSVINLDMSKKSKdtkLIILGDVHGQLHDVLWLFNRFGIPSSTNIYIFNGDIADRGE 281
Cdd:cd07418                              34 VLPVNVFDSLVLTAHKILHR--EPNCVRIDVEDVCE---VVVVGDVHGQLHDVLFLLEDAGFPDQNRFYVFNGDYVDRGA 108
                                                    90       100       110       120       130       140       150       160
                                            ....*....|....*....|....*....|....*....|....*....|....*....|....*....|....*....|
seqsig_10bcd44708039df9e56ab0942d5be2de 282 NATEIFILLFIFKLSCNDSVIINRGNHECSYMNEVYGFYNEVLSKYDNT---IFDLFQNIFELLGLAVNVQNQIFVVHGG 358
Cdd:cd07418                             109 WGLETFLLLLSWKVLLPDRVYLLRGNHESKFCTSMYGFEQEVLTKYGDKgkhVYRKCLGCFEGLPLASIIAGRVYTAHGG 188
                                                   170       180       190       200       210       220       230       240
                                            ....*....|....*....|....*....|....*....|....*....|....*....|....*....|....*....|
seqsig_10bcd44708039df9e56ab0942d5be2de 359 L-----------------------SRYQDITLKEIDELDRKKQEILHPEQYEDIVI-FDLLWSDPQKKEGIGGNARGNNC 414
Cdd:cd07418                             189 LfrspslpkrkkqkgknrrvllllePESESLKLGTLDDLMKARRSVLDPPGEGSNLIpGDVLWSDPSLTPGLSPNKQRGIG 268
                                                   250       260       270       280       290       300       310       320
                                            ....*....|....*....|....*....|....*....|....*....|....*....|....*....|....*....|
seqsig_10bcd44708039df9e56ab0942d5be2de 415 ITFGPDVTEMFLKNNNLDILIRSHQVPKT----------LKGIESHHE---GKCITLFSASNYC------NKIKNLGAAI 475
Cdd:cd07418                             269 LLWGPDCTEEFLEKNNLKLIIRSHEGPDArekrpglagmNKGYTVDHDvesGKLITLFSAPDYPqfqateERYNNKGAYI 348
                                                   330       340
                                            ....*....|....*....|
seqsig_10bcd44708039df9e56ab0942d5be2de 476 IFN----QDLTFEVQEYMSP 491
Cdd:cd07418                             349 ILQppdfSDPQFHTFEAVKP 368
```

Cd Length: 63 **Bit Score:** 37.53 **E-value:** 1.76e-03

```
                                                    10        20        30        40        50        60
                                            ....*....|....*....|....*....|....*....|....*....|....*....|....*..
seqsig_10bcd44708039df9e56ab0942d5be2de 647 LRETLMVFDKNLDGKVSFAEFEQVLRDLNIDLSNEQIRILVRLINSNslcnntnlqENDKIDVAEFI 713
Cdd:cd00051                               2 LREAFRLFDKDGDGTISADELKAALKSLGEGLSEEEIDEMIREVDKD---------GDGKIDFEEFL 59
```

Cd Length: 60 **Bit Score:** 44.66 **E-value:** 8.73e-06

```
                                                    10        20        30        40        50        60
                                            ....*....|....*....|....*....|....*....|....*....|....*....|....*
seqsig_10bcd44708039df9e56ab0942d5be2de 788 KNYDNFGNGYLDYNDFVKAIKNFDMNKISKEVEFEVDddilmELAKSIDITKSSKINFLEFLQAF 852
Cdd:pfam13499                             1 KLLDKDGDGYIDVEELRKLLKALGLKLTDEEVEELIE-----ADFNEIDKDGDGRISFEEFLEAM 60
```

PF3D7_1464600 (ACP5, Pox_Ag35)

Cd Length: 277 **Bit Score:** 117.02 **E-value:** 2.15e-28

```
                                                    10        20        30        40        50        60        70        80
                                            ....*....|....*....|....*....|....*....|....*....|....*....|....*....|....*....|
seqsig_68ce03ae104ba33302ba77bd3b5d16de 766 IPFYSVLGEKDYFYFPSEQIQehYSKRIPG--YFMPNNYYCVNYDFTynpvkknvngdeddddeeeeegdsGTDRKVKt 843
Cdd:cd07378                              71 VPWYLVLGNHDYSGNVSAQID--YTKRPNSprWTMPAYYYRVSFPFP------------------------SSDTTVE- 122
```

```
                                                     90       100       110       120       130       140       150       160
                                             ....*....|....*....|....*....|....*....|....*....|....*....|....*....|....*....|
seqsig_68ce03ae104ba33302ba77bd3b5d16de  844 qekfrasFIFIDTWALMVGFPII------RNYRAFREQFNWLSKTLyeSAKKSDWIFVVGHHPLISSGRRSDNysyeeHS 917
Cdd:cd07378                              123 -------FIMIDTVPLCGNSDDIaspygpPNGKLAEEQLAWLEKTL--AASTADWKIVVGHHPIYSSGEHGPT-----SC 188
                                                    170       180       190       200       210       220       230       240
                                             ....*....|....*....|....*....|....*....|....*....|....*....|....*....|....*....|
seqsig_68ce03ae104ba33302ba77bd3b5d16de  918 FHDIIRDFLFNYHVDAYFSAHDHLMEYIKFG--SVDLFINGSSSRVLfdnssmgrgyfgkiigklyplscyvLKTIHTGL 995
Cdd:cd07378                              189 LVDRLLPLLKKYKVDAYLSGHDHNLQHIKDDgsGTSFVVSGAGSKAR------------------------PSVKHIDK 243
                                                    250       260       270
                                             ....*....|....*....|....*....|....*....
seqsig_68ce03ae104ba33302ba77bd3b5d16de  996 KPKGCNinryskWYNKSDIGFSTHKLTKDELVTQFISSR 1034
Cdd:cd07378                              244 VPQFFS------GFTSSGGGFAYLELTKEELTVRFYDAD 276
```

Cd Length: 198 **Bit Score:** 39.36 **E-value:** 2.79e-03

```
                                                     10        20        30        40        50        60        70        80
                                             ....*....|....*....|....*....|....*....|....*....|....*....|....*....|....*....|
seqsig_68ce03ae104ba33302ba77bd3b5d16de 1244 KKKKEKEKNGNEQEEAEEETEVVEEidelkemekkrkESEGDITGEDENEIKEEQKDNEKEDETYEEYLDESqyndeEIP 1323
Cdd:pfam03286                             68 SKKKDKEKLTEEEKKPESDDDKTEE------------NENDPDNNEESGDSQESASANSLSDIDNEDDMDDS-----DLK 130
                                                     90       100
                                             ....*....|....*....|.
seqsig_68ce03ae104ba33302ba77bd3b5d16de 1324 LV-KQVHKDFKKLANQEKKLS 1343
Cdd:pfam03286                            131 LAtENIIKDLKKLNARVSAVS 151
```

PF3D7_1466100 (Bsu1, Kelch, Kelch, Kelch, Kelch)

Cd Length: 311 **Bit Score:** 586.68 **E-value:** 0e+00

```
                                 10        20        30        40        50        60        70        80
                         ....*....|....*....|....*....|....*....|....*....|....*....|....*....|....*....|
lcl|local_MNNGSFKETS 522 IITTLLNPNITQfeiqynHNSESIFIIPWANISVLCSIVIDIFKQEDMVLKLRAPIKIYGDIHGQYYDLMRMFQLYKCPV 601
Cdd:cd07419            1 IITHLLKPRIWK------PPTDRRFFFNWNEILELCDAAEDIFKQEPMVLRLRAPIKIFGDIHGQFGDLMRLFDEYGSPV 74
                                 90       100       110       120       130       140       150       160
                         ....*....|....*....|....*....|....*....|....*....|....*....|....*....|....*....|
lcl|local_MNNGSFKETS 602 EEdlgeklnAIGDIDSNDYLFLGDYVDRGSNSLEVICLLFALKCKYPKQIHLIRGNHEDVAINSLYGFQEECKRRLKEDV 681
Cdd:cd07419           75 TE-------AAGDIEYIDYLFLGDYVDRGSNSLETICLLLALKVKYPNQIHLIRGNHEDRDINALFGFREECKERLGEDP 147
                                170       180       190       200       210       220       230       240
                         ....*....|....*....|....*....|....*....|....*....|....*....|....*....|....*....|
lcl|local_MNNGSFKETS 682 TDKDSCWYQINQVFEWLPIGAIVEDKILCVHGGIGKSINQISDISQLKRPLvvsqvPQNLNEQKVTDLLWSDPTDNDSIL 761
Cdd:cd07419          148 NDGDSVWRRINRLFEWLPLAAIIEDKILCMHGGIGRSINHVSEIEDLKRPL-----TMEFGEQVVMDLLWSDPTENDSVL 222
                                250       260       270       280       290       300       310       320
                         ....*....|....*....|....*....|....*....|....*....|....*....|....*....|....*....|
lcl|local_MNNGSFKETS 762 GTIPNDIrDPDGTGHIVKYGPDRVHKFLEENDLQLIIRAHECVMDGFERFAGGKLITLFSATNYCNSHKNAGALLFIRRD 841
Cdd:cd07419          223 GLRPNAI-DPRGPGLIVKFGPDRVHRFLEENDLQMIIRAHECVMDGFERFAQGKLITLFSATNYCGTAGNAGAILVLGRD 301
                                330
                         ....*....|
lcl|local_MNNGSFKETS 842 LTVIPKLIYP 851
Cdd:cd07419          302 LTIIPKLIHP 311
```

Cd Length: 48 **Bit Score:** 44.59 **E-value:** 6.40e-06

```
                                 10        20        30        40        50
                         ....*....|....*....|....*....|....*....|....*....|...
lcl|local_MNNGSFKETS  35 NNKVAIFGGAIGDAGKYNitdDIYLYDLTQNKWKKLitENTPSARAAHAAACV 87
Cdd:pfam13415          1 GGKIYVFGGLGDGGTRLN---DLWVYDLDTNTWEKL--GDLPGPRAGHAATVI 48
```

Cd Length: 42 **Bit Score:** 43.75 **E-value:** 1.37e-05

```
                                 10        20        30        40
                         ....*....|....*....|....*....|....*....|.
lcl|local_MNNGSFKETS  75 TPSARAAHAAaCVDEQQLVIYGGATGGGSLSLDDLYILDLR 115
Cdd:pfam13854          1 LPVPRANHCA-VVVGGEIYLYGGYTSGNGQSSDDVYVLSLP 40
```

Cd Length: 48 **Bit Score:** 43.13 **E-value:** 2.13e-05

```
                                 10        20        30        40
                         ....*....|....*....|....*....|....*....|....*.
lcl|local_MNNGSFKETS 249 RYQHTSVFIGSKIFILGGRNDNGCAVPLSTALYNTETIEWVTLPSI 294
Cdd:pfam07646          2 RYPHASVVVGGKLYVVGGSTGLGDLSSSDLWVLDPETNVWTELPAL 47
```

Cd Length: 49 **Bit Score:** 56.11 **E-value:** 6.86e-10

```
                                 10        20        30        40
                         ....*....|....*....|....*....|....*....|....*..
lcl|local_MNNGSFKETS  24 PRFGHTATYLGNNKVAIFGGAIGDAGKYNitdDIYLYDLTQNKWKKL 70
Cdd:pfam13418          1 PRAYHTSTSIGDGRLYLFGGENEDGSVLS---DVWVFDLSTNTWTRL 44
```
