# Supplementary material for: Genome wide in silico analysis of Plasmodium falciparum phosphatome
Source: BMC Genomics. 2014 Nov 25;15:1024. doi: 10.1186/1471-2164-15-1024 (PMC4256932; doi:10.1186/1471-2164-15-1024)
Supplement: Supplementary file 6 — Additional file 6:Parameters used in various software and tools.(DOCX 15 KB) [file 12864_2014_6717_MOESM6_ESM.docx]

**Parameters Used in various tools and software’s**

**Sequence Retrieval**

**PlasmoDB version 9.2**: Text search “Phosphatase”.

Field:- Alias, EC descriptions, Gene ID, Gene notes, Genes of previous release, Gene product, GO terms and definitions, Metabolic pathway names and descriptions, Protein domain names and descriptions, Rodent Malaria Phenotype, Similar proteins, User comments.

Organsims:- All

**EuPathDB**: Text search “Phosphatase”.

Field:- Alias, EC descriptions, Gene ID, Gene notes, Genes of previous release, Gene product, GO terms and definitions, Metabolic pathway names and descriptions, Protein domain names and descriptions, Rodent Malaria Phenotype, Similar proteins, User comments.

Organisms: *Toxoplasma gondii* (ToxoDB 7.3)*,* [*Eimeria tenella*](javascript:void(0)) (ToxoDB 7.3)*,* [*Cryptosporidium parvum*](javascript:void(0)) (CryptoDB 6.0)*,* [*Theileria parva*](javascript:void(0)) (PiroplasmaDB 5.0)*,* [*Babesia bovis*](javascript:void(0)) (PiroplasmaDB 5.0)*.*

**Uniprot:** Text Search “Phosphatase AND organism name AND referenced AND reviewed” against Uniprot Database on Nov 2012.

**Annotation**

**Conserved Domain Database (CDD)**

Search Method = CDD, PFAM, SMART, TIGRFAM, COG, PRK

Threshold e-value = 0.01

*only superfamily domain results are selected.

NCBI pBLAST = Default parameters, Database used- non redundant database, Protein Data Bank proteins

**Multiple sequence alignment**

**Clustal X1 2.1**

- Pairwise alignment:- Gap opening Penalty = 10, Gap extension penalty = 0.1,

Protein weight matrix = Gonnet 250

- Multiple alignment:- Gap opening Penalty = 10, Gap extension penalty = 0.2,

Delay divergent sequences = 30%, Negative Matrix = Off, Protein weight matrix = Gonnet series

- Protein gap parameters:- Residue specific penalties = On, Hydrophilic penalties = On,

Hydrophilic residues = GPSNDQEKP, Gap separation distance = 4, End gap separation = Off,

Iterations = Off.

**Muscle (Incorporated with Mega 5.2.1)**

- Gap penalties:- Gap opening Penalty = -2.9, Gap extension penalty = 0,

Hydrophobicity multiplier = 1.2.

- Memory used/Iterations:- Maximum memory (in MB) = 2124, Maximum iterations = 8
- Advanced options:- 1^st^ Clustering method (1.2) = UPGMB, 2^nd^ Clustering Method = UPGMB,

Minimum diagonal length (ƛ) = 24.

**Phylogenetic Tree**

**Mega 5.2.1**

- Method: - Neighbor Joining
- Bootstrap value = 1000
- Substitution model = Jones-Taylor-Thornton (JTT)
- Substitution type = Amino acid,
- Pattern among lineage = Uniform rate and Same (Homogenous)
- Gap/missing data treatment = Partial deletion (site coverage cutoff 95%)

**Interacting Partner Prediction**

**String Database Parameters**

Prediction Method: - Neighborhood, Gene Fusion, Co-expression, Co-occurrence, Experiment, Database Search, Text-mining

Medium confidence for interacting partner selection = 0.40

PlasmoMAP: Complete 3D7 strain data.

**Ortholog Analysis**

OrthoMCL Database: Default parameters assigned by database developers.
