# Supplementary material for: Genome wide in silico analysis of Plasmodium falciparum phosphatome
Source: BMC Genomics. 2014 Nov 25;15:1024. doi: 10.1186/1471-2164-15-1024 (PMC4256932; doi:10.1186/1471-2164-15-1024)
Supplement: Supplementary file 7 — Additional file 7:STRING prediction of interacting partners for P. falciparum phosphatases.(DOCX 570 KB) [file 12864_2014_6717_MOESM7_ESM.docx]

| | 1. **Your Input:** **PF3D7_0303200** | | | 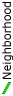 | 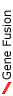 | 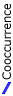 | 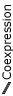 | 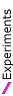 | 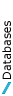 | 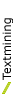 | 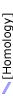 | 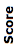 | | --- | --- | --- | --- | --- | --- | --- | --- | --- | --- | --- | --- | | 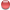 | [PFC0150w](http://string-db.org/newstring_cgi/display_single_node.pl?taskId=xQcYbwASRS74&node=400487&targetmode=proteins) | hypothetical protein, conserved (1171 aa) | |  |  | *(Plasmodium falciparum)* | | **Predicted Functional Partners:** | | | | 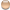 | [PKG](http://string-db.org/newstring_cgi/display_single_node.pl?taskId=xQcYbwASRS74&node=399622&targetmode=proteins) | cGMP-dependent protein kinase 1, beta isozyme, putative (853 aa) |  |  |  | [**•**](http://string-db.org/newstring_cgi/show_coexpression_evidence.pl?taskId=xQcYbwASRS74&node2=399622) |  |  |  |  | 0.958 | | 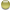 | [PF14_0681](http://string-db.org/newstring_cgi/display_single_node.pl?taskId=xQcYbwASRS74&node=399966&targetmode=proteins) | diacylglycerol kinase, putative (488 aa) |  |  |  | [**•**](http://string-db.org/newstring_cgi/show_coexpression_evidence.pl?taskId=xQcYbwASRS74&node2=399966) |  |  |  |  | 0.948 | | 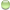 | [MAL8P1.153-1](http://string-db.org/newstring_cgi/display_single_node.pl?taskId=xQcYbwASRS74&node=397576&targetmode=proteins) | hypothetical protein, conserved (2577 aa) |  |  |  | [**•**](http://string-db.org/newstring_cgi/show_coexpression_evidence.pl?taskId=xQcYbwASRS74&node2=397576) |  |  |  |  | 0.917 | | 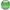 | [PFC0995c](http://string-db.org/newstring_cgi/display_single_node.pl?taskId=xQcYbwASRS74&node=400674&targetmode=proteins) | diacylglycerol O-acyltransferase, putative (653 aa) |  |  |  |  |  | [**•**](http://string-db.org/newstring_cgi/show_set_evidence.pl?data_channel=database&taskId=xQcYbwASRS74&node2=400674) |  |  | 0.899 | | 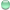 | [PFL1565c](http://string-db.org/newstring_cgi/display_single_node.pl?taskId=xQcYbwASRS74&node=402305&targetmode=proteins) | hypothetical protein, conserved (959 aa) |  |  |  | [**•**](http://string-db.org/newstring_cgi/show_coexpression_evidence.pl?taskId=xQcYbwASRS74&node2=402305) |  |  |  |  | 0.863 | | 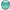 | [RhopH2](http://string-db.org/newstring_cgi/display_single_node.pl?taskId=xQcYbwASRS74&node=401903&targetmode=proteins) | High molecular weight rhoptry protein-2 (1378 aa) |  |  |  | [**•**](http://string-db.org/newstring_cgi/show_coexpression_evidence.pl?taskId=xQcYbwASRS74&node2=401903) |  |  |  |  | 0.861 | | 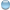 | [PFE1130w](http://string-db.org/newstring_cgi/display_single_node.pl?taskId=xQcYbwASRS74&node=401185&targetmode=proteins) | hypothetical protein, conserved (483 aa) |  |  |  | [**•**](http://string-db.org/newstring_cgi/show_coexpression_evidence.pl?taskId=xQcYbwASRS74&node2=401185) |  |  |  |  | 0.861 | | 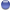 | [PFE0440w](http://string-db.org/newstring_cgi/display_single_node.pl?taskId=xQcYbwASRS74&node=401047&targetmode=proteins) | hypothetical protein, conserved (3134 aa) |  |  |  | [**•**](http://string-db.org/newstring_cgi/show_coexpression_evidence.pl?taskId=xQcYbwASRS74&node2=401047) |  |  |  | **•** | 0.861 | | 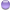 | [PF13_0086](http://string-db.org/newstring_cgi/display_single_node.pl?taskId=xQcYbwASRS74&node=399009&targetmode=proteins) | hypothetical protein, conserved (477 aa) |  |  |  | [**•**](http://string-db.org/newstring_cgi/show_coexpression_evidence.pl?taskId=xQcYbwASRS74&node2=399009) |  |  |  |  | 0.861 | | 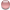 | [PF10_0342](http://string-db.org/newstring_cgi/display_single_node.pl?taskId=xQcYbwASRS74&node=398338&targetmode=proteins) | hypothetical protein (561 aa) |  |  |  | [**•**](http://string-db.org/newstring_cgi/show_coexpression_evidence.pl?taskId=xQcYbwASRS74&node2=398338) |  |  |  |  | 0.861 | |
| --- | --- | --- | --- | --- | --- | --- | --- | --- | --- | --- | --- | --- | --- | --- | --- | --- | --- | --- | --- | --- | --- | --- | --- | --- | --- | --- | --- | --- | --- | --- | --- | --- | --- | --- | --- | --- | --- | --- | --- | --- | --- | --- | --- | --- | --- | --- | --- | --- | --- | --- | --- | --- | --- | --- | --- | --- | --- | --- | --- | --- | --- | --- | --- | --- | --- | --- | --- | --- | --- | --- | --- | --- | --- | --- | --- | --- | --- | --- | --- | --- | --- | --- | --- | --- | --- | --- | --- | --- | --- | --- | --- | --- | --- | --- | --- | --- | --- | --- | --- | --- | --- | --- | --- | --- | --- | --- | --- | --- | --- | --- | --- | --- | --- | --- | --- | --- | --- | --- | --- | --- | --- | --- | --- | --- | --- | --- | --- | --- | --- | --- | --- | --- | --- | --- | --- | --- | --- | --- | --- | --- | --- |

2.

| | | | **Your Input:** PF3D7_0305600 | | | 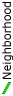 | 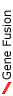 | 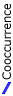 | 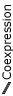 | 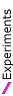 | 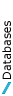 | 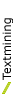 | 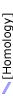 | 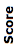 | | --- | --- | --- | --- | --- | --- | --- | --- | --- | --- | --- | --- | | 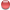 | [PFC0250c](http://string-db.org/newstring_cgi/display_single_node.pl?taskId=QRTmmL2Vg7P7&node=400512&targetmode=proteins) | AP endonuclease (DNA-(apurinic or apyrimidinic site) lyase), putative (617 aa) | |  |  | *(Plasmodium falciparum)* | | **Predicted Functional Partners:** | | | | 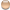 | [PFF0670w-1](http://string-db.org/newstring_cgi/display_single_node.pl?taskId=QRTmmL2Vg7P7&node=401418&targetmode=proteins) | hypothetical protein, conserved (4095 aa) |  |  |  | [**•**](http://string-db.org/newstring_cgi/show_coexpression_evidence.pl?taskId=QRTmmL2Vg7P7&node2=401418) |  |  |  |  | 0.960 | | 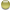 | [PFA_0285c](http://string-db.org/newstring_cgi/display_single_node.pl?taskId=QRTmmL2Vg7P7&node=400130&targetmode=proteins) | hypothetical protein, conserved (832 aa) |  |  |  | [**•**](http://string-db.org/newstring_cgi/show_coexpression_evidence.pl?taskId=QRTmmL2Vg7P7&node2=400130) |  |  |  |  | 0.960 | | 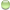 | [PF14_0533-1](http://string-db.org/newstring_cgi/display_single_node.pl?taskId=QRTmmL2Vg7P7&node=399812&targetmode=proteins) | hypothetical protein (1374 aa) |  |  |  | [**•**](http://string-db.org/newstring_cgi/show_coexpression_evidence.pl?taskId=QRTmmL2Vg7P7&node2=399812) |  |  |  |  | 0.960 | | 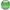 | [Pf92](http://string-db.org/newstring_cgi/display_single_node.pl?taskId=QRTmmL2Vg7P7&node=399245&targetmode=proteins) | cysteine-rich surface protein (796 aa) |  |  |  | [**•**](http://string-db.org/newstring_cgi/show_coexpression_evidence.pl?taskId=QRTmmL2Vg7P7&node2=399245) |  |  |  |  | 0.960 | | 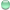 | [PF13_0327](http://string-db.org/newstring_cgi/display_single_node.pl?taskId=QRTmmL2Vg7P7&node=399235&targetmode=proteins) | cytochrome c oxidase subunit 2, putative; Cytochrome c oxidase is the component of the respirat [...] (228 aa) |  |  |  | [**•**](http://string-db.org/newstring_cgi/show_coexpression_evidence.pl?taskId=QRTmmL2Vg7P7&node2=399235) |  |  |  |  | 0.960 | | 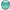 | [MAL13P1.215-1](http://string-db.org/newstring_cgi/display_single_node.pl?taskId=QRTmmL2Vg7P7&node=397084&targetmode=proteins) | hypothetical protein, conserved (315 aa) |  |  |  | [**•**](http://string-db.org/newstring_cgi/show_coexpression_evidence.pl?taskId=QRTmmL2Vg7P7&node2=397084) |  |  |  |  | 0.948 | | 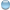 | [PFI0160w](http://string-db.org/newstring_cgi/display_single_node.pl?taskId=QRTmmL2Vg7P7&node=401641&targetmode=proteins) | hypothetical protein, conserved (3135 aa) |  |  |  | [**•**](http://string-db.org/newstring_cgi/show_coexpression_evidence.pl?taskId=QRTmmL2Vg7P7&node2=401641) |  |  |  |  | 0.935 | | 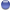 | [cyc-2](http://string-db.org/newstring_cgi/display_single_node.pl?taskId=QRTmmL2Vg7P7&node=402256&targetmode=proteins) | hypothetical protein, conserved (2281 aa) |  |  |  | [**•**](http://string-db.org/newstring_cgi/show_coexpression_evidence.pl?taskId=QRTmmL2Vg7P7&node2=402256) |  |  |  |  | 0.917 | | 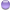 | [PFE0235c](http://string-db.org/newstring_cgi/display_single_node.pl?taskId=QRTmmL2Vg7P7&node=401005&targetmode=proteins) | hypothetical protein, conserved (5639 aa) |  |  |  | [**•**](http://string-db.org/newstring_cgi/show_coexpression_evidence.pl?taskId=QRTmmL2Vg7P7&node2=401005) |  |  |  |  | 0.913 | | 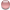 | [PF13_0095](http://string-db.org/newstring_cgi/display_single_node.pl?taskId=QRTmmL2Vg7P7&node=399017&targetmode=proteins) | DNA replication licensing factor mcm4-related (1005 aa) |  |  |  | [**•**](http://string-db.org/newstring_cgi/show_coexpression_evidence.pl?taskId=QRTmmL2Vg7P7&node2=399017) |  |  |  |  | 0.911 | | 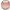 | [PF11_0386](http://string-db.org/newstring_cgi/display_single_node.pl?taskId=QRTmmL2Vg7P7&node=398777&targetmode=proteins) | 30S ribosomal protein S14, putative (172 aa) |  |  |  | [**•**](http://string-db.org/newstring_cgi/show_coexpression_evidence.pl?taskId=QRTmmL2Vg7P7&node2=398777) |  |  |  |  | 0.901 | | 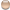 | [PFF0720w](http://string-db.org/newstring_cgi/display_single_node.pl?taskId=QRTmmL2Vg7P7&node=401429&targetmode=proteins) | hypothetical membrane protein, conserved (1096 aa) |  |  |  | [**•**](http://string-db.org/newstring_cgi/show_coexpression_evidence.pl?taskId=QRTmmL2Vg7P7&node2=401429) |  |  |  |  | 0.897 | | 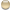 | [PfPDI-9](http://string-db.org/newstring_cgi/display_single_node.pl?taskId=QRTmmL2Vg7P7&node=401802&targetmode=proteins) | protein disulfide isomerase, putative (515 aa) |  |  |  | [**•**](http://string-db.org/newstring_cgi/show_coexpression_evidence.pl?taskId=QRTmmL2Vg7P7&node2=401802) |  |  |  |  | 0.863 | | 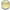 | [PFI0565w](http://string-db.org/newstring_cgi/display_single_node.pl?taskId=QRTmmL2Vg7P7&node=401724&targetmode=proteins) | hypothetical protein, conserved (474 aa) |  |  |  | [**•**](http://string-db.org/newstring_cgi/show_coexpression_evidence.pl?taskId=QRTmmL2Vg7P7&node2=401724) |  |  |  |  | 0.861 | | 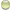 | [PF13_0160](http://string-db.org/newstring_cgi/display_single_node.pl?taskId=QRTmmL2Vg7P7&node=399075&targetmode=proteins) | hypothetical protein, conserved (302 aa) |  |  |  | [**•**](http://string-db.org/newstring_cgi/show_coexpression_evidence.pl?taskId=QRTmmL2Vg7P7&node2=399075) |  |  |  |  | 0.861 | | 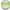 | [PFL0350c](http://string-db.org/newstring_cgi/display_single_node.pl?taskId=QRTmmL2Vg7P7&node=402055&targetmode=proteins) | hypothetical protein, conserved (2612 aa) |  |  |  | [**•**](http://string-db.org/newstring_cgi/show_coexpression_evidence.pl?taskId=QRTmmL2Vg7P7&node2=402055) |  |  |  | **•** | 0.861 | | 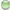 | [PFF1470c](http://string-db.org/newstring_cgi/display_single_node.pl?taskId=QRTmmL2Vg7P7&node=401582&targetmode=proteins) | DNA polymerase epsilon, catalytic subunit a, putative (2907 aa) |  |  |  | [**•**](http://string-db.org/newstring_cgi/show_coexpression_evidence.pl?taskId=QRTmmL2Vg7P7&node2=401582) |  |  |  |  | 0.861 | | 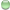 | [R17](http://string-db.org/newstring_cgi/display_single_node.pl?taskId=QRTmmL2Vg7P7&node=396970&targetmode=proteins) | hypothetical protein, conserved (1070 aa) |  |  |  | [**•**](http://string-db.org/newstring_cgi/show_coexpression_evidence.pl?taskId=QRTmmL2Vg7P7&node2=396970) |  |  |  |  | 0.861 | | 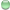 | [PFE1515w](http://string-db.org/newstring_cgi/display_single_node.pl?taskId=QRTmmL2Vg7P7&node=401261&targetmode=proteins) | hypothetical protein, conserved (1419 aa) |  |  |  | [**•**](http://string-db.org/newstring_cgi/show_coexpression_evidence.pl?taskId=QRTmmL2Vg7P7&node2=401261) |  |  |  | **•** | 0.859 | | 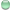 | [PFC0860w](http://string-db.org/newstring_cgi/display_single_node.pl?taskId=QRTmmL2Vg7P7&node=400645&targetmode=proteins) | kinesin, putative (1200 aa) |  |  |  | [**•**](http://string-db.org/newstring_cgi/show_coexpression_evidence.pl?taskId=QRTmmL2Vg7P7&node2=400645) |  |  |  | **•** | 0.859 | | | --- | --- | --- | --- | --- | --- | --- | --- | --- | --- | --- | --- | --- | --- | --- | --- | --- | --- | --- | --- | --- | --- | --- | --- | --- | --- | --- | --- | --- | --- | --- | --- | --- | --- | --- | --- | --- | --- | --- | --- | --- | --- | --- | --- | --- | --- | --- | --- | --- | --- | --- | --- | --- | --- | --- | --- | --- | --- | --- | --- | --- | --- | --- | --- | --- | --- | --- | --- | --- | --- | --- | --- | --- | --- | --- | --- | --- | --- | --- | --- | --- | --- | --- | --- | --- | --- | --- | --- | --- | --- | --- | --- | --- | --- | --- | --- | --- | --- | --- | --- | --- | --- | --- | --- | --- | --- | --- | --- | --- | --- | --- | --- | --- | --- | --- | --- | --- | --- | --- | --- | --- | --- | --- | --- | --- | --- | --- | --- | --- | --- | --- | --- | --- | --- | --- | --- | --- | --- | --- | --- | --- | --- | --- | --- | --- | --- | --- | --- | --- | --- | --- | --- | --- | --- | --- | --- | --- | --- | --- | --- | --- | --- | --- | --- | --- | --- | --- | --- | --- | --- | --- | --- | --- | --- | --- | --- | --- | --- | --- | --- | --- | --- | --- | --- | --- | --- | --- | --- | --- | --- | --- | --- | --- | --- | --- | --- | --- | --- | --- | --- | --- | --- | --- | --- | --- | --- | --- | --- | --- | --- | --- | --- | --- | --- | --- | --- | --- | --- | --- | --- | --- | --- | --- | --- | --- | --- | --- | --- | --- | --- | --- | --- | --- | --- | --- | --- | --- | --- | --- | --- | --- | --- | --- | --- | --- | --- | --- | --- | --- | --- | --- | --- | --- | --- | --- | --- | --- | --- | --- | --- | --- | --- | | | --- | --- | --- | --- | --- | --- | --- | --- | --- | --- | --- | --- | --- | --- | --- | --- | --- | --- | --- | --- | --- | --- | --- | --- | --- | --- | --- | --- | --- | --- | --- | --- | --- | --- | --- | --- | --- | --- | --- | --- | --- | --- | --- | --- | --- | --- | --- | --- | --- | --- | --- | --- | --- | --- | --- | --- | --- | --- | --- | --- | --- | --- | --- | --- | --- | --- | --- | --- | --- | --- | --- | --- | --- | --- | --- | --- | --- | --- | --- | --- | --- | --- | --- | --- | --- | --- | --- | --- | --- | --- | --- | --- | --- | --- | --- | --- | --- | --- | --- | --- | --- | --- | --- | --- | --- | --- | --- | --- | --- | --- | --- | --- | --- | --- | --- | --- | --- | --- | --- | --- | --- | --- | --- | --- | --- | --- | --- | --- | --- | --- | --- | --- | --- | --- | --- | --- | --- | --- | --- | --- | --- | --- | --- | --- | --- | --- | --- | --- | --- | --- | --- | --- | --- | --- | --- | --- | --- | --- | --- | --- | --- | --- | --- | --- | --- | --- | --- | --- | --- | --- | --- | --- | --- | --- | --- | --- | --- | --- | --- | --- | --- | --- | --- | --- | --- | --- | --- | --- | --- | --- | --- | --- | --- | --- | --- | --- | --- | --- | --- | --- | --- | --- | --- | --- | --- | --- | --- | --- | --- | --- | --- | --- | --- | --- | --- | --- | --- | --- | --- | --- | --- | --- | --- | --- | --- | --- | --- | --- | --- | --- | --- | --- | --- | --- | --- | --- | --- | --- | --- | --- | --- | --- | --- | --- | --- | --- | --- | --- | --- | --- | --- | --- | --- | --- | --- | --- | --- | --- | --- | --- | --- | --- | --- | |
| --- | --- | --- | --- | --- | --- | --- | --- | --- | --- | --- | --- | --- | --- | --- | --- | --- | --- | --- | --- | --- | --- | --- | --- | --- | --- | --- | --- | --- | --- | --- | --- | --- | --- | --- | --- | --- | --- | --- | --- | --- | --- | --- | --- | --- | --- | --- | --- | --- | --- | --- | --- | --- | --- | --- | --- | --- | --- | --- | --- | --- | --- | --- | --- | --- | --- | --- | --- | --- | --- | --- | --- | --- | --- | --- | --- | --- | --- | --- | --- | --- | --- | --- | --- | --- | --- | --- | --- | --- | --- | --- | --- | --- | --- | --- | --- | --- | --- | --- | --- | --- | --- | --- | --- | --- | --- | --- | --- | --- | --- | --- | --- | --- | --- | --- | --- | --- | --- | --- | --- | --- | --- | --- | --- | --- | --- | --- | --- | --- | --- | --- | --- | --- | --- | --- | --- | --- | --- | --- | --- | --- | --- | --- | --- | --- | --- | --- | --- | --- | --- | --- | --- | --- | --- | --- | --- | --- | --- | --- | --- | --- | --- | --- | --- | --- | --- | --- | --- | --- | --- | --- | --- | --- | --- | --- | --- | --- | --- | --- | --- | --- | --- | --- | --- | --- | --- | --- | --- | --- | --- | --- | --- | --- | --- | --- | --- | --- | --- | --- | --- | --- | --- | --- | --- | --- | --- | --- | --- | --- | --- | --- | --- | --- | --- | --- | --- | --- | --- | --- | --- | --- | --- | --- | --- | --- | --- | --- | --- | --- | --- | --- | --- | --- | --- | --- | --- | --- | --- | --- | --- | --- | --- | --- | --- | --- | --- | --- | --- | --- | --- | --- | --- | --- | --- | --- | --- | --- | --- | --- | --- | --- | --- | --- | --- |
| |  | | --- | |

3.

| | **Your Input:** **PF3D7_0309000** | | | 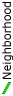 | 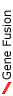 | 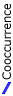 | 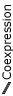 | 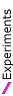 | 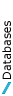 | 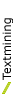 | 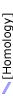 | 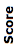 | | --- | --- | --- | --- | --- | --- | --- | --- | --- | --- | --- | --- | | 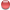 | [PFC0380w](http://string-db.org/newstring_cgi/display_single_node.pl?taskId=E__6vQ1CNvfj&node=400544&targetmode=proteins) | dual-specificity protein phosphatase, putative (575 aa) | |  |  | *(Plasmodium falciparum)* | | **Predicted Functional Partners:** | | | | 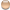 | [PTPS](http://string-db.org/newstring_cgi/display_single_node.pl?taskId=E__6vQ1CNvfj&node=401559&targetmode=proteins) | 6-pyruvoyl tetrahydropterin synthase, putative (173 aa) |  |  |  | [**•**](http://string-db.org/newstring_cgi/show_coexpression_evidence.pl?taskId=E__6vQ1CNvfj&node2=401559) |  |  | [**•**](http://string-db.org/newstring_cgi/show_textmining_evidence.pl?taskId=E__6vQ1CNvfj&node2=401559) |  | 0.797 | | 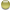 | [PFD0505c](http://string-db.org/newstring_cgi/display_single_node.pl?taskId=E__6vQ1CNvfj&node=400803&targetmode=proteins) | protein phosphatase 2C (906 aa) |  |  |  | [**•**](http://string-db.org/newstring_cgi/show_coexpression_evidence.pl?taskId=E__6vQ1CNvfj&node2=400803) |  |  | [**•**](http://string-db.org/newstring_cgi/show_textmining_evidence.pl?taskId=E__6vQ1CNvfj&node2=400803) |  | 0.734 | | 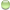 | [PF14_0525](http://string-db.org/newstring_cgi/display_single_node.pl?taskId=E__6vQ1CNvfj&node=399804&targetmode=proteins) | hypothetical protein (89 aa) |  |  |  |  |  |  | [**•**](http://string-db.org/newstring_cgi/show_textmining_evidence.pl?taskId=E__6vQ1CNvfj&node2=399804) |  | 0.685 | | 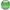 | [PF11_0281](http://string-db.org/newstring_cgi/display_single_node.pl?taskId=E__6vQ1CNvfj&node=398671&targetmode=proteins) | hypothetical protein (247 aa) |  |  |  |  |  |  | [**•**](http://string-db.org/newstring_cgi/show_textmining_evidence.pl?taskId=E__6vQ1CNvfj&node2=398671) |  | 0.685 | | 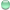 | [PF11_0396](http://string-db.org/newstring_cgi/display_single_node.pl?taskId=E__6vQ1CNvfj&node=398787&targetmode=proteins) | Protein phosphatase 2C (938 aa) |  |  |  |  |  |  | [**•**](http://string-db.org/newstring_cgi/show_textmining_evidence.pl?taskId=E__6vQ1CNvfj&node2=398787) |  | 0.683 | | 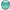 | [PRL](http://string-db.org/newstring_cgi/display_single_node.pl?taskId=E__6vQ1CNvfj&node=398526&targetmode=proteins) | protein tyrosine phosphatase, putative (218 aa) |  |  |  |  |  |  | [**•**](http://string-db.org/newstring_cgi/show_textmining_evidence.pl?taskId=E__6vQ1CNvfj&node2=398526) |  | 0.679 | | 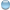 | [Rab1b](http://string-db.org/newstring_cgi/display_single_node.pl?taskId=E__6vQ1CNvfj&node=401084&targetmode=proteins) | Rab1b, GTPase (200 aa) |  |  |  |  |  |  | [**•**](http://string-db.org/newstring_cgi/show_textmining_evidence.pl?taskId=E__6vQ1CNvfj&node2=401084) |  | 0.651 | | 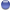 | [VPS16](http://string-db.org/newstring_cgi/display_single_node.pl?taskId=E__6vQ1CNvfj&node=402380&targetmode=proteins) | hypothetical protein, conserved (1032 aa) |  |  |  | [**•**](http://string-db.org/newstring_cgi/show_coexpression_evidence.pl?taskId=E__6vQ1CNvfj&node2=402380) |  |  | [**•**](http://string-db.org/newstring_cgi/show_textmining_evidence.pl?taskId=E__6vQ1CNvfj&node2=402380) |  | 0.637 | | 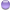 | [PFC0805w](http://string-db.org/newstring_cgi/display_single_node.pl?taskId=E__6vQ1CNvfj&node=400633&targetmode=proteins) | DNA-directed RNA polymerase II, putative; DNA-dependent RNA polymerase catalyzes the transcript [...] (2457 aa) |  |  |  | [**•**](http://string-db.org/newstring_cgi/show_coexpression_evidence.pl?taskId=E__6vQ1CNvfj&node2=400633) |  |  | [**•**](http://string-db.org/newstring_cgi/show_textmining_evidence.pl?taskId=E__6vQ1CNvfj&node2=400633) |  | 0.610 | | 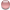 | [PFC0375c](http://string-db.org/newstring_cgi/display_single_node.pl?taskId=E__6vQ1CNvfj&node=400543&targetmode=proteins) | splicing factor, putative (1386 aa) |  |  |  | [**•**](http://string-db.org/newstring_cgi/show_coexpression_evidence.pl?taskId=E__6vQ1CNvfj&node2=400543) |  |  |  |  | 0.547 | |
| --- | --- | --- | --- | --- | --- | --- | --- | --- | --- | --- | --- | --- | --- | --- | --- | --- | --- | --- | --- | --- | --- | --- | --- | --- | --- | --- | --- | --- | --- | --- | --- | --- | --- | --- | --- | --- | --- | --- | --- | --- | --- | --- | --- | --- | --- | --- | --- | --- | --- | --- | --- | --- | --- | --- | --- | --- | --- | --- | --- | --- | --- | --- | --- | --- | --- | --- | --- | --- | --- | --- | --- | --- | --- | --- | --- | --- | --- | --- | --- | --- | --- | --- | --- | --- | --- | --- | --- | --- | --- | --- | --- | --- | --- | --- | --- | --- | --- | --- | --- | --- | --- | --- | --- | --- | --- | --- | --- | --- | --- | --- | --- | --- | --- | --- | --- | --- | --- | --- | --- | --- | --- | --- | --- | --- | --- | --- | --- | --- | --- | --- | --- | --- | --- | --- | --- | --- | --- | --- | --- | --- | --- |

4.

| | | | **Your Input:** **PF3D7_0310300** | | | 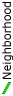 | 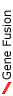 | 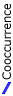 | 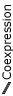 | 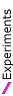 | 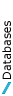 | 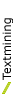 | 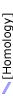 | 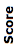 | | --- | --- | --- | --- | --- | --- | --- | --- | --- | --- | --- | --- | | 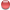 | [PFC0430w](http://string-db.org/newstring_cgi/display_single_node.pl?taskId=aCMeVLpCLVSr&node=400556&targetmode=proteins) | hypothetical protein, conserved (1146 aa) | |  |  | *(Plasmodium falciparum)* | | **Predicted Functional Partners:** | | | | 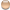 | [PFI0165c](http://string-db.org/newstring_cgi/display_single_node.pl?taskId=aCMeVLpCLVSr&node=401642&targetmode=proteins) | DEAD%2FDEAH box helicase, putative (2536 aa) |  |  |  | [**•**](http://string-db.org/newstring_cgi/show_coexpression_evidence.pl?taskId=aCMeVLpCLVSr&node2=401642) |  |  |  | **•** | 0.855 | | 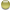 | [PFF1030w](http://string-db.org/newstring_cgi/display_single_node.pl?taskId=aCMeVLpCLVSr&node=401492&targetmode=proteins) | hypothetical protein, conserved (793 aa) |  |  |  | [**•**](http://string-db.org/newstring_cgi/show_coexpression_evidence.pl?taskId=aCMeVLpCLVSr&node2=401492) |  |  |  |  | 0.835 | | 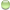 | [PF14_0156](http://string-db.org/newstring_cgi/display_single_node.pl?taskId=aCMeVLpCLVSr&node=399426&targetmode=proteins) | dimethyladenosine transferase, putative (381 aa) |  |  |  | [**•**](http://string-db.org/newstring_cgi/show_coexpression_evidence.pl?taskId=aCMeVLpCLVSr&node2=399426) |  |  |  |  | 0.819 | | 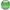 | [PF11_0275](http://string-db.org/newstring_cgi/display_single_node.pl?taskId=aCMeVLpCLVSr&node=398665&targetmode=proteins) | hypothetical protein (1368 aa) |  |  |  | [**•**](http://string-db.org/newstring_cgi/show_coexpression_evidence.pl?taskId=aCMeVLpCLVSr&node2=398665) |  |  |  |  | 0.800 | | 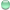 | [CCp2](http://string-db.org/newstring_cgi/display_single_node.pl?taskId=aCMeVLpCLVSr&node=399811&targetmode=proteins) | hypothetical protein (1617 aa) |  |  |  |  |  |  | [**•**](http://string-db.org/newstring_cgi/show_textmining_evidence.pl?taskId=aCMeVLpCLVSr&node2=399811) |  | 0.800 | | 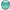 | [PFE0245c](http://string-db.org/newstring_cgi/display_single_node.pl?taskId=aCMeVLpCLVSr&node=401007&targetmode=proteins) | hypothetical protein, conserved (2961 aa) |  |  |  | [**•**](http://string-db.org/newstring_cgi/show_coexpression_evidence.pl?taskId=aCMeVLpCLVSr&node2=401007) |  |  |  |  | 0.788 | | 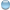 | [PF11_0418](http://string-db.org/newstring_cgi/display_single_node.pl?taskId=aCMeVLpCLVSr&node=398809&targetmode=proteins) | hypothetical protein (2275 aa) |  |  |  | [**•**](http://string-db.org/newstring_cgi/show_coexpression_evidence.pl?taskId=aCMeVLpCLVSr&node2=398809) |  |  |  |  | 0.780 | | 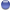 | [PF14_0456](http://string-db.org/newstring_cgi/display_single_node.pl?taskId=aCMeVLpCLVSr&node=399733&targetmode=proteins) | hypothetical protein, conserved (1275 aa) |  |  |  | [**•**](http://string-db.org/newstring_cgi/show_coexpression_evidence.pl?taskId=aCMeVLpCLVSr&node2=399733) |  |  |  |  | 0.774 | | 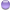 | [MAL7P1.15](http://string-db.org/newstring_cgi/display_single_node.pl?taskId=aCMeVLpCLVSr&node=397385&targetmode=proteins) | hypothetical membrane protein, conserved (4230 aa) |  |  |  | [**•**](http://string-db.org/newstring_cgi/show_coexpression_evidence.pl?taskId=aCMeVLpCLVSr&node2=397385) |  |  |  |  | 0.774 | | 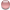 | [PFI1070c-1](http://string-db.org/newstring_cgi/display_single_node.pl?taskId=aCMeVLpCLVSr&node=401826&targetmode=proteins) | BRIX domain, putative (395 aa) |  |  |  | [**•**](http://string-db.org/newstring_cgi/show_coexpression_evidence.pl?taskId=aCMeVLpCLVSr&node2=401826) |  |  |  |  | 0.772 | | | --- | --- | --- | --- | --- | --- | --- | --- | --- | --- | --- | --- | --- | --- | --- | --- | --- | --- | --- | --- | --- | --- | --- | --- | --- | --- | --- | --- | --- | --- | --- | --- | --- | --- | --- | --- | --- | --- | --- | --- | --- | --- | --- | --- | --- | --- | --- | --- | --- | --- | --- | --- | --- | --- | --- | --- | --- | --- | --- | --- | --- | --- | --- | --- | --- | --- | --- | --- | --- | --- | --- | --- | --- | --- | --- | --- | --- | --- | --- | --- | --- | --- | --- | --- | --- | --- | --- | --- | --- | --- | --- | --- | --- | --- | --- | --- | --- | --- | --- | --- | --- | --- | --- | --- | --- | --- | --- | --- | --- | --- | --- | --- | --- | --- | --- | --- | --- | --- | --- | --- | --- | --- | --- | --- | --- | --- | --- | --- | --- | --- | --- | --- | --- | --- | --- | --- | --- | --- | --- | --- | --- | --- | | | --- | --- | --- | --- | --- | --- | --- | --- | --- | --- | --- | --- | --- | --- | --- | --- | --- | --- | --- | --- | --- | --- | --- | --- | --- | --- | --- | --- | --- | --- | --- | --- | --- | --- | --- | --- | --- | --- | --- | --- | --- | --- | --- | --- | --- | --- | --- | --- | --- | --- | --- | --- | --- | --- | --- | --- | --- | --- | --- | --- | --- | --- | --- | --- | --- | --- | --- | --- | --- | --- | --- | --- | --- | --- | --- | --- | --- | --- | --- | --- | --- | --- | --- | --- | --- | --- | --- | --- | --- | --- | --- | --- | --- | --- | --- | --- | --- | --- | --- | --- | --- | --- | --- | --- | --- | --- | --- | --- | --- | --- | --- | --- | --- | --- | --- | --- | --- | --- | --- | --- | --- | --- | --- | --- | --- | --- | --- | --- | --- | --- | --- | --- | --- | --- | --- | --- | --- | --- | --- | --- | --- | --- | --- | |
| --- | --- | --- | --- | --- | --- | --- | --- | --- | --- | --- | --- | --- | --- | --- | --- | --- | --- | --- | --- | --- | --- | --- | --- | --- | --- | --- | --- | --- | --- | --- | --- | --- | --- | --- | --- | --- | --- | --- | --- | --- | --- | --- | --- | --- | --- | --- | --- | --- | --- | --- | --- | --- | --- | --- | --- | --- | --- | --- | --- | --- | --- | --- | --- | --- | --- | --- | --- | --- | --- | --- | --- | --- | --- | --- | --- | --- | --- | --- | --- | --- | --- | --- | --- | --- | --- | --- | --- | --- | --- | --- | --- | --- | --- | --- | --- | --- | --- | --- | --- | --- | --- | --- | --- | --- | --- | --- | --- | --- | --- | --- | --- | --- | --- | --- | --- | --- | --- | --- | --- | --- | --- | --- | --- | --- | --- | --- | --- | --- | --- | --- | --- | --- | --- | --- | --- | --- | --- | --- | --- | --- | --- | --- | --- |
| |  | | --- | |

5.

| | **Your Input:** **PF3D7_0314400** | | | 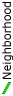 | 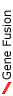 | 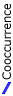 | 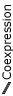 | 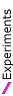 | 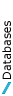 | 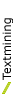 | 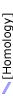 | 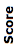 | | --- | --- | --- | --- | --- | --- | --- | --- | --- | --- | --- | --- | | 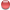 | [PFC0595c](http://string-db.org/newstring_cgi/display_single_node.pl?taskId=iyPT0K3imfRX&node=400594&targetmode=proteins) | serine%2Fthreonine protein phosphatase, putative (308 aa) | |  |  | *(Plasmodium falciparum)* | | **Predicted Functional Partners:** | | | |  | [PF14_0064](http://string-db.org/newstring_cgi/display_single_node.pl?taskId=iyPT0K3imfRX&node=399333&targetmode=proteins) | vacuolar protein sorting 29, putative (194 aa) |  |  |  | [**•**](http://string-db.org/newstring_cgi/show_coexpression_evidence.pl?taskId=iyPT0K3imfRX&node2=399333) |  |  | [**•**](http://string-db.org/newstring_cgi/show_textmining_evidence.pl?taskId=iyPT0K3imfRX&node2=399333) |  | 0.672 | |  | [PF14_0614](http://string-db.org/newstring_cgi/display_single_node.pl?taskId=iyPT0K3imfRX&node=399896&targetmode=proteins) | hypothetical protein (1502 aa) |  |  |  |  |  |  | [**•**](http://string-db.org/newstring_cgi/show_textmining_evidence.pl?taskId=iyPT0K3imfRX&node2=399896) |  | 0.651 | |  | [PF13_0222](http://string-db.org/newstring_cgi/display_single_node.pl?taskId=iyPT0K3imfRX&node=399135&targetmode=proteins) | RNA lariat debranching enzyme, putative (575 aa) |  |  |  |  |  |  | [**•**](http://string-db.org/newstring_cgi/show_textmining_evidence.pl?taskId=iyPT0K3imfRX&node2=399135) |  | 0.651 | |  | [PFL0300c](http://string-db.org/newstring_cgi/display_single_node.pl?taskId=iyPT0K3imfRX&node=402045&targetmode=proteins) | phosphoesterase, putative (304 aa) |  |  |  |  |  |  | [**•**](http://string-db.org/newstring_cgi/show_textmining_evidence.pl?taskId=iyPT0K3imfRX&node2=402045) |  | 0.651 | |  | [PfEF-1beta](http://string-db.org/newstring_cgi/display_single_node.pl?taskId=iyPT0K3imfRX&node=401740&targetmode=proteins) | EF-1B (276 aa) |  |  |  |  |  |  | [**•**](http://string-db.org/newstring_cgi/show_textmining_evidence.pl?taskId=iyPT0K3imfRX&node2=401740) |  | 0.649 | |  | [PFD0770c](http://string-db.org/newstring_cgi/display_single_node.pl?taskId=iyPT0K3imfRX&node=400856&targetmode=proteins) | ribosomal protein l15, putative (220 aa) |  |  |  |  |  |  | [**•**](http://string-db.org/newstring_cgi/show_textmining_evidence.pl?taskId=iyPT0K3imfRX&node2=400856) |  | 0.647 | |  | [PF14_0036](http://string-db.org/newstring_cgi/display_single_node.pl?taskId=iyPT0K3imfRX&node=399305&targetmode=proteins) | acid phosphatase, putative (302 aa) |  |  |  |  |  |  | [**•**](http://string-db.org/newstring_cgi/show_textmining_evidence.pl?taskId=iyPT0K3imfRX&node2=399305) |  | 0.643 | |  | [Rab2](http://string-db.org/newstring_cgi/display_single_node.pl?taskId=iyPT0K3imfRX&node=402291&targetmode=proteins) | Rab2, GTPase (213 aa) |  |  |  |  |  |  | [**•**](http://string-db.org/newstring_cgi/show_textmining_evidence.pl?taskId=iyPT0K3imfRX&node2=402291) |  | 0.641 | |  | [PF14_0280](http://string-db.org/newstring_cgi/display_single_node.pl?taskId=iyPT0K3imfRX&node=399554&targetmode=proteins) | phosphotyrosyl phosphatase activator, putative (319 aa) |  |  |  |  | [**•**](http://string-db.org/newstring_cgi/show_set_evidence.pl?data_channel=experimental&taskId=iyPT0K3imfRX&node2=399554) |  | [**•**](http://string-db.org/newstring_cgi/show_textmining_evidence.pl?taskId=iyPT0K3imfRX&node2=399554) |  | 0.605 | |  | [PFA0515w](http://string-db.org/newstring_cgi/display_single_node.pl?taskId=iyPT0K3imfRX&node=400177&targetmode=proteins) | phosphatidylinositol-4-phosphate 5-kinase, putative (1710 aa) |  |  |  | [**•**](http://string-db.org/newstring_cgi/show_coexpression_evidence.pl?taskId=iyPT0K3imfRX&node2=400177) |  |  | [**•**](http://string-db.org/newstring_cgi/show_textmining_evidence.pl?taskId=iyPT0K3imfRX&node2=400177) |  | 0.601 | |
| --- | --- | --- | --- | --- | --- | --- | --- | --- | --- | --- | --- | --- | --- | --- | --- | --- | --- | --- | --- | --- | --- | --- | --- | --- | --- | --- | --- | --- | --- | --- | --- | --- | --- | --- | --- | --- | --- | --- | --- | --- | --- | --- | --- | --- | --- | --- | --- | --- | --- | --- | --- | --- | --- | --- | --- | --- | --- | --- | --- | --- | --- | --- | --- | --- | --- | --- | --- | --- | --- | --- | --- | --- | --- | --- | --- | --- | --- | --- | --- | --- | --- | --- | --- | --- | --- | --- | --- | --- | --- | --- | --- | --- | --- | --- | --- | --- | --- | --- | --- | --- | --- | --- | --- | --- | --- | --- | --- | --- | --- | --- | --- | --- | --- | --- | --- | --- | --- | --- | --- | --- | --- | --- | --- | --- | --- | --- | --- | --- | --- | --- | --- | --- | --- | --- | --- | --- | --- | --- | --- | --- | --- |

6.

| | **Your Input:** **PF3D7_0319200** | | |  |  |  |  |  |  |  |  |  | | --- | --- | --- | --- | --- | --- | --- | --- | --- | --- | --- | --- | |  | [PFC0850c](http://string-db.org/newstring_cgi/display_single_node.pl?taskId=6rw99wM3Fi_i&node=400643&targetmode=proteins) | hypothetical protein, conserved (906 aa) | |  |  | *(Plasmodium falciparum)* | | **Predicted Functional Partners:** | | | |  | [PFE1055c](http://string-db.org/newstring_cgi/display_single_node.pl?taskId=6rw99wM3Fi_i&node=401169&targetmode=proteins) | hypothetical protein, conserved (518 aa) |  |  |  | [**•**](http://string-db.org/newstring_cgi/show_coexpression_evidence.pl?taskId=6rw99wM3Fi_i&node2=401169) |  |  |  | **•** | 0.547 | |  | [PF13_0263](http://string-db.org/newstring_cgi/display_single_node.pl?taskId=6rw99wM3Fi_i&node=399175&targetmode=proteins) | hypothetical protein, conserved (305 aa) |  |  |  | [**•**](http://string-db.org/newstring_cgi/show_coexpression_evidence.pl?taskId=6rw99wM3Fi_i&node2=399175) |  |  |  |  | 0.435 | |  | [PF13_0042](http://string-db.org/newstring_cgi/display_single_node.pl?taskId=6rw99wM3Fi_i&node=398967&targetmode=proteins) | fork head domain protein, putative (561 aa) |  |  |  | [**•**](http://string-db.org/newstring_cgi/show_coexpression_evidence.pl?taskId=6rw99wM3Fi_i&node2=398967) |  |  |  | **•** | 0.430 | |  | [PF07_0097](http://string-db.org/newstring_cgi/display_single_node.pl?taskId=6rw99wM3Fi_i&node=397800&targetmode=proteins) | hypothetical protein, conserved (1422 aa) |  |  |  | [**•**](http://string-db.org/newstring_cgi/show_coexpression_evidence.pl?taskId=6rw99wM3Fi_i&node2=397800) |  |  |  |  | 0.419 | |  | [PF11_0305](http://string-db.org/newstring_cgi/display_single_node.pl?taskId=6rw99wM3Fi_i&node=398695&targetmode=proteins) | hypothetical protein (634 aa) |  |  |  | [**•**](http://string-db.org/newstring_cgi/show_coexpression_evidence.pl?taskId=6rw99wM3Fi_i&node2=398695) |  |  |  |  | 0.417 | |  | [PF11_0232](http://string-db.org/newstring_cgi/display_single_node.pl?taskId=6rw99wM3Fi_i&node=398621&targetmode=proteins) | hypothetical protein (851 aa) |  |  |  | [**•**](http://string-db.org/newstring_cgi/show_coexpression_evidence.pl?taskId=6rw99wM3Fi_i&node2=398621) |  |  |  |  | 0.414 | |  | [PFL0310c](http://string-db.org/newstring_cgi/display_single_node.pl?taskId=6rw99wM3Fi_i&node=402047&targetmode=proteins) | eukaryotictranslation initiation factor 3 subunit 8, putative (984 aa) |  |  |  | [**•**](http://string-db.org/newstring_cgi/show_coexpression_evidence.pl?taskId=6rw99wM3Fi_i&node2=402047) |  |  |  |  | 0.411 | |  | [PFF0165c](http://string-db.org/newstring_cgi/display_single_node.pl?taskId=6rw99wM3Fi_i&node=401316&targetmode=proteins) | hypothetical protein, conserved (1103 aa) |  |  |  | [**•**](http://string-db.org/newstring_cgi/show_coexpression_evidence.pl?taskId=6rw99wM3Fi_i&node2=401316) |  |  |  |  | 0.411 | |
| --- | --- | --- | --- | --- | --- | --- | --- | --- | --- | --- | --- | --- | --- | --- | --- | --- | --- | --- | --- | --- | --- | --- | --- | --- | --- | --- | --- | --- | --- | --- | --- | --- | --- | --- | --- | --- | --- | --- | --- | --- | --- | --- | --- | --- | --- | --- | --- | --- | --- | --- | --- | --- | --- | --- | --- | --- | --- | --- | --- | --- | --- | --- | --- | --- | --- | --- | --- | --- | --- | --- | --- | --- | --- | --- | --- | --- | --- | --- | --- | --- | --- | --- | --- | --- | --- | --- | --- | --- | --- | --- | --- | --- | --- | --- | --- | --- | --- | --- | --- | --- | --- | --- | --- | --- | --- | --- | --- | --- | --- | --- | --- | --- | --- | --- | --- | --- | --- |

7.

| | | | **Your Input:** **PF3D7_0322100** | | |  |  |  |  |  |  |  |  |  | | --- | --- | --- | --- | --- | --- | --- | --- | --- | --- | --- | --- | |  | [Prt1](http://string-db.org/newstring_cgi/display_single_node.pl?taskId=LJ_XA97bFmio&node=400672&targetmode=proteins) | mRNA capping enzyme (617 aa) | |  |  | *(Plasmodium falciparum)* | | **Predicted Functional Partners:** | | | |  | [Pgt1](http://string-db.org/newstring_cgi/display_single_node.pl?taskId=LJ_XA97bFmio&node=399414&targetmode=proteins) | mRNA capping enzyme, putative (520 aa) |  |  |  |  |  |  | [**•**](http://string-db.org/newstring_cgi/show_textmining_evidence.pl?taskId=LJ_XA97bFmio&node2=399414) |  | 0.909 | |  | [PFI1195c](http://string-db.org/newstring_cgi/display_single_node.pl?taskId=LJ_XA97bFmio&node=401851&targetmode=proteins) | Thiamine pyrophosphokinase (400 aa) |  |  |  |  |  |  | [**•**](http://string-db.org/newstring_cgi/show_textmining_evidence.pl?taskId=LJ_XA97bFmio&node2=401851) |  | 0.579 | |  | [GAK](http://string-db.org/newstring_cgi/display_single_node.pl?taskId=LJ_XA97bFmio&node=400853&targetmode=proteins) | adenylate kinase 1 (229 aa) |  |  |  |  |  |  | [**•**](http://string-db.org/newstring_cgi/show_textmining_evidence.pl?taskId=LJ_XA97bFmio&node2=400853) |  | 0.544 | |  | [PFD0590c](http://string-db.org/newstring_cgi/display_single_node.pl?taskId=LJ_XA97bFmio&node=400817&targetmode=proteins) | DNA polymerase alpha (1912 aa) |  |  |  |  |  |  | [**•**](http://string-db.org/newstring_cgi/show_textmining_evidence.pl?taskId=LJ_XA97bFmio&node2=400817) |  | 0.485 | |  | [PFF1470c](http://string-db.org/newstring_cgi/display_single_node.pl?taskId=LJ_XA97bFmio&node=401582&targetmode=proteins) | DNA polymerase epsilon, catalytic subunit a, putative (2907 aa) |  |  |  |  |  |  | [**•**](http://string-db.org/newstring_cgi/show_textmining_evidence.pl?taskId=LJ_XA97bFmio&node2=401582) |  | 0.484 | |  | [PF10_0362](http://string-db.org/newstring_cgi/display_single_node.pl?taskId=LJ_XA97bFmio&node=398358&targetmode=proteins) | DNA polymerase zeta catalytic subunit, putative (2240 aa) |  |  |  |  |  |  | [**•**](http://string-db.org/newstring_cgi/show_textmining_evidence.pl?taskId=LJ_XA97bFmio&node2=398358) |  | 0.484 | |  | [PF10_0165](http://string-db.org/newstring_cgi/display_single_node.pl?taskId=LJ_XA97bFmio&node=398151&targetmode=proteins) | DNA polymerase delta catalytic subunit; This polymerase possesses two enzymatic activities- DNA [...] (1094 aa) |  |  |  |  |  |  | [**•**](http://string-db.org/newstring_cgi/show_textmining_evidence.pl?taskId=LJ_XA97bFmio&node2=398151) |  | 0.481 | | | --- | --- | --- | --- | --- | --- | --- | --- | --- | --- | --- | --- | --- | --- | --- | --- | --- | --- | --- | --- | --- | --- | --- | --- | --- | --- | --- | --- | --- | --- | --- | --- | --- | --- | --- | --- | --- | --- | --- | --- | --- | --- | --- | --- | --- | --- | --- | --- | --- | --- | --- | --- | --- | --- | --- | --- | --- | --- | --- | --- | --- | --- | --- | --- | --- | --- | --- | --- | --- | --- | --- | --- | --- | --- | --- | --- | --- | --- | --- | --- | --- | --- | --- | --- | --- | --- | --- | --- | --- | --- | --- | --- | --- | --- | --- | --- | --- | --- | --- | --- | --- | --- | --- | --- | --- | --- | | | --- | --- | --- | --- | --- | --- | --- | --- | --- | --- | --- | --- | --- | --- | --- | --- | --- | --- | --- | --- | --- | --- | --- | --- | --- | --- | --- | --- | --- | --- | --- | --- | --- | --- | --- | --- | --- | --- | --- | --- | --- | --- | --- | --- | --- | --- | --- | --- | --- | --- | --- | --- | --- | --- | --- | --- | --- | --- | --- | --- | --- | --- | --- | --- | --- | --- | --- | --- | --- | --- | --- | --- | --- | --- | --- | --- | --- | --- | --- | --- | --- | --- | --- | --- | --- | --- | --- | --- | --- | --- | --- | --- | --- | --- | --- | --- | --- | --- | --- | --- | --- | --- | --- | --- | --- | --- | --- | |
| --- | --- | --- | --- | --- | --- | --- | --- | --- | --- | --- | --- | --- | --- | --- | --- | --- | --- | --- | --- | --- | --- | --- | --- | --- | --- | --- | --- | --- | --- | --- | --- | --- | --- | --- | --- | --- | --- | --- | --- | --- | --- | --- | --- | --- | --- | --- | --- | --- | --- | --- | --- | --- | --- | --- | --- | --- | --- | --- | --- | --- | --- | --- | --- | --- | --- | --- | --- | --- | --- | --- | --- | --- | --- | --- | --- | --- | --- | --- | --- | --- | --- | --- | --- | --- | --- | --- | --- | --- | --- | --- | --- | --- | --- | --- | --- | --- | --- | --- | --- | --- | --- | --- | --- | --- | --- | --- | --- |
| |  | | --- | |

8.

| | **Your Input:** **PF3D7_0410300** | | |  |  |  |  |  |  |  |  |  | | --- | --- | --- | --- | --- | --- | --- | --- | --- | --- | --- | --- | |  | [PFD0505c](http://string-db.org/newstring_cgi/display_single_node.pl?taskId=UATQFNrJFE6Y&node=400803&targetmode=proteins) | protein phosphatase 2C (906 aa) | |  |  | *(Plasmodium falciparum)* | | **Predicted Functional Partners:** | | | |  | [PfEF-1beta](http://string-db.org/newstring_cgi/display_single_node.pl?taskId=UATQFNrJFE6Y&node=401740&targetmode=proteins) | EF-1B (276 aa) |  |  |  |  |  |  | [**•**](http://string-db.org/newstring_cgi/show_textmining_evidence.pl?taskId=UATQFNrJFE6Y&node2=401740) |  | 0.975 | |  | [PFC0380w](http://string-db.org/newstring_cgi/display_single_node.pl?taskId=UATQFNrJFE6Y&node=400544&targetmode=proteins) | dual-specificity protein phosphatase, putative (575 aa) |  |  |  | [**•**](http://string-db.org/newstring_cgi/show_coexpression_evidence.pl?taskId=UATQFNrJFE6Y&node2=400544) |  |  | [**•**](http://string-db.org/newstring_cgi/show_textmining_evidence.pl?taskId=UATQFNrJFE6Y&node2=400544) |  | 0.734 | |  | [RON4](http://string-db.org/newstring_cgi/display_single_node.pl?taskId=UATQFNrJFE6Y&node=398555&targetmode=proteins) | hypothetical protein (2966 aa) |  |  |  |  |  |  | [**•**](http://string-db.org/newstring_cgi/show_textmining_evidence.pl?taskId=UATQFNrJFE6Y&node2=398555) |  | 0.651 | |  | [PfRON2](http://string-db.org/newstring_cgi/display_single_node.pl?taskId=UATQFNrJFE6Y&node=399773&targetmode=proteins) | hypothetical protein (2189 aa) |  |  |  |  |  |  | [**•**](http://string-db.org/newstring_cgi/show_textmining_evidence.pl?taskId=UATQFNrJFE6Y&node2=399773) |  | 0.647 | |  | [PP1](http://string-db.org/newstring_cgi/display_single_node.pl?taskId=UATQFNrJFE6Y&node=399412&targetmode=proteins) | serine%2Fthreonine protein phosphatase, putative (304 aa) |  |  |  |  |  |  | [**•**](http://string-db.org/newstring_cgi/show_textmining_evidence.pl?taskId=UATQFNrJFE6Y&node2=399412) |  | 0.641 | |  | [GAP45](http://string-db.org/newstring_cgi/display_single_node.pl?taskId=UATQFNrJFE6Y&node=402208&targetmode=proteins) | gliding-associated protein 45, GAP45 putative (204 aa) |  |  |  |  |  |  | [**•**](http://string-db.org/newstring_cgi/show_textmining_evidence.pl?taskId=UATQFNrJFE6Y&node2=402208) |  | 0.579 | |  | [PF14_0545](http://string-db.org/newstring_cgi/display_single_node.pl?taskId=UATQFNrJFE6Y&node=399824&targetmode=proteins) | thioredoxin; Participates in various redox reactions through the reversible oxidation of its ac [...] (104 aa) |  |  |  |  |  |  | [**•**](http://string-db.org/newstring_cgi/show_textmining_evidence.pl?taskId=UATQFNrJFE6Y&node2=399824) |  | 0.579 | |  | [PF14_0323](http://string-db.org/newstring_cgi/display_single_node.pl?taskId=UATQFNrJFE6Y&node=399597&targetmode=proteins) | calmodulin; Calmodulin mediates the control of a large number of enzymes and other proteins by [...] (149 aa) |  |  |  |  |  |  | [**•**](http://string-db.org/newstring_cgi/show_textmining_evidence.pl?taskId=UATQFNrJFE6Y&node2=399597) |  | 0.579 | |  | [PFE1360c](http://string-db.org/newstring_cgi/display_single_node.pl?taskId=UATQFNrJFE6Y&node=401232&targetmode=proteins) | methionine aminopeptidase, putative; Removes the amino-terminal methionine from nascent protein [...] (235 aa) |  |  |  | [**•**](http://string-db.org/newstring_cgi/show_coexpression_evidence.pl?taskId=UATQFNrJFE6Y&node2=401232) |  |  | [**•**](http://string-db.org/newstring_cgi/show_textmining_evidence.pl?taskId=UATQFNrJFE6Y&node2=401232) |  | 0.521 | |  | [PF10_0150](http://string-db.org/newstring_cgi/display_single_node.pl?taskId=UATQFNrJFE6Y&node=398134&targetmode=proteins) | methionine aminopeptidase, putative; Removes the amino-terminal methionine from nascent protein [...] (517 aa) |  |  |  | [**•**](http://string-db.org/newstring_cgi/show_coexpression_evidence.pl?taskId=UATQFNrJFE6Y&node2=398134) |  |  | [**•**](http://string-db.org/newstring_cgi/show_textmining_evidence.pl?taskId=UATQFNrJFE6Y&node2=398134) |  | 0.511 | |
| --- | --- | --- | --- | --- | --- | --- | --- | --- | --- | --- | --- | --- | --- | --- | --- | --- | --- | --- | --- | --- | --- | --- | --- | --- | --- | --- | --- | --- | --- | --- | --- | --- | --- | --- | --- | --- | --- | --- | --- | --- | --- | --- | --- | --- | --- | --- | --- | --- | --- | --- | --- | --- | --- | --- | --- | --- | --- | --- | --- | --- | --- | --- | --- | --- | --- | --- | --- | --- | --- | --- | --- | --- | --- | --- | --- | --- | --- | --- | --- | --- | --- | --- | --- | --- | --- | --- | --- | --- | --- | --- | --- | --- | --- | --- | --- | --- | --- | --- | --- | --- | --- | --- | --- | --- | --- | --- | --- | --- | --- | --- | --- | --- | --- | --- | --- | --- | --- | --- | --- | --- | --- | --- | --- | --- | --- | --- | --- | --- | --- | --- | --- | --- | --- | --- | --- | --- | --- | --- | --- | --- | --- |

9.

| | **Your Input:** **PF3D7_0413500** | | |  |  |  |  |  |  |  |  |  | | --- | --- | --- | --- | --- | --- | --- | --- | --- | --- | --- | --- | |  | [PFD0660w](http://string-db.org/newstring_cgi/display_single_node.pl?taskId=2GyPSAAvgl9t&node=400832&targetmode=proteins) | phosphoglycerate mutase, putative (295 aa) | |  |  | *(Plasmodium falciparum)* | | **Predicted Functional Partners:** | | | |  | [ENO](http://string-db.org/newstring_cgi/display_single_node.pl?taskId=2GyPSAAvgl9t&node=398140&targetmode=proteins) | enolase (446 aa) |  |  |  |  |  | [**•**](http://string-db.org/newstring_cgi/show_set_evidence.pl?data_channel=database&taskId=2GyPSAAvgl9t&node2=398140) | [**•**](http://string-db.org/newstring_cgi/show_textmining_evidence.pl?taskId=2GyPSAAvgl9t&node2=398140) |  | 0.962 | |  | [PFI0160w](http://string-db.org/newstring_cgi/display_single_node.pl?taskId=2GyPSAAvgl9t&node=401641&targetmode=proteins) | hypothetical protein, conserved (3135 aa) |  |  |  | [**•**](http://string-db.org/newstring_cgi/show_coexpression_evidence.pl?taskId=2GyPSAAvgl9t&node2=401641) |  |  |  |  | 0.960 | |  | [PGK](http://string-db.org/newstring_cgi/display_single_node.pl?taskId=2GyPSAAvgl9t&node=401833&targetmode=proteins) | Phosphoglycerate kinase (416 aa) |  |  |  | [**•**](http://string-db.org/newstring_cgi/show_coexpression_evidence.pl?taskId=2GyPSAAvgl9t&node2=401833) |  | [**•**](http://string-db.org/newstring_cgi/show_set_evidence.pl?data_channel=database&taskId=2GyPSAAvgl9t&node2=401833) | [**•**](http://string-db.org/newstring_cgi/show_textmining_evidence.pl?taskId=2GyPSAAvgl9t&node2=401833) |  | 0.959 | |  | [PFC0435w](http://string-db.org/newstring_cgi/display_single_node.pl?taskId=2GyPSAAvgl9t&node=400557&targetmode=proteins) | hypothetical protein, conserved (1294 aa) |  |  |  | [**•**](http://string-db.org/newstring_cgi/show_coexpression_evidence.pl?taskId=2GyPSAAvgl9t&node2=400557) |  |  |  |  | 0.944 | |  | [GAP50](http://string-db.org/newstring_cgi/display_single_node.pl?taskId=2GyPSAAvgl9t&node=401788&targetmode=proteins) | acid phosphatase, putative (396 aa) |  |  |  | [**•**](http://string-db.org/newstring_cgi/show_coexpression_evidence.pl?taskId=2GyPSAAvgl9t&node2=401788) |  |  |  |  | 0.935 | |  | [PFB0190c](http://string-db.org/newstring_cgi/display_single_node.pl?taskId=2GyPSAAvgl9t&node=400262&targetmode=proteins) | hypothetical protein (2295 aa) |  |  |  | [**•**](http://string-db.org/newstring_cgi/show_coexpression_evidence.pl?taskId=2GyPSAAvgl9t&node2=400262) |  |  |  |  | 0.925 | |  | [PF14_0341](http://string-db.org/newstring_cgi/display_single_node.pl?taskId=2GyPSAAvgl9t&node=399615&targetmode=proteins) | glucose-6-phosphate isomerase (579 aa) |  |  |  |  |  | [**•**](http://string-db.org/newstring_cgi/show_set_evidence.pl?data_channel=database&taskId=2GyPSAAvgl9t&node2=399615) | [**•**](http://string-db.org/newstring_cgi/show_textmining_evidence.pl?taskId=2GyPSAAvgl9t&node2=399615) |  | 0.911 | |  | [PF11_0208](http://string-db.org/newstring_cgi/display_single_node.pl?taskId=2GyPSAAvgl9t&node=398597&targetmode=proteins) | phosphoglycerate mutase, putative (250 aa) |  |  |  |  |  | [**•**](http://string-db.org/newstring_cgi/show_set_evidence.pl?data_channel=database&taskId=2GyPSAAvgl9t&node2=398597) | [**•**](http://string-db.org/newstring_cgi/show_textmining_evidence.pl?taskId=2GyPSAAvgl9t&node2=398597) |  | 0.910 | |  | [PFL1160c](http://string-db.org/newstring_cgi/display_single_node.pl?taskId=2GyPSAAvgl9t&node=402222&targetmode=proteins) | hypothetical protein, conserved (192 aa) |  |  |  | [**•**](http://string-db.org/newstring_cgi/show_coexpression_evidence.pl?taskId=2GyPSAAvgl9t&node2=402222) |  |  | [**•**](http://string-db.org/newstring_cgi/show_textmining_evidence.pl?taskId=2GyPSAAvgl9t&node2=402222) |  | 0.909 | |  | [SERA-6](http://string-db.org/newstring_cgi/display_single_node.pl?taskId=2GyPSAAvgl9t&node=400296&targetmode=proteins) | cysteine protease, putative (893 aa) |  |  |  | [**•**](http://string-db.org/newstring_cgi/show_coexpression_evidence.pl?taskId=2GyPSAAvgl9t&node2=400296) |  |  |  |  | 0.861 | |  | [MAL7P1.17](http://string-db.org/newstring_cgi/display_single_node.pl?taskId=2GyPSAAvgl9t&node=397405&targetmode=proteins) | hypothetical membrane protein, conserved (3559 aa) |  |  |  | [**•**](http://string-db.org/newstring_cgi/show_coexpression_evidence.pl?taskId=2GyPSAAvgl9t&node2=397405) |  |  |  |  | 0.861 | |  | [ABRA](http://string-db.org/newstring_cgi/display_single_node.pl?taskId=2GyPSAAvgl9t&node=402267&targetmode=proteins) | Merozoite Surface Protein 9, MSP-9 (743 aa) |  |  |  | [**•**](http://string-db.org/newstring_cgi/show_coexpression_evidence.pl?taskId=2GyPSAAvgl9t&node2=402267) |  |  |  |  | 0.861 | |  | [PFL1870c](http://string-db.org/newstring_cgi/display_single_node.pl?taskId=2GyPSAAvgl9t&node=402367&targetmode=proteins) | sphingomyelin phosphodiesterase (393 aa) |  |  |  | [**•**](http://string-db.org/newstring_cgi/show_coexpression_evidence.pl?taskId=2GyPSAAvgl9t&node2=402367) |  |  |  |  | 0.859 | |  | [cyc-2](http://string-db.org/newstring_cgi/display_single_node.pl?taskId=2GyPSAAvgl9t&node=402256&targetmode=proteins) | hypothetical protein, conserved (2281 aa) |  |  |  | [**•**](http://string-db.org/newstring_cgi/show_coexpression_evidence.pl?taskId=2GyPSAAvgl9t&node2=402256) |  |  |  |  | 0.859 | |  | [PFI0975c](http://string-db.org/newstring_cgi/display_single_node.pl?taskId=2GyPSAAvgl9t&node=401807&targetmode=proteins) | hypothetical protein, conserved (3381 aa) |  |  |  | [**•**](http://string-db.org/newstring_cgi/show_coexpression_evidence.pl?taskId=2GyPSAAvgl9t&node2=401807) |  |  |  |  | 0.859 | |  | [PF14_0380](http://string-db.org/newstring_cgi/display_single_node.pl?taskId=2GyPSAAvgl9t&node=399656&targetmode=proteins) | hypothetical protein (1014 aa) |  |  |  | [**•**](http://string-db.org/newstring_cgi/show_coexpression_evidence.pl?taskId=2GyPSAAvgl9t&node2=399656) |  |  |  |  | 0.859 | |  | [Pf92](http://string-db.org/newstring_cgi/display_single_node.pl?taskId=2GyPSAAvgl9t&node=399245&targetmode=proteins) | cysteine-rich surface protein (796 aa) |  |  |  | [**•**](http://string-db.org/newstring_cgi/show_coexpression_evidence.pl?taskId=2GyPSAAvgl9t&node2=399245) |  |  |  |  | 0.857 | |  | [RhopH2](http://string-db.org/newstring_cgi/display_single_node.pl?taskId=2GyPSAAvgl9t&node=401903&targetmode=proteins) | High molecular weight rhoptry protein-2 (1378 aa) |  |  |  | [**•**](http://string-db.org/newstring_cgi/show_coexpression_evidence.pl?taskId=2GyPSAAvgl9t&node2=401903) |  |  |  |  | 0.855 | |  | [PF13_0299](http://string-db.org/newstring_cgi/display_single_node.pl?taskId=2GyPSAAvgl9t&node=399210&targetmode=proteins) | hypothetical protein, conserved (964 aa) |  |  |  | [**•**](http://string-db.org/newstring_cgi/show_coexpression_evidence.pl?taskId=2GyPSAAvgl9t&node2=399210) |  |  |  |  | 0.855 | |  | [PF14_0681](http://string-db.org/newstring_cgi/display_single_node.pl?taskId=2GyPSAAvgl9t&node=399966&targetmode=proteins) | diacylglycerol kinase, putative (488 aa) |  |  |  | [**•**](http://string-db.org/newstring_cgi/show_coexpression_evidence.pl?taskId=2GyPSAAvgl9t&node2=399966) |  |  |  |  | 0.849 | |
| --- | --- | --- | --- | --- | --- | --- | --- | --- | --- | --- | --- | --- | --- | --- | --- | --- | --- | --- | --- | --- | --- | --- | --- | --- | --- | --- | --- | --- | --- | --- | --- | --- | --- | --- | --- | --- | --- | --- | --- | --- | --- | --- | --- | --- | --- | --- | --- | --- | --- | --- | --- | --- | --- | --- | --- | --- | --- | --- | --- | --- | --- | --- | --- | --- | --- | --- | --- | --- | --- | --- | --- | --- | --- | --- | --- | --- | --- | --- | --- | --- | --- | --- | --- | --- | --- | --- | --- | --- | --- | --- | --- | --- | --- | --- | --- | --- | --- | --- | --- | --- | --- | --- | --- | --- | --- | --- | --- | --- | --- | --- | --- | --- | --- | --- | --- | --- | --- | --- | --- | --- | --- | --- | --- | --- | --- | --- | --- | --- | --- | --- | --- | --- | --- | --- | --- | --- | --- | --- | --- | --- | --- | --- | --- | --- | --- | --- | --- | --- | --- | --- | --- | --- | --- | --- | --- | --- | --- | --- | --- | --- | --- | --- | --- | --- | --- | --- | --- | --- | --- | --- | --- | --- | --- | --- | --- | --- | --- | --- | --- | --- | --- | --- | --- | --- | --- | --- | --- | --- | --- | --- | --- | --- | --- | --- | --- | --- | --- | --- | --- | --- | --- | --- | --- | --- | --- | --- | --- | --- | --- | --- | --- | --- | --- | --- | --- | --- | --- | --- | --- | --- | --- | --- | --- | --- | --- | --- | --- | --- | --- | --- | --- | --- | --- | --- | --- | --- | --- | --- | --- | --- | --- | --- | --- | --- | --- | --- | --- | --- | --- | --- | --- | --- | --- | --- | --- | --- | --- | --- | --- | --- | --- |

10,

| | **Your Input:** **PF3D7_0515900** | | |  |  |  |  |  |  |  |  |  | | --- | --- | --- | --- | --- | --- | --- | --- | --- | --- | --- | --- | |  | [PFE0795c](http://string-db.org/newstring_cgi/display_single_node.pl?taskId=dKr_BWKEO7ze&node=401117&targetmode=proteins) | nif-like protein, putative (328 aa) | |  |  | *(Plasmodium falciparum)* | | **Predicted Functional Partners:** | | | |  | [PFE0225w](http://string-db.org/newstring_cgi/display_single_node.pl?taskId=dKr_BWKEO7ze&node=401003&targetmode=proteins) | 3-methyl-2-oxobutanoate dehydrogenase (lipoamide), putative (381 aa) |  |  |  |  |  |  | [**•**](http://string-db.org/newstring_cgi/show_textmining_evidence.pl?taskId=dKr_BWKEO7ze&node2=401003) |  | 0.883 | |  | [PF11_0284](http://string-db.org/newstring_cgi/display_single_node.pl?taskId=dKr_BWKEO7ze&node=398674&targetmode=proteins) | methyltransferase, putative (293 aa) |  |  |  |  |  |  | [**•**](http://string-db.org/newstring_cgi/show_textmining_evidence.pl?taskId=dKr_BWKEO7ze&node2=398674) |  | 0.835 | |  | [PF11_0362](http://string-db.org/newstring_cgi/display_single_node.pl?taskId=dKr_BWKEO7ze&node=398753&targetmode=proteins) | protein phosphatase, putative (689 aa) |  |  |  |  |  |  | [**•**](http://string-db.org/newstring_cgi/show_textmining_evidence.pl?taskId=dKr_BWKEO7ze&node2=398753) |  | 0.688 | |  | [PF10_0124](http://string-db.org/newstring_cgi/display_single_node.pl?taskId=dKr_BWKEO7ze&node=398108&targetmode=proteins) | hypothetical protein (1438 aa) |  |  |  |  |  |  | [**•**](http://string-db.org/newstring_cgi/show_textmining_evidence.pl?taskId=dKr_BWKEO7ze&node2=398108) |  | 0.688 | |  | [PF10_0093](http://string-db.org/newstring_cgi/display_single_node.pl?taskId=dKr_BWKEO7ze&node=398077&targetmode=proteins) | hypothetical protein (345 aa) |  |  |  |  |  |  | [**•**](http://string-db.org/newstring_cgi/show_textmining_evidence.pl?taskId=dKr_BWKEO7ze&node2=398077) |  | 0.688 | |  | [MAL8P1.109](http://string-db.org/newstring_cgi/display_single_node.pl?taskId=dKr_BWKEO7ze&node=397534&targetmode=proteins) | Protein phosphatase 2C, putative (545 aa) |  |  |  |  |  |  | [**•**](http://string-db.org/newstring_cgi/show_textmining_evidence.pl?taskId=dKr_BWKEO7ze&node2=397534) |  | 0.688 | |  | [MAL13P1.44](http://string-db.org/newstring_cgi/display_single_node.pl?taskId=dKr_BWKEO7ze&node=397260&targetmode=proteins) | protein phosphatase 2c-like protein, putative (827 aa) |  |  |  |  |  |  | [**•**](http://string-db.org/newstring_cgi/show_textmining_evidence.pl?taskId=dKr_BWKEO7ze&node2=397260) |  | 0.688 | |  | [MAL13P1.174-1](http://string-db.org/newstring_cgi/display_single_node.pl?taskId=dKr_BWKEO7ze&node=397043&targetmode=proteins) | MSP7-like protein (281 aa) |  |  |  |  |  |  | [**•**](http://string-db.org/newstring_cgi/show_textmining_evidence.pl?taskId=dKr_BWKEO7ze&node2=397043) |  | 0.687 | |  | [PFI1245c](http://string-db.org/newstring_cgi/display_single_node.pl?taskId=dKr_BWKEO7ze&node=401862&targetmode=proteins) | Protein phosphatase-beta (466 aa) |  |  |  |  |  |  | [**•**](http://string-db.org/newstring_cgi/show_textmining_evidence.pl?taskId=dKr_BWKEO7ze&node2=401862) |  | 0.649 | |  | [PfPP5](http://string-db.org/newstring_cgi/display_single_node.pl?taskId=dKr_BWKEO7ze&node=397148&targetmode=proteins) | serine%2Fthreonine protein phosphatase pfPp5 (658 aa) |  |  |  |  |  |  | [**•**](http://string-db.org/newstring_cgi/show_textmining_evidence.pl?taskId=dKr_BWKEO7ze&node2=397148) |  | 0.589 | |
| --- | --- | --- | --- | --- | --- | --- | --- | --- | --- | --- | --- | --- | --- | --- | --- | --- | --- | --- | --- | --- | --- | --- | --- | --- | --- | --- | --- | --- | --- | --- | --- | --- | --- | --- | --- | --- | --- | --- | --- | --- | --- | --- | --- | --- | --- | --- | --- | --- | --- | --- | --- | --- | --- | --- | --- | --- | --- | --- | --- | --- | --- | --- | --- | --- | --- | --- | --- | --- | --- | --- | --- | --- | --- | --- | --- | --- | --- | --- | --- | --- | --- | --- | --- | --- | --- | --- | --- | --- | --- | --- | --- | --- | --- | --- | --- | --- | --- | --- | --- | --- | --- | --- | --- | --- | --- | --- | --- | --- | --- | --- | --- | --- | --- | --- | --- | --- | --- | --- | --- | --- | --- | --- | --- | --- | --- | --- | --- | --- | --- | --- | --- | --- | --- | --- | --- | --- | --- | --- | --- | --- | --- |

11.

| | | | **Your Input:** **PF3D7_0520100** | | |  |  |  |  |  |  |  |  |  | | --- | --- | --- | --- | --- | --- | --- | --- | --- | --- | --- | --- | |  | [PFE1010w](http://string-db.org/newstring_cgi/display_single_node.pl?taskId=XodIhzHs0E9j&node=401160&targetmode=proteins) | protein phosphatase 2c, putative (697 aa) | |  |  | *(Plasmodium falciparum)* | | **Predicted Functional Partners:** | | | |  | [PF14_0523](http://string-db.org/newstring_cgi/display_single_node.pl?taskId=XodIhzHs0E9j&node=399802&targetmode=proteins) | protein phosphatase 2C, putative (289 aa) |  |  |  |  |  |  | [**•**](http://string-db.org/newstring_cgi/show_textmining_evidence.pl?taskId=XodIhzHs0E9j&node2=399802) |  | 0.690 | |  | [PF10_0093](http://string-db.org/newstring_cgi/display_single_node.pl?taskId=XodIhzHs0E9j&node=398077&targetmode=proteins) | hypothetical protein (345 aa) |  |  |  |  |  |  | [**•**](http://string-db.org/newstring_cgi/show_textmining_evidence.pl?taskId=XodIhzHs0E9j&node2=398077) |  | 0.688 | |  | [PfEF-1beta](http://string-db.org/newstring_cgi/display_single_node.pl?taskId=XodIhzHs0E9j&node=401740&targetmode=proteins) | EF-1B (276 aa) |  |  |  | [**•**](http://string-db.org/newstring_cgi/show_coexpression_evidence.pl?taskId=XodIhzHs0E9j&node2=401740) |  |  | [**•**](http://string-db.org/newstring_cgi/show_textmining_evidence.pl?taskId=XodIhzHs0E9j&node2=401740) |  | 0.670 | |  | [PF14_0677](http://string-db.org/newstring_cgi/display_single_node.pl?taskId=XodIhzHs0E9j&node=399962&targetmode=proteins) | RNA 3'-Terminal Phosphate Cyclase-like protein, putative (467 aa) |  |  |  | [**•**](http://string-db.org/newstring_cgi/show_coexpression_evidence.pl?taskId=XodIhzHs0E9j&node2=399962) |  |  |  |  | 0.601 | |  | [PF10_0197](http://string-db.org/newstring_cgi/display_single_node.pl?taskId=XodIhzHs0E9j&node=398186&targetmode=proteins) | hypothetical protein (601 aa) |  |  |  | [**•**](http://string-db.org/newstring_cgi/show_coexpression_evidence.pl?taskId=XodIhzHs0E9j&node2=398186) |  |  |  |  | 0.547 | |  | [PF14_0456](http://string-db.org/newstring_cgi/display_single_node.pl?taskId=XodIhzHs0E9j&node=399733&targetmode=proteins) | hypothetical protein, conserved (1275 aa) |  |  |  | [**•**](http://string-db.org/newstring_cgi/show_coexpression_evidence.pl?taskId=XodIhzHs0E9j&node2=399733) |  |  |  |  | 0.543 | |  | [PFF1070c](http://string-db.org/newstring_cgi/display_single_node.pl?taskId=XodIhzHs0E9j&node=401500&targetmode=proteins) | hypothetical protein, conserved (860 aa) |  |  |  | [**•**](http://string-db.org/newstring_cgi/show_coexpression_evidence.pl?taskId=XodIhzHs0E9j&node2=401500) |  |  |  |  | 0.523 | |  | [PFE0245c](http://string-db.org/newstring_cgi/display_single_node.pl?taskId=XodIhzHs0E9j&node=401007&targetmode=proteins) | hypothetical protein, conserved (2961 aa) |  |  |  | [**•**](http://string-db.org/newstring_cgi/show_coexpression_evidence.pl?taskId=XodIhzHs0E9j&node2=401007) |  |  |  |  | 0.523 | |  | [PF14_0125](http://string-db.org/newstring_cgi/display_single_node.pl?taskId=XodIhzHs0E9j&node=399394&targetmode=proteins) | deoxyhypusine synthase (496 aa) |  |  |  | [**•**](http://string-db.org/newstring_cgi/show_coexpression_evidence.pl?taskId=XodIhzHs0E9j&node2=399394) |  |  |  |  | 0.518 | |  | [PF13_0027](http://string-db.org/newstring_cgi/display_single_node.pl?taskId=XodIhzHs0E9j&node=398952&targetmode=proteins) | hypothetical protein, conserved (771 aa) |  |  |  | [**•**](http://string-db.org/newstring_cgi/show_coexpression_evidence.pl?taskId=XodIhzHs0E9j&node2=398952) |  |  | [**•**](http://string-db.org/newstring_cgi/show_textmining_evidence.pl?taskId=XodIhzHs0E9j&node2=398952) |  | 0.513 | | | --- | --- | --- | --- | --- | --- | --- | --- | --- | --- | --- | --- | --- | --- | --- | --- | --- | --- | --- | --- | --- | --- | --- | --- | --- | --- | --- | --- | --- | --- | --- | --- | --- | --- | --- | --- | --- | --- | --- | --- | --- | --- | --- | --- | --- | --- | --- | --- | --- | --- | --- | --- | --- | --- | --- | --- | --- | --- | --- | --- | --- | --- | --- | --- | --- | --- | --- | --- | --- | --- | --- | --- | --- | --- | --- | --- | --- | --- | --- | --- | --- | --- | --- | --- | --- | --- | --- | --- | --- | --- | --- | --- | --- | --- | --- | --- | --- | --- | --- | --- | --- | --- | --- | --- | --- | --- | --- | --- | --- | --- | --- | --- | --- | --- | --- | --- | --- | --- | --- | --- | --- | --- | --- | --- | --- | --- | --- | --- | --- | --- | --- | --- | --- | --- | --- | --- | --- | --- | --- | --- | --- | --- | | | --- | --- | --- | --- | --- | --- | --- | --- | --- | --- | --- | --- | --- | --- | --- | --- | --- | --- | --- | --- | --- | --- | --- | --- | --- | --- | --- | --- | --- | --- | --- | --- | --- | --- | --- | --- | --- | --- | --- | --- | --- | --- | --- | --- | --- | --- | --- | --- | --- | --- | --- | --- | --- | --- | --- | --- | --- | --- | --- | --- | --- | --- | --- | --- | --- | --- | --- | --- | --- | --- | --- | --- | --- | --- | --- | --- | --- | --- | --- | --- | --- | --- | --- | --- | --- | --- | --- | --- | --- | --- | --- | --- | --- | --- | --- | --- | --- | --- | --- | --- | --- | --- | --- | --- | --- | --- | --- | --- | --- | --- | --- | --- | --- | --- | --- | --- | --- | --- | --- | --- | --- | --- | --- | --- | --- | --- | --- | --- | --- | --- | --- | --- | --- | --- | --- | --- | --- | --- | --- | --- | --- | --- | --- | |
| --- | --- | --- | --- | --- | --- | --- | --- | --- | --- | --- | --- | --- | --- | --- | --- | --- | --- | --- | --- | --- | --- | --- | --- | --- | --- | --- | --- | --- | --- | --- | --- | --- | --- | --- | --- | --- | --- | --- | --- | --- | --- | --- | --- | --- | --- | --- | --- | --- | --- | --- | --- | --- | --- | --- | --- | --- | --- | --- | --- | --- | --- | --- | --- | --- | --- | --- | --- | --- | --- | --- | --- | --- | --- | --- | --- | --- | --- | --- | --- | --- | --- | --- | --- | --- | --- | --- | --- | --- | --- | --- | --- | --- | --- | --- | --- | --- | --- | --- | --- | --- | --- | --- | --- | --- | --- | --- | --- | --- | --- | --- | --- | --- | --- | --- | --- | --- | --- | --- | --- | --- | --- | --- | --- | --- | --- | --- | --- | --- | --- | --- | --- | --- | --- | --- | --- | --- | --- | --- | --- | --- | --- | --- | --- |
| |  | | --- | |

13.

| | **Your Input:** **PF3D7_0610500** | | |  |  |  |  |  |  |  |  |  | | --- | --- | --- | --- | --- | --- | --- | --- | --- | --- | --- | --- | |  | [PFF0515c](http://string-db.org/newstring_cgi/display_single_node.pl?taskId=8xeSrMaYw_2H&node=401386&targetmode=proteins) | hypothetical protein, conserved (123 aa) | |  |  | *(Plasmodium falciparum)* | | **Predicted Functional Partners:** | | | |  | [PF07_0027](http://string-db.org/newstring_cgi/display_single_node.pl?taskId=8xeSrMaYw_2H&node=397729&targetmode=proteins) | DNA-directed RNA polymerase 2 8.2 kDa polypeptide, putative (69 aa) |  |  |  | [**•**](http://string-db.org/newstring_cgi/show_coexpression_evidence.pl?taskId=8xeSrMaYw_2H&node2=397729) |  |  |  |  | 0.653 | |  | [PF13_0296](http://string-db.org/newstring_cgi/display_single_node.pl?taskId=8xeSrMaYw_2H&node=399207&targetmode=proteins) | hypothetical protein, conserved (95 aa) |  |  |  | [**•**](http://string-db.org/newstring_cgi/show_coexpression_evidence.pl?taskId=8xeSrMaYw_2H&node2=399207) |  |  |  |  | 0.570 | |  | [PF14_0072](http://string-db.org/newstring_cgi/display_single_node.pl?taskId=8xeSrMaYw_2H&node=399341&targetmode=proteins) | hypothetical protein, conserved (134 aa) |  |  |  | [**•**](http://string-db.org/newstring_cgi/show_coexpression_evidence.pl?taskId=8xeSrMaYw_2H&node2=399341) |  |  |  |  | 0.528 | |  | [PFF0120w](http://string-db.org/newstring_cgi/display_single_node.pl?taskId=8xeSrMaYw_2H&node=401307&targetmode=proteins) | putative geranylgeranyltransferase (379 aa) |  |  |  | [**•**](http://string-db.org/newstring_cgi/show_coexpression_evidence.pl?taskId=8xeSrMaYw_2H&node2=401307) |  |  |  |  | 0.523 | |  | [MAL13P1.257-1](http://string-db.org/newstring_cgi/display_single_node.pl?taskId=8xeSrMaYw_2H&node=397131&targetmode=proteins) | hypothetical protein, conserved (156 aa) |  |  |  | [**•**](http://string-db.org/newstring_cgi/show_coexpression_evidence.pl?taskId=8xeSrMaYw_2H&node2=397131) |  |  |  |  | 0.507 | |  | [PF13_0341](http://string-db.org/newstring_cgi/display_single_node.pl?taskId=8xeSrMaYw_2H&node=399248&targetmode=proteins) | DNA-directed RNA polymerase 2, putative (205 aa) |  |  |  | [**•**](http://string-db.org/newstring_cgi/show_coexpression_evidence.pl?taskId=8xeSrMaYw_2H&node2=399248) |  |  |  |  | 0.481 | |  | [PF13_0023](http://string-db.org/newstring_cgi/display_single_node.pl?taskId=8xeSrMaYw_2H&node=398948&targetmode=proteins) | DNA-directed RNA polymerase 2, putative; DNA-dependent RNA polymerase catalyzes the transcripti [...] (126 aa) |  |  |  | [**•**](http://string-db.org/newstring_cgi/show_coexpression_evidence.pl?taskId=8xeSrMaYw_2H&node2=398948) |  |  |  |  | 0.466 | |  | [PFE1020w](http://string-db.org/newstring_cgi/display_single_node.pl?taskId=8xeSrMaYw_2H&node=401162&targetmode=proteins) | u6 snRNA-associated sm-like protein Lsm2, putative (103 aa) |  |  |  | [**•**](http://string-db.org/newstring_cgi/show_coexpression_evidence.pl?taskId=8xeSrMaYw_2H&node2=401162) |  |  |  |  | 0.454 | |  | [MAL13P1.253-1](http://string-db.org/newstring_cgi/display_single_node.pl?taskId=8xeSrMaYw_2H&node=397127&targetmode=proteins) | small nuclear ribonucleoprotein, putative (93 aa) |  |  |  | [**•**](http://string-db.org/newstring_cgi/show_coexpression_evidence.pl?taskId=8xeSrMaYw_2H&node2=397127) |  |  |  |  | 0.450 | |  | [MAL7P1.98](http://string-db.org/newstring_cgi/display_single_node.pl?taskId=8xeSrMaYw_2H&node=397521&targetmode=proteins) | hypothetical protein, conserved (153 aa) |  |  |  | [**•**](http://string-db.org/newstring_cgi/show_coexpression_evidence.pl?taskId=8xeSrMaYw_2H&node2=397521) |  |  |  |  | 0.449 | |
| --- | --- | --- | --- | --- | --- | --- | --- | --- | --- | --- | --- | --- | --- | --- | --- | --- | --- | --- | --- | --- | --- | --- | --- | --- | --- | --- | --- | --- | --- | --- | --- | --- | --- | --- | --- | --- | --- | --- | --- | --- | --- | --- | --- | --- | --- | --- | --- | --- | --- | --- | --- | --- | --- | --- | --- | --- | --- | --- | --- | --- | --- | --- | --- | --- | --- | --- | --- | --- | --- | --- | --- | --- | --- | --- | --- | --- | --- | --- | --- | --- | --- | --- | --- | --- | --- | --- | --- | --- | --- | --- | --- | --- | --- | --- | --- | --- | --- | --- | --- | --- | --- | --- | --- | --- | --- | --- | --- | --- | --- | --- | --- | --- | --- | --- | --- | --- | --- | --- | --- | --- | --- | --- | --- | --- | --- | --- | --- | --- | --- | --- | --- | --- | --- | --- | --- | --- | --- | --- | --- | --- | --- |

14.

| | **Your Input:** **PF3D7_0625000.1** | | |  |  |  |  |  |  |  |  |  | | --- | --- | --- | --- | --- | --- | --- | --- | --- | --- | --- | --- | |  | [PfPAP](http://string-db.org/newstring_cgi/display_single_node.pl?taskId=3kKZ3kWhzEb6&node=401529&targetmode=proteins) | hypothetical membrane protein, conserved (461 aa) | |  |  | *(Plasmodium falciparum)* | | **Predicted Functional Partners:** | | | |  | [PF10_0264](http://string-db.org/newstring_cgi/display_single_node.pl?taskId=3kKZ3kWhzEb6&node=398256&targetmode=proteins) | 40S ribosomal protein, putative; Required for the assembly and/or stability of the 40S ribosoma [...] (263 aa) |  |  |  |  |  |  | [**•**](http://string-db.org/newstring_cgi/show_textmining_evidence.pl?taskId=3kKZ3kWhzEb6&node2=398256) |  | 0.782 | |  | [P2](http://string-db.org/newstring_cgi/display_single_node.pl?taskId=3kKZ3kWhzEb6&node=400549&targetmode=proteins) | 60S Acidic ribosomal protein P2; Plays an important role in the elongation step of protein synt [...] (112 aa) |  |  |  |  |  |  | [**•**](http://string-db.org/newstring_cgi/show_textmining_evidence.pl?taskId=3kKZ3kWhzEb6&node2=400549) |  | 0.688 | |  | [PF13_0349](http://string-db.org/newstring_cgi/display_single_node.pl?taskId=3kKZ3kWhzEb6&node=399256&targetmode=proteins) | nucleoside diphosphate kinase b%3B putative (149 aa) |  |  |  |  |  |  | [**•**](http://string-db.org/newstring_cgi/show_textmining_evidence.pl?taskId=3kKZ3kWhzEb6&node2=399256) |  | 0.681 | |  | [PfEF-1beta](http://string-db.org/newstring_cgi/display_single_node.pl?taskId=3kKZ3kWhzEb6&node=401740&targetmode=proteins) | EF-1B (276 aa) |  |  |  |  |  |  | [**•**](http://string-db.org/newstring_cgi/show_textmining_evidence.pl?taskId=3kKZ3kWhzEb6&node2=401740) |  | 0.655 | |  | [PfRACK](http://string-db.org/newstring_cgi/display_single_node.pl?taskId=3kKZ3kWhzEb6&node=397856&targetmode=proteins) | receptor for activated C kinase homolog, PfRACK (323 aa) |  |  |  |  |  |  | [**•**](http://string-db.org/newstring_cgi/show_textmining_evidence.pl?taskId=3kKZ3kWhzEb6&node2=397856) |  | 0.651 | |  | [MEF-1](http://string-db.org/newstring_cgi/display_single_node.pl?taskId=3kKZ3kWhzEb6&node=399216&targetmode=proteins) | elongation factor 1 alpha; This protein promotes the GTP-dependent binding of aminoacyl-tRNA to [...] (443 aa) |  |  |  |  |  |  | [**•**](http://string-db.org/newstring_cgi/show_textmining_evidence.pl?taskId=3kKZ3kWhzEb6&node2=399216) |  | 0.643 | |  | [PF13_0304](http://string-db.org/newstring_cgi/display_single_node.pl?taskId=3kKZ3kWhzEb6&node=399215&targetmode=proteins) | elongation factor 1 alpha; This protein promotes the GTP-dependent binding of aminoacyl-tRNA to [...] (443 aa) |  |  |  |  |  |  | [**•**](http://string-db.org/newstring_cgi/show_textmining_evidence.pl?taskId=3kKZ3kWhzEb6&node2=399215) |  | 0.643 | |  | [PFI0180w](http://string-db.org/newstring_cgi/display_single_node.pl?taskId=3kKZ3kWhzEb6&node=401645&targetmode=proteins) | alpha tubulin; Tubulin is the major constituent of microtubules. It binds two moles of GTP, one [...] (453 aa) |  |  |  |  |  |  | [**•**](http://string-db.org/newstring_cgi/show_textmining_evidence.pl?taskId=3kKZ3kWhzEb6&node2=401645) |  | 0.587 | |  | [PF10_0084](http://string-db.org/newstring_cgi/display_single_node.pl?taskId=3kKZ3kWhzEb6&node=398068&targetmode=proteins) | tubulin beta chain, putative; Tubulin is the major constituent of microtubules. It binds two mo [...] (445 aa) |  |  |  |  |  |  | [**•**](http://string-db.org/newstring_cgi/show_textmining_evidence.pl?taskId=3kKZ3kWhzEb6&node2=398068) |  | 0.579 | |  | [PF08_0108](http://string-db.org/newstring_cgi/display_single_node.pl?taskId=3kKZ3kWhzEb6&node=397945&targetmode=proteins) | pepsinogen, putative (573 aa) |  |  |  | [**•**](http://string-db.org/newstring_cgi/show_coexpression_evidence.pl?taskId=3kKZ3kWhzEb6&node2=397945) |  |  |  |  | 0.499 | |
| --- | --- | --- | --- | --- | --- | --- | --- | --- | --- | --- | --- | --- | --- | --- | --- | --- | --- | --- | --- | --- | --- | --- | --- | --- | --- | --- | --- | --- | --- | --- | --- | --- | --- | --- | --- | --- | --- | --- | --- | --- | --- | --- | --- | --- | --- | --- | --- | --- | --- | --- | --- | --- | --- | --- | --- | --- | --- | --- | --- | --- | --- | --- | --- | --- | --- | --- | --- | --- | --- | --- | --- | --- | --- | --- | --- | --- | --- | --- | --- | --- | --- | --- | --- | --- | --- | --- | --- | --- | --- | --- | --- | --- | --- | --- | --- | --- | --- | --- | --- | --- | --- | --- | --- | --- | --- | --- | --- | --- | --- | --- | --- | --- | --- | --- | --- | --- | --- | --- | --- | --- | --- | --- | --- | --- | --- | --- | --- | --- | --- | --- | --- | --- | --- | --- | --- | --- | --- | --- | --- | --- | --- |

15.

| | | | **Your Input:** **PF3D7_0625000.2** | | |  |  |  |  |  |  |  |  |  | | --- | --- | --- | --- | --- | --- | --- | --- | --- | --- | --- | --- | |  | [PfPAP](http://string-db.org/newstring_cgi/display_single_node.pl?taskId=CDmJucf_pYfn&node=401529&targetmode=proteins) | hypothetical membrane protein, conserved (461 aa) | |  |  | *(Plasmodium falciparum)* | | **Predicted Functional Partners:** | | | |  | [PF10_0264](http://string-db.org/newstring_cgi/display_single_node.pl?taskId=CDmJucf_pYfn&node=398256&targetmode=proteins) | 40S ribosomal protein, putative; Required for the assembly and/or stability of the 40S ribosoma [...] (263 aa) |  |  |  |  |  |  | [**•**](http://string-db.org/newstring_cgi/show_textmining_evidence.pl?taskId=CDmJucf_pYfn&node2=398256) |  | 0.782 | |  | [P2](http://string-db.org/newstring_cgi/display_single_node.pl?taskId=CDmJucf_pYfn&node=400549&targetmode=proteins) | 60S Acidic ribosomal protein P2; Plays an important role in the elongation step of protein synt [...] (112 aa) |  |  |  |  |  |  | [**•**](http://string-db.org/newstring_cgi/show_textmining_evidence.pl?taskId=CDmJucf_pYfn&node2=400549) |  | 0.688 | |  | [PF13_0349](http://string-db.org/newstring_cgi/display_single_node.pl?taskId=CDmJucf_pYfn&node=399256&targetmode=proteins) | nucleoside diphosphate kinase b%3B putative (149 aa) |  |  |  |  |  |  | [**•**](http://string-db.org/newstring_cgi/show_textmining_evidence.pl?taskId=CDmJucf_pYfn&node2=399256) |  | 0.681 | |  | [PfEF-1beta](http://string-db.org/newstring_cgi/display_single_node.pl?taskId=CDmJucf_pYfn&node=401740&targetmode=proteins) | EF-1B (276 aa) |  |  |  |  |  |  | [**•**](http://string-db.org/newstring_cgi/show_textmining_evidence.pl?taskId=CDmJucf_pYfn&node2=401740) |  | 0.655 | |  | [PfRACK](http://string-db.org/newstring_cgi/display_single_node.pl?taskId=CDmJucf_pYfn&node=397856&targetmode=proteins) | receptor for activated C kinase homolog, PfRACK (323 aa) |  |  |  |  |  |  | [**•**](http://string-db.org/newstring_cgi/show_textmining_evidence.pl?taskId=CDmJucf_pYfn&node2=397856) |  | 0.651 | |  | [MEF-1](http://string-db.org/newstring_cgi/display_single_node.pl?taskId=CDmJucf_pYfn&node=399216&targetmode=proteins) | elongation factor 1 alpha; This protein promotes the GTP-dependent binding of aminoacyl-tRNA to [...] (443 aa) |  |  |  |  |  |  | [**•**](http://string-db.org/newstring_cgi/show_textmining_evidence.pl?taskId=CDmJucf_pYfn&node2=399216) |  | 0.643 | |  | [PF13_0304](http://string-db.org/newstring_cgi/display_single_node.pl?taskId=CDmJucf_pYfn&node=399215&targetmode=proteins) | elongation factor 1 alpha; This protein promotes the GTP-dependent binding of aminoacyl-tRNA to [...] (443 aa) |  |  |  |  |  |  | [**•**](http://string-db.org/newstring_cgi/show_textmining_evidence.pl?taskId=CDmJucf_pYfn&node2=399215) |  | 0.643 | |  | [PFI0180w](http://string-db.org/newstring_cgi/display_single_node.pl?taskId=CDmJucf_pYfn&node=401645&targetmode=proteins) | alpha tubulin; Tubulin is the major constituent of microtubules. It binds two moles of GTP, one [...] (453 aa) |  |  |  |  |  |  | [**•**](http://string-db.org/newstring_cgi/show_textmining_evidence.pl?taskId=CDmJucf_pYfn&node2=401645) |  | 0.587 | |  | [PF10_0084](http://string-db.org/newstring_cgi/display_single_node.pl?taskId=CDmJucf_pYfn&node=398068&targetmode=proteins) | tubulin beta chain, putative; Tubulin is the major constituent of microtubules. It binds two mo [...] (445 aa) |  |  |  |  |  |  | [**•**](http://string-db.org/newstring_cgi/show_textmining_evidence.pl?taskId=CDmJucf_pYfn&node2=398068) |  | 0.579 | |  | [PF08_0108](http://string-db.org/newstring_cgi/display_single_node.pl?taskId=CDmJucf_pYfn&node=397945&targetmode=proteins) | pepsinogen, putative (573 aa) |  |  |  | [**•**](http://string-db.org/newstring_cgi/show_coexpression_evidence.pl?taskId=CDmJucf_pYfn&node2=397945) |  |  |  |  | 0.499 | | | --- | --- | --- | --- | --- | --- | --- | --- | --- | --- | --- | --- | --- | --- | --- | --- | --- | --- | --- | --- | --- | --- | --- | --- | --- | --- | --- | --- | --- | --- | --- | --- | --- | --- | --- | --- | --- | --- | --- | --- | --- | --- | --- | --- | --- | --- | --- | --- | --- | --- | --- | --- | --- | --- | --- | --- | --- | --- | --- | --- | --- | --- | --- | --- | --- | --- | --- | --- | --- | --- | --- | --- | --- | --- | --- | --- | --- | --- | --- | --- | --- | --- | --- | --- | --- | --- | --- | --- | --- | --- | --- | --- | --- | --- | --- | --- | --- | --- | --- | --- | --- | --- | --- | --- | --- | --- | --- | --- | --- | --- | --- | --- | --- | --- | --- | --- | --- | --- | --- | --- | --- | --- | --- | --- | --- | --- | --- | --- | --- | --- | --- | --- | --- | --- | --- | --- | --- | --- | --- | --- | --- | --- | | | --- | --- | --- | --- | --- | --- | --- | --- | --- | --- | --- | --- | --- | --- | --- | --- | --- | --- | --- | --- | --- | --- | --- | --- | --- | --- | --- | --- | --- | --- | --- | --- | --- | --- | --- | --- | --- | --- | --- | --- | --- | --- | --- | --- | --- | --- | --- | --- | --- | --- | --- | --- | --- | --- | --- | --- | --- | --- | --- | --- | --- | --- | --- | --- | --- | --- | --- | --- | --- | --- | --- | --- | --- | --- | --- | --- | --- | --- | --- | --- | --- | --- | --- | --- | --- | --- | --- | --- | --- | --- | --- | --- | --- | --- | --- | --- | --- | --- | --- | --- | --- | --- | --- | --- | --- | --- | --- | --- | --- | --- | --- | --- | --- | --- | --- | --- | --- | --- | --- | --- | --- | --- | --- | --- | --- | --- | --- | --- | --- | --- | --- | --- | --- | --- | --- | --- | --- | --- | --- | --- | --- | --- | --- | |
| --- | --- | --- | --- | --- | --- | --- | --- | --- | --- | --- | --- | --- | --- | --- | --- | --- | --- | --- | --- | --- | --- | --- | --- | --- | --- | --- | --- | --- | --- | --- | --- | --- | --- | --- | --- | --- | --- | --- | --- | --- | --- | --- | --- | --- | --- | --- | --- | --- | --- | --- | --- | --- | --- | --- | --- | --- | --- | --- | --- | --- | --- | --- | --- | --- | --- | --- | --- | --- | --- | --- | --- | --- | --- | --- | --- | --- | --- | --- | --- | --- | --- | --- | --- | --- | --- | --- | --- | --- | --- | --- | --- | --- | --- | --- | --- | --- | --- | --- | --- | --- | --- | --- | --- | --- | --- | --- | --- | --- | --- | --- | --- | --- | --- | --- | --- | --- | --- | --- | --- | --- | --- | --- | --- | --- | --- | --- | --- | --- | --- | --- | --- | --- | --- | --- | --- | --- | --- | --- | --- | --- | --- | --- | --- |
|  |

16.

| | **Your Input:** **PF3D7_0705500** | | |  |  |  |  |  |  |  |  |  | | --- | --- | --- | --- | --- | --- | --- | --- | --- | --- | --- | --- | |  | [PF07_0024](http://string-db.org/newstring_cgi/display_single_node.pl?taskId=pM3PSozRLXEt&node=397726&targetmode=proteins) | inositol phosphatase, putative (2814 aa) | |  |  | *(Plasmodium falciparum)* | | **Predicted Functional Partners:** | | | |  | [PF13_0324](http://string-db.org/newstring_cgi/display_single_node.pl?taskId=pM3PSozRLXEt&node=399233&targetmode=proteins) | Sec24 subunit, putative (956 aa) |  |  |  | [**•**](http://string-db.org/newstring_cgi/show_coexpression_evidence.pl?taskId=pM3PSozRLXEt&node2=399233) |  |  |  |  | 0.547 | |  | [PFD0185c](http://string-db.org/newstring_cgi/display_single_node.pl?taskId=pM3PSozRLXEt&node=400738&targetmode=proteins) | peptidase (734 aa) |  |  |  | [**•**](http://string-db.org/newstring_cgi/show_coexpression_evidence.pl?taskId=pM3PSozRLXEt&node2=400738) |  |  |  |  | 0.441 | |
| --- | --- | --- | --- | --- | --- | --- | --- | --- | --- | --- | --- | --- | --- | --- | --- | --- | --- | --- | --- | --- | --- | --- | --- | --- | --- | --- | --- | --- | --- | --- | --- | --- | --- | --- | --- | --- | --- | --- | --- | --- | --- | --- | --- | --- | --- |

17.

| | | | **Your Input:** **PF3D7_0715000** | | |  |  |  |  |  |  |  |  |  | | --- | --- | --- | --- | --- | --- | --- | --- | --- | --- | --- | --- | |  | [PF07_0059](http://string-db.org/newstring_cgi/display_single_node.pl?taskId=jZATL0RWnemv&node=397761&targetmode=proteins) | 4-nitrophenylphosphatase, putative (322 aa) | |  |  | *(Plasmodium falciparum)* | | **Predicted Functional Partners:** | | | |  | [PF10_0334](http://string-db.org/newstring_cgi/display_single_node.pl?taskId=jZATL0RWnemv&node=398330&targetmode=proteins) | flavoprotein subunit of succinate dehydrogenase (631 aa) | [**•**](http://string-db.org/newstring_cgi/show_neighborhood.pl?taskId=jZATL0RWnemv&node2=398330) |  |  |  |  |  | [**•**](http://string-db.org/newstring_cgi/show_textmining_evidence.pl?taskId=jZATL0RWnemv&node2=398330) |  | 0.718 | |  | [PfPP5](http://string-db.org/newstring_cgi/display_single_node.pl?taskId=jZATL0RWnemv&node=397148&targetmode=proteins) | serine%2Fthreonine protein phosphatase pfPp5 (658 aa) |  |  |  | [**•**](http://string-db.org/newstring_cgi/show_coexpression_evidence.pl?taskId=jZATL0RWnemv&node2=397148) |  |  | [**•**](http://string-db.org/newstring_cgi/show_textmining_evidence.pl?taskId=jZATL0RWnemv&node2=397148) |  | 0.707 | |  | [PFL0630w](http://string-db.org/newstring_cgi/display_single_node.pl?taskId=jZATL0RWnemv&node=402112&targetmode=proteins) | iron-sulfur subunit of succinate dehydrogenase (321 aa) | [**•**](http://string-db.org/newstring_cgi/show_neighborhood.pl?taskId=jZATL0RWnemv&node2=402112) |  |  | [**•**](http://string-db.org/newstring_cgi/show_coexpression_evidence.pl?taskId=jZATL0RWnemv&node2=402112) |  |  | [**•**](http://string-db.org/newstring_cgi/show_textmining_evidence.pl?taskId=jZATL0RWnemv&node2=402112) |  | 0.700 | |  | [ATPase4](http://string-db.org/newstring_cgi/display_single_node.pl?taskId=jZATL0RWnemv&node=402103&targetmode=proteins) | non-SERCA-type Ca2%2B -transporting P-ATPase (1208 aa) |  |  |  |  |  |  | [**•**](http://string-db.org/newstring_cgi/show_textmining_evidence.pl?taskId=jZATL0RWnemv&node2=402103) |  | 0.688 | |  | [CK1](http://string-db.org/newstring_cgi/display_single_node.pl?taskId=jZATL0RWnemv&node=398768&targetmode=proteins) | casein kinase 1, PfCK1; Casein kinases are operationally defined by their preferential utilizat [...] (323 aa) |  |  |  |  |  |  | [**•**](http://string-db.org/newstring_cgi/show_textmining_evidence.pl?taskId=jZATL0RWnemv&node2=398768) |  | 0.688 | |  | [PF14_0100](http://string-db.org/newstring_cgi/display_single_node.pl?taskId=jZATL0RWnemv&node=399369&targetmode=proteins) | cytidine triphosphate synthetase (858 aa) | [**•**](http://string-db.org/newstring_cgi/show_neighborhood.pl?taskId=jZATL0RWnemv&node2=399369) |  |  | [**•**](http://string-db.org/newstring_cgi/show_coexpression_evidence.pl?taskId=jZATL0RWnemv&node2=399369) |  |  | [**•**](http://string-db.org/newstring_cgi/show_textmining_evidence.pl?taskId=jZATL0RWnemv&node2=399369) |  | 0.624 | |  | [PFC0395w](http://string-db.org/newstring_cgi/display_single_node.pl?taskId=jZATL0RWnemv&node=400548&targetmode=proteins) | asparagine synthetase, putative (610 aa) | [**•**](http://string-db.org/newstring_cgi/show_neighborhood.pl?taskId=jZATL0RWnemv&node2=400548) |  |  | [**•**](http://string-db.org/newstring_cgi/show_coexpression_evidence.pl?taskId=jZATL0RWnemv&node2=400548) | [**•**](http://string-db.org/newstring_cgi/show_set_evidence.pl?data_channel=experimental&taskId=jZATL0RWnemv&node2=400548) |  | [**•**](http://string-db.org/newstring_cgi/show_textmining_evidence.pl?taskId=jZATL0RWnemv&node2=400548) |  | 0.604 | |  | [PF07_0043](http://string-db.org/newstring_cgi/display_single_node.pl?taskId=jZATL0RWnemv&node=397745&targetmode=proteins) | 60S ribosomal protein L34-a, putative (150 aa) |  | [**•**](http://string-db.org/newstring_cgi/show_fusion_evidence.pl?taskId=jZATL0RWnemv&node2=397745) |  | [**•**](http://string-db.org/newstring_cgi/show_coexpression_evidence.pl?taskId=jZATL0RWnemv&node2=397745) |  |  |  |  | 0.573 | |  | [PFE0660c](http://string-db.org/newstring_cgi/display_single_node.pl?taskId=jZATL0RWnemv&node=401091&targetmode=proteins) | purine nucleotide phosphorylase, putative (245 aa) |  |  |  |  |  | [**•**](http://string-db.org/newstring_cgi/show_set_evidence.pl?data_channel=database&taskId=jZATL0RWnemv&node2=401091) | [**•**](http://string-db.org/newstring_cgi/show_textmining_evidence.pl?taskId=jZATL0RWnemv&node2=401091) |  | 0.573 | |  | [PF14_0520](http://string-db.org/newstring_cgi/display_single_node.pl?taskId=jZATL0RWnemv&node=399799&targetmode=proteins) | 6-phosphogluconate dehydrogenase, decarboxylating, putative (468 aa) |  |  |  | [**•**](http://string-db.org/newstring_cgi/show_coexpression_evidence.pl?taskId=jZATL0RWnemv&node2=399799) |  |  | [**•**](http://string-db.org/newstring_cgi/show_textmining_evidence.pl?taskId=jZATL0RWnemv&node2=399799) |  | 0.566 | | | --- | --- | --- | --- | --- | --- | --- | --- | --- | --- | --- | --- | --- | --- | --- | --- | --- | --- | --- | --- | --- | --- | --- | --- | --- | --- | --- | --- | --- | --- | --- | --- | --- | --- | --- | --- | --- | --- | --- | --- | --- | --- | --- | --- | --- | --- | --- | --- | --- | --- | --- | --- | --- | --- | --- | --- | --- | --- | --- | --- | --- | --- | --- | --- | --- | --- | --- | --- | --- | --- | --- | --- | --- | --- | --- | --- | --- | --- | --- | --- | --- | --- | --- | --- | --- | --- | --- | --- | --- | --- | --- | --- | --- | --- | --- | --- | --- | --- | --- | --- | --- | --- | --- | --- | --- | --- | --- | --- | --- | --- | --- | --- | --- | --- | --- | --- | --- | --- | --- | --- | --- | --- | --- | --- | --- | --- | --- | --- | --- | --- | --- | --- | --- | --- | --- | --- | --- | --- | --- | --- | --- | --- | | | --- | --- | --- | --- | --- | --- | --- | --- | --- | --- | --- | --- | --- | --- | --- | --- | --- | --- | --- | --- | --- | --- | --- | --- | --- | --- | --- | --- | --- | --- | --- | --- | --- | --- | --- | --- | --- | --- | --- | --- | --- | --- | --- | --- | --- | --- | --- | --- | --- | --- | --- | --- | --- | --- | --- | --- | --- | --- | --- | --- | --- | --- | --- | --- | --- | --- | --- | --- | --- | --- | --- | --- | --- | --- | --- | --- | --- | --- | --- | --- | --- | --- | --- | --- | --- | --- | --- | --- | --- | --- | --- | --- | --- | --- | --- | --- | --- | --- | --- | --- | --- | --- | --- | --- | --- | --- | --- | --- | --- | --- | --- | --- | --- | --- | --- | --- | --- | --- | --- | --- | --- | --- | --- | --- | --- | --- | --- | --- | --- | --- | --- | --- | --- | --- | --- | --- | --- | --- | --- | --- | --- | --- | --- | |
| --- | --- | --- | --- | --- | --- | --- | --- | --- | --- | --- | --- | --- | --- | --- | --- | --- | --- | --- | --- | --- | --- | --- | --- | --- | --- | --- | --- | --- | --- | --- | --- | --- | --- | --- | --- | --- | --- | --- | --- | --- | --- | --- | --- | --- | --- | --- | --- | --- | --- | --- | --- | --- | --- | --- | --- | --- | --- | --- | --- | --- | --- | --- | --- | --- | --- | --- | --- | --- | --- | --- | --- | --- | --- | --- | --- | --- | --- | --- | --- | --- | --- | --- | --- | --- | --- | --- | --- | --- | --- | --- | --- | --- | --- | --- | --- | --- | --- | --- | --- | --- | --- | --- | --- | --- | --- | --- | --- | --- | --- | --- | --- | --- | --- | --- | --- | --- | --- | --- | --- | --- | --- | --- | --- | --- | --- | --- | --- | --- | --- | --- | --- | --- | --- | --- | --- | --- | --- | --- | --- | --- | --- | --- | --- |
| |  | | --- | |

18.

| | | | **Your Input:** **PF3D7_0726900** | | |  |  |  |  |  |  |  |  |  | | --- | --- | --- | --- | --- | --- | --- | --- | --- | --- | --- | --- | |  | [PF07_0110](http://string-db.org/newstring_cgi/display_single_node.pl?taskId=_mAYLeZFFv4u&node=397814&targetmode=proteins) | hypothetical protein, conserved (519 aa) | |  |  | *(Plasmodium falciparum)* | | **Predicted Functional Partners:** | | | |  | [PF10_0124](http://string-db.org/newstring_cgi/display_single_node.pl?taskId=_mAYLeZFFv4u&node=398108&targetmode=proteins) | hypothetical protein (1438 aa) |  |  |  |  |  |  | [**•**](http://string-db.org/newstring_cgi/show_textmining_evidence.pl?taskId=_mAYLeZFFv4u&node2=398108) |  | 0.690 | |  | [PF11_0362](http://string-db.org/newstring_cgi/display_single_node.pl?taskId=_mAYLeZFFv4u&node=398753&targetmode=proteins) | protein phosphatase, putative (689 aa) |  |  |  |  |  |  | [**•**](http://string-db.org/newstring_cgi/show_textmining_evidence.pl?taskId=_mAYLeZFFv4u&node2=398753) |  | 0.688 | |  | [PF10_0093](http://string-db.org/newstring_cgi/display_single_node.pl?taskId=_mAYLeZFFv4u&node=398077&targetmode=proteins) | hypothetical protein (345 aa) |  |  |  |  |  |  | [**•**](http://string-db.org/newstring_cgi/show_textmining_evidence.pl?taskId=_mAYLeZFFv4u&node2=398077) |  | 0.688 | |  | [MAL8P1.109](http://string-db.org/newstring_cgi/display_single_node.pl?taskId=_mAYLeZFFv4u&node=397534&targetmode=proteins) | Protein phosphatase 2C, putative (545 aa) |  |  |  |  |  |  | [**•**](http://string-db.org/newstring_cgi/show_textmining_evidence.pl?taskId=_mAYLeZFFv4u&node2=397534) |  | 0.688 | |  | [MAL13P1.44](http://string-db.org/newstring_cgi/display_single_node.pl?taskId=_mAYLeZFFv4u&node=397260&targetmode=proteins) | protein phosphatase 2c-like protein, putative (827 aa) |  |  |  |  |  |  | [**•**](http://string-db.org/newstring_cgi/show_textmining_evidence.pl?taskId=_mAYLeZFFv4u&node2=397260) |  | 0.688 | |  | [MAL13P1.174-1](http://string-db.org/newstring_cgi/display_single_node.pl?taskId=_mAYLeZFFv4u&node=397043&targetmode=proteins) | MSP7-like protein (281 aa) |  |  |  |  |  |  | [**•**](http://string-db.org/newstring_cgi/show_textmining_evidence.pl?taskId=_mAYLeZFFv4u&node2=397043) |  | 0.688 | |  | [PfPP5](http://string-db.org/newstring_cgi/display_single_node.pl?taskId=_mAYLeZFFv4u&node=397148&targetmode=proteins) | serine%2Fthreonine protein phosphatase pfPp5 (658 aa) |  |  |  | [**•**](http://string-db.org/newstring_cgi/show_coexpression_evidence.pl?taskId=_mAYLeZFFv4u&node2=397148) |  |  | [**•**](http://string-db.org/newstring_cgi/show_textmining_evidence.pl?taskId=_mAYLeZFFv4u&node2=397148) |  | 0.668 | |  | [PFI1245c](http://string-db.org/newstring_cgi/display_single_node.pl?taskId=_mAYLeZFFv4u&node=401862&targetmode=proteins) | Protein phosphatase-beta (466 aa) |  |  |  |  |  |  | [**•**](http://string-db.org/newstring_cgi/show_textmining_evidence.pl?taskId=_mAYLeZFFv4u&node2=401862) |  | 0.651 | |  | [PFL0320w](http://string-db.org/newstring_cgi/display_single_node.pl?taskId=_mAYLeZFFv4u&node=402049&targetmode=proteins) | hypothetical protein, conserved (346 aa) |  |  |  |  |  |  | [**•**](http://string-db.org/newstring_cgi/show_textmining_evidence.pl?taskId=_mAYLeZFFv4u&node2=402049) |  | 0.485 | |  | [PF13_0027](http://string-db.org/newstring_cgi/display_single_node.pl?taskId=_mAYLeZFFv4u&node=398952&targetmode=proteins) | hypothetical protein, conserved (771 aa) |  |  |  |  |  |  | [**•**](http://string-db.org/newstring_cgi/show_textmining_evidence.pl?taskId=_mAYLeZFFv4u&node2=398952) |  | 0.485 | | | --- | --- | --- | --- | --- | --- | --- | --- | --- | --- | --- | --- | --- | --- | --- | --- | --- | --- | --- | --- | --- | --- | --- | --- | --- | --- | --- | --- | --- | --- | --- | --- | --- | --- | --- | --- | --- | --- | --- | --- | --- | --- | --- | --- | --- | --- | --- | --- | --- | --- | --- | --- | --- | --- | --- | --- | --- | --- | --- | --- | --- | --- | --- | --- | --- | --- | --- | --- | --- | --- | --- | --- | --- | --- | --- | --- | --- | --- | --- | --- | --- | --- | --- | --- | --- | --- | --- | --- | --- | --- | --- | --- | --- | --- | --- | --- | --- | --- | --- | --- | --- | --- | --- | --- | --- | --- | --- | --- | --- | --- | --- | --- | --- | --- | --- | --- | --- | --- | --- | --- | --- | --- | --- | --- | --- | --- | --- | --- | --- | --- | --- | --- | --- | --- | --- | --- | --- | --- | --- | --- | --- | --- | | | --- | --- | --- | --- | --- | --- | --- | --- | --- | --- | --- | --- | --- | --- | --- | --- | --- | --- | --- | --- | --- | --- | --- | --- | --- | --- | --- | --- | --- | --- | --- | --- | --- | --- | --- | --- | --- | --- | --- | --- | --- | --- | --- | --- | --- | --- | --- | --- | --- | --- | --- | --- | --- | --- | --- | --- | --- | --- | --- | --- | --- | --- | --- | --- | --- | --- | --- | --- | --- | --- | --- | --- | --- | --- | --- | --- | --- | --- | --- | --- | --- | --- | --- | --- | --- | --- | --- | --- | --- | --- | --- | --- | --- | --- | --- | --- | --- | --- | --- | --- | --- | --- | --- | --- | --- | --- | --- | --- | --- | --- | --- | --- | --- | --- | --- | --- | --- | --- | --- | --- | --- | --- | --- | --- | --- | --- | --- | --- | --- | --- | --- | --- | --- | --- | --- | --- | --- | --- | --- | --- | --- | --- | --- | |
| --- | --- | --- | --- | --- | --- | --- | --- | --- | --- | --- | --- | --- | --- | --- | --- | --- | --- | --- | --- | --- | --- | --- | --- | --- | --- | --- | --- | --- | --- | --- | --- | --- | --- | --- | --- | --- | --- | --- | --- | --- | --- | --- | --- | --- | --- | --- | --- | --- | --- | --- | --- | --- | --- | --- | --- | --- | --- | --- | --- | --- | --- | --- | --- | --- | --- | --- | --- | --- | --- | --- | --- | --- | --- | --- | --- | --- | --- | --- | --- | --- | --- | --- | --- | --- | --- | --- | --- | --- | --- | --- | --- | --- | --- | --- | --- | --- | --- | --- | --- | --- | --- | --- | --- | --- | --- | --- | --- | --- | --- | --- | --- | --- | --- | --- | --- | --- | --- | --- | --- | --- | --- | --- | --- | --- | --- | --- | --- | --- | --- | --- | --- | --- | --- | --- | --- | --- | --- | --- | --- | --- | --- | --- | --- |
| |  | | --- | |

19.

| | **Your Input:** **PF3D7_0802800** | | |  |  |  |  |  |  |  |  |  | | --- | --- | --- | --- | --- | --- | --- | --- | --- | --- | --- | --- | |  | [PF08_0129](http://string-db.org/newstring_cgi/display_single_node.pl?taskId=5fQPujFcjC05&node=397966&targetmode=proteins) | protein phosphatase, putative (604 aa) | |  |  | *(Plasmodium falciparum)* | | **Predicted Functional Partners:** | | | |  | [PF14_0607](http://string-db.org/newstring_cgi/display_single_node.pl?taskId=5fQPujFcjC05&node=399889&targetmode=proteins) | hypothetical protein (1068 aa) |  |  |  | [**•**](http://string-db.org/newstring_cgi/show_coexpression_evidence.pl?taskId=5fQPujFcjC05&node2=399889) |  |  |  |  | 0.960 | |  | [PfRON2](http://string-db.org/newstring_cgi/display_single_node.pl?taskId=5fQPujFcjC05&node=399773&targetmode=proteins) | hypothetical protein (2189 aa) |  |  |  | [**•**](http://string-db.org/newstring_cgi/show_coexpression_evidence.pl?taskId=5fQPujFcjC05&node2=399773) |  |  |  |  | 0.952 | |  | [PF14_0323](http://string-db.org/newstring_cgi/display_single_node.pl?taskId=5fQPujFcjC05&node=399597&targetmode=proteins) | calmodulin; Calmodulin mediates the control of a large number of enzymes and other proteins by [...] (149 aa) |  |  |  | [**•**](http://string-db.org/newstring_cgi/show_coexpression_evidence.pl?taskId=5fQPujFcjC05&node2=399597) | [**•**](http://string-db.org/newstring_cgi/show_set_evidence.pl?data_channel=experimental&taskId=5fQPujFcjC05&node2=399597) | [**•**](http://string-db.org/newstring_cgi/show_set_evidence.pl?data_channel=database&taskId=5fQPujFcjC05&node2=399597) | [**•**](http://string-db.org/newstring_cgi/show_textmining_evidence.pl?taskId=5fQPujFcjC05&node2=399597) |  | 0.939 | |  | [PFL0300c](http://string-db.org/newstring_cgi/display_single_node.pl?taskId=5fQPujFcjC05&node=402045&targetmode=proteins) | phosphoesterase, putative (304 aa) |  |  |  | [**•**](http://string-db.org/newstring_cgi/show_coexpression_evidence.pl?taskId=5fQPujFcjC05&node2=402045) |  |  | [**•**](http://string-db.org/newstring_cgi/show_textmining_evidence.pl?taskId=5fQPujFcjC05&node2=402045) |  | 0.937 | |  | [PFE0785c](http://string-db.org/newstring_cgi/display_single_node.pl?taskId=5fQPujFcjC05&node=401115&targetmode=proteins) | drug%2Fmetabolite transporter (456 aa) |  |  |  | [**•**](http://string-db.org/newstring_cgi/show_coexpression_evidence.pl?taskId=5fQPujFcjC05&node2=401115) |  |  |  |  | 0.935 | |  | [PFF0200c-1](http://string-db.org/newstring_cgi/display_single_node.pl?taskId=5fQPujFcjC05&node=401323&targetmode=proteins) | hypothetical protein, conserved (1979 aa) |  |  |  | [**•**](http://string-db.org/newstring_cgi/show_coexpression_evidence.pl?taskId=5fQPujFcjC05&node2=401323) |  |  |  |  | 0.927 | |  | [PF08_0082](http://string-db.org/newstring_cgi/display_single_node.pl?taskId=5fQPujFcjC05&node=397919&targetmode=proteins) | hypothetical protein, conserved (662 aa) |  |  |  | [**•**](http://string-db.org/newstring_cgi/show_coexpression_evidence.pl?taskId=5fQPujFcjC05&node2=397919) |  |  |  |  | 0.927 | |  | [PF10_0254](http://string-db.org/newstring_cgi/display_single_node.pl?taskId=5fQPujFcjC05&node=398245&targetmode=proteins) | hypothetical protein (303 aa) |  |  |  | [**•**](http://string-db.org/newstring_cgi/show_coexpression_evidence.pl?taskId=5fQPujFcjC05&node2=398245) |  |  |  |  | 0.925 | |  | [PFL1565c](http://string-db.org/newstring_cgi/display_single_node.pl?taskId=5fQPujFcjC05&node=402305&targetmode=proteins) | hypothetical protein, conserved (959 aa) |  |  |  | [**•**](http://string-db.org/newstring_cgi/show_coexpression_evidence.pl?taskId=5fQPujFcjC05&node2=402305) |  |  |  |  | 0.901 | |  | [PFL2505c](http://string-db.org/newstring_cgi/display_single_node.pl?taskId=5fQPujFcjC05&node=402494&targetmode=proteins) | hypothetical protein, conserved (2215 aa) |  |  |  | [**•**](http://string-db.org/newstring_cgi/show_coexpression_evidence.pl?taskId=5fQPujFcjC05&node2=402494) |  |  |  |  | 0.877 | |  | [PFD0380c](http://string-db.org/newstring_cgi/display_single_node.pl?taskId=5fQPujFcjC05&node=400778&targetmode=proteins) | hypothetical protein, conserved (1629 aa) |  |  |  | [**•**](http://string-db.org/newstring_cgi/show_coexpression_evidence.pl?taskId=5fQPujFcjC05&node2=400778) |  |  |  |  | 0.877 | |  | [PfPKAc](http://string-db.org/newstring_cgi/display_single_node.pl?taskId=5fQPujFcjC05&node=401954&targetmode=proteins) | cAMP-dependent protein kinase catalytic subunit (342 aa) |  |  |  | [**•**](http://string-db.org/newstring_cgi/show_coexpression_evidence.pl?taskId=5fQPujFcjC05&node2=401954) |  |  | [**•**](http://string-db.org/newstring_cgi/show_textmining_evidence.pl?taskId=5fQPujFcjC05&node2=401954) |  | 0.870 | |  | [PFF1365c](http://string-db.org/newstring_cgi/display_single_node.pl?taskId=5fQPujFcjC05&node=401560&targetmode=proteins) | HECT-domain (ubiquitin-transferase), putative; Putative E3 ubiquitin-protein ligase (By similar [...] (10286 aa) |  |  |  | [**•**](http://string-db.org/newstring_cgi/show_coexpression_evidence.pl?taskId=5fQPujFcjC05&node2=401560) |  |  |  |  | 0.869 | |  | [MAL13P1.130-1](http://string-db.org/newstring_cgi/display_single_node.pl?taskId=5fQPujFcjC05&node=396996&targetmode=proteins) | hypothetical protein, conserved (303 aa) |  |  |  | [**•**](http://string-db.org/newstring_cgi/show_coexpression_evidence.pl?taskId=5fQPujFcjC05&node2=396996) |  |  | [**•**](http://string-db.org/newstring_cgi/show_textmining_evidence.pl?taskId=5fQPujFcjC05&node2=396996) |  | 0.869 | |  | [PF11_0300](http://string-db.org/newstring_cgi/display_single_node.pl?taskId=5fQPujFcjC05&node=398690&targetmode=proteins) | hypothetical protein (642 aa) |  |  |  | [**•**](http://string-db.org/newstring_cgi/show_coexpression_evidence.pl?taskId=5fQPujFcjC05&node2=398690) |  |  |  |  | 0.867 | |  | [MAL8P1.150](http://string-db.org/newstring_cgi/display_single_node.pl?taskId=5fQPujFcjC05&node=397573&targetmode=proteins) | hypothetical protein, conserved (2166 aa) |  |  |  | [**•**](http://string-db.org/newstring_cgi/show_coexpression_evidence.pl?taskId=5fQPujFcjC05&node2=397573) |  |  |  |  | 0.861 | |  | [MAL13P1.38](http://string-db.org/newstring_cgi/display_single_node.pl?taskId=5fQPujFcjC05&node=397241&targetmode=proteins) | hypothetical protein, conserved (796 aa) |  |  |  | [**•**](http://string-db.org/newstring_cgi/show_coexpression_evidence.pl?taskId=5fQPujFcjC05&node2=397241) |  |  |  |  | 0.861 | |  | [MAL8P1.73](http://string-db.org/newstring_cgi/display_single_node.pl?taskId=5fQPujFcjC05&node=397675&targetmode=proteins) | hypothetical protein, conserved (1213 aa) |  |  |  | [**•**](http://string-db.org/newstring_cgi/show_coexpression_evidence.pl?taskId=5fQPujFcjC05&node2=397675) |  |  |  |  | 0.861 | |  | [MAL7P1.119](http://string-db.org/newstring_cgi/display_single_node.pl?taskId=5fQPujFcjC05&node=397358&targetmode=proteins) | hypothetical protein, conserved (749 aa) |  |  |  | [**•**](http://string-db.org/newstring_cgi/show_coexpression_evidence.pl?taskId=5fQPujFcjC05&node2=397358) |  |  |  |  | 0.861 | |  | [PFI1560c](http://string-db.org/newstring_cgi/display_single_node.pl?taskId=5fQPujFcjC05&node=401927&targetmode=proteins) | hypothetical protein, conserved (1247 aa) |  |  |  | [**•**](http://string-db.org/newstring_cgi/show_coexpression_evidence.pl?taskId=5fQPujFcjC05&node2=401927) |  |  |  |  | 0.859 | |
| --- | --- | --- | --- | --- | --- | --- | --- | --- | --- | --- | --- | --- | --- | --- | --- | --- | --- | --- | --- | --- | --- | --- | --- | --- | --- | --- | --- | --- | --- | --- | --- | --- | --- | --- | --- | --- | --- | --- | --- | --- | --- | --- | --- | --- | --- | --- | --- | --- | --- | --- | --- | --- | --- | --- | --- | --- | --- | --- | --- | --- | --- | --- | --- | --- | --- | --- | --- | --- | --- | --- | --- | --- | --- | --- | --- | --- | --- | --- | --- | --- | --- | --- | --- | --- | --- | --- | --- | --- | --- | --- | --- | --- | --- | --- | --- | --- | --- | --- | --- | --- | --- | --- | --- | --- | --- | --- | --- | --- | --- | --- | --- | --- | --- | --- | --- | --- | --- | --- | --- | --- | --- | --- | --- | --- | --- | --- | --- | --- | --- | --- | --- | --- | --- | --- | --- | --- | --- | --- | --- | --- | --- | --- | --- | --- | --- | --- | --- | --- | --- | --- | --- | --- | --- | --- | --- | --- | --- | --- | --- | --- | --- | --- | --- | --- | --- | --- | --- | --- | --- | --- | --- | --- | --- | --- | --- | --- | --- | --- | --- | --- | --- | --- | --- | --- | --- | --- | --- | --- | --- | --- | --- | --- | --- | --- | --- | --- | --- | --- | --- | --- | --- | --- | --- | --- | --- | --- | --- | --- | --- | --- | --- | --- | --- | --- | --- | --- | --- | --- | --- | --- | --- | --- | --- | --- | --- | --- | --- | --- | --- | --- | --- | --- | --- | --- | --- | --- | --- | --- | --- | --- | --- | --- | --- | --- | --- | --- | --- | --- | --- | --- | --- | --- | --- | --- | --- | --- | --- | --- | --- | --- | --- |

20.

| | **Your Input:** **>PF3D7_0805600** | | |  |  |  |  |  |  |  |  |  | | --- | --- | --- | --- | --- | --- | --- | --- | --- | --- | --- | --- | |  | [MAL8P1.202](http://string-db.org/newstring_cgi/display_single_node.pl?taskId=ZzWWZsanfjRY&node=397594&targetmode=proteins) | hypothetical protein, conserved (302 aa) | |  |  | *(Plasmodium falciparum)* | | **Predicted Functional Partners:** | | | |  | [PFL0305c](http://string-db.org/newstring_cgi/display_single_node.pl?taskId=ZzWWZsanfjRY&node=402046&targetmode=proteins) | hypothetical protein, conserved (449 aa) |  |  |  |  |  |  | [**•**](http://string-db.org/newstring_cgi/show_textmining_evidence.pl?taskId=ZzWWZsanfjRY&node2=402046) |  | 0.786 | |  | [PFI1220w-1](http://string-db.org/newstring_cgi/display_single_node.pl?taskId=ZzWWZsanfjRY&node=401857&targetmode=proteins) | hypothetical protein, conserved (170 aa) |  |  |  |  |  |  | [**•**](http://string-db.org/newstring_cgi/show_textmining_evidence.pl?taskId=ZzWWZsanfjRY&node2=401857) |  | 0.782 | |  | [MAL7P1.339](http://string-db.org/newstring_cgi/display_single_node.pl?taskId=ZzWWZsanfjRY&node=397472&targetmode=proteins) | Ca%2B%2B chelating serine protease, putative (165 aa) |  |  |  |  |  |  | [**•**](http://string-db.org/newstring_cgi/show_textmining_evidence.pl?taskId=ZzWWZsanfjRY&node2=397472) |  | 0.782 | |  | [PF14_0036](http://string-db.org/newstring_cgi/display_single_node.pl?taskId=ZzWWZsanfjRY&node=399305&targetmode=proteins) | acid phosphatase, putative (302 aa) |  |  |  |  |  |  | [**•**](http://string-db.org/newstring_cgi/show_textmining_evidence.pl?taskId=ZzWWZsanfjRY&node2=399305) |  | 0.688 | |  | [PF14_0662](http://string-db.org/newstring_cgi/display_single_node.pl?taskId=ZzWWZsanfjRY&node=399946&targetmode=proteins) | hypothetical protein (437 aa) |  |  |  |  |  |  | [**•**](http://string-db.org/newstring_cgi/show_textmining_evidence.pl?taskId=ZzWWZsanfjRY&node2=399946) |  | 0.659 | |  | [PFI1216w](http://string-db.org/newstring_cgi/display_single_node.pl?taskId=ZzWWZsanfjRY&node=401856&targetmode=proteins) | telomeric repeat binding factor 1 (101 aa) |  |  |  |  |  |  | [**•**](http://string-db.org/newstring_cgi/show_textmining_evidence.pl?taskId=ZzWWZsanfjRY&node2=401856) |  | 0.585 | |  | [PFF0573c](http://string-db.org/newstring_cgi/display_single_node.pl?taskId=ZzWWZsanfjRY&node=401398&targetmode=proteins) | 60S ribosomal protein L39, putative (62 aa) |  |  |  |  |  |  | [**•**](http://string-db.org/newstring_cgi/show_textmining_evidence.pl?taskId=ZzWWZsanfjRY&node2=401398) |  | 0.585 | |  | [PFF0685c](http://string-db.org/newstring_cgi/display_single_node.pl?taskId=ZzWWZsanfjRY&node=401422&targetmode=proteins) | Hypothetical protein, conserved (1842 aa) |  |  |  |  |  |  | [**•**](http://string-db.org/newstring_cgi/show_textmining_evidence.pl?taskId=ZzWWZsanfjRY&node2=401422) |  | 0.581 | |  | [PFF0683c](http://string-db.org/newstring_cgi/display_single_node.pl?taskId=ZzWWZsanfjRY&node=401421&targetmode=proteins) | conserved Plasmodium protein, unknown function (935 aa) |  |  |  |  |  |  | [**•**](http://string-db.org/newstring_cgi/show_textmining_evidence.pl?taskId=ZzWWZsanfjRY&node2=401421) |  | 0.581 | |  | [PFL1920c](http://string-db.org/newstring_cgi/display_single_node.pl?taskId=ZzWWZsanfjRY&node=402377&targetmode=proteins) | hydroxyethylthiazole kinase, putative (302 aa) |  |  |  |  |  |  | [**•**](http://string-db.org/newstring_cgi/show_textmining_evidence.pl?taskId=ZzWWZsanfjRY&node2=402377) |  | 0.579 | |
| --- | --- | --- | --- | --- | --- | --- | --- | --- | --- | --- | --- | --- | --- | --- | --- | --- | --- | --- | --- | --- | --- | --- | --- | --- | --- | --- | --- | --- | --- | --- | --- | --- | --- | --- | --- | --- | --- | --- | --- | --- | --- | --- | --- | --- | --- | --- | --- | --- | --- | --- | --- | --- | --- | --- | --- | --- | --- | --- | --- | --- | --- | --- | --- | --- | --- | --- | --- | --- | --- | --- | --- | --- | --- | --- | --- | --- | --- | --- | --- | --- | --- | --- | --- | --- | --- | --- | --- | --- | --- | --- | --- | --- | --- | --- | --- | --- | --- | --- | --- | --- | --- | --- | --- | --- | --- | --- | --- | --- | --- | --- | --- | --- | --- | --- | --- | --- | --- | --- | --- | --- | --- | --- | --- | --- | --- | --- | --- | --- | --- | --- | --- | --- | --- | --- | --- | --- | --- | --- | --- | --- | --- |

21.

| | | | **Your Input:** **PF3D7_0810300** | | |  |  |  |  |  |  |  |  |  | | --- | --- | --- | --- | --- | --- | --- | --- | --- | --- | --- | --- | |  | [MAL8P1.109](http://string-db.org/newstring_cgi/display_single_node.pl?taskId=so6lh2WOiIo4&node=397534&targetmode=proteins) | Protein phosphatase 2C, putative (545 aa) | |  |  | *(Plasmodium falciparum)* | | **Predicted Functional Partners:** | | | |  | [PFD1110w](http://string-db.org/newstring_cgi/display_single_node.pl?taskId=so6lh2WOiIo4&node=400931&targetmode=proteins) | hypothetical membrane protein, conserved (372 aa) |  |  |  | [**•**](http://string-db.org/newstring_cgi/show_coexpression_evidence.pl?taskId=so6lh2WOiIo4&node2=400931) |  |  |  |  | 0.960 | |  | [MAL13P1.130-1](http://string-db.org/newstring_cgi/display_single_node.pl?taskId=so6lh2WOiIo4&node=396996&targetmode=proteins) | hypothetical protein, conserved (303 aa) |  |  |  | [**•**](http://string-db.org/newstring_cgi/show_coexpression_evidence.pl?taskId=so6lh2WOiIo4&node2=396996) |  |  |  |  | 0.960 | |  | [MAL8P1.153-1](http://string-db.org/newstring_cgi/display_single_node.pl?taskId=so6lh2WOiIo4&node=397576&targetmode=proteins) | hypothetical protein, conserved (2577 aa) |  |  |  | [**•**](http://string-db.org/newstring_cgi/show_coexpression_evidence.pl?taskId=so6lh2WOiIo4&node2=397576) |  |  |  |  | 0.958 | |  | [PF11_0477](http://string-db.org/newstring_cgi/display_single_node.pl?taskId=so6lh2WOiIo4&node=398868&targetmode=proteins) | CCAAT-box DNA binding protein subunit B (1301 aa) |  |  |  | [**•**](http://string-db.org/newstring_cgi/show_coexpression_evidence.pl?taskId=so6lh2WOiIo4&node2=398868) |  |  |  |  | 0.954 | |  | [ABRA](http://string-db.org/newstring_cgi/display_single_node.pl?taskId=so6lh2WOiIo4&node=402267&targetmode=proteins) | Merozoite Surface Protein 9, MSP-9 (743 aa) |  |  |  | [**•**](http://string-db.org/newstring_cgi/show_coexpression_evidence.pl?taskId=so6lh2WOiIo4&node2=402267) |  |  |  |  | 0.952 | |  | [MSP1](http://string-db.org/newstring_cgi/display_single_node.pl?taskId=so6lh2WOiIo4&node=401910&targetmode=proteins) | merozoite surface protein 1, precursor (1720 aa) |  |  |  | [**•**](http://string-db.org/newstring_cgi/show_coexpression_evidence.pl?taskId=so6lh2WOiIo4&node2=401910) |  |  |  |  | 0.948 | |  | [PFC0945w](http://string-db.org/newstring_cgi/display_single_node.pl?taskId=so6lh2WOiIo4&node=400665&targetmode=proteins) | protein kinase, putative (328 aa) |  |  |  | [**•**](http://string-db.org/newstring_cgi/show_coexpression_evidence.pl?taskId=so6lh2WOiIo4&node2=400665) |  |  |  |  | 0.938 | |  | [PFL2215w](http://string-db.org/newstring_cgi/display_single_node.pl?taskId=so6lh2WOiIo4&node=402438&targetmode=proteins) | actin; Actins are highly conserved proteins that are involved in various types of cell motility [...] (376 aa) |  |  |  | [**•**](http://string-db.org/newstring_cgi/show_coexpression_evidence.pl?taskId=so6lh2WOiIo4&node2=402438) |  |  |  |  | 0.931 | |  | [PF11_0464](http://string-db.org/newstring_cgi/display_single_node.pl?taskId=so6lh2WOiIo4&node=398854&targetmode=proteins) | serine%2Fthreonine protein kinase (2075 aa) |  |  |  | [**•**](http://string-db.org/newstring_cgi/show_coexpression_evidence.pl?taskId=so6lh2WOiIo4&node2=398854) |  |  |  |  | 0.925 | |  | [RON4](http://string-db.org/newstring_cgi/display_single_node.pl?taskId=so6lh2WOiIo4&node=398555&targetmode=proteins) | hypothetical protein (2966 aa) |  |  |  | [**•**](http://string-db.org/newstring_cgi/show_coexpression_evidence.pl?taskId=so6lh2WOiIo4&node2=398555) |  |  |  |  | 0.925 | |  | [CPK1](http://string-db.org/newstring_cgi/display_single_node.pl?taskId=so6lh2WOiIo4&node=400394&targetmode=proteins) | Pf Calcium-dependent protein kinase 1; Calcium-dependent kinase which may phosphorylate protein [...] (524 aa) |  |  |  | [**•**](http://string-db.org/newstring_cgi/show_coexpression_evidence.pl?taskId=so6lh2WOiIo4&node2=400394) |  |  |  |  | 0.913 | |  | [MAL7P1.119](http://string-db.org/newstring_cgi/display_single_node.pl?taskId=so6lh2WOiIo4&node=397358&targetmode=proteins) | hypothetical protein, conserved (749 aa) |  |  |  | [**•**](http://string-db.org/newstring_cgi/show_coexpression_evidence.pl?taskId=so6lh2WOiIo4&node2=397358) |  |  |  |  | 0.913 | |  | [PF11_0415](http://string-db.org/newstring_cgi/display_single_node.pl?taskId=so6lh2WOiIo4&node=398806&targetmode=proteins) | hypothetical protein (167 aa) |  |  |  | [**•**](http://string-db.org/newstring_cgi/show_coexpression_evidence.pl?taskId=so6lh2WOiIo4&node2=398806) |  |  |  |  | 0.911 | |  | [PFL1565c](http://string-db.org/newstring_cgi/display_single_node.pl?taskId=so6lh2WOiIo4&node=402305&targetmode=proteins) | hypothetical protein, conserved (959 aa) |  |  |  | [**•**](http://string-db.org/newstring_cgi/show_coexpression_evidence.pl?taskId=so6lh2WOiIo4&node2=402305) |  |  |  |  | 0.907 | |  | [PFE0785c](http://string-db.org/newstring_cgi/display_single_node.pl?taskId=so6lh2WOiIo4&node=401115&targetmode=proteins) | drug%2Fmetabolite transporter (456 aa) |  |  |  | [**•**](http://string-db.org/newstring_cgi/show_coexpression_evidence.pl?taskId=so6lh2WOiIo4&node2=401115) |  |  |  |  | 0.897 | |  | [PFL2505c](http://string-db.org/newstring_cgi/display_single_node.pl?taskId=so6lh2WOiIo4&node=402494&targetmode=proteins) | hypothetical protein, conserved (2215 aa) |  |  |  | [**•**](http://string-db.org/newstring_cgi/show_coexpression_evidence.pl?taskId=so6lh2WOiIo4&node2=402494) |  |  |  |  | 0.889 | |  | [PDI-14](http://string-db.org/newstring_cgi/display_single_node.pl?taskId=so6lh2WOiIo4&node=399979&targetmode=proteins) | protein disulfide isomerase, putative (553 aa) |  |  |  | [**•**](http://string-db.org/newstring_cgi/show_coexpression_evidence.pl?taskId=so6lh2WOiIo4&node2=399979) |  |  |  |  | 0.889 | |  | [MTIP](http://string-db.org/newstring_cgi/display_single_node.pl?taskId=so6lh2WOiIo4&node=402440&targetmode=proteins) | myosin A tail domain interacting protein MTIP, putative (204 aa) |  |  |  | [**•**](http://string-db.org/newstring_cgi/show_coexpression_evidence.pl?taskId=so6lh2WOiIo4&node2=402440) |  |  | [**•**](http://string-db.org/newstring_cgi/show_textmining_evidence.pl?taskId=so6lh2WOiIo4&node2=402440) |  | 0.887 | |  | [MAL13P1.308-1](http://string-db.org/newstring_cgi/display_single_node.pl?taskId=so6lh2WOiIo4&node=397184&targetmode=proteins) | hypothetical protein, conserved (2605 aa) |  |  |  | [**•**](http://string-db.org/newstring_cgi/show_coexpression_evidence.pl?taskId=so6lh2WOiIo4&node2=397184) |  |  |  |  | 0.879 | |  | [PF10_0306](http://string-db.org/newstring_cgi/display_single_node.pl?taskId=so6lh2WOiIo4&node=398298&targetmode=proteins) | MORN repeat containing protein (422 aa) |  |  |  | [**•**](http://string-db.org/newstring_cgi/show_coexpression_evidence.pl?taskId=so6lh2WOiIo4&node2=398298) |  |  |  |  | 0.863 | | | --- | --- | --- | --- | --- | --- | --- | --- | --- | --- | --- | --- | --- | --- | --- | --- | --- | --- | --- | --- | --- | --- | --- | --- | --- | --- | --- | --- | --- | --- | --- | --- | --- | --- | --- | --- | --- | --- | --- | --- | --- | --- | --- | --- | --- | --- | --- | --- | --- | --- | --- | --- | --- | --- | --- | --- | --- | --- | --- | --- | --- | --- | --- | --- | --- | --- | --- | --- | --- | --- | --- | --- | --- | --- | --- | --- | --- | --- | --- | --- | --- | --- | --- | --- | --- | --- | --- | --- | --- | --- | --- | --- | --- | --- | --- | --- | --- | --- | --- | --- | --- | --- | --- | --- | --- | --- | --- | --- | --- | --- | --- | --- | --- | --- | --- | --- | --- | --- | --- | --- | --- | --- | --- | --- | --- | --- | --- | --- | --- | --- | --- | --- | --- | --- | --- | --- | --- | --- | --- | --- | --- | --- | --- | --- | --- | --- | --- | --- | --- | --- | --- | --- | --- | --- | --- | --- | --- | --- | --- | --- | --- | --- | --- | --- | --- | --- | --- | --- | --- | --- | --- | --- | --- | --- | --- | --- | --- | --- | --- | --- | --- | --- | --- | --- | --- | --- | --- | --- | --- | --- | --- | --- | --- | --- | --- | --- | --- | --- | --- | --- | --- | --- | --- | --- | --- | --- | --- | --- | --- | --- | --- | --- | --- | --- | --- | --- | --- | --- | --- | --- | --- | --- | --- | --- | --- | --- | --- | --- | --- | --- | --- | --- | --- | --- | --- | --- | --- | --- | --- | --- | --- | --- | --- | --- | --- | --- | --- | --- | --- | --- | --- | --- | --- | --- | --- | --- | --- | --- | --- | --- | --- | --- | | | --- | --- | --- | --- | --- | --- | --- | --- | --- | --- | --- | --- | --- | --- | --- | --- | --- | --- | --- | --- | --- | --- | --- | --- | --- | --- | --- | --- | --- | --- | --- | --- | --- | --- | --- | --- | --- | --- | --- | --- | --- | --- | --- | --- | --- | --- | --- | --- | --- | --- | --- | --- | --- | --- | --- | --- | --- | --- | --- | --- | --- | --- | --- | --- | --- | --- | --- | --- | --- | --- | --- | --- | --- | --- | --- | --- | --- | --- | --- | --- | --- | --- | --- | --- | --- | --- | --- | --- | --- | --- | --- | --- | --- | --- | --- | --- | --- | --- | --- | --- | --- | --- | --- | --- | --- | --- | --- | --- | --- | --- | --- | --- | --- | --- | --- | --- | --- | --- | --- | --- | --- | --- | --- | --- | --- | --- | --- | --- | --- | --- | --- | --- | --- | --- | --- | --- | --- | --- | --- | --- | --- | --- | --- | --- | --- | --- | --- | --- | --- | --- | --- | --- | --- | --- | --- | --- | --- | --- | --- | --- | --- | --- | --- | --- | --- | --- | --- | --- | --- | --- | --- | --- | --- | --- | --- | --- | --- | --- | --- | --- | --- | --- | --- | --- | --- | --- | --- | --- | --- | --- | --- | --- | --- | --- | --- | --- | --- | --- | --- | --- | --- | --- | --- | --- | --- | --- | --- | --- | --- | --- | --- | --- | --- | --- | --- | --- | --- | --- | --- | --- | --- | --- | --- | --- | --- | --- | --- | --- | --- | --- | --- | --- | --- | --- | --- | --- | --- | --- | --- | --- | --- | --- | --- | --- | --- | --- | --- | --- | --- | --- | --- | --- | --- | --- | --- | --- | --- | --- | --- | --- | --- | --- | --- | |
| --- | --- | --- | --- | --- | --- | --- | --- | --- | --- | --- | --- | --- | --- | --- | --- | --- | --- | --- | --- | --- | --- | --- | --- | --- | --- | --- | --- | --- | --- | --- | --- | --- | --- | --- | --- | --- | --- | --- | --- | --- | --- | --- | --- | --- | --- | --- | --- | --- | --- | --- | --- | --- | --- | --- | --- | --- | --- | --- | --- | --- | --- | --- | --- | --- | --- | --- | --- | --- | --- | --- | --- | --- | --- | --- | --- | --- | --- | --- | --- | --- | --- | --- | --- | --- | --- | --- | --- | --- | --- | --- | --- | --- | --- | --- | --- | --- | --- | --- | --- | --- | --- | --- | --- | --- | --- | --- | --- | --- | --- | --- | --- | --- | --- | --- | --- | --- | --- | --- | --- | --- | --- | --- | --- | --- | --- | --- | --- | --- | --- | --- | --- | --- | --- | --- | --- | --- | --- | --- | --- | --- | --- | --- | --- | --- | --- | --- | --- | --- | --- | --- | --- | --- | --- | --- | --- | --- | --- | --- | --- | --- | --- | --- | --- | --- | --- | --- | --- | --- | --- | --- | --- | --- | --- | --- | --- | --- | --- | --- | --- | --- | --- | --- | --- | --- | --- | --- | --- | --- | --- | --- | --- | --- | --- | --- | --- | --- | --- | --- | --- | --- | --- | --- | --- | --- | --- | --- | --- | --- | --- | --- | --- | --- | --- | --- | --- | --- | --- | --- | --- | --- | --- | --- | --- | --- | --- | --- | --- | --- | --- | --- | --- | --- | --- | --- | --- | --- | --- | --- | --- | --- | --- | --- | --- | --- | --- | --- | --- | --- | --- | --- | --- | --- | --- | --- | --- | --- | --- | --- | --- | --- | --- | --- | --- |
| |  | | --- | |

22.

| | | | **Your Input:** **PF3D7_0810500** | | |  |  |  |  |  |  |  |  |  | | --- | --- | --- | --- | --- | --- | --- | --- | --- | --- | --- | --- | |  | [MAL8P1.108](http://string-db.org/newstring_cgi/display_single_node.pl?taskId=XLWV9AtO_y4O&node=397533&targetmode=proteins) | protein phosphatase, putative (303 aa) | |  |  | *(Plasmodium falciparum)* | | **Predicted Functional Partners:** | | | |  | [PF10_0093](http://string-db.org/newstring_cgi/display_single_node.pl?taskId=XLWV9AtO_y4O&node=398077&targetmode=proteins) | hypothetical protein (345 aa) |  |  |  |  |  |  | [**•**](http://string-db.org/newstring_cgi/show_textmining_evidence.pl?taskId=XLWV9AtO_y4O&node2=398077) |  | 0.688 | |  | [PFL0320w](http://string-db.org/newstring_cgi/display_single_node.pl?taskId=XLWV9AtO_y4O&node=402049&targetmode=proteins) | hypothetical protein, conserved (346 aa) |  |  |  |  |  |  | [**•**](http://string-db.org/newstring_cgi/show_textmining_evidence.pl?taskId=XLWV9AtO_y4O&node2=402049) |  | 0.486 | |  | [PF13_0027](http://string-db.org/newstring_cgi/display_single_node.pl?taskId=XLWV9AtO_y4O&node=398952&targetmode=proteins) | hypothetical protein, conserved (771 aa) |  |  |  |  |  |  | [**•**](http://string-db.org/newstring_cgi/show_textmining_evidence.pl?taskId=XLWV9AtO_y4O&node2=398952) |  | 0.486 | |  | [MAL13P1.168-1](http://string-db.org/newstring_cgi/display_single_node.pl?taskId=XLWV9AtO_y4O&node=397036&targetmode=proteins) | hypothetical protein, conserved (267 aa) |  |  |  |  |  |  | [**•**](http://string-db.org/newstring_cgi/show_textmining_evidence.pl?taskId=XLWV9AtO_y4O&node2=397036) |  | 0.486 | |  | [PF14_0525](http://string-db.org/newstring_cgi/display_single_node.pl?taskId=XLWV9AtO_y4O&node=399804&targetmode=proteins) | hypothetical protein (89 aa) |  |  |  |  |  |  | [**•**](http://string-db.org/newstring_cgi/show_textmining_evidence.pl?taskId=XLWV9AtO_y4O&node2=399804) |  | 0.484 | |  | [PF11_0281](http://string-db.org/newstring_cgi/display_single_node.pl?taskId=XLWV9AtO_y4O&node=398671&targetmode=proteins) | hypothetical protein (247 aa) |  |  |  |  |  |  | [**•**](http://string-db.org/newstring_cgi/show_textmining_evidence.pl?taskId=XLWV9AtO_y4O&node2=398671) |  | 0.484 | |  | [MAL13P1.275-1](http://string-db.org/newstring_cgi/display_single_node.pl?taskId=XLWV9AtO_y4O&node=397149&targetmode=proteins) | NLI interacting factor-like phosphatase, putative (1288 aa) |  |  |  |  |  |  | [**•**](http://string-db.org/newstring_cgi/show_textmining_evidence.pl?taskId=XLWV9AtO_y4O&node2=397149) |  | 0.484 | |  | [PRL](http://string-db.org/newstring_cgi/display_single_node.pl?taskId=XLWV9AtO_y4O&node=398526&targetmode=proteins) | protein tyrosine phosphatase, putative (218 aa) |  |  |  |  |  |  | [**•**](http://string-db.org/newstring_cgi/show_textmining_evidence.pl?taskId=XLWV9AtO_y4O&node2=398526) |  | 0.466 | |  | [PF10_0124](http://string-db.org/newstring_cgi/display_single_node.pl?taskId=XLWV9AtO_y4O&node=398108&targetmode=proteins) | hypothetical protein (1438 aa) |  |  |  |  |  |  | [**•**](http://string-db.org/newstring_cgi/show_textmining_evidence.pl?taskId=XLWV9AtO_y4O&node2=398108) |  | 0.460 | |  | [PF10_0104](http://string-db.org/newstring_cgi/display_single_node.pl?taskId=XLWV9AtO_y4O&node=398088&targetmode=proteins) | hypothetical protein (223 aa) |  |  |  | [**•**](http://string-db.org/newstring_cgi/show_coexpression_evidence.pl?taskId=XLWV9AtO_y4O&node2=398088) |  |  |  |  | 0.458 | | | --- | --- | --- | --- | --- | --- | --- | --- | --- | --- | --- | --- | --- | --- | --- | --- | --- | --- | --- | --- | --- | --- | --- | --- | --- | --- | --- | --- | --- | --- | --- | --- | --- | --- | --- | --- | --- | --- | --- | --- | --- | --- | --- | --- | --- | --- | --- | --- | --- | --- | --- | --- | --- | --- | --- | --- | --- | --- | --- | --- | --- | --- | --- | --- | --- | --- | --- | --- | --- | --- | --- | --- | --- | --- | --- | --- | --- | --- | --- | --- | --- | --- | --- | --- | --- | --- | --- | --- | --- | --- | --- | --- | --- | --- | --- | --- | --- | --- | --- | --- | --- | --- | --- | --- | --- | --- | --- | --- | --- | --- | --- | --- | --- | --- | --- | --- | --- | --- | --- | --- | --- | --- | --- | --- | --- | --- | --- | --- | --- | --- | --- | --- | --- | --- | --- | --- | --- | --- | --- | --- | --- | --- | | | --- | --- | --- | --- | --- | --- | --- | --- | --- | --- | --- | --- | --- | --- | --- | --- | --- | --- | --- | --- | --- | --- | --- | --- | --- | --- | --- | --- | --- | --- | --- | --- | --- | --- | --- | --- | --- | --- | --- | --- | --- | --- | --- | --- | --- | --- | --- | --- | --- | --- | --- | --- | --- | --- | --- | --- | --- | --- | --- | --- | --- | --- | --- | --- | --- | --- | --- | --- | --- | --- | --- | --- | --- | --- | --- | --- | --- | --- | --- | --- | --- | --- | --- | --- | --- | --- | --- | --- | --- | --- | --- | --- | --- | --- | --- | --- | --- | --- | --- | --- | --- | --- | --- | --- | --- | --- | --- | --- | --- | --- | --- | --- | --- | --- | --- | --- | --- | --- | --- | --- | --- | --- | --- | --- | --- | --- | --- | --- | --- | --- | --- | --- | --- | --- | --- | --- | --- | --- | --- | --- | --- | --- | --- | |
| --- | --- | --- | --- | --- | --- | --- | --- | --- | --- | --- | --- | --- | --- | --- | --- | --- | --- | --- | --- | --- | --- | --- | --- | --- | --- | --- | --- | --- | --- | --- | --- | --- | --- | --- | --- | --- | --- | --- | --- | --- | --- | --- | --- | --- | --- | --- | --- | --- | --- | --- | --- | --- | --- | --- | --- | --- | --- | --- | --- | --- | --- | --- | --- | --- | --- | --- | --- | --- | --- | --- | --- | --- | --- | --- | --- | --- | --- | --- | --- | --- | --- | --- | --- | --- | --- | --- | --- | --- | --- | --- | --- | --- | --- | --- | --- | --- | --- | --- | --- | --- | --- | --- | --- | --- | --- | --- | --- | --- | --- | --- | --- | --- | --- | --- | --- | --- | --- | --- | --- | --- | --- | --- | --- | --- | --- | --- | --- | --- | --- | --- | --- | --- | --- | --- | --- | --- | --- | --- | --- | --- | --- | --- | --- |
| |  | | --- | |

23.

| | **Your Input:** **PF3D7_0912400** | | |  |  |  |  |  |  |  |  |  | | --- | --- | --- | --- | --- | --- | --- | --- | --- | --- | --- | --- | |  | [PFI0605c](http://string-db.org/newstring_cgi/display_single_node.pl?taskId=wS4_tNis96o2&node=401732&targetmode=proteins) | hypothetical protein, conserved (446 aa) | |  |  | *(Plasmodium falciparum)* | | **Predicted Functional Partners:** | | | |  | [GTP-CH](http://string-db.org/newstring_cgi/display_single_node.pl?taskId=wS4_tNis96o2&node=402221&targetmode=proteins) | GTP cyclohydrolase I (389 aa) |  |  |  | [**•**](http://string-db.org/newstring_cgi/show_coexpression_evidence.pl?taskId=wS4_tNis96o2&node2=402221) |  | [**•**](http://string-db.org/newstring_cgi/show_set_evidence.pl?data_channel=database&taskId=wS4_tNis96o2&node2=402221) |  |  | 0.904 | |  | [PTPS](http://string-db.org/newstring_cgi/display_single_node.pl?taskId=wS4_tNis96o2&node=401559&targetmode=proteins) | 6-pyruvoyl tetrahydropterin synthase, putative (173 aa) |  |  |  |  |  | [**•**](http://string-db.org/newstring_cgi/show_set_evidence.pl?data_channel=database&taskId=wS4_tNis96o2&node2=401559) |  |  | 0.899 | |  | [MAL8P1.69](http://string-db.org/newstring_cgi/display_single_node.pl?taskId=wS4_tNis96o2&node=397670&targetmode=proteins) | 14-3-3 protein homologue, putative (256 aa) |  |  |  | [**•**](http://string-db.org/newstring_cgi/show_coexpression_evidence.pl?taskId=wS4_tNis96o2&node2=397670) |  |  |  |  | 0.547 | |  | [PF13_0349](http://string-db.org/newstring_cgi/display_single_node.pl?taskId=wS4_tNis96o2&node=399256&targetmode=proteins) | nucleoside diphosphate kinase b%3B putative (149 aa) | [**•**](http://string-db.org/newstring_cgi/show_neighborhood.pl?taskId=wS4_tNis96o2&node2=399256) |  |  | [**•**](http://string-db.org/newstring_cgi/show_coexpression_evidence.pl?taskId=wS4_tNis96o2&node2=399256) |  |  |  |  | 0.473 | |  | [PFI1545c](http://string-db.org/newstring_cgi/display_single_node.pl?taskId=wS4_tNis96o2&node=401924&targetmode=proteins) | proteasome precursor, putative (282 aa) |  |  |  | [**•**](http://string-db.org/newstring_cgi/show_coexpression_evidence.pl?taskId=wS4_tNis96o2&node2=401924) |  |  |  |  | 0.458 | |
| --- | --- | --- | --- | --- | --- | --- | --- | --- | --- | --- | --- | --- | --- | --- | --- | --- | --- | --- | --- | --- | --- | --- | --- | --- | --- | --- | --- | --- | --- | --- | --- | --- | --- | --- | --- | --- | --- | --- | --- | --- | --- | --- | --- | --- | --- | --- | --- | --- | --- | --- | --- | --- | --- | --- | --- | --- | --- | --- | --- | --- | --- | --- | --- | --- | --- | --- | --- | --- | --- | --- | --- | --- | --- | --- | --- | --- | --- | --- | --- | --- | --- |

24.

| | | | **Your Input:** **PF3D7_0918000** | | |  |  |  |  |  |  |  |  |  | | --- | --- | --- | --- | --- | --- | --- | --- | --- | --- | --- | --- | |  | [GAP50](http://string-db.org/newstring_cgi/display_single_node.pl?taskId=JeXqyrvubnA6&node=401788&targetmode=proteins) | acid phosphatase, putative (396 aa) | |  |  | *(Plasmodium falciparum)* | | **Predicted Functional Partners:** | | | |  | [GAP45](http://string-db.org/newstring_cgi/display_single_node.pl?taskId=JeXqyrvubnA6&node=402208&targetmode=proteins) | gliding-associated protein 45, GAP45 putative (204 aa) |  |  |  | [**•**](http://string-db.org/newstring_cgi/show_coexpression_evidence.pl?taskId=JeXqyrvubnA6&node2=402208) |  |  | [**•**](http://string-db.org/newstring_cgi/show_textmining_evidence.pl?taskId=JeXqyrvubnA6&node2=402208) |  | 0.992 | |  | [PFL2215w](http://string-db.org/newstring_cgi/display_single_node.pl?taskId=JeXqyrvubnA6&node=402438&targetmode=proteins) | actin; Actins are highly conserved proteins that are involved in various types of cell motility [...] (376 aa) |  |  |  | [**•**](http://string-db.org/newstring_cgi/show_coexpression_evidence.pl?taskId=JeXqyrvubnA6&node2=402438) |  |  | [**•**](http://string-db.org/newstring_cgi/show_textmining_evidence.pl?taskId=JeXqyrvubnA6&node2=402438) |  | 0.975 | |  | [RhopH2](http://string-db.org/newstring_cgi/display_single_node.pl?taskId=JeXqyrvubnA6&node=401903&targetmode=proteins) | High molecular weight rhoptry protein-2 (1378 aa) |  |  |  | [**•**](http://string-db.org/newstring_cgi/show_coexpression_evidence.pl?taskId=JeXqyrvubnA6&node2=401903) |  |  | [**•**](http://string-db.org/newstring_cgi/show_textmining_evidence.pl?taskId=JeXqyrvubnA6&node2=401903) |  | 0.965 | |  | [Pf92](http://string-db.org/newstring_cgi/display_single_node.pl?taskId=JeXqyrvubnA6&node=399245&targetmode=proteins) | cysteine-rich surface protein (796 aa) |  |  |  | [**•**](http://string-db.org/newstring_cgi/show_coexpression_evidence.pl?taskId=JeXqyrvubnA6&node2=399245) |  |  | [**•**](http://string-db.org/newstring_cgi/show_textmining_evidence.pl?taskId=JeXqyrvubnA6&node2=399245) |  | 0.965 | |  | [PFI0975c](http://string-db.org/newstring_cgi/display_single_node.pl?taskId=JeXqyrvubnA6&node=401807&targetmode=proteins) | hypothetical protein, conserved (3381 aa) |  |  |  | [**•**](http://string-db.org/newstring_cgi/show_coexpression_evidence.pl?taskId=JeXqyrvubnA6&node2=401807) |  |  |  |  | 0.960 | |  | [PFI0160w](http://string-db.org/newstring_cgi/display_single_node.pl?taskId=JeXqyrvubnA6&node=401641&targetmode=proteins) | hypothetical protein, conserved (3135 aa) |  |  |  | [**•**](http://string-db.org/newstring_cgi/show_coexpression_evidence.pl?taskId=JeXqyrvubnA6&node2=401641) |  |  |  |  | 0.960 | |  | [PFF0670w-1](http://string-db.org/newstring_cgi/display_single_node.pl?taskId=JeXqyrvubnA6&node=401418&targetmode=proteins) | hypothetical protein, conserved (4095 aa) |  |  |  | [**•**](http://string-db.org/newstring_cgi/show_coexpression_evidence.pl?taskId=JeXqyrvubnA6&node2=401418) |  |  |  |  | 0.960 | |  | [PFE0785c](http://string-db.org/newstring_cgi/display_single_node.pl?taskId=JeXqyrvubnA6&node=401115&targetmode=proteins) | drug%2Fmetabolite transporter (456 aa) |  |  |  | [**•**](http://string-db.org/newstring_cgi/show_coexpression_evidence.pl?taskId=JeXqyrvubnA6&node2=401115) |  |  |  |  | 0.960 | |  | [AQP](http://string-db.org/newstring_cgi/display_single_node.pl?taskId=JeXqyrvubnA6&node=398729&targetmode=proteins) | Aquaglyceroporin (258 aa) |  |  |  | [**•**](http://string-db.org/newstring_cgi/show_coexpression_evidence.pl?taskId=JeXqyrvubnA6&node2=398729) |  |  |  |  | 0.958 | |  | [PF11_0300](http://string-db.org/newstring_cgi/display_single_node.pl?taskId=JeXqyrvubnA6&node=398690&targetmode=proteins) | hypothetical protein (642 aa) |  |  |  | [**•**](http://string-db.org/newstring_cgi/show_coexpression_evidence.pl?taskId=JeXqyrvubnA6&node2=398690) |  |  |  |  | 0.958 | |  | [Pfn](http://string-db.org/newstring_cgi/display_single_node.pl?taskId=JeXqyrvubnA6&node=401928&targetmode=proteins) | conserved protein; Essential for the invasive blood stages of the parasite. Binds to proline ri [...] (171 aa) |  |  |  | [**•**](http://string-db.org/newstring_cgi/show_coexpression_evidence.pl?taskId=JeXqyrvubnA6&node2=401928) |  |  |  |  | 0.954 | |  | [PF10_0330](http://string-db.org/newstring_cgi/display_single_node.pl?taskId=JeXqyrvubnA6&node=398325&targetmode=proteins) | ubiquitin-conjugating enzyme, putative (191 aa) |  |  |  | [**•**](http://string-db.org/newstring_cgi/show_coexpression_evidence.pl?taskId=JeXqyrvubnA6&node2=398325) |  |  |  |  | 0.952 | |  | [PFD1110w](http://string-db.org/newstring_cgi/display_single_node.pl?taskId=JeXqyrvubnA6&node=400931&targetmode=proteins) | hypothetical membrane protein, conserved (372 aa) |  |  |  | [**•**](http://string-db.org/newstring_cgi/show_coexpression_evidence.pl?taskId=JeXqyrvubnA6&node2=400931) |  |  | [**•**](http://string-db.org/newstring_cgi/show_textmining_evidence.pl?taskId=JeXqyrvubnA6&node2=400931) |  | 0.948 | |  | [PFL2505c](http://string-db.org/newstring_cgi/display_single_node.pl?taskId=JeXqyrvubnA6&node=402494&targetmode=proteins) | hypothetical protein, conserved (2215 aa) |  |  |  | [**•**](http://string-db.org/newstring_cgi/show_coexpression_evidence.pl?taskId=JeXqyrvubnA6&node2=402494) |  |  |  |  | 0.944 | |  | [RON4](http://string-db.org/newstring_cgi/display_single_node.pl?taskId=JeXqyrvubnA6&node=398555&targetmode=proteins) | hypothetical protein (2966 aa) |  |  |  | [**•**](http://string-db.org/newstring_cgi/show_coexpression_evidence.pl?taskId=JeXqyrvubnA6&node2=398555) |  |  | [**•**](http://string-db.org/newstring_cgi/show_textmining_evidence.pl?taskId=JeXqyrvubnA6&node2=398555) |  | 0.936 | |  | [PFL1160c](http://string-db.org/newstring_cgi/display_single_node.pl?taskId=JeXqyrvubnA6&node=402222&targetmode=proteins) | hypothetical protein, conserved (192 aa) |  |  |  | [**•**](http://string-db.org/newstring_cgi/show_coexpression_evidence.pl?taskId=JeXqyrvubnA6&node2=402222) |  |  |  |  | 0.935 | |  | [PFD0660w](http://string-db.org/newstring_cgi/display_single_node.pl?taskId=JeXqyrvubnA6&node=400832&targetmode=proteins) | phosphoglycerate mutase, putative (295 aa) |  |  |  | [**•**](http://string-db.org/newstring_cgi/show_coexpression_evidence.pl?taskId=JeXqyrvubnA6&node2=400832) |  |  |  |  | 0.935 | |  | [PfPDI-9](http://string-db.org/newstring_cgi/display_single_node.pl?taskId=JeXqyrvubnA6&node=401802&targetmode=proteins) | protein disulfide isomerase, putative (515 aa) |  |  |  | [**•**](http://string-db.org/newstring_cgi/show_coexpression_evidence.pl?taskId=JeXqyrvubnA6&node2=401802) |  |  |  |  | 0.927 | |  | [PF14_0492](http://string-db.org/newstring_cgi/display_single_node.pl?taskId=JeXqyrvubnA6&node=399770&targetmode=proteins) | protein phosphatase 2b regulatory subunit, putative (179 aa) |  |  |  | [**•**](http://string-db.org/newstring_cgi/show_coexpression_evidence.pl?taskId=JeXqyrvubnA6&node2=399770) |  |  |  |  | 0.925 | |  | [MTIP](http://string-db.org/newstring_cgi/display_single_node.pl?taskId=JeXqyrvubnA6&node=402440&targetmode=proteins) | myosin A tail domain interacting protein MTIP, putative (204 aa) |  |  |  | [**•**](http://string-db.org/newstring_cgi/show_coexpression_evidence.pl?taskId=JeXqyrvubnA6&node2=402440) |  |  | [**•**](http://string-db.org/newstring_cgi/show_textmining_evidence.pl?taskId=JeXqyrvubnA6&node2=402440) |  | 0.923 | |  | [MAL13P1.215-1](http://string-db.org/newstring_cgi/display_single_node.pl?taskId=JeXqyrvubnA6&node=397084&targetmode=proteins) | hypothetical protein, conserved (315 aa) |  |  |  | [**•**](http://string-db.org/newstring_cgi/show_coexpression_evidence.pl?taskId=JeXqyrvubnA6&node2=397084) |  |  |  |  | 0.921 | |  | [PDEbeta](http://string-db.org/newstring_cgi/display_single_node.pl?taskId=JeXqyrvubnA6&node=396982&targetmode=proteins) | cAMP-specific 3',5'-cyclic phosphodiesterase 4D, putative (1139 aa) |  |  |  | [**•**](http://string-db.org/newstring_cgi/show_coexpression_evidence.pl?taskId=JeXqyrvubnA6&node2=396982) |  |  |  |  | 0.917 | |  | [PKG](http://string-db.org/newstring_cgi/display_single_node.pl?taskId=JeXqyrvubnA6&node=399622&targetmode=proteins) | cGMP-dependent protein kinase 1, beta isozyme, putative (853 aa) |  |  |  | [**•**](http://string-db.org/newstring_cgi/show_coexpression_evidence.pl?taskId=JeXqyrvubnA6&node2=399622) |  |  | [**•**](http://string-db.org/newstring_cgi/show_textmining_evidence.pl?taskId=JeXqyrvubnA6&node2=399622) |  | 0.913 | |  | [MAL7P1.17](http://string-db.org/newstring_cgi/display_single_node.pl?taskId=JeXqyrvubnA6&node=397405&targetmode=proteins) | hypothetical membrane protein, conserved (3559 aa) |  |  |  | [**•**](http://string-db.org/newstring_cgi/show_coexpression_evidence.pl?taskId=JeXqyrvubnA6&node2=397405) |  |  |  |  | 0.911 | |  | [MSP1](http://string-db.org/newstring_cgi/display_single_node.pl?taskId=JeXqyrvubnA6&node=401910&targetmode=proteins) | merozoite surface protein 1, precursor (1720 aa) |  |  |  | [**•**](http://string-db.org/newstring_cgi/show_coexpression_evidence.pl?taskId=JeXqyrvubnA6&node2=401910) |  |  | [**•**](http://string-db.org/newstring_cgi/show_textmining_evidence.pl?taskId=JeXqyrvubnA6&node2=401910) |  | 0.905 | |  | [PF13_0233](http://string-db.org/newstring_cgi/display_single_node.pl?taskId=JeXqyrvubnA6&node=399146&targetmode=proteins) | myosin a; Myosins are actin-based motor molecules with ATPase activity. Unconventional myosins [...] (818 aa) |  |  |  |  |  |  | [**•**](http://string-db.org/newstring_cgi/show_textmining_evidence.pl?taskId=JeXqyrvubnA6&node2=399146) |  | 0.903 | |  | [PF10_0147](http://string-db.org/newstring_cgi/display_single_node.pl?taskId=JeXqyrvubnA6&node=398131&targetmode=proteins) | FAD synthetase, putative (328 aa) |  |  |  |  |  | [**•**](http://string-db.org/newstring_cgi/show_set_evidence.pl?data_channel=database&taskId=JeXqyrvubnA6&node2=398131) |  |  | 0.899 | |  | [MAL13P1.292-1](http://string-db.org/newstring_cgi/display_single_node.pl?taskId=JeXqyrvubnA6&node=397167&targetmode=proteins) | riboflavin kinase %2F FAD synthase family protein, putative (707 aa) |  |  |  |  |  | [**•**](http://string-db.org/newstring_cgi/show_set_evidence.pl?data_channel=database&taskId=JeXqyrvubnA6&node2=397167) |  |  | 0.899 | |  | [MAL13P1.130-1](http://string-db.org/newstring_cgi/display_single_node.pl?taskId=JeXqyrvubnA6&node=396996&targetmode=proteins) | hypothetical protein, conserved (303 aa) |  |  |  | [**•**](http://string-db.org/newstring_cgi/show_coexpression_evidence.pl?taskId=JeXqyrvubnA6&node2=396996) |  |  | [**•**](http://string-db.org/newstring_cgi/show_textmining_evidence.pl?taskId=JeXqyrvubnA6&node2=396996) |  | 0.897 | |  | [PF10_0020](http://string-db.org/newstring_cgi/display_single_node.pl?taskId=JeXqyrvubnA6&node=397998&targetmode=proteins) | hypothetical protein (763 aa) |  |  |  | [**•**](http://string-db.org/newstring_cgi/show_coexpression_evidence.pl?taskId=JeXqyrvubnA6&node2=397998) |  |  |  |  | 0.897 | |  | [SERA-6](http://string-db.org/newstring_cgi/display_single_node.pl?taskId=JeXqyrvubnA6&node=400296&targetmode=proteins) | cysteine protease, putative (893 aa) |  |  |  | [**•**](http://string-db.org/newstring_cgi/show_coexpression_evidence.pl?taskId=JeXqyrvubnA6&node2=400296) |  |  |  |  | 0.893 | |  | [CPK1](http://string-db.org/newstring_cgi/display_single_node.pl?taskId=JeXqyrvubnA6&node=400394&targetmode=proteins) | Pf Calcium-dependent protein kinase 1; Calcium-dependent kinase which may phosphorylate protein [...] (524 aa) |  |  |  | [**•**](http://string-db.org/newstring_cgi/show_coexpression_evidence.pl?taskId=JeXqyrvubnA6&node2=400394) |  |  | [**•**](http://string-db.org/newstring_cgi/show_textmining_evidence.pl?taskId=JeXqyrvubnA6&node2=400394) |  | 0.891 | |  | [PFL1870c](http://string-db.org/newstring_cgi/display_single_node.pl?taskId=JeXqyrvubnA6&node=402367&targetmode=proteins) | sphingomyelin phosphodiesterase (393 aa) |  |  |  | [**•**](http://string-db.org/newstring_cgi/show_coexpression_evidence.pl?taskId=JeXqyrvubnA6&node2=402367) |  |  |  |  | 0.883 | |  | [MAL8P1.153-1](http://string-db.org/newstring_cgi/display_single_node.pl?taskId=JeXqyrvubnA6&node=397576&targetmode=proteins) | hypothetical protein, conserved (2577 aa) |  |  |  | [**•**](http://string-db.org/newstring_cgi/show_coexpression_evidence.pl?taskId=JeXqyrvubnA6&node2=397576) |  |  |  |  | 0.879 | |  | [ABRA](http://string-db.org/newstring_cgi/display_single_node.pl?taskId=JeXqyrvubnA6&node=402267&targetmode=proteins) | Merozoite Surface Protein 9, MSP-9 (743 aa) |  |  |  | [**•**](http://string-db.org/newstring_cgi/show_coexpression_evidence.pl?taskId=JeXqyrvubnA6&node2=402267) |  |  |  |  | 0.873 | |  | [RAP2](http://string-db.org/newstring_cgi/display_single_node.pl?taskId=JeXqyrvubnA6&node=400974&targetmode=proteins) | rhoptry-associated protein 2, RAP2 (398 aa) |  |  |  | [**•**](http://string-db.org/newstring_cgi/show_coexpression_evidence.pl?taskId=JeXqyrvubnA6&node2=400974) |  |  |  |  | 0.863 | |  | [Pfcrk-4](http://string-db.org/newstring_cgi/display_single_node.pl?taskId=JeXqyrvubnA6&node=400623&targetmode=proteins) | protein kinase, predicted (1553 aa) |  |  |  | [**•**](http://string-db.org/newstring_cgi/show_coexpression_evidence.pl?taskId=JeXqyrvubnA6&node2=400623) |  |  |  |  | 0.863 | |  | [MAL8P1.146](http://string-db.org/newstring_cgi/display_single_node.pl?taskId=JeXqyrvubnA6&node=397568&targetmode=proteins) | filament assembling protein, putative (728 aa) |  |  |  | [**•**](http://string-db.org/newstring_cgi/show_coexpression_evidence.pl?taskId=JeXqyrvubnA6&node2=397568) |  |  |  |  | 0.863 | |  | [PFF0595c](http://string-db.org/newstring_cgi/display_single_node.pl?taskId=JeXqyrvubnA6&node=401403&targetmode=proteins) | leucine-rich repeat protein 5, LRR5 (1864 aa) |  |  |  | [**•**](http://string-db.org/newstring_cgi/show_coexpression_evidence.pl?taskId=JeXqyrvubnA6&node2=401403) |  |  |  |  | 0.861 | |  | [PF14_0681](http://string-db.org/newstring_cgi/display_single_node.pl?taskId=JeXqyrvubnA6&node=399966&targetmode=proteins) | diacylglycerol kinase, putative (488 aa) |  |  |  | [**•**](http://string-db.org/newstring_cgi/show_coexpression_evidence.pl?taskId=JeXqyrvubnA6&node2=399966) |  |  |  |  | 0.859 | |  | [PFL0580w](http://string-db.org/newstring_cgi/display_single_node.pl?taskId=JeXqyrvubnA6&node=402101&targetmode=proteins) | DNA replication licensing factor mcm5, putative (758 aa) |  |  |  | [**•**](http://string-db.org/newstring_cgi/show_coexpression_evidence.pl?taskId=JeXqyrvubnA6&node2=402101) |  |  |  |  | 0.857 | |  | [MAL7P1.18](http://string-db.org/newstring_cgi/display_single_node.pl?taskId=JeXqyrvubnA6&node=397415&targetmode=proteins) | serine%2Fthreonine protein kinase, putative (1603 aa) |  |  |  | [**•**](http://string-db.org/newstring_cgi/show_coexpression_evidence.pl?taskId=JeXqyrvubnA6&node2=397415) |  |  |  |  | 0.857 | |  | [PFB0190c](http://string-db.org/newstring_cgi/display_single_node.pl?taskId=JeXqyrvubnA6&node=400262&targetmode=proteins) | hypothetical protein (2295 aa) |  |  |  | [**•**](http://string-db.org/newstring_cgi/show_coexpression_evidence.pl?taskId=JeXqyrvubnA6&node2=400262) |  |  |  |  | 0.857 | |  | [PF14_0090](http://string-db.org/newstring_cgi/display_single_node.pl?taskId=JeXqyrvubnA6&node=399359&targetmode=proteins) | hypothetical protein (382 aa) |  |  |  | [**•**](http://string-db.org/newstring_cgi/show_coexpression_evidence.pl?taskId=JeXqyrvubnA6&node2=399359) |  |  |  |  | 0.855 | |  | [PF13_0299](http://string-db.org/newstring_cgi/display_single_node.pl?taskId=JeXqyrvubnA6&node=399210&targetmode=proteins) | hypothetical protein, conserved (964 aa) |  |  |  | [**•**](http://string-db.org/newstring_cgi/show_coexpression_evidence.pl?taskId=JeXqyrvubnA6&node2=399210) |  |  |  |  | 0.855 | |  | [MAL13P1.308-1](http://string-db.org/newstring_cgi/display_single_node.pl?taskId=JeXqyrvubnA6&node=397184&targetmode=proteins) | hypothetical protein, conserved (2605 aa) |  |  |  | [**•**](http://string-db.org/newstring_cgi/show_coexpression_evidence.pl?taskId=JeXqyrvubnA6&node2=397184) |  |  |  |  | 0.855 | |  | [PFE0165w](http://string-db.org/newstring_cgi/display_single_node.pl?taskId=JeXqyrvubnA6&node=400991&targetmode=proteins) | actin depolymerizing factor, putative; Not involved in actin polymerisation, instead functions [...] (122 aa) |  |  |  | [**•**](http://string-db.org/newstring_cgi/show_coexpression_evidence.pl?taskId=JeXqyrvubnA6&node2=400991) |  |  |  |  | 0.855 | |  | [PFD0940w](http://string-db.org/newstring_cgi/display_single_node.pl?taskId=JeXqyrvubnA6&node=400891&targetmode=proteins) | hypothetical protein, conserved (2472 aa) |  |  |  | [**•**](http://string-db.org/newstring_cgi/show_coexpression_evidence.pl?taskId=JeXqyrvubnA6&node2=400891) |  |  |  |  | 0.855 | |  | [PFC0435w](http://string-db.org/newstring_cgi/display_single_node.pl?taskId=JeXqyrvubnA6&node=400557&targetmode=proteins) | hypothetical protein, conserved (1294 aa) |  |  |  | [**•**](http://string-db.org/newstring_cgi/show_coexpression_evidence.pl?taskId=JeXqyrvubnA6&node2=400557) |  |  |  |  | 0.855 | |  | [PF11_0443](http://string-db.org/newstring_cgi/display_single_node.pl?taskId=JeXqyrvubnA6&node=398834&targetmode=proteins) | hypothetical protein, conserved (321 aa) |  |  |  | [**•**](http://string-db.org/newstring_cgi/show_coexpression_evidence.pl?taskId=JeXqyrvubnA6&node2=398834) |  |  |  |  | 0.855 | | | --- | --- | --- | --- | --- | --- | --- | --- | --- | --- | --- | --- | --- | --- | --- | --- | --- | --- | --- | --- | --- | --- | --- | --- | --- | --- | --- | --- | --- | --- | --- | --- | --- | --- | --- | --- | --- | --- | --- | --- | --- | --- | --- | --- | --- | --- | --- | --- | --- | --- | --- | --- | --- | --- | --- | --- | --- | --- | --- | --- | --- | --- | --- | --- | --- | --- | --- | --- | --- | --- | --- | --- | --- | --- | --- | --- | --- | --- | --- | --- | --- | --- | --- | --- | --- | --- | --- | --- | --- | --- | --- | --- | --- | --- | --- | --- | --- | --- | --- | --- | --- | --- | --- | --- | --- | --- | --- | --- | --- | --- | --- | --- | --- | --- | --- | --- | --- | --- | --- | --- | --- | --- | --- | --- | --- | --- | --- | --- | --- | --- | --- | --- | --- | --- | --- | --- | --- | --- | --- | --- | --- | --- | --- | --- | --- | --- | --- | --- | --- | --- | --- | --- | --- | --- | --- | --- | --- | --- | --- | --- | --- | --- | --- | --- | --- | --- | --- | --- | --- | --- | --- | --- | --- | --- | --- | --- | --- | --- | --- | --- | --- | --- | --- | --- | --- | --- | --- | --- | --- | --- | --- | --- | --- | --- | --- | --- | --- | --- | --- | --- | --- | --- | --- | --- | --- | --- | --- | --- | --- | --- | --- | --- | --- | --- | --- | --- | --- | --- | --- | --- | --- | --- | --- | --- | --- | --- | --- | --- | --- | --- | --- | --- | --- | --- | --- | --- | --- | --- | --- | --- | --- | --- | --- | --- | --- | --- | --- | --- | --- | --- | --- | --- | --- | --- | --- | --- | --- | --- | --- | --- | --- | --- | --- | --- | --- | --- | --- | --- | --- | --- | --- | --- | --- | --- | --- | --- | --- | --- | --- | --- | --- | --- | --- | --- | --- | --- | --- | --- | --- | --- | --- | --- | --- | --- | --- | --- | --- | --- | --- | --- | --- | --- | --- | --- | --- | --- | --- | --- | --- | --- | --- | --- | --- | --- | --- | --- | --- | --- | --- | --- | --- | --- | --- | --- | --- | --- | --- | --- | --- | --- | --- | --- | --- | --- | --- | --- | --- | --- | --- | --- | --- | --- | --- | --- | --- | --- | --- | --- | --- | --- | --- | --- | --- | --- | --- | --- | --- | --- | --- | --- | --- | --- | --- | --- | --- | --- | --- | --- | --- | --- | --- | --- | --- | --- | --- | --- | --- | --- | --- | --- | --- | --- | --- | --- | --- | --- | --- | --- | --- | --- | --- | --- | --- | --- | --- | --- | --- | --- | --- | --- | --- | --- | --- | --- | --- | --- | --- | --- | --- | --- | --- | --- | --- | --- | --- | --- | --- | --- | --- | --- | --- | --- | --- | --- | --- | --- | --- | --- | --- | --- | --- | --- | --- | --- | --- | --- | --- | --- | --- | --- | --- | --- | --- | --- | --- | --- | --- | --- | --- | --- | --- | --- | --- | --- | --- | --- | --- | --- | --- | --- | --- | --- | --- | --- | --- | --- | --- | --- | --- | --- | --- | --- | --- | --- | --- | --- | --- | --- | --- | --- | --- | --- | --- | --- | --- | --- | --- | --- | --- | --- | --- | --- | --- | --- | --- | --- | --- | --- | --- | --- | --- | --- | --- | --- | --- | --- | --- | --- | --- | --- | --- | --- | --- | --- | --- | --- | --- | --- | --- | --- | --- | --- | --- | --- | --- | --- | --- | --- | --- | --- | --- | --- | --- | --- | --- | --- | --- | --- | --- | --- | --- | --- | --- | --- | --- | --- | --- | --- | --- | --- | --- | --- | --- | --- | --- | --- | --- | --- | --- | --- | --- | --- | --- | --- | --- | --- | --- | --- | --- | --- | --- | --- | --- | --- | --- | --- | --- | --- | --- | --- | --- | --- | --- | --- | --- | --- | --- | --- | --- | --- | --- | --- | --- | --- | --- | --- | --- | --- | --- | --- | --- | --- | --- | --- | --- | --- | --- | --- | --- | --- | --- | --- | --- | --- | --- | --- | --- | --- | --- | --- | --- | --- | | | --- | --- | --- | --- | --- | --- | --- | --- | --- | --- | --- | --- | --- | --- | --- | --- | --- | --- | --- | --- | --- | --- | --- | --- | --- | --- | --- | --- | --- | --- | --- | --- | --- | --- | --- | --- | --- | --- | --- | --- | --- | --- | --- | --- | --- | --- | --- | --- | --- | --- | --- | --- | --- | --- | --- | --- | --- | --- | --- | --- | --- | --- | --- | --- | --- | --- | --- | --- | --- | --- | --- | --- | --- | --- | --- | --- | --- | --- | --- | --- | --- | --- | --- | --- | --- | --- | --- | --- | --- | --- | --- | --- | --- | --- | --- | --- | --- | --- | --- | --- | --- | --- | --- | --- | --- | --- | --- | --- | --- | --- | --- | --- | --- | --- | --- | --- | --- | --- | --- | --- | --- | --- | --- | --- | --- | --- | --- | --- | --- | --- | --- | --- | --- | --- | --- | --- | --- | --- | --- | --- | --- | --- | --- | --- | --- | --- | --- | --- | --- | --- | --- | --- | --- | --- | --- | --- | --- | --- | --- | --- | --- | --- | --- | --- | --- | --- | --- | --- | --- | --- | --- | --- | --- | --- | --- | --- | --- | --- | --- | --- | --- | --- | --- | --- | --- | --- | --- | --- | --- | --- | --- | --- | --- | --- | --- | --- | --- | --- | --- | --- | --- | --- | --- | --- | --- | --- | --- | --- | --- | --- | --- | --- | --- | --- | --- | --- | --- | --- | --- | --- | --- | --- | --- | --- | --- | --- | --- | --- | --- | --- | --- | --- | --- | --- | --- | --- | --- | --- | --- | --- | --- | --- | --- | --- | --- | --- | --- | --- | --- | --- | --- | --- | --- | --- | --- | --- | --- | --- | --- | --- | --- | --- | --- | --- | --- | --- | --- | --- | --- | --- | --- | --- | --- | --- | --- | --- | --- | --- | --- | --- | --- | --- | --- | --- | --- | --- | --- | --- | --- | --- | --- | --- | --- | --- | --- | --- | --- | --- | --- | --- | --- | --- | --- | --- | --- | --- | --- | --- | --- | --- | --- | --- | --- | --- | --- | --- | --- | --- | --- | --- | --- | --- | --- | --- | --- | --- | --- | --- | --- | --- | --- | --- | --- | --- | --- | --- | --- | --- | --- | --- | --- | --- | --- | --- | --- | --- | --- | --- | --- | --- | --- | --- | --- | --- | --- | --- | --- | --- | --- | --- | --- | --- | --- | --- | --- | --- | --- | --- | --- | --- | --- | --- | --- | --- | --- | --- | --- | --- | --- | --- | --- | --- | --- | --- | --- | --- | --- | --- | --- | --- | --- | --- | --- | --- | --- | --- | --- | --- | --- | --- | --- | --- | --- | --- | --- | --- | --- | --- | --- | --- | --- | --- | --- | --- | --- | --- | --- | --- | --- | --- | --- | --- | --- | --- | --- | --- | --- | --- | --- | --- | --- | --- | --- | --- | --- | --- | --- | --- | --- | --- | --- | --- | --- | --- | --- | --- | --- | --- | --- | --- | --- | --- | --- | --- | --- | --- | --- | --- | --- | --- | --- | --- | --- | --- | --- | --- | --- | --- | --- | --- | --- | --- | --- | --- | --- | --- | --- | --- | --- | --- | --- | --- | --- | --- | --- | --- | --- | --- | --- | --- | --- | --- | --- | --- | --- | --- | --- | --- | --- | --- | --- | --- | --- | --- | --- | --- | --- | --- | --- | --- | --- | --- | --- | --- | --- | --- | --- | --- | --- | --- | --- | --- | --- | --- | --- | --- | --- | --- | --- | --- | --- | --- | --- | --- | --- | --- | --- | --- | --- | --- | --- | --- | --- | --- | --- | --- | --- | --- | --- | --- | --- | --- | --- | --- | --- | --- | --- | --- | --- | --- | --- | --- | --- | --- | --- | --- | --- | --- | --- | --- | --- | --- | --- | --- | --- | --- | --- | --- | --- | --- | --- | --- | --- | --- | --- | --- | --- | --- | --- | --- | --- | --- | --- | --- | --- | --- | --- | --- | --- | --- | --- | --- | --- | --- | --- | --- | --- | --- | --- | --- | --- | --- | --- | --- | --- | --- | --- | --- | --- | --- | --- | --- | --- | |
| --- | --- | --- | --- | --- | --- | --- | --- | --- | --- | --- | --- | --- | --- | --- | --- | --- | --- | --- | --- | --- | --- | --- | --- | --- | --- | --- | --- | --- | --- | --- | --- | --- | --- | --- | --- | --- | --- | --- | --- | --- | --- | --- | --- | --- | --- | --- | --- | --- | --- | --- | --- | --- | --- | --- | --- | --- | --- | --- | --- | --- | --- | --- | --- | --- | --- | --- | --- | --- | --- | --- | --- | --- | --- | --- | --- | --- | --- | --- | --- | --- | --- | --- | --- | --- | --- | --- | --- | --- | --- | --- | --- | --- | --- | --- | --- | --- | --- | --- | --- | --- | --- | --- | --- | --- | --- | --- | --- | --- | --- | --- | --- | --- | --- | --- | --- | --- | --- | --- | --- | --- | --- | --- | --- | --- | --- | --- | --- | --- | --- | --- | --- | --- | --- | --- | --- | --- | --- | --- | --- | --- | --- | --- | --- | --- | --- | --- | --- | --- | --- | --- | --- | --- | --- | --- | --- | --- | --- | --- | --- | --- | --- | --- | --- | --- | --- | --- | --- | --- | --- | --- | --- | --- | --- | --- | --- | --- | --- | --- | --- | --- | --- | --- | --- | --- | --- | --- | --- | --- | --- | --- | --- | --- | --- | --- | --- | --- | --- | --- | --- | --- | --- | --- | --- | --- | --- | --- | --- | --- | --- | --- | --- | --- | --- | --- | --- | --- | --- | --- | --- | --- | --- | --- | --- | --- | --- | --- | --- | --- | --- | --- | --- | --- | --- | --- | --- | --- | --- | --- | --- | --- | --- | --- | --- | --- | --- | --- | --- | --- | --- | --- | --- | --- | --- | --- | --- | --- | --- | --- | --- | --- | --- | --- | --- | --- | --- | --- | --- | --- | --- | --- | --- | --- | --- | --- | --- | --- | --- | --- | --- | --- | --- | --- | --- | --- | --- | --- | --- | --- | --- | --- | --- | --- | --- | --- | --- | --- | --- | --- | --- | --- | --- | --- | --- | --- | --- | --- | --- | --- | --- | --- | --- | --- | --- | --- | --- | --- | --- | --- | --- | --- | --- | --- | --- | --- | --- | --- | --- | --- | --- | --- | --- | --- | --- | --- | --- | --- | --- | --- | --- | --- | --- | --- | --- | --- | --- | --- | --- | --- | --- | --- | --- | --- | --- | --- | --- | --- | --- | --- | --- | --- | --- | --- | --- | --- | --- | --- | --- | --- | --- | --- | --- | --- | --- | --- | --- | --- | --- | --- | --- | --- | --- | --- | --- | --- | --- | --- | --- | --- | --- | --- | --- | --- | --- | --- | --- | --- | --- | --- | --- | --- | --- | --- | --- | --- | --- | --- | --- | --- | --- | --- | --- | --- | --- | --- | --- | --- | --- | --- | --- | --- | --- | --- | --- | --- | --- | --- | --- | --- | --- | --- | --- | --- | --- | --- | --- | --- | --- | --- | --- | --- | --- | --- | --- | --- | --- | --- | --- | --- | --- | --- | --- | --- | --- | --- | --- | --- | --- | --- | --- | --- | --- | --- | --- | --- | --- | --- | --- | --- | --- | --- | --- | --- | --- | --- | --- | --- | --- | --- | --- | --- | --- | --- | --- | --- | --- | --- | --- | --- | --- | --- | --- | --- | --- | --- | --- | --- | --- | --- | --- | --- | --- | --- | --- | --- | --- | --- | --- | --- | --- | --- | --- | --- | --- | --- | --- | --- | --- | --- | --- | --- | --- | --- | --- | --- | --- | --- | --- | --- | --- | --- | --- | --- | --- | --- | --- | --- | --- | --- | --- | --- | --- | --- | --- | --- | --- | --- | --- | --- | --- | --- | --- | --- | --- | --- | --- | --- | --- | --- | --- | --- | --- | --- | --- | --- | --- | --- | --- | --- | --- | --- | --- | --- | --- | --- | --- | --- | --- | --- | --- | --- | --- | --- | --- | --- | --- | --- | --- | --- | --- | --- | --- | --- | --- | --- | --- | --- | --- | --- | --- | --- | --- | --- | --- | --- | --- | --- | --- | --- | --- | --- | --- | --- | --- | --- | --- | --- | --- | --- | --- | --- | --- | --- | --- |
|  |

25

| | **Your Input:** **PF3D7_0925400** | | |  |  |  |  |  |  |  |  |  | | --- | --- | --- | --- | --- | --- | --- | --- | --- | --- | --- | --- | |  | [PFI1245c](http://string-db.org/newstring_cgi/display_single_node.pl?taskId=HGXsOKg0usue&node=401862&targetmode=proteins) | Protein phosphatase-beta (466 aa) | |  |  | *(Plasmodium falciparum)* | | **Predicted Functional Partners:** | | | |  | [PF14_0064](http://string-db.org/newstring_cgi/display_single_node.pl?taskId=HGXsOKg0usue&node=399333&targetmode=proteins) | vacuolar protein sorting 29, putative (194 aa) |  |  |  | [**•**](http://string-db.org/newstring_cgi/show_coexpression_evidence.pl?taskId=HGXsOKg0usue&node2=399333) |  |  | [**•**](http://string-db.org/newstring_cgi/show_textmining_evidence.pl?taskId=HGXsOKg0usue&node2=399333) |  | 0.658 | |  | [PF14_0029](http://string-db.org/newstring_cgi/display_single_node.pl?taskId=HGXsOKg0usue&node=399296&targetmode=proteins) | hypothetical protein (2456 aa) |  |  |  |  |  |  | [**•**](http://string-db.org/newstring_cgi/show_textmining_evidence.pl?taskId=HGXsOKg0usue&node2=399296) |  | 0.651 | |  | [PF07_0110](http://string-db.org/newstring_cgi/display_single_node.pl?taskId=HGXsOKg0usue&node=397814&targetmode=proteins) | hypothetical protein, conserved (519 aa) |  |  |  |  |  |  | [**•**](http://string-db.org/newstring_cgi/show_textmining_evidence.pl?taskId=HGXsOKg0usue&node2=397814) |  | 0.651 | |  | [PFE0795c](http://string-db.org/newstring_cgi/display_single_node.pl?taskId=HGXsOKg0usue&node=401117&targetmode=proteins) | nif-like protein, putative (328 aa) |  |  |  |  |  |  | [**•**](http://string-db.org/newstring_cgi/show_textmining_evidence.pl?taskId=HGXsOKg0usue&node2=401117) |  | 0.649 | |  | [PF14_0614](http://string-db.org/newstring_cgi/display_single_node.pl?taskId=HGXsOKg0usue&node=399896&targetmode=proteins) | hypothetical protein (1502 aa) |  |  |  |  |  |  | [**•**](http://string-db.org/newstring_cgi/show_textmining_evidence.pl?taskId=HGXsOKg0usue&node2=399896) |  | 0.649 | |  | [PF13_0222](http://string-db.org/newstring_cgi/display_single_node.pl?taskId=HGXsOKg0usue&node=399135&targetmode=proteins) | RNA lariat debranching enzyme, putative (575 aa) |  |  |  |  |  |  | [**•**](http://string-db.org/newstring_cgi/show_textmining_evidence.pl?taskId=HGXsOKg0usue&node2=399135) |  | 0.649 | |  | [PF11_0362](http://string-db.org/newstring_cgi/display_single_node.pl?taskId=HGXsOKg0usue&node=398753&targetmode=proteins) | protein phosphatase, putative (689 aa) |  |  |  |  |  |  | [**•**](http://string-db.org/newstring_cgi/show_textmining_evidence.pl?taskId=HGXsOKg0usue&node2=398753) |  | 0.649 | |  | [PF10_0124](http://string-db.org/newstring_cgi/display_single_node.pl?taskId=HGXsOKg0usue&node=398108&targetmode=proteins) | hypothetical protein (1438 aa) |  |  |  |  |  |  | [**•**](http://string-db.org/newstring_cgi/show_textmining_evidence.pl?taskId=HGXsOKg0usue&node2=398108) |  | 0.649 | |  | [PF10_0093](http://string-db.org/newstring_cgi/display_single_node.pl?taskId=HGXsOKg0usue&node=398077&targetmode=proteins) | hypothetical protein (345 aa) |  |  |  |  |  |  | [**•**](http://string-db.org/newstring_cgi/show_textmining_evidence.pl?taskId=HGXsOKg0usue&node2=398077) |  | 0.649 | |  | [MAL8P1.109](http://string-db.org/newstring_cgi/display_single_node.pl?taskId=HGXsOKg0usue&node=397534&targetmode=proteins) | Protein phosphatase 2C, putative (545 aa) |  |  |  |  |  |  | [**•**](http://string-db.org/newstring_cgi/show_textmining_evidence.pl?taskId=HGXsOKg0usue&node2=397534) |  | 0.649 | |
| --- | --- | --- | --- | --- | --- | --- | --- | --- | --- | --- | --- | --- | --- | --- | --- | --- | --- | --- | --- | --- | --- | --- | --- | --- | --- | --- | --- | --- | --- | --- | --- | --- | --- | --- | --- | --- | --- | --- | --- | --- | --- | --- | --- | --- | --- | --- | --- | --- | --- | --- | --- | --- | --- | --- | --- | --- | --- | --- | --- | --- | --- | --- | --- | --- | --- | --- | --- | --- | --- | --- | --- | --- | --- | --- | --- | --- | --- | --- | --- | --- | --- | --- | --- | --- | --- | --- | --- | --- | --- | --- | --- | --- | --- | --- | --- | --- | --- | --- | --- | --- | --- | --- | --- | --- | --- | --- | --- | --- | --- | --- | --- | --- | --- | --- | --- | --- | --- | --- | --- | --- | --- | --- | --- | --- | --- | --- | --- | --- | --- | --- | --- | --- | --- | --- | --- | --- | --- | --- | --- | --- | --- |

26.

| | **Your Input:** **PF3D7_0927700** | | |  |  |  |  |  |  |  |  |  | | --- | --- | --- | --- | --- | --- | --- | --- | --- | --- | --- | --- | |  | [PFI1360c](http://string-db.org/newstring_cgi/display_single_node.pl?taskId=KRvlzBf4_RuU&node=401886&targetmode=proteins) | serine%2Fthreonine protein phosphatase, putative (312 aa) | |  |  | *(Plasmodium falciparum)* | | **Predicted Functional Partners:** | | | |  | [PF14_0064](http://string-db.org/newstring_cgi/display_single_node.pl?taskId=KRvlzBf4_RuU&node=399333&targetmode=proteins) | vacuolar protein sorting 29, putative (194 aa) |  |  |  | [**•**](http://string-db.org/newstring_cgi/show_coexpression_evidence.pl?taskId=KRvlzBf4_RuU&node2=399333) |  |  | [**•**](http://string-db.org/newstring_cgi/show_textmining_evidence.pl?taskId=KRvlzBf4_RuU&node2=399333) |  | 0.739 | |  | [PFL0300c](http://string-db.org/newstring_cgi/display_single_node.pl?taskId=KRvlzBf4_RuU&node=402045&targetmode=proteins) | phosphoesterase, putative (304 aa) |  |  |  | [**•**](http://string-db.org/newstring_cgi/show_coexpression_evidence.pl?taskId=KRvlzBf4_RuU&node2=402045) |  |  | [**•**](http://string-db.org/newstring_cgi/show_textmining_evidence.pl?taskId=KRvlzBf4_RuU&node2=402045) |  | 0.673 | |  | [PFA_0285c](http://string-db.org/newstring_cgi/display_single_node.pl?taskId=KRvlzBf4_RuU&node=400130&targetmode=proteins) | hypothetical protein, conserved (832 aa) |  |  |  | [**•**](http://string-db.org/newstring_cgi/show_coexpression_evidence.pl?taskId=KRvlzBf4_RuU&node2=400130) |  |  |  |  | 0.671 | |  | [PF14_0614](http://string-db.org/newstring_cgi/display_single_node.pl?taskId=KRvlzBf4_RuU&node=399896&targetmode=proteins) | hypothetical protein (1502 aa) |  |  |  |  |  |  | [**•**](http://string-db.org/newstring_cgi/show_textmining_evidence.pl?taskId=KRvlzBf4_RuU&node2=399896) |  | 0.651 | |  | [PF13_0222](http://string-db.org/newstring_cgi/display_single_node.pl?taskId=KRvlzBf4_RuU&node=399135&targetmode=proteins) | RNA lariat debranching enzyme, putative (575 aa) |  |  |  |  |  |  | [**•**](http://string-db.org/newstring_cgi/show_textmining_evidence.pl?taskId=KRvlzBf4_RuU&node2=399135) |  | 0.651 | |  | [PF14_0660](http://string-db.org/newstring_cgi/display_single_node.pl?taskId=KRvlzBf4_RuU&node=399944&targetmode=proteins) | hypothetical protein (358 aa) |  |  |  |  |  |  | [**•**](http://string-db.org/newstring_cgi/show_textmining_evidence.pl?taskId=KRvlzBf4_RuU&node2=399944) |  | 0.651 | |  | [PfEF-1beta](http://string-db.org/newstring_cgi/display_single_node.pl?taskId=KRvlzBf4_RuU&node=401740&targetmode=proteins) | EF-1B (276 aa) |  |  |  |  |  |  | [**•**](http://string-db.org/newstring_cgi/show_textmining_evidence.pl?taskId=KRvlzBf4_RuU&node2=401740) |  | 0.649 | |  | [PF14_0463](http://string-db.org/newstring_cgi/display_single_node.pl?taskId=KRvlzBf4_RuU&node=399739&targetmode=proteins) | chloroquine resistance marker protein (3704 aa) |  |  |  | [**•**](http://string-db.org/newstring_cgi/show_coexpression_evidence.pl?taskId=KRvlzBf4_RuU&node2=399739) |  |  |  |  | 0.647 | |  | [PFD0770c](http://string-db.org/newstring_cgi/display_single_node.pl?taskId=KRvlzBf4_RuU&node=400856&targetmode=proteins) | ribosomal protein l15, putative (220 aa) |  |  |  |  |  |  | [**•**](http://string-db.org/newstring_cgi/show_textmining_evidence.pl?taskId=KRvlzBf4_RuU&node2=400856) |  | 0.645 | |  | [PF14_0036](http://string-db.org/newstring_cgi/display_single_node.pl?taskId=KRvlzBf4_RuU&node=399305&targetmode=proteins) | acid phosphatase, putative (302 aa) |  |  |  |  |  |  | [**•**](http://string-db.org/newstring_cgi/show_textmining_evidence.pl?taskId=KRvlzBf4_RuU&node2=399305) |  | 0.641 | |
| --- | --- | --- | --- | --- | --- | --- | --- | --- | --- | --- | --- | --- | --- | --- | --- | --- | --- | --- | --- | --- | --- | --- | --- | --- | --- | --- | --- | --- | --- | --- | --- | --- | --- | --- | --- | --- | --- | --- | --- | --- | --- | --- | --- | --- | --- | --- | --- | --- | --- | --- | --- | --- | --- | --- | --- | --- | --- | --- | --- | --- | --- | --- | --- | --- | --- | --- | --- | --- | --- | --- | --- | --- | --- | --- | --- | --- | --- | --- | --- | --- | --- | --- | --- | --- | --- | --- | --- | --- | --- | --- | --- | --- | --- | --- | --- | --- | --- | --- | --- | --- | --- | --- | --- | --- | --- | --- | --- | --- | --- | --- | --- | --- | --- | --- | --- | --- | --- | --- | --- | --- | --- | --- | --- | --- | --- | --- | --- | --- | --- | --- | --- | --- | --- | --- | --- | --- | --- | --- | --- | --- | --- |

27.

| | | | **Your Input:** **PF3D7_1009600** | | |  |  |  |  |  |  |  |  |  | | --- | --- | --- | --- | --- | --- | --- | --- | --- | --- | --- | --- | |  | [PF10_0093](http://string-db.org/newstring_cgi/display_single_node.pl?taskId=v2bPlLWI8yv2&node=398077&targetmode=proteins) | hypothetical protein (345 aa) | |  |  | *(Plasmodium falciparum)* | | **Predicted Functional Partners:** | | | |  | [PFL2365w](http://string-db.org/newstring_cgi/display_single_node.pl?taskId=v2bPlLWI8yv2&node=402468&targetmode=proteins) | hypothetical protein, conserved (1027 aa) |  |  |  |  |  |  | [**•**](http://string-db.org/newstring_cgi/show_textmining_evidence.pl?taskId=v2bPlLWI8yv2&node2=402468) |  | 0.688 | |  | [PFE1010w](http://string-db.org/newstring_cgi/display_single_node.pl?taskId=v2bPlLWI8yv2&node=401160&targetmode=proteins) | protein phosphatase 2c, putative (697 aa) |  |  |  |  |  |  | [**•**](http://string-db.org/newstring_cgi/show_textmining_evidence.pl?taskId=v2bPlLWI8yv2&node2=401160) |  | 0.688 | |  | [PFE0795c](http://string-db.org/newstring_cgi/display_single_node.pl?taskId=v2bPlLWI8yv2&node=401117&targetmode=proteins) | nif-like protein, putative (328 aa) |  |  |  |  |  |  | [**•**](http://string-db.org/newstring_cgi/show_textmining_evidence.pl?taskId=v2bPlLWI8yv2&node2=401117) |  | 0.688 | |  | [PF14_0523](http://string-db.org/newstring_cgi/display_single_node.pl?taskId=v2bPlLWI8yv2&node=399802&targetmode=proteins) | protein phosphatase 2C, putative (289 aa) |  |  |  |  |  |  | [**•**](http://string-db.org/newstring_cgi/show_textmining_evidence.pl?taskId=v2bPlLWI8yv2&node2=399802) |  | 0.688 | |  | [PF11_0362](http://string-db.org/newstring_cgi/display_single_node.pl?taskId=v2bPlLWI8yv2&node=398753&targetmode=proteins) | protein phosphatase, putative (689 aa) |  |  |  |  |  |  | [**•**](http://string-db.org/newstring_cgi/show_textmining_evidence.pl?taskId=v2bPlLWI8yv2&node2=398753) |  | 0.688 | |  | [PF10_0124](http://string-db.org/newstring_cgi/display_single_node.pl?taskId=v2bPlLWI8yv2&node=398108&targetmode=proteins) | hypothetical protein (1438 aa) |  |  |  |  |  |  | [**•**](http://string-db.org/newstring_cgi/show_textmining_evidence.pl?taskId=v2bPlLWI8yv2&node2=398108) |  | 0.688 | |  | [PF07_0110](http://string-db.org/newstring_cgi/display_single_node.pl?taskId=v2bPlLWI8yv2&node=397814&targetmode=proteins) | hypothetical protein, conserved (519 aa) |  |  |  |  |  |  | [**•**](http://string-db.org/newstring_cgi/show_textmining_evidence.pl?taskId=v2bPlLWI8yv2&node2=397814) |  | 0.688 | |  | [MAL8P1.109](http://string-db.org/newstring_cgi/display_single_node.pl?taskId=v2bPlLWI8yv2&node=397534&targetmode=proteins) | Protein phosphatase 2C, putative (545 aa) |  |  |  |  |  |  | [**•**](http://string-db.org/newstring_cgi/show_textmining_evidence.pl?taskId=v2bPlLWI8yv2&node2=397534) |  | 0.688 | |  | [MAL8P1.108](http://string-db.org/newstring_cgi/display_single_node.pl?taskId=v2bPlLWI8yv2&node=397533&targetmode=proteins) | protein phosphatase, putative (303 aa) |  |  |  |  |  |  | [**•**](http://string-db.org/newstring_cgi/show_textmining_evidence.pl?taskId=v2bPlLWI8yv2&node2=397533) |  | 0.688 | |  | [MAL13P1.44](http://string-db.org/newstring_cgi/display_single_node.pl?taskId=v2bPlLWI8yv2&node=397260&targetmode=proteins) | protein phosphatase 2c-like protein, putative (827 aa) |  |  |  |  |  |  | [**•**](http://string-db.org/newstring_cgi/show_textmining_evidence.pl?taskId=v2bPlLWI8yv2&node2=397260) |  | 0.688 | |  | [MAL13P1.174-1](http://string-db.org/newstring_cgi/display_single_node.pl?taskId=v2bPlLWI8yv2&node=397043&targetmode=proteins) | MSP7-like protein (281 aa) |  |  |  |  |  |  | [**•**](http://string-db.org/newstring_cgi/show_textmining_evidence.pl?taskId=v2bPlLWI8yv2&node2=397043) |  | 0.687 | |  | [PFI1245c](http://string-db.org/newstring_cgi/display_single_node.pl?taskId=v2bPlLWI8yv2&node=401862&targetmode=proteins) | Protein phosphatase-beta (466 aa) |  |  |  |  |  |  | [**•**](http://string-db.org/newstring_cgi/show_textmining_evidence.pl?taskId=v2bPlLWI8yv2&node2=401862) |  | 0.649 | |  | [PfPP5](http://string-db.org/newstring_cgi/display_single_node.pl?taskId=v2bPlLWI8yv2&node=397148&targetmode=proteins) | serine%2Fthreonine protein phosphatase pfPp5 (658 aa) |  |  |  |  |  |  | [**•**](http://string-db.org/newstring_cgi/show_textmining_evidence.pl?taskId=v2bPlLWI8yv2&node2=397148) |  | 0.587 | |  | [PF11_0396](http://string-db.org/newstring_cgi/display_single_node.pl?taskId=v2bPlLWI8yv2&node=398787&targetmode=proteins) | Protein phosphatase 2C (938 aa) |  |  |  |  |  |  | [**•**](http://string-db.org/newstring_cgi/show_textmining_evidence.pl?taskId=v2bPlLWI8yv2&node2=398787) |  | 0.587 | |  | [PFL0320w](http://string-db.org/newstring_cgi/display_single_node.pl?taskId=v2bPlLWI8yv2&node=402049&targetmode=proteins) | hypothetical protein, conserved (346 aa) |  |  |  |  |  |  | [**•**](http://string-db.org/newstring_cgi/show_textmining_evidence.pl?taskId=v2bPlLWI8yv2&node2=402049) |  | 0.480 | |  | [PF13_0027](http://string-db.org/newstring_cgi/display_single_node.pl?taskId=v2bPlLWI8yv2&node=398952&targetmode=proteins) | hypothetical protein, conserved (771 aa) |  |  |  |  |  |  | [**•**](http://string-db.org/newstring_cgi/show_textmining_evidence.pl?taskId=v2bPlLWI8yv2&node2=398952) |  | 0.480 | |  | [MAL13P1.168-1](http://string-db.org/newstring_cgi/display_single_node.pl?taskId=v2bPlLWI8yv2&node=397036&targetmode=proteins) | hypothetical protein, conserved (267 aa) |  |  |  |  |  |  | [**•**](http://string-db.org/newstring_cgi/show_textmining_evidence.pl?taskId=v2bPlLWI8yv2&node2=397036) |  | 0.480 | |  | [PF14_0525](http://string-db.org/newstring_cgi/display_single_node.pl?taskId=v2bPlLWI8yv2&node=399804&targetmode=proteins) | hypothetical protein (89 aa) |  |  |  |  |  |  | [**•**](http://string-db.org/newstring_cgi/show_textmining_evidence.pl?taskId=v2bPlLWI8yv2&node2=399804) |  | 0.480 | |  | [PF11_0281](http://string-db.org/newstring_cgi/display_single_node.pl?taskId=v2bPlLWI8yv2&node=398671&targetmode=proteins) | hypothetical protein (247 aa) |  |  |  |  |  |  | [**•**](http://string-db.org/newstring_cgi/show_textmining_evidence.pl?taskId=v2bPlLWI8yv2&node2=398671) |  | 0.480 | |  | [MAL13P1.275-1](http://string-db.org/newstring_cgi/display_single_node.pl?taskId=v2bPlLWI8yv2&node=397149&targetmode=proteins) | NLI interacting factor-like phosphatase, putative (1288 aa) |  |  |  |  |  |  | [**•**](http://string-db.org/newstring_cgi/show_textmining_evidence.pl?taskId=v2bPlLWI8yv2&node2=397149) |  | 0.480 | | | --- | --- | --- | --- | --- | --- | --- | --- | --- | --- | --- | --- | --- | --- | --- | --- | --- | --- | --- | --- | --- | --- | --- | --- | --- | --- | --- | --- | --- | --- | --- | --- | --- | --- | --- | --- | --- | --- | --- | --- | --- | --- | --- | --- | --- | --- | --- | --- | --- | --- | --- | --- | --- | --- | --- | --- | --- | --- | --- | --- | --- | --- | --- | --- | --- | --- | --- | --- | --- | --- | --- | --- | --- | --- | --- | --- | --- | --- | --- | --- | --- | --- | --- | --- | --- | --- | --- | --- | --- | --- | --- | --- | --- | --- | --- | --- | --- | --- | --- | --- | --- | --- | --- | --- | --- | --- | --- | --- | --- | --- | --- | --- | --- | --- | --- | --- | --- | --- | --- | --- | --- | --- | --- | --- | --- | --- | --- | --- | --- | --- | --- | --- | --- | --- | --- | --- | --- | --- | --- | --- | --- | --- | --- | --- | --- | --- | --- | --- | --- | --- | --- | --- | --- | --- | --- | --- | --- | --- | --- | --- | --- | --- | --- | --- | --- | --- | --- | --- | --- | --- | --- | --- | --- | --- | --- | --- | --- | --- | --- | --- | --- | --- | --- | --- | --- | --- | --- | --- | --- | --- | --- | --- | --- | --- | --- | --- | --- | --- | --- | --- | --- | --- | --- | --- | --- | --- | --- | --- | --- | --- | --- | --- | --- | --- | --- | --- | --- | --- | --- | --- | --- | --- | --- | --- | --- | --- | --- | --- | --- | --- | --- | --- | --- | --- | --- | --- | --- | --- | --- | --- | --- | --- | --- | --- | --- | --- | --- | --- | --- | --- | --- | --- | --- | --- | --- | --- | --- | --- | --- | --- | --- | --- | | | --- | --- | --- | --- | --- | --- | --- | --- | --- | --- | --- | --- | --- | --- | --- | --- | --- | --- | --- | --- | --- | --- | --- | --- | --- | --- | --- | --- | --- | --- | --- | --- | --- | --- | --- | --- | --- | --- | --- | --- | --- | --- | --- | --- | --- | --- | --- | --- | --- | --- | --- | --- | --- | --- | --- | --- | --- | --- | --- | --- | --- | --- | --- | --- | --- | --- | --- | --- | --- | --- | --- | --- | --- | --- | --- | --- | --- | --- | --- | --- | --- | --- | --- | --- | --- | --- | --- | --- | --- | --- | --- | --- | --- | --- | --- | --- | --- | --- | --- | --- | --- | --- | --- | --- | --- | --- | --- | --- | --- | --- | --- | --- | --- | --- | --- | --- | --- | --- | --- | --- | --- | --- | --- | --- | --- | --- | --- | --- | --- | --- | --- | --- | --- | --- | --- | --- | --- | --- | --- | --- | --- | --- | --- | --- | --- | --- | --- | --- | --- | --- | --- | --- | --- | --- | --- | --- | --- | --- | --- | --- | --- | --- | --- | --- | --- | --- | --- | --- | --- | --- | --- | --- | --- | --- | --- | --- | --- | --- | --- | --- | --- | --- | --- | --- | --- | --- | --- | --- | --- | --- | --- | --- | --- | --- | --- | --- | --- | --- | --- | --- | --- | --- | --- | --- | --- | --- | --- | --- | --- | --- | --- | --- | --- | --- | --- | --- | --- | --- | --- | --- | --- | --- | --- | --- | --- | --- | --- | --- | --- | --- | --- | --- | --- | --- | --- | --- | --- | --- | --- | --- | --- | --- | --- | --- | --- | --- | --- | --- | --- | --- | --- | --- | --- | --- | --- | --- | --- | --- | --- | --- | --- | --- | --- | |
| --- | --- | --- | --- | --- | --- | --- | --- | --- | --- | --- | --- | --- | --- | --- | --- | --- | --- | --- | --- | --- | --- | --- | --- | --- | --- | --- | --- | --- | --- | --- | --- | --- | --- | --- | --- | --- | --- | --- | --- | --- | --- | --- | --- | --- | --- | --- | --- | --- | --- | --- | --- | --- | --- | --- | --- | --- | --- | --- | --- | --- | --- | --- | --- | --- | --- | --- | --- | --- | --- | --- | --- | --- | --- | --- | --- | --- | --- | --- | --- | --- | --- | --- | --- | --- | --- | --- | --- | --- | --- | --- | --- | --- | --- | --- | --- | --- | --- | --- | --- | --- | --- | --- | --- | --- | --- | --- | --- | --- | --- | --- | --- | --- | --- | --- | --- | --- | --- | --- | --- | --- | --- | --- | --- | --- | --- | --- | --- | --- | --- | --- | --- | --- | --- | --- | --- | --- | --- | --- | --- | --- | --- | --- | --- | --- | --- | --- | --- | --- | --- | --- | --- | --- | --- | --- | --- | --- | --- | --- | --- | --- | --- | --- | --- | --- | --- | --- | --- | --- | --- | --- | --- | --- | --- | --- | --- | --- | --- | --- | --- | --- | --- | --- | --- | --- | --- | --- | --- | --- | --- | --- | --- | --- | --- | --- | --- | --- | --- | --- | --- | --- | --- | --- | --- | --- | --- | --- | --- | --- | --- | --- | --- | --- | --- | --- | --- | --- | --- | --- | --- | --- | --- | --- | --- | --- | --- | --- | --- | --- | --- | --- | --- | --- | --- | --- | --- | --- | --- | --- | --- | --- | --- | --- | --- | --- | --- | --- | --- | --- | --- | --- | --- | --- | --- | --- | --- | --- | --- | --- | --- | --- | --- | --- | --- |
| |  | | --- | |

28.

| | **Your Input:** **PF3D7_1012700** | | |  |  |  |  |  |  |  |  |  | | --- | --- | --- | --- | --- | --- | --- | --- | --- | --- | --- | --- | |  | [PF10_0124](http://string-db.org/newstring_cgi/display_single_node.pl?taskId=D4o4rP7qviIK&node=398108&targetmode=proteins) | hypothetical protein (1438 aa) | |  |  | *(Plasmodium falciparum)* | | **Predicted Functional Partners:** | | | |  | [PFC0805w](http://string-db.org/newstring_cgi/display_single_node.pl?taskId=D4o4rP7qviIK&node=400633&targetmode=proteins) | DNA-directed RNA polymerase II, putative; DNA-dependent RNA polymerase catalyzes the transcript [...] (2457 aa) |  |  |  |  |  | [**•**](http://string-db.org/newstring_cgi/show_set_evidence.pl?data_channel=database&taskId=D4o4rP7qviIK&node2=400633) | [**•**](http://string-db.org/newstring_cgi/show_textmining_evidence.pl?taskId=D4o4rP7qviIK&node2=400633) |  | 0.906 | |  | [PFL0665c](http://string-db.org/newstring_cgi/display_single_node.pl?taskId=D4o4rP7qviIK&node=402120&targetmode=proteins) | RNA polymerase subunit 8c, putative (145 aa) |  |  |  |  |  | [**•**](http://string-db.org/newstring_cgi/show_set_evidence.pl?data_channel=database&taskId=D4o4rP7qviIK&node2=402120) |  |  | 0.899 | |  | [PFI1130c](http://string-db.org/newstring_cgi/display_single_node.pl?taskId=D4o4rP7qviIK&node=401838&targetmode=proteins) | DNA-directed RNA polymerase II, putative (335 aa) |  |  |  |  |  | [**•**](http://string-db.org/newstring_cgi/show_set_evidence.pl?data_channel=database&taskId=D4o4rP7qviIK&node2=401838) |  |  | 0.899 | |  | [PFF0535c](http://string-db.org/newstring_cgi/display_single_node.pl?taskId=D4o4rP7qviIK&node=401390&targetmode=proteins) | trancription factor, putative (1280 aa) |  |  |  |  |  | [**•**](http://string-db.org/newstring_cgi/show_set_evidence.pl?data_channel=database&taskId=D4o4rP7qviIK&node2=401390) |  |  | 0.899 | |  | [PFD0750w](http://string-db.org/newstring_cgi/display_single_node.pl?taskId=D4o4rP7qviIK&node=400852&targetmode=proteins) | nuclear cap-binding protein, putative (234 aa) |  |  |  |  |  | [**•**](http://string-db.org/newstring_cgi/show_set_evidence.pl?data_channel=database&taskId=D4o4rP7qviIK&node2=400852) |  |  | 0.899 | |  | [PFC0155c](http://string-db.org/newstring_cgi/display_single_node.pl?taskId=D4o4rP7qviIK&node=400488&targetmode=proteins) | DNA-directed RNA polymerase subunit I, putative (163 aa) |  |  |  |  |  | [**•**](http://string-db.org/newstring_cgi/show_set_evidence.pl?data_channel=database&taskId=D4o4rP7qviIK&node2=400488) |  |  | 0.899 | |  | [PFB0715w](http://string-db.org/newstring_cgi/display_single_node.pl?taskId=D4o4rP7qviIK&node=400375&targetmode=proteins) | DNA-directed RNA polymerase II second largest subunit, putative (1364 aa) |  |  |  |  |  | [**•**](http://string-db.org/newstring_cgi/show_set_evidence.pl?data_channel=database&taskId=D4o4rP7qviIK&node2=400375) |  | **•** | 0.899 | |  | [PFB0245c](http://string-db.org/newstring_cgi/display_single_node.pl?taskId=D4o4rP7qviIK&node=400276&targetmode=proteins) | DNA-directed RNA polymerase II 16 kDa subunit, putative (132 aa) |  |  |  |  |  | [**•**](http://string-db.org/newstring_cgi/show_set_evidence.pl?data_channel=database&taskId=D4o4rP7qviIK&node2=400276) |  |  | 0.899 | |  | [PFA_0505c](http://string-db.org/newstring_cgi/display_single_node.pl?taskId=D4o4rP7qviIK&node=400175&targetmode=proteins) | DNA-directed RNA polymerase 2 subunit, putative (249 aa) |  |  |  |  |  | [**•**](http://string-db.org/newstring_cgi/show_set_evidence.pl?data_channel=database&taskId=D4o4rP7qviIK&node2=400175) |  |  | 0.899 | |  | [PF13_0341](http://string-db.org/newstring_cgi/display_single_node.pl?taskId=D4o4rP7qviIK&node=399248&targetmode=proteins) | DNA-directed RNA polymerase 2, putative (205 aa) |  |  |  |  |  | [**•**](http://string-db.org/newstring_cgi/show_set_evidence.pl?data_channel=database&taskId=D4o4rP7qviIK&node2=399248) |  |  | 0.899 | |  | [PF13_0023](http://string-db.org/newstring_cgi/display_single_node.pl?taskId=D4o4rP7qviIK&node=398948&targetmode=proteins) | DNA-directed RNA polymerase 2, putative; DNA-dependent RNA polymerase catalyzes the transcripti [...] (126 aa) |  |  |  |  |  | [**•**](http://string-db.org/newstring_cgi/show_set_evidence.pl?data_channel=database&taskId=D4o4rP7qviIK&node2=398948) |  |  | 0.899 | |  | [PF10_0293](http://string-db.org/newstring_cgi/display_single_node.pl?taskId=D4o4rP7qviIK&node=398285&targetmode=proteins) | transcription factor, putative (133 aa) |  |  |  |  |  | [**•**](http://string-db.org/newstring_cgi/show_set_evidence.pl?data_channel=database&taskId=D4o4rP7qviIK&node2=398285) |  |  | 0.899 | |  | [PF10_0269](http://string-db.org/newstring_cgi/display_single_node.pl?taskId=D4o4rP7qviIK&node=398261&targetmode=proteins) | DNA-directed RNA polymerase II, putative (177 aa) |  |  |  |  |  | [**•**](http://string-db.org/newstring_cgi/show_set_evidence.pl?data_channel=database&taskId=D4o4rP7qviIK&node2=398261) |  |  | 0.899 | |  | [PF07_0027](http://string-db.org/newstring_cgi/display_single_node.pl?taskId=D4o4rP7qviIK&node=397729&targetmode=proteins) | DNA-directed RNA polymerase 2 8.2 kDa polypeptide, putative (69 aa) |  |  |  |  |  | [**•**](http://string-db.org/newstring_cgi/show_set_evidence.pl?data_channel=database&taskId=D4o4rP7qviIK&node2=397729) |  |  | 0.899 | |  | [MAL13P1.174-1](http://string-db.org/newstring_cgi/display_single_node.pl?taskId=D4o4rP7qviIK&node=397043&targetmode=proteins) | MSP7-like protein (281 aa) |  |  |  | [**•**](http://string-db.org/newstring_cgi/show_coexpression_evidence.pl?taskId=D4o4rP7qviIK&node2=397043) |  |  | [**•**](http://string-db.org/newstring_cgi/show_textmining_evidence.pl?taskId=D4o4rP7qviIK&node2=397043) |  | 0.709 | |  | [PF07_0110](http://string-db.org/newstring_cgi/display_single_node.pl?taskId=D4o4rP7qviIK&node=397814&targetmode=proteins) | hypothetical protein, conserved (519 aa) |  |  |  |  |  |  | [**•**](http://string-db.org/newstring_cgi/show_textmining_evidence.pl?taskId=D4o4rP7qviIK&node2=397814) |  | 0.690 | |  | [PFE0795c](http://string-db.org/newstring_cgi/display_single_node.pl?taskId=D4o4rP7qviIK&node=401117&targetmode=proteins) | nif-like protein, putative (328 aa) |  |  |  |  |  |  | [**•**](http://string-db.org/newstring_cgi/show_textmining_evidence.pl?taskId=D4o4rP7qviIK&node2=401117) |  | 0.688 | |  | [PF10_0093](http://string-db.org/newstring_cgi/display_single_node.pl?taskId=D4o4rP7qviIK&node=398077&targetmode=proteins) | hypothetical protein (345 aa) |  |  |  |  |  |  | [**•**](http://string-db.org/newstring_cgi/show_textmining_evidence.pl?taskId=D4o4rP7qviIK&node2=398077) |  | 0.688 | |  | [MAL8P1.109](http://string-db.org/newstring_cgi/display_single_node.pl?taskId=D4o4rP7qviIK&node=397534&targetmode=proteins) | Protein phosphatase 2C, putative (545 aa) |  |  |  |  |  |  | [**•**](http://string-db.org/newstring_cgi/show_textmining_evidence.pl?taskId=D4o4rP7qviIK&node2=397534) |  | 0.688 | |  | [MAL13P1.44](http://string-db.org/newstring_cgi/display_single_node.pl?taskId=D4o4rP7qviIK&node=397260&targetmode=proteins) | protein phosphatase 2c-like protein, putative (827 aa) |  |  |  |  |  |  | [**•**](http://string-db.org/newstring_cgi/show_textmining_evidence.pl?taskId=D4o4rP7qviIK&node2=397260) |  | 0.688 | |
| --- | --- | --- | --- | --- | --- | --- | --- | --- | --- | --- | --- | --- | --- | --- | --- | --- | --- | --- | --- | --- | --- | --- | --- | --- | --- | --- | --- | --- | --- | --- | --- | --- | --- | --- | --- | --- | --- | --- | --- | --- | --- | --- | --- | --- | --- | --- | --- | --- | --- | --- | --- | --- | --- | --- | --- | --- | --- | --- | --- | --- | --- | --- | --- | --- | --- | --- | --- | --- | --- | --- | --- | --- | --- | --- | --- | --- | --- | --- | --- | --- | --- | --- | --- | --- | --- | --- | --- | --- | --- | --- | --- | --- | --- | --- | --- | --- | --- | --- | --- | --- | --- | --- | --- | --- | --- | --- | --- | --- | --- | --- | --- | --- | --- | --- | --- | --- | --- | --- | --- | --- | --- | --- | --- | --- | --- | --- | --- | --- | --- | --- | --- | --- | --- | --- | --- | --- | --- | --- | --- | --- | --- | --- | --- | --- | --- | --- | --- | --- | --- | --- | --- | --- | --- | --- | --- | --- | --- | --- | --- | --- | --- | --- | --- | --- | --- | --- | --- | --- | --- | --- | --- | --- | --- | --- | --- | --- | --- | --- | --- | --- | --- | --- | --- | --- | --- | --- | --- | --- | --- | --- | --- | --- | --- | --- | --- | --- | --- | --- | --- | --- | --- | --- | --- | --- | --- | --- | --- | --- | --- | --- | --- | --- | --- | --- | --- | --- | --- | --- | --- | --- | --- | --- | --- | --- | --- | --- | --- | --- | --- | --- | --- | --- | --- | --- | --- | --- | --- | --- | --- | --- | --- | --- | --- | --- | --- | --- | --- | --- | --- | --- | --- | --- | --- | --- | --- | --- | --- | --- | --- | --- | --- |

29.

| | **Your Input:** **PF3D7_1018200** | | |  |  |  |  |  |  |  |  |  | | --- | --- | --- | --- | --- | --- | --- | --- | --- | --- | --- | --- | |  | [PF10_0177a](http://string-db.org/newstring_cgi/display_single_node.pl?taskId=Gu7m_AjnHrAE&node=398164&targetmode=proteins) | erythrocyte membrane-associated antigen (2166 aa) | |  |  | *(Plasmodium falciparum)* | | **Predicted Functional Partners:** | | | |  | [PFD0770c](http://string-db.org/newstring_cgi/display_single_node.pl?taskId=Gu7m_AjnHrAE&node=400856&targetmode=proteins) | ribosomal protein l15, putative (220 aa) |  |  |  |  |  |  | [**•**](http://string-db.org/newstring_cgi/show_textmining_evidence.pl?taskId=Gu7m_AjnHrAE&node2=400856) |  | 0.653 | |  | [Rab2](http://string-db.org/newstring_cgi/display_single_node.pl?taskId=Gu7m_AjnHrAE&node=402291&targetmode=proteins) | Rab2, GTPase (213 aa) |  |  |  |  |  |  | [**•**](http://string-db.org/newstring_cgi/show_textmining_evidence.pl?taskId=Gu7m_AjnHrAE&node2=402291) |  | 0.651 | |  | [PfEF-1beta](http://string-db.org/newstring_cgi/display_single_node.pl?taskId=Gu7m_AjnHrAE&node=401740&targetmode=proteins) | EF-1B (276 aa) |  |  |  |  |  |  | [**•**](http://string-db.org/newstring_cgi/show_textmining_evidence.pl?taskId=Gu7m_AjnHrAE&node2=401740) |  | 0.643 | |  | [KIN](http://string-db.org/newstring_cgi/display_single_node.pl?taskId=Gu7m_AjnHrAE&node=399795&targetmode=proteins) | serine%2Fthreonine-protein kinase (765 aa) |  |  |  |  |  |  | [**•**](http://string-db.org/newstring_cgi/show_textmining_evidence.pl?taskId=Gu7m_AjnHrAE&node2=399795) |  | 0.583 | |  | [PfPK9](http://string-db.org/newstring_cgi/display_single_node.pl?taskId=Gu7m_AjnHrAE&node=399008&targetmode=proteins) | serine%2Fthreonine protein kinase, putative (367 aa) |  |  |  |  |  |  | [**•**](http://string-db.org/newstring_cgi/show_textmining_evidence.pl?taskId=Gu7m_AjnHrAE&node2=399008) |  | 0.583 | |  | [PF10_0268](http://string-db.org/newstring_cgi/display_single_node.pl?taskId=Gu7m_AjnHrAE&node=398260&targetmode=proteins) | merozoite capping protein 1 (393 aa) |  |  |  |  |  |  | [**•**](http://string-db.org/newstring_cgi/show_textmining_evidence.pl?taskId=Gu7m_AjnHrAE&node2=398260) |  | 0.579 | |  | [PF10_0203](http://string-db.org/newstring_cgi/display_single_node.pl?taskId=Gu7m_AjnHrAE&node=398190&targetmode=proteins) | ADP-ribosylation factor; GTP-binding protein that functions as an allosteric activator of the c [...] (181 aa) |  |  |  |  |  |  | [**•**](http://string-db.org/newstring_cgi/show_textmining_evidence.pl?taskId=Gu7m_AjnHrAE&node2=398190) |  | 0.579 | |  | [PF11_0301](http://string-db.org/newstring_cgi/display_single_node.pl?taskId=Gu7m_AjnHrAE&node=398691&targetmode=proteins) | spermidine synthase (321 aa) |  |  |  |  |  |  | [**•**](http://string-db.org/newstring_cgi/show_textmining_evidence.pl?taskId=Gu7m_AjnHrAE&node2=398691) |  | 0.486 | |  | [PfPK6](http://string-db.org/newstring_cgi/display_single_node.pl?taskId=Gu7m_AjnHrAE&node=397055&targetmode=proteins) | Pf protein kinase 6 (305 aa) |  |  |  |  |  |  | [**•**](http://string-db.org/newstring_cgi/show_textmining_evidence.pl?taskId=Gu7m_AjnHrAE&node2=397055) |  | 0.480 | |
| --- | --- | --- | --- | --- | --- | --- | --- | --- | --- | --- | --- | --- | --- | --- | --- | --- | --- | --- | --- | --- | --- | --- | --- | --- | --- | --- | --- | --- | --- | --- | --- | --- | --- | --- | --- | --- | --- | --- | --- | --- | --- | --- | --- | --- | --- | --- | --- | --- | --- | --- | --- | --- | --- | --- | --- | --- | --- | --- | --- | --- | --- | --- | --- | --- | --- | --- | --- | --- | --- | --- | --- | --- | --- | --- | --- | --- | --- | --- | --- | --- | --- | --- | --- | --- | --- | --- | --- | --- | --- | --- | --- | --- | --- | --- | --- | --- | --- | --- | --- | --- | --- | --- | --- | --- | --- | --- | --- | --- | --- | --- | --- | --- | --- | --- | --- | --- | --- | --- | --- | --- | --- | --- | --- | --- | --- | --- | --- | --- | --- |

30.

| | **Your Input:** **PF3D7_1111600** | | |  |  |  |  |  |  |  |  |  | | --- | --- | --- | --- | --- | --- | --- | --- | --- | --- | --- | --- | |  | [PF11_0122](http://string-db.org/newstring_cgi/display_single_node.pl?taskId=dp8slr93rIff&node=398513&targetmode=proteins) | hypothetical protein (779 aa) | |  |  | *(Plasmodium falciparum)* | | **Predicted Functional Partners:** | | | |  | [PFB0725c](http://string-db.org/newstring_cgi/display_single_node.pl?taskId=dp8slr93rIff&node=400377&targetmode=proteins) | hypothetical protein (108 aa) |  |  |  | [**•**](http://string-db.org/newstring_cgi/show_coexpression_evidence.pl?taskId=dp8slr93rIff&node2=400377) |  |  |  |  | 0.411 | |  | [PF14_0551](http://string-db.org/newstring_cgi/display_single_node.pl?taskId=dp8slr93rIff&node=399830&targetmode=proteins) | hypothetical protein (503 aa) |  |  |  | [**•**](http://string-db.org/newstring_cgi/show_coexpression_evidence.pl?taskId=dp8slr93rIff&node2=399830) |  |  |  |  | 0.405 | |
| --- | --- | --- | --- | --- | --- | --- | --- | --- | --- | --- | --- | --- | --- | --- | --- | --- | --- | --- | --- | --- | --- | --- | --- | --- | --- | --- | --- | --- | --- | --- | --- | --- | --- | --- | --- | --- | --- | --- | --- | --- | --- | --- | --- | --- | --- |

31.

| | **Your Input:** **PF3D7_1113100** | | |  |  |  |  |  |  |  |  |  | | --- | --- | --- | --- | --- | --- | --- | --- | --- | --- | --- | --- | |  | [PRL](http://string-db.org/newstring_cgi/display_single_node.pl?taskId=YKRYooI_NI_4&node=398526&targetmode=proteins) | protein tyrosine phosphatase, putative (218 aa) | |  |  | *(Plasmodium falciparum)* | | **Predicted Functional Partners:** | | | |  | [PF14_0525](http://string-db.org/newstring_cgi/display_single_node.pl?taskId=YKRYooI_NI_4&node=399804&targetmode=proteins) | hypothetical protein (89 aa) |  |  |  |  |  |  | [**•**](http://string-db.org/newstring_cgi/show_textmining_evidence.pl?taskId=YKRYooI_NI_4&node2=399804) |  | 0.780 | |  | [PF11_0281](http://string-db.org/newstring_cgi/display_single_node.pl?taskId=YKRYooI_NI_4&node=398671&targetmode=proteins) | hypothetical protein (247 aa) |  |  |  |  |  |  | [**•**](http://string-db.org/newstring_cgi/show_textmining_evidence.pl?taskId=YKRYooI_NI_4&node2=398671) |  | 0.780 | |  | [MAL13P1.168-1](http://string-db.org/newstring_cgi/display_single_node.pl?taskId=YKRYooI_NI_4&node=397036&targetmode=proteins) | hypothetical protein, conserved (267 aa) |  |  |  |  |  |  | [**•**](http://string-db.org/newstring_cgi/show_textmining_evidence.pl?taskId=YKRYooI_NI_4&node2=397036) |  | 0.768 | |  | [PFC0380w](http://string-db.org/newstring_cgi/display_single_node.pl?taskId=YKRYooI_NI_4&node=400544&targetmode=proteins) | dual-specificity protein phosphatase, putative (575 aa) |  |  |  |  |  |  | [**•**](http://string-db.org/newstring_cgi/show_textmining_evidence.pl?taskId=YKRYooI_NI_4&node2=400544) |  | 0.679 | |  | [PTPS](http://string-db.org/newstring_cgi/display_single_node.pl?taskId=YKRYooI_NI_4&node=401559&targetmode=proteins) | 6-pyruvoyl tetrahydropterin synthase, putative (173 aa) |  |  |  |  |  |  | [**•**](http://string-db.org/newstring_cgi/show_textmining_evidence.pl?taskId=YKRYooI_NI_4&node2=401559) |  | 0.659 | |  | [MAL13P1.275-1](http://string-db.org/newstring_cgi/display_single_node.pl?taskId=YKRYooI_NI_4&node=397149&targetmode=proteins) | NLI interacting factor-like phosphatase, putative (1288 aa) |  |  |  | [**•**](http://string-db.org/newstring_cgi/show_coexpression_evidence.pl?taskId=YKRYooI_NI_4&node2=397149) |  |  | [**•**](http://string-db.org/newstring_cgi/show_textmining_evidence.pl?taskId=YKRYooI_NI_4&node2=397149) |  | 0.510 | |  | [lytB](http://string-db.org/newstring_cgi/display_single_node.pl?taskId=YKRYooI_NI_4&node=400118&targetmode=proteins) | LytB protein (535 aa) | [**•**](http://string-db.org/newstring_cgi/show_neighborhood.pl?taskId=YKRYooI_NI_4&node2=400118) |  |  |  |  |  |  |  | 0.501 | |  | [PFL0320w](http://string-db.org/newstring_cgi/display_single_node.pl?taskId=YKRYooI_NI_4&node=402049&targetmode=proteins) | hypothetical protein, conserved (346 aa) |  |  |  |  |  |  | [**•**](http://string-db.org/newstring_cgi/show_textmining_evidence.pl?taskId=YKRYooI_NI_4&node2=402049) |  | 0.486 | |  | [PF13_0027](http://string-db.org/newstring_cgi/display_single_node.pl?taskId=YKRYooI_NI_4&node=398952&targetmode=proteins) | hypothetical protein, conserved (771 aa) |  |  |  |  |  |  | [**•**](http://string-db.org/newstring_cgi/show_textmining_evidence.pl?taskId=YKRYooI_NI_4&node2=398952) |  | 0.486 | |  | [PBGS](http://string-db.org/newstring_cgi/display_single_node.pl?taskId=YKRYooI_NI_4&node=399657&targetmode=proteins) | delta-aminolevulinic acid dehydratase (451 aa) | [**•**](http://string-db.org/newstring_cgi/show_neighborhood.pl?taskId=YKRYooI_NI_4&node2=399657) |  |  |  |  |  | [**•**](http://string-db.org/newstring_cgi/show_textmining_evidence.pl?taskId=YKRYooI_NI_4&node2=399657) |  | 0.481 | |
| --- | --- | --- | --- | --- | --- | --- | --- | --- | --- | --- | --- | --- | --- | --- | --- | --- | --- | --- | --- | --- | --- | --- | --- | --- | --- | --- | --- | --- | --- | --- | --- | --- | --- | --- | --- | --- | --- | --- | --- | --- | --- | --- | --- | --- | --- | --- | --- | --- | --- | --- | --- | --- | --- | --- | --- | --- | --- | --- | --- | --- | --- | --- | --- | --- | --- | --- | --- | --- | --- | --- | --- | --- | --- | --- | --- | --- | --- | --- | --- | --- | --- | --- | --- | --- | --- | --- | --- | --- | --- | --- | --- | --- | --- | --- | --- | --- | --- | --- | --- | --- | --- | --- | --- | --- | --- | --- | --- | --- | --- | --- | --- | --- | --- | --- | --- | --- | --- | --- | --- | --- | --- | --- | --- | --- | --- | --- | --- | --- | --- | --- | --- | --- | --- | --- | --- | --- | --- | --- | --- | --- | --- |

32.

| | | | **Your Input:** **PF3D7_1120100** | | |  |  |  |  |  |  |  |  |  | | --- | --- | --- | --- | --- | --- | --- | --- | --- | --- | --- | --- | |  | [PF11_0208](http://string-db.org/newstring_cgi/display_single_node.pl?taskId=jubfsMdYIZiH&node=398597&targetmode=proteins) | phosphoglycerate mutase, putative (250 aa) | |  |  | *(Plasmodium falciparum)* | | **Predicted Functional Partners:** | | | |  | [ENO](http://string-db.org/newstring_cgi/display_single_node.pl?taskId=jubfsMdYIZiH&node=398140&targetmode=proteins) | enolase (446 aa) | [**•**](http://string-db.org/newstring_cgi/show_neighborhood.pl?taskId=jubfsMdYIZiH&node2=398140) |  |  | [**•**](http://string-db.org/newstring_cgi/show_coexpression_evidence.pl?taskId=jubfsMdYIZiH&node2=398140) | [**•**](http://string-db.org/newstring_cgi/show_set_evidence.pl?data_channel=experimental&taskId=jubfsMdYIZiH&node2=398140) | [**•**](http://string-db.org/newstring_cgi/show_set_evidence.pl?data_channel=database&taskId=jubfsMdYIZiH&node2=398140) | [**•**](http://string-db.org/newstring_cgi/show_textmining_evidence.pl?taskId=jubfsMdYIZiH&node2=398140) |  | 0.999 | |  | [PGK](http://string-db.org/newstring_cgi/display_single_node.pl?taskId=jubfsMdYIZiH&node=401833&targetmode=proteins) | Phosphoglycerate kinase (416 aa) |  |  |  | [**•**](http://string-db.org/newstring_cgi/show_coexpression_evidence.pl?taskId=jubfsMdYIZiH&node2=401833) | [**•**](http://string-db.org/newstring_cgi/show_set_evidence.pl?data_channel=experimental&taskId=jubfsMdYIZiH&node2=401833) | [**•**](http://string-db.org/newstring_cgi/show_set_evidence.pl?data_channel=database&taskId=jubfsMdYIZiH&node2=401833) | [**•**](http://string-db.org/newstring_cgi/show_textmining_evidence.pl?taskId=jubfsMdYIZiH&node2=401833) |  | 0.999 | |  | [TPI](http://string-db.org/newstring_cgi/display_single_node.pl?taskId=jubfsMdYIZiH&node=399654&targetmode=proteins) | triose-phosphate isomerase (248 aa) | [**•**](http://string-db.org/newstring_cgi/show_neighborhood.pl?taskId=jubfsMdYIZiH&node2=399654) |  |  | [**•**](http://string-db.org/newstring_cgi/show_coexpression_evidence.pl?taskId=jubfsMdYIZiH&node2=399654) |  | [**•**](http://string-db.org/newstring_cgi/show_set_evidence.pl?data_channel=database&taskId=jubfsMdYIZiH&node2=399654) | [**•**](http://string-db.org/newstring_cgi/show_textmining_evidence.pl?taskId=jubfsMdYIZiH&node2=399654) |  | 0.998 | |  | [PF14_0341](http://string-db.org/newstring_cgi/display_single_node.pl?taskId=jubfsMdYIZiH&node=399615&targetmode=proteins) | glucose-6-phosphate isomerase (579 aa) | [**•**](http://string-db.org/newstring_cgi/show_neighborhood.pl?taskId=jubfsMdYIZiH&node2=399615) |  |  | [**•**](http://string-db.org/newstring_cgi/show_coexpression_evidence.pl?taskId=jubfsMdYIZiH&node2=399615) |  | [**•**](http://string-db.org/newstring_cgi/show_set_evidence.pl?data_channel=database&taskId=jubfsMdYIZiH&node2=399615) | [**•**](http://string-db.org/newstring_cgi/show_textmining_evidence.pl?taskId=jubfsMdYIZiH&node2=399615) |  | 0.996 | |  | [GAPDH](http://string-db.org/newstring_cgi/display_single_node.pl?taskId=jubfsMdYIZiH&node=399879&targetmode=proteins) | glyceraldehyde-3-phosphate dehydrogenase (337 aa) | [**•**](http://string-db.org/newstring_cgi/show_neighborhood.pl?taskId=jubfsMdYIZiH&node2=399879) |  |  | [**•**](http://string-db.org/newstring_cgi/show_coexpression_evidence.pl?taskId=jubfsMdYIZiH&node2=399879) | [**•**](http://string-db.org/newstring_cgi/show_set_evidence.pl?data_channel=experimental&taskId=jubfsMdYIZiH&node2=399879) | [**•**](http://string-db.org/newstring_cgi/show_set_evidence.pl?data_channel=database&taskId=jubfsMdYIZiH&node2=399879) | [**•**](http://string-db.org/newstring_cgi/show_textmining_evidence.pl?taskId=jubfsMdYIZiH&node2=399879) |  | 0.996 | |  | [PFF1300w](http://string-db.org/newstring_cgi/display_single_node.pl?taskId=jubfsMdYIZiH&node=401547&targetmode=proteins) | pyruvate kinase, putative (511 aa) | [**•**](http://string-db.org/newstring_cgi/show_neighborhood.pl?taskId=jubfsMdYIZiH&node2=401547) |  |  | [**•**](http://string-db.org/newstring_cgi/show_coexpression_evidence.pl?taskId=jubfsMdYIZiH&node2=401547) |  |  | [**•**](http://string-db.org/newstring_cgi/show_textmining_evidence.pl?taskId=jubfsMdYIZiH&node2=401547) |  | 0.993 | |  | [PfTK](http://string-db.org/newstring_cgi/display_single_node.pl?taskId=jubfsMdYIZiH&node=401389&targetmode=proteins) | transketolase, putative (672 aa) | [**•**](http://string-db.org/newstring_cgi/show_neighborhood.pl?taskId=jubfsMdYIZiH&node2=401389) |  |  | [**•**](http://string-db.org/newstring_cgi/show_coexpression_evidence.pl?taskId=jubfsMdYIZiH&node2=401389) |  | [**•**](http://string-db.org/newstring_cgi/show_set_evidence.pl?data_channel=database&taskId=jubfsMdYIZiH&node2=401389) | [**•**](http://string-db.org/newstring_cgi/show_textmining_evidence.pl?taskId=jubfsMdYIZiH&node2=401389) |  | 0.964 | |  | [PF14_0425](http://string-db.org/newstring_cgi/display_single_node.pl?taskId=jubfsMdYIZiH&node=399701&targetmode=proteins) | fructose-bisphosphate aldolase (369 aa) |  |  |  | [**•**](http://string-db.org/newstring_cgi/show_coexpression_evidence.pl?taskId=jubfsMdYIZiH&node2=399701) | [**•**](http://string-db.org/newstring_cgi/show_set_evidence.pl?data_channel=experimental&taskId=jubfsMdYIZiH&node2=399701) | [**•**](http://string-db.org/newstring_cgi/show_set_evidence.pl?data_channel=database&taskId=jubfsMdYIZiH&node2=399701) | [**•**](http://string-db.org/newstring_cgi/show_textmining_evidence.pl?taskId=jubfsMdYIZiH&node2=399701) |  | 0.913 | |  | [PFD0660w](http://string-db.org/newstring_cgi/display_single_node.pl?taskId=jubfsMdYIZiH&node=400832&targetmode=proteins) | phosphoglycerate mutase, putative (295 aa) |  |  |  |  |  | [**•**](http://string-db.org/newstring_cgi/show_set_evidence.pl?data_channel=database&taskId=jubfsMdYIZiH&node2=400832) | [**•**](http://string-db.org/newstring_cgi/show_textmining_evidence.pl?taskId=jubfsMdYIZiH&node2=400832) |  | 0.910 | |  | [PF08_0075](http://string-db.org/newstring_cgi/display_single_node.pl?taskId=jubfsMdYIZiH&node=397912&targetmode=proteins) | 60S ribosomal protein L13, putative (215 aa) |  |  |  | [**•**](http://string-db.org/newstring_cgi/show_coexpression_evidence.pl?taskId=jubfsMdYIZiH&node2=397912) |  |  |  |  | 0.899 | |  | [PfLDH](http://string-db.org/newstring_cgi/display_single_node.pl?taskId=jubfsMdYIZiH&node=399057&targetmode=proteins) | L-lactate dehydrogenase (316 aa) |  |  |  | [**•**](http://string-db.org/newstring_cgi/show_coexpression_evidence.pl?taskId=jubfsMdYIZiH&node2=399057) | [**•**](http://string-db.org/newstring_cgi/show_set_evidence.pl?data_channel=experimental&taskId=jubfsMdYIZiH&node2=399057) |  | [**•**](http://string-db.org/newstring_cgi/show_textmining_evidence.pl?taskId=jubfsMdYIZiH&node2=399057) |  | 0.872 | |  | [PFE0730c](http://string-db.org/newstring_cgi/display_single_node.pl?taskId=jubfsMdYIZiH&node=401104&targetmode=proteins) | ribose 5-phosphate epimerase, putative (236 aa) | [**•**](http://string-db.org/newstring_cgi/show_neighborhood.pl?taskId=jubfsMdYIZiH&node2=401104) |  |  |  |  |  | [**•**](http://string-db.org/newstring_cgi/show_textmining_evidence.pl?taskId=jubfsMdYIZiH&node2=401104) |  | 0.872 | |  | [adsS](http://string-db.org/newstring_cgi/display_single_node.pl?taskId=jubfsMdYIZiH&node=399199&targetmode=proteins) | adenylosuccinate synthetase; Plays an important role in the de novo pathway of purine nucleotid [...] (442 aa) | [**•**](http://string-db.org/newstring_cgi/show_neighborhood.pl?taskId=jubfsMdYIZiH&node2=399199) |  |  | [**•**](http://string-db.org/newstring_cgi/show_coexpression_evidence.pl?taskId=jubfsMdYIZiH&node2=399199) |  |  | [**•**](http://string-db.org/newstring_cgi/show_textmining_evidence.pl?taskId=jubfsMdYIZiH&node2=399199) |  | 0.856 | |  | [PFD0770c](http://string-db.org/newstring_cgi/display_single_node.pl?taskId=jubfsMdYIZiH&node=400856&targetmode=proteins) | ribosomal protein l15, putative (220 aa) |  |  |  | [**•**](http://string-db.org/newstring_cgi/show_coexpression_evidence.pl?taskId=jubfsMdYIZiH&node2=400856) |  |  | [**•**](http://string-db.org/newstring_cgi/show_textmining_evidence.pl?taskId=jubfsMdYIZiH&node2=400856) |  | 0.842 | |  | [PF08_0045](http://string-db.org/newstring_cgi/display_single_node.pl?taskId=jubfsMdYIZiH&node=397882&targetmode=proteins) | 2-oxoglutarate dehydrogenase e1 component (1038 aa) | [**•**](http://string-db.org/newstring_cgi/show_neighborhood.pl?taskId=jubfsMdYIZiH&node2=397882) |  |  |  |  |  | [**•**](http://string-db.org/newstring_cgi/show_textmining_evidence.pl?taskId=jubfsMdYIZiH&node2=397882) |  | 0.833 | |  | [PF10_0210](http://string-db.org/newstring_cgi/display_single_node.pl?taskId=jubfsMdYIZiH&node=398197&targetmode=proteins) | deoxyribose-phosphate aldolase, putative (263 aa) |  |  |  | [**•**](http://string-db.org/newstring_cgi/show_coexpression_evidence.pl?taskId=jubfsMdYIZiH&node2=398197) |  | [**•**](http://string-db.org/newstring_cgi/show_set_evidence.pl?data_channel=database&taskId=jubfsMdYIZiH&node2=398197) | [**•**](http://string-db.org/newstring_cgi/show_textmining_evidence.pl?taskId=jubfsMdYIZiH&node2=398197) |  | 0.832 | |  | [PfRACK](http://string-db.org/newstring_cgi/display_single_node.pl?taskId=jubfsMdYIZiH&node=397856&targetmode=proteins) | receptor for activated C kinase homolog, PfRACK (323 aa) |  |  |  | [**•**](http://string-db.org/newstring_cgi/show_coexpression_evidence.pl?taskId=jubfsMdYIZiH&node2=397856) |  |  |  |  | 0.816 | |  | [PFL1720w](http://string-db.org/newstring_cgi/display_single_node.pl?taskId=jubfsMdYIZiH&node=402337&targetmode=proteins) | Serine hydroxymethyltransferase (442 aa) | [**•**](http://string-db.org/newstring_cgi/show_neighborhood.pl?taskId=jubfsMdYIZiH&node2=402337) |  |  | [**•**](http://string-db.org/newstring_cgi/show_coexpression_evidence.pl?taskId=jubfsMdYIZiH&node2=402337) |  |  | [**•**](http://string-db.org/newstring_cgi/show_textmining_evidence.pl?taskId=jubfsMdYIZiH&node2=402337) |  | 0.809 | |  | [Cpn10](http://string-db.org/newstring_cgi/display_single_node.pl?taskId=jubfsMdYIZiH&node=402135&targetmode=proteins) | 10 kd chaperonin, putative (91 aa) | [**•**](http://string-db.org/newstring_cgi/show_neighborhood.pl?taskId=jubfsMdYIZiH&node2=402135) |  |  | [**•**](http://string-db.org/newstring_cgi/show_coexpression_evidence.pl?taskId=jubfsMdYIZiH&node2=402135) |  |  | [**•**](http://string-db.org/newstring_cgi/show_textmining_evidence.pl?taskId=jubfsMdYIZiH&node2=402135) |  | 0.799 | |  | [TPx1](http://string-db.org/newstring_cgi/display_single_node.pl?taskId=jubfsMdYIZiH&node=399644&targetmode=proteins) | 2-Cys peroxiredoxin (195 aa) | [**•**](http://string-db.org/newstring_cgi/show_neighborhood.pl?taskId=jubfsMdYIZiH&node2=399644) |  |  | [**•**](http://string-db.org/newstring_cgi/show_coexpression_evidence.pl?taskId=jubfsMdYIZiH&node2=399644) | [**•**](http://string-db.org/newstring_cgi/show_set_evidence.pl?data_channel=experimental&taskId=jubfsMdYIZiH&node2=399644) |  | [**•**](http://string-db.org/newstring_cgi/show_textmining_evidence.pl?taskId=jubfsMdYIZiH&node2=399644) |  | 0.791 | | | --- | --- | --- | --- | --- | --- | --- | --- | --- | --- | --- | --- | --- | --- | --- | --- | --- | --- | --- | --- | --- | --- | --- | --- | --- | --- | --- | --- | --- | --- | --- | --- | --- | --- | --- | --- | --- | --- | --- | --- | --- | --- | --- | --- | --- | --- | --- | --- | --- | --- | --- | --- | --- | --- | --- | --- | --- | --- | --- | --- | --- | --- | --- | --- | --- | --- | --- | --- | --- | --- | --- | --- | --- | --- | --- | --- | --- | --- | --- | --- | --- | --- | --- | --- | --- | --- | --- | --- | --- | --- | --- | --- | --- | --- | --- | --- | --- | --- | --- | --- | --- | --- | --- | --- | --- | --- | --- | --- | --- | --- | --- | --- | --- | --- | --- | --- | --- | --- | --- | --- | --- | --- | --- | --- | --- | --- | --- | --- | --- | --- | --- | --- | --- | --- | --- | --- | --- | --- | --- | --- | --- | --- | --- | --- | --- | --- | --- | --- | --- | --- | --- | --- | --- | --- | --- | --- | --- | --- | --- | --- | --- | --- | --- | --- | --- | --- | --- | --- | --- | --- | --- | --- | --- | --- | --- | --- | --- | --- | --- | --- | --- | --- | --- | --- | --- | --- | --- | --- | --- | --- | --- | --- | --- | --- | --- | --- | --- | --- | --- | --- | --- | --- | --- | --- | --- | --- | --- | --- | --- | --- | --- | --- | --- | --- | --- | --- | --- | --- | --- | --- | --- | --- | --- | --- | --- | --- | --- | --- | --- | --- | --- | --- | --- | --- | --- | --- | --- | --- | --- | --- | --- | --- | --- | --- | --- | --- | --- | --- | --- | --- | --- | --- | --- | --- | --- | --- | --- | --- | --- | --- | --- | --- | | | --- | --- | --- | --- | --- | --- | --- | --- | --- | --- | --- | --- | --- | --- | --- | --- | --- | --- | --- | --- | --- | --- | --- | --- | --- | --- | --- | --- | --- | --- | --- | --- | --- | --- | --- | --- | --- | --- | --- | --- | --- | --- | --- | --- | --- | --- | --- | --- | --- | --- | --- | --- | --- | --- | --- | --- | --- | --- | --- | --- | --- | --- | --- | --- | --- | --- | --- | --- | --- | --- | --- | --- | --- | --- | --- | --- | --- | --- | --- | --- | --- | --- | --- | --- | --- | --- | --- | --- | --- | --- | --- | --- | --- | --- | --- | --- | --- | --- | --- | --- | --- | --- | --- | --- | --- | --- | --- | --- | --- | --- | --- | --- | --- | --- | --- | --- | --- | --- | --- | --- | --- | --- | --- | --- | --- | --- | --- | --- | --- | --- | --- | --- | --- | --- | --- | --- | --- | --- | --- | --- | --- | --- | --- | --- | --- | --- | --- | --- | --- | --- | --- | --- | --- | --- | --- | --- | --- | --- | --- | --- | --- | --- | --- | --- | --- | --- | --- | --- | --- | --- | --- | --- | --- | --- | --- | --- | --- | --- | --- | --- | --- | --- | --- | --- | --- | --- | --- | --- | --- | --- | --- | --- | --- | --- | --- | --- | --- | --- | --- | --- | --- | --- | --- | --- | --- | --- | --- | --- | --- | --- | --- | --- | --- | --- | --- | --- | --- | --- | --- | --- | --- | --- | --- | --- | --- | --- | --- | --- | --- | --- | --- | --- | --- | --- | --- | --- | --- | --- | --- | --- | --- | --- | --- | --- | --- | --- | --- | --- | --- | --- | --- | --- | --- | --- | --- | --- | --- | --- | --- | --- | --- | --- | --- | |
| --- | --- | --- | --- | --- | --- | --- | --- | --- | --- | --- | --- | --- | --- | --- | --- | --- | --- | --- | --- | --- | --- | --- | --- | --- | --- | --- | --- | --- | --- | --- | --- | --- | --- | --- | --- | --- | --- | --- | --- | --- | --- | --- | --- | --- | --- | --- | --- | --- | --- | --- | --- | --- | --- | --- | --- | --- | --- | --- | --- | --- | --- | --- | --- | --- | --- | --- | --- | --- | --- | --- | --- | --- | --- | --- | --- | --- | --- | --- | --- | --- | --- | --- | --- | --- | --- | --- | --- | --- | --- | --- | --- | --- | --- | --- | --- | --- | --- | --- | --- | --- | --- | --- | --- | --- | --- | --- | --- | --- | --- | --- | --- | --- | --- | --- | --- | --- | --- | --- | --- | --- | --- | --- | --- | --- | --- | --- | --- | --- | --- | --- | --- | --- | --- | --- | --- | --- | --- | --- | --- | --- | --- | --- | --- | --- | --- | --- | --- | --- | --- | --- | --- | --- | --- | --- | --- | --- | --- | --- | --- | --- | --- | --- | --- | --- | --- | --- | --- | --- | --- | --- | --- | --- | --- | --- | --- | --- | --- | --- | --- | --- | --- | --- | --- | --- | --- | --- | --- | --- | --- | --- | --- | --- | --- | --- | --- | --- | --- | --- | --- | --- | --- | --- | --- | --- | --- | --- | --- | --- | --- | --- | --- | --- | --- | --- | --- | --- | --- | --- | --- | --- | --- | --- | --- | --- | --- | --- | --- | --- | --- | --- | --- | --- | --- | --- | --- | --- | --- | --- | --- | --- | --- | --- | --- | --- | --- | --- | --- | --- | --- | --- | --- | --- | --- | --- | --- | --- | --- | --- | --- | --- | --- | --- | --- |
| |  | | --- | |

33.

| | | | **Your Input:** **>PF3D7_1127000** | | |  |  |  |  |  |  |  |  |  | | --- | --- | --- | --- | --- | --- | --- | --- | --- | --- | --- | --- | |  | [PF11_0281](http://string-db.org/newstring_cgi/display_single_node.pl?taskId=5Dk9c9_Yz7g_&node=398671&targetmode=proteins) | hypothetical protein (247 aa) | |  |  | *(Plasmodium falciparum)* | | **Predicted Functional Partners:** | | | |  | [PF14_0525](http://string-db.org/newstring_cgi/display_single_node.pl?taskId=5Dk9c9_Yz7g_&node=399804&targetmode=proteins) | hypothetical protein (89 aa) |  |  |  |  |  |  | [**•**](http://string-db.org/newstring_cgi/show_textmining_evidence.pl?taskId=5Dk9c9_Yz7g_&node2=399804) |  | 0.786 | |  | [PRL](http://string-db.org/newstring_cgi/display_single_node.pl?taskId=5Dk9c9_Yz7g_&node=398526&targetmode=proteins) | protein tyrosine phosphatase, putative (218 aa) |  |  |  |  |  |  | [**•**](http://string-db.org/newstring_cgi/show_textmining_evidence.pl?taskId=5Dk9c9_Yz7g_&node2=398526) |  | 0.780 | |  | [PFC0380w](http://string-db.org/newstring_cgi/display_single_node.pl?taskId=5Dk9c9_Yz7g_&node=400544&targetmode=proteins) | dual-specificity protein phosphatase, putative (575 aa) |  |  |  |  |  |  | [**•**](http://string-db.org/newstring_cgi/show_textmining_evidence.pl?taskId=5Dk9c9_Yz7g_&node2=400544) |  | 0.685 | |  | [PFL0320w](http://string-db.org/newstring_cgi/display_single_node.pl?taskId=5Dk9c9_Yz7g_&node=402049&targetmode=proteins) | hypothetical protein, conserved (346 aa) |  |  |  |  |  |  | [**•**](http://string-db.org/newstring_cgi/show_textmining_evidence.pl?taskId=5Dk9c9_Yz7g_&node2=402049) |  | 0.579 | |  | [PF13_0027](http://string-db.org/newstring_cgi/display_single_node.pl?taskId=5Dk9c9_Yz7g_&node=398952&targetmode=proteins) | hypothetical protein, conserved (771 aa) |  |  |  |  |  |  | [**•**](http://string-db.org/newstring_cgi/show_textmining_evidence.pl?taskId=5Dk9c9_Yz7g_&node2=398952) |  | 0.579 | |  | [MAL13P1.275-1](http://string-db.org/newstring_cgi/display_single_node.pl?taskId=5Dk9c9_Yz7g_&node=397149&targetmode=proteins) | NLI interacting factor-like phosphatase, putative (1288 aa) |  |  |  |  |  |  | [**•**](http://string-db.org/newstring_cgi/show_textmining_evidence.pl?taskId=5Dk9c9_Yz7g_&node2=397149) |  | 0.579 | |  | [MAL13P1.168-1](http://string-db.org/newstring_cgi/display_single_node.pl?taskId=5Dk9c9_Yz7g_&node=397036&targetmode=proteins) | hypothetical protein, conserved (267 aa) |  |  |  |  |  |  | [**•**](http://string-db.org/newstring_cgi/show_textmining_evidence.pl?taskId=5Dk9c9_Yz7g_&node2=397036) |  | 0.579 | |  | [PFL2365w](http://string-db.org/newstring_cgi/display_single_node.pl?taskId=5Dk9c9_Yz7g_&node=402468&targetmode=proteins) | hypothetical protein, conserved (1027 aa) |  |  |  |  |  |  | [**•**](http://string-db.org/newstring_cgi/show_textmining_evidence.pl?taskId=5Dk9c9_Yz7g_&node2=402468) |  | 0.484 | |  | [PF14_0523](http://string-db.org/newstring_cgi/display_single_node.pl?taskId=5Dk9c9_Yz7g_&node=399802&targetmode=proteins) | protein phosphatase 2C, putative (289 aa) |  |  |  |  |  |  | [**•**](http://string-db.org/newstring_cgi/show_textmining_evidence.pl?taskId=5Dk9c9_Yz7g_&node2=399802) |  | 0.484 | |  | [MAL8P1.108](http://string-db.org/newstring_cgi/display_single_node.pl?taskId=5Dk9c9_Yz7g_&node=397533&targetmode=proteins) | protein phosphatase, putative (303 aa) |  |  |  |  |  |  | [**•**](http://string-db.org/newstring_cgi/show_textmining_evidence.pl?taskId=5Dk9c9_Yz7g_&node2=397533) |  | 0.484 | | | --- | --- | --- | --- | --- | --- | --- | --- | --- | --- | --- | --- | --- | --- | --- | --- | --- | --- | --- | --- | --- | --- | --- | --- | --- | --- | --- | --- | --- | --- | --- | --- | --- | --- | --- | --- | --- | --- | --- | --- | --- | --- | --- | --- | --- | --- | --- | --- | --- | --- | --- | --- | --- | --- | --- | --- | --- | --- | --- | --- | --- | --- | --- | --- | --- | --- | --- | --- | --- | --- | --- | --- | --- | --- | --- | --- | --- | --- | --- | --- | --- | --- | --- | --- | --- | --- | --- | --- | --- | --- | --- | --- | --- | --- | --- | --- | --- | --- | --- | --- | --- | --- | --- | --- | --- | --- | --- | --- | --- | --- | --- | --- | --- | --- | --- | --- | --- | --- | --- | --- | --- | --- | --- | --- | --- | --- | --- | --- | --- | --- | --- | --- | --- | --- | --- | --- | --- | --- | --- | --- | --- | --- | | | --- | --- | --- | --- | --- | --- | --- | --- | --- | --- | --- | --- | --- | --- | --- | --- | --- | --- | --- | --- | --- | --- | --- | --- | --- | --- | --- | --- | --- | --- | --- | --- | --- | --- | --- | --- | --- | --- | --- | --- | --- | --- | --- | --- | --- | --- | --- | --- | --- | --- | --- | --- | --- | --- | --- | --- | --- | --- | --- | --- | --- | --- | --- | --- | --- | --- | --- | --- | --- | --- | --- | --- | --- | --- | --- | --- | --- | --- | --- | --- | --- | --- | --- | --- | --- | --- | --- | --- | --- | --- | --- | --- | --- | --- | --- | --- | --- | --- | --- | --- | --- | --- | --- | --- | --- | --- | --- | --- | --- | --- | --- | --- | --- | --- | --- | --- | --- | --- | --- | --- | --- | --- | --- | --- | --- | --- | --- | --- | --- | --- | --- | --- | --- | --- | --- | --- | --- | --- | --- | --- | --- | --- | --- | |
| --- | --- | --- | --- | --- | --- | --- | --- | --- | --- | --- | --- | --- | --- | --- | --- | --- | --- | --- | --- | --- | --- | --- | --- | --- | --- | --- | --- | --- | --- | --- | --- | --- | --- | --- | --- | --- | --- | --- | --- | --- | --- | --- | --- | --- | --- | --- | --- | --- | --- | --- | --- | --- | --- | --- | --- | --- | --- | --- | --- | --- | --- | --- | --- | --- | --- | --- | --- | --- | --- | --- | --- | --- | --- | --- | --- | --- | --- | --- | --- | --- | --- | --- | --- | --- | --- | --- | --- | --- | --- | --- | --- | --- | --- | --- | --- | --- | --- | --- | --- | --- | --- | --- | --- | --- | --- | --- | --- | --- | --- | --- | --- | --- | --- | --- | --- | --- | --- | --- | --- | --- | --- | --- | --- | --- | --- | --- | --- | --- | --- | --- | --- | --- | --- | --- | --- | --- | --- | --- | --- | --- | --- | --- | --- |
| |  | | --- | |

34.

| | | | **Your Input:** **PF3D7_1135100** | | |  |  |  |  |  |  |  |  |  | | --- | --- | --- | --- | --- | --- | --- | --- | --- | --- | --- | --- | |  | [PF11_0362](http://string-db.org/newstring_cgi/display_single_node.pl?taskId=rNxiq_bIxgsy&node=398753&targetmode=proteins) | protein phosphatase, putative (689 aa) | |  |  | *(Plasmodium falciparum)* | | **Predicted Functional Partners:** | | | |  | [MAL13P1.44](http://string-db.org/newstring_cgi/display_single_node.pl?taskId=rNxiq_bIxgsy&node=397260&targetmode=proteins) | protein phosphatase 2c-like protein, putative (827 aa) |  |  |  | [**•**](http://string-db.org/newstring_cgi/show_coexpression_evidence.pl?taskId=rNxiq_bIxgsy&node2=397260) |  |  | [**•**](http://string-db.org/newstring_cgi/show_textmining_evidence.pl?taskId=rNxiq_bIxgsy&node2=397260) |  | 0.715 | |  | [MAL8P1.109](http://string-db.org/newstring_cgi/display_single_node.pl?taskId=rNxiq_bIxgsy&node=397534&targetmode=proteins) | Protein phosphatase 2C, putative (545 aa) |  |  |  | [**•**](http://string-db.org/newstring_cgi/show_coexpression_evidence.pl?taskId=rNxiq_bIxgsy&node2=397534) |  |  | [**•**](http://string-db.org/newstring_cgi/show_textmining_evidence.pl?taskId=rNxiq_bIxgsy&node2=397534) |  | 0.707 | |  | [PFE0795c](http://string-db.org/newstring_cgi/display_single_node.pl?taskId=rNxiq_bIxgsy&node=401117&targetmode=proteins) | nif-like protein, putative (328 aa) |  |  |  |  |  |  | [**•**](http://string-db.org/newstring_cgi/show_textmining_evidence.pl?taskId=rNxiq_bIxgsy&node2=401117) |  | 0.688 | |  | [PF14_0523](http://string-db.org/newstring_cgi/display_single_node.pl?taskId=rNxiq_bIxgsy&node=399802&targetmode=proteins) | protein phosphatase 2C, putative (289 aa) |  |  |  |  |  |  | [**•**](http://string-db.org/newstring_cgi/show_textmining_evidence.pl?taskId=rNxiq_bIxgsy&node2=399802) |  | 0.688 | |  | [PF10_0093](http://string-db.org/newstring_cgi/display_single_node.pl?taskId=rNxiq_bIxgsy&node=398077&targetmode=proteins) | hypothetical protein (345 aa) |  |  |  |  |  |  | [**•**](http://string-db.org/newstring_cgi/show_textmining_evidence.pl?taskId=rNxiq_bIxgsy&node2=398077) |  | 0.688 | |  | [PF07_0110](http://string-db.org/newstring_cgi/display_single_node.pl?taskId=rNxiq_bIxgsy&node=397814&targetmode=proteins) | hypothetical protein, conserved (519 aa) |  |  |  |  |  |  | [**•**](http://string-db.org/newstring_cgi/show_textmining_evidence.pl?taskId=rNxiq_bIxgsy&node2=397814) |  | 0.688 | |  | [MAL13P1.174-1](http://string-db.org/newstring_cgi/display_single_node.pl?taskId=rNxiq_bIxgsy&node=397043&targetmode=proteins) | MSP7-like protein (281 aa) |  |  |  |  |  |  | [**•**](http://string-db.org/newstring_cgi/show_textmining_evidence.pl?taskId=rNxiq_bIxgsy&node2=397043) |  | 0.687 | |  | [PF14_0018](http://string-db.org/newstring_cgi/display_single_node.pl?taskId=rNxiq_bIxgsy&node=399285&targetmode=proteins) | hypothetical protein (493 aa) |  |  |  | [**•**](http://string-db.org/newstring_cgi/show_coexpression_evidence.pl?taskId=rNxiq_bIxgsy&node2=399285) |  |  |  |  | 0.653 | |  | [PFI1245c](http://string-db.org/newstring_cgi/display_single_node.pl?taskId=rNxiq_bIxgsy&node=401862&targetmode=proteins) | Protein phosphatase-beta (466 aa) |  |  |  |  |  |  | [**•**](http://string-db.org/newstring_cgi/show_textmining_evidence.pl?taskId=rNxiq_bIxgsy&node2=401862) |  | 0.649 | |  | [PfPP5](http://string-db.org/newstring_cgi/display_single_node.pl?taskId=rNxiq_bIxgsy&node=397148&targetmode=proteins) | serine%2Fthreonine protein phosphatase pfPp5 (658 aa) |  |  |  |  |  |  | [**•**](http://string-db.org/newstring_cgi/show_textmining_evidence.pl?taskId=rNxiq_bIxgsy&node2=397148) |  | 0.587 | | | --- | --- | --- | --- | --- | --- | --- | --- | --- | --- | --- | --- | --- | --- | --- | --- | --- | --- | --- | --- | --- | --- | --- | --- | --- | --- | --- | --- | --- | --- | --- | --- | --- | --- | --- | --- | --- | --- | --- | --- | --- | --- | --- | --- | --- | --- | --- | --- | --- | --- | --- | --- | --- | --- | --- | --- | --- | --- | --- | --- | --- | --- | --- | --- | --- | --- | --- | --- | --- | --- | --- | --- | --- | --- | --- | --- | --- | --- | --- | --- | --- | --- | --- | --- | --- | --- | --- | --- | --- | --- | --- | --- | --- | --- | --- | --- | --- | --- | --- | --- | --- | --- | --- | --- | --- | --- | --- | --- | --- | --- | --- | --- | --- | --- | --- | --- | --- | --- | --- | --- | --- | --- | --- | --- | --- | --- | --- | --- | --- | --- | --- | --- | --- | --- | --- | --- | --- | --- | --- | --- | --- | --- | | | --- | --- | --- | --- | --- | --- | --- | --- | --- | --- | --- | --- | --- | --- | --- | --- | --- | --- | --- | --- | --- | --- | --- | --- | --- | --- | --- | --- | --- | --- | --- | --- | --- | --- | --- | --- | --- | --- | --- | --- | --- | --- | --- | --- | --- | --- | --- | --- | --- | --- | --- | --- | --- | --- | --- | --- | --- | --- | --- | --- | --- | --- | --- | --- | --- | --- | --- | --- | --- | --- | --- | --- | --- | --- | --- | --- | --- | --- | --- | --- | --- | --- | --- | --- | --- | --- | --- | --- | --- | --- | --- | --- | --- | --- | --- | --- | --- | --- | --- | --- | --- | --- | --- | --- | --- | --- | --- | --- | --- | --- | --- | --- | --- | --- | --- | --- | --- | --- | --- | --- | --- | --- | --- | --- | --- | --- | --- | --- | --- | --- | --- | --- | --- | --- | --- | --- | --- | --- | --- | --- | --- | --- | --- | |
| --- | --- | --- | --- | --- | --- | --- | --- | --- | --- | --- | --- | --- | --- | --- | --- | --- | --- | --- | --- | --- | --- | --- | --- | --- | --- | --- | --- | --- | --- | --- | --- | --- | --- | --- | --- | --- | --- | --- | --- | --- | --- | --- | --- | --- | --- | --- | --- | --- | --- | --- | --- | --- | --- | --- | --- | --- | --- | --- | --- | --- | --- | --- | --- | --- | --- | --- | --- | --- | --- | --- | --- | --- | --- | --- | --- | --- | --- | --- | --- | --- | --- | --- | --- | --- | --- | --- | --- | --- | --- | --- | --- | --- | --- | --- | --- | --- | --- | --- | --- | --- | --- | --- | --- | --- | --- | --- | --- | --- | --- | --- | --- | --- | --- | --- | --- | --- | --- | --- | --- | --- | --- | --- | --- | --- | --- | --- | --- | --- | --- | --- | --- | --- | --- | --- | --- | --- | --- | --- | --- | --- | --- | --- | --- |
| |  | | --- | |

35.

| | | | **Your Input:** **>PF3D7_1138500** | | |  |  |  |  |  |  |  |  |  | | --- | --- | --- | --- | --- | --- | --- | --- | --- | --- | --- | --- | |  | [PF11_0396](http://string-db.org/newstring_cgi/display_single_node.pl?taskId=rIopfw8UKtUX&node=398787&targetmode=proteins) | Protein phosphatase 2C (938 aa) | |  |  | *(Plasmodium falciparum)* | | **Predicted Functional Partners:** | | | |  | [PfEF-1beta](http://string-db.org/newstring_cgi/display_single_node.pl?taskId=rIopfw8UKtUX&node=401740&targetmode=proteins) | EF-1B (276 aa) |  |  |  |  |  |  | [**•**](http://string-db.org/newstring_cgi/show_textmining_evidence.pl?taskId=rIopfw8UKtUX&node2=401740) |  | 0.966 | |  | [PFC0380w](http://string-db.org/newstring_cgi/display_single_node.pl?taskId=rIopfw8UKtUX&node=400544&targetmode=proteins) | dual-specificity protein phosphatase, putative (575 aa) |  |  |  |  |  |  | [**•**](http://string-db.org/newstring_cgi/show_textmining_evidence.pl?taskId=rIopfw8UKtUX&node2=400544) |  | 0.683 | |  | [RON4](http://string-db.org/newstring_cgi/display_single_node.pl?taskId=rIopfw8UKtUX&node=398555&targetmode=proteins) | hypothetical protein (2966 aa) |  |  |  |  |  |  | [**•**](http://string-db.org/newstring_cgi/show_textmining_evidence.pl?taskId=rIopfw8UKtUX&node2=398555) |  | 0.647 | |  | [PfCyP24](http://string-db.org/newstring_cgi/display_single_node.pl?taskId=rIopfw8UKtUX&node=397958&targetmode=proteins) | peptidyl-prolyl cis-trans isomerase precursor; PPIases accelerate the folding of proteins. It c [...] (217 aa) |  |  |  |  |  |  | [**•**](http://string-db.org/newstring_cgi/show_textmining_evidence.pl?taskId=rIopfw8UKtUX&node2=397958) |  | 0.641 | |  | [PfRON2](http://string-db.org/newstring_cgi/display_single_node.pl?taskId=rIopfw8UKtUX&node=399773&targetmode=proteins) | hypothetical protein (2189 aa) |  |  |  |  |  |  | [**•**](http://string-db.org/newstring_cgi/show_textmining_evidence.pl?taskId=rIopfw8UKtUX&node2=399773) |  | 0.639 | |  | [PP1](http://string-db.org/newstring_cgi/display_single_node.pl?taskId=rIopfw8UKtUX&node=399412&targetmode=proteins) | serine%2Fthreonine protein phosphatase, putative (304 aa) |  |  |  |  |  |  | [**•**](http://string-db.org/newstring_cgi/show_textmining_evidence.pl?taskId=rIopfw8UKtUX&node2=399412) |  | 0.589 | |  | [PF10_0093](http://string-db.org/newstring_cgi/display_single_node.pl?taskId=rIopfw8UKtUX&node=398077&targetmode=proteins) | hypothetical protein (345 aa) |  |  |  |  |  |  | [**•**](http://string-db.org/newstring_cgi/show_textmining_evidence.pl?taskId=rIopfw8UKtUX&node2=398077) |  | 0.587 | |  | [PF14_0630](http://string-db.org/newstring_cgi/display_single_node.pl?taskId=rIopfw8UKtUX&node=399913&targetmode=proteins) | protein serine%2Fthreonine phosphatase (889 aa) |  |  |  |  |  |  | [**•**](http://string-db.org/newstring_cgi/show_textmining_evidence.pl?taskId=rIopfw8UKtUX&node2=399913) |  | 0.585 | |  | [vapA](http://string-db.org/newstring_cgi/display_single_node.pl?taskId=rIopfw8UKtUX&node=398988&targetmode=proteins) | vacuolar ATP synthase, catalytic subunit a; Catalytic subunit of the peripheral V1 complex of v [...] (611 aa) |  |  |  |  |  |  | [**•**](http://string-db.org/newstring_cgi/show_textmining_evidence.pl?taskId=rIopfw8UKtUX&node2=398988) |  | 0.583 | |  | [PFE0465c](http://string-db.org/newstring_cgi/display_single_node.pl?taskId=rIopfw8UKtUX&node=401052&targetmode=proteins) | RNA polymerase I (2914 aa) |  |  |  |  |  |  | [**•**](http://string-db.org/newstring_cgi/show_textmining_evidence.pl?taskId=rIopfw8UKtUX&node2=401052) |  | 0.579 | | | --- | --- | --- | --- | --- | --- | --- | --- | --- | --- | --- | --- | --- | --- | --- | --- | --- | --- | --- | --- | --- | --- | --- | --- | --- | --- | --- | --- | --- | --- | --- | --- | --- | --- | --- | --- | --- | --- | --- | --- | --- | --- | --- | --- | --- | --- | --- | --- | --- | --- | --- | --- | --- | --- | --- | --- | --- | --- | --- | --- | --- | --- | --- | --- | --- | --- | --- | --- | --- | --- | --- | --- | --- | --- | --- | --- | --- | --- | --- | --- | --- | --- | --- | --- | --- | --- | --- | --- | --- | --- | --- | --- | --- | --- | --- | --- | --- | --- | --- | --- | --- | --- | --- | --- | --- | --- | --- | --- | --- | --- | --- | --- | --- | --- | --- | --- | --- | --- | --- | --- | --- | --- | --- | --- | --- | --- | --- | --- | --- | --- | --- | --- | --- | --- | --- | --- | --- | --- | --- | --- | --- | --- | | | --- | --- | --- | --- | --- | --- | --- | --- | --- | --- | --- | --- | --- | --- | --- | --- | --- | --- | --- | --- | --- | --- | --- | --- | --- | --- | --- | --- | --- | --- | --- | --- | --- | --- | --- | --- | --- | --- | --- | --- | --- | --- | --- | --- | --- | --- | --- | --- | --- | --- | --- | --- | --- | --- | --- | --- | --- | --- | --- | --- | --- | --- | --- | --- | --- | --- | --- | --- | --- | --- | --- | --- | --- | --- | --- | --- | --- | --- | --- | --- | --- | --- | --- | --- | --- | --- | --- | --- | --- | --- | --- | --- | --- | --- | --- | --- | --- | --- | --- | --- | --- | --- | --- | --- | --- | --- | --- | --- | --- | --- | --- | --- | --- | --- | --- | --- | --- | --- | --- | --- | --- | --- | --- | --- | --- | --- | --- | --- | --- | --- | --- | --- | --- | --- | --- | --- | --- | --- | --- | --- | --- | --- | --- | |
| --- | --- | --- | --- | --- | --- | --- | --- | --- | --- | --- | --- | --- | --- | --- | --- | --- | --- | --- | --- | --- | --- | --- | --- | --- | --- | --- | --- | --- | --- | --- | --- | --- | --- | --- | --- | --- | --- | --- | --- | --- | --- | --- | --- | --- | --- | --- | --- | --- | --- | --- | --- | --- | --- | --- | --- | --- | --- | --- | --- | --- | --- | --- | --- | --- | --- | --- | --- | --- | --- | --- | --- | --- | --- | --- | --- | --- | --- | --- | --- | --- | --- | --- | --- | --- | --- | --- | --- | --- | --- | --- | --- | --- | --- | --- | --- | --- | --- | --- | --- | --- | --- | --- | --- | --- | --- | --- | --- | --- | --- | --- | --- | --- | --- | --- | --- | --- | --- | --- | --- | --- | --- | --- | --- | --- | --- | --- | --- | --- | --- | --- | --- | --- | --- | --- | --- | --- | --- | --- | --- | --- | --- | --- | --- |
|  |

36.

| | | | **Your Input:** **PF3D7_1206000** | | |  |  |  |  |  |  |  |  |  | | --- | --- | --- | --- | --- | --- | --- | --- | --- | --- | --- | --- | |  | [PFL0300c](http://string-db.org/newstring_cgi/display_single_node.pl?taskId=x2fhB446WfKt&node=402045&targetmode=proteins) | phosphoesterase, putative (304 aa) | |  |  | *(Plasmodium falciparum)* | | **Predicted Functional Partners:** | | | |  | [PFB0935w](http://string-db.org/newstring_cgi/display_single_node.pl?taskId=x2fhB446WfKt&node=400425&targetmode=proteins) | cytoadherence linked asexual protein 2 (1440 aa) |  |  |  | [**•**](http://string-db.org/newstring_cgi/show_coexpression_evidence.pl?taskId=x2fhB446WfKt&node2=400425) |  |  |  |  | 0.938 | |  | [PF08_0129](http://string-db.org/newstring_cgi/display_single_node.pl?taskId=x2fhB446WfKt&node=397966&targetmode=proteins) | protein phosphatase, putative (604 aa) |  |  |  | [**•**](http://string-db.org/newstring_cgi/show_coexpression_evidence.pl?taskId=x2fhB446WfKt&node2=397966) |  |  | [**•**](http://string-db.org/newstring_cgi/show_textmining_evidence.pl?taskId=x2fhB446WfKt&node2=397966) |  | 0.937 | |  | [PfRON2](http://string-db.org/newstring_cgi/display_single_node.pl?taskId=x2fhB446WfKt&node=399773&targetmode=proteins) | hypothetical protein (2189 aa) |  |  |  | [**•**](http://string-db.org/newstring_cgi/show_coexpression_evidence.pl?taskId=x2fhB446WfKt&node2=399773) |  |  |  |  | 0.921 | |  | [PP7](http://string-db.org/newstring_cgi/display_single_node.pl?taskId=x2fhB446WfKt&node=399495&targetmode=proteins) | PP1-like protein serine%2Fthreonine phosphatase (959 aa) |  |  |  | [**•**](http://string-db.org/newstring_cgi/show_coexpression_evidence.pl?taskId=x2fhB446WfKt&node2=399495) |  |  | [**•**](http://string-db.org/newstring_cgi/show_textmining_evidence.pl?taskId=x2fhB446WfKt&node2=399495) |  | 0.914 | |  | [PFF1365c](http://string-db.org/newstring_cgi/display_single_node.pl?taskId=x2fhB446WfKt&node=401560&targetmode=proteins) | HECT-domain (ubiquitin-transferase), putative; Putative E3 ubiquitin-protein ligase (By similar [...] (10286 aa) |  |  |  | [**•**](http://string-db.org/newstring_cgi/show_coexpression_evidence.pl?taskId=x2fhB446WfKt&node2=401560) |  |  |  |  | 0.897 | |  | [PF14_0607](http://string-db.org/newstring_cgi/display_single_node.pl?taskId=x2fhB446WfKt&node=399889&targetmode=proteins) | hypothetical protein (1068 aa) |  |  |  | [**•**](http://string-db.org/newstring_cgi/show_coexpression_evidence.pl?taskId=x2fhB446WfKt&node2=399889) |  |  |  |  | 0.889 | |  | [ROM4](http://string-db.org/newstring_cgi/display_single_node.pl?taskId=x2fhB446WfKt&node=401027&targetmode=proteins) | rhomboid protease, putative (759 aa) |  |  |  | [**•**](http://string-db.org/newstring_cgi/show_coexpression_evidence.pl?taskId=x2fhB446WfKt&node2=401027) |  |  |  |  | 0.863 | |  | [VP2](http://string-db.org/newstring_cgi/display_single_node.pl?taskId=x2fhB446WfKt&node=402333&targetmode=proteins) | V-type K%2B - independent h%2B -translocating inorganic pyrophosphatse (1044 aa) |  |  |  | [**•**](http://string-db.org/newstring_cgi/show_coexpression_evidence.pl?taskId=x2fhB446WfKt&node2=402333) |  |  |  |  | 0.861 | |  | [PF11_0528](http://string-db.org/newstring_cgi/display_single_node.pl?taskId=x2fhB446WfKt&node=398922&targetmode=proteins) | hypothetical protein (5922 aa) |  |  |  | [**•**](http://string-db.org/newstring_cgi/show_coexpression_evidence.pl?taskId=x2fhB446WfKt&node2=398922) |  |  |  |  | 0.861 | |  | [PF10_0220](http://string-db.org/newstring_cgi/display_single_node.pl?taskId=x2fhB446WfKt&node=398209&targetmode=proteins) | hypothetical protein (275 aa) |  |  |  | [**•**](http://string-db.org/newstring_cgi/show_coexpression_evidence.pl?taskId=x2fhB446WfKt&node2=398209) |  |  |  |  | 0.861 | | | --- | --- | --- | --- | --- | --- | --- | --- | --- | --- | --- | --- | --- | --- | --- | --- | --- | --- | --- | --- | --- | --- | --- | --- | --- | --- | --- | --- | --- | --- | --- | --- | --- | --- | --- | --- | --- | --- | --- | --- | --- | --- | --- | --- | --- | --- | --- | --- | --- | --- | --- | --- | --- | --- | --- | --- | --- | --- | --- | --- | --- | --- | --- | --- | --- | --- | --- | --- | --- | --- | --- | --- | --- | --- | --- | --- | --- | --- | --- | --- | --- | --- | --- | --- | --- | --- | --- | --- | --- | --- | --- | --- | --- | --- | --- | --- | --- | --- | --- | --- | --- | --- | --- | --- | --- | --- | --- | --- | --- | --- | --- | --- | --- | --- | --- | --- | --- | --- | --- | --- | --- | --- | --- | --- | --- | --- | --- | --- | --- | --- | --- | --- | --- | --- | --- | --- | --- | --- | --- | --- | --- | --- | | | --- | --- | --- | --- | --- | --- | --- | --- | --- | --- | --- | --- | --- | --- | --- | --- | --- | --- | --- | --- | --- | --- | --- | --- | --- | --- | --- | --- | --- | --- | --- | --- | --- | --- | --- | --- | --- | --- | --- | --- | --- | --- | --- | --- | --- | --- | --- | --- | --- | --- | --- | --- | --- | --- | --- | --- | --- | --- | --- | --- | --- | --- | --- | --- | --- | --- | --- | --- | --- | --- | --- | --- | --- | --- | --- | --- | --- | --- | --- | --- | --- | --- | --- | --- | --- | --- | --- | --- | --- | --- | --- | --- | --- | --- | --- | --- | --- | --- | --- | --- | --- | --- | --- | --- | --- | --- | --- | --- | --- | --- | --- | --- | --- | --- | --- | --- | --- | --- | --- | --- | --- | --- | --- | --- | --- | --- | --- | --- | --- | --- | --- | --- | --- | --- | --- | --- | --- | --- | --- | --- | --- | --- | --- | |
| --- | --- | --- | --- | --- | --- | --- | --- | --- | --- | --- | --- | --- | --- | --- | --- | --- | --- | --- | --- | --- | --- | --- | --- | --- | --- | --- | --- | --- | --- | --- | --- | --- | --- | --- | --- | --- | --- | --- | --- | --- | --- | --- | --- | --- | --- | --- | --- | --- | --- | --- | --- | --- | --- | --- | --- | --- | --- | --- | --- | --- | --- | --- | --- | --- | --- | --- | --- | --- | --- | --- | --- | --- | --- | --- | --- | --- | --- | --- | --- | --- | --- | --- | --- | --- | --- | --- | --- | --- | --- | --- | --- | --- | --- | --- | --- | --- | --- | --- | --- | --- | --- | --- | --- | --- | --- | --- | --- | --- | --- | --- | --- | --- | --- | --- | --- | --- | --- | --- | --- | --- | --- | --- | --- | --- | --- | --- | --- | --- | --- | --- | --- | --- | --- | --- | --- | --- | --- | --- | --- | --- | --- | --- | --- |
|  |

37.

| | | | **Your Input:** **>PF3D7_1206400** | | |  |  |  |  |  |  |  |  |  | | --- | --- | --- | --- | --- | --- | --- | --- | --- | --- | --- | --- | |  | [PFL0320w](http://string-db.org/newstring_cgi/display_single_node.pl?taskId=EbBFPpBRg8Xq&node=402049&targetmode=proteins) | hypothetical protein, conserved (346 aa) | |  |  | *(Plasmodium falciparum)* | | **Predicted Functional Partners:** | | | |  | [PF13_0027](http://string-db.org/newstring_cgi/display_single_node.pl?taskId=EbBFPpBRg8Xq&node=398952&targetmode=proteins) | hypothetical protein, conserved (771 aa) |  |  |  |  |  |  | [**•**](http://string-db.org/newstring_cgi/show_textmining_evidence.pl?taskId=EbBFPpBRg8Xq&node2=398952) |  | 0.786 | |  | [PTPS](http://string-db.org/newstring_cgi/display_single_node.pl?taskId=EbBFPpBRg8Xq&node=401559&targetmode=proteins) | 6-pyruvoyl tetrahydropterin synthase, putative (173 aa) |  |  |  |  |  |  | [**•**](http://string-db.org/newstring_cgi/show_textmining_evidence.pl?taskId=EbBFPpBRg8Xq&node2=401559) |  | 0.651 | |  | [PF14_0525](http://string-db.org/newstring_cgi/display_single_node.pl?taskId=EbBFPpBRg8Xq&node=399804&targetmode=proteins) | hypothetical protein (89 aa) |  |  |  |  |  |  | [**•**](http://string-db.org/newstring_cgi/show_textmining_evidence.pl?taskId=EbBFPpBRg8Xq&node2=399804) |  | 0.579 | |  | [PF11_0281](http://string-db.org/newstring_cgi/display_single_node.pl?taskId=EbBFPpBRg8Xq&node=398671&targetmode=proteins) | hypothetical protein (247 aa) |  |  |  |  |  |  | [**•**](http://string-db.org/newstring_cgi/show_textmining_evidence.pl?taskId=EbBFPpBRg8Xq&node2=398671) |  | 0.579 | |  | [MAL13P1.275-1](http://string-db.org/newstring_cgi/display_single_node.pl?taskId=EbBFPpBRg8Xq&node=397149&targetmode=proteins) | NLI interacting factor-like phosphatase, putative (1288 aa) |  |  |  |  |  |  | [**•**](http://string-db.org/newstring_cgi/show_textmining_evidence.pl?taskId=EbBFPpBRg8Xq&node2=397149) |  | 0.579 | |  | [MAL13P1.168-1](http://string-db.org/newstring_cgi/display_single_node.pl?taskId=EbBFPpBRg8Xq&node=397036&targetmode=proteins) | hypothetical protein, conserved (267 aa) |  |  |  |  |  |  | [**•**](http://string-db.org/newstring_cgi/show_textmining_evidence.pl?taskId=EbBFPpBRg8Xq&node2=397036) |  | 0.579 | |  | [PFL0325w](http://string-db.org/newstring_cgi/display_single_node.pl?taskId=EbBFPpBRg8Xq&node=402050&targetmode=proteins) | hypothetical protein, conserved (651 aa) |  | [**•**](http://string-db.org/newstring_cgi/show_fusion_evidence.pl?taskId=EbBFPpBRg8Xq&node2=402050) |  |  |  |  |  |  | 0.578 | |  | [PF14_0660](http://string-db.org/newstring_cgi/display_single_node.pl?taskId=EbBFPpBRg8Xq&node=399944&targetmode=proteins) | hypothetical protein (358 aa) |  |  |  | [**•**](http://string-db.org/newstring_cgi/show_coexpression_evidence.pl?taskId=EbBFPpBRg8Xq&node2=399944) |  |  | [**•**](http://string-db.org/newstring_cgi/show_textmining_evidence.pl?taskId=EbBFPpBRg8Xq&node2=399944) |  | 0.518 | |  | [PFL0300c](http://string-db.org/newstring_cgi/display_single_node.pl?taskId=EbBFPpBRg8Xq&node=402045&targetmode=proteins) | phosphoesterase, putative (304 aa) |  |  |  | [**•**](http://string-db.org/newstring_cgi/show_coexpression_evidence.pl?taskId=EbBFPpBRg8Xq&node2=402045) |  |  | [**•**](http://string-db.org/newstring_cgi/show_textmining_evidence.pl?taskId=EbBFPpBRg8Xq&node2=402045) |  | 0.517 | |  | [MAL8P1.109](http://string-db.org/newstring_cgi/display_single_node.pl?taskId=EbBFPpBRg8Xq&node=397534&targetmode=proteins) | Protein phosphatase 2C, putative (545 aa) |  |  |  | [**•**](http://string-db.org/newstring_cgi/show_coexpression_evidence.pl?taskId=EbBFPpBRg8Xq&node2=397534) |  |  | [**•**](http://string-db.org/newstring_cgi/show_textmining_evidence.pl?taskId=EbBFPpBRg8Xq&node2=397534) |  | 0.502 | | | --- | --- | --- | --- | --- | --- | --- | --- | --- | --- | --- | --- | --- | --- | --- | --- | --- | --- | --- | --- | --- | --- | --- | --- | --- | --- | --- | --- | --- | --- | --- | --- | --- | --- | --- | --- | --- | --- | --- | --- | --- | --- | --- | --- | --- | --- | --- | --- | --- | --- | --- | --- | --- | --- | --- | --- | --- | --- | --- | --- | --- | --- | --- | --- | --- | --- | --- | --- | --- | --- | --- | --- | --- | --- | --- | --- | --- | --- | --- | --- | --- | --- | --- | --- | --- | --- | --- | --- | --- | --- | --- | --- | --- | --- | --- | --- | --- | --- | --- | --- | --- | --- | --- | --- | --- | --- | --- | --- | --- | --- | --- | --- | --- | --- | --- | --- | --- | --- | --- | --- | --- | --- | --- | --- | --- | --- | --- | --- | --- | --- | --- | --- | --- | --- | --- | --- | --- | --- | --- | --- | --- | --- | | | --- | --- | --- | --- | --- | --- | --- | --- | --- | --- | --- | --- | --- | --- | --- | --- | --- | --- | --- | --- | --- | --- | --- | --- | --- | --- | --- | --- | --- | --- | --- | --- | --- | --- | --- | --- | --- | --- | --- | --- | --- | --- | --- | --- | --- | --- | --- | --- | --- | --- | --- | --- | --- | --- | --- | --- | --- | --- | --- | --- | --- | --- | --- | --- | --- | --- | --- | --- | --- | --- | --- | --- | --- | --- | --- | --- | --- | --- | --- | --- | --- | --- | --- | --- | --- | --- | --- | --- | --- | --- | --- | --- | --- | --- | --- | --- | --- | --- | --- | --- | --- | --- | --- | --- | --- | --- | --- | --- | --- | --- | --- | --- | --- | --- | --- | --- | --- | --- | --- | --- | --- | --- | --- | --- | --- | --- | --- | --- | --- | --- | --- | --- | --- | --- | --- | --- | --- | --- | --- | --- | --- | --- | --- | |
| --- | --- | --- | --- | --- | --- | --- | --- | --- | --- | --- | --- | --- | --- | --- | --- | --- | --- | --- | --- | --- | --- | --- | --- | --- | --- | --- | --- | --- | --- | --- | --- | --- | --- | --- | --- | --- | --- | --- | --- | --- | --- | --- | --- | --- | --- | --- | --- | --- | --- | --- | --- | --- | --- | --- | --- | --- | --- | --- | --- | --- | --- | --- | --- | --- | --- | --- | --- | --- | --- | --- | --- | --- | --- | --- | --- | --- | --- | --- | --- | --- | --- | --- | --- | --- | --- | --- | --- | --- | --- | --- | --- | --- | --- | --- | --- | --- | --- | --- | --- | --- | --- | --- | --- | --- | --- | --- | --- | --- | --- | --- | --- | --- | --- | --- | --- | --- | --- | --- | --- | --- | --- | --- | --- | --- | --- | --- | --- | --- | --- | --- | --- | --- | --- | --- | --- | --- | --- | --- | --- | --- | --- | --- | --- |
| |  | | --- | |

38.

| | | | **Your Input:** **>PF3D7_1208900** | | |  |  |  |  |  |  |  |  |  | | --- | --- | --- | --- | --- | --- | --- | --- | --- | --- | --- | --- | |  | [PFL0445w](http://string-db.org/newstring_cgi/display_single_node.pl?taskId=OSH6KrNzF_nR&node=402074&targetmode=proteins) | hypothetical protein, conserved (1442 aa) | |  |  | *(Plasmodium falciparum)* | | **Predicted Functional Partners:** | | | |  | [PFE0090w](http://string-db.org/newstring_cgi/display_single_node.pl?taskId=OSH6KrNzF_nR&node=400976&targetmode=proteins) | hypothetical protein, conserved (1076 aa) |  |  |  | [**•**](http://string-db.org/newstring_cgi/show_coexpression_evidence.pl?taskId=OSH6KrNzF_nR&node2=400976) |  |  |  |  | 0.575 | |  | [MAL8P1.101](http://string-db.org/newstring_cgi/display_single_node.pl?taskId=OSH6KrNzF_nR&node=397526&targetmode=proteins) | hypothetical protein, conserved (1103 aa) |  |  |  | [**•**](http://string-db.org/newstring_cgi/show_coexpression_evidence.pl?taskId=OSH6KrNzF_nR&node2=397526) |  |  |  |  | 0.508 | |  | [PFE0500c](http://string-db.org/newstring_cgi/display_single_node.pl?taskId=OSH6KrNzF_nR&node=401059&targetmode=proteins) | hypothetical protein, conserved (2535 aa) |  |  |  | [**•**](http://string-db.org/newstring_cgi/show_coexpression_evidence.pl?taskId=OSH6KrNzF_nR&node2=401059) |  |  |  | **•** | 0.433 | |  | [PFD0595w](http://string-db.org/newstring_cgi/display_single_node.pl?taskId=OSH6KrNzF_nR&node=400819&targetmode=proteins) | hypothetical protein, conserved (778 aa) |  |  |  | [**•**](http://string-db.org/newstring_cgi/show_coexpression_evidence.pl?taskId=OSH6KrNzF_nR&node2=400819) |  |  |  |  | 0.428 | | | --- | --- | --- | --- | --- | --- | --- | --- | --- | --- | --- | --- | --- | --- | --- | --- | --- | --- | --- | --- | --- | --- | --- | --- | --- | --- | --- | --- | --- | --- | --- | --- | --- | --- | --- | --- | --- | --- | --- | --- | --- | --- | --- | --- | --- | --- | --- | --- | --- | --- | --- | --- | --- | --- | --- | --- | --- | --- | --- | --- | --- | --- | --- | --- | --- | --- | --- | --- | --- | --- | | | --- | --- | --- | --- | --- | --- | --- | --- | --- | --- | --- | --- | --- | --- | --- | --- | --- | --- | --- | --- | --- | --- | --- | --- | --- | --- | --- | --- | --- | --- | --- | --- | --- | --- | --- | --- | --- | --- | --- | --- | --- | --- | --- | --- | --- | --- | --- | --- | --- | --- | --- | --- | --- | --- | --- | --- | --- | --- | --- | --- | --- | --- | --- | --- | --- | --- | --- | --- | --- | --- | --- | |
| --- | --- | --- | --- | --- | --- | --- | --- | --- | --- | --- | --- | --- | --- | --- | --- | --- | --- | --- | --- | --- | --- | --- | --- | --- | --- | --- | --- | --- | --- | --- | --- | --- | --- | --- | --- | --- | --- | --- | --- | --- | --- | --- | --- | --- | --- | --- | --- | --- | --- | --- | --- | --- | --- | --- | --- | --- | --- | --- | --- | --- | --- | --- | --- | --- | --- | --- | --- | --- | --- | --- | --- |
| |  | | --- | |

39.

| | | | **Your Input:** **>PF3D7_1226100** | | |  |  |  |  |  |  |  |  |  | | --- | --- | --- | --- | --- | --- | --- | --- | --- | --- | --- | --- | |  | [PFL1260w](http://string-db.org/newstring_cgi/display_single_node.pl?taskId=GhoF1Dt5tfqb&node=402242&targetmode=proteins) | hydrolase %2F phosphatase, putative (316 aa) | |  |  | *(Plasmodium falciparum)* | | **Predicted Functional Partners:** | | | |  | [PFE0150c](http://string-db.org/newstring_cgi/display_single_node.pl?taskId=GhoF1Dt5tfqb&node=400988&targetmode=proteins) | 4-diphosphocytidyl-2c-methyl-D-erythritol kinase (CMK), putative (537 aa) |  |  |  |  |  |  | [**•**](http://string-db.org/newstring_cgi/show_textmining_evidence.pl?taskId=GhoF1Dt5tfqb&node2=400988) |  | 0.893 | |  | [PF11_0479](http://string-db.org/newstring_cgi/display_single_node.pl?taskId=GhoF1Dt5tfqb&node=398870&targetmode=proteins) | hypothetical protein (3207 aa) |  |  |  |  |  |  | [**•**](http://string-db.org/newstring_cgi/show_textmining_evidence.pl?taskId=GhoF1Dt5tfqb&node2=398870) |  | 0.893 | |  | [PF13_0142](http://string-db.org/newstring_cgi/display_single_node.pl?taskId=GhoF1Dt5tfqb&node=399058&targetmode=proteins) | u6 snRNA-associated sm-like protein, putative (77 aa) |  |  |  |  |  |  | [**•**](http://string-db.org/newstring_cgi/show_textmining_evidence.pl?taskId=GhoF1Dt5tfqb&node2=399058) |  | 0.794 | |  | [SERA-2](http://string-db.org/newstring_cgi/display_single_node.pl?taskId=GhoF1Dt5tfqb&node=400300&targetmode=proteins) | cysteine protease, putative (1096 aa) |  |  |  |  |  |  | [**•**](http://string-db.org/newstring_cgi/show_textmining_evidence.pl?taskId=GhoF1Dt5tfqb&node2=400300) |  | 0.774 | |  | [MAL8P1.4](http://string-db.org/newstring_cgi/display_single_node.pl?taskId=GhoF1Dt5tfqb&node=397639&targetmode=proteins) | hypothetical protein, conserved (456 aa) |  |  |  |  |  |  | [**•**](http://string-db.org/newstring_cgi/show_textmining_evidence.pl?taskId=GhoF1Dt5tfqb&node2=397639) |  | 0.738 | |  | [PFD1050w](http://string-db.org/newstring_cgi/display_single_node.pl?taskId=GhoF1Dt5tfqb&node=400919&targetmode=proteins) | alpha-tubulin ii; Tubulin is the major constituent of microtubules. It binds two moles of GTP, [...] (450 aa) |  |  |  |  |  |  | [**•**](http://string-db.org/newstring_cgi/show_textmining_evidence.pl?taskId=GhoF1Dt5tfqb&node2=400919) |  | 0.690 | |  | [PF14_0155](http://string-db.org/newstring_cgi/display_single_node.pl?taskId=GhoF1Dt5tfqb&node=399425&targetmode=proteins) | serine C-palmitoyltransferase, putative (572 aa) |  |  |  | [**•**](http://string-db.org/newstring_cgi/show_coexpression_evidence.pl?taskId=GhoF1Dt5tfqb&node2=399425) |  |  | [**•**](http://string-db.org/newstring_cgi/show_textmining_evidence.pl?taskId=GhoF1Dt5tfqb&node2=399425) |  | 0.687 | |  | [PFI0980w](http://string-db.org/newstring_cgi/display_single_node.pl?taskId=GhoF1Dt5tfqb&node=401808&targetmode=proteins) | hypothetical membrane protein, conserved (612 aa) |  |  |  | [**•**](http://string-db.org/newstring_cgi/show_coexpression_evidence.pl?taskId=GhoF1Dt5tfqb&node2=401808) |  |  | [**•**](http://string-db.org/newstring_cgi/show_textmining_evidence.pl?taskId=GhoF1Dt5tfqb&node2=401808) |  | 0.682 | |  | [PFE0405c](http://string-db.org/newstring_cgi/display_single_node.pl?taskId=GhoF1Dt5tfqb&node=401040&targetmode=proteins) | hypothetical protein, conserved (355 aa) |  |  |  | [**•**](http://string-db.org/newstring_cgi/show_coexpression_evidence.pl?taskId=GhoF1Dt5tfqb&node2=401040) |  |  | [**•**](http://string-db.org/newstring_cgi/show_textmining_evidence.pl?taskId=GhoF1Dt5tfqb&node2=401040) |  | 0.675 | |  | [DPM1](http://string-db.org/newstring_cgi/display_single_node.pl?taskId=GhoF1Dt5tfqb&node=398818&targetmode=proteins) | dolichyl-phosphate b-D-mannosyltransferase, putative (259 aa) |  |  |  |  |  |  | [**•**](http://string-db.org/newstring_cgi/show_textmining_evidence.pl?taskId=GhoF1Dt5tfqb&node2=398818) |  | 0.581 | | | --- | --- | --- | --- | --- | --- | --- | --- | --- | --- | --- | --- | --- | --- | --- | --- | --- | --- | --- | --- | --- | --- | --- | --- | --- | --- | --- | --- | --- | --- | --- | --- | --- | --- | --- | --- | --- | --- | --- | --- | --- | --- | --- | --- | --- | --- | --- | --- | --- | --- | --- | --- | --- | --- | --- | --- | --- | --- | --- | --- | --- | --- | --- | --- | --- | --- | --- | --- | --- | --- | --- | --- | --- | --- | --- | --- | --- | --- | --- | --- | --- | --- | --- | --- | --- | --- | --- | --- | --- | --- | --- | --- | --- | --- | --- | --- | --- | --- | --- | --- | --- | --- | --- | --- | --- | --- | --- | --- | --- | --- | --- | --- | --- | --- | --- | --- | --- | --- | --- | --- | --- | --- | --- | --- | --- | --- | --- | --- | --- | --- | --- | --- | --- | --- | --- | --- | --- | --- | --- | --- | --- | --- | | | --- | --- | --- | --- | --- | --- | --- | --- | --- | --- | --- | --- | --- | --- | --- | --- | --- | --- | --- | --- | --- | --- | --- | --- | --- | --- | --- | --- | --- | --- | --- | --- | --- | --- | --- | --- | --- | --- | --- | --- | --- | --- | --- | --- | --- | --- | --- | --- | --- | --- | --- | --- | --- | --- | --- | --- | --- | --- | --- | --- | --- | --- | --- | --- | --- | --- | --- | --- | --- | --- | --- | --- | --- | --- | --- | --- | --- | --- | --- | --- | --- | --- | --- | --- | --- | --- | --- | --- | --- | --- | --- | --- | --- | --- | --- | --- | --- | --- | --- | --- | --- | --- | --- | --- | --- | --- | --- | --- | --- | --- | --- | --- | --- | --- | --- | --- | --- | --- | --- | --- | --- | --- | --- | --- | --- | --- | --- | --- | --- | --- | --- | --- | --- | --- | --- | --- | --- | --- | --- | --- | --- | --- | --- | |
| --- | --- | --- | --- | --- | --- | --- | --- | --- | --- | --- | --- | --- | --- | --- | --- | --- | --- | --- | --- | --- | --- | --- | --- | --- | --- | --- | --- | --- | --- | --- | --- | --- | --- | --- | --- | --- | --- | --- | --- | --- | --- | --- | --- | --- | --- | --- | --- | --- | --- | --- | --- | --- | --- | --- | --- | --- | --- | --- | --- | --- | --- | --- | --- | --- | --- | --- | --- | --- | --- | --- | --- | --- | --- | --- | --- | --- | --- | --- | --- | --- | --- | --- | --- | --- | --- | --- | --- | --- | --- | --- | --- | --- | --- | --- | --- | --- | --- | --- | --- | --- | --- | --- | --- | --- | --- | --- | --- | --- | --- | --- | --- | --- | --- | --- | --- | --- | --- | --- | --- | --- | --- | --- | --- | --- | --- | --- | --- | --- | --- | --- | --- | --- | --- | --- | --- | --- | --- | --- | --- | --- | --- | --- | --- |
| |  | | --- | |

40.

| | | | **Your Input:** **>PF3D7_1238600** | | |  |  |  |  |  |  |  |  |  | | --- | --- | --- | --- | --- | --- | --- | --- | --- | --- | --- | --- | |  | [PFL1870c](http://string-db.org/newstring_cgi/display_single_node.pl?taskId=FMCcV4xQWgVP&node=402367&targetmode=proteins) | sphingomyelin phosphodiesterase (393 aa) | |  |  | *(Plasmodium falciparum)* | | **Predicted Functional Partners:** | | | |  | [PFE0785c](http://string-db.org/newstring_cgi/display_single_node.pl?taskId=FMCcV4xQWgVP&node=401115&targetmode=proteins) | drug%2Fmetabolite transporter (456 aa) |  |  |  | [**•**](http://string-db.org/newstring_cgi/show_coexpression_evidence.pl?taskId=FMCcV4xQWgVP&node2=401115) |  |  |  |  | 0.960 | |  | [PDEbeta](http://string-db.org/newstring_cgi/display_single_node.pl?taskId=FMCcV4xQWgVP&node=396982&targetmode=proteins) | cAMP-specific 3',5'-cyclic phosphodiesterase 4D, putative (1139 aa) |  |  |  | [**•**](http://string-db.org/newstring_cgi/show_coexpression_evidence.pl?taskId=FMCcV4xQWgVP&node2=396982) |  |  |  |  | 0.960 | |  | [ABRA](http://string-db.org/newstring_cgi/display_single_node.pl?taskId=FMCcV4xQWgVP&node=402267&targetmode=proteins) | Merozoite Surface Protein 9, MSP-9 (743 aa) |  |  |  | [**•**](http://string-db.org/newstring_cgi/show_coexpression_evidence.pl?taskId=FMCcV4xQWgVP&node2=402267) |  |  |  |  | 0.958 | |  | [PFI1560c](http://string-db.org/newstring_cgi/display_single_node.pl?taskId=FMCcV4xQWgVP&node=401927&targetmode=proteins) | hypothetical protein, conserved (1247 aa) |  |  |  | [**•**](http://string-db.org/newstring_cgi/show_coexpression_evidence.pl?taskId=FMCcV4xQWgVP&node2=401927) |  |  |  |  | 0.958 | |  | [PFD1110w](http://string-db.org/newstring_cgi/display_single_node.pl?taskId=FMCcV4xQWgVP&node=400931&targetmode=proteins) | hypothetical membrane protein, conserved (372 aa) |  |  |  | [**•**](http://string-db.org/newstring_cgi/show_coexpression_evidence.pl?taskId=FMCcV4xQWgVP&node2=400931) |  |  |  |  | 0.958 | |  | [PFD0940w](http://string-db.org/newstring_cgi/display_single_node.pl?taskId=FMCcV4xQWgVP&node=400891&targetmode=proteins) | hypothetical protein, conserved (2472 aa) |  |  |  | [**•**](http://string-db.org/newstring_cgi/show_coexpression_evidence.pl?taskId=FMCcV4xQWgVP&node2=400891) |  |  |  |  | 0.954 | |  | [PF14_0091](http://string-db.org/newstring_cgi/display_single_node.pl?taskId=FMCcV4xQWgVP&node=399360&targetmode=proteins) | hypothetical protein (132 aa) |  |  |  | [**•**](http://string-db.org/newstring_cgi/show_coexpression_evidence.pl?taskId=FMCcV4xQWgVP&node2=399360) |  |  |  |  | 0.954 | |  | [PFL2215w](http://string-db.org/newstring_cgi/display_single_node.pl?taskId=FMCcV4xQWgVP&node=402438&targetmode=proteins) | actin; Actins are highly conserved proteins that are involved in various types of cell motility [...] (376 aa) |  |  |  | [**•**](http://string-db.org/newstring_cgi/show_coexpression_evidence.pl?taskId=FMCcV4xQWgVP&node2=402438) |  |  |  |  | 0.952 | |  | [MAL8P1.153-1](http://string-db.org/newstring_cgi/display_single_node.pl?taskId=FMCcV4xQWgVP&node=397576&targetmode=proteins) | hypothetical protein, conserved (2577 aa) |  |  |  | [**•**](http://string-db.org/newstring_cgi/show_coexpression_evidence.pl?taskId=FMCcV4xQWgVP&node2=397576) |  |  |  |  | 0.948 | |  | [PFE1130w](http://string-db.org/newstring_cgi/display_single_node.pl?taskId=FMCcV4xQWgVP&node=401185&targetmode=proteins) | hypothetical protein, conserved (483 aa) |  |  |  | [**•**](http://string-db.org/newstring_cgi/show_coexpression_evidence.pl?taskId=FMCcV4xQWgVP&node2=401185) |  |  |  |  | 0.944 | |  | [RAP2](http://string-db.org/newstring_cgi/display_single_node.pl?taskId=FMCcV4xQWgVP&node=400974&targetmode=proteins) | rhoptry-associated protein 2, RAP2 (398 aa) |  |  |  | [**•**](http://string-db.org/newstring_cgi/show_coexpression_evidence.pl?taskId=FMCcV4xQWgVP&node2=400974) |  |  |  |  | 0.942 | |  | [RhopH2](http://string-db.org/newstring_cgi/display_single_node.pl?taskId=FMCcV4xQWgVP&node=401903&targetmode=proteins) | High molecular weight rhoptry protein-2 (1378 aa) |  |  |  | [**•**](http://string-db.org/newstring_cgi/show_coexpression_evidence.pl?taskId=FMCcV4xQWgVP&node2=401903) |  |  |  | **•** | 0.935 | |  | [MAL13P1.152-1](http://string-db.org/newstring_cgi/display_single_node.pl?taskId=FMCcV4xQWgVP&node=397019&targetmode=proteins) | hypothetical protein, conserved (861 aa) |  |  |  | [**•**](http://string-db.org/newstring_cgi/show_coexpression_evidence.pl?taskId=FMCcV4xQWgVP&node2=397019) |  |  |  |  | 0.921 | |  | [PPR](http://string-db.org/newstring_cgi/display_single_node.pl?taskId=FMCcV4xQWgVP&node=399330&targetmode=proteins) | hypothetical protein (608 aa) |  |  |  |  |  |  | [**•**](http://string-db.org/newstring_cgi/show_textmining_evidence.pl?taskId=FMCcV4xQWgVP&node2=399330) |  | 0.903 | |  | [RON4](http://string-db.org/newstring_cgi/display_single_node.pl?taskId=FMCcV4xQWgVP&node=398555&targetmode=proteins) | hypothetical protein (2966 aa) |  |  |  | [**•**](http://string-db.org/newstring_cgi/show_coexpression_evidence.pl?taskId=FMCcV4xQWgVP&node2=398555) |  |  |  |  | 0.893 | |  | [MAL7P1.17](http://string-db.org/newstring_cgi/display_single_node.pl?taskId=FMCcV4xQWgVP&node=397405&targetmode=proteins) | hypothetical membrane protein, conserved (3559 aa) |  |  |  | [**•**](http://string-db.org/newstring_cgi/show_coexpression_evidence.pl?taskId=FMCcV4xQWgVP&node2=397405) |  |  |  |  | 0.893 | |  | [PFL1160c](http://string-db.org/newstring_cgi/display_single_node.pl?taskId=FMCcV4xQWgVP&node=402222&targetmode=proteins) | hypothetical protein, conserved (192 aa) |  |  |  | [**•**](http://string-db.org/newstring_cgi/show_coexpression_evidence.pl?taskId=FMCcV4xQWgVP&node2=402222) |  |  |  |  | 0.887 | |  | [GAP50](http://string-db.org/newstring_cgi/display_single_node.pl?taskId=FMCcV4xQWgVP&node=401788&targetmode=proteins) | acid phosphatase, putative (396 aa) |  |  |  | [**•**](http://string-db.org/newstring_cgi/show_coexpression_evidence.pl?taskId=FMCcV4xQWgVP&node2=401788) |  |  |  |  | 0.883 | |  | [PF14_0681](http://string-db.org/newstring_cgi/display_single_node.pl?taskId=FMCcV4xQWgVP&node=399966&targetmode=proteins) | diacylglycerol kinase, putative (488 aa) |  |  |  | [**•**](http://string-db.org/newstring_cgi/show_coexpression_evidence.pl?taskId=FMCcV4xQWgVP&node2=399966) |  |  |  |  | 0.883 | |  | [MAL13P1.130-1](http://string-db.org/newstring_cgi/display_single_node.pl?taskId=FMCcV4xQWgVP&node=396996&targetmode=proteins) | hypothetical protein, conserved (303 aa) |  |  |  | [**•**](http://string-db.org/newstring_cgi/show_coexpression_evidence.pl?taskId=FMCcV4xQWgVP&node2=396996) |  |  |  |  | 0.873 | | | --- | --- | --- | --- | --- | --- | --- | --- | --- | --- | --- | --- | --- | --- | --- | --- | --- | --- | --- | --- | --- | --- | --- | --- | --- | --- | --- | --- | --- | --- | --- | --- | --- | --- | --- | --- | --- | --- | --- | --- | --- | --- | --- | --- | --- | --- | --- | --- | --- | --- | --- | --- | --- | --- | --- | --- | --- | --- | --- | --- | --- | --- | --- | --- | --- | --- | --- | --- | --- | --- | --- | --- | --- | --- | --- | --- | --- | --- | --- | --- | --- | --- | --- | --- | --- | --- | --- | --- | --- | --- | --- | --- | --- | --- | --- | --- | --- | --- | --- | --- | --- | --- | --- | --- | --- | --- | --- | --- | --- | --- | --- | --- | --- | --- | --- | --- | --- | --- | --- | --- | --- | --- | --- | --- | --- | --- | --- | --- | --- | --- | --- | --- | --- | --- | --- | --- | --- | --- | --- | --- | --- | --- | --- | --- | --- | --- | --- | --- | --- | --- | --- | --- | --- | --- | --- | --- | --- | --- | --- | --- | --- | --- | --- | --- | --- | --- | --- | --- | --- | --- | --- | --- | --- | --- | --- | --- | --- | --- | --- | --- | --- | --- | --- | --- | --- | --- | --- | --- | --- | --- | --- | --- | --- | --- | --- | --- | --- | --- | --- | --- | --- | --- | --- | --- | --- | --- | --- | --- | --- | --- | --- | --- | --- | --- | --- | --- | --- | --- | --- | --- | --- | --- | --- | --- | --- | --- | --- | --- | --- | --- | --- | --- | --- | --- | --- | --- | --- | --- | --- | --- | --- | --- | --- | --- | --- | --- | --- | --- | --- | --- | --- | --- | --- | --- | --- | --- | --- | --- | --- | --- | --- | --- | | | --- | --- | --- | --- | --- | --- | --- | --- | --- | --- | --- | --- | --- | --- | --- | --- | --- | --- | --- | --- | --- | --- | --- | --- | --- | --- | --- | --- | --- | --- | --- | --- | --- | --- | --- | --- | --- | --- | --- | --- | --- | --- | --- | --- | --- | --- | --- | --- | --- | --- | --- | --- | --- | --- | --- | --- | --- | --- | --- | --- | --- | --- | --- | --- | --- | --- | --- | --- | --- | --- | --- | --- | --- | --- | --- | --- | --- | --- | --- | --- | --- | --- | --- | --- | --- | --- | --- | --- | --- | --- | --- | --- | --- | --- | --- | --- | --- | --- | --- | --- | --- | --- | --- | --- | --- | --- | --- | --- | --- | --- | --- | --- | --- | --- | --- | --- | --- | --- | --- | --- | --- | --- | --- | --- | --- | --- | --- | --- | --- | --- | --- | --- | --- | --- | --- | --- | --- | --- | --- | --- | --- | --- | --- | --- | --- | --- | --- | --- | --- | --- | --- | --- | --- | --- | --- | --- | --- | --- | --- | --- | --- | --- | --- | --- | --- | --- | --- | --- | --- | --- | --- | --- | --- | --- | --- | --- | --- | --- | --- | --- | --- | --- | --- | --- | --- | --- | --- | --- | --- | --- | --- | --- | --- | --- | --- | --- | --- | --- | --- | --- | --- | --- | --- | --- | --- | --- | --- | --- | --- | --- | --- | --- | --- | --- | --- | --- | --- | --- | --- | --- | --- | --- | --- | --- | --- | --- | --- | --- | --- | --- | --- | --- | --- | --- | --- | --- | --- | --- | --- | --- | --- | --- | --- | --- | --- | --- | --- | --- | --- | --- | --- | --- | --- | --- | --- | --- | --- | --- | --- | --- | --- | --- | --- | |
| --- | --- | --- | --- | --- | --- | --- | --- | --- | --- | --- | --- | --- | --- | --- | --- | --- | --- | --- | --- | --- | --- | --- | --- | --- | --- | --- | --- | --- | --- | --- | --- | --- | --- | --- | --- | --- | --- | --- | --- | --- | --- | --- | --- | --- | --- | --- | --- | --- | --- | --- | --- | --- | --- | --- | --- | --- | --- | --- | --- | --- | --- | --- | --- | --- | --- | --- | --- | --- | --- | --- | --- | --- | --- | --- | --- | --- | --- | --- | --- | --- | --- | --- | --- | --- | --- | --- | --- | --- | --- | --- | --- | --- | --- | --- | --- | --- | --- | --- | --- | --- | --- | --- | --- | --- | --- | --- | --- | --- | --- | --- | --- | --- | --- | --- | --- | --- | --- | --- | --- | --- | --- | --- | --- | --- | --- | --- | --- | --- | --- | --- | --- | --- | --- | --- | --- | --- | --- | --- | --- | --- | --- | --- | --- | --- | --- | --- | --- | --- | --- | --- | --- | --- | --- | --- | --- | --- | --- | --- | --- | --- | --- | --- | --- | --- | --- | --- | --- | --- | --- | --- | --- | --- | --- | --- | --- | --- | --- | --- | --- | --- | --- | --- | --- | --- | --- | --- | --- | --- | --- | --- | --- | --- | --- | --- | --- | --- | --- | --- | --- | --- | --- | --- | --- | --- | --- | --- | --- | --- | --- | --- | --- | --- | --- | --- | --- | --- | --- | --- | --- | --- | --- | --- | --- | --- | --- | --- | --- | --- | --- | --- | --- | --- | --- | --- | --- | --- | --- | --- | --- | --- | --- | --- | --- | --- | --- | --- | --- | --- | --- | --- | --- | --- | --- | --- | --- | --- | --- | --- | --- | --- | --- | --- | --- |
|  |

| | |  | | --- | | | --- | --- | |
| --- | --- | --- |
| |  | | --- | |

41.

| | | | **Your Input:** **>PF3D7_1249300** | | |  |  |  |  |  |  |  |  |  | | --- | --- | --- | --- | --- | --- | --- | --- | --- | --- | --- | --- | |  | [PFL2365w](http://string-db.org/newstring_cgi/display_single_node.pl?taskId=vtBsRqVrPA4C&node=402468&targetmode=proteins) | hypothetical protein, conserved (1027 aa) | |  |  | *(Plasmodium falciparum)* | | **Predicted Functional Partners:** | | | |  | [PF10_0093](http://string-db.org/newstring_cgi/display_single_node.pl?taskId=vtBsRqVrPA4C&node=398077&targetmode=proteins) | hypothetical protein (345 aa) |  |  |  |  |  |  | [**•**](http://string-db.org/newstring_cgi/show_textmining_evidence.pl?taskId=vtBsRqVrPA4C&node2=398077) |  | 0.688 | |  | [PFL0320w](http://string-db.org/newstring_cgi/display_single_node.pl?taskId=vtBsRqVrPA4C&node=402049&targetmode=proteins) | hypothetical protein, conserved (346 aa) |  |  |  |  |  |  | [**•**](http://string-db.org/newstring_cgi/show_textmining_evidence.pl?taskId=vtBsRqVrPA4C&node2=402049) |  | 0.486 | |  | [PF13_0027](http://string-db.org/newstring_cgi/display_single_node.pl?taskId=vtBsRqVrPA4C&node=398952&targetmode=proteins) | hypothetical protein, conserved (771 aa) |  |  |  |  |  |  | [**•**](http://string-db.org/newstring_cgi/show_textmining_evidence.pl?taskId=vtBsRqVrPA4C&node2=398952) |  | 0.486 | |  | [MAL13P1.168-1](http://string-db.org/newstring_cgi/display_single_node.pl?taskId=vtBsRqVrPA4C&node=397036&targetmode=proteins) | hypothetical protein, conserved (267 aa) |  |  |  |  |  |  | [**•**](http://string-db.org/newstring_cgi/show_textmining_evidence.pl?taskId=vtBsRqVrPA4C&node2=397036) |  | 0.486 | |  | [PF14_0525](http://string-db.org/newstring_cgi/display_single_node.pl?taskId=vtBsRqVrPA4C&node=399804&targetmode=proteins) | hypothetical protein (89 aa) |  |  |  |  |  |  | [**•**](http://string-db.org/newstring_cgi/show_textmining_evidence.pl?taskId=vtBsRqVrPA4C&node2=399804) |  | 0.484 | |  | [PF11_0281](http://string-db.org/newstring_cgi/display_single_node.pl?taskId=vtBsRqVrPA4C&node=398671&targetmode=proteins) | hypothetical protein (247 aa) |  |  |  |  |  |  | [**•**](http://string-db.org/newstring_cgi/show_textmining_evidence.pl?taskId=vtBsRqVrPA4C&node2=398671) |  | 0.484 | |  | [MAL13P1.275-1](http://string-db.org/newstring_cgi/display_single_node.pl?taskId=vtBsRqVrPA4C&node=397149&targetmode=proteins) | NLI interacting factor-like phosphatase, putative (1288 aa) |  |  |  |  |  |  | [**•**](http://string-db.org/newstring_cgi/show_textmining_evidence.pl?taskId=vtBsRqVrPA4C&node2=397149) |  | 0.484 | |  | [PRL](http://string-db.org/newstring_cgi/display_single_node.pl?taskId=vtBsRqVrPA4C&node=398526&targetmode=proteins) | protein tyrosine phosphatase, putative (218 aa) |  |  |  |  |  |  | [**•**](http://string-db.org/newstring_cgi/show_textmining_evidence.pl?taskId=vtBsRqVrPA4C&node2=398526) |  | 0.466 | |  | [PF10_0124](http://string-db.org/newstring_cgi/display_single_node.pl?taskId=vtBsRqVrPA4C&node=398108&targetmode=proteins) | hypothetical protein (1438 aa) |  |  |  |  |  |  | [**•**](http://string-db.org/newstring_cgi/show_textmining_evidence.pl?taskId=vtBsRqVrPA4C&node2=398108) |  | 0.460 | |  | [PF07_0110](http://string-db.org/newstring_cgi/display_single_node.pl?taskId=vtBsRqVrPA4C&node=397814&targetmode=proteins) | hypothetical protein, conserved (519 aa) |  |  |  |  |  |  | [**•**](http://string-db.org/newstring_cgi/show_textmining_evidence.pl?taskId=vtBsRqVrPA4C&node2=397814) |  | 0.454 | | | --- | --- | --- | --- | --- | --- | --- | --- | --- | --- | --- | --- | --- | --- | --- | --- | --- | --- | --- | --- | --- | --- | --- | --- | --- | --- | --- | --- | --- | --- | --- | --- | --- | --- | --- | --- | --- | --- | --- | --- | --- | --- | --- | --- | --- | --- | --- | --- | --- | --- | --- | --- | --- | --- | --- | --- | --- | --- | --- | --- | --- | --- | --- | --- | --- | --- | --- | --- | --- | --- | --- | --- | --- | --- | --- | --- | --- | --- | --- | --- | --- | --- | --- | --- | --- | --- | --- | --- | --- | --- | --- | --- | --- | --- | --- | --- | --- | --- | --- | --- | --- | --- | --- | --- | --- | --- | --- | --- | --- | --- | --- | --- | --- | --- | --- | --- | --- | --- | --- | --- | --- | --- | --- | --- | --- | --- | --- | --- | --- | --- | --- | --- | --- | --- | --- | --- | --- | --- | --- | --- | --- | --- | | | --- | --- | --- | --- | --- | --- | --- | --- | --- | --- | --- | --- | --- | --- | --- | --- | --- | --- | --- | --- | --- | --- | --- | --- | --- | --- | --- | --- | --- | --- | --- | --- | --- | --- | --- | --- | --- | --- | --- | --- | --- | --- | --- | --- | --- | --- | --- | --- | --- | --- | --- | --- | --- | --- | --- | --- | --- | --- | --- | --- | --- | --- | --- | --- | --- | --- | --- | --- | --- | --- | --- | --- | --- | --- | --- | --- | --- | --- | --- | --- | --- | --- | --- | --- | --- | --- | --- | --- | --- | --- | --- | --- | --- | --- | --- | --- | --- | --- | --- | --- | --- | --- | --- | --- | --- | --- | --- | --- | --- | --- | --- | --- | --- | --- | --- | --- | --- | --- | --- | --- | --- | --- | --- | --- | --- | --- | --- | --- | --- | --- | --- | --- | --- | --- | --- | --- | --- | --- | --- | --- | --- | --- | --- | |
| --- | --- | --- | --- | --- | --- | --- | --- | --- | --- | --- | --- | --- | --- | --- | --- | --- | --- | --- | --- | --- | --- | --- | --- | --- | --- | --- | --- | --- | --- | --- | --- | --- | --- | --- | --- | --- | --- | --- | --- | --- | --- | --- | --- | --- | --- | --- | --- | --- | --- | --- | --- | --- | --- | --- | --- | --- | --- | --- | --- | --- | --- | --- | --- | --- | --- | --- | --- | --- | --- | --- | --- | --- | --- | --- | --- | --- | --- | --- | --- | --- | --- | --- | --- | --- | --- | --- | --- | --- | --- | --- | --- | --- | --- | --- | --- | --- | --- | --- | --- | --- | --- | --- | --- | --- | --- | --- | --- | --- | --- | --- | --- | --- | --- | --- | --- | --- | --- | --- | --- | --- | --- | --- | --- | --- | --- | --- | --- | --- | --- | --- | --- | --- | --- | --- | --- | --- | --- | --- | --- | --- | --- | --- | --- |
| |  | | --- | |

42.

| | | | **Your Input:** **>PF3D7_1305500** | | |  |  |  |  |  |  |  |  |  | | --- | --- | --- | --- | --- | --- | --- | --- | --- | --- | --- | --- | |  | [PF13_0027](http://string-db.org/newstring_cgi/display_single_node.pl?taskId=lJB0SP2xly2d&node=398952&targetmode=proteins) | hypothetical protein, conserved (771 aa) | |  |  | *(Plasmodium falciparum)* | | **Predicted Functional Partners:** | | | |  | [PFL0320w](http://string-db.org/newstring_cgi/display_single_node.pl?taskId=lJB0SP2xly2d&node=402049&targetmode=proteins) | hypothetical protein, conserved (346 aa) |  |  |  |  |  |  | [**•**](http://string-db.org/newstring_cgi/show_textmining_evidence.pl?taskId=lJB0SP2xly2d&node2=402049) |  | 0.786 | |  | [PTPS](http://string-db.org/newstring_cgi/display_single_node.pl?taskId=lJB0SP2xly2d&node=401559&targetmode=proteins) | 6-pyruvoyl tetrahydropterin synthase, putative (173 aa) |  |  |  |  |  |  | [**•**](http://string-db.org/newstring_cgi/show_textmining_evidence.pl?taskId=lJB0SP2xly2d&node2=401559) |  | 0.651 | |  | [PF14_0525](http://string-db.org/newstring_cgi/display_single_node.pl?taskId=lJB0SP2xly2d&node=399804&targetmode=proteins) | hypothetical protein (89 aa) |  |  |  |  |  |  | [**•**](http://string-db.org/newstring_cgi/show_textmining_evidence.pl?taskId=lJB0SP2xly2d&node2=399804) |  | 0.579 | |  | [PF11_0281](http://string-db.org/newstring_cgi/display_single_node.pl?taskId=lJB0SP2xly2d&node=398671&targetmode=proteins) | hypothetical protein (247 aa) |  |  |  |  |  |  | [**•**](http://string-db.org/newstring_cgi/show_textmining_evidence.pl?taskId=lJB0SP2xly2d&node2=398671) |  | 0.579 | |  | [MAL13P1.275-1](http://string-db.org/newstring_cgi/display_single_node.pl?taskId=lJB0SP2xly2d&node=397149&targetmode=proteins) | NLI interacting factor-like phosphatase, putative (1288 aa) |  |  |  |  |  |  | [**•**](http://string-db.org/newstring_cgi/show_textmining_evidence.pl?taskId=lJB0SP2xly2d&node2=397149) |  | 0.579 | |  | [MAL13P1.168-1](http://string-db.org/newstring_cgi/display_single_node.pl?taskId=lJB0SP2xly2d&node=397036&targetmode=proteins) | hypothetical protein, conserved (267 aa) |  |  |  |  |  |  | [**•**](http://string-db.org/newstring_cgi/show_textmining_evidence.pl?taskId=lJB0SP2xly2d&node2=397036) |  | 0.579 | |  | [PFE1010w](http://string-db.org/newstring_cgi/display_single_node.pl?taskId=lJB0SP2xly2d&node=401160&targetmode=proteins) | protein phosphatase 2c, putative (697 aa) |  |  |  | [**•**](http://string-db.org/newstring_cgi/show_coexpression_evidence.pl?taskId=lJB0SP2xly2d&node2=401160) |  |  | [**•**](http://string-db.org/newstring_cgi/show_textmining_evidence.pl?taskId=lJB0SP2xly2d&node2=401160) |  | 0.513 | |  | [PFL2365w](http://string-db.org/newstring_cgi/display_single_node.pl?taskId=lJB0SP2xly2d&node=402468&targetmode=proteins) | hypothetical protein, conserved (1027 aa) |  |  |  |  |  |  | [**•**](http://string-db.org/newstring_cgi/show_textmining_evidence.pl?taskId=lJB0SP2xly2d&node2=402468) |  | 0.486 | |  | [PF14_0523](http://string-db.org/newstring_cgi/display_single_node.pl?taskId=lJB0SP2xly2d&node=399802&targetmode=proteins) | protein phosphatase 2C, putative (289 aa) |  |  |  |  |  |  | [**•**](http://string-db.org/newstring_cgi/show_textmining_evidence.pl?taskId=lJB0SP2xly2d&node2=399802) |  | 0.486 | |  | [PRL](http://string-db.org/newstring_cgi/display_single_node.pl?taskId=lJB0SP2xly2d&node=398526&targetmode=proteins) | protein tyrosine phosphatase, putative (218 aa) |  |  |  |  |  |  | [**•**](http://string-db.org/newstring_cgi/show_textmining_evidence.pl?taskId=lJB0SP2xly2d&node2=398526) |  | 0.486 | | | --- | --- | --- | --- | --- | --- | --- | --- | --- | --- | --- | --- | --- | --- | --- | --- | --- | --- | --- | --- | --- | --- | --- | --- | --- | --- | --- | --- | --- | --- | --- | --- | --- | --- | --- | --- | --- | --- | --- | --- | --- | --- | --- | --- | --- | --- | --- | --- | --- | --- | --- | --- | --- | --- | --- | --- | --- | --- | --- | --- | --- | --- | --- | --- | --- | --- | --- | --- | --- | --- | --- | --- | --- | --- | --- | --- | --- | --- | --- | --- | --- | --- | --- | --- | --- | --- | --- | --- | --- | --- | --- | --- | --- | --- | --- | --- | --- | --- | --- | --- | --- | --- | --- | --- | --- | --- | --- | --- | --- | --- | --- | --- | --- | --- | --- | --- | --- | --- | --- | --- | --- | --- | --- | --- | --- | --- | --- | --- | --- | --- | --- | --- | --- | --- | --- | --- | --- | --- | --- | --- | --- | --- | | | --- | --- | --- | --- | --- | --- | --- | --- | --- | --- | --- | --- | --- | --- | --- | --- | --- | --- | --- | --- | --- | --- | --- | --- | --- | --- | --- | --- | --- | --- | --- | --- | --- | --- | --- | --- | --- | --- | --- | --- | --- | --- | --- | --- | --- | --- | --- | --- | --- | --- | --- | --- | --- | --- | --- | --- | --- | --- | --- | --- | --- | --- | --- | --- | --- | --- | --- | --- | --- | --- | --- | --- | --- | --- | --- | --- | --- | --- | --- | --- | --- | --- | --- | --- | --- | --- | --- | --- | --- | --- | --- | --- | --- | --- | --- | --- | --- | --- | --- | --- | --- | --- | --- | --- | --- | --- | --- | --- | --- | --- | --- | --- | --- | --- | --- | --- | --- | --- | --- | --- | --- | --- | --- | --- | --- | --- | --- | --- | --- | --- | --- | --- | --- | --- | --- | --- | --- | --- | --- | --- | --- | --- | --- | |
| --- | --- | --- | --- | --- | --- | --- | --- | --- | --- | --- | --- | --- | --- | --- | --- | --- | --- | --- | --- | --- | --- | --- | --- | --- | --- | --- | --- | --- | --- | --- | --- | --- | --- | --- | --- | --- | --- | --- | --- | --- | --- | --- | --- | --- | --- | --- | --- | --- | --- | --- | --- | --- | --- | --- | --- | --- | --- | --- | --- | --- | --- | --- | --- | --- | --- | --- | --- | --- | --- | --- | --- | --- | --- | --- | --- | --- | --- | --- | --- | --- | --- | --- | --- | --- | --- | --- | --- | --- | --- | --- | --- | --- | --- | --- | --- | --- | --- | --- | --- | --- | --- | --- | --- | --- | --- | --- | --- | --- | --- | --- | --- | --- | --- | --- | --- | --- | --- | --- | --- | --- | --- | --- | --- | --- | --- | --- | --- | --- | --- | --- | --- | --- | --- | --- | --- | --- | --- | --- | --- | --- | --- | --- | --- |
| |  | | --- | |

43.

| | | | **Your Input:** **>PF3D7_1309200** | | |  |  |  |  |  |  |  |  |  | | --- | --- | --- | --- | --- | --- | --- | --- | --- | --- | --- | --- | |  | [MAL13P1.44](http://string-db.org/newstring_cgi/display_single_node.pl?taskId=NcA9XBN2mSqE&node=397260&targetmode=proteins) | protein phosphatase 2c-like protein, putative (827 aa) | |  |  | *(Plasmodium falciparum)* | | **Predicted Functional Partners:** | | | |  | [PF11_0362](http://string-db.org/newstring_cgi/display_single_node.pl?taskId=NcA9XBN2mSqE&node=398753&targetmode=proteins) | protein phosphatase, putative (689 aa) |  |  |  | [**•**](http://string-db.org/newstring_cgi/show_coexpression_evidence.pl?taskId=NcA9XBN2mSqE&node2=398753) |  |  | [**•**](http://string-db.org/newstring_cgi/show_textmining_evidence.pl?taskId=NcA9XBN2mSqE&node2=398753) |  | 0.715 | |  | [PFE0795c](http://string-db.org/newstring_cgi/display_single_node.pl?taskId=NcA9XBN2mSqE&node=401117&targetmode=proteins) | nif-like protein, putative (328 aa) |  |  |  |  |  |  | [**•**](http://string-db.org/newstring_cgi/show_textmining_evidence.pl?taskId=NcA9XBN2mSqE&node2=401117) |  | 0.688 | |  | [PF14_0523](http://string-db.org/newstring_cgi/display_single_node.pl?taskId=NcA9XBN2mSqE&node=399802&targetmode=proteins) | protein phosphatase 2C, putative (289 aa) |  |  |  |  |  |  | [**•**](http://string-db.org/newstring_cgi/show_textmining_evidence.pl?taskId=NcA9XBN2mSqE&node2=399802) |  | 0.688 | |  | [PF10_0124](http://string-db.org/newstring_cgi/display_single_node.pl?taskId=NcA9XBN2mSqE&node=398108&targetmode=proteins) | hypothetical protein (1438 aa) |  |  |  |  |  |  | [**•**](http://string-db.org/newstring_cgi/show_textmining_evidence.pl?taskId=NcA9XBN2mSqE&node2=398108) |  | 0.688 | |  | [PF10_0093](http://string-db.org/newstring_cgi/display_single_node.pl?taskId=NcA9XBN2mSqE&node=398077&targetmode=proteins) | hypothetical protein (345 aa) |  |  |  |  |  |  | [**•**](http://string-db.org/newstring_cgi/show_textmining_evidence.pl?taskId=NcA9XBN2mSqE&node2=398077) |  | 0.688 | |  | [PF07_0110](http://string-db.org/newstring_cgi/display_single_node.pl?taskId=NcA9XBN2mSqE&node=397814&targetmode=proteins) | hypothetical protein, conserved (519 aa) |  |  |  |  |  |  | [**•**](http://string-db.org/newstring_cgi/show_textmining_evidence.pl?taskId=NcA9XBN2mSqE&node2=397814) |  | 0.688 | |  | [MAL13P1.174-1](http://string-db.org/newstring_cgi/display_single_node.pl?taskId=NcA9XBN2mSqE&node=397043&targetmode=proteins) | MSP7-like protein (281 aa) |  |  |  |  |  |  | [**•**](http://string-db.org/newstring_cgi/show_textmining_evidence.pl?taskId=NcA9XBN2mSqE&node2=397043) |  | 0.687 | |  | [PFI1245c](http://string-db.org/newstring_cgi/display_single_node.pl?taskId=NcA9XBN2mSqE&node=401862&targetmode=proteins) | Protein phosphatase-beta (466 aa) |  |  |  |  |  |  | [**•**](http://string-db.org/newstring_cgi/show_textmining_evidence.pl?taskId=NcA9XBN2mSqE&node2=401862) |  | 0.649 | |  | [PfPP5](http://string-db.org/newstring_cgi/display_single_node.pl?taskId=NcA9XBN2mSqE&node=397148&targetmode=proteins) | serine%2Fthreonine protein phosphatase pfPp5 (658 aa) |  |  |  |  |  |  | [**•**](http://string-db.org/newstring_cgi/show_textmining_evidence.pl?taskId=NcA9XBN2mSqE&node2=397148) |  | 0.587 | |  | [PFL0320w](http://string-db.org/newstring_cgi/display_single_node.pl?taskId=NcA9XBN2mSqE&node=402049&targetmode=proteins) | hypothetical protein, conserved (346 aa) |  |  |  |  |  |  | [**•**](http://string-db.org/newstring_cgi/show_textmining_evidence.pl?taskId=NcA9XBN2mSqE&node2=402049) |  | 0.480 | | | --- | --- | --- | --- | --- | --- | --- | --- | --- | --- | --- | --- | --- | --- | --- | --- | --- | --- | --- | --- | --- | --- | --- | --- | --- | --- | --- | --- | --- | --- | --- | --- | --- | --- | --- | --- | --- | --- | --- | --- | --- | --- | --- | --- | --- | --- | --- | --- | --- | --- | --- | --- | --- | --- | --- | --- | --- | --- | --- | --- | --- | --- | --- | --- | --- | --- | --- | --- | --- | --- | --- | --- | --- | --- | --- | --- | --- | --- | --- | --- | --- | --- | --- | --- | --- | --- | --- | --- | --- | --- | --- | --- | --- | --- | --- | --- | --- | --- | --- | --- | --- | --- | --- | --- | --- | --- | --- | --- | --- | --- | --- | --- | --- | --- | --- | --- | --- | --- | --- | --- | --- | --- | --- | --- | --- | --- | --- | --- | --- | --- | --- | --- | --- | --- | --- | --- | --- | --- | --- | --- | --- | --- | | | --- | --- | --- | --- | --- | --- | --- | --- | --- | --- | --- | --- | --- | --- | --- | --- | --- | --- | --- | --- | --- | --- | --- | --- | --- | --- | --- | --- | --- | --- | --- | --- | --- | --- | --- | --- | --- | --- | --- | --- | --- | --- | --- | --- | --- | --- | --- | --- | --- | --- | --- | --- | --- | --- | --- | --- | --- | --- | --- | --- | --- | --- | --- | --- | --- | --- | --- | --- | --- | --- | --- | --- | --- | --- | --- | --- | --- | --- | --- | --- | --- | --- | --- | --- | --- | --- | --- | --- | --- | --- | --- | --- | --- | --- | --- | --- | --- | --- | --- | --- | --- | --- | --- | --- | --- | --- | --- | --- | --- | --- | --- | --- | --- | --- | --- | --- | --- | --- | --- | --- | --- | --- | --- | --- | --- | --- | --- | --- | --- | --- | --- | --- | --- | --- | --- | --- | --- | --- | --- | --- | --- | --- | --- | |
| --- | --- | --- | --- | --- | --- | --- | --- | --- | --- | --- | --- | --- | --- | --- | --- | --- | --- | --- | --- | --- | --- | --- | --- | --- | --- | --- | --- | --- | --- | --- | --- | --- | --- | --- | --- | --- | --- | --- | --- | --- | --- | --- | --- | --- | --- | --- | --- | --- | --- | --- | --- | --- | --- | --- | --- | --- | --- | --- | --- | --- | --- | --- | --- | --- | --- | --- | --- | --- | --- | --- | --- | --- | --- | --- | --- | --- | --- | --- | --- | --- | --- | --- | --- | --- | --- | --- | --- | --- | --- | --- | --- | --- | --- | --- | --- | --- | --- | --- | --- | --- | --- | --- | --- | --- | --- | --- | --- | --- | --- | --- | --- | --- | --- | --- | --- | --- | --- | --- | --- | --- | --- | --- | --- | --- | --- | --- | --- | --- | --- | --- | --- | --- | --- | --- | --- | --- | --- | --- | --- | --- | --- | --- | --- |
| |  | | --- | |

44.

| | | | **Your Input:** **>PF3D7_1322000** | | |  |  |  |  |  |  |  |  |  | | --- | --- | --- | --- | --- | --- | --- | --- | --- | --- | --- | --- | |  | [MAL13P1.121-1](http://string-db.org/newstring_cgi/display_single_node.pl?taskId=2YfL_XhmYlxQ&node=396986&targetmode=proteins) | hypothetical protein, conserved (565 aa) | |  |  | *(Plasmodium falciparum)* | | **Predicted Functional Partners:** | | | |  | [PFE0450w](http://string-db.org/newstring_cgi/display_single_node.pl?taskId=2YfL_XhmYlxQ&node=401049&targetmode=proteins) | chromosome condensation protein, putative (1708 aa) |  |  |  | [**•**](http://string-db.org/newstring_cgi/show_coexpression_evidence.pl?taskId=2YfL_XhmYlxQ&node2=401049) |  |  |  |  | 0.855 | |  | [PF10_0020](http://string-db.org/newstring_cgi/display_single_node.pl?taskId=2YfL_XhmYlxQ&node=397998&targetmode=proteins) | hypothetical protein (763 aa) |  |  |  | [**•**](http://string-db.org/newstring_cgi/show_coexpression_evidence.pl?taskId=2YfL_XhmYlxQ&node2=397998) |  |  |  |  | 0.837 | |  | [PF07_0012](http://string-db.org/newstring_cgi/display_single_node.pl?taskId=2YfL_XhmYlxQ&node=397714&targetmode=proteins) | hypothetical protein, conserved (1540 aa) |  |  |  | [**•**](http://string-db.org/newstring_cgi/show_coexpression_evidence.pl?taskId=2YfL_XhmYlxQ&node2=397714) |  |  |  |  | 0.819 | |  | [R17](http://string-db.org/newstring_cgi/display_single_node.pl?taskId=2YfL_XhmYlxQ&node=396970&targetmode=proteins) | hypothetical protein, conserved (1070 aa) |  |  |  | [**•**](http://string-db.org/newstring_cgi/show_coexpression_evidence.pl?taskId=2YfL_XhmYlxQ&node2=396970) |  |  |  |  | 0.813 | |  | [PFE0090w](http://string-db.org/newstring_cgi/display_single_node.pl?taskId=2YfL_XhmYlxQ&node=400976&targetmode=proteins) | hypothetical protein, conserved (1076 aa) |  |  |  | [**•**](http://string-db.org/newstring_cgi/show_coexpression_evidence.pl?taskId=2YfL_XhmYlxQ&node2=400976) |  |  |  |  | 0.780 | |  | [MAL13P1.19](http://string-db.org/newstring_cgi/display_single_node.pl?taskId=2YfL_XhmYlxQ&node=397059&targetmode=proteins) | peptidase, putative (9271 aa) |  |  |  | [**•**](http://string-db.org/newstring_cgi/show_coexpression_evidence.pl?taskId=2YfL_XhmYlxQ&node2=397059) |  |  |  |  | 0.770 | |  | [PF11_0342](http://string-db.org/newstring_cgi/display_single_node.pl?taskId=2YfL_XhmYlxQ&node=398733&targetmode=proteins) | hypothetical protein (2072 aa) |  |  |  | [**•**](http://string-db.org/newstring_cgi/show_coexpression_evidence.pl?taskId=2YfL_XhmYlxQ&node2=398733) |  |  |  |  | 0.752 | |  | [PFB0530c](http://string-db.org/newstring_cgi/display_single_node.pl?taskId=2YfL_XhmYlxQ&node=400338&targetmode=proteins) | hypothetical protein (430 aa) |  |  |  | [**•**](http://string-db.org/newstring_cgi/show_coexpression_evidence.pl?taskId=2YfL_XhmYlxQ&node2=400338) |  |  |  |  | 0.744 | |  | [Pf92](http://string-db.org/newstring_cgi/display_single_node.pl?taskId=2YfL_XhmYlxQ&node=399245&targetmode=proteins) | cysteine-rich surface protein (796 aa) |  |  |  | [**•**](http://string-db.org/newstring_cgi/show_coexpression_evidence.pl?taskId=2YfL_XhmYlxQ&node2=399245) |  |  |  |  | 0.740 | |  | [MAL7P1.167](http://string-db.org/newstring_cgi/display_single_node.pl?taskId=2YfL_XhmYlxQ&node=397404&targetmode=proteins) | hypothetical protein, conserved (2773 aa) |  |  |  | [**•**](http://string-db.org/newstring_cgi/show_coexpression_evidence.pl?taskId=2YfL_XhmYlxQ&node2=397404) |  |  |  |  | 0.736 | | | --- | --- | --- | --- | --- | --- | --- | --- | --- | --- | --- | --- | --- | --- | --- | --- | --- | --- | --- | --- | --- | --- | --- | --- | --- | --- | --- | --- | --- | --- | --- | --- | --- | --- | --- | --- | --- | --- | --- | --- | --- | --- | --- | --- | --- | --- | --- | --- | --- | --- | --- | --- | --- | --- | --- | --- | --- | --- | --- | --- | --- | --- | --- | --- | --- | --- | --- | --- | --- | --- | --- | --- | --- | --- | --- | --- | --- | --- | --- | --- | --- | --- | --- | --- | --- | --- | --- | --- | --- | --- | --- | --- | --- | --- | --- | --- | --- | --- | --- | --- | --- | --- | --- | --- | --- | --- | --- | --- | --- | --- | --- | --- | --- | --- | --- | --- | --- | --- | --- | --- | --- | --- | --- | --- | --- | --- | --- | --- | --- | --- | --- | --- | --- | --- | --- | --- | --- | --- | --- | --- | --- | --- | | | --- | --- | --- | --- | --- | --- | --- | --- | --- | --- | --- | --- | --- | --- | --- | --- | --- | --- | --- | --- | --- | --- | --- | --- | --- | --- | --- | --- | --- | --- | --- | --- | --- | --- | --- | --- | --- | --- | --- | --- | --- | --- | --- | --- | --- | --- | --- | --- | --- | --- | --- | --- | --- | --- | --- | --- | --- | --- | --- | --- | --- | --- | --- | --- | --- | --- | --- | --- | --- | --- | --- | --- | --- | --- | --- | --- | --- | --- | --- | --- | --- | --- | --- | --- | --- | --- | --- | --- | --- | --- | --- | --- | --- | --- | --- | --- | --- | --- | --- | --- | --- | --- | --- | --- | --- | --- | --- | --- | --- | --- | --- | --- | --- | --- | --- | --- | --- | --- | --- | --- | --- | --- | --- | --- | --- | --- | --- | --- | --- | --- | --- | --- | --- | --- | --- | --- | --- | --- | --- | --- | --- | --- | --- | |
| --- | --- | --- | --- | --- | --- | --- | --- | --- | --- | --- | --- | --- | --- | --- | --- | --- | --- | --- | --- | --- | --- | --- | --- | --- | --- | --- | --- | --- | --- | --- | --- | --- | --- | --- | --- | --- | --- | --- | --- | --- | --- | --- | --- | --- | --- | --- | --- | --- | --- | --- | --- | --- | --- | --- | --- | --- | --- | --- | --- | --- | --- | --- | --- | --- | --- | --- | --- | --- | --- | --- | --- | --- | --- | --- | --- | --- | --- | --- | --- | --- | --- | --- | --- | --- | --- | --- | --- | --- | --- | --- | --- | --- | --- | --- | --- | --- | --- | --- | --- | --- | --- | --- | --- | --- | --- | --- | --- | --- | --- | --- | --- | --- | --- | --- | --- | --- | --- | --- | --- | --- | --- | --- | --- | --- | --- | --- | --- | --- | --- | --- | --- | --- | --- | --- | --- | --- | --- | --- | --- | --- | --- | --- | --- |
| |  | | --- | |

45.

| | | | **Your Input:** **>PF3D7_1331600** | | |  |  |  |  |  |  |  |  |  | | --- | --- | --- | --- | --- | --- | --- | --- | --- | --- | --- | --- | |  | [MAL13P1.168-1](http://string-db.org/newstring_cgi/display_single_node.pl?taskId=xco_sKnOTkby&node=397036&targetmode=proteins) | hypothetical protein, conserved (267 aa) | |  |  | *(Plasmodium falciparum)* | | **Predicted Functional Partners:** | | | |  | [PF10_0360](http://string-db.org/newstring_cgi/display_single_node.pl?taskId=xco_sKnOTkby&node=398356&targetmode=proteins) | acetyl-coA transporter (464 aa) |  |  |  | [**•**](http://string-db.org/newstring_cgi/show_coexpression_evidence.pl?taskId=xco_sKnOTkby&node2=398356) |  |  |  |  | 0.935 | |  | [PFF0720w](http://string-db.org/newstring_cgi/display_single_node.pl?taskId=xco_sKnOTkby&node=401429&targetmode=proteins) | hypothetical membrane protein, conserved (1096 aa) |  |  |  | [**•**](http://string-db.org/newstring_cgi/show_coexpression_evidence.pl?taskId=xco_sKnOTkby&node2=401429) |  |  |  |  | 0.901 | |  | [PFI0565w](http://string-db.org/newstring_cgi/display_single_node.pl?taskId=xco_sKnOTkby&node=401724&targetmode=proteins) | hypothetical protein, conserved (474 aa) |  |  |  | [**•**](http://string-db.org/newstring_cgi/show_coexpression_evidence.pl?taskId=xco_sKnOTkby&node2=401724) |  |  |  |  | 0.863 | |  | [Pf92](http://string-db.org/newstring_cgi/display_single_node.pl?taskId=xco_sKnOTkby&node=399245&targetmode=proteins) | cysteine-rich surface protein (796 aa) |  |  |  | [**•**](http://string-db.org/newstring_cgi/show_coexpression_evidence.pl?taskId=xco_sKnOTkby&node2=399245) |  |  |  |  | 0.863 | |  | [PfPDI-9](http://string-db.org/newstring_cgi/display_single_node.pl?taskId=xco_sKnOTkby&node=401802&targetmode=proteins) | protein disulfide isomerase, putative (515 aa) |  |  |  | [**•**](http://string-db.org/newstring_cgi/show_coexpression_evidence.pl?taskId=xco_sKnOTkby&node2=401802) |  |  |  |  | 0.861 | |  | [PFF0375c](http://string-db.org/newstring_cgi/display_single_node.pl?taskId=xco_sKnOTkby&node=401358&targetmode=proteins) | hypothetical protein, conserved (847 aa) |  |  |  | [**•**](http://string-db.org/newstring_cgi/show_coexpression_evidence.pl?taskId=xco_sKnOTkby&node2=401358) |  |  |  |  | 0.861 | |  | [MAL8P1.53](http://string-db.org/newstring_cgi/display_single_node.pl?taskId=xco_sKnOTkby&node=397653&targetmode=proteins) | hypothetical protein, conserved (514 aa) |  |  |  | [**•**](http://string-db.org/newstring_cgi/show_coexpression_evidence.pl?taskId=xco_sKnOTkby&node2=397653) |  |  |  |  | 0.861 | |  | [PFF0670w-1](http://string-db.org/newstring_cgi/display_single_node.pl?taskId=xco_sKnOTkby&node=401418&targetmode=proteins) | hypothetical protein, conserved (4095 aa) |  |  |  | [**•**](http://string-db.org/newstring_cgi/show_coexpression_evidence.pl?taskId=xco_sKnOTkby&node2=401418) |  |  |  |  | 0.859 | |  | [PF10_0020](http://string-db.org/newstring_cgi/display_single_node.pl?taskId=xco_sKnOTkby&node=397998&targetmode=proteins) | hypothetical protein (763 aa) |  |  |  | [**•**](http://string-db.org/newstring_cgi/show_coexpression_evidence.pl?taskId=xco_sKnOTkby&node2=397998) |  |  |  |  | 0.859 | |  | [cyc-2](http://string-db.org/newstring_cgi/display_single_node.pl?taskId=xco_sKnOTkby&node=402256&targetmode=proteins) | hypothetical protein, conserved (2281 aa) |  |  |  | [**•**](http://string-db.org/newstring_cgi/show_coexpression_evidence.pl?taskId=xco_sKnOTkby&node2=402256) |  |  |  |  | 0.859 | |  | [gpi1](http://string-db.org/newstring_cgi/display_single_node.pl?taskId=xco_sKnOTkby&node=401469&targetmode=proteins) | N-acetylglucosamine transferase (641 aa) |  |  |  | [**•**](http://string-db.org/newstring_cgi/show_coexpression_evidence.pl?taskId=xco_sKnOTkby&node2=401469) |  |  |  |  | 0.857 | |  | [PF14_0105](http://string-db.org/newstring_cgi/display_single_node.pl?taskId=xco_sKnOTkby&node=399374&targetmode=proteins) | hypothetical protein (334 aa) |  |  |  | [**•**](http://string-db.org/newstring_cgi/show_coexpression_evidence.pl?taskId=xco_sKnOTkby&node2=399374) |  |  |  |  | 0.857 | |  | [PF14_0215](http://string-db.org/newstring_cgi/display_single_node.pl?taskId=xco_sKnOTkby&node=399486&targetmode=proteins) | hypothetical protein (518 aa) |  |  |  | [**•**](http://string-db.org/newstring_cgi/show_coexpression_evidence.pl?taskId=xco_sKnOTkby&node2=399486) |  |  |  |  | 0.857 | |  | [PFF0335c](http://string-db.org/newstring_cgi/display_single_node.pl?taskId=xco_sKnOTkby&node=401350&targetmode=proteins) | hypothetical protein (299 aa) |  |  |  | [**•**](http://string-db.org/newstring_cgi/show_coexpression_evidence.pl?taskId=xco_sKnOTkby&node2=401350) |  |  |  |  | 0.855 | |  | [PFF0640w](http://string-db.org/newstring_cgi/display_single_node.pl?taskId=xco_sKnOTkby&node=401412&targetmode=proteins) | hypothetical protein, conserved (60 aa) |  |  |  | [**•**](http://string-db.org/newstring_cgi/show_coexpression_evidence.pl?taskId=xco_sKnOTkby&node2=401412) |  |  |  |  | 0.853 | |  | [PFC0250c](http://string-db.org/newstring_cgi/display_single_node.pl?taskId=xco_sKnOTkby&node=400512&targetmode=proteins) | AP endonuclease (DNA-(apurinic or apyrimidinic site) lyase), putative (617 aa) |  |  |  | [**•**](http://string-db.org/newstring_cgi/show_coexpression_evidence.pl?taskId=xco_sKnOTkby&node2=400512) |  |  |  |  | 0.847 | |  | [PFA_0285c](http://string-db.org/newstring_cgi/display_single_node.pl?taskId=xco_sKnOTkby&node=400130&targetmode=proteins) | hypothetical protein, conserved (832 aa) |  |  |  | [**•**](http://string-db.org/newstring_cgi/show_coexpression_evidence.pl?taskId=xco_sKnOTkby&node2=400130) |  |  |  |  | 0.847 | |  | [PF13_0032](http://string-db.org/newstring_cgi/display_single_node.pl?taskId=xco_sKnOTkby&node=398957&targetmode=proteins) | hydrolase, putative (478 aa) |  |  |  | [**•**](http://string-db.org/newstring_cgi/show_coexpression_evidence.pl?taskId=xco_sKnOTkby&node2=398957) |  |  |  |  | 0.847 | |  | [PFL0580w](http://string-db.org/newstring_cgi/display_single_node.pl?taskId=xco_sKnOTkby&node=402101&targetmode=proteins) | DNA replication licensing factor mcm5, putative (758 aa) |  |  |  | [**•**](http://string-db.org/newstring_cgi/show_coexpression_evidence.pl?taskId=xco_sKnOTkby&node2=402101) |  |  |  |  | 0.845 | |  | [PFA_0485w](http://string-db.org/newstring_cgi/display_single_node.pl?taskId=xco_sKnOTkby&node=400171&targetmode=proteins) | dolichol kinase (783 aa) |  |  |  | [**•**](http://string-db.org/newstring_cgi/show_coexpression_evidence.pl?taskId=xco_sKnOTkby&node2=400171) |  |  |  |  | 0.845 | | | --- | --- | --- | --- | --- | --- | --- | --- | --- | --- | --- | --- | --- | --- | --- | --- | --- | --- | --- | --- | --- | --- | --- | --- | --- | --- | --- | --- | --- | --- | --- | --- | --- | --- | --- | --- | --- | --- | --- | --- | --- | --- | --- | --- | --- | --- | --- | --- | --- | --- | --- | --- | --- | --- | --- | --- | --- | --- | --- | --- | --- | --- | --- | --- | --- | --- | --- | --- | --- | --- | --- | --- | --- | --- | --- | --- | --- | --- | --- | --- | --- | --- | --- | --- | --- | --- | --- | --- | --- | --- | --- | --- | --- | --- | --- | --- | --- | --- | --- | --- | --- | --- | --- | --- | --- | --- | --- | --- | --- | --- | --- | --- | --- | --- | --- | --- | --- | --- | --- | --- | --- | --- | --- | --- | --- | --- | --- | --- | --- | --- | --- | --- | --- | --- | --- | --- | --- | --- | --- | --- | --- | --- | --- | --- | --- | --- | --- | --- | --- | --- | --- | --- | --- | --- | --- | --- | --- | --- | --- | --- | --- | --- | --- | --- | --- | --- | --- | --- | --- | --- | --- | --- | --- | --- | --- | --- | --- | --- | --- | --- | --- | --- | --- | --- | --- | --- | --- | --- | --- | --- | --- | --- | --- | --- | --- | --- | --- | --- | --- | --- | --- | --- | --- | --- | --- | --- | --- | --- | --- | --- | --- | --- | --- | --- | --- | --- | --- | --- | --- | --- | --- | --- | --- | --- | --- | --- | --- | --- | --- | --- | --- | --- | --- | --- | --- | --- | --- | --- | --- | --- | --- | --- | --- | --- | --- | --- | --- | --- | --- | --- | --- | --- | --- | --- | --- | --- | --- | --- | --- | --- | --- | --- | | | --- | --- | --- | --- | --- | --- | --- | --- | --- | --- | --- | --- | --- | --- | --- | --- | --- | --- | --- | --- | --- | --- | --- | --- | --- | --- | --- | --- | --- | --- | --- | --- | --- | --- | --- | --- | --- | --- | --- | --- | --- | --- | --- | --- | --- | --- | --- | --- | --- | --- | --- | --- | --- | --- | --- | --- | --- | --- | --- | --- | --- | --- | --- | --- | --- | --- | --- | --- | --- | --- | --- | --- | --- | --- | --- | --- | --- | --- | --- | --- | --- | --- | --- | --- | --- | --- | --- | --- | --- | --- | --- | --- | --- | --- | --- | --- | --- | --- | --- | --- | --- | --- | --- | --- | --- | --- | --- | --- | --- | --- | --- | --- | --- | --- | --- | --- | --- | --- | --- | --- | --- | --- | --- | --- | --- | --- | --- | --- | --- | --- | --- | --- | --- | --- | --- | --- | --- | --- | --- | --- | --- | --- | --- | --- | --- | --- | --- | --- | --- | --- | --- | --- | --- | --- | --- | --- | --- | --- | --- | --- | --- | --- | --- | --- | --- | --- | --- | --- | --- | --- | --- | --- | --- | --- | --- | --- | --- | --- | --- | --- | --- | --- | --- | --- | --- | --- | --- | --- | --- | --- | --- | --- | --- | --- | --- | --- | --- | --- | --- | --- | --- | --- | --- | --- | --- | --- | --- | --- | --- | --- | --- | --- | --- | --- | --- | --- | --- | --- | --- | --- | --- | --- | --- | --- | --- | --- | --- | --- | --- | --- | --- | --- | --- | --- | --- | --- | --- | --- | --- | --- | --- | --- | --- | --- | --- | --- | --- | --- | --- | --- | --- | --- | --- | --- | --- | --- | --- | --- | --- | --- | --- | --- | --- | |
| --- | --- | --- | --- | --- | --- | --- | --- | --- | --- | --- | --- | --- | --- | --- | --- | --- | --- | --- | --- | --- | --- | --- | --- | --- | --- | --- | --- | --- | --- | --- | --- | --- | --- | --- | --- | --- | --- | --- | --- | --- | --- | --- | --- | --- | --- | --- | --- | --- | --- | --- | --- | --- | --- | --- | --- | --- | --- | --- | --- | --- | --- | --- | --- | --- | --- | --- | --- | --- | --- | --- | --- | --- | --- | --- | --- | --- | --- | --- | --- | --- | --- | --- | --- | --- | --- | --- | --- | --- | --- | --- | --- | --- | --- | --- | --- | --- | --- | --- | --- | --- | --- | --- | --- | --- | --- | --- | --- | --- | --- | --- | --- | --- | --- | --- | --- | --- | --- | --- | --- | --- | --- | --- | --- | --- | --- | --- | --- | --- | --- | --- | --- | --- | --- | --- | --- | --- | --- | --- | --- | --- | --- | --- | --- | --- | --- | --- | --- | --- | --- | --- | --- | --- | --- | --- | --- | --- | --- | --- | --- | --- | --- | --- | --- | --- | --- | --- | --- | --- | --- | --- | --- | --- | --- | --- | --- | --- | --- | --- | --- | --- | --- | --- | --- | --- | --- | --- | --- | --- | --- | --- | --- | --- | --- | --- | --- | --- | --- | --- | --- | --- | --- | --- | --- | --- | --- | --- | --- | --- | --- | --- | --- | --- | --- | --- | --- | --- | --- | --- | --- | --- | --- | --- | --- | --- | --- | --- | --- | --- | --- | --- | --- | --- | --- | --- | --- | --- | --- | --- | --- | --- | --- | --- | --- | --- | --- | --- | --- | --- | --- | --- | --- | --- | --- | --- | --- | --- | --- | --- | --- | --- | --- | --- | --- |
| |  | | --- | |

46.

| | | | **Your Input:** **>PF3D7_1340600** | | |  |  |  |  |  |  |  |  |  | | --- | --- | --- | --- | --- | --- | --- | --- | --- | --- | --- | --- | |  | [PF13_0222](http://string-db.org/newstring_cgi/display_single_node.pl?taskId=uTk4_QW_wOrt&node=399135&targetmode=proteins) | RNA lariat debranching enzyme, putative (575 aa) | |  |  | *(Plasmodium falciparum)* | | **Predicted Functional Partners:** | | | |  | [PF14_0036](http://string-db.org/newstring_cgi/display_single_node.pl?taskId=uTk4_QW_wOrt&node=399305&targetmode=proteins) | acid phosphatase, putative (302 aa) |  |  |  | [**•**](http://string-db.org/newstring_cgi/show_coexpression_evidence.pl?taskId=uTk4_QW_wOrt&node2=399305) |  |  | [**•**](http://string-db.org/newstring_cgi/show_textmining_evidence.pl?taskId=uTk4_QW_wOrt&node2=399305) |  | 0.697 | |  | [PFL0300c](http://string-db.org/newstring_cgi/display_single_node.pl?taskId=uTk4_QW_wOrt&node=402045&targetmode=proteins) | phosphoesterase, putative (304 aa) |  |  |  |  |  |  | [**•**](http://string-db.org/newstring_cgi/show_textmining_evidence.pl?taskId=uTk4_QW_wOrt&node2=402045) |  | 0.688 | |  | [PF14_0660](http://string-db.org/newstring_cgi/display_single_node.pl?taskId=uTk4_QW_wOrt&node=399944&targetmode=proteins) | hypothetical protein (358 aa) |  |  |  |  |  |  | [**•**](http://string-db.org/newstring_cgi/show_textmining_evidence.pl?taskId=uTk4_QW_wOrt&node2=399944) |  | 0.688 | |  | [PF14_0614](http://string-db.org/newstring_cgi/display_single_node.pl?taskId=uTk4_QW_wOrt&node=399896&targetmode=proteins) | hypothetical protein (1502 aa) |  |  |  |  |  |  | [**•**](http://string-db.org/newstring_cgi/show_textmining_evidence.pl?taskId=uTk4_QW_wOrt&node2=399896) |  | 0.688 | |  | [PF14_0064](http://string-db.org/newstring_cgi/display_single_node.pl?taskId=uTk4_QW_wOrt&node=399333&targetmode=proteins) | vacuolar protein sorting 29, putative (194 aa) |  |  |  |  |  |  | [**•**](http://string-db.org/newstring_cgi/show_textmining_evidence.pl?taskId=uTk4_QW_wOrt&node2=399333) |  | 0.688 | |  | [PFI1360c](http://string-db.org/newstring_cgi/display_single_node.pl?taskId=uTk4_QW_wOrt&node=401886&targetmode=proteins) | serine%2Fthreonine protein phosphatase, putative (312 aa) |  |  |  |  |  |  | [**•**](http://string-db.org/newstring_cgi/show_textmining_evidence.pl?taskId=uTk4_QW_wOrt&node2=401886) |  | 0.651 | |  | [PFC0595c](http://string-db.org/newstring_cgi/display_single_node.pl?taskId=uTk4_QW_wOrt&node=400594&targetmode=proteins) | serine%2Fthreonine protein phosphatase, putative (308 aa) |  |  |  |  |  |  | [**•**](http://string-db.org/newstring_cgi/show_textmining_evidence.pl?taskId=uTk4_QW_wOrt&node2=400594) |  | 0.651 | |  | [PP1](http://string-db.org/newstring_cgi/display_single_node.pl?taskId=uTk4_QW_wOrt&node=399412&targetmode=proteins) | serine%2Fthreonine protein phosphatase, putative (304 aa) |  |  |  |  |  |  | [**•**](http://string-db.org/newstring_cgi/show_textmining_evidence.pl?taskId=uTk4_QW_wOrt&node2=399412) |  | 0.651 | |  | [PF08_0129](http://string-db.org/newstring_cgi/display_single_node.pl?taskId=uTk4_QW_wOrt&node=397966&targetmode=proteins) | protein phosphatase, putative (604 aa) |  |  |  |  |  |  | [**•**](http://string-db.org/newstring_cgi/show_textmining_evidence.pl?taskId=uTk4_QW_wOrt&node2=397966) |  | 0.651 | |  | [PFI1245c](http://string-db.org/newstring_cgi/display_single_node.pl?taskId=uTk4_QW_wOrt&node=401862&targetmode=proteins) | Protein phosphatase-beta (466 aa) |  |  |  |  |  |  | [**•**](http://string-db.org/newstring_cgi/show_textmining_evidence.pl?taskId=uTk4_QW_wOrt&node2=401862) |  | 0.649 | |  | [PF14_0630](http://string-db.org/newstring_cgi/display_single_node.pl?taskId=uTk4_QW_wOrt&node=399913&targetmode=proteins) | protein serine%2Fthreonine phosphatase (889 aa) |  |  |  |  |  |  | [**•**](http://string-db.org/newstring_cgi/show_textmining_evidence.pl?taskId=uTk4_QW_wOrt&node2=399913) |  | 0.649 | |  | [PP7](http://string-db.org/newstring_cgi/display_single_node.pl?taskId=uTk4_QW_wOrt&node=399495&targetmode=proteins) | PP1-like protein serine%2Fthreonine phosphatase (959 aa) |  |  |  |  |  |  | [**•**](http://string-db.org/newstring_cgi/show_textmining_evidence.pl?taskId=uTk4_QW_wOrt&node2=399495) |  | 0.649 | |  | [PFB0370c](http://string-db.org/newstring_cgi/display_single_node.pl?taskId=uTk4_QW_wOrt&node=400303&targetmode=proteins) | RNA-binding protein, putative (300 aa) |  |  |  | [**•**](http://string-db.org/newstring_cgi/show_coexpression_evidence.pl?taskId=uTk4_QW_wOrt&node2=400303) |  |  |  |  | 0.545 | |  | [PFC0365w](http://string-db.org/newstring_cgi/display_single_node.pl?taskId=uTk4_QW_wOrt&node=400540&targetmode=proteins) | conserved protein, putative (532 aa) |  |  |  | [**•**](http://string-db.org/newstring_cgi/show_coexpression_evidence.pl?taskId=uTk4_QW_wOrt&node2=400540) |  |  | [**•**](http://string-db.org/newstring_cgi/show_textmining_evidence.pl?taskId=uTk4_QW_wOrt&node2=400540) |  | 0.492 | |  | [PFF1150w](http://string-db.org/newstring_cgi/display_single_node.pl?taskId=uTk4_QW_wOrt&node=401517&targetmode=proteins) | ribonuclease H1 large subunit, putative; Endonuclease that specifically degrades the RNA of RNA [...] (288 aa) |  |  |  | [**•**](http://string-db.org/newstring_cgi/show_coexpression_evidence.pl?taskId=uTk4_QW_wOrt&node2=401517) |  |  |  |  | 0.485 | |  | [PFL0320w](http://string-db.org/newstring_cgi/display_single_node.pl?taskId=uTk4_QW_wOrt&node=402049&targetmode=proteins) | hypothetical protein, conserved (346 aa) |  |  |  |  |  |  | [**•**](http://string-db.org/newstring_cgi/show_textmining_evidence.pl?taskId=uTk4_QW_wOrt&node2=402049) |  | 0.481 | |  | [PF13_0027](http://string-db.org/newstring_cgi/display_single_node.pl?taskId=uTk4_QW_wOrt&node=398952&targetmode=proteins) | hypothetical protein, conserved (771 aa) |  |  |  |  |  |  | [**•**](http://string-db.org/newstring_cgi/show_textmining_evidence.pl?taskId=uTk4_QW_wOrt&node2=398952) |  | 0.481 | |  | [MAL13P1.168-1](http://string-db.org/newstring_cgi/display_single_node.pl?taskId=uTk4_QW_wOrt&node=397036&targetmode=proteins) | hypothetical protein, conserved (267 aa) |  |  |  |  |  |  | [**•**](http://string-db.org/newstring_cgi/show_textmining_evidence.pl?taskId=uTk4_QW_wOrt&node2=397036) |  | 0.481 | |  | [PF14_0525](http://string-db.org/newstring_cgi/display_single_node.pl?taskId=uTk4_QW_wOrt&node=399804&targetmode=proteins) | hypothetical protein (89 aa) |  |  |  |  |  |  | [**•**](http://string-db.org/newstring_cgi/show_textmining_evidence.pl?taskId=uTk4_QW_wOrt&node2=399804) |  | 0.480 | |  | [PF11_0281](http://string-db.org/newstring_cgi/display_single_node.pl?taskId=uTk4_QW_wOrt&node=398671&targetmode=proteins) | hypothetical protein (247 aa) |  |  |  |  |  |  | [**•**](http://string-db.org/newstring_cgi/show_textmining_evidence.pl?taskId=uTk4_QW_wOrt&node2=398671) |  | 0.480 | | | --- | --- | --- | --- | --- | --- | --- | --- | --- | --- | --- | --- | --- | --- | --- | --- | --- | --- | --- | --- | --- | --- | --- | --- | --- | --- | --- | --- | --- | --- | --- | --- | --- | --- | --- | --- | --- | --- | --- | --- | --- | --- | --- | --- | --- | --- | --- | --- | --- | --- | --- | --- | --- | --- | --- | --- | --- | --- | --- | --- | --- | --- | --- | --- | --- | --- | --- | --- | --- | --- | --- | --- | --- | --- | --- | --- | --- | --- | --- | --- | --- | --- | --- | --- | --- | --- | --- | --- | --- | --- | --- | --- | --- | --- | --- | --- | --- | --- | --- | --- | --- | --- | --- | --- | --- | --- | --- | --- | --- | --- | --- | --- | --- | --- | --- | --- | --- | --- | --- | --- | --- | --- | --- | --- | --- | --- | --- | --- | --- | --- | --- | --- | --- | --- | --- | --- | --- | --- | --- | --- | --- | --- | --- | --- | --- | --- | --- | --- | --- | --- | --- | --- | --- | --- | --- | --- | --- | --- | --- | --- | --- | --- | --- | --- | --- | --- | --- | --- | --- | --- | --- | --- | --- | --- | --- | --- | --- | --- | --- | --- | --- | --- | --- | --- | --- | --- | --- | --- | --- | --- | --- | --- | --- | --- | --- | --- | --- | --- | --- | --- | --- | --- | --- | --- | --- | --- | --- | --- | --- | --- | --- | --- | --- | --- | --- | --- | --- | --- | --- | --- | --- | --- | --- | --- | --- | --- | --- | --- | --- | --- | --- | --- | --- | --- | --- | --- | --- | --- | --- | --- | --- | --- | --- | --- | --- | --- | --- | --- | --- | --- | --- | --- | --- | --- | --- | --- | --- | --- | --- | --- | --- | --- | | | --- | --- | --- | --- | --- | --- | --- | --- | --- | --- | --- | --- | --- | --- | --- | --- | --- | --- | --- | --- | --- | --- | --- | --- | --- | --- | --- | --- | --- | --- | --- | --- | --- | --- | --- | --- | --- | --- | --- | --- | --- | --- | --- | --- | --- | --- | --- | --- | --- | --- | --- | --- | --- | --- | --- | --- | --- | --- | --- | --- | --- | --- | --- | --- | --- | --- | --- | --- | --- | --- | --- | --- | --- | --- | --- | --- | --- | --- | --- | --- | --- | --- | --- | --- | --- | --- | --- | --- | --- | --- | --- | --- | --- | --- | --- | --- | --- | --- | --- | --- | --- | --- | --- | --- | --- | --- | --- | --- | --- | --- | --- | --- | --- | --- | --- | --- | --- | --- | --- | --- | --- | --- | --- | --- | --- | --- | --- | --- | --- | --- | --- | --- | --- | --- | --- | --- | --- | --- | --- | --- | --- | --- | --- | --- | --- | --- | --- | --- | --- | --- | --- | --- | --- | --- | --- | --- | --- | --- | --- | --- | --- | --- | --- | --- | --- | --- | --- | --- | --- | --- | --- | --- | --- | --- | --- | --- | --- | --- | --- | --- | --- | --- | --- | --- | --- | --- | --- | --- | --- | --- | --- | --- | --- | --- | --- | --- | --- | --- | --- | --- | --- | --- | --- | --- | --- | --- | --- | --- | --- | --- | --- | --- | --- | --- | --- | --- | --- | --- | --- | --- | --- | --- | --- | --- | --- | --- | --- | --- | --- | --- | --- | --- | --- | --- | --- | --- | --- | --- | --- | --- | --- | --- | --- | --- | --- | --- | --- | --- | --- | --- | --- | --- | --- | --- | --- | --- | --- | --- | --- | --- | --- | --- | --- | |
| --- | --- | --- | --- | --- | --- | --- | --- | --- | --- | --- | --- | --- | --- | --- | --- | --- | --- | --- | --- | --- | --- | --- | --- | --- | --- | --- | --- | --- | --- | --- | --- | --- | --- | --- | --- | --- | --- | --- | --- | --- | --- | --- | --- | --- | --- | --- | --- | --- | --- | --- | --- | --- | --- | --- | --- | --- | --- | --- | --- | --- | --- | --- | --- | --- | --- | --- | --- | --- | --- | --- | --- | --- | --- | --- | --- | --- | --- | --- | --- | --- | --- | --- | --- | --- | --- | --- | --- | --- | --- | --- | --- | --- | --- | --- | --- | --- | --- | --- | --- | --- | --- | --- | --- | --- | --- | --- | --- | --- | --- | --- | --- | --- | --- | --- | --- | --- | --- | --- | --- | --- | --- | --- | --- | --- | --- | --- | --- | --- | --- | --- | --- | --- | --- | --- | --- | --- | --- | --- | --- | --- | --- | --- | --- | --- | --- | --- | --- | --- | --- | --- | --- | --- | --- | --- | --- | --- | --- | --- | --- | --- | --- | --- | --- | --- | --- | --- | --- | --- | --- | --- | --- | --- | --- | --- | --- | --- | --- | --- | --- | --- | --- | --- | --- | --- | --- | --- | --- | --- | --- | --- | --- | --- | --- | --- | --- | --- | --- | --- | --- | --- | --- | --- | --- | --- | --- | --- | --- | --- | --- | --- | --- | --- | --- | --- | --- | --- | --- | --- | --- | --- | --- | --- | --- | --- | --- | --- | --- | --- | --- | --- | --- | --- | --- | --- | --- | --- | --- | --- | --- | --- | --- | --- | --- | --- | --- | --- | --- | --- | --- | --- | --- | --- | --- | --- | --- | --- | --- | --- | --- | --- | --- | --- | --- |
| |  | | --- | |

47.

| | | | **Your Input:** **>PF3D7_1354200** | | |  |  |  |  |  |  |  |  |  | | --- | --- | --- | --- | --- | --- | --- | --- | --- | --- | --- | --- | |  | [PF13_0285](http://string-db.org/newstring_cgi/display_single_node.pl?taskId=GlLLdjik974_&node=399197&targetmode=proteins) | inositol-polyphosphate 5-phosphatase (803 aa) | |  |  | *(Plasmodium falciparum)* | | **Predicted Functional Partners:** | | | |  | [PF08_0031](http://string-db.org/newstring_cgi/display_single_node.pl?taskId=GlLLdjik974_&node=397868&targetmode=proteins) | 2-Oxoglutarat/malate translocator protein, putative (318 aa) |  |  |  |  |  |  | [**•**](http://string-db.org/newstring_cgi/show_textmining_evidence.pl?taskId=GlLLdjik974_&node2=397868) |  | 0.780 | |  | [PfATPase7](http://string-db.org/newstring_cgi/display_single_node.pl?taskId=GlLLdjik974_&node=400641&targetmode=proteins) | P-type ATPase, putative (1864 aa) |  |  |  |  |  |  | [**•**](http://string-db.org/newstring_cgi/show_textmining_evidence.pl?taskId=GlLLdjik974_&node2=400641) |  | 0.688 | |  | [PF10_0014-1](http://string-db.org/newstring_cgi/display_single_node.pl?taskId=GlLLdjik974_&node=397992&targetmode=proteins) | hypothetical protein (193 aa) |  |  |  |  |  |  | [**•**](http://string-db.org/newstring_cgi/show_textmining_evidence.pl?taskId=GlLLdjik974_&node2=397992) |  | 0.683 | |  | [PF14_0749](http://string-db.org/newstring_cgi/display_single_node.pl?taskId=GlLLdjik974_&node=400035&targetmode=proteins) | acyl CoA binding protein (90 aa) |  |  |  |  |  |  | [**•**](http://string-db.org/newstring_cgi/show_textmining_evidence.pl?taskId=GlLLdjik974_&node2=400035) |  | 0.681 | |  | [PF10_0016-1](http://string-db.org/newstring_cgi/display_single_node.pl?taskId=GlLLdjik974_&node=397994&targetmode=proteins) | acyl CoA binding protein, putative (90 aa) |  |  |  |  |  |  | [**•**](http://string-db.org/newstring_cgi/show_textmining_evidence.pl?taskId=GlLLdjik974_&node2=397994) |  | 0.659 | |  | [MAL13P1.82](http://string-db.org/newstring_cgi/display_single_node.pl?taskId=GlLLdjik974_&node=397324&targetmode=proteins) | phosphatidylinositol synthase, putative (173 aa) |  |  |  |  |  |  | [**•**](http://string-db.org/newstring_cgi/show_textmining_evidence.pl?taskId=GlLLdjik974_&node2=397324) |  | 0.655 | |  | [fabZ](http://string-db.org/newstring_cgi/display_single_node.pl?taskId=GlLLdjik974_&node=399044&targetmode=proteins) | beta-hydroxyacyl-acp dehydratase precursor (230 aa) |  |  |  |  |  |  | [**•**](http://string-db.org/newstring_cgi/show_textmining_evidence.pl?taskId=GlLLdjik974_&node2=399044) |  | 0.649 | |  | [PF13_0358](http://string-db.org/newstring_cgi/display_single_node.pl?taskId=GlLLdjik974_&node=399265&targetmode=proteins) | mitochondrial import inner membrane translocase, putative (92 aa) |  |  |  |  |  |  | [**•**](http://string-db.org/newstring_cgi/show_textmining_evidence.pl?taskId=GlLLdjik974_&node2=399265) |  | 0.579 | |  | [PF13_0300](http://string-db.org/newstring_cgi/display_single_node.pl?taskId=GlLLdjik974_&node=399211&targetmode=proteins) | mitochondrial inner membrane translocase, putative (167 aa) |  |  |  |  |  |  | [**•**](http://string-db.org/newstring_cgi/show_textmining_evidence.pl?taskId=GlLLdjik974_&node2=399211) |  | 0.579 | |  | [PF10_0210](http://string-db.org/newstring_cgi/display_single_node.pl?taskId=GlLLdjik974_&node=398197&targetmode=proteins) | deoxyribose-phosphate aldolase, putative (263 aa) |  |  |  |  |  |  | [**•**](http://string-db.org/newstring_cgi/show_textmining_evidence.pl?taskId=GlLLdjik974_&node2=398197) |  | 0.579 | | | --- | --- | --- | --- | --- | --- | --- | --- | --- | --- | --- | --- | --- | --- | --- | --- | --- | --- | --- | --- | --- | --- | --- | --- | --- | --- | --- | --- | --- | --- | --- | --- | --- | --- | --- | --- | --- | --- | --- | --- | --- | --- | --- | --- | --- | --- | --- | --- | --- | --- | --- | --- | --- | --- | --- | --- | --- | --- | --- | --- | --- | --- | --- | --- | --- | --- | --- | --- | --- | --- | --- | --- | --- | --- | --- | --- | --- | --- | --- | --- | --- | --- | --- | --- | --- | --- | --- | --- | --- | --- | --- | --- | --- | --- | --- | --- | --- | --- | --- | --- | --- | --- | --- | --- | --- | --- | --- | --- | --- | --- | --- | --- | --- | --- | --- | --- | --- | --- | --- | --- | --- | --- | --- | --- | --- | --- | --- | --- | --- | --- | --- | --- | --- | --- | --- | --- | --- | --- | --- | --- | --- | --- | | | --- | --- | --- | --- | --- | --- | --- | --- | --- | --- | --- | --- | --- | --- | --- | --- | --- | --- | --- | --- | --- | --- | --- | --- | --- | --- | --- | --- | --- | --- | --- | --- | --- | --- | --- | --- | --- | --- | --- | --- | --- | --- | --- | --- | --- | --- | --- | --- | --- | --- | --- | --- | --- | --- | --- | --- | --- | --- | --- | --- | --- | --- | --- | --- | --- | --- | --- | --- | --- | --- | --- | --- | --- | --- | --- | --- | --- | --- | --- | --- | --- | --- | --- | --- | --- | --- | --- | --- | --- | --- | --- | --- | --- | --- | --- | --- | --- | --- | --- | --- | --- | --- | --- | --- | --- | --- | --- | --- | --- | --- | --- | --- | --- | --- | --- | --- | --- | --- | --- | --- | --- | --- | --- | --- | --- | --- | --- | --- | --- | --- | --- | --- | --- | --- | --- | --- | --- | --- | --- | --- | --- | --- | --- | |
| --- | --- | --- | --- | --- | --- | --- | --- | --- | --- | --- | --- | --- | --- | --- | --- | --- | --- | --- | --- | --- | --- | --- | --- | --- | --- | --- | --- | --- | --- | --- | --- | --- | --- | --- | --- | --- | --- | --- | --- | --- | --- | --- | --- | --- | --- | --- | --- | --- | --- | --- | --- | --- | --- | --- | --- | --- | --- | --- | --- | --- | --- | --- | --- | --- | --- | --- | --- | --- | --- | --- | --- | --- | --- | --- | --- | --- | --- | --- | --- | --- | --- | --- | --- | --- | --- | --- | --- | --- | --- | --- | --- | --- | --- | --- | --- | --- | --- | --- | --- | --- | --- | --- | --- | --- | --- | --- | --- | --- | --- | --- | --- | --- | --- | --- | --- | --- | --- | --- | --- | --- | --- | --- | --- | --- | --- | --- | --- | --- | --- | --- | --- | --- | --- | --- | --- | --- | --- | --- | --- | --- | --- | --- | --- |
| |  | | --- | |

48.

| | | | **Your Input:** **>PF3D7_1355500** | | |  |  |  |  |  |  |  |  |  | | --- | --- | --- | --- | --- | --- | --- | --- | --- | --- | --- | --- | |  | [PfPP5](http://string-db.org/newstring_cgi/display_single_node.pl?taskId=BLJO2IRVcWqD&node=397148&targetmode=proteins) | serine%2Fthreonine protein phosphatase pfPp5 (658 aa) | |  |  | *(Plasmodium falciparum)* | | **Predicted Functional Partners:** | | | |  | [PF07_0059](http://string-db.org/newstring_cgi/display_single_node.pl?taskId=BLJO2IRVcWqD&node=397761&targetmode=proteins) | 4-nitrophenylphosphatase, putative (322 aa) |  |  |  | [**•**](http://string-db.org/newstring_cgi/show_coexpression_evidence.pl?taskId=BLJO2IRVcWqD&node2=397761) |  |  | [**•**](http://string-db.org/newstring_cgi/show_textmining_evidence.pl?taskId=BLJO2IRVcWqD&node2=397761) |  | 0.707 | |  | [PFL2140c](http://string-db.org/newstring_cgi/display_single_node.pl?taskId=BLJO2IRVcWqD&node=402423&targetmode=proteins) | ADP-ribosylation factor GTPase-activating protein (332 aa) |  |  |  |  |  |  | [**•**](http://string-db.org/newstring_cgi/show_textmining_evidence.pl?taskId=BLJO2IRVcWqD&node2=402423) |  | 0.681 | |  | [PF07_0110](http://string-db.org/newstring_cgi/display_single_node.pl?taskId=BLJO2IRVcWqD&node=397814&targetmode=proteins) | hypothetical protein, conserved (519 aa) |  |  |  | [**•**](http://string-db.org/newstring_cgi/show_coexpression_evidence.pl?taskId=BLJO2IRVcWqD&node2=397814) |  |  | [**•**](http://string-db.org/newstring_cgi/show_textmining_evidence.pl?taskId=BLJO2IRVcWqD&node2=397814) |  | 0.668 | |  | [PF10_0203](http://string-db.org/newstring_cgi/display_single_node.pl?taskId=BLJO2IRVcWqD&node=398190&targetmode=proteins) | ADP-ribosylation factor; GTP-binding protein that functions as an allosteric activator of the c [...] (181 aa) |  |  |  |  |  |  | [**•**](http://string-db.org/newstring_cgi/show_textmining_evidence.pl?taskId=BLJO2IRVcWqD&node2=398190) |  | 0.649 | |  | [PF10_0124](http://string-db.org/newstring_cgi/display_single_node.pl?taskId=BLJO2IRVcWqD&node=398108&targetmode=proteins) | hypothetical protein (1438 aa) |  |  |  |  |  |  | [**•**](http://string-db.org/newstring_cgi/show_textmining_evidence.pl?taskId=BLJO2IRVcWqD&node2=398108) |  | 0.641 | |  | [PFE1370w](http://string-db.org/newstring_cgi/display_single_node.pl?taskId=BLJO2IRVcWqD&node=401235&targetmode=proteins) | hsp70 interacting protein, putative (458 aa) |  |  |  | [**•**](http://string-db.org/newstring_cgi/show_coexpression_evidence.pl?taskId=BLJO2IRVcWqD&node2=401235) |  |  | [**•**](http://string-db.org/newstring_cgi/show_textmining_evidence.pl?taskId=BLJO2IRVcWqD&node2=401235) |  | 0.628 | |  | [PFD0770c](http://string-db.org/newstring_cgi/display_single_node.pl?taskId=BLJO2IRVcWqD&node=400856&targetmode=proteins) | ribosomal protein l15, putative (220 aa) |  |  |  | [**•**](http://string-db.org/newstring_cgi/show_coexpression_evidence.pl?taskId=BLJO2IRVcWqD&node2=400856) |  |  | [**•**](http://string-db.org/newstring_cgi/show_textmining_evidence.pl?taskId=BLJO2IRVcWqD&node2=400856) |  | 0.615 | |  | [PfEF-1beta](http://string-db.org/newstring_cgi/display_single_node.pl?taskId=BLJO2IRVcWqD&node=401740&targetmode=proteins) | EF-1B (276 aa) |  |  |  | [**•**](http://string-db.org/newstring_cgi/show_coexpression_evidence.pl?taskId=BLJO2IRVcWqD&node2=401740) |  |  | [**•**](http://string-db.org/newstring_cgi/show_textmining_evidence.pl?taskId=BLJO2IRVcWqD&node2=401740) |  | 0.612 | |  | [PfCyP19](http://string-db.org/newstring_cgi/display_single_node.pl?taskId=BLJO2IRVcWqD&node=400671&targetmode=proteins) | PFCYP19, cyclophilin, peptidyl-prolyl cis-trans isomerase; PPIases accelerate the folding of pr [...] (171 aa) |  |  |  | [**•**](http://string-db.org/newstring_cgi/show_coexpression_evidence.pl?taskId=BLJO2IRVcWqD&node2=400671) |  |  | [**•**](http://string-db.org/newstring_cgi/show_textmining_evidence.pl?taskId=BLJO2IRVcWqD&node2=400671) |  | 0.612 | |  | [PF11_0351](http://string-db.org/newstring_cgi/display_single_node.pl?taskId=BLJO2IRVcWqD&node=398742&targetmode=proteins) | heat shock protein hsp70 homologue (663 aa) |  |  |  | [**•**](http://string-db.org/newstring_cgi/show_coexpression_evidence.pl?taskId=BLJO2IRVcWqD&node2=398742) |  |  | [**•**](http://string-db.org/newstring_cgi/show_textmining_evidence.pl?taskId=BLJO2IRVcWqD&node2=398742) |  | 0.612 | | | --- | --- | --- | --- | --- | --- | --- | --- | --- | --- | --- | --- | --- | --- | --- | --- | --- | --- | --- | --- | --- | --- | --- | --- | --- | --- | --- | --- | --- | --- | --- | --- | --- | --- | --- | --- | --- | --- | --- | --- | --- | --- | --- | --- | --- | --- | --- | --- | --- | --- | --- | --- | --- | --- | --- | --- | --- | --- | --- | --- | --- | --- | --- | --- | --- | --- | --- | --- | --- | --- | --- | --- | --- | --- | --- | --- | --- | --- | --- | --- | --- | --- | --- | --- | --- | --- | --- | --- | --- | --- | --- | --- | --- | --- | --- | --- | --- | --- | --- | --- | --- | --- | --- | --- | --- | --- | --- | --- | --- | --- | --- | --- | --- | --- | --- | --- | --- | --- | --- | --- | --- | --- | --- | --- | --- | --- | --- | --- | --- | --- | --- | --- | --- | --- | --- | --- | --- | --- | --- | --- | --- | --- | | | --- | --- | --- | --- | --- | --- | --- | --- | --- | --- | --- | --- | --- | --- | --- | --- | --- | --- | --- | --- | --- | --- | --- | --- | --- | --- | --- | --- | --- | --- | --- | --- | --- | --- | --- | --- | --- | --- | --- | --- | --- | --- | --- | --- | --- | --- | --- | --- | --- | --- | --- | --- | --- | --- | --- | --- | --- | --- | --- | --- | --- | --- | --- | --- | --- | --- | --- | --- | --- | --- | --- | --- | --- | --- | --- | --- | --- | --- | --- | --- | --- | --- | --- | --- | --- | --- | --- | --- | --- | --- | --- | --- | --- | --- | --- | --- | --- | --- | --- | --- | --- | --- | --- | --- | --- | --- | --- | --- | --- | --- | --- | --- | --- | --- | --- | --- | --- | --- | --- | --- | --- | --- | --- | --- | --- | --- | --- | --- | --- | --- | --- | --- | --- | --- | --- | --- | --- | --- | --- | --- | --- | --- | --- | |
| --- | --- | --- | --- | --- | --- | --- | --- | --- | --- | --- | --- | --- | --- | --- | --- | --- | --- | --- | --- | --- | --- | --- | --- | --- | --- | --- | --- | --- | --- | --- | --- | --- | --- | --- | --- | --- | --- | --- | --- | --- | --- | --- | --- | --- | --- | --- | --- | --- | --- | --- | --- | --- | --- | --- | --- | --- | --- | --- | --- | --- | --- | --- | --- | --- | --- | --- | --- | --- | --- | --- | --- | --- | --- | --- | --- | --- | --- | --- | --- | --- | --- | --- | --- | --- | --- | --- | --- | --- | --- | --- | --- | --- | --- | --- | --- | --- | --- | --- | --- | --- | --- | --- | --- | --- | --- | --- | --- | --- | --- | --- | --- | --- | --- | --- | --- | --- | --- | --- | --- | --- | --- | --- | --- | --- | --- | --- | --- | --- | --- | --- | --- | --- | --- | --- | --- | --- | --- | --- | --- | --- | --- | --- | --- |
|  |

49.

| | | | **Your Input:** **>PF3D7_1355700** | | |  |  |  |  |  |  |  |  |  | | --- | --- | --- | --- | --- | --- | --- | --- | --- | --- | --- | --- | |  | [MAL13P1.275-1](http://string-db.org/newstring_cgi/display_single_node.pl?taskId=VrvA4uMJlaDK&node=397149&targetmode=proteins) | NLI interacting factor-like phosphatase, putative (1288 aa) | |  |  | *(Plasmodium falciparum)* | | **Predicted Functional Partners:** | | | |  | [PFL0320w](http://string-db.org/newstring_cgi/display_single_node.pl?taskId=VrvA4uMJlaDK&node=402049&targetmode=proteins) | hypothetical protein, conserved (346 aa) |  |  |  |  |  |  | [**•**](http://string-db.org/newstring_cgi/show_textmining_evidence.pl?taskId=VrvA4uMJlaDK&node2=402049) |  | 0.579 | |  | [PF14_0525](http://string-db.org/newstring_cgi/display_single_node.pl?taskId=VrvA4uMJlaDK&node=399804&targetmode=proteins) | hypothetical protein (89 aa) |  |  |  |  |  |  | [**•**](http://string-db.org/newstring_cgi/show_textmining_evidence.pl?taskId=VrvA4uMJlaDK&node2=399804) |  | 0.579 | |  | [PF13_0027](http://string-db.org/newstring_cgi/display_single_node.pl?taskId=VrvA4uMJlaDK&node=398952&targetmode=proteins) | hypothetical protein, conserved (771 aa) |  |  |  |  |  |  | [**•**](http://string-db.org/newstring_cgi/show_textmining_evidence.pl?taskId=VrvA4uMJlaDK&node2=398952) |  | 0.579 | |  | [PF11_0281](http://string-db.org/newstring_cgi/display_single_node.pl?taskId=VrvA4uMJlaDK&node=398671&targetmode=proteins) | hypothetical protein (247 aa) |  |  |  |  |  |  | [**•**](http://string-db.org/newstring_cgi/show_textmining_evidence.pl?taskId=VrvA4uMJlaDK&node2=398671) |  | 0.579 | |  | [MAL13P1.168-1](http://string-db.org/newstring_cgi/display_single_node.pl?taskId=VrvA4uMJlaDK&node=397036&targetmode=proteins) | hypothetical protein, conserved (267 aa) |  |  |  |  |  |  | [**•**](http://string-db.org/newstring_cgi/show_textmining_evidence.pl?taskId=VrvA4uMJlaDK&node2=397036) |  | 0.579 | |  | [PRL](http://string-db.org/newstring_cgi/display_single_node.pl?taskId=VrvA4uMJlaDK&node=398526&targetmode=proteins) | protein tyrosine phosphatase, putative (218 aa) |  |  |  | [**•**](http://string-db.org/newstring_cgi/show_coexpression_evidence.pl?taskId=VrvA4uMJlaDK&node2=398526) |  |  | [**•**](http://string-db.org/newstring_cgi/show_textmining_evidence.pl?taskId=VrvA4uMJlaDK&node2=398526) |  | 0.510 | |  | [PFL2365w](http://string-db.org/newstring_cgi/display_single_node.pl?taskId=VrvA4uMJlaDK&node=402468&targetmode=proteins) | hypothetical protein, conserved (1027 aa) |  |  |  |  |  |  | [**•**](http://string-db.org/newstring_cgi/show_textmining_evidence.pl?taskId=VrvA4uMJlaDK&node2=402468) |  | 0.484 | |  | [PF14_0523](http://string-db.org/newstring_cgi/display_single_node.pl?taskId=VrvA4uMJlaDK&node=399802&targetmode=proteins) | protein phosphatase 2C, putative (289 aa) |  |  |  |  |  |  | [**•**](http://string-db.org/newstring_cgi/show_textmining_evidence.pl?taskId=VrvA4uMJlaDK&node2=399802) |  | 0.484 | |  | [MAL8P1.108](http://string-db.org/newstring_cgi/display_single_node.pl?taskId=VrvA4uMJlaDK&node=397533&targetmode=proteins) | protein phosphatase, putative (303 aa) |  |  |  |  |  |  | [**•**](http://string-db.org/newstring_cgi/show_textmining_evidence.pl?taskId=VrvA4uMJlaDK&node2=397533) |  | 0.484 | |  | [PFE1010w](http://string-db.org/newstring_cgi/display_single_node.pl?taskId=VrvA4uMJlaDK&node=401160&targetmode=proteins) | protein phosphatase 2c, putative (697 aa) |  |  |  |  |  |  | [**•**](http://string-db.org/newstring_cgi/show_textmining_evidence.pl?taskId=VrvA4uMJlaDK&node2=401160) |  | 0.481 | | | --- | --- | --- | --- | --- | --- | --- | --- | --- | --- | --- | --- | --- | --- | --- | --- | --- | --- | --- | --- | --- | --- | --- | --- | --- | --- | --- | --- | --- | --- | --- | --- | --- | --- | --- | --- | --- | --- | --- | --- | --- | --- | --- | --- | --- | --- | --- | --- | --- | --- | --- | --- | --- | --- | --- | --- | --- | --- | --- | --- | --- | --- | --- | --- | --- | --- | --- | --- | --- | --- | --- | --- | --- | --- | --- | --- | --- | --- | --- | --- | --- | --- | --- | --- | --- | --- | --- | --- | --- | --- | --- | --- | --- | --- | --- | --- | --- | --- | --- | --- | --- | --- | --- | --- | --- | --- | --- | --- | --- | --- | --- | --- | --- | --- | --- | --- | --- | --- | --- | --- | --- | --- | --- | --- | --- | --- | --- | --- | --- | --- | --- | --- | --- | --- | --- | --- | --- | --- | --- | --- | --- | --- | | | --- | --- | --- | --- | --- | --- | --- | --- | --- | --- | --- | --- | --- | --- | --- | --- | --- | --- | --- | --- | --- | --- | --- | --- | --- | --- | --- | --- | --- | --- | --- | --- | --- | --- | --- | --- | --- | --- | --- | --- | --- | --- | --- | --- | --- | --- | --- | --- | --- | --- | --- | --- | --- | --- | --- | --- | --- | --- | --- | --- | --- | --- | --- | --- | --- | --- | --- | --- | --- | --- | --- | --- | --- | --- | --- | --- | --- | --- | --- | --- | --- | --- | --- | --- | --- | --- | --- | --- | --- | --- | --- | --- | --- | --- | --- | --- | --- | --- | --- | --- | --- | --- | --- | --- | --- | --- | --- | --- | --- | --- | --- | --- | --- | --- | --- | --- | --- | --- | --- | --- | --- | --- | --- | --- | --- | --- | --- | --- | --- | --- | --- | --- | --- | --- | --- | --- | --- | --- | --- | --- | --- | --- | --- | |
| --- | --- | --- | --- | --- | --- | --- | --- | --- | --- | --- | --- | --- | --- | --- | --- | --- | --- | --- | --- | --- | --- | --- | --- | --- | --- | --- | --- | --- | --- | --- | --- | --- | --- | --- | --- | --- | --- | --- | --- | --- | --- | --- | --- | --- | --- | --- | --- | --- | --- | --- | --- | --- | --- | --- | --- | --- | --- | --- | --- | --- | --- | --- | --- | --- | --- | --- | --- | --- | --- | --- | --- | --- | --- | --- | --- | --- | --- | --- | --- | --- | --- | --- | --- | --- | --- | --- | --- | --- | --- | --- | --- | --- | --- | --- | --- | --- | --- | --- | --- | --- | --- | --- | --- | --- | --- | --- | --- | --- | --- | --- | --- | --- | --- | --- | --- | --- | --- | --- | --- | --- | --- | --- | --- | --- | --- | --- | --- | --- | --- | --- | --- | --- | --- | --- | --- | --- | --- | --- | --- | --- | --- | --- | --- |
| |  | | --- | |

50.

| | | | **Your Input:** **>PF3D7_1363200** | | |  |  |  |  |  |  |  |  |  | | --- | --- | --- | --- | --- | --- | --- | --- | --- | --- | --- | --- | |  | [PF13_0334](http://string-db.org/newstring_cgi/display_single_node.pl?taskId=_kNEuyOSlfcY&node=399241&targetmode=proteins) | polynucleotide kinase, putative (462 aa) | |  |  | *(Plasmodium falciparum)* | | **Predicted Functional Partners:** | | | |  | [PF11_0416](http://string-db.org/newstring_cgi/display_single_node.pl?taskId=_kNEuyOSlfcY&node=398807&targetmode=proteins) | myosin heavy chain subunit, putative (1600 aa) |  |  |  |  |  |  | [**•**](http://string-db.org/newstring_cgi/show_textmining_evidence.pl?taskId=_kNEuyOSlfcY&node2=398807) |  | 0.659 | |  | [PFE0175c](http://string-db.org/newstring_cgi/display_single_node.pl?taskId=_kNEuyOSlfcY&node=400993&targetmode=proteins) | unconventional myosin pfm-b (801 aa) |  |  |  |  |  |  | [**•**](http://string-db.org/newstring_cgi/show_textmining_evidence.pl?taskId=_kNEuyOSlfcY&node2=400993) |  | 0.655 | |  | [MyoE](http://string-db.org/newstring_cgi/display_single_node.pl?taskId=_kNEuyOSlfcY&node=401419&targetmode=proteins) | myosin-like protein, putative (2153 aa) |  |  |  |  |  |  | [**•**](http://string-db.org/newstring_cgi/show_textmining_evidence.pl?taskId=_kNEuyOSlfcY&node2=401419) |  | 0.649 | |  | [PF14_0125](http://string-db.org/newstring_cgi/display_single_node.pl?taskId=_kNEuyOSlfcY&node=399394&targetmode=proteins) | deoxyhypusine synthase (496 aa) |  |  |  | [**•**](http://string-db.org/newstring_cgi/show_coexpression_evidence.pl?taskId=_kNEuyOSlfcY&node2=399394) |  |  |  |  | 0.639 | |  | [PF14_0536](http://string-db.org/newstring_cgi/display_single_node.pl?taskId=_kNEuyOSlfcY&node=399815&targetmode=proteins) | hypothetical protein (415 aa) |  |  |  | [**•**](http://string-db.org/newstring_cgi/show_coexpression_evidence.pl?taskId=_kNEuyOSlfcY&node2=399815) |  |  |  |  | 0.633 | |  | [PF14_0052](http://string-db.org/newstring_cgi/display_single_node.pl?taskId=_kNEuyOSlfcY&node=399321&targetmode=proteins) | hypothetical protein, conserved (575 aa) |  |  |  | [**•**](http://string-db.org/newstring_cgi/show_coexpression_evidence.pl?taskId=_kNEuyOSlfcY&node2=399321) |  |  | [**•**](http://string-db.org/newstring_cgi/show_textmining_evidence.pl?taskId=_kNEuyOSlfcY&node2=399321) |  | 0.631 | |  | [PFF1480w](http://string-db.org/newstring_cgi/display_single_node.pl?taskId=_kNEuyOSlfcY&node=401584&targetmode=proteins) | microtubule-associated protein ytm1 homologue, putative (498 aa) |  |  |  | [**•**](http://string-db.org/newstring_cgi/show_coexpression_evidence.pl?taskId=_kNEuyOSlfcY&node2=401584) |  |  |  |  | 0.587 | |  | [PFE1535w](http://string-db.org/newstring_cgi/display_single_node.pl?taskId=_kNEuyOSlfcY&node=401265&targetmode=proteins) | hypothetical protein, conserved (1083 aa) |  |  |  | [**•**](http://string-db.org/newstring_cgi/show_coexpression_evidence.pl?taskId=_kNEuyOSlfcY&node2=401265) |  |  |  |  | 0.547 | |  | [gS](http://string-db.org/newstring_cgi/display_single_node.pl?taskId=_kNEuyOSlfcY&node=401080&targetmode=proteins) | glutathione synthetase (655 aa) |  |  |  | [**•**](http://string-db.org/newstring_cgi/show_coexpression_evidence.pl?taskId=_kNEuyOSlfcY&node2=401080) |  |  |  |  | 0.547 | |  | [PFA_0530c](http://string-db.org/newstring_cgi/display_single_node.pl?taskId=_kNEuyOSlfcY&node=400180&targetmode=proteins) | adenylate kinase, putative (186 aa) |  |  |  | [**•**](http://string-db.org/newstring_cgi/show_coexpression_evidence.pl?taskId=_kNEuyOSlfcY&node2=400180) |  |  |  |  | 0.547 | | | --- | --- | --- | --- | --- | --- | --- | --- | --- | --- | --- | --- | --- | --- | --- | --- | --- | --- | --- | --- | --- | --- | --- | --- | --- | --- | --- | --- | --- | --- | --- | --- | --- | --- | --- | --- | --- | --- | --- | --- | --- | --- | --- | --- | --- | --- | --- | --- | --- | --- | --- | --- | --- | --- | --- | --- | --- | --- | --- | --- | --- | --- | --- | --- | --- | --- | --- | --- | --- | --- | --- | --- | --- | --- | --- | --- | --- | --- | --- | --- | --- | --- | --- | --- | --- | --- | --- | --- | --- | --- | --- | --- | --- | --- | --- | --- | --- | --- | --- | --- | --- | --- | --- | --- | --- | --- | --- | --- | --- | --- | --- | --- | --- | --- | --- | --- | --- | --- | --- | --- | --- | --- | --- | --- | --- | --- | --- | --- | --- | --- | --- | --- | --- | --- | --- | --- | --- | --- | --- | --- | --- | --- | | | --- | --- | --- | --- | --- | --- | --- | --- | --- | --- | --- | --- | --- | --- | --- | --- | --- | --- | --- | --- | --- | --- | --- | --- | --- | --- | --- | --- | --- | --- | --- | --- | --- | --- | --- | --- | --- | --- | --- | --- | --- | --- | --- | --- | --- | --- | --- | --- | --- | --- | --- | --- | --- | --- | --- | --- | --- | --- | --- | --- | --- | --- | --- | --- | --- | --- | --- | --- | --- | --- | --- | --- | --- | --- | --- | --- | --- | --- | --- | --- | --- | --- | --- | --- | --- | --- | --- | --- | --- | --- | --- | --- | --- | --- | --- | --- | --- | --- | --- | --- | --- | --- | --- | --- | --- | --- | --- | --- | --- | --- | --- | --- | --- | --- | --- | --- | --- | --- | --- | --- | --- | --- | --- | --- | --- | --- | --- | --- | --- | --- | --- | --- | --- | --- | --- | --- | --- | --- | --- | --- | --- | --- | --- | |
| --- | --- | --- | --- | --- | --- | --- | --- | --- | --- | --- | --- | --- | --- | --- | --- | --- | --- | --- | --- | --- | --- | --- | --- | --- | --- | --- | --- | --- | --- | --- | --- | --- | --- | --- | --- | --- | --- | --- | --- | --- | --- | --- | --- | --- | --- | --- | --- | --- | --- | --- | --- | --- | --- | --- | --- | --- | --- | --- | --- | --- | --- | --- | --- | --- | --- | --- | --- | --- | --- | --- | --- | --- | --- | --- | --- | --- | --- | --- | --- | --- | --- | --- | --- | --- | --- | --- | --- | --- | --- | --- | --- | --- | --- | --- | --- | --- | --- | --- | --- | --- | --- | --- | --- | --- | --- | --- | --- | --- | --- | --- | --- | --- | --- | --- | --- | --- | --- | --- | --- | --- | --- | --- | --- | --- | --- | --- | --- | --- | --- | --- | --- | --- | --- | --- | --- | --- | --- | --- | --- | --- | --- | --- | --- |
| |  | | --- | |

51.

| | | | **Your Input:** **>PF3D7_1363500** | | |  |  |  |  |  |  |  |  |  | | --- | --- | --- | --- | --- | --- | --- | --- | --- | --- | --- | --- | |  | [PF13_0336](http://string-db.org/newstring_cgi/display_single_node.pl?taskId=Tx9qAYJr9gC8&node=399243&targetmode=proteins) | hypothetical protein, conserved (836 aa) | |  |  | *(Plasmodium falciparum)* | | **Predicted Functional Partners:** | | | |  | [PFL0680c](http://string-db.org/newstring_cgi/display_single_node.pl?taskId=Tx9qAYJr9gC8&node=402123&targetmode=proteins) | hypothetical protein, conserved (146 aa) |  |  |  | [**•**](http://string-db.org/newstring_cgi/show_coexpression_evidence.pl?taskId=Tx9qAYJr9gC8&node2=402123) |  |  |  |  | 0.547 | |  | [PF10_0316](http://string-db.org/newstring_cgi/display_single_node.pl?taskId=Tx9qAYJr9gC8&node=398310&targetmode=proteins) | phosphatidyl inositol glycan, class A, putative (497 aa) |  |  |  | [**•**](http://string-db.org/newstring_cgi/show_coexpression_evidence.pl?taskId=Tx9qAYJr9gC8&node2=398310) |  |  |  |  | 0.547 | |  | [PF13_0254](http://string-db.org/newstring_cgi/display_single_node.pl?taskId=Tx9qAYJr9gC8&node=399166&targetmode=proteins) | hypothetical protein, conserved (3855 aa) |  |  |  | [**•**](http://string-db.org/newstring_cgi/show_coexpression_evidence.pl?taskId=Tx9qAYJr9gC8&node2=399166) |  |  |  |  | 0.441 | | | --- | --- | --- | --- | --- | --- | --- | --- | --- | --- | --- | --- | --- | --- | --- | --- | --- | --- | --- | --- | --- | --- | --- | --- | --- | --- | --- | --- | --- | --- | --- | --- | --- | --- | --- | --- | --- | --- | --- | --- | --- | --- | --- | --- | --- | --- | --- | --- | --- | --- | --- | --- | --- | --- | --- | --- | --- | --- | | | --- | --- | --- | --- | --- | --- | --- | --- | --- | --- | --- | --- | --- | --- | --- | --- | --- | --- | --- | --- | --- | --- | --- | --- | --- | --- | --- | --- | --- | --- | --- | --- | --- | --- | --- | --- | --- | --- | --- | --- | --- | --- | --- | --- | --- | --- | --- | --- | --- | --- | --- | --- | --- | --- | --- | --- | --- | --- | --- | |
| --- | --- | --- | --- | --- | --- | --- | --- | --- | --- | --- | --- | --- | --- | --- | --- | --- | --- | --- | --- | --- | --- | --- | --- | --- | --- | --- | --- | --- | --- | --- | --- | --- | --- | --- | --- | --- | --- | --- | --- | --- | --- | --- | --- | --- | --- | --- | --- | --- | --- | --- | --- | --- | --- | --- | --- | --- | --- | --- | --- |
| |  | | --- | |

52.

| | | | **Your Input:** **>PF3D7_1403900** | | |  |  |  |  |  |  |  |  |  | | --- | --- | --- | --- | --- | --- | --- | --- | --- | --- | --- | --- | |  | [PF14_0036](http://string-db.org/newstring_cgi/display_single_node.pl?taskId=sYOqgHKS356k&node=399305&targetmode=proteins) | acid phosphatase, putative (302 aa) | |  |  | *(Plasmodium falciparum)* | | **Predicted Functional Partners:** | | | |  | [PF13_0222](http://string-db.org/newstring_cgi/display_single_node.pl?taskId=sYOqgHKS356k&node=399135&targetmode=proteins) | RNA lariat debranching enzyme, putative (575 aa) |  |  |  | [**•**](http://string-db.org/newstring_cgi/show_coexpression_evidence.pl?taskId=sYOqgHKS356k&node2=399135) |  |  | [**•**](http://string-db.org/newstring_cgi/show_textmining_evidence.pl?taskId=sYOqgHKS356k&node2=399135) |  | 0.697 | |  | [PFL0305c](http://string-db.org/newstring_cgi/display_single_node.pl?taskId=sYOqgHKS356k&node=402046&targetmode=proteins) | hypothetical protein, conserved (449 aa) |  |  |  |  |  |  | [**•**](http://string-db.org/newstring_cgi/show_textmining_evidence.pl?taskId=sYOqgHKS356k&node2=402046) |  | 0.688 | |  | [MAL8P1.202](http://string-db.org/newstring_cgi/display_single_node.pl?taskId=sYOqgHKS356k&node=397594&targetmode=proteins) | hypothetical protein, conserved (302 aa) |  |  |  |  |  |  | [**•**](http://string-db.org/newstring_cgi/show_textmining_evidence.pl?taskId=sYOqgHKS356k&node2=397594) |  | 0.688 | |  | [PFI1220w-1](http://string-db.org/newstring_cgi/display_single_node.pl?taskId=sYOqgHKS356k&node=401857&targetmode=proteins) | hypothetical protein, conserved (170 aa) |  |  |  |  |  |  | [**•**](http://string-db.org/newstring_cgi/show_textmining_evidence.pl?taskId=sYOqgHKS356k&node2=401857) |  | 0.687 | |  | [MAL7P1.339](http://string-db.org/newstring_cgi/display_single_node.pl?taskId=sYOqgHKS356k&node=397472&targetmode=proteins) | Ca%2B%2B chelating serine protease, putative (165 aa) |  |  |  |  |  |  | [**•**](http://string-db.org/newstring_cgi/show_textmining_evidence.pl?taskId=sYOqgHKS356k&node2=397472) |  | 0.687 | |  | [PF14_0662](http://string-db.org/newstring_cgi/display_single_node.pl?taskId=sYOqgHKS356k&node=399946&targetmode=proteins) | hypothetical protein (437 aa) |  |  |  | [**•**](http://string-db.org/newstring_cgi/show_coexpression_evidence.pl?taskId=sYOqgHKS356k&node2=399946) |  |  | [**•**](http://string-db.org/newstring_cgi/show_textmining_evidence.pl?taskId=sYOqgHKS356k&node2=399946) |  | 0.683 | |  | [PF14_0614](http://string-db.org/newstring_cgi/display_single_node.pl?taskId=sYOqgHKS356k&node=399896&targetmode=proteins) | hypothetical protein (1502 aa) |  |  |  |  |  |  | [**•**](http://string-db.org/newstring_cgi/show_textmining_evidence.pl?taskId=sYOqgHKS356k&node2=399896) |  | 0.683 | |  | [PF14_0064](http://string-db.org/newstring_cgi/display_single_node.pl?taskId=sYOqgHKS356k&node=399333&targetmode=proteins) | vacuolar protein sorting 29, putative (194 aa) |  |  |  |  |  |  | [**•**](http://string-db.org/newstring_cgi/show_textmining_evidence.pl?taskId=sYOqgHKS356k&node2=399333) |  | 0.683 | |  | [PFL0300c](http://string-db.org/newstring_cgi/display_single_node.pl?taskId=sYOqgHKS356k&node=402045&targetmode=proteins) | phosphoesterase, putative (304 aa) |  |  |  |  |  |  | [**•**](http://string-db.org/newstring_cgi/show_textmining_evidence.pl?taskId=sYOqgHKS356k&node2=402045) |  | 0.681 | |  | [PF14_0660](http://string-db.org/newstring_cgi/display_single_node.pl?taskId=sYOqgHKS356k&node=399944&targetmode=proteins) | hypothetical protein (358 aa) |  |  |  |  |  |  | [**•**](http://string-db.org/newstring_cgi/show_textmining_evidence.pl?taskId=sYOqgHKS356k&node2=399944) |  | 0.681 | |  | [PFC0595c](http://string-db.org/newstring_cgi/display_single_node.pl?taskId=sYOqgHKS356k&node=400594&targetmode=proteins) | serine%2Fthreonine protein phosphatase, putative (308 aa) |  |  |  |  |  |  | [**•**](http://string-db.org/newstring_cgi/show_textmining_evidence.pl?taskId=sYOqgHKS356k&node2=400594) |  | 0.643 | |  | [PFI1360c](http://string-db.org/newstring_cgi/display_single_node.pl?taskId=sYOqgHKS356k&node=401886&targetmode=proteins) | serine%2Fthreonine protein phosphatase, putative (312 aa) |  |  |  |  |  |  | [**•**](http://string-db.org/newstring_cgi/show_textmining_evidence.pl?taskId=sYOqgHKS356k&node2=401886) |  | 0.641 | |  | [PF08_0129](http://string-db.org/newstring_cgi/display_single_node.pl?taskId=sYOqgHKS356k&node=397966&targetmode=proteins) | protein phosphatase, putative (604 aa) |  |  |  |  |  |  | [**•**](http://string-db.org/newstring_cgi/show_textmining_evidence.pl?taskId=sYOqgHKS356k&node2=397966) |  | 0.641 | |  | [PP1](http://string-db.org/newstring_cgi/display_single_node.pl?taskId=sYOqgHKS356k&node=399412&targetmode=proteins) | serine%2Fthreonine protein phosphatase, putative (304 aa) |  |  |  |  |  |  | [**•**](http://string-db.org/newstring_cgi/show_textmining_evidence.pl?taskId=sYOqgHKS356k&node2=399412) |  | 0.589 | |  | [PFI1245c](http://string-db.org/newstring_cgi/display_single_node.pl?taskId=sYOqgHKS356k&node=401862&targetmode=proteins) | Protein phosphatase-beta (466 aa) |  |  |  |  |  |  | [**•**](http://string-db.org/newstring_cgi/show_textmining_evidence.pl?taskId=sYOqgHKS356k&node2=401862) |  | 0.583 | |  | [PP7](http://string-db.org/newstring_cgi/display_single_node.pl?taskId=sYOqgHKS356k&node=399495&targetmode=proteins) | PP1-like protein serine%2Fthreonine phosphatase (959 aa) |  |  |  |  |  |  | [**•**](http://string-db.org/newstring_cgi/show_textmining_evidence.pl?taskId=sYOqgHKS356k&node2=399495) |  | 0.583 | |  | [PF14_0630](http://string-db.org/newstring_cgi/display_single_node.pl?taskId=sYOqgHKS356k&node=399913&targetmode=proteins) | protein serine%2Fthreonine phosphatase (889 aa) |  |  |  |  |  |  | [**•**](http://string-db.org/newstring_cgi/show_textmining_evidence.pl?taskId=sYOqgHKS356k&node2=399913) |  | 0.581 | |  | [PfPP5](http://string-db.org/newstring_cgi/display_single_node.pl?taskId=sYOqgHKS356k&node=397148&targetmode=proteins) | serine%2Fthreonine protein phosphatase pfPp5 (658 aa) |  |  |  |  |  |  | [**•**](http://string-db.org/newstring_cgi/show_textmining_evidence.pl?taskId=sYOqgHKS356k&node2=397148) |  | 0.501 | |  | [PFI1216w](http://string-db.org/newstring_cgi/display_single_node.pl?taskId=sYOqgHKS356k&node=401856&targetmode=proteins) | telomeric repeat binding factor 1 (101 aa) |  |  |  |  |  |  | [**•**](http://string-db.org/newstring_cgi/show_textmining_evidence.pl?taskId=sYOqgHKS356k&node2=401856) |  | 0.419 | |  | [PFF0573c](http://string-db.org/newstring_cgi/display_single_node.pl?taskId=sYOqgHKS356k&node=401398&targetmode=proteins) | 60S ribosomal protein L39, putative (62 aa) |  |  |  |  |  |  | [**•**](http://string-db.org/newstring_cgi/show_textmining_evidence.pl?taskId=sYOqgHKS356k&node2=401398) |  | 0.419 | | | --- | --- | --- | --- | --- | --- | --- | --- | --- | --- | --- | --- | --- | --- | --- | --- | --- | --- | --- | --- | --- | --- | --- | --- | --- | --- | --- | --- | --- | --- | --- | --- | --- | --- | --- | --- | --- | --- | --- | --- | --- | --- | --- | --- | --- | --- | --- | --- | --- | --- | --- | --- | --- | --- | --- | --- | --- | --- | --- | --- | --- | --- | --- | --- | --- | --- | --- | --- | --- | --- | --- | --- | --- | --- | --- | --- | --- | --- | --- | --- | --- | --- | --- | --- | --- | --- | --- | --- | --- | --- | --- | --- | --- | --- | --- | --- | --- | --- | --- | --- | --- | --- | --- | --- | --- | --- | --- | --- | --- | --- | --- | --- | --- | --- | --- | --- | --- | --- | --- | --- | --- | --- | --- | --- | --- | --- | --- | --- | --- | --- | --- | --- | --- | --- | --- | --- | --- | --- | --- | --- | --- | --- | --- | --- | --- | --- | --- | --- | --- | --- | --- | --- | --- | --- | --- | --- | --- | --- | --- | --- | --- | --- | --- | --- | --- | --- | --- | --- | --- | --- | --- | --- | --- | --- | --- | --- | --- | --- | --- | --- | --- | --- | --- | --- | --- | --- | --- | --- | --- | --- | --- | --- | --- | --- | --- | --- | --- | --- | --- | --- | --- | --- | --- | --- | --- | --- | --- | --- | --- | --- | --- | --- | --- | --- | --- | --- | --- | --- | --- | --- | --- | --- | --- | --- | --- | --- | --- | --- | --- | --- | --- | --- | --- | --- | --- | --- | --- | --- | --- | --- | --- | --- | --- | --- | --- | --- | --- | --- | --- | --- | --- | --- | --- | --- | --- | --- | --- | --- | --- | --- | --- | --- | | | --- | --- | --- | --- | --- | --- | --- | --- | --- | --- | --- | --- | --- | --- | --- | --- | --- | --- | --- | --- | --- | --- | --- | --- | --- | --- | --- | --- | --- | --- | --- | --- | --- | --- | --- | --- | --- | --- | --- | --- | --- | --- | --- | --- | --- | --- | --- | --- | --- | --- | --- | --- | --- | --- | --- | --- | --- | --- | --- | --- | --- | --- | --- | --- | --- | --- | --- | --- | --- | --- | --- | --- | --- | --- | --- | --- | --- | --- | --- | --- | --- | --- | --- | --- | --- | --- | --- | --- | --- | --- | --- | --- | --- | --- | --- | --- | --- | --- | --- | --- | --- | --- | --- | --- | --- | --- | --- | --- | --- | --- | --- | --- | --- | --- | --- | --- | --- | --- | --- | --- | --- | --- | --- | --- | --- | --- | --- | --- | --- | --- | --- | --- | --- | --- | --- | --- | --- | --- | --- | --- | --- | --- | --- | --- | --- | --- | --- | --- | --- | --- | --- | --- | --- | --- | --- | --- | --- | --- | --- | --- | --- | --- | --- | --- | --- | --- | --- | --- | --- | --- | --- | --- | --- | --- | --- | --- | --- | --- | --- | --- | --- | --- | --- | --- | --- | --- | --- | --- | --- | --- | --- | --- | --- | --- | --- | --- | --- | --- | --- | --- | --- | --- | --- | --- | --- | --- | --- | --- | --- | --- | --- | --- | --- | --- | --- | --- | --- | --- | --- | --- | --- | --- | --- | --- | --- | --- | --- | --- | --- | --- | --- | --- | --- | --- | --- | --- | --- | --- | --- | --- | --- | --- | --- | --- | --- | --- | --- | --- | --- | --- | --- | --- | --- | --- | --- | --- | --- | --- | --- | --- | --- | --- | --- | |
| --- | --- | --- | --- | --- | --- | --- | --- | --- | --- | --- | --- | --- | --- | --- | --- | --- | --- | --- | --- | --- | --- | --- | --- | --- | --- | --- | --- | --- | --- | --- | --- | --- | --- | --- | --- | --- | --- | --- | --- | --- | --- | --- | --- | --- | --- | --- | --- | --- | --- | --- | --- | --- | --- | --- | --- | --- | --- | --- | --- | --- | --- | --- | --- | --- | --- | --- | --- | --- | --- | --- | --- | --- | --- | --- | --- | --- | --- | --- | --- | --- | --- | --- | --- | --- | --- | --- | --- | --- | --- | --- | --- | --- | --- | --- | --- | --- | --- | --- | --- | --- | --- | --- | --- | --- | --- | --- | --- | --- | --- | --- | --- | --- | --- | --- | --- | --- | --- | --- | --- | --- | --- | --- | --- | --- | --- | --- | --- | --- | --- | --- | --- | --- | --- | --- | --- | --- | --- | --- | --- | --- | --- | --- | --- | --- | --- | --- | --- | --- | --- | --- | --- | --- | --- | --- | --- | --- | --- | --- | --- | --- | --- | --- | --- | --- | --- | --- | --- | --- | --- | --- | --- | --- | --- | --- | --- | --- | --- | --- | --- | --- | --- | --- | --- | --- | --- | --- | --- | --- | --- | --- | --- | --- | --- | --- | --- | --- | --- | --- | --- | --- | --- | --- | --- | --- | --- | --- | --- | --- | --- | --- | --- | --- | --- | --- | --- | --- | --- | --- | --- | --- | --- | --- | --- | --- | --- | --- | --- | --- | --- | --- | --- | --- | --- | --- | --- | --- | --- | --- | --- | --- | --- | --- | --- | --- | --- | --- | --- | --- | --- | --- | --- | --- | --- | --- | --- | --- | --- | --- | --- | --- | --- | --- | --- |
| |  | | --- | |

53

| | | | **Your Input:** **>PF3D7_1406700** | | |  |  |  |  |  |  |  |  |  | | --- | --- | --- | --- | --- | --- | --- | --- | --- | --- | --- | --- | |  | [PF14_0064](http://string-db.org/newstring_cgi/display_single_node.pl?taskId=Mgu13pskn5zG&node=399333&targetmode=proteins) | vacuolar protein sorting 29, putative (194 aa) | |  |  | *(Plasmodium falciparum)* | | **Predicted Functional Partners:** | | | |  | [PFL2415w](http://string-db.org/newstring_cgi/display_single_node.pl?taskId=Mgu13pskn5zG&node=402478&targetmode=proteins) | Hbeta58%2FVps26 protein homolog, putative; May play a role in vesicular protein sorting, simila [...] (297 aa) |  |  |  | [**•**](http://string-db.org/newstring_cgi/show_coexpression_evidence.pl?taskId=Mgu13pskn5zG&node2=402478) | [**•**](http://string-db.org/newstring_cgi/show_set_evidence.pl?data_channel=experimental&taskId=Mgu13pskn5zG&node2=402478) |  | [**•**](http://string-db.org/newstring_cgi/show_textmining_evidence.pl?taskId=Mgu13pskn5zG&node2=402478) |  | 0.900 | |  | [PF14_0462](http://string-db.org/newstring_cgi/display_single_node.pl?taskId=Mgu13pskn5zG&node=399738&targetmode=proteins) | SEL-1 protein, putative (851 aa) |  |  |  | [**•**](http://string-db.org/newstring_cgi/show_coexpression_evidence.pl?taskId=Mgu13pskn5zG&node2=399738) |  |  |  |  | 0.768 | |  | [MAL7P1.110](http://string-db.org/newstring_cgi/display_single_node.pl?taskId=Mgu13pskn5zG&node=397350&targetmode=proteins) | Ham1-like protein, putative (198 aa) | [**•**](http://string-db.org/newstring_cgi/show_neighborhood.pl?taskId=Mgu13pskn5zG&node2=397350) |  |  | [**•**](http://string-db.org/newstring_cgi/show_coexpression_evidence.pl?taskId=Mgu13pskn5zG&node2=397350) |  |  |  |  | 0.743 | |  | [PFI1360c](http://string-db.org/newstring_cgi/display_single_node.pl?taskId=Mgu13pskn5zG&node=401886&targetmode=proteins) | serine%2Fthreonine protein phosphatase, putative (312 aa) |  |  |  | [**•**](http://string-db.org/newstring_cgi/show_coexpression_evidence.pl?taskId=Mgu13pskn5zG&node2=401886) |  |  | [**•**](http://string-db.org/newstring_cgi/show_textmining_evidence.pl?taskId=Mgu13pskn5zG&node2=401886) |  | 0.739 | |  | [PP1](http://string-db.org/newstring_cgi/display_single_node.pl?taskId=Mgu13pskn5zG&node=399412&targetmode=proteins) | serine%2Fthreonine protein phosphatase, putative (304 aa) |  |  |  | [**•**](http://string-db.org/newstring_cgi/show_coexpression_evidence.pl?taskId=Mgu13pskn5zG&node2=399412) |  |  | [**•**](http://string-db.org/newstring_cgi/show_textmining_evidence.pl?taskId=Mgu13pskn5zG&node2=399412) |  | 0.693 | |  | [PFL0300c](http://string-db.org/newstring_cgi/display_single_node.pl?taskId=Mgu13pskn5zG&node=402045&targetmode=proteins) | phosphoesterase, putative (304 aa) |  |  |  |  |  |  | [**•**](http://string-db.org/newstring_cgi/show_textmining_evidence.pl?taskId=Mgu13pskn5zG&node2=402045) |  | 0.688 | |  | [PF14_0660](http://string-db.org/newstring_cgi/display_single_node.pl?taskId=Mgu13pskn5zG&node=399944&targetmode=proteins) | hypothetical protein (358 aa) |  |  |  |  |  |  | [**•**](http://string-db.org/newstring_cgi/show_textmining_evidence.pl?taskId=Mgu13pskn5zG&node2=399944) |  | 0.688 | |  | [PF14_0614](http://string-db.org/newstring_cgi/display_single_node.pl?taskId=Mgu13pskn5zG&node=399896&targetmode=proteins) | hypothetical protein (1502 aa) |  |  |  |  |  |  | [**•**](http://string-db.org/newstring_cgi/show_textmining_evidence.pl?taskId=Mgu13pskn5zG&node2=399896) |  | 0.688 | |  | [PF13_0222](http://string-db.org/newstring_cgi/display_single_node.pl?taskId=Mgu13pskn5zG&node=399135&targetmode=proteins) | RNA lariat debranching enzyme, putative (575 aa) |  |  |  |  |  |  | [**•**](http://string-db.org/newstring_cgi/show_textmining_evidence.pl?taskId=Mgu13pskn5zG&node2=399135) |  | 0.688 | |  | [PF14_0036](http://string-db.org/newstring_cgi/display_single_node.pl?taskId=Mgu13pskn5zG&node=399305&targetmode=proteins) | acid phosphatase, putative (302 aa) |  |  |  |  |  |  | [**•**](http://string-db.org/newstring_cgi/show_textmining_evidence.pl?taskId=Mgu13pskn5zG&node2=399305) |  | 0.683 | | | --- | --- | --- | --- | --- | --- | --- | --- | --- | --- | --- | --- | --- | --- | --- | --- | --- | --- | --- | --- | --- | --- | --- | --- | --- | --- | --- | --- | --- | --- | --- | --- | --- | --- | --- | --- | --- | --- | --- | --- | --- | --- | --- | --- | --- | --- | --- | --- | --- | --- | --- | --- | --- | --- | --- | --- | --- | --- | --- | --- | --- | --- | --- | --- | --- | --- | --- | --- | --- | --- | --- | --- | --- | --- | --- | --- | --- | --- | --- | --- | --- | --- | --- | --- | --- | --- | --- | --- | --- | --- | --- | --- | --- | --- | --- | --- | --- | --- | --- | --- | --- | --- | --- | --- | --- | --- | --- | --- | --- | --- | --- | --- | --- | --- | --- | --- | --- | --- | --- | --- | --- | --- | --- | --- | --- | --- | --- | --- | --- | --- | --- | --- | --- | --- | --- | --- | --- | --- | --- | --- | --- | --- | | | --- | --- | --- | --- | --- | --- | --- | --- | --- | --- | --- | --- | --- | --- | --- | --- | --- | --- | --- | --- | --- | --- | --- | --- | --- | --- | --- | --- | --- | --- | --- | --- | --- | --- | --- | --- | --- | --- | --- | --- | --- | --- | --- | --- | --- | --- | --- | --- | --- | --- | --- | --- | --- | --- | --- | --- | --- | --- | --- | --- | --- | --- | --- | --- | --- | --- | --- | --- | --- | --- | --- | --- | --- | --- | --- | --- | --- | --- | --- | --- | --- | --- | --- | --- | --- | --- | --- | --- | --- | --- | --- | --- | --- | --- | --- | --- | --- | --- | --- | --- | --- | --- | --- | --- | --- | --- | --- | --- | --- | --- | --- | --- | --- | --- | --- | --- | --- | --- | --- | --- | --- | --- | --- | --- | --- | --- | --- | --- | --- | --- | --- | --- | --- | --- | --- | --- | --- | --- | --- | --- | --- | --- | --- | |
| --- | --- | --- | --- | --- | --- | --- | --- | --- | --- | --- | --- | --- | --- | --- | --- | --- | --- | --- | --- | --- | --- | --- | --- | --- | --- | --- | --- | --- | --- | --- | --- | --- | --- | --- | --- | --- | --- | --- | --- | --- | --- | --- | --- | --- | --- | --- | --- | --- | --- | --- | --- | --- | --- | --- | --- | --- | --- | --- | --- | --- | --- | --- | --- | --- | --- | --- | --- | --- | --- | --- | --- | --- | --- | --- | --- | --- | --- | --- | --- | --- | --- | --- | --- | --- | --- | --- | --- | --- | --- | --- | --- | --- | --- | --- | --- | --- | --- | --- | --- | --- | --- | --- | --- | --- | --- | --- | --- | --- | --- | --- | --- | --- | --- | --- | --- | --- | --- | --- | --- | --- | --- | --- | --- | --- | --- | --- | --- | --- | --- | --- | --- | --- | --- | --- | --- | --- | --- | --- | --- | --- | --- | --- | --- |
| |  | | --- | |

54.

| | | | **Your Input:** **>PF3D7_1414400** | | |  |  |  |  |  |  |  |  |  | | --- | --- | --- | --- | --- | --- | --- | --- | --- | --- | --- | --- | |  | [PP1](http://string-db.org/newstring_cgi/display_single_node.pl?taskId=yI35rEpnqtus&node=399412&targetmode=proteins) | serine%2Fthreonine protein phosphatase, putative (304 aa) | |  |  | *(Plasmodium falciparum)* | | **Predicted Functional Partners:** | | | |  | [GAP50](http://string-db.org/newstring_cgi/display_single_node.pl?taskId=yI35rEpnqtus&node=401788&targetmode=proteins) | acid phosphatase, putative (396 aa) |  |  |  | [**•**](http://string-db.org/newstring_cgi/show_coexpression_evidence.pl?taskId=yI35rEpnqtus&node2=401788) |  |  | [**•**](http://string-db.org/newstring_cgi/show_textmining_evidence.pl?taskId=yI35rEpnqtus&node2=401788) |  | 0.840 | |  | [PFE1345c](http://string-db.org/newstring_cgi/display_single_node.pl?taskId=yI35rEpnqtus&node=401229&targetmode=proteins) | minichromosome maintenance protein 3, putative (962 aa) |  |  |  | [**•**](http://string-db.org/newstring_cgi/show_coexpression_evidence.pl?taskId=yI35rEpnqtus&node2=401229) |  |  |  |  | 0.806 | |  | [PF10_0020](http://string-db.org/newstring_cgi/display_single_node.pl?taskId=yI35rEpnqtus&node=397998&targetmode=proteins) | hypothetical protein (763 aa) |  |  |  | [**•**](http://string-db.org/newstring_cgi/show_coexpression_evidence.pl?taskId=yI35rEpnqtus&node2=397998) |  |  |  |  | 0.806 | |  | [MAL8P1.53](http://string-db.org/newstring_cgi/display_single_node.pl?taskId=yI35rEpnqtus&node=397653&targetmode=proteins) | hypothetical protein, conserved (514 aa) |  |  |  | [**•**](http://string-db.org/newstring_cgi/show_coexpression_evidence.pl?taskId=yI35rEpnqtus&node2=397653) |  |  |  |  | 0.744 | |  | [PFL0300c](http://string-db.org/newstring_cgi/display_single_node.pl?taskId=yI35rEpnqtus&node=402045&targetmode=proteins) | phosphoesterase, putative (304 aa) |  |  |  | [**•**](http://string-db.org/newstring_cgi/show_coexpression_evidence.pl?taskId=yI35rEpnqtus&node2=402045) |  |  | [**•**](http://string-db.org/newstring_cgi/show_textmining_evidence.pl?taskId=yI35rEpnqtus&node2=402045) |  | 0.732 | |  | [PFF0670w-1](http://string-db.org/newstring_cgi/display_single_node.pl?taskId=yI35rEpnqtus&node=401418&targetmode=proteins) | hypothetical protein, conserved (4095 aa) |  |  |  | [**•**](http://string-db.org/newstring_cgi/show_coexpression_evidence.pl?taskId=yI35rEpnqtus&node2=401418) |  |  |  |  | 0.732 | |  | [PF10_0337](http://string-db.org/newstring_cgi/display_single_node.pl?taskId=yI35rEpnqtus&node=398333&targetmode=proteins) | ADP-ribosylation factor-like protein (178 aa) |  |  |  | [**•**](http://string-db.org/newstring_cgi/show_coexpression_evidence.pl?taskId=yI35rEpnqtus&node2=398333) |  |  |  |  | 0.724 | |  | [PFL2215w](http://string-db.org/newstring_cgi/display_single_node.pl?taskId=yI35rEpnqtus&node=402438&targetmode=proteins) | actin; Actins are highly conserved proteins that are involved in various types of cell motility [...] (376 aa) |  |  |  | [**•**](http://string-db.org/newstring_cgi/show_coexpression_evidence.pl?taskId=yI35rEpnqtus&node2=402438) |  |  | [**•**](http://string-db.org/newstring_cgi/show_textmining_evidence.pl?taskId=yI35rEpnqtus&node2=402438) |  | 0.717 | |  | [Pfn](http://string-db.org/newstring_cgi/display_single_node.pl?taskId=yI35rEpnqtus&node=401928&targetmode=proteins) | conserved protein; Essential for the invasive blood stages of the parasite. Binds to proline ri [...] (171 aa) |  |  |  | [**•**](http://string-db.org/newstring_cgi/show_coexpression_evidence.pl?taskId=yI35rEpnqtus&node2=401928) |  |  |  |  | 0.708 | |  | [PFC0475c](http://string-db.org/newstring_cgi/display_single_node.pl?taskId=yI35rEpnqtus&node=400566&targetmode=proteins) | hypothetical protein, conserved (953 aa) |  |  |  | [**•**](http://string-db.org/newstring_cgi/show_coexpression_evidence.pl?taskId=yI35rEpnqtus&node2=400566) |  |  |  |  | 0.708 | | | --- | --- | --- | --- | --- | --- | --- | --- | --- | --- | --- | --- | --- | --- | --- | --- | --- | --- | --- | --- | --- | --- | --- | --- | --- | --- | --- | --- | --- | --- | --- | --- | --- | --- | --- | --- | --- | --- | --- | --- | --- | --- | --- | --- | --- | --- | --- | --- | --- | --- | --- | --- | --- | --- | --- | --- | --- | --- | --- | --- | --- | --- | --- | --- | --- | --- | --- | --- | --- | --- | --- | --- | --- | --- | --- | --- | --- | --- | --- | --- | --- | --- | --- | --- | --- | --- | --- | --- | --- | --- | --- | --- | --- | --- | --- | --- | --- | --- | --- | --- | --- | --- | --- | --- | --- | --- | --- | --- | --- | --- | --- | --- | --- | --- | --- | --- | --- | --- | --- | --- | --- | --- | --- | --- | --- | --- | --- | --- | --- | --- | --- | --- | --- | --- | --- | --- | --- | --- | --- | --- | --- | --- | | | --- | --- | --- | --- | --- | --- | --- | --- | --- | --- | --- | --- | --- | --- | --- | --- | --- | --- | --- | --- | --- | --- | --- | --- | --- | --- | --- | --- | --- | --- | --- | --- | --- | --- | --- | --- | --- | --- | --- | --- | --- | --- | --- | --- | --- | --- | --- | --- | --- | --- | --- | --- | --- | --- | --- | --- | --- | --- | --- | --- | --- | --- | --- | --- | --- | --- | --- | --- | --- | --- | --- | --- | --- | --- | --- | --- | --- | --- | --- | --- | --- | --- | --- | --- | --- | --- | --- | --- | --- | --- | --- | --- | --- | --- | --- | --- | --- | --- | --- | --- | --- | --- | --- | --- | --- | --- | --- | --- | --- | --- | --- | --- | --- | --- | --- | --- | --- | --- | --- | --- | --- | --- | --- | --- | --- | --- | --- | --- | --- | --- | --- | --- | --- | --- | --- | --- | --- | --- | --- | --- | --- | --- | --- | |
| --- | --- | --- | --- | --- | --- | --- | --- | --- | --- | --- | --- | --- | --- | --- | --- | --- | --- | --- | --- | --- | --- | --- | --- | --- | --- | --- | --- | --- | --- | --- | --- | --- | --- | --- | --- | --- | --- | --- | --- | --- | --- | --- | --- | --- | --- | --- | --- | --- | --- | --- | --- | --- | --- | --- | --- | --- | --- | --- | --- | --- | --- | --- | --- | --- | --- | --- | --- | --- | --- | --- | --- | --- | --- | --- | --- | --- | --- | --- | --- | --- | --- | --- | --- | --- | --- | --- | --- | --- | --- | --- | --- | --- | --- | --- | --- | --- | --- | --- | --- | --- | --- | --- | --- | --- | --- | --- | --- | --- | --- | --- | --- | --- | --- | --- | --- | --- | --- | --- | --- | --- | --- | --- | --- | --- | --- | --- | --- | --- | --- | --- | --- | --- | --- | --- | --- | --- | --- | --- | --- | --- | --- | --- | --- |
|  |

55.

| | | | **Your Input:** **>PF3D7_1423300** | | |  |  |  |  |  |  |  |  |  | | --- | --- | --- | --- | --- | --- | --- | --- | --- | --- | --- | --- | |  | [PP7](http://string-db.org/newstring_cgi/display_single_node.pl?taskId=UGuXufYU98MF&node=399495&targetmode=proteins) | PP1-like protein serine%2Fthreonine phosphatase (959 aa) | |  |  | *(Plasmodium falciparum)* | | **Predicted Functional Partners:** | | | |  | [PfPKAc](http://string-db.org/newstring_cgi/display_single_node.pl?taskId=UGuXufYU98MF&node=401954&targetmode=proteins) | cAMP-dependent protein kinase catalytic subunit (342 aa) |  |  |  | [**•**](http://string-db.org/newstring_cgi/show_coexpression_evidence.pl?taskId=UGuXufYU98MF&node2=401954) |  |  | [**•**](http://string-db.org/newstring_cgi/show_textmining_evidence.pl?taskId=UGuXufYU98MF&node2=401954) |  | 0.971 | |  | [PF10_0352](http://string-db.org/newstring_cgi/display_single_node.pl?taskId=UGuXufYU98MF&node=398348&targetmode=proteins) | merozoite surface protein, putative (405 aa) |  |  |  | [**•**](http://string-db.org/newstring_cgi/show_coexpression_evidence.pl?taskId=UGuXufYU98MF&node2=398348) |  |  | [**•**](http://string-db.org/newstring_cgi/show_textmining_evidence.pl?taskId=UGuXufYU98MF&node2=398348) |  | 0.963 | |  | [PF10_0344](http://string-db.org/newstring_cgi/display_single_node.pl?taskId=UGuXufYU98MF&node=398340&targetmode=proteins) | glutamate-rich protein (1233 aa) |  |  |  | [**•**](http://string-db.org/newstring_cgi/show_coexpression_evidence.pl?taskId=UGuXufYU98MF&node2=398340) |  |  | [**•**](http://string-db.org/newstring_cgi/show_textmining_evidence.pl?taskId=UGuXufYU98MF&node2=398340) |  | 0.961 | |  | [AMA1](http://string-db.org/newstring_cgi/display_single_node.pl?taskId=UGuXufYU98MF&node=398735&targetmode=proteins) | apical membrane antigen 1, AMA1; Involved in parasite invasion of erythrocytes (622 aa) |  |  |  | [**•**](http://string-db.org/newstring_cgi/show_coexpression_evidence.pl?taskId=UGuXufYU98MF&node2=398735) |  |  | [**•**](http://string-db.org/newstring_cgi/show_textmining_evidence.pl?taskId=UGuXufYU98MF&node2=398735) |  | 0.961 | |  | [PF14_0102](http://string-db.org/newstring_cgi/display_single_node.pl?taskId=UGuXufYU98MF&node=399371&targetmode=proteins) | rhoptry-associated protein 1, RAP1 (782 aa) |  |  |  | [**•**](http://string-db.org/newstring_cgi/show_coexpression_evidence.pl?taskId=UGuXufYU98MF&node2=399371) |  |  | [**•**](http://string-db.org/newstring_cgi/show_textmining_evidence.pl?taskId=UGuXufYU98MF&node2=399371) |  | 0.960 | |  | [PFL2460w](http://string-db.org/newstring_cgi/display_single_node.pl?taskId=UGuXufYU98MF&node=402487&targetmode=proteins) | coronin (602 aa) |  |  |  | [**•**](http://string-db.org/newstring_cgi/show_coexpression_evidence.pl?taskId=UGuXufYU98MF&node2=402487) |  |  |  |  | 0.960 | |  | [PFL1435c](http://string-db.org/newstring_cgi/display_single_node.pl?taskId=UGuXufYU98MF&node=402278&targetmode=proteins) | myosin d (2231 aa) |  |  |  | [**•**](http://string-db.org/newstring_cgi/show_coexpression_evidence.pl?taskId=UGuXufYU98MF&node2=402278) |  |  |  |  | 0.960 | |  | [PFI0540w](http://string-db.org/newstring_cgi/display_single_node.pl?taskId=UGuXufYU98MF&node=401719&targetmode=proteins) | hypothetical protein, conserved (1165 aa) |  |  |  | [**•**](http://string-db.org/newstring_cgi/show_coexpression_evidence.pl?taskId=UGuXufYU98MF&node2=401719) |  |  |  |  | 0.960 | |  | [PFI0410c](http://string-db.org/newstring_cgi/display_single_node.pl?taskId=UGuXufYU98MF&node=401693&targetmode=proteins) | hypothetical protein, conserved (2054 aa) |  |  |  | [**•**](http://string-db.org/newstring_cgi/show_coexpression_evidence.pl?taskId=UGuXufYU98MF&node2=401693) |  |  |  |  | 0.960 | |  | [PFI0175w](http://string-db.org/newstring_cgi/display_single_node.pl?taskId=UGuXufYU98MF&node=401644&targetmode=proteins) | hypothetical protein, conserved (742 aa) |  |  |  | [**•**](http://string-db.org/newstring_cgi/show_coexpression_evidence.pl?taskId=UGuXufYU98MF&node2=401644) |  |  |  |  | 0.960 | |  | [PFF1365c](http://string-db.org/newstring_cgi/display_single_node.pl?taskId=UGuXufYU98MF&node=401560&targetmode=proteins) | HECT-domain (ubiquitin-transferase), putative; Putative E3 ubiquitin-protein ligase (By similar [...] (10286 aa) |  |  |  | [**•**](http://string-db.org/newstring_cgi/show_coexpression_evidence.pl?taskId=UGuXufYU98MF&node2=401560) |  |  |  |  | 0.960 | |  | [PFF0870w](http://string-db.org/newstring_cgi/display_single_node.pl?taskId=UGuXufYU98MF&node=401460&targetmode=proteins) | hypothetical protein, conserved (795 aa) |  |  |  | [**•**](http://string-db.org/newstring_cgi/show_coexpression_evidence.pl?taskId=UGuXufYU98MF&node2=401460) |  |  |  |  | 0.960 | |  | [MyoE](http://string-db.org/newstring_cgi/display_single_node.pl?taskId=UGuXufYU98MF&node=401419&targetmode=proteins) | myosin-like protein, putative (2153 aa) |  |  |  | [**•**](http://string-db.org/newstring_cgi/show_coexpression_evidence.pl?taskId=UGuXufYU98MF&node2=401419) |  |  |  |  | 0.960 | |  | [PFF0645c](http://string-db.org/newstring_cgi/display_single_node.pl?taskId=UGuXufYU98MF&node=401413&targetmode=proteins) | integral membrane protein (1347 aa) |  |  |  | [**•**](http://string-db.org/newstring_cgi/show_coexpression_evidence.pl?taskId=UGuXufYU98MF&node2=401413) |  |  |  |  | 0.960 | |  | [RAP2](http://string-db.org/newstring_cgi/display_single_node.pl?taskId=UGuXufYU98MF&node=400974&targetmode=proteins) | rhoptry-associated protein 2, RAP2 (398 aa) |  |  |  | [**•**](http://string-db.org/newstring_cgi/show_coexpression_evidence.pl?taskId=UGuXufYU98MF&node2=400974) |  |  |  |  | 0.960 | |  | [PFD1100c](http://string-db.org/newstring_cgi/display_single_node.pl?taskId=UGuXufYU98MF&node=400929&targetmode=proteins) | hypothetical protein, conserved (476 aa) |  |  |  | [**•**](http://string-db.org/newstring_cgi/show_coexpression_evidence.pl?taskId=UGuXufYU98MF&node2=400929) |  |  |  |  | 0.960 | |  | [PFD0900w](http://string-db.org/newstring_cgi/display_single_node.pl?taskId=UGuXufYU98MF&node=400883&targetmode=proteins) | hypothetical protein, conserved (2011 aa) |  |  |  | [**•**](http://string-db.org/newstring_cgi/show_coexpression_evidence.pl?taskId=UGuXufYU98MF&node2=400883) |  |  |  |  | 0.960 | |  | [PFD0385w](http://string-db.org/newstring_cgi/display_single_node.pl?taskId=UGuXufYU98MF&node=400780&targetmode=proteins) | hypothetical protein, conserved (1960 aa) |  |  |  | [**•**](http://string-db.org/newstring_cgi/show_coexpression_evidence.pl?taskId=UGuXufYU98MF&node2=400780) |  |  |  |  | 0.960 | |  | [ASP](http://string-db.org/newstring_cgi/display_single_node.pl?taskId=UGuXufYU98MF&node=400760&targetmode=proteins) | apical sushi protein, ASP (731 aa) |  |  |  | [**•**](http://string-db.org/newstring_cgi/show_coexpression_evidence.pl?taskId=UGuXufYU98MF&node2=400760) |  |  |  |  | 0.960 | |  | [PFD0230c](http://string-db.org/newstring_cgi/display_single_node.pl?taskId=UGuXufYU98MF&node=400747&targetmode=proteins) | protease, putative (939 aa) |  |  |  | [**•**](http://string-db.org/newstring_cgi/show_coexpression_evidence.pl?taskId=UGuXufYU98MF&node2=400747) |  |  |  |  | 0.960 | |  | [PFC0820w](http://string-db.org/newstring_cgi/display_single_node.pl?taskId=UGuXufYU98MF&node=400636&targetmode=proteins) | hypothetical protein, conserved (4981 aa) |  |  |  | [**•**](http://string-db.org/newstring_cgi/show_coexpression_evidence.pl?taskId=UGuXufYU98MF&node2=400636) |  |  |  |  | 0.960 | |  | [PFC0355c](http://string-db.org/newstring_cgi/display_single_node.pl?taskId=UGuXufYU98MF&node=400538&targetmode=proteins) | hypothetical protein (337 aa) |  |  |  | [**•**](http://string-db.org/newstring_cgi/show_coexpression_evidence.pl?taskId=UGuXufYU98MF&node2=400538) |  |  |  |  | 0.960 | |  | [PFC0185w](http://string-db.org/newstring_cgi/display_single_node.pl?taskId=UGuXufYU98MF&node=400496&targetmode=proteins) | hypothetical protein, conserved (525 aa) |  |  |  | [**•**](http://string-db.org/newstring_cgi/show_coexpression_evidence.pl?taskId=UGuXufYU98MF&node2=400496) |  |  |  |  | 0.960 | |  | [RON6](http://string-db.org/newstring_cgi/display_single_node.pl?taskId=UGuXufYU98MF&node=400367&targetmode=proteins) | hypothetical protein (951 aa) |  |  |  | [**•**](http://string-db.org/newstring_cgi/show_coexpression_evidence.pl?taskId=UGuXufYU98MF&node2=400367) |  |  |  |  | 0.960 | |  | [PFB0475c](http://string-db.org/newstring_cgi/display_single_node.pl?taskId=UGuXufYU98MF&node=400326&targetmode=proteins) | hypothetical protein, conserved (446 aa) |  |  |  | [**•**](http://string-db.org/newstring_cgi/show_coexpression_evidence.pl?taskId=UGuXufYU98MF&node2=400326) |  |  |  |  | 0.960 | |  | [PFA_0440w](http://string-db.org/newstring_cgi/display_single_node.pl?taskId=UGuXufYU98MF&node=400162&targetmode=proteins) | hypothetical protein, conserved (224 aa) |  |  |  | [**•**](http://string-db.org/newstring_cgi/show_coexpression_evidence.pl?taskId=UGuXufYU98MF&node2=400162) |  |  |  |  | 0.960 | |  | [PF14_0652](http://string-db.org/newstring_cgi/display_single_node.pl?taskId=UGuXufYU98MF&node=399936&targetmode=proteins) | hypothetical protein (862 aa) |  |  |  | [**•**](http://string-db.org/newstring_cgi/show_coexpression_evidence.pl?taskId=UGuXufYU98MF&node2=399936) |  |  |  |  | 0.960 | |  | [PF14_0607](http://string-db.org/newstring_cgi/display_single_node.pl?taskId=UGuXufYU98MF&node=399889&targetmode=proteins) | hypothetical protein (1068 aa) |  |  |  | [**•**](http://string-db.org/newstring_cgi/show_coexpression_evidence.pl?taskId=UGuXufYU98MF&node2=399889) |  |  |  |  | 0.960 | |  | [PF14_0586](http://string-db.org/newstring_cgi/display_single_node.pl?taskId=UGuXufYU98MF&node=399866&targetmode=proteins) | hypothetical protein (323 aa) |  |  |  | [**•**](http://string-db.org/newstring_cgi/show_coexpression_evidence.pl?taskId=UGuXufYU98MF&node2=399866) |  |  |  |  | 0.960 | |  | [PF14_0527](http://string-db.org/newstring_cgi/display_single_node.pl?taskId=UGuXufYU98MF&node=399806&targetmode=proteins) | hypothetical protein (610 aa) |  |  |  | [**•**](http://string-db.org/newstring_cgi/show_coexpression_evidence.pl?taskId=UGuXufYU98MF&node2=399806) |  |  |  |  | 0.960 | |  | [PF13_0173](http://string-db.org/newstring_cgi/display_single_node.pl?taskId=UGuXufYU98MF&node=399088&targetmode=proteins) | hypothetical protein, conserved (1001 aa) |  |  |  | [**•**](http://string-db.org/newstring_cgi/show_coexpression_evidence.pl?taskId=UGuXufYU98MF&node2=399088) |  |  |  |  | 0.960 | |  | [PF13_0058](http://string-db.org/newstring_cgi/display_single_node.pl?taskId=UGuXufYU98MF&node=398981&targetmode=proteins) | RNA-binding protein, putative (143 aa) |  |  |  | [**•**](http://string-db.org/newstring_cgi/show_coexpression_evidence.pl?taskId=UGuXufYU98MF&node2=398981) |  |  |  |  | 0.960 | |  | [PF11_0464](http://string-db.org/newstring_cgi/display_single_node.pl?taskId=UGuXufYU98MF&node=398854&targetmode=proteins) | serine%2Fthreonine protein kinase (2075 aa) |  |  |  | [**•**](http://string-db.org/newstring_cgi/show_coexpression_evidence.pl?taskId=UGuXufYU98MF&node2=398854) |  |  |  |  | 0.960 | |  | [PF11_0442-1](http://string-db.org/newstring_cgi/display_single_node.pl?taskId=UGuXufYU98MF&node=398833&targetmode=proteins) | hypothetical protein (1604 aa) |  |  |  | [**•**](http://string-db.org/newstring_cgi/show_coexpression_evidence.pl?taskId=UGuXufYU98MF&node2=398833) |  |  |  |  | 0.960 | |  | [PF11_0277](http://string-db.org/newstring_cgi/display_single_node.pl?taskId=UGuXufYU98MF&node=398667&targetmode=proteins) | hypothetical protein (350 aa) |  |  |  | [**•**](http://string-db.org/newstring_cgi/show_coexpression_evidence.pl?taskId=UGuXufYU98MF&node2=398667) |  |  |  |  | 0.960 | |  | [PF11_0193](http://string-db.org/newstring_cgi/display_single_node.pl?taskId=UGuXufYU98MF&node=398582&targetmode=proteins) | hypothetical protein (268 aa) |  |  |  | [**•**](http://string-db.org/newstring_cgi/show_coexpression_evidence.pl?taskId=UGuXufYU98MF&node2=398582) |  |  |  |  | 0.960 | |  | [PF10_0368](http://string-db.org/newstring_cgi/display_single_node.pl?taskId=UGuXufYU98MF&node=398364&targetmode=proteins) | dynamin protein, putative (709 aa) |  |  |  | [**•**](http://string-db.org/newstring_cgi/show_coexpression_evidence.pl?taskId=UGuXufYU98MF&node2=398364) |  |  |  |  | 0.960 | |  | [PF10_0351](http://string-db.org/newstring_cgi/display_single_node.pl?taskId=UGuXufYU98MF&node=398347&targetmode=proteins) | hypothetical protein (566 aa) |  |  |  | [**•**](http://string-db.org/newstring_cgi/show_coexpression_evidence.pl?taskId=UGuXufYU98MF&node2=398347) |  |  |  |  | 0.960 | |  | [PF10_0306](http://string-db.org/newstring_cgi/display_single_node.pl?taskId=UGuXufYU98MF&node=398298&targetmode=proteins) | MORN repeat containing protein (422 aa) |  |  |  | [**•**](http://string-db.org/newstring_cgi/show_coexpression_evidence.pl?taskId=UGuXufYU98MF&node2=398298) |  |  |  |  | 0.960 | |  | [PF10_0138](http://string-db.org/newstring_cgi/display_single_node.pl?taskId=UGuXufYU98MF&node=398122&targetmode=proteins) | hypothetical protein (1455 aa) |  |  |  | [**•**](http://string-db.org/newstring_cgi/show_coexpression_evidence.pl?taskId=UGuXufYU98MF&node2=398122) |  |  |  |  | 0.960 | |  | [PF10_0094](http://string-db.org/newstring_cgi/display_single_node.pl?taskId=UGuXufYU98MF&node=398078&targetmode=proteins) | tubulin-tyrosine ligase, putative (553 aa) |  |  |  | [**•**](http://string-db.org/newstring_cgi/show_coexpression_evidence.pl?taskId=UGuXufYU98MF&node2=398078) |  |  |  |  | 0.960 | |  | [PF10_0039](http://string-db.org/newstring_cgi/display_single_node.pl?taskId=UGuXufYU98MF&node=398018&targetmode=proteins) | hypothetical protein (281 aa) |  |  |  | [**•**](http://string-db.org/newstring_cgi/show_coexpression_evidence.pl?taskId=UGuXufYU98MF&node2=398018) |  |  |  |  | 0.960 | |  | [PF08_0058](http://string-db.org/newstring_cgi/display_single_node.pl?taskId=UGuXufYU98MF&node=397895&targetmode=proteins) | hypothetical protein, conserved (962 aa) |  |  |  | [**•**](http://string-db.org/newstring_cgi/show_coexpression_evidence.pl?taskId=UGuXufYU98MF&node2=397895) |  |  |  |  | 0.960 | |  | [PF08_0035](http://string-db.org/newstring_cgi/display_single_node.pl?taskId=UGuXufYU98MF&node=397872&targetmode=proteins) | hypothetical protein, conserved (1176 aa) |  |  |  | [**•**](http://string-db.org/newstring_cgi/show_coexpression_evidence.pl?taskId=UGuXufYU98MF&node2=397872) |  |  |  |  | 0.960 | |  | [PF08_0008](http://string-db.org/newstring_cgi/display_single_node.pl?taskId=UGuXufYU98MF&node=397845&targetmode=proteins) | hypothetical protein, conserved (738 aa) |  |  |  | [**•**](http://string-db.org/newstring_cgi/show_coexpression_evidence.pl?taskId=UGuXufYU98MF&node2=397845) |  |  |  |  | 0.960 | |  | [CPK4](http://string-db.org/newstring_cgi/display_single_node.pl?taskId=UGuXufYU98MF&node=397774&targetmode=proteins) | calcium-dependent protein kinase 4; Calcium-dependent kinase that plays a central role in the s [...] (528 aa) |  |  |  | [**•**](http://string-db.org/newstring_cgi/show_coexpression_evidence.pl?taskId=UGuXufYU98MF&node2=397774) |  |  |  |  | 0.960 | |  | [MAL8P1.73](http://string-db.org/newstring_cgi/display_single_node.pl?taskId=UGuXufYU98MF&node=397675&targetmode=proteins) | hypothetical protein, conserved (1213 aa) |  |  |  | [**•**](http://string-db.org/newstring_cgi/show_coexpression_evidence.pl?taskId=UGuXufYU98MF&node2=397675) |  |  |  |  | 0.960 | |  | [MAL8P1.150](http://string-db.org/newstring_cgi/display_single_node.pl?taskId=UGuXufYU98MF&node=397573&targetmode=proteins) | hypothetical protein, conserved (2166 aa) |  |  |  | [**•**](http://string-db.org/newstring_cgi/show_coexpression_evidence.pl?taskId=UGuXufYU98MF&node2=397573) |  |  |  |  | 0.960 | |  | [MAL7P1.119](http://string-db.org/newstring_cgi/display_single_node.pl?taskId=UGuXufYU98MF&node=397358&targetmode=proteins) | hypothetical protein, conserved (749 aa) |  |  |  | [**•**](http://string-db.org/newstring_cgi/show_coexpression_evidence.pl?taskId=UGuXufYU98MF&node2=397358) |  |  |  |  | 0.960 | |  | [MAL13P1.306-1](http://string-db.org/newstring_cgi/display_single_node.pl?taskId=UGuXufYU98MF&node=397182&targetmode=proteins) | hypothetical protein, conserved (375 aa) |  |  |  | [**•**](http://string-db.org/newstring_cgi/show_coexpression_evidence.pl?taskId=UGuXufYU98MF&node2=397182) |  |  |  |  | 0.960 | | | --- | --- | --- | --- | --- | --- | --- | --- | --- | --- | --- | --- | --- | --- | --- | --- | --- | --- | --- | --- | --- | --- | --- | --- | --- | --- | --- | --- | --- | --- | --- | --- | --- | --- | --- | --- | --- | --- | --- | --- | --- | --- | --- | --- | --- | --- | --- | --- | --- | --- | --- | --- | --- | --- | --- | --- | --- | --- | --- | --- | --- | --- | --- | --- | --- | --- | --- | --- | --- | --- | --- | --- | --- | --- | --- | --- | --- | --- | --- | --- | --- | --- | --- | --- | --- | --- | --- | --- | --- | --- | --- | --- | --- | --- | --- | --- | --- | --- | --- | --- | --- | --- | --- | --- | --- | --- | --- | --- | --- | --- | --- | --- | --- | --- | --- | --- | --- | --- | --- | --- | --- | --- | --- | --- | --- | --- | --- | --- | --- | --- | --- | --- | --- | --- | --- | --- | --- | --- | --- | --- | --- | --- | --- | --- | --- | --- | --- | --- | --- | --- | --- | --- | --- | --- | --- | --- | --- | --- | --- | --- | --- | --- | --- | --- | --- | --- | --- | --- | --- | --- | --- | --- | --- | --- | --- | --- | --- | --- | --- | --- | --- | --- | --- | --- | --- | --- | --- | --- | --- | --- | --- | --- | --- | --- | --- | --- | --- | --- | --- | --- | --- | --- | --- | --- | --- | --- | --- | --- | --- | --- | --- | --- | --- | --- | --- | --- | --- | --- | --- | --- | --- | --- | --- | --- | --- | --- | --- | --- | --- | --- | --- | --- | --- | --- | --- | --- | --- | --- | --- | --- | --- | --- | --- | --- | --- | --- | --- | --- | --- | --- | --- | --- | --- | --- | --- | --- | --- | --- | --- | --- | --- | --- | --- | --- | --- | --- | --- | --- | --- | --- | --- | --- | --- | --- | --- | --- | --- | --- | --- | --- | --- | --- | --- | --- | --- | --- | --- | --- | --- | --- | --- | --- | --- | --- | --- | --- | --- | --- | --- | --- | --- | --- | --- | --- | --- | --- | --- | --- | --- | --- | --- | --- | --- | --- | --- | --- | --- | --- | --- | --- | --- | --- | --- | --- | --- | --- | --- | --- | --- | --- | --- | --- | --- | --- | --- | --- | --- | --- | --- | --- | --- | --- | --- | --- | --- | --- | --- | --- | --- | --- | --- | --- | --- | --- | --- | --- | --- | --- | --- | --- | --- | --- | --- | --- | --- | --- | --- | --- | --- | --- | --- | --- | --- | --- | --- | --- | --- | --- | --- | --- | --- | --- | --- | --- | --- | --- | --- | --- | --- | --- | --- | --- | --- | --- | --- | --- | --- | --- | --- | --- | --- | --- | --- | --- | --- | --- | --- | --- | --- | --- | --- | --- | --- | --- | --- | --- | --- | --- | --- | --- | --- | --- | --- | --- | --- | --- | --- | --- | --- | --- | --- | --- | --- | --- | --- | --- | --- | --- | --- | --- | --- | --- | --- | --- | --- | --- | --- | --- | --- | --- | --- | --- | --- | --- | --- | --- | --- | --- | --- | --- | --- | --- | --- | --- | --- | --- | --- | --- | --- | --- | --- | --- | --- | --- | --- | --- | --- | --- | --- | --- | --- | --- | --- | --- | --- | --- | --- | --- | --- | --- | --- | --- | --- | --- | --- | --- | --- | --- | --- | --- | --- | --- | --- | --- | --- | --- | --- | --- | --- | --- | --- | --- | --- | --- | --- | --- | --- | --- | --- | --- | --- | --- | --- | --- | --- | --- | --- | --- | --- | --- | --- | --- | --- | --- | --- | --- | --- | --- | --- | --- | --- | --- | --- | --- | --- | --- | --- | --- | --- | --- | --- | --- | --- | --- | --- | --- | --- | --- | --- | --- | --- | --- | --- | --- | --- | --- | --- | --- | --- | --- | --- | --- | --- | --- | --- | --- | --- | --- | --- | --- | --- | --- | --- | --- | --- | --- | --- | --- | --- | --- | --- | --- | --- | --- | --- | --- | --- | --- | --- | --- | --- | --- | --- | --- | --- | --- | --- | --- | --- | --- | --- | --- | --- | --- | --- | --- | --- | --- | --- | --- | --- | --- | | | --- | --- | --- | --- | --- | --- | --- | --- | --- | --- | --- | --- | --- | --- | --- | --- | --- | --- | --- | --- | --- | --- | --- | --- | --- | --- | --- | --- | --- | --- | --- | --- | --- | --- | --- | --- | --- | --- | --- | --- | --- | --- | --- | --- | --- | --- | --- | --- | --- | --- | --- | --- | --- | --- | --- | --- | --- | --- | --- | --- | --- | --- | --- | --- | --- | --- | --- | --- | --- | --- | --- | --- | --- | --- | --- | --- | --- | --- | --- | --- | --- | --- | --- | --- | --- | --- | --- | --- | --- | --- | --- | --- | --- | --- | --- | --- | --- | --- | --- | --- | --- | --- | --- | --- | --- | --- | --- | --- | --- | --- | --- | --- | --- | --- | --- | --- | --- | --- | --- | --- | --- | --- | --- | --- | --- | --- | --- | --- | --- | --- | --- | --- | --- | --- | --- | --- | --- | --- | --- | --- | --- | --- | --- | --- | --- | --- | --- | --- | --- | --- | --- | --- | --- | --- | --- | --- | --- | --- | --- | --- | --- | --- | --- | --- | --- | --- | --- | --- | --- | --- | --- | --- | --- | --- | --- | --- | --- | --- | --- | --- | --- | --- | --- | --- | --- | --- | --- | --- | --- | --- | --- | --- | --- | --- | --- | --- | --- | --- | --- | --- | --- | --- | --- | --- | --- | --- | --- | --- | --- | --- | --- | --- | --- | --- | --- | --- | --- | --- | --- | --- | --- | --- | --- | --- | --- | --- | --- | --- | --- | --- | --- | --- | --- | --- | --- | --- | --- | --- | --- | --- | --- | --- | --- | --- | --- | --- | --- | --- | --- | --- | --- | --- | --- | --- | --- | --- | --- | --- | --- | --- | --- | --- | --- | --- | --- | --- | --- | --- | --- | --- | --- | --- | --- | --- | --- | --- | --- | --- | --- | --- | --- | --- | --- | --- | --- | --- | --- | --- | --- | --- | --- | --- | --- | --- | --- | --- | --- | --- | --- | --- | --- | --- | --- | --- | --- | --- | --- | --- | --- | --- | --- | --- | --- | --- | --- | --- | --- | --- | --- | --- | --- | --- | --- | --- | --- | --- | --- | --- | --- | --- | --- | --- | --- | --- | --- | --- | --- | --- | --- | --- | --- | --- | --- | --- | --- | --- | --- | --- | --- | --- | --- | --- | --- | --- | --- | --- | --- | --- | --- | --- | --- | --- | --- | --- | --- | --- | --- | --- | --- | --- | --- | --- | --- | --- | --- | --- | --- | --- | --- | --- | --- | --- | --- | --- | --- | --- | --- | --- | --- | --- | --- | --- | --- | --- | --- | --- | --- | --- | --- | --- | --- | --- | --- | --- | --- | --- | --- | --- | --- | --- | --- | --- | --- | --- | --- | --- | --- | --- | --- | --- | --- | --- | --- | --- | --- | --- | --- | --- | --- | --- | --- | --- | --- | --- | --- | --- | --- | --- | --- | --- | --- | --- | --- | --- | --- | --- | --- | --- | --- | --- | --- | --- | --- | --- | --- | --- | --- | --- | --- | --- | --- | --- | --- | --- | --- | --- | --- | --- | --- | --- | --- | --- | --- | --- | --- | --- | --- | --- | --- | --- | --- | --- | --- | --- | --- | --- | --- | --- | --- | --- | --- | --- | --- | --- | --- | --- | --- | --- | --- | --- | --- | --- | --- | --- | --- | --- | --- | --- | --- | --- | --- | --- | --- | --- | --- | --- | --- | --- | --- | --- | --- | --- | --- | --- | --- | --- | --- | --- | --- | --- | --- | --- | --- | --- | --- | --- | --- | --- | --- | --- | --- | --- | --- | --- | --- | --- | --- | --- | --- | --- | --- | --- | --- | --- | --- | --- | --- | --- | --- | --- | --- | --- | --- | --- | --- | --- | --- | --- | --- | --- | --- | --- | --- | --- | --- | --- | --- | --- | --- | --- | --- | --- | --- | --- | --- | --- | --- | --- | --- | --- | --- | --- | --- | --- | --- | --- | --- | --- | --- | --- | --- | --- | --- | --- | --- | --- | --- | --- | --- | --- | --- | --- | --- | --- | --- | --- | --- | --- | --- | --- | --- | --- | --- | |
| --- | --- | --- | --- | --- | --- | --- | --- | --- | --- | --- | --- | --- | --- | --- | --- | --- | --- | --- | --- | --- | --- | --- | --- | --- | --- | --- | --- | --- | --- | --- | --- | --- | --- | --- | --- | --- | --- | --- | --- | --- | --- | --- | --- | --- | --- | --- | --- | --- | --- | --- | --- | --- | --- | --- | --- | --- | --- | --- | --- | --- | --- | --- | --- | --- | --- | --- | --- | --- | --- | --- | --- | --- | --- | --- | --- | --- | --- | --- | --- | --- | --- | --- | --- | --- | --- | --- | --- | --- | --- | --- | --- | --- | --- | --- | --- | --- | --- | --- | --- | --- | --- | --- | --- | --- | --- | --- | --- | --- | --- | --- | --- | --- | --- | --- | --- | --- | --- | --- | --- | --- | --- | --- | --- | --- | --- | --- | --- | --- | --- | --- | --- | --- | --- | --- | --- | --- | --- | --- | --- | --- | --- | --- | --- | --- | --- | --- | --- | --- | --- | --- | --- | --- | --- | --- | --- | --- | --- | --- | --- | --- | --- | --- | --- | --- | --- | --- | --- | --- | --- | --- | --- | --- | --- | --- | --- | --- | --- | --- | --- | --- | --- | --- | --- | --- | --- | --- | --- | --- | --- | --- | --- | --- | --- | --- | --- | --- | --- | --- | --- | --- | --- | --- | --- | --- | --- | --- | --- | --- | --- | --- | --- | --- | --- | --- | --- | --- | --- | --- | --- | --- | --- | --- | --- | --- | --- | --- | --- | --- | --- | --- | --- | --- | --- | --- | --- | --- | --- | --- | --- | --- | --- | --- | --- | --- | --- | --- | --- | --- | --- | --- | --- | --- | --- | --- | --- | --- | --- | --- | --- | --- | --- | --- | --- | --- | --- | --- | --- | --- | --- | --- | --- | --- | --- | --- | --- | --- | --- | --- | --- | --- | --- | --- | --- | --- | --- | --- | --- | --- | --- | --- | --- | --- | --- | --- | --- | --- | --- | --- | --- | --- | --- | --- | --- | --- | --- | --- | --- | --- | --- | --- | --- | --- | --- | --- | --- | --- | --- | --- | --- | --- | --- | --- | --- | --- | --- | --- | --- | --- | --- | --- | --- | --- | --- | --- | --- | --- | --- | --- | --- | --- | --- | --- | --- | --- | --- | --- | --- | --- | --- | --- | --- | --- | --- | --- | --- | --- | --- | --- | --- | --- | --- | --- | --- | --- | --- | --- | --- | --- | --- | --- | --- | --- | --- | --- | --- | --- | --- | --- | --- | --- | --- | --- | --- | --- | --- | --- | --- | --- | --- | --- | --- | --- | --- | --- | --- | --- | --- | --- | --- | --- | --- | --- | --- | --- | --- | --- | --- | --- | --- | --- | --- | --- | --- | --- | --- | --- | --- | --- | --- | --- | --- | --- | --- | --- | --- | --- | --- | --- | --- | --- | --- | --- | --- | --- | --- | --- | --- | --- | --- | --- | --- | --- | --- | --- | --- | --- | --- | --- | --- | --- | --- | --- | --- | --- | --- | --- | --- | --- | --- | --- | --- | --- | --- | --- | --- | --- | --- | --- | --- | --- | --- | --- | --- | --- | --- | --- | --- | --- | --- | --- | --- | --- | --- | --- | --- | --- | --- | --- | --- | --- | --- | --- | --- | --- | --- | --- | --- | --- | --- | --- | --- | --- | --- | --- | --- | --- | --- | --- | --- | --- | --- | --- | --- | --- | --- | --- | --- | --- | --- | --- | --- | --- | --- | --- | --- | --- | --- | --- | --- | --- | --- | --- | --- | --- | --- | --- | --- | --- | --- | --- | --- | --- | --- | --- | --- | --- | --- | --- | --- | --- | --- | --- | --- | --- | --- | --- | --- | --- | --- | --- | --- | --- | --- | --- | --- | --- | --- | --- | --- | --- | --- | --- | --- | --- | --- | --- | --- | --- | --- | --- | --- | --- | --- | --- | --- | --- | --- | --- | --- | --- | --- | --- | --- | --- | --- | --- | --- | --- | --- | --- | --- | --- | --- | --- | --- | --- | --- | --- | --- | --- | --- | --- | --- | --- | --- | --- | --- | --- | --- | --- | --- | --- | --- |
|  |

56.

| | | | **Your Input:** **>PF3D7_1430600** | | |  |  |  |  |  |  |  |  |  | | --- | --- | --- | --- | --- | --- | --- | --- | --- | --- | --- | --- | |  | [PF14_0285](http://string-db.org/newstring_cgi/display_single_node.pl?taskId=CJ0LVOSlBLrL&node=399559&targetmode=proteins) | exodeoxyribonuclease III, putative (876 aa) | |  |  | *(Plasmodium falciparum)* | | **Predicted Functional Partners:** | | | |  | [PFA_0545c](http://string-db.org/newstring_cgi/display_single_node.pl?taskId=CJ0LVOSlBLrL&node=400183&targetmode=proteins) | replication factor c protein, putative (1167 aa) |  |  |  | [**•**](http://string-db.org/newstring_cgi/show_coexpression_evidence.pl?taskId=CJ0LVOSlBLrL&node2=400183) |  |  |  |  | 0.479 | |  | [PF11_0159](http://string-db.org/newstring_cgi/display_single_node.pl?taskId=CJ0LVOSlBLrL&node=398546&targetmode=proteins) | hypothetical protein (144 aa) |  |  |  | [**•**](http://string-db.org/newstring_cgi/show_coexpression_evidence.pl?taskId=CJ0LVOSlBLrL&node2=398546) |  |  |  |  | 0.425 | |  | [PFI0960w](http://string-db.org/newstring_cgi/display_single_node.pl?taskId=CJ0LVOSlBLrL&node=401804&targetmode=proteins) | dolichyl-diphosphooligosaccharide--protein-glyc ot ransferase,putative (477 aa) |  |  |  | [**•**](http://string-db.org/newstring_cgi/show_coexpression_evidence.pl?taskId=CJ0LVOSlBLrL&node2=401804) |  |  |  |  | 0.423 | | | --- | --- | --- | --- | --- | --- | --- | --- | --- | --- | --- | --- | --- | --- | --- | --- | --- | --- | --- | --- | --- | --- | --- | --- | --- | --- | --- | --- | --- | --- | --- | --- | --- | --- | --- | --- | --- | --- | --- | --- | --- | --- | --- | --- | --- | --- | --- | --- | --- | --- | --- | --- | --- | --- | --- | --- | --- | --- | | | --- | --- | --- | --- | --- | --- | --- | --- | --- | --- | --- | --- | --- | --- | --- | --- | --- | --- | --- | --- | --- | --- | --- | --- | --- | --- | --- | --- | --- | --- | --- | --- | --- | --- | --- | --- | --- | --- | --- | --- | --- | --- | --- | --- | --- | --- | --- | --- | --- | --- | --- | --- | --- | --- | --- | --- | --- | --- | --- | |
| --- | --- | --- | --- | --- | --- | --- | --- | --- | --- | --- | --- | --- | --- | --- | --- | --- | --- | --- | --- | --- | --- | --- | --- | --- | --- | --- | --- | --- | --- | --- | --- | --- | --- | --- | --- | --- | --- | --- | --- | --- | --- | --- | --- | --- | --- | --- | --- | --- | --- | --- | --- | --- | --- | --- | --- | --- | --- | --- | --- |
| |  | | --- | |

57.

| **Your Input>PF3D7_1431800:** | | |  |  |  |  |  |  |  |  |  |
| --- | --- | --- | --- | --- | --- | --- | --- | --- | --- | --- | --- |
|  | [PF14_0297](http://string-db.org/newstring_cgi/display_single_node.pl?taskId=Caco_SSeP0SB&node=399571&targetmode=proteins) | ecto-nucleoside triphosphate diphosphohydrolase 1, putative (827 aa) |  |  |  |  |  |  |  |  |  |
|  |  | *(Plasmodium falciparum)* |  |  |  |  |  |  |  |  |  |
| **Predicted Functional Partners:** | | |  |  |  |  |  |  |  |  |  |
|  | [PF10_0363](http://string-db.org/newstring_cgi/display_single_node.pl?taskId=Caco_SSeP0SB&node=398359&targetmode=proteins) | pyruvate kinase, putative (745 aa) |  |  |  | [**•**](http://string-db.org/newstring_cgi/show_coexpression_evidence.pl?taskId=Caco_SSeP0SB&node2=398359) |  | [**•**](http://string-db.org/newstring_cgi/show_set_evidence.pl?data_channel=database&taskId=Caco_SSeP0SB&node2=398359) |  |  | 0.910 |
|  | [PFL1655c](http://string-db.org/newstring_cgi/display_single_node.pl?taskId=Caco_SSeP0SB&node=402324&targetmode=proteins) | epsilon DNA polymerase, putative (624 aa) |  |  |  | [**•**](http://string-db.org/newstring_cgi/show_coexpression_evidence.pl?taskId=Caco_SSeP0SB&node2=402324) |  | [**•**](http://string-db.org/newstring_cgi/show_set_evidence.pl?data_channel=database&taskId=Caco_SSeP0SB&node2=402324) |  |  | 0.907 |
|  | [POM1](http://string-db.org/newstring_cgi/display_single_node.pl?taskId=Caco_SSeP0SB&node=399381&targetmode=proteins) | POM1, putative (2016 aa) |  |  |  | [**•**](http://string-db.org/newstring_cgi/show_coexpression_evidence.pl?taskId=Caco_SSeP0SB&node2=399381) |  | [**•**](http://string-db.org/newstring_cgi/show_set_evidence.pl?data_channel=database&taskId=Caco_SSeP0SB&node2=399381) |  |  | 0.907 |
|  | [PFC0340w](http://string-db.org/newstring_cgi/display_single_node.pl?taskId=Caco_SSeP0SB&node=400535&targetmode=proteins) | DNA polymerase delta small subunit, putative (498 aa) |  |  |  | [**•**](http://string-db.org/newstring_cgi/show_coexpression_evidence.pl?taskId=Caco_SSeP0SB&node2=400535) |  | [**•**](http://string-db.org/newstring_cgi/show_set_evidence.pl?data_channel=database&taskId=Caco_SSeP0SB&node2=400535) |  |  | 0.906 |
|  | [PF10_0165](http://string-db.org/newstring_cgi/display_single_node.pl?taskId=Caco_SSeP0SB&node=398151&targetmode=proteins) | DNA polymerase delta catalytic subunit; This polymerase possesses two enzymatic activities- DNA [...] (1094 aa) |  |  |  | [**•**](http://string-db.org/newstring_cgi/show_coexpression_evidence.pl?taskId=Caco_SSeP0SB&node2=398151) |  | [**•**](http://string-db.org/newstring_cgi/show_set_evidence.pl?data_channel=database&taskId=Caco_SSeP0SB&node2=398151) |  |  | 0.905 |
|  | [PFE0215w](http://string-db.org/newstring_cgi/display_single_node.pl?taskId=Caco_SSeP0SB&node=401001&targetmode=proteins) | ATP-dependent helicase, putative (755 aa) |  |  |  | [**•**](http://string-db.org/newstring_cgi/show_coexpression_evidence.pl?taskId=Caco_SSeP0SB&node2=401001) |  | [**•**](http://string-db.org/newstring_cgi/show_set_evidence.pl?data_channel=database&taskId=Caco_SSeP0SB&node2=401001) |  |  | 0.905 |
|  | [PF10_0121](http://string-db.org/newstring_cgi/display_single_node.pl?taskId=Caco_SSeP0SB&node=398105&targetmode=proteins) | hypoxanthine phosphoribosyltransferase; Works with guanine, hypoxanthine and xanthine (231 aa) |  |  |  | [**•**](http://string-db.org/newstring_cgi/show_coexpression_evidence.pl?taskId=Caco_SSeP0SB&node2=398105) |  | [**•**](http://string-db.org/newstring_cgi/show_set_evidence.pl?data_channel=database&taskId=Caco_SSeP0SB&node2=398105) |  |  | 0.905 |
|  | [PF14_0602](http://string-db.org/newstring_cgi/display_single_node.pl?taskId=Caco_SSeP0SB&node=399883&targetmode=proteins) | DNA polymerase alpha subunit, putative (539 aa) |  |  |  | [**•**](http://string-db.org/newstring_cgi/show_coexpression_evidence.pl?taskId=Caco_SSeP0SB&node2=399883) |  | [**•**](http://string-db.org/newstring_cgi/show_set_evidence.pl?data_channel=database&taskId=Caco_SSeP0SB&node2=399883) |  |  | 0.904 |
|  | [PF14_0053](http://string-db.org/newstring_cgi/display_single_node.pl?taskId=Caco_SSeP0SB&node=399322&targetmode=proteins) | ribonucleotide reductase small subunit; Provides the precursors necessary for DNA synthesis. Ca [...] (349 aa) |  |  |  | [**•**](http://string-db.org/newstring_cgi/show_coexpression_evidence.pl?taskId=Caco_SSeP0SB&node2=399322) |  | [**•**](http://string-db.org/newstring_cgi/show_set_evidence.pl?data_channel=database&taskId=Caco_SSeP0SB&node2=399322) |  |  | 0.904 |
|  | [TMK](http://string-db.org/newstring_cgi/display_single_node.pl?taskId=Caco_SSeP0SB&node=402488&targetmode=proteins) | thymidylate kinase, putative (210 aa) |  |  |  |  |  | [**•**](http://string-db.org/newstring_cgi/show_set_evidence.pl?data_channel=database&taskId=Caco_SSeP0SB&node2=402488) |  |  | 0.899 |
|  | [PFL2010c](http://string-db.org/newstring_cgi/display_single_node.pl?taskId=Caco_SSeP0SB&node=402396&targetmode=proteins) | DEAD%2FDEAH box helicase, putative (894 aa) |  |  |  |  |  | [**•**](http://string-db.org/newstring_cgi/show_set_evidence.pl?data_channel=database&taskId=Caco_SSeP0SB&node2=402396) |  |  | 0.899 |
|  | [PFL1525c](http://string-db.org/newstring_cgi/display_single_node.pl?taskId=Caco_SSeP0SB&node=402296&targetmode=proteins) | pre-mRNA splicing factor RNA helicase, putative (1168 aa) |  |  |  |  |  | [**•**](http://string-db.org/newstring_cgi/show_set_evidence.pl?data_channel=database&taskId=Caco_SSeP0SB&node2=402296) |  |  | 0.899 |
|  | [PFL1435c](http://string-db.org/newstring_cgi/display_single_node.pl?taskId=Caco_SSeP0SB&node=402278&targetmode=proteins) | myosin d (2231 aa) |  |  |  |  |  | [**•**](http://string-db.org/newstring_cgi/show_set_evidence.pl?data_channel=database&taskId=Caco_SSeP0SB&node2=402278) |  |  | 0.899 |
|  | [PFL0665c](http://string-db.org/newstring_cgi/display_single_node.pl?taskId=Caco_SSeP0SB&node=402120&targetmode=proteins) | RNA polymerase subunit 8c, putative (145 aa) |  |  |  |  |  | [**•**](http://string-db.org/newstring_cgi/show_set_evidence.pl?data_channel=database&taskId=Caco_SSeP0SB&node2=402120) |  |  | 0.899 |
|  | [PFL0560c](http://string-db.org/newstring_cgi/display_single_node.pl?taskId=Caco_SSeP0SB&node=402097&targetmode=proteins) | minichromosome maintenance protein, putative (1024 aa) |  |  |  |  |  | [**•**](http://string-db.org/newstring_cgi/show_set_evidence.pl?data_channel=database&taskId=Caco_SSeP0SB&node2=402097) |  |  | 0.899 |
|  | [PDE1](http://string-db.org/newstring_cgi/display_single_node.pl?taskId=Caco_SSeP0SB&node=402080&targetmode=proteins) | 3',5'-cyclic-nucleotide phosphodiesterase, putative (842 aa) |  |  |  |  |  | [**•**](http://string-db.org/newstring_cgi/show_set_evidence.pl?data_channel=database&taskId=Caco_SSeP0SB&node2=402080) |  |  | 0.899 |
|  | [PFL0330c](http://string-db.org/newstring_cgi/display_single_node.pl?taskId=Caco_SSeP0SB&node=402051&targetmode=proteins) | DNA-directed RNA polymerase III subunit, putative (1450 aa) |  |  |  |  |  | [**•**](http://string-db.org/newstring_cgi/show_set_evidence.pl?data_channel=database&taskId=Caco_SSeP0SB&node2=402051) |  |  | 0.899 |
|  | [PFL0100c](http://string-db.org/newstring_cgi/display_single_node.pl?taskId=Caco_SSeP0SB&node=402005&targetmode=proteins) | ATP dependent RNA helicase, putative (807 aa) |  |  |  |  |  | [**•**](http://string-db.org/newstring_cgi/show_set_evidence.pl?data_channel=database&taskId=Caco_SSeP0SB&node2=402005) |  |  | 0.899 |
|  | [PfGK](http://string-db.org/newstring_cgi/display_single_node.pl?taskId=Caco_SSeP0SB&node=401898&targetmode=proteins) | guanylate kinase, putative (199 aa) |  |  |  |  |  | [**•**](http://string-db.org/newstring_cgi/show_set_evidence.pl?data_channel=database&taskId=Caco_SSeP0SB&node2=401898) |  |  | 0.899 |
|  | [PFI1130c](http://string-db.org/newstring_cgi/display_single_node.pl?taskId=Caco_SSeP0SB&node=401838&targetmode=proteins) | DNA-directed RNA polymerase II, putative (335 aa) |  |  |  |  |  | [**•**](http://string-db.org/newstring_cgi/show_set_evidence.pl?data_channel=database&taskId=Caco_SSeP0SB&node2=401838) |  |  | 0.899 |
|  | [PFI1020c](http://string-db.org/newstring_cgi/display_single_node.pl?taskId=Caco_SSeP0SB&node=401816&targetmode=proteins) | Inosine-5'-monophosphate dehydrogenase (510 aa) |  |  |  |  |  | [**•**](http://string-db.org/newstring_cgi/show_set_evidence.pl?data_channel=database&taskId=Caco_SSeP0SB&node2=401816) |  |  | 0.899 |
|  | [PFI0910w](http://string-db.org/newstring_cgi/display_single_node.pl?taskId=Caco_SSeP0SB&node=401794&targetmode=proteins) | DNA helicase, putative (728 aa) |  |  |  |  |  | [**•**](http://string-db.org/newstring_cgi/show_set_evidence.pl?data_channel=database&taskId=Caco_SSeP0SB&node2=401794) |  |  | 0.899 |
|  | [PFI0530c](http://string-db.org/newstring_cgi/display_single_node.pl?taskId=Caco_SSeP0SB&node=401717&targetmode=proteins) | DNA primase, large subunit, putative (525 aa) |  |  |  |  |  | [**•**](http://string-db.org/newstring_cgi/show_set_evidence.pl?data_channel=database&taskId=Caco_SSeP0SB&node2=401717) |  |  | 0.899 |
|  | [PFF1470c](http://string-db.org/newstring_cgi/display_single_node.pl?taskId=Caco_SSeP0SB&node=401582&targetmode=proteins) | DNA polymerase epsilon, catalytic subunit a, putative (2907 aa) |  |  |  |  |  | [**•**](http://string-db.org/newstring_cgi/show_set_evidence.pl?data_channel=database&taskId=Caco_SSeP0SB&node2=401582) |  | **•** | 0.899 |
|  | [PFF1300w](http://string-db.org/newstring_cgi/display_single_node.pl?taskId=Caco_SSeP0SB&node=401547&targetmode=proteins) | pyruvate kinase, putative (511 aa) |  |  |  |  |  | [**•**](http://string-db.org/newstring_cgi/show_set_evidence.pl?data_channel=database&taskId=Caco_SSeP0SB&node2=401547) |  |  | 0.899 |
|  | [PFF1225c](http://string-db.org/newstring_cgi/display_single_node.pl?taskId=Caco_SSeP0SB&node=401532&targetmode=proteins) | DNA polymerase 1, putative (1444 aa) |  |  |  |  |  | [**•**](http://string-db.org/newstring_cgi/show_set_evidence.pl?data_channel=database&taskId=Caco_SSeP0SB&node2=401532) |  |  | 0.899 |
|  | [PFF0275c](http://string-db.org/newstring_cgi/display_single_node.pl?taskId=Caco_SSeP0SB&node=401338&targetmode=proteins) | nucleoside diphosphate kinase, putative (1828 aa) |  |  |  |  |  | [**•**](http://string-db.org/newstring_cgi/show_set_evidence.pl?data_channel=database&taskId=Caco_SSeP0SB&node2=401338) |  | **•** | 0.899 |
|  | [PFE1035c](http://string-db.org/newstring_cgi/display_single_node.pl?taskId=Caco_SSeP0SB&node=401165&targetmode=proteins) | BIS(5'-nucleosyl)-tetraphosphatase (diadenosine tetraphosphatase), putative (153 aa) |  |  |  |  |  | [**•**](http://string-db.org/newstring_cgi/show_set_evidence.pl?data_channel=database&taskId=Caco_SSeP0SB&node2=401165) |  |  | 0.899 |
|  | [PFE0465c](http://string-db.org/newstring_cgi/display_single_node.pl?taskId=Caco_SSeP0SB&node=401052&targetmode=proteins) | RNA polymerase I (2914 aa) |  |  |  |  |  | [**•**](http://string-db.org/newstring_cgi/show_set_evidence.pl?data_channel=database&taskId=Caco_SSeP0SB&node2=401052) |  |  | 0.899 |
|  | [PFE0205w](http://string-db.org/newstring_cgi/display_single_node.pl?taskId=Caco_SSeP0SB&node=400999&targetmode=proteins) | ATP-dependent helicase, putative (502 aa) |  |  |  |  |  | [**•**](http://string-db.org/newstring_cgi/show_set_evidence.pl?data_channel=database&taskId=Caco_SSeP0SB&node2=400999) |  | **•** | 0.899 |
|  | [PFD1060w](http://string-db.org/newstring_cgi/display_single_node.pl?taskId=Caco_SSeP0SB&node=400921&targetmode=proteins) | u5 small nuclear ribonucleoprotein-specific protein, putative (2874 aa) |  |  |  |  |  | [**•**](http://string-db.org/newstring_cgi/show_set_evidence.pl?data_channel=database&taskId=Caco_SSeP0SB&node2=400921) |  |  | 0.899 |
|  | [PFD0805w](http://string-db.org/newstring_cgi/display_single_node.pl?taskId=Caco_SSeP0SB&node=400863&targetmode=proteins) | prohibitin-like protein, putative (287 aa) |  |  |  |  |  | [**•**](http://string-db.org/newstring_cgi/show_set_evidence.pl?data_channel=database&taskId=Caco_SSeP0SB&node2=400863) |  |  | 0.899 |
|  | [GAK](http://string-db.org/newstring_cgi/display_single_node.pl?taskId=Caco_SSeP0SB&node=400853&targetmode=proteins) | adenylate kinase 1 (229 aa) |  |  |  |  |  | [**•**](http://string-db.org/newstring_cgi/show_set_evidence.pl?data_channel=database&taskId=Caco_SSeP0SB&node2=400853) |  |  | 0.899 |
|  | [PFD0590c](http://string-db.org/newstring_cgi/display_single_node.pl?taskId=Caco_SSeP0SB&node=400817&targetmode=proteins) | DNA polymerase alpha (1912 aa) |  |  |  |  |  | [**•**](http://string-db.org/newstring_cgi/show_set_evidence.pl?data_channel=database&taskId=Caco_SSeP0SB&node2=400817) |  | **•** | 0.899 |
|  | [PFD0565c](http://string-db.org/newstring_cgi/display_single_node.pl?taskId=Caco_SSeP0SB&node=400814&targetmode=proteins) | RNA helicase, putative (1304 aa) |  |  |  |  |  | [**•**](http://string-db.org/newstring_cgi/show_set_evidence.pl?data_channel=database&taskId=Caco_SSeP0SB&node2=400814) |  | **•** | 0.899 |
|  | [PFD0245c](http://string-db.org/newstring_cgi/display_single_node.pl?taskId=Caco_SSeP0SB&node=400750&targetmode=proteins) | ATP-dependent RNA helicae-like protein, putative (567 aa) |  |  |  |  |  | [**•**](http://string-db.org/newstring_cgi/show_set_evidence.pl?data_channel=database&taskId=Caco_SSeP0SB&node2=400750) |  |  | 0.899 |
|  | [PFC0805w](http://string-db.org/newstring_cgi/display_single_node.pl?taskId=Caco_SSeP0SB&node=400633&targetmode=proteins) | DNA-directed RNA polymerase II, putative; DNA-dependent RNA polymerase catalyzes the transcript [...] (2457 aa) |  |  |  |  |  | [**•**](http://string-db.org/newstring_cgi/show_set_evidence.pl?data_channel=database&taskId=Caco_SSeP0SB&node2=400633) |  |  | 0.899 |
|  | [PFB0715w](http://string-db.org/newstring_cgi/display_single_node.pl?taskId=Caco_SSeP0SB&node=400375&targetmode=proteins) | DNA-directed RNA polymerase II second largest subunit, putative (1364 aa) |  |  |  |  |  | [**•**](http://string-db.org/newstring_cgi/show_set_evidence.pl?data_channel=database&taskId=Caco_SSeP0SB&node2=400375) |  |  | 0.899 |
|  | [ASL](http://string-db.org/newstring_cgi/display_single_node.pl?taskId=Caco_SSeP0SB&node=400288&targetmode=proteins) | adenylosuccinate lyase, putative (471 aa) |  |  |  |  |  | [**•**](http://string-db.org/newstring_cgi/show_set_evidence.pl?data_channel=database&taskId=Caco_SSeP0SB&node2=400288) |  |  | 0.899 |
|  | [PFB0290c](http://string-db.org/newstring_cgi/display_single_node.pl?taskId=Caco_SSeP0SB&node=400287&targetmode=proteins) | transcription factor, putative (106 aa) |  |  |  |  |  | [**•**](http://string-db.org/newstring_cgi/show_set_evidence.pl?data_channel=database&taskId=Caco_SSeP0SB&node2=400287) |  |  | 0.899 |
|  | [PFB0245c](http://string-db.org/newstring_cgi/display_single_node.pl?taskId=Caco_SSeP0SB&node=400276&targetmode=proteins) | DNA-directed RNA polymerase II 16 kDa subunit, putative (132 aa) |  |  |  |  |  | [**•**](http://string-db.org/newstring_cgi/show_set_evidence.pl?data_channel=database&taskId=Caco_SSeP0SB&node2=400276) |  |  | 0.899 |
|  | [PFA_0555c](http://string-db.org/newstring_cgi/display_single_node.pl?taskId=Caco_SSeP0SB&node=400185&targetmode=proteins) | UMP-CMP kinase, putative (371 aa) |  |  |  |  |  | [**•**](http://string-db.org/newstring_cgi/show_set_evidence.pl?data_channel=database&taskId=Caco_SSeP0SB&node2=400185) |  |  | 0.899 |
|  | [PFA_0505c](http://string-db.org/newstring_cgi/display_single_node.pl?taskId=Caco_SSeP0SB&node=400175&targetmode=proteins) | DNA-directed RNA polymerase 2 subunit, putative (249 aa) |  |  |  |  |  | [**•**](http://string-db.org/newstring_cgi/show_set_evidence.pl?data_channel=database&taskId=Caco_SSeP0SB&node2=400175) |  |  | 0.899 |
|  | [PF14_0695](http://string-db.org/newstring_cgi/display_single_node.pl?taskId=Caco_SSeP0SB&node=399980&targetmode=proteins) | DNA-directed RNA polymerase, alpha subunit, truncated, putative (825 aa) |  |  |  |  |  | [**•**](http://string-db.org/newstring_cgi/show_set_evidence.pl?data_channel=database&taskId=Caco_SSeP0SB&node2=399980) |  |  | 0.899 |
|  | [PDEdelta](http://string-db.org/newstring_cgi/display_single_node.pl?taskId=Caco_SSeP0SB&node=399957&targetmode=proteins) | cyclic nucleotide phosphodiesterase, putative (813 aa) |  |  |  |  |  | [**•**](http://string-db.org/newstring_cgi/show_set_evidence.pl?data_channel=database&taskId=Caco_SSeP0SB&node2=399957) |  |  | 0.899 |
|  | [PF14_0563](http://string-db.org/newstring_cgi/display_single_node.pl?taskId=Caco_SSeP0SB&node=399842&targetmode=proteins) | DEAD-box RNA helicase, putative (741 aa) |  |  |  |  |  | [**•**](http://string-db.org/newstring_cgi/show_set_evidence.pl?data_channel=database&taskId=Caco_SSeP0SB&node2=399842) |  |  | 0.899 |
|  | [PF14_0370](http://string-db.org/newstring_cgi/display_single_node.pl?taskId=Caco_SSeP0SB&node=399646&targetmode=proteins) | RNA helicase, putative (2472 aa) |  |  |  |  |  | [**•**](http://string-db.org/newstring_cgi/show_set_evidence.pl?data_channel=database&taskId=Caco_SSeP0SB&node2=399646) |  |  | 0.899 |
|  | [PF14_0366](http://string-db.org/newstring_cgi/display_single_node.pl?taskId=Caco_SSeP0SB&node=399642&targetmode=proteins) | small subunit DNA primase; DNA primase is the polymerase that synthesizes small RNA primers for [...] (452 aa) |  |  |  |  |  | [**•**](http://string-db.org/newstring_cgi/show_set_evidence.pl?data_channel=database&taskId=Caco_SSeP0SB&node2=399642) |  |  | 0.899 |
|  | [PF14_0352](http://string-db.org/newstring_cgi/display_single_node.pl?taskId=Caco_SSeP0SB&node=399628&targetmode=proteins) | ribonucleoside-diphosphate reductase, large subunit; Provides the precursors necessary for DNA [...] (847 aa) |  |  |  |  |  | [**•**](http://string-db.org/newstring_cgi/show_set_evidence.pl?data_channel=database&taskId=Caco_SSeP0SB&node2=399628) |  |  | 0.899 |
|  | [PF14_0278](http://string-db.org/newstring_cgi/display_single_node.pl?taskId=Caco_SSeP0SB&node=399552&targetmode=proteins) | ATP-dependent DNA helicase, putative (1440 aa) |  |  |  |  |  | [**•**](http://string-db.org/newstring_cgi/show_set_evidence.pl?data_channel=database&taskId=Caco_SSeP0SB&node2=399552) |  |  | 0.899 |

58.

| **Your Input:** **>PF3D7_1455100** | | |  |  |  |  |  |  |  |  |  |
| --- | --- | --- | --- | --- | --- | --- | --- | --- | --- | --- | --- |
|  | [PF14_0525](http://string-db.org/newstring_cgi/display_single_node.pl?taskId=Yqna9JZm63wB&node=399804&targetmode=proteins) | hypothetical protein (89 aa) |  |  |  |  |  |  |  |  |  |
|  |  | *(Plasmodium falciparum)* |  |  |  |  |  |  |  |  |  |
| **Predicted Functional Partners:** | | |  |  |  |  |  |  |  |  |  |
|  | [PF14_0524](http://string-db.org/newstring_cgi/display_single_node.pl?taskId=Yqna9JZm63wB&node=399803&targetmode=proteins) | protein phosphatase 7 homolog, putative (96 aa) |  | [**•**](http://string-db.org/newstring_cgi/show_fusion_evidence.pl?taskId=Yqna9JZm63wB&node2=399803) |  | [**•**](http://string-db.org/newstring_cgi/show_coexpression_evidence.pl?taskId=Yqna9JZm63wB&node2=399803) |  |  |  |  | 0.874 |
|  | [PF11_0281](http://string-db.org/newstring_cgi/display_single_node.pl?taskId=Yqna9JZm63wB&node=398671&targetmode=proteins) | hypothetical protein (247 aa) |  |  |  |  |  |  | [**•**](http://string-db.org/newstring_cgi/show_textmining_evidence.pl?taskId=Yqna9JZm63wB&node2=398671) |  | 0.786 |
|  | [PRL](http://string-db.org/newstring_cgi/display_single_node.pl?taskId=Yqna9JZm63wB&node=398526&targetmode=proteins) | protein tyrosine phosphatase, putative (218 aa) |  |  |  |  |  |  | [**•**](http://string-db.org/newstring_cgi/show_textmining_evidence.pl?taskId=Yqna9JZm63wB&node2=398526) |  | 0.780 |
|  | [PFC0380w](http://string-db.org/newstring_cgi/display_single_node.pl?taskId=Yqna9JZm63wB&node=400544&targetmode=proteins) | dual-specificity protein phosphatase, putative (575 aa) |  |  |  |  |  |  | [**•**](http://string-db.org/newstring_cgi/show_textmining_evidence.pl?taskId=Yqna9JZm63wB&node2=400544) |  | 0.685 |
|  | [PFL0320w](http://string-db.org/newstring_cgi/display_single_node.pl?taskId=Yqna9JZm63wB&node=402049&targetmode=proteins) | hypothetical protein, conserved (346 aa) |  |  |  |  |  |  | [**•**](http://string-db.org/newstring_cgi/show_textmining_evidence.pl?taskId=Yqna9JZm63wB&node2=402049) |  | 0.579 |
|  | [PF13_0027](http://string-db.org/newstring_cgi/display_single_node.pl?taskId=Yqna9JZm63wB&node=398952&targetmode=proteins) | hypothetical protein, conserved (771 aa) |  |  |  |  |  |  | [**•**](http://string-db.org/newstring_cgi/show_textmining_evidence.pl?taskId=Yqna9JZm63wB&node2=398952) |  | 0.579 |
|  | [MAL13P1.275-1](http://string-db.org/newstring_cgi/display_single_node.pl?taskId=Yqna9JZm63wB&node=397149&targetmode=proteins) | NLI interacting factor-like phosphatase, putative (1288 aa) |  |  |  |  |  |  | [**•**](http://string-db.org/newstring_cgi/show_textmining_evidence.pl?taskId=Yqna9JZm63wB&node2=397149) |  | 0.579 |
|  | [MAL13P1.168-1](http://string-db.org/newstring_cgi/display_single_node.pl?taskId=Yqna9JZm63wB&node=397036&targetmode=proteins) | hypothetical protein, conserved (267 aa) |  |  |  |  |  |  | [**•**](http://string-db.org/newstring_cgi/show_textmining_evidence.pl?taskId=Yqna9JZm63wB&node2=397036) |  | 0.579 |
|  | [PFL2365w](http://string-db.org/newstring_cgi/display_single_node.pl?taskId=Yqna9JZm63wB&node=402468&targetmode=proteins) | hypothetical protein, conserved (1027 aa) |  |  |  |  |  |  | [**•**](http://string-db.org/newstring_cgi/show_textmining_evidence.pl?taskId=Yqna9JZm63wB&node2=402468) |  | 0.484 |
|  | [PF14_0523](http://string-db.org/newstring_cgi/display_single_node.pl?taskId=Yqna9JZm63wB&node=399802&targetmode=proteins) | protein phosphatase 2C, putative (289 aa) |  |  |  |  |  |  | [**•**](http://string-db.org/newstring_cgi/show_textmining_evidence.pl?taskId=Yqna9JZm63wB&node2=399802) |  | 0.484 |

59.

| **Your Input:** **>PF3D7_1464600** | | |  |  |  |  |  |  |  |  |  |
| --- | --- | --- | --- | --- | --- | --- | --- | --- | --- | --- | --- |
|  | [PF14_0614](http://string-db.org/newstring_cgi/display_single_node.pl?taskId=f_ZCMWe4_Ip_&node=399896&targetmode=proteins) | hypothetical protein (1502 aa) |  |  |  |  |  |  |  |  |  |
|  |  | *(Plasmodium falciparum)* |  |  |  |  |  |  |  |  |  |
| **Predicted Functional Partners:** | | |  |  |  |  |  |  |  |  |  |
|  | [PFL0300c](http://string-db.org/newstring_cgi/display_single_node.pl?taskId=f_ZCMWe4_Ip_&node=402045&targetmode=proteins) | phosphoesterase, putative (304 aa) |  |  |  |  |  |  | [**•**](http://string-db.org/newstring_cgi/show_textmining_evidence.pl?taskId=f_ZCMWe4_Ip_&node2=402045) |  | 0.688 |
|  | [PF14_0660](http://string-db.org/newstring_cgi/display_single_node.pl?taskId=f_ZCMWe4_Ip_&node=399944&targetmode=proteins) | hypothetical protein (358 aa) |  |  |  |  |  |  | [**•**](http://string-db.org/newstring_cgi/show_textmining_evidence.pl?taskId=f_ZCMWe4_Ip_&node2=399944) |  | 0.688 |
|  | [PF14_0064](http://string-db.org/newstring_cgi/display_single_node.pl?taskId=f_ZCMWe4_Ip_&node=399333&targetmode=proteins) | vacuolar protein sorting 29, putative (194 aa) |  |  |  |  |  |  | [**•**](http://string-db.org/newstring_cgi/show_textmining_evidence.pl?taskId=f_ZCMWe4_Ip_&node2=399333) |  | 0.688 |
|  | [PF13_0222](http://string-db.org/newstring_cgi/display_single_node.pl?taskId=f_ZCMWe4_Ip_&node=399135&targetmode=proteins) | RNA lariat debranching enzyme, putative (575 aa) |  |  |  |  |  |  | [**•**](http://string-db.org/newstring_cgi/show_textmining_evidence.pl?taskId=f_ZCMWe4_Ip_&node2=399135) |  | 0.688 |
|  | [PF14_0036](http://string-db.org/newstring_cgi/display_single_node.pl?taskId=f_ZCMWe4_Ip_&node=399305&targetmode=proteins) | acid phosphatase, putative (302 aa) |  |  |  |  |  |  | [**•**](http://string-db.org/newstring_cgi/show_textmining_evidence.pl?taskId=f_ZCMWe4_Ip_&node2=399305) |  | 0.683 |
|  | [PFI1360c](http://string-db.org/newstring_cgi/display_single_node.pl?taskId=f_ZCMWe4_Ip_&node=401886&targetmode=proteins) | serine%2Fthreonine protein phosphatase, putative (312 aa) |  |  |  |  |  |  | [**•**](http://string-db.org/newstring_cgi/show_textmining_evidence.pl?taskId=f_ZCMWe4_Ip_&node2=401886) |  | 0.651 |
|  | [PFC0595c](http://string-db.org/newstring_cgi/display_single_node.pl?taskId=f_ZCMWe4_Ip_&node=400594&targetmode=proteins) | serine%2Fthreonine protein phosphatase, putative (308 aa) |  |  |  |  |  |  | [**•**](http://string-db.org/newstring_cgi/show_textmining_evidence.pl?taskId=f_ZCMWe4_Ip_&node2=400594) |  | 0.651 |
|  | [PP1](http://string-db.org/newstring_cgi/display_single_node.pl?taskId=f_ZCMWe4_Ip_&node=399412&targetmode=proteins) | serine%2Fthreonine protein phosphatase, putative (304 aa) |  |  |  |  |  |  | [**•**](http://string-db.org/newstring_cgi/show_textmining_evidence.pl?taskId=f_ZCMWe4_Ip_&node2=399412) |  | 0.651 |
|  | [PF08_0129](http://string-db.org/newstring_cgi/display_single_node.pl?taskId=f_ZCMWe4_Ip_&node=397966&targetmode=proteins) | protein phosphatase, putative (604 aa) |  |  |  |  |  |  | [**•**](http://string-db.org/newstring_cgi/show_textmining_evidence.pl?taskId=f_ZCMWe4_Ip_&node2=397966) |  | 0.651 |
|  | [PFI1245c](http://string-db.org/newstring_cgi/display_single_node.pl?taskId=f_ZCMWe4_Ip_&node=401862&targetmode=proteins) | Protein phosphatase-beta (466 aa) |  |  |  |  |  |  | [**•**](http://string-db.org/newstring_cgi/show_textmining_evidence.pl?taskId=f_ZCMWe4_Ip_&node2=401862) |  | 0.649 |
|  | [PF14_0630](http://string-db.org/newstring_cgi/display_single_node.pl?taskId=f_ZCMWe4_Ip_&node=399913&targetmode=proteins) | protein serine%2Fthreonine phosphatase (889 aa) |  |  |  |  |  |  | [**•**](http://string-db.org/newstring_cgi/show_textmining_evidence.pl?taskId=f_ZCMWe4_Ip_&node2=399913) |  | 0.649 |
|  | [PP7](http://string-db.org/newstring_cgi/display_single_node.pl?taskId=f_ZCMWe4_Ip_&node=399495&targetmode=proteins) | PP1-like protein serine%2Fthreonine phosphatase (959 aa) |  |  |  |  |  |  | [**•**](http://string-db.org/newstring_cgi/show_textmining_evidence.pl?taskId=f_ZCMWe4_Ip_&node2=399495) |  | 0.649 |
|  | [PfPP5](http://string-db.org/newstring_cgi/display_single_node.pl?taskId=f_ZCMWe4_Ip_&node=397148&targetmode=proteins) | serine%2Fthreonine protein phosphatase pfPp5 (658 aa) |  |  |  |  |  |  | [**•**](http://string-db.org/newstring_cgi/show_textmining_evidence.pl?taskId=f_ZCMWe4_Ip_&node2=397148) |  | 0.595 |
|  | [PFL0320w](http://string-db.org/newstring_cgi/display_single_node.pl?taskId=f_ZCMWe4_Ip_&node=402049&targetmode=proteins) | hypothetical protein, conserved (346 aa) |  |  |  |  |  |  | [**•**](http://string-db.org/newstring_cgi/show_textmining_evidence.pl?taskId=f_ZCMWe4_Ip_&node2=402049) |  | 0.481 |
|  | [PF13_0027](http://string-db.org/newstring_cgi/display_single_node.pl?taskId=f_ZCMWe4_Ip_&node=398952&targetmode=proteins) | hypothetical protein, conserved (771 aa) |  |  |  |  |  |  | [**•**](http://string-db.org/newstring_cgi/show_textmining_evidence.pl?taskId=f_ZCMWe4_Ip_&node2=398952) |  | 0.481 |
|  | [MAL13P1.168-1](http://string-db.org/newstring_cgi/display_single_node.pl?taskId=f_ZCMWe4_Ip_&node=397036&targetmode=proteins) | hypothetical protein, conserved (267 aa) |  |  |  |  |  |  | [**•**](http://string-db.org/newstring_cgi/show_textmining_evidence.pl?taskId=f_ZCMWe4_Ip_&node2=397036) |  | 0.481 |
|  | [PF14_0525](http://string-db.org/newstring_cgi/display_single_node.pl?taskId=f_ZCMWe4_Ip_&node=399804&targetmode=proteins) | hypothetical protein (89 aa) |  |  |  |  |  |  | [**•**](http://string-db.org/newstring_cgi/show_textmining_evidence.pl?taskId=f_ZCMWe4_Ip_&node2=399804) |  | 0.480 |
|  | [PF11_0281](http://string-db.org/newstring_cgi/display_single_node.pl?taskId=f_ZCMWe4_Ip_&node=398671&targetmode=proteins) | hypothetical protein (247 aa) |  |  |  |  |  |  | [**•**](http://string-db.org/newstring_cgi/show_textmining_evidence.pl?taskId=f_ZCMWe4_Ip_&node2=398671) |  | 0.480 |
|  | [MAL13P1.275-1](http://string-db.org/newstring_cgi/display_single_node.pl?taskId=f_ZCMWe4_Ip_&node=397149&targetmode=proteins) | NLI interacting factor-like phosphatase, putative (1288 aa) |  |  |  |  |  |  | [**•**](http://string-db.org/newstring_cgi/show_textmining_evidence.pl?taskId=f_ZCMWe4_Ip_&node2=397149) |  | 0.480 |
|  | [PFL2365w](http://string-db.org/newstring_cgi/display_single_node.pl?taskId=f_ZCMWe4_Ip_&node=402468&targetmode=proteins) | hypothetical protein, conserved (1027 aa) |  |  |  |  |  |  | [**•**](http://string-db.org/newstring_cgi/show_textmining_evidence.pl?taskId=f_ZCMWe4_Ip_&node2=402468) |  | 0.420 |

60.

| **Your Input:** **>PF3D7_1466100** | | |  |  |  |  |  |  |  |  |  |
| --- | --- | --- | --- | --- | --- | --- | --- | --- | --- | --- | --- |
|  | [PF14_0630](http://string-db.org/newstring_cgi/display_single_node.pl?taskId=fs3TMFW_kL3X&node=399913&targetmode=proteins) | protein serine%2Fthreonine phosphatase (889 aa) |  |  |  |  |  |  |  |  |  |
|  |  | *(Plasmodium falciparum)* |  |  |  |  |  |  |  |  |  |
| **Predicted Functional Partners:** | | |  |  |  |  |  |  |  |  |  |
|  | [PF14_0064](http://string-db.org/newstring_cgi/display_single_node.pl?taskId=fs3TMFW_kL3X&node=399333&targetmode=proteins) | vacuolar protein sorting 29, putative (194 aa) |  |  |  | [**•**](http://string-db.org/newstring_cgi/show_coexpression_evidence.pl?taskId=fs3TMFW_kL3X&node2=399333) |  |  | [**•**](http://string-db.org/newstring_cgi/show_textmining_evidence.pl?taskId=fs3TMFW_kL3X&node2=399333) |  | 0.668 |
|  | [PF14_0660](http://string-db.org/newstring_cgi/display_single_node.pl?taskId=fs3TMFW_kL3X&node=399944&targetmode=proteins) | hypothetical protein (358 aa) |  |  |  | [**•**](http://string-db.org/newstring_cgi/show_coexpression_evidence.pl?taskId=fs3TMFW_kL3X&node2=399944) |  |  | [**•**](http://string-db.org/newstring_cgi/show_textmining_evidence.pl?taskId=fs3TMFW_kL3X&node2=399944) |  | 0.663 |
|  | [PFL0300c](http://string-db.org/newstring_cgi/display_single_node.pl?taskId=fs3TMFW_kL3X&node=402045&targetmode=proteins) | phosphoesterase, putative (304 aa) |  |  |  | [**•**](http://string-db.org/newstring_cgi/show_coexpression_evidence.pl?taskId=fs3TMFW_kL3X&node2=402045) |  |  | [**•**](http://string-db.org/newstring_cgi/show_textmining_evidence.pl?taskId=fs3TMFW_kL3X&node2=402045) |  | 0.661 |
|  | [PF14_0614](http://string-db.org/newstring_cgi/display_single_node.pl?taskId=fs3TMFW_kL3X&node=399896&targetmode=proteins) | hypothetical protein (1502 aa) |  |  |  |  |  |  | [**•**](http://string-db.org/newstring_cgi/show_textmining_evidence.pl?taskId=fs3TMFW_kL3X&node2=399896) |  | 0.649 |
|  | [PF13_0222](http://string-db.org/newstring_cgi/display_single_node.pl?taskId=fs3TMFW_kL3X&node=399135&targetmode=proteins) | RNA lariat debranching enzyme, putative (575 aa) |  |  |  |  |  |  | [**•**](http://string-db.org/newstring_cgi/show_textmining_evidence.pl?taskId=fs3TMFW_kL3X&node2=399135) |  | 0.649 |
|  | [vapA](http://string-db.org/newstring_cgi/display_single_node.pl?taskId=fs3TMFW_kL3X&node=398988&targetmode=proteins) | vacuolar ATP synthase, catalytic subunit a; Catalytic subunit of the peripheral V1 complex of v [...] (611 aa) |  |  |  |  |  |  | [**•**](http://string-db.org/newstring_cgi/show_textmining_evidence.pl?taskId=fs3TMFW_kL3X&node2=398988) |  | 0.647 |
|  | [PF14_0553](http://string-db.org/newstring_cgi/display_single_node.pl?taskId=fs3TMFW_kL3X&node=399832&targetmode=proteins) | cysteine proteinase falcipain-1; Probably degrades erythrocyte hemoglobin (569 aa) |  |  |  |  |  |  | [**•**](http://string-db.org/newstring_cgi/show_textmining_evidence.pl?taskId=fs3TMFW_kL3X&node2=399832) |  | 0.643 |
|  | [PFE0465c](http://string-db.org/newstring_cgi/display_single_node.pl?taskId=fs3TMFW_kL3X&node=401052&targetmode=proteins) | RNA polymerase I (2914 aa) |  |  |  |  |  |  | [**•**](http://string-db.org/newstring_cgi/show_textmining_evidence.pl?taskId=fs3TMFW_kL3X&node2=401052) |  | 0.643 |
|  | [PfEF-1beta](http://string-db.org/newstring_cgi/display_single_node.pl?taskId=fs3TMFW_kL3X&node=401740&targetmode=proteins) | EF-1B (276 aa) |  |  |  |  |  |  | [**•**](http://string-db.org/newstring_cgi/show_textmining_evidence.pl?taskId=fs3TMFW_kL3X&node2=401740) |  | 0.593 |
|  | [PFD0770c](http://string-db.org/newstring_cgi/display_single_node.pl?taskId=fs3TMFW_kL3X&node=400856&targetmode=proteins) | ribosomal protein l15, putative (220 aa) |  |  |  |  |  |  | [**•**](http://string-db.org/newstring_cgi/show_textmining_evidence.pl?taskId=fs3TMFW_kL3X&node2=400856) |  | 0.585 |

61.

| **Your Input:** **>PF3D7_1469200** | | |  |  |  |  |  |  |  |  |  |
| --- | --- | --- | --- | --- | --- | --- | --- | --- | --- | --- | --- |
|  | [PF14_0660](http://string-db.org/newstring_cgi/display_single_node.pl?taskId=7NM7_03hXWas&node=399944&targetmode=proteins) | hypothetical protein (358 aa) |  |  |  |  |  |  |  |  |  |
|  |  | *(Plasmodium falciparum)* |  |  |  |  |  |  |  |  |  |
| **Predicted Functional Partners:** | | |  |  |  |  |  |  |  |  |  |
|  | [PP7](http://string-db.org/newstring_cgi/display_single_node.pl?taskId=7NM7_03hXWas&node=399495&targetmode=proteins) | PP1-like protein serine%2Fthreonine phosphatase (959 aa) |  |  |  | [**•**](http://string-db.org/newstring_cgi/show_coexpression_evidence.pl?taskId=7NM7_03hXWas&node2=399495) |  |  | [**•**](http://string-db.org/newstring_cgi/show_textmining_evidence.pl?taskId=7NM7_03hXWas&node2=399495) | **•** | 0.876 |
|  | [MAL8P1.150](http://string-db.org/newstring_cgi/display_single_node.pl?taskId=7NM7_03hXWas&node=397573&targetmode=proteins) | hypothetical protein, conserved (2166 aa) |  |  |  | [**•**](http://string-db.org/newstring_cgi/show_coexpression_evidence.pl?taskId=7NM7_03hXWas&node2=397573) |  |  |  |  | 0.851 |
|  | [MAL13P1.278-1](http://string-db.org/newstring_cgi/display_single_node.pl?taskId=7NM7_03hXWas&node=397152&targetmode=proteins) | Ser%2FThr protein kinase (4044 aa) |  |  |  | [**•**](http://string-db.org/newstring_cgi/show_coexpression_evidence.pl?taskId=7NM7_03hXWas&node2=397152) |  |  |  |  | 0.847 |
|  | [PFD1100c](http://string-db.org/newstring_cgi/display_single_node.pl?taskId=7NM7_03hXWas&node=400929&targetmode=proteins) | hypothetical protein, conserved (476 aa) |  |  |  | [**•**](http://string-db.org/newstring_cgi/show_coexpression_evidence.pl?taskId=7NM7_03hXWas&node2=400929) |  |  |  |  | 0.831 |
|  | [PF10_0138](http://string-db.org/newstring_cgi/display_single_node.pl?taskId=7NM7_03hXWas&node=398122&targetmode=proteins) | hypothetical protein (1455 aa) |  |  |  | [**•**](http://string-db.org/newstring_cgi/show_coexpression_evidence.pl?taskId=7NM7_03hXWas&node2=398122) |  |  |  |  | 0.829 |
|  | [PF08_0129](http://string-db.org/newstring_cgi/display_single_node.pl?taskId=7NM7_03hXWas&node=397966&targetmode=proteins) | protein phosphatase, putative (604 aa) |  |  |  | [**•**](http://string-db.org/newstring_cgi/show_coexpression_evidence.pl?taskId=7NM7_03hXWas&node2=397966) |  |  | [**•**](http://string-db.org/newstring_cgi/show_textmining_evidence.pl?taskId=7NM7_03hXWas&node2=397966) |  | 0.788 |
|  | [PF11_0277](http://string-db.org/newstring_cgi/display_single_node.pl?taskId=7NM7_03hXWas&node=398667&targetmode=proteins) | hypothetical protein (350 aa) |  |  |  | [**•**](http://string-db.org/newstring_cgi/show_coexpression_evidence.pl?taskId=7NM7_03hXWas&node2=398667) |  |  |  |  | 0.788 |
|  | [PF14_0173-1](http://string-db.org/newstring_cgi/display_single_node.pl?taskId=7NM7_03hXWas&node=399443&targetmode=proteins) | hypothetical protein (838 aa) |  |  |  | [**•**](http://string-db.org/newstring_cgi/show_coexpression_evidence.pl?taskId=7NM7_03hXWas&node2=399443) |  |  |  |  | 0.780 |
|  | [PFL1435c](http://string-db.org/newstring_cgi/display_single_node.pl?taskId=7NM7_03hXWas&node=402278&targetmode=proteins) | myosin d (2231 aa) |  |  |  | [**•**](http://string-db.org/newstring_cgi/show_coexpression_evidence.pl?taskId=7NM7_03hXWas&node2=402278) |  |  |  |  | 0.774 |
|  | [PF13_0090](http://string-db.org/newstring_cgi/display_single_node.pl?taskId=7NM7_03hXWas&node=399013&targetmode=proteins) | ADP-ribosylation factor, putative (224 aa) |  |  |  | [**•**](http://string-db.org/newstring_cgi/show_coexpression_evidence.pl?taskId=7NM7_03hXWas&node2=399013) |  |  |  |  | 0.772 |
|  | [PF11_0464](http://string-db.org/newstring_cgi/display_single_node.pl?taskId=7NM7_03hXWas&node=398854&targetmode=proteins) | serine%2Fthreonine protein kinase (2075 aa) |  |  |  | [**•**](http://string-db.org/newstring_cgi/show_coexpression_evidence.pl?taskId=7NM7_03hXWas&node2=398854) |  |  |  |  | 0.772 |
|  | [PF11_0060](http://string-db.org/newstring_cgi/display_single_node.pl?taskId=7NM7_03hXWas&node=398454&targetmode=proteins) | protein kinase (1620 aa) |  |  |  | [**•**](http://string-db.org/newstring_cgi/show_coexpression_evidence.pl?taskId=7NM7_03hXWas&node2=398454) |  |  |  |  | 0.770 |
|  | [PF10_0348](http://string-db.org/newstring_cgi/display_single_node.pl?taskId=7NM7_03hXWas&node=398344&targetmode=proteins) | Erythrocyte membrane protein, putative (697 aa) |  |  |  | [**•**](http://string-db.org/newstring_cgi/show_coexpression_evidence.pl?taskId=7NM7_03hXWas&node2=398344) |  |  |  |  | 0.770 |
|  | [PF10_0156](http://string-db.org/newstring_cgi/display_single_node.pl?taskId=7NM7_03hXWas&node=398141&targetmode=proteins) | hypothetical protein (453 aa) |  |  |  | [**•**](http://string-db.org/newstring_cgi/show_coexpression_evidence.pl?taskId=7NM7_03hXWas&node2=398141) |  |  |  |  | 0.770 |
|  | [ASP](http://string-db.org/newstring_cgi/display_single_node.pl?taskId=7NM7_03hXWas&node=400760&targetmode=proteins) | apical sushi protein, ASP (731 aa) |  |  |  | [**•**](http://string-db.org/newstring_cgi/show_coexpression_evidence.pl?taskId=7NM7_03hXWas&node2=400760) |  |  |  |  | 0.770 |
|  | [PF10_0094](http://string-db.org/newstring_cgi/display_single_node.pl?taskId=7NM7_03hXWas&node=398078&targetmode=proteins) | tubulin-tyrosine ligase, putative (553 aa) |  |  |  | [**•**](http://string-db.org/newstring_cgi/show_coexpression_evidence.pl?taskId=7NM7_03hXWas&node2=398078) |  |  |  |  | 0.770 |
|  | [RON6](http://string-db.org/newstring_cgi/display_single_node.pl?taskId=7NM7_03hXWas&node=400367&targetmode=proteins) | hypothetical protein (951 aa) |  |  |  | [**•**](http://string-db.org/newstring_cgi/show_coexpression_evidence.pl?taskId=7NM7_03hXWas&node2=400367) |  |  |  |  | 0.764 |
|  | [PF13_0326](http://string-db.org/newstring_cgi/display_single_node.pl?taskId=7NM7_03hXWas&node=399234&targetmode=proteins) | actin-depolymerizing factor, putative; Not involved in actin polymerisation, instead functions [...] (143 aa) |  |  |  | [**•**](http://string-db.org/newstring_cgi/show_coexpression_evidence.pl?taskId=7NM7_03hXWas&node2=399234) |  |  |  |  | 0.760 |
|  | [CPK4](http://string-db.org/newstring_cgi/display_single_node.pl?taskId=7NM7_03hXWas&node=397774&targetmode=proteins) | calcium-dependent protein kinase 4; Calcium-dependent kinase that plays a central role in the s [...] (528 aa) |  |  |  | [**•**](http://string-db.org/newstring_cgi/show_coexpression_evidence.pl?taskId=7NM7_03hXWas&node2=397774) |  |  |  |  | 0.760 |
|  | [MAL7P1.119](http://string-db.org/newstring_cgi/display_single_node.pl?taskId=7NM7_03hXWas&node=397358&targetmode=proteins) | hypothetical protein, conserved (749 aa) |  |  |  | [**•**](http://string-db.org/newstring_cgi/show_coexpression_evidence.pl?taskId=7NM7_03hXWas&node2=397358) |  |  |  |  | 0.756 |
