# Supplementary figures and images for: Genome wide in silico analysis of Plasmodium falciparum phosphatome
Source: BMC Genomics. 2014 Nov 25;15:1024. doi: 10.1186/1471-2164-15-1024 (PMC4256932; doi:10.1186/1471-2164-15-1024)

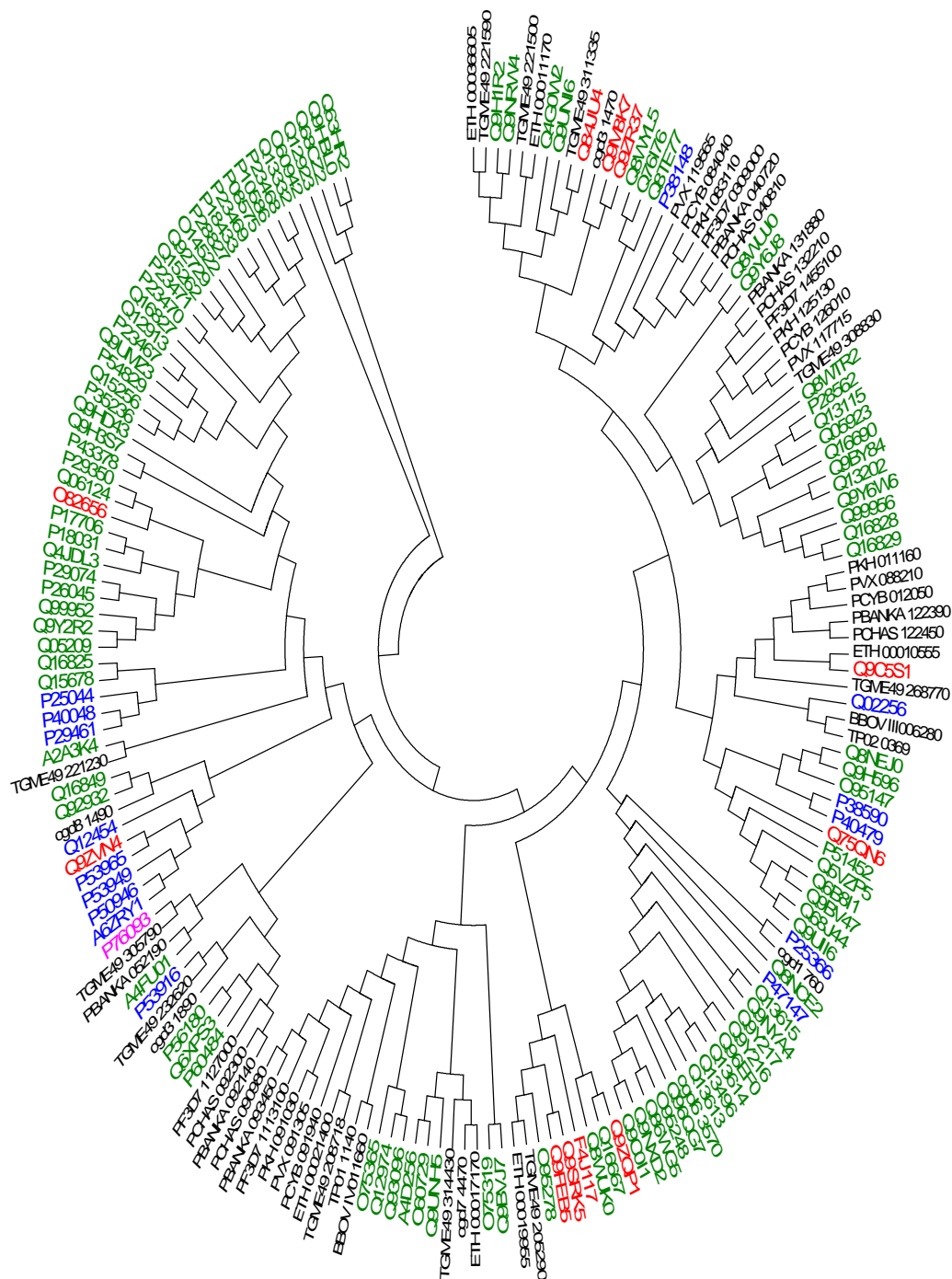

Supplement: Supplementary file 9 — Additional file 9:Phylogenetic analysis for PTPc domain superfamily. H. sapiens (green), S. cerevisiae (blue), A. thaliana (red) and P. falciparum (ID PF3D7), P. berghei (PBANKA), P. vivax (PVX), P. chabaudi chabaudi (PCHAS), P. cynomolgi (PCYB), P. knowlesi (PKH), T. gondii (TGME49), and E. tenella (ETH), B. bovis (BBOV), T. parva (TP), C. parvam (cgd) is used to perform evolutionary analysis. MEGA software is used to perform Phylogenetic analysis. Sequence alignment is performed using Clustal X and Muscle. NJ method is used to generate the phylogenetic tree. (PDF 98 KB) [file 12864_2014_6717_MOESM9_ESM.pdf]

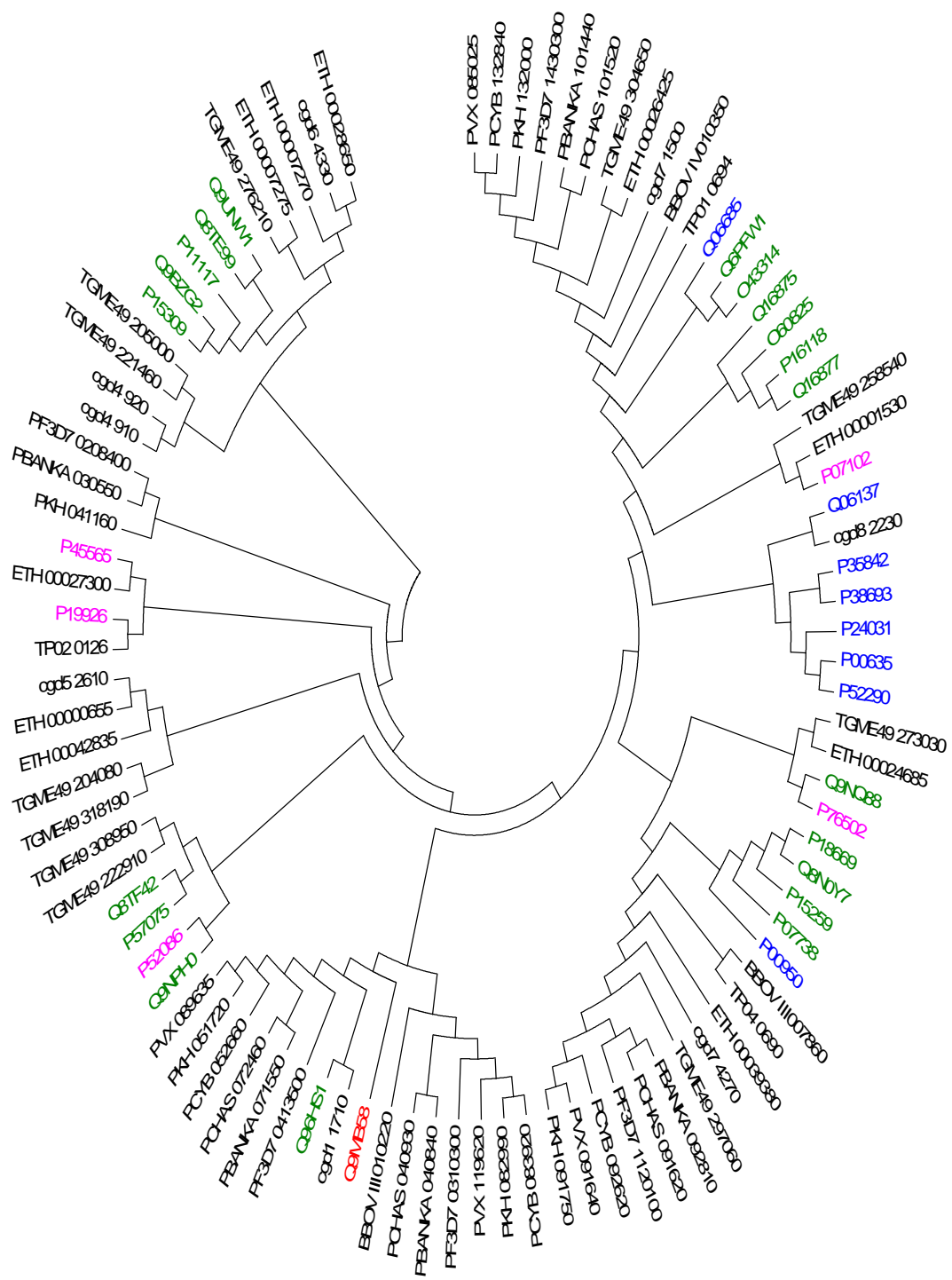

Supplement: Supplementary file 10 — Additional file 10:Phylogenetic analysis for HP domain superfamily. H. sapiens (green), E.coli (pink), S. cerevisiae (blue), A. thaliana (red), P. falciparum (PF3D7), P. berghei (PBANKA), P. vivax (PVX), P. chabaudi chabaudi (PCHAS), P. cynomolgi (PCYB), P. knowlesi (PKH), T. gondii (TGME49), and E. tenella (ETH), B. bovis (BBOV), T. parva (TP), C. parvam (cgd) is used to perform evolutionary analysis. MEGA software is used to perform Phylogenetic analysis. Sequence alignment is performed using Clustal X and Muscle. NJ method is used to generate the phylogenetic tree. (PDF 68 KB) [file 12864_2014_6717_MOESM10_ESM.pdf]

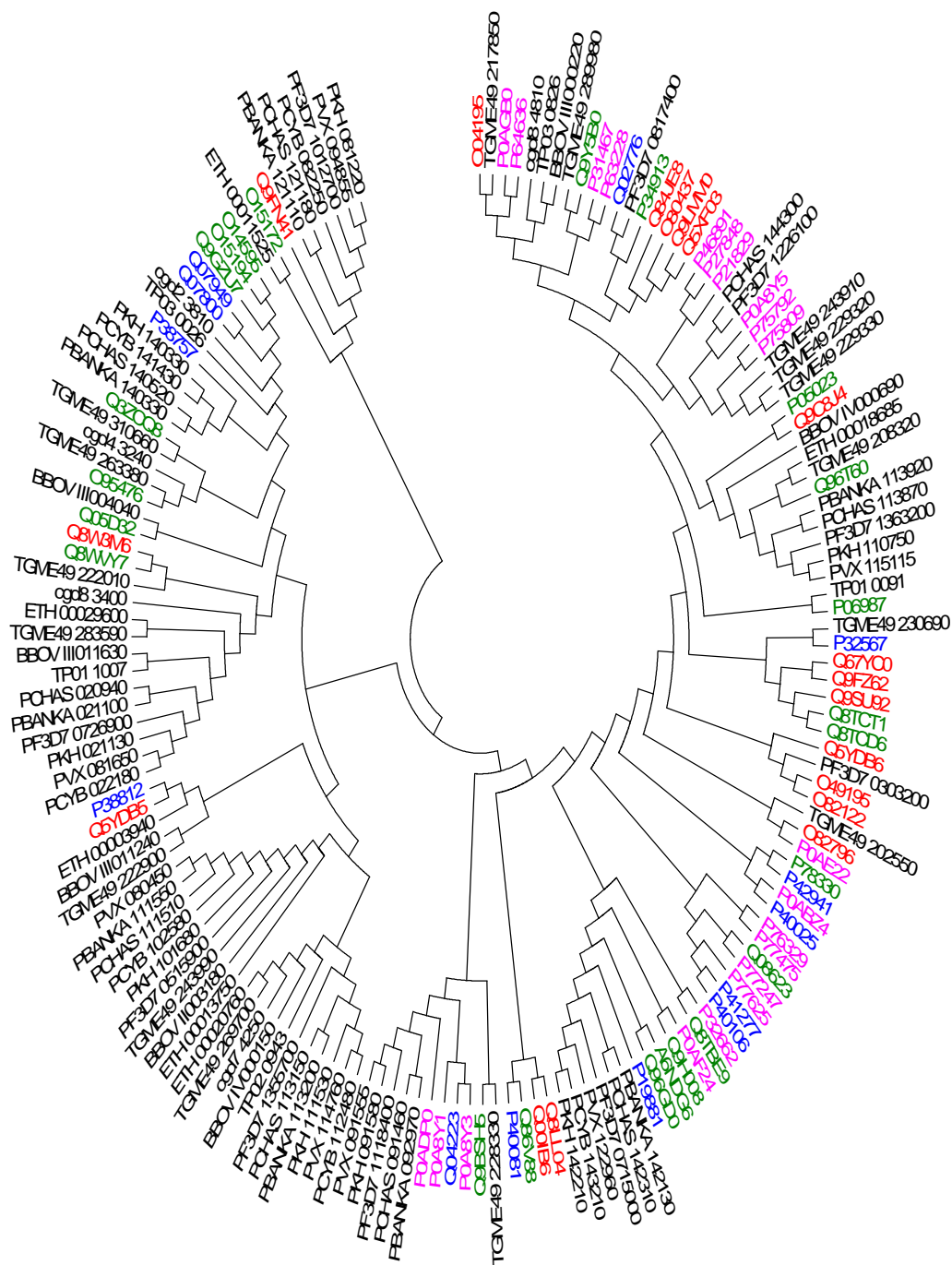

Supplement: Supplementary file 11 — Additional file 11:Phylogenetic analysis for HAD domain superfamily. H. sapiens (green), E.coli (pink), S. cerevisiae (blue), A. thaliana (red), P. falciparum (PF3D7), P. berghei (PBANKA), P. vivax (PVX), P. chabaudi chabaudi (PCHAS), P. cynomolgi (PCYB), P. knowlesi (PKH), T. gondii (TGME49), and E. tenella (ETH), B. bovis (BBOV), T. parva (TP), C. parvam (cgd) is used to perform evolutionary analysis. MEGA software is used to perform Phylogenetic analysis. Sequence alignment is performed using Clustal X and Muscle. NJ method is used to generate the phylogenetic tree. (PDF 86 KB) [file 12864_2014_6717_MOESM11_ESM.pdf]

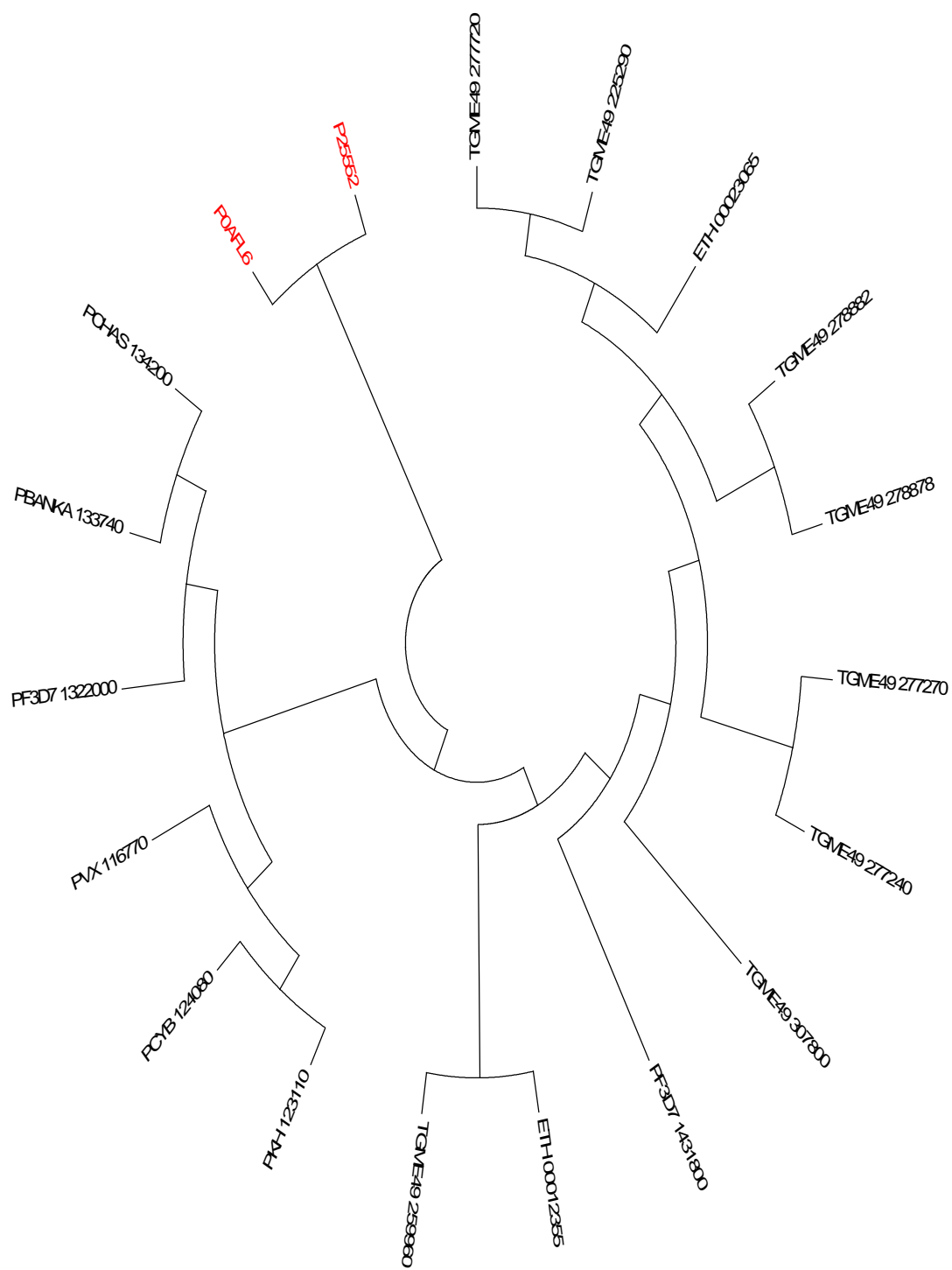

Supplement: Supplementary file 12 — Additional file 12:Phylogenetic analysis for Nucleoside Phosphatase domain superfamily. E.coli (red), P. falciparum (PF3D7), P. berghei (PBANKA), P. vivax (PVX), P. chabaudi chabaudi (PCHAS), P. cynomolgi (PCYB), P. knowlesi (PKH), T. gondii (TGME49), and E. tenella (ETH), B. bovis (BBOV), T. parva (TP), C. parvam (cgd) is used to perform evolutionary analysis. MEGA software is used to perform Phylogenetic analysis. Sequence alignment is performed using Clustal X and Muscle. NJ method is used to generate the phylogenetic tree. (PDF 38 KB) [file 12864_2014_6717_MOESM12_ESM.pdf]

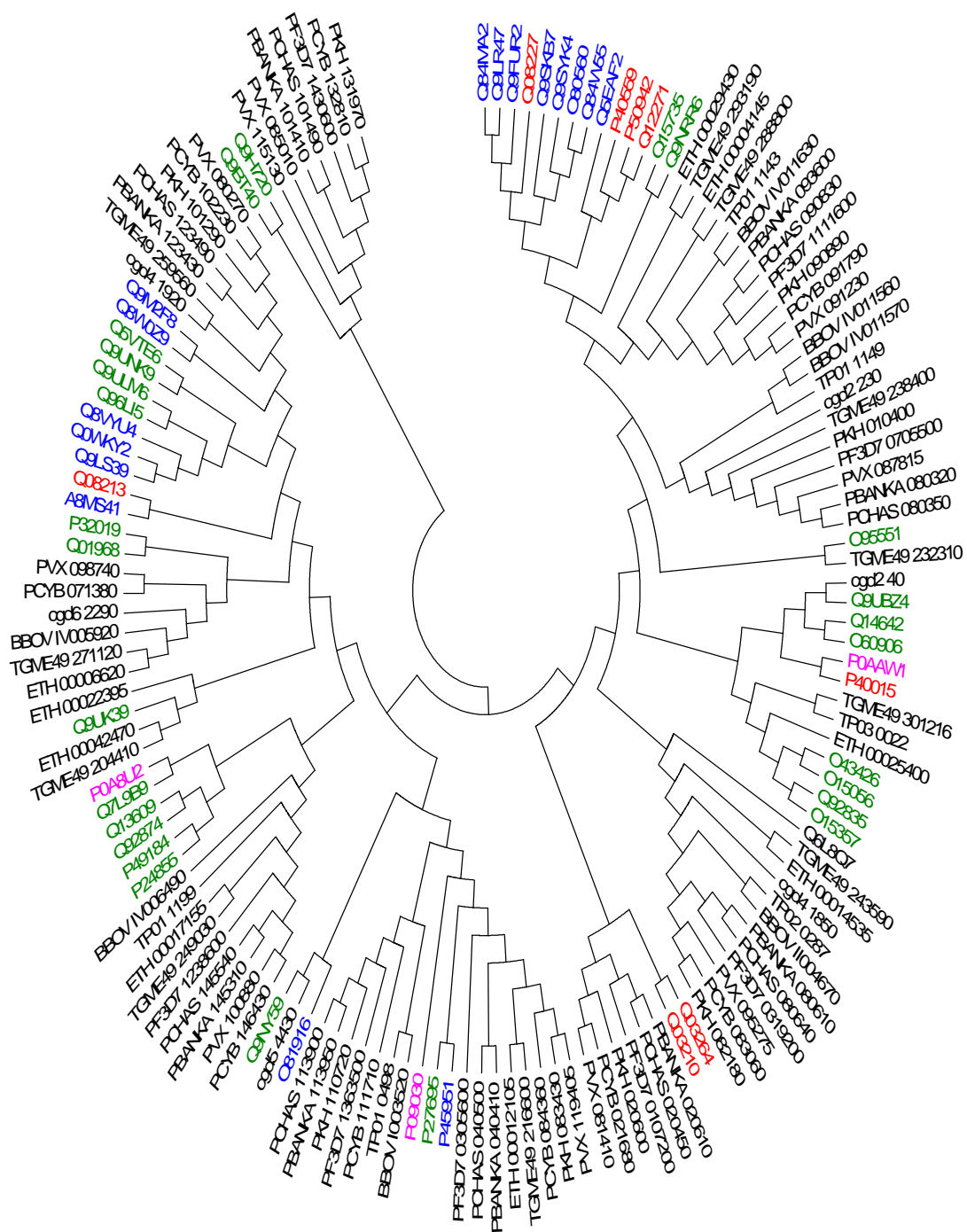

Supplement: Supplementary file 13 — Additional file 13:Phylogenetic analysis for EEP domain superfamily. H. sapiens (green), E.coli (pink), S. cerevisiae (red), A. thaliana (blue), P. falciparum (PF3D7), P. berghei (PBANKA), P. vivax (PVX), P. chabaudi chabaudi (PCHAS), P. cynomolgi (PCYB), P. knowlesi (PKH), T. gondii (TGME49), and E. tenella (ETH), B. bovis (BBOV), T. parva (TP), C. parvam (cgd) is used to perform evolutionary analysis. MEGA software is used to perform Phylogenetic analysis. Sequence alignment is performed using Clustal X and Muscle. NJ method is used to generate the phylogenetic tree. (PDF 90 KB) [file 12864_2014_6717_MOESM13_ESM.pdf]

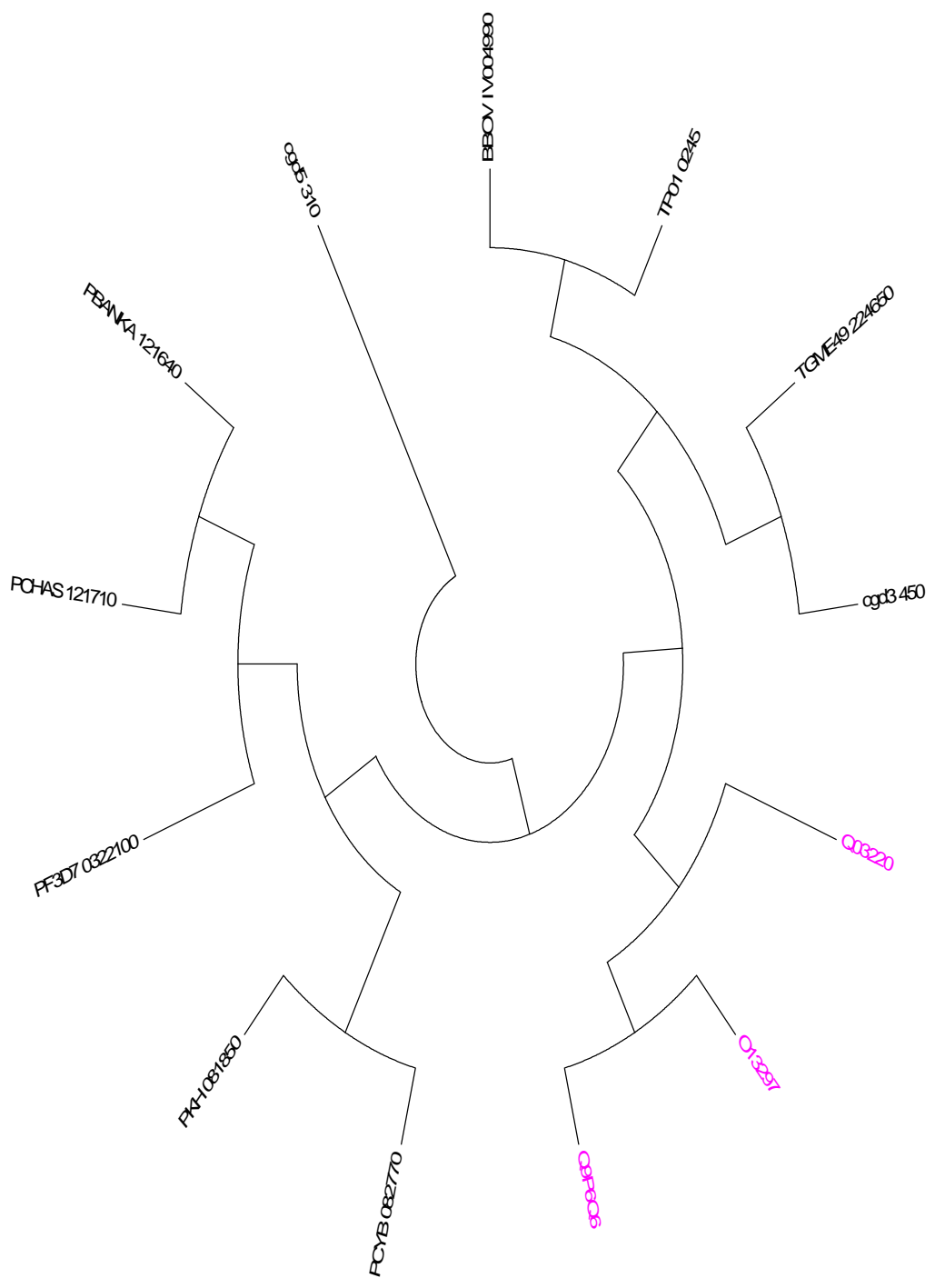

Supplement: Supplementary file 14 — Additional file 14:Phylogenetic analysis for CYTH_like_Pases domain superfamily. S. cerevisiae (pink), P. falciparum (PF3D7), P. berghei (PBANKA), P. chabaudi chabaudi (PCHAS), P. cynomolgi (PCYB), P. knowlesi (PKH) and T. gondii (TGME49), B. bovis (BBOV), T. parva (TP), C. parvam (cgd) is used to perform evolutionary analysis. MEGA software is used to perform Phylogenetic analysis. Sequence alignment is performed using Clustal X and Muscle. NJ method is used to generate the phylogenetic tree. (PDF 34 KB) [file 12864_2014_6717_MOESM14_ESM.pdf]

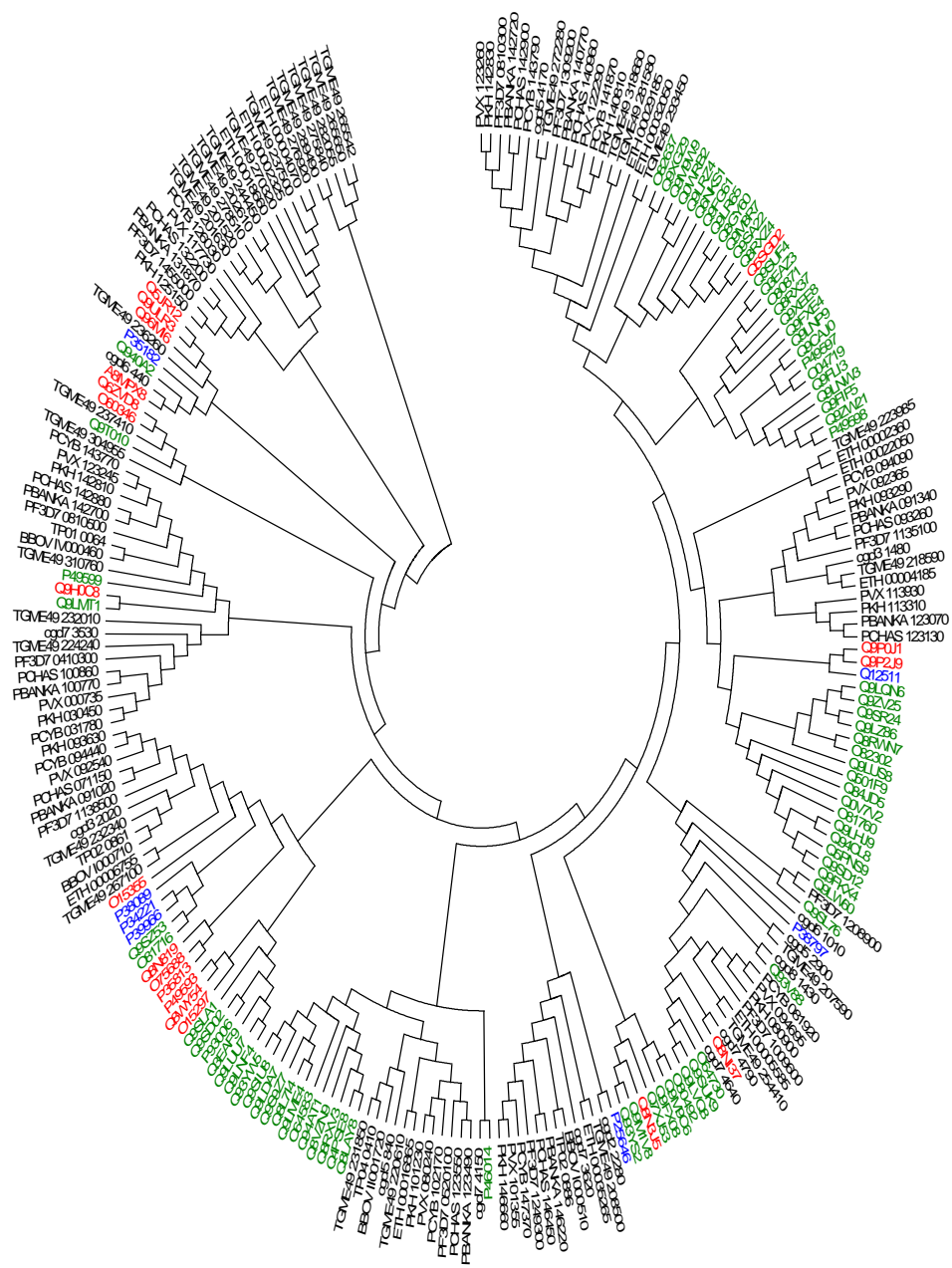

Supplement: Supplementary file 15 — Additional file 15:Phylogenetic analysis for PP2Cc domain superfamily. H. sapiens (red), S. cerevisiae (blue), A. thaliana (green), P. falciparum (PF3D7), P. berghei (PBANKA), P. vivax (PVX), P. chabaudi chabaudi (PCHAS), P. cynomolgi (PCYB), P. knowlesi (PKH), T. gondii (TGME49), and E. tenella (ETH), B. bovis (BBOV), T. parva (TP), C. parvam (cgd) is used to perform evolutionary analysis. MEGA software is used to perform Phylogenetic analysis. Sequence alignment is performed using Clustal X and Muscle. NJ method is used to generate the phylogenetic tree. (PDF 104 KB) [file 12864_2014_6717_MOESM15_ESM.pdf]

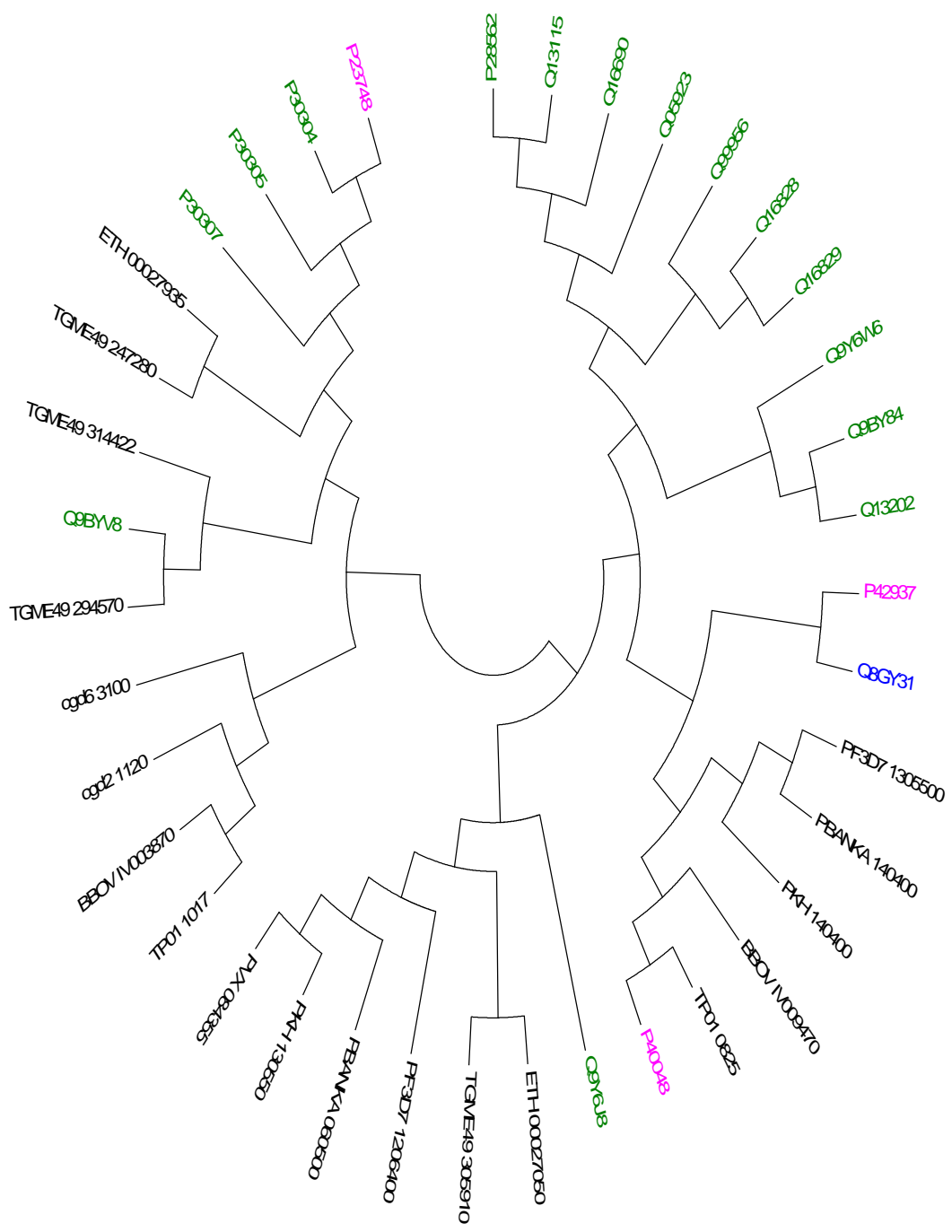

Supplement: Supplementary file 16 — Additional file 16:Phylogenetic analysis for RHOD domain superfamily. H. sapiens (green), S. cerevisiae (pink), A. thaliana (blue), P. falciparum (PF3D7), P. berghei (PBANKA), P. vivax (PVX), P. cynomolgi (PCYB), P. knowlesi (PKH), T. gondii (TGME49), and E. tenella (ETH), B. bovis (BBOV), T. parva (TP), C. parvam (cgd) is used to perform evolutionary analysis. MEGA software is used to perform Phylogenetic analysis. Sequence alignment is performed using Clustal X and Muscle. NJ method is used to generate the phylogenetic tree. (PDF 49 KB) [file 12864_2014_6717_MOESM16_ESM.pdf]

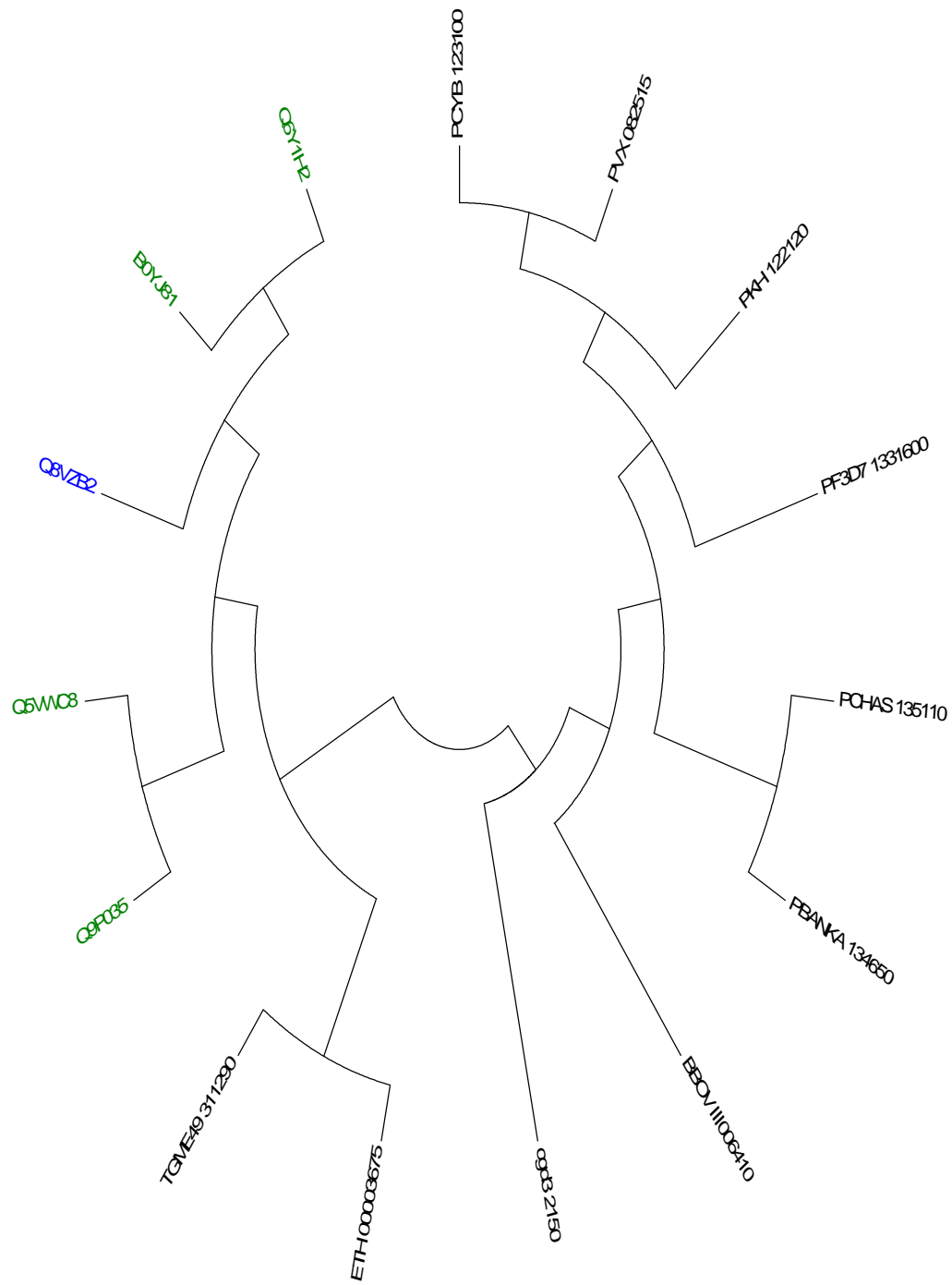

Supplement: Supplementary file 17 — Additional file 17:Phylogenetic analysis for PTPLA domain superfamily. H. sapiens (green), A. thaliana (blue), P. falciparum (PF3D7), P. berghei (PBANKA), P. vivax (PVX), P. chabaudi chabaudi (PCHAS), P. cynomolgi (PCYB), P. knowlesi (PKH), T. gondii (TGME49), and E. tenella (ETH), B. bovis (BBOV), C. parvam (cgd) is used to perform evolutionary analysis. MEGA software is used to perform Phylogenetic analysis. Sequence alignment is performed using Clustal X and Muscle. NJ method is used to generate the phylogenetic tree. (PDF 34 KB) [file 12864_2014_6717_MOESM17_ESM.pdf]

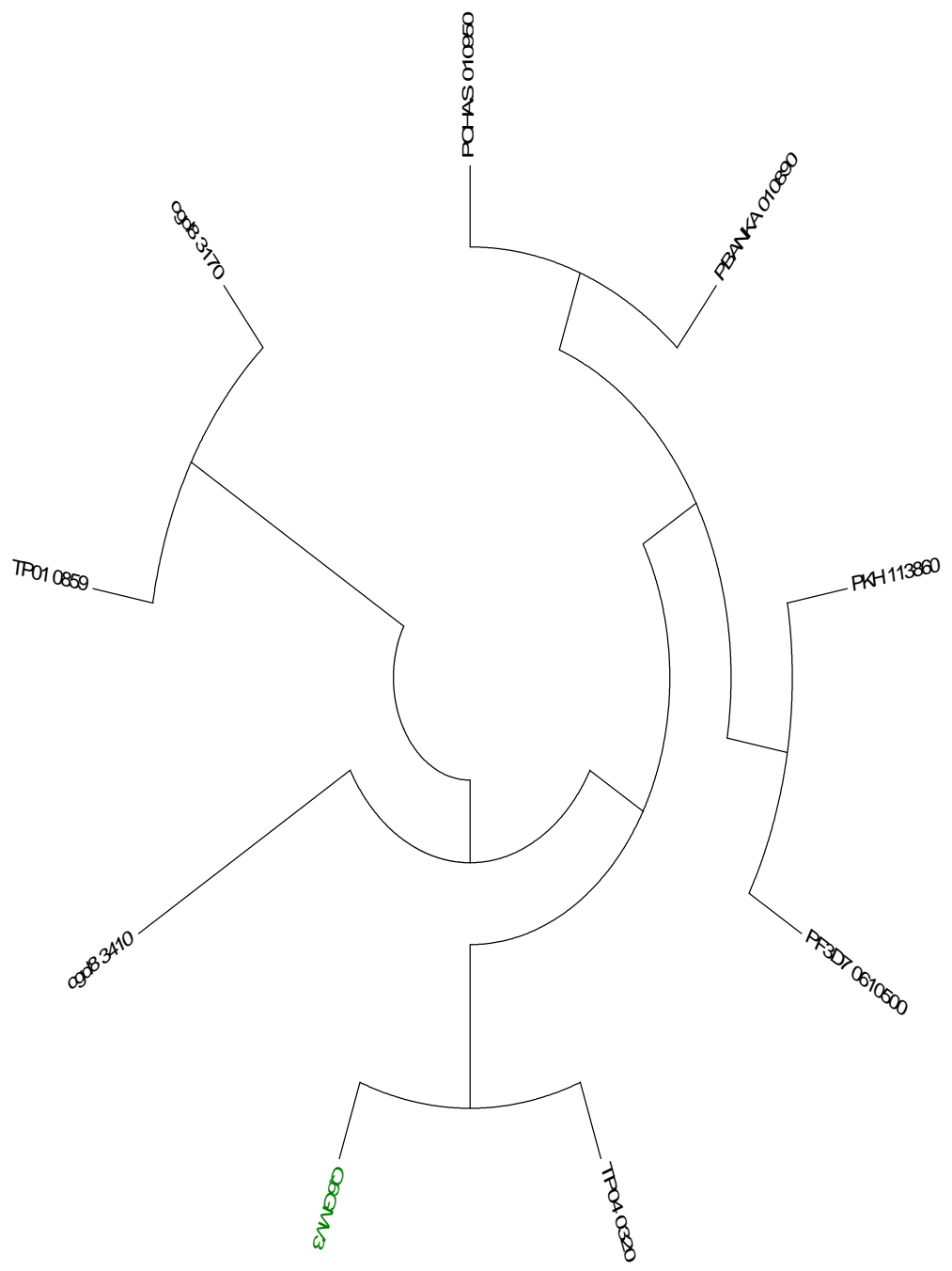

Supplement: Supplementary file 18 — Additional file 18:Phylogenetic analysis for PTH2 domain superfamily. H. sapiens (green), P. falciparum (ID PF3D7), P. berghei (PBANKA), P. chabaudi chabaudi (PCHAS) and P. knowlesi (PKH), B. bovis (BBOV), T. parva (TP), C. parvam (cgd) is used to perform evolutionary analysis. MEGA software is used to perform Phylogenetic analysis. Sequence alignment is performed using Clustal X and Muscle. NJ method is used to generate the phylogenetic tree. (PDF 28 KB) [file 12864_2014_6717_MOESM18_ESM.pdf]

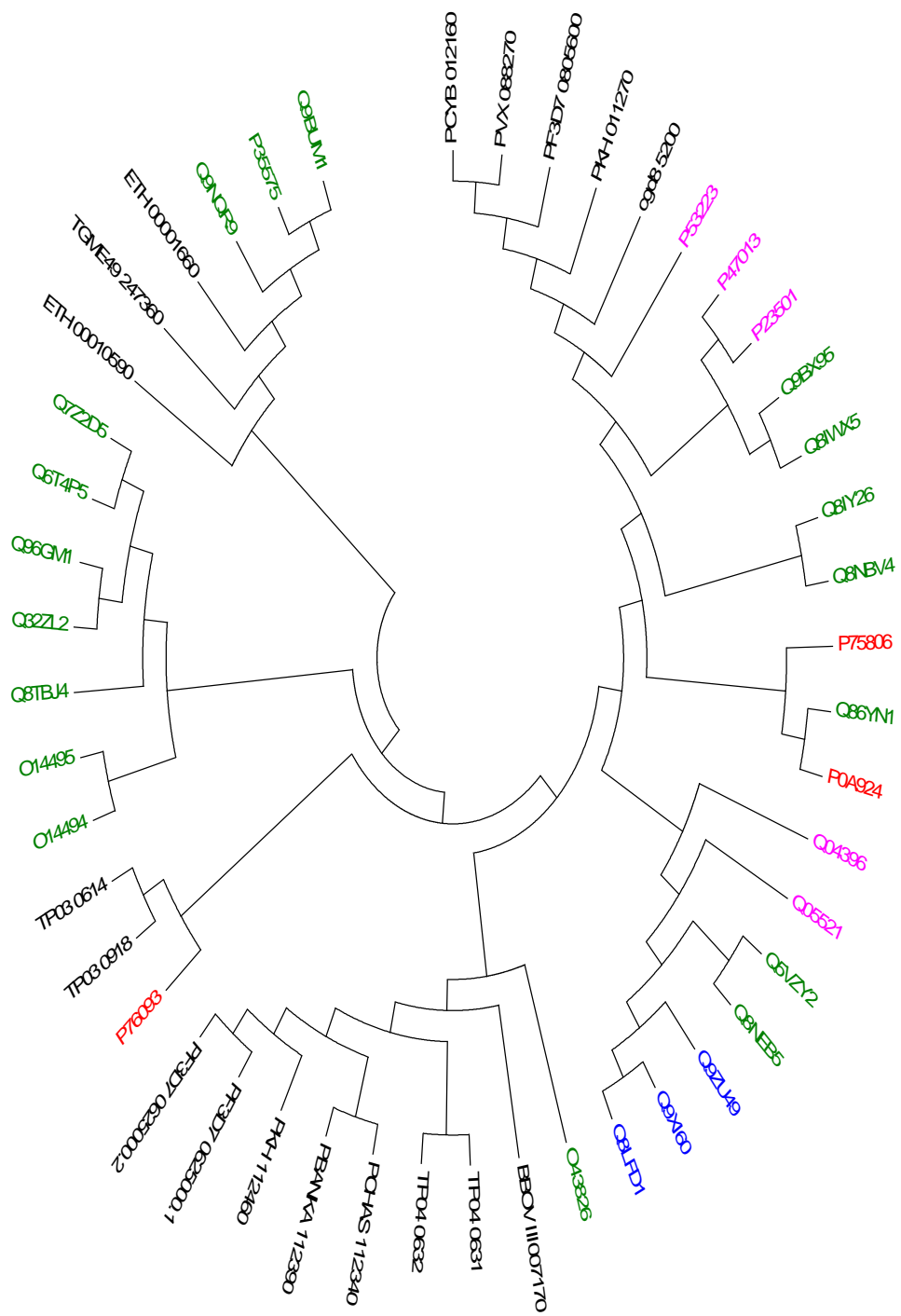

Supplement: Supplementary file 19 — Additional file 19:Phylogenetic analysis for PAP2 domain superfamily. H. sapiens (green), E.coli (red), S. cerevisiae (pink), A. thaliana (blue) and P. falciparum (PF3D7), P. berghei (PBANKA), P. vivax (PVX), P. chabaudi chabaudi (PCHAS), P. cynomolgi (PCYB), P. knowlesi (PKH), T. gondii (TGME49), and E. tenella (ETH), B. bovis (BBOV), T. parva (TP), C. parvam (cgd) is used to perform evolutionary analysis. MEGA software is used to perform Phylogenetic analysis. Sequence alignment is performed using Clustal X and Muscle. NJ method is used to generate the phylogenetic tree. (PDF 51 KB) [file 12864_2014_6717_MOESM19_ESM.pdf]

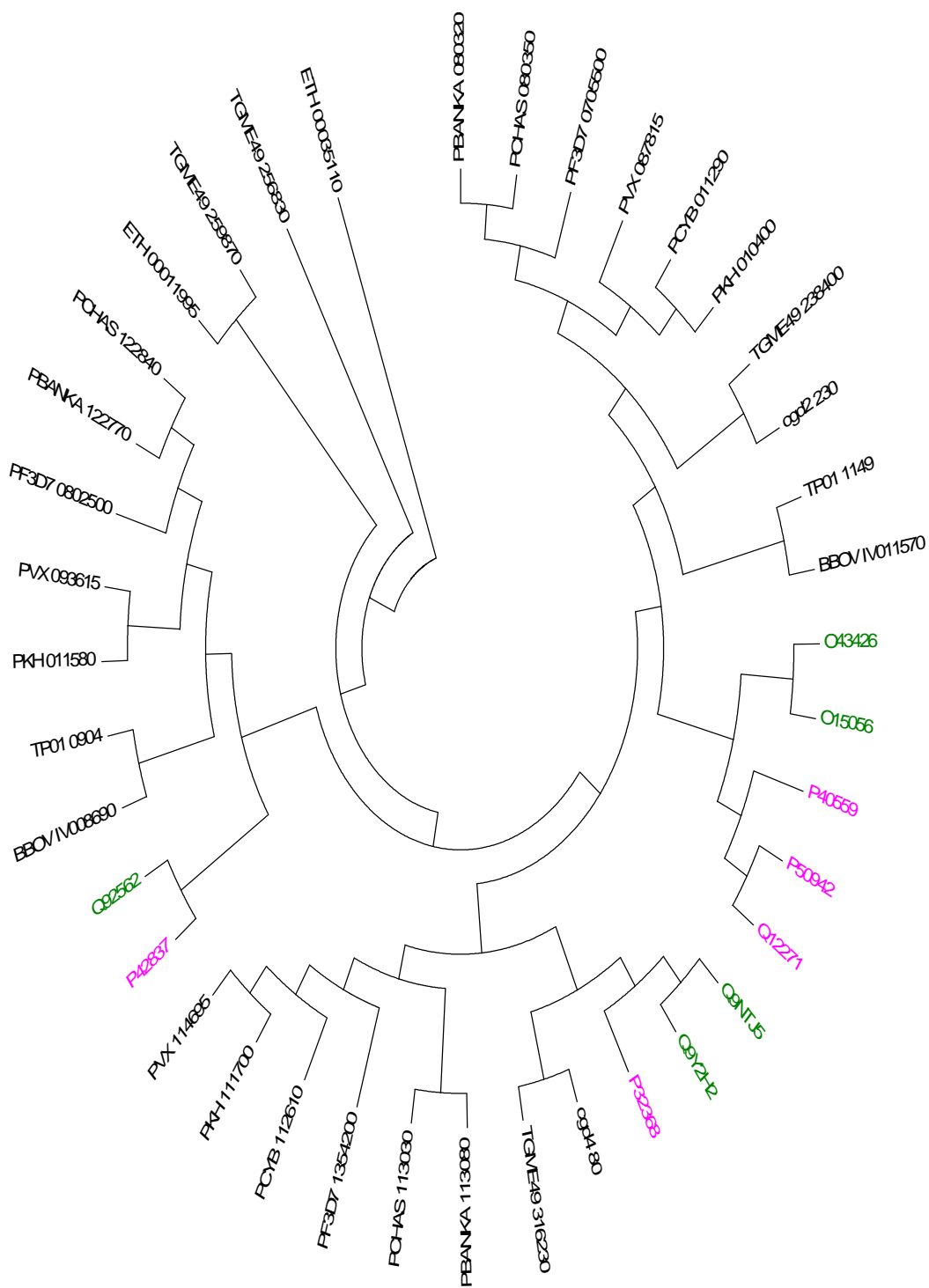

Supplement: Supplementary file 20 — Additional file 20:Phylogenetic analysis for Syja_N domain superfamily. H. sapiens (green), S. cerevisiae (pink), P. falciparum (PF3D7), P. berghei (PBANKA), P. vivax (PVX), P. chabaudi chabaudi (PCHAS), P. cynomolgi (PCYB), P. knowlesi (PKH), T. gondii (TGME49), and E. tenella (ETH), B. bovis (BBOV), T. parva (TP), C. parvam (cgd) is used to perform evolutionary analysis. MEGA software is used to perform Phylogenetic analysis. Sequence alignment is performed using Clustal X and Muscle. NJ method is used to generate the phylogenetic tree. (PDF 52 KB) [file 12864_2014_6717_MOESM20_ESM.pdf]
